# Opinions on integrating couple counselling and female sexual reproductive health services into Voluntary Medical Male Circumcision services in Lilongwe, Malawi

**DOI:** 10.1371/journal.pone.0273627

**Published:** 2022-09-09

**Authors:** Bertha Maseko, Agatha Bula, Simone Sasse, Annie Thom, Mercy Tsidya, Jennifer Tang, Mina C. Hosseinipour

**Affiliations:** 1 Malawi Liverpool Welcome Trust Clinical Research Programme, Blantyre, Malawi; 2 UNC Project-Malawi, Lilongwe, Malawi; 3 Department of Obstetrics and Gynecology, New York University, New York, New York, United States of America; 4 Department of Obstetrics and Gynecology, University of North Carolina at Chapel Hill, Chapel Hill, North Carolina, United States of America; 5 Department of Medicine, University of North Carolina at Chapel Hill, Chapel Hill, North Carolina, United States of America; University of Washington, UNITED STATES

## Abstract

**Background:**

Couples HIV Counselling and Testing (CHCT) has been found to be potentially beneficial than individual HIV Counselling and Testing for prevention and treatment of HIV. However, there are few health care opportunities for men and women to access health services together, leading to underutilization of CHCT service. Integrating female Sexual and Reproductive Health (SRH) services into male-dominated service could be more effective than trying to integrate men’s health services into female-dominated health services. A potential site for male-female service integration could be Voluntary Medical Male Circumcision (VMMC) centers.

**Methodology:**

We conducted a qualitative study in Lilongwe, Malawi between June to August 2018. Twenty VMMC clients, 20 peers and 20 VMMC providers completed individual in-depth interviews to share their opinions on what they thought about integrating CHCT and other SRH Services into VMMC services. These proposed SRH services include family planning, cervical cancer screening, sexually transmitted infection management and pre-exposure prophylaxis (PrEP). Content analysis was used to analyze the results.

**Results:**

All participants were receptive to integration of CHCT, and most accepted the integration of SRH services into VMMC Services. Most VMMC clients, peers and care providers said that CHCT integration would help couples to know their HIV status, prevent HIV transmission, encourage healthy relationships, and provide a chance for women to participate in VMMC counselling and wound care. However, integration of other services, such as family planning and cervical cancer screening, drew mixed opinions among participants. Most VMMC clients, peers and providers felt that integration of services would promote male involvement and increase men’s knowledge in feminine sexual reproductive health services. A few providers expressed concerns over service integration, citing reasons such as overcrowding, work overload, gender mixing, and lack of provider capacity and space. Most participants supported integrating PrEP with VMMC Services and felt that PrEP would complement VMMC in HIV prevention. Few providers, peers and VMMC clients felt the addition of PrEP to VMMC services would lead to high-risk sexual activity that would then increase the risk for HIV acquisition. A few participants recommended community sensitization when integrating some of sexual reproductive health services into VMMC Services to mitigate negative perceptions about VMMC services and encourage service uptake among couples

**Conclusion:**

Most participants service providers, VMMC clients and Peers were receptive to integrating SRH services, particularly HIV prevention services such as CHCT and PrEP, into male dominated VMMC services. Adequate community sensitization is required when introducing other SRH services into VMMC services.

## Introduction

HIV/AIDS remains a major public health problem in sub-Saharan countries, such as Malawi, mainly affecting people in the reproductive age group of 15 to 49 years. Heterosexual intercourse remains the epidemic’s driving force of transmission, with 67% of infections occurring among steady relationships [1–4]. Prevention remains critical among the sexually active population. To intensify response to the HIV epidemic, Malawi expanded the “test and treat” approach by adopting universal antiretroviral therapy (ART) treatment for all HIV-infected persons in 2016–2017. In its 2015–2020 National Strategic Plan (NSP), Malawi set it’s WHO 90-90-90 targets for HIV treatment and prevention [5]. The overall goal was to achieve a 70% reduction of new HIV infections by 2020. The country aims to strengthen primary and secondary prevention efforts to realize the 90-90-90 targets, with much focus on maintaining HIV negative individual’s HIV free. Couples HIV Counselling and Testing (CHCT) has been a component of the country’s HIV prevention strategy [6]. However, the uptake of health care services as a couple has been low in part due to female-dominated health care settings. Efforts by women to bring in men for CHCT and other sexual and reproductive health (SRH) services have proved difficult despite evidence that CHCT is better than individual testing in preventing new HIV infections [6].

We explored views and opinions on service integration of CHCT and other SRH services in male-dominated Voluntary Medical Male Circumcision (VMMC) services in Lilongwe- Malawi. VMMC services have become an important tool for bringing men to HIV Counselling and Testing Services (HCTS) in Malawi. However, lack of female partner involvement in VMMC prevents women from utilizing this health care opportunity for CHCT.

In this analysis, we assess the views and opinions on integrating CHTC, PrEP and other SRH services into VMMC services and how best the services can be offered within the VMMC services. The analysis also includes reasons for and against the integration of services.

## Methods

### Study overview and setting

This formative study used qualitative methods to source views and opinions of integrating CHCT Services and other SRH services into VMMC services. The study was conducted in Lilongwe, Malawi between June, and August 2018. VMMC providers, peers and men receiving VMMC services participated in individual in-depth interviews to share their opinions on integrating CHCT and other SRH services into male dominated VMMC service environment.

At the VMMC clinic, men seeking VMMC are offered HIV counselling and testing and screened for STIs based on signs and symptoms and provided with syndromic management if symptoms are reported. If such treatment is provided, female partners (contacts) are offered STI treatment per standard of care. Partners to STI treated participants were free to receive treatment at VMMC clinic or any clinic of their choice.

No active couple mobilization strategies were in place to promote uptake of couple services at VMMC Clinic. No Pre-exposure prophylaxis (PrEP) was offered, and no female focused SRH services, such as cervical cancer screening and family planning services, were available at the VMMC clinic. Table 1 below outlines the status of services at the VMMC clinic at the time of the study.

**Table 1:** Overview of the services offered at Bwaila VMMC clinic.

Services available	Individual	Couples
HIV counselling and testing	X	X
STI Screening	X	NA
Syndromic STI treatment	X	X
Pre-exposure prophylaxis	NA	
Family Planning	NA	
Cervical cancer screening	NA	

X = Available Services NA = Service not available

### Study population

The study recruited 20 VMMC service providers (S1 File), 20 peers who motivate others to come for VMMC or guide clients flow within the clinic (S2 File), and 20 VMMC clients (S3 File). VMMC Clinic staff were briefed about the study before commencement.

Eligibility criteria for participants included being male or female, aged ≥18 years old, from within Lilongwe, able to provide consent, and willing to participate in-depth interviews (IDIs). The participants were recruited from the VMMC clinic for convenience. Study debriefing sessions were conducted with VMMC service providers. Group and one on one sensitization talks were conducted within VMMC clinic to raise awareness about the study among potential participants. Recruitment occurred through self-referral after clinic sensitization and through peer or a friend’s referral.

### Data collection

IDIs were conducted among VMMC providers (including nurses and clinicians), VMMC clients and peers. Well-trained interviewers, with skills in qualitative data collection, fluent in both the local language- (Chichewa) and English conducted the in-depth interviews in a private clinic setting. The study used interview guides developed by investigators to guide the interview (S4 File). The interview guides included predetermined themes (Table 2). Basic demographic characteristics, including age, marital status, and education were collected and documented separately. The interviews were digitally recorded to ensure all data is captured. The areas of formative investigation during data collection using in-depth interviews are summarized in Table 2 below.

**Table 2:** Summary of topic areas for all study participants.

Theme	Subtheme
Acceptability of offering couples HIV Counselling and testing services at circumcision clinics	Why it might be acceptable
	Why it might not be acceptable
Acceptability of other sexual and reproductive health services at circumcision clinics	Which services would or would not be acceptable?
	Why or why not would they be acceptable
How to implement couples HIV counselling and testing services and other sexual and reproductive health services at circumcision clinics	Where in the clinic should they be offered? When during the circumcision visits should they be offered
Barriers and facilitators to SRH service integration into VMMC service	How to overcome potential barriers

### Data analysis

All IDIs were transcribed and translated from Chichewa into English. The analysis process consisted of reading for content, identifying key themes and sub-themes and interpretation. The data were uploaded into NVivo 12 software for content analysis. Relevant codes from guides, transcripts and notes were developed and final codebook was created. All transcripts were coded by two independent researchers. Comparative technique and reconciliation were applied for final coding. Inter-rater reliability was measured with a pooled Cohen’s kappa.

Interpretation of IDIs focused on the acceptability of services by clients and providers, potential barriers, and facilitators of service uptake, as well as how and when the services should be offered.

## Ethical considerations

### Approval requirements

The Malawi National Health Sciences Research Committee (NHSRC) and the UNC Institutional Review Board in Chapel Hill approved the study prior to the conduct of any research activity. All research and implementation staff were trained in human subject protection and good clinical practices prior to the start of data collection. All participants provided written informed consent prior to participation in the research.

## Results

The study enrolled sixty participants. Participant characteristics are described in Table 3 below.

**Table 3:** Baseline characteristics of in-depth Interview participants.

Interviewed Participants (N = 60)	VMMC clients (n = 20)		Peers/clinic aides (n = 20)		Service Providers (n = 20)	
	N	%	N	%	N	%
**Age**						
18–24 years	10	50%	11	55%	0	0%
25–35 years	9	45%	5	25%	9	45%
36 years and over	1	5%	4	20%	11	55%
**Marital Status**						
Single	15	75%	12	60%	1	5%
Married	5	25%	8	40%	19	95%
**Education**						
Attended Primary School only	4	20%	3	15%	0	%
Attended Secondary School	11	55%	14	70%	2	10%
Attended College	5	25%	3	15%	18	90%

### Views and opinions on integration

#### Couples HIV counselling and testing services

HIV testing and counselling are components of VMMC services. Despite the availability of CHCT services at the VMMC clinic, the HIV testing focused on VMMC clients only. When asked about integration of CHCT Services into VMMC services, all participants expressed interest towards the integration. Some participants said that the integration would promote partner involvement in health care services, specifically VMMC, which is currently considered men’s affair. One VMMC provider said;

“Integration *is very good because when you do VMMC*, *people just think is the clinic for men*, *but when you do everything (integrated services) people will be coming to get everything*, *whether family planning*, *ART*, *testing*, *STIs*. *Like here*, *when someone is HIV positive*, *he is referred to (Name of Hospital)*. *Of course*, *it’s near*, *but when somebody comes*, *he has to get everything and not be referred outside*, *so the integration is important*.”

***42 years old*, *married*, *VMMC service provider***

Furthermore, participants explained that the integration of CHCT Services would enhance couples’ understanding of prevention messages and eliminate communication errors among couples as expressed below.

“*It is good*, *it is a good idea because if you integrate(couple) HIV testing and voluntary male medical circumcision aaahhh there is…*. *it’s more like a saying which is already there for circumcision*, *which everybody talks about that when you are circumcised*, *you are protected 60% from contracting HIV so some misquote that*. *So*, *involving both partners will make them understand that the 40% chances are still there for them to contract the virus*, *so condom use should not be ignored*. *So*, *I think the integration of these services will be a good idea*.”

***23 years old*, *unmarried*, *VMMC client***

#### Family planning services

All service providers and the majority of VMMC clients and Peers expressed interest in family planning service integration. Most participants said that integration of family planning into VMMC services is an opportunity to educate young men and increase knowledge and positive attitudes towards family planning services.

“*For (us) men*, *we do not know (about family planning)*, *because when they say family planning*, *people think that men do not need family planning and women also do not*, *especially men*. *Men*, *we are the ones who are far behind than women… yea*: *…… It is one way which can help us*, *we as youth… yea*.”

***36 years old*, *unmarried*, *peer***

Some participants viewed family planning integration as a good way of minimizing client referrals between departments because they are inconvenient. One ***51 years old*, *married*, *VMMC service provider*** explained; “*It is important because as I said*, *let people come and get whatever the service they want because the moment you refer them*, *they don’t go to the services anymore because when they come*, *they want that service at that time*, *so it’s important*.

Minimizing movements of clients between departments would increase service uptake and prevent loss of clients.

### Cervical cancer screening services

When asked about cervical cancer screening integration, participants expressed interest in integrating cervical cancer screening into VMMC services. Most participants accepted the integration because of the belief that VMMC protects women from cervical cancer *as this 18-year-old unmarried peer explained*; “*I feel like it’s very important because when a man wants to get VMMC it’s because he wants to prevent his partner from cervical cancer*. *So VMMC on its own does not prevent cervical cancer if it (cancer) was already there*, *it’s only from the time the man gets VMMC forward*. *So*, *if the woman gets screened before the man gets VMMC it can help even if she is found out to have the cancer because she can be put on treatment*. *If she doesn’t have it*, *then VMMC will be able to prevent it*. *This is better than a man just doing VMMC without knowing whether the partner already has it or not*. *There have been several cases where women have cervical cancer and yet their partners did VMMC*. *So*, *I feel this is very important*.

Integrating Cancer screening into VMMC will help couples know if the woman has cancer or not and put on treatment when necessary to save lives.

Furthermore, once services were integrated, women would get more involved in VMMC and will become knowledgeable about circumcision. This participant explains:

“*That will be a very good thing to do as one of the advantages for circumcision*, *helps women to prevent getting cervical cancer*. *So*, *if it can be integrated in this project*, *it can be wonderful*. *And more women can surely understand the circumcision of their husbands*, *so that they can be encouraging men to go for circumcision*. *So that can be perfect …*.”

***32 years old*, *unmarried*, *VMMC Service provider***

### Pre-exposure prophylaxis

Prior to discussions, participants were asked if they had ever heard of PrEP All participants had no prior knowledge of PrEP The interviewer explained what PrEP is to participants as follows:

“*PrEP is anti-HIV medicine that keeps HIV-negative people from being infected*. *There is a single pill that is taken once daily*, *and if you take it regularly*, *it is highly effective at preventing people from being infected*”.

The interviewer ensured that participants demonstrated an understanding of PrEP before continuing with the interview regarding the potential use of PrEP One VMMC client defined PrEP as follows, and felt that integrating it with VMMC services would be appropriate:

“*It’s because PrEP is anti-HIV drugs*. *When we come here for VMMC*, *all our thoughts are on HIV prevention because they say the risk for HIV infection is lower for those that get VMMC*. *So*, *the bottom line in all this is HIV prevention*. *If VMMC is for HIV prevention and PrEP is also for the same cause*, *then combining this two will be for the better good*”.

***38 years old*, *unmarried*, *VMMC client***

When asked their opinion on integrating PrEP into VMMC services, most VMMC clients felt that PrEP was another good way of protecting themselves from contracting HIV. Some clients emphasized the benefits of PrEP, especially for discordant couples to prevent infecting each other with HIV

“*It’s a good thing because*, *when you come to the hospital with your wife and one of you is positive*, *the doctors or nurses should say that the drug is available that can be used to prevent cross infection*, *you can be having intercourse even having kids without any problem*”.

***37 years old*, *unmarried*, *VMMC client***

Some VMMC providers discussed PrEP integration in the context of potential decreased condom use after VMMC, noting that clients feel they are protected from HIV after undergoing VMMC. In such cases, accessing PrEP within the VMMC services would further protect the circumcised men from HIV infection.

“*My opinion is that this pill will help because when people have done circumcision*, *they may no longer be using condoms*, *so I think this integration should happen in the clinics to protect such people from contracting the virus*”.

***32 years old*, *unmarried*, *VMMC service provider***

### Sexually transmitted infections

Although STI screening and management services were available, there was no formal STI clinic as a service to the public but as an integral process of assessing eligibility of VMMC Clients to undergo circumcision. Participants felt that integrating STI services into VMMC services to be a much-needed service to the community at large, more specifically when conducting VMMC outreach clinics in geographically disadvantaged populations in need of STI services.

“*I think that’s a welcome idea because some people live very far away from hospitals*, *yet VMMC has outreach clinics in the communities*. *So*, *if they integrate VMMC with STI management then it can (reach out and) reduce the number of STI infections (in communities)*, *and people will take care of themselves because they will know at an early stage and get treatment for the STIs*”.

***24 years old*, *unmarried*, *peer***

Introducing new services into VMMC services has the potential to change how clients perceive the services. To mitigate potential negative perceptions and encourage the uptake of integrated CHCT and other SRH services within VMMC services, some participants recommended for community sensitization to raise awareness amongst couples and public. *For the integration of couple HIV testing*, *this one participant said the following*:

“*I think if we can do more sensitization campaigns*, *this will help so that we can impart knowledge to both parties so that they can know the benefits of integrating the (CHTC) service*”.

**36 *years old*, *married*, *Service provider***

Furthermore, one participant explained the importance of sensitization in helping people make informed decisions to access integrated services at VMMC clinics.

“*…*.. *It’s like I said that if the people are sensitized on the integration of VMMC and cervical cancer and family planning then when going to the VMMC clinic they will have an open mind knowing that when they come to the VMMC clinic they will get messages about family planning and cervical cancer*. *………So*, *they will prepare their minds and know what happens here at the clinic unlike someone from the village who doesn’t know anything and when he comes here*, *he will just hear* “*After this we want to screen you for cancer or something*” *so it will come to them as something they were not expecting*”.

***21 years old*, *unmarried*, *VMMC client***

### Proposed ways of offering services

Integrating services require stationing services at strategic points for easy access and quality service provision. Participants suggested various ways on how services could be offered within the VMMC services to meet their satisfaction with the integration. The majority suggested the services be within the VMMC clinic but in separate rooms particularly for services such as family planning, cervical cancer screening and PrEP For example, this participant explained on how family planning can be offered in VMMC Clinic:

“*If the room is outside the clinic*, *then it will be a special family planning clinic which can make people to feel shy to access such services since they are on the open*. *So*, *making the room to be inside will help people to go for family planning without shame because others won’t know that they have gotten such services*”

***20 years old*, *unmarried*, *VMMC client***

A few participants suggested the services be incorporated within the specific points within the clinic flow. This participant explained:

“*So I feel that it would be better that once people come they should be told about family planning or maybe they can just combine everything starting from what is involved for one to be circumcised*, *STI screening…or they can either be offering the service at the beginning and at the HCT (HIV counselling and Testing) room because some people don’t feel comfortable being told issues like these in a group while others are comfortable like that*, *it just depends on the approach they use*. *………… So right at the beginning and at the HCT room can help people to feel free*”.

***19 years old*, *unmarried*, *VMMC client***

Finally, the majority of the participants felt that sensitization of individuals and communities is required when planning for successful and smooth implementation of the integration.

### Reasons for disinterest and concerns about service integration

Even though most participants accepted the idea of integrating the services, some did not agree to it. They presented various reasons against integration. Some participants were concerned with gender mixing. One 19-year-old unmarried participants said; “*Mmm*, *I feel like sometimes it’s hard for men and women to feel free with each other*………..: *So as it is at the VMMC clinic*, *I think it’s easier for men to come and talk about issues concerning VMMC*, *especially for those that went through it already to encourage others*, *because sometimes it’s easy to understand someone that went through an experience more than a doctor*………*So I feel that it is better for the VMMC clinic to be the way it is so that men can freely talk about issues*. *Sometimes we speak so loudly that it helps someone who is not there speaking with us*, *but if we combine then we cannot speak as loud as we do because there will be women around*. *That’s why I said earlier on that any type of screening should be done at the beginning so that the partners can be done*, *go home and leave men to have a chance to talk and encourage each other*”. This suggest that men feel comfortable and open to each other in their own space rather than mixing with women.

Other participants were concerned about sustainability of the services. This peer was specifically concerned with PrEP sustainability and he said;

**“***I think that cannot be a good idea as I earlier on said*, *that the VMMC Clinic is sponsored by NGOs*, *and these are not stable people*. *They can be here today*, *and tomorrow you find that they are gone*. *So*, *if at first*, *you received the medicines (PrEP) from these VMMC people (VMMC clinic)*, *and once they are gone*, *you will not complete the dose and that will be a challenge to you*. *I prefer that these medicines should be found at a stable place where anyone who would like to use them should have that direct access to get them*.”

***31 years old Married peer***

Furthermore, concerns arose about lack of provider capacity to provide various services, lack of adequate space to accommodate people during services, and long waiting times that may have a negative impact on uptake of VMMC and its allied services if too many services were integrated. Some participants were concerned that men would shun VMMC services due to compromised privacy if female SRH services were integrated into VMMC. Training of staff, creating space and public awareness on service integration were suggested as ways to address the concerns.

## Discussion

In this study, VMMC Participants, peers, as well as providers showed interest towards integration of CHCT Services, PrEP and other SRH services into VMMC services. Much of their acceptability was grounded in the fact that couples will be able to know their health status, prevent STIs, receive more health education and prevent loss of clients through referrals to other departments for services. While VMMC services are male-dominated services in Malawi, the service should potentially be viewed as a unique opportunity to address other health needs for men and their partners in addition to HIV prevention.

Integration of services has been a focus for over 20 years [7–9]. HIV and SRH integration has developed the most prominence since the discovery of HIV in the 1980s [8].

Participants found the integration to be an interesting way of promoting couple services and to have men bring in their spouses for services. Early diagnosis and treatment of HIV, cervical cancer and STI screening and management were perceived as important SRH services for service integration. It is therefore suggestive that the integration would provide a unique couples services environment and support the 90: 90: 90 strategy by increasing CHCT Services within the VMMC clinic.

Integration of services will give an opportunity to men to be educated and get involved in services, which previously have been considered as female dominated. Studies have extensively documented the positive impact of integrated services on service uptake and male involvement [10]. In addition, female involvement in VMMC services has been found to increase VMMC service uptake [11–14] Participants suggested to implement individual and community sensitization to raise awareness on services offered at VMMC, encourage men to bring along their partners. The VMMC clinic need to create space to accommodate couples and feminine sexual reproductive health service. Women should be encouraged to get involved in VMMC services to promote support for their spouse. A recent demand creation intervention in Uganda and Kenya [15]. by Semeere et al. (2016) highlighted the feasibility of pregnant women engaging their partners regarding VMMC. Another study in Zimbabwe showed that integration of VMMC with other services is feasible and has a similar impact as vertical delivery, with potential benefits of capacity building, sustainability and health system strengthening [16]. This suggests that integration of other services into VMMC services can be feasible and should equally be viewed as an entry point to SRH services and other health issues and education.

Men are considered decision makers in most families, including on health matters [17, 18]. Making services accessible to the deciders will provide the opportunity to promote responsible, educated men, including young men, as early as possible on the critical role they can play in ensuring the health of their families and communities. The introduction of women’s health services such as family planning and cervical cancer screening would contradict the belief that VMMC clinic is only for men. These beliefs lead to missed opportunities for partners testing and service engagement at VMMC clinic. The integration of SRH services into VMMC services would encourage men to take greater responsibility for preventing HIV transmission, including promoting positive gender norms and practices.

There were a few limitations for this study. Women were not involved in the IDIs, despite that the services being studied in this research have a direct impact on female clients. Women’s views on this integration could have provided important data on how women view the integration of services into VMMC service and how it may affect them. In addition, because participants were already receiving some of the services, such as STI management and HIV testing, as part of the VMMC procedure, their positive impressions of the VMMC services context may reflect on their desirability for the integration. In addition, most participants were younger men and unmarried with no knowledge of couple dynamics with regards to receiving health care as a couple. Their acceptability may change with changes in their marital status and increased l understanding of what it would be like to receive services as couples.

Nevertheless, our findings in this study suggest that integration of CHCT Services and other SRH services may be acceptable and act as an entry point to HIV prevention and treatment services to couples in Malawi. As the services are being integrated into VMMC services in Malawi, it is important to ensure adequate sensitization for both men and their partners and their communities. Furthermore, the successful integration may hinge on delivery of the services. The services should be in an environment that is sensitive to men’s concerns. These findings can inform the implementation of the VMMC integration program for VMMC clients and their partners.

## Supporting information

S1 File Providers transcripts. (DOCX)

S2 File Peers or clinic aides transcripts. (DOCX)

S3 File Male index clients transcripts. (DOCX)

S4 File English and Chichewa interview guides. (DOCX)
