# Supplementary material for: Opinions on integrating couple counselling and female sexual reproductive health services into Voluntary Medical Male Circumcision services in Lilongwe, Malawi
Source: PLoS One. 2022 Sep 9;17(9):e0273627. doi: 10.1371/journal.pone.0273627 (PMC9462804; doi:10.1371/journal.pone.0273627)
Supplement: S1 File — (DOCX) [file pone.0273627.s001.docx]

**D 43 STUDY**

**Date of Interview: 26 June 2018**

**Type of Participant: Provider**

**Interview Number: D-43-0001**

**Interviewer: C. L.**

**Total Interview Time: 31 minutes 27 seconds**

**Interview Summary:** **(from summary sheet)**

| **SERVICE TO BE INTERGRATED** | **THOUGHTS ON INTERGRATION** |
| --- | --- |
| Couple HIV Testing and Counseling | Information will get to the clients as couples and it will help because clients they will not need to go to other clinics for the service. |
| STI Services | Thinks it will help to reduce short-cuts that happen during the VMMC process when there are too many clients at the clinic. |
| Family Planning | The integration would help the clients to access the service at the clinic since some fail to go to other clinics when they are told that VMMC clinic does not offer family planning. |
| Cervical Cancer Screening | Clients are told that VMMC reduces the risk of cervical cancer so this integration will help the providers not just to say this but also to provide another way of further reducing that risk for their partners. |
| PrEP | Not comfortable with PrEP because it can make people to be reckless with their lives |
| Other Services | Thinks cervical cancer screening and family planning are the best to be integrated into VMMC |

**Remarks:**

**Participant was relaxed and had no trouble understanding the questions. He seemed reserved when the issue of PrEP came up and said he wouldn’t encourage people to be taking it.**

**Interview Text:**

1. I: Thank you for taking the time to talk with me today.
2. *R: Thank you.*
3. I: I would like to ask you some questions today about the way you feel and what you think about some issues related to the service you provide and how we can include other services in Voluntary Medical Male Circumcision clinics.
4. *R: Alright.*
5. I: There is no right or wrong answer to these questions. We would like to hear your opinion and your experiences in your own words. Do you have any questions before we begin?
6. *R: No, I don’t have any questions.*
7. I: There is no question?
8. *R: No.*
9. I: Okay. How are you involved in client care at this clinic?
10. *R: I am a hygienist, I clean the whole clinic starting with the reception so that when our clients come they find a clean environment.*
11. I: Do your client talk to you about the services that you provide here?
12. *R: Yes they do talk to us.*
13. I: Okay, can you give an example of a time when they talked to you?
14. *R: For example when some clients come they don’t know where to start or what is involved for one to be circumcised so we show them where to sit and if the receptionists are available we refer them to the reception. Then they get registered then they go for HTS (HIV Testing Service).*
15. I: Okay. So now I want us to talk about Couple HIV Testing and Counseling. What happens when a client brings a partner to the Medical Male Circumcision clinic?
16. *R: What happens is that when a couple comes we ask them “How can we help you?” So they say “We want to access male medical circumcision service” and we ask “Have you come with a partner?” And they say “Yes” Then we tell them “You have done well, there is nothing wrong with bringing your partner here”. So the man gets registered and they both go to HTC (HIV Testing and Counseling). So information about circumcision process, wound care, preventing infections- since circumcision only reduces the risk of infections by 60 percent and that means the remaining 40 percent is left for them to either be faithful to one partner or else to use condoms. All this information is given to them as a couple together so that when they go home they will be able to avoid other problems because they were together when the information was being given.*
17. I: Mmm, so the woman is there when you give the man the information?
18. *R: Yes they are together.*
19. I: Okay. Alright.
20. *R: Yea.*
21. I: What do you think are the motivators for men to bring their spouses for HIT testing?
22. *R: There maybe a number of reasons one of which maybe a lack of trust between the couple thinking that the spouse is promiscuous and they may think “ Maybe if we go together for testing then things can be better”. Another reason is that mostly we men find it difficult to make a decision especially when it comes to medical issues. SO if maybe the woman heard from her friends about the benefits of medical male circumcision and she tell s the husband, he may be reluctant to go for circumcision but if the wife tells him “let me escort you there” he is motivated to go.*
23. I: So it’s like the woman motivates the man to go…?
24. *R: Yes because on his own the man can just be saying “I will go later “but the woman put an amount of pressure on him so that he goes.*
25. I: Okay. How about those that don’t bring their partners for testing, what do you think makes them not to bring their partners?
26. *R: I think there might be two or three reasons. One reason maybe that the person is not yet married, or has not yet reached the age of finding a partner.*
27. I: Mmm.
28. *R: … for other people it might just be a decision that they make to say “I cannot bring my wife or girlfriend to such a place”. So since we are born differently it’s just how some people are.*
29. I: Mmm. So what makes them make that decision to not take their wives or girlfriends along? We want to understand the reason why they make such a decision.
30. *R: For some people it’s because they are shy. They feel that maybe they have not yet made the decision to marry the current partner so they feel shy to reveal such information to a girlfriend. They feel like “She will know my HIV status and if this thing ends badly in the future she may end up telling other people about my status [laughing].*
31. I: Sure. How about for those that are married? What makes them to make the decision of not bringing their partners?
32. *R: Most men consider women as not the first people to be considered when making a decision. They say “I can do this on my own, what can a woman tell me?”*
33. I: Mmm
34. *R: Men have such an attitude most of the times.*
35. I: Okay. So what do you think can be done so that men can be bringing their partners for couple testing at this clinic?
36. *R: Mmm, firstly we need to emphasize on sensitizations so that the people have enough information. Secondly there needs to be privacy because to reveal a person’s status is not easy. So when the couple comes, we need to have a private place where we can sit with them and talk to them in a way that no one can be able to hear us. Because it may happen that you are talking about a certain issue and some people are passing by and get to hear what you are talking about so that becomes a problem.*
37. I: Mmm okay. You as a health care provider, what is your opinion on integrating couple counseling with Voluntary Medical Male circumcision services?
38. *R: Yea.*
39. I: What is your opinion about integrating these services?
40. *R: I feel that it would be good for us if these services are integrated because if the information gets to the people as couples, they would be encouraging each other. As I said earlier that we men have a problem with going to the hospital. If you have a headache and you come to the hospital they tell you to stand in a queue with women, so for you to stand in a queue like that with other people’s wives, you feel like “Mmm, why don’t I just go home and look for a pain killer then come back later to the hospital?”. But as it is here, if there were some separate rooms for consultation for men and women, that would be great.*
41. I: Okay, so what do you think would be the barriers or concerns for this integration?
42. *R: Of course there might be some concerns because some people may not be really happy about it but we can start and as it goes, people can get used to it because people may have a negative attitude whenever something has just been established but as time goes by they get used to it after some time.*
43. I: Alright, fine.
44. *R: Mmm.*
45. I: So what can be done to overcome these concerns?
46. *R: We need to civic educate the people so that they understand how this will work out in more details because sometimes we just rush to tell the people without giving them the details. If we just say “we are doing circumcision” people will wonder “What is circumcision? I have never heard about that” That is because we are of different cultures, there are some people who get circumcised at a young age because of their culture while others do not get circumcised because it’s not in their culture. So you need to clearly explain to the person about it because others think that “I have been circumcised and I am 100 percent protected so I will just be having unprotected sex anyhow”. You find that two to three days the person has contracted infections and will be blaming you to say “You lied to me! You said I would not get infected because I am circumcised”. When I actual sense the person is only be safe by 60 percent.*
47. I: Okay.
48. *R: Yea.*
49. I: Now I would like to discuss with you about sexual reproductive health services and Pills for HIV prevention: called pre-exposure prophylaxis. (PrEP)
50. *R: Yea*
51. I: Sexual reproductive health includes services that promote good sexual health and reproduction.
52. *R: Yea*
53. I: They include but not limited to family planning, cervical cancer screening sexual transmitted infection (STI) management, and many more.
54. *R:Yea*
55. I: Today we will only talk about family planning, diagnosis and management of STIs, Cervical cancer screening, and PrEP... We will look at each of these one by one.
56. *R: Okay.*
57. I: Let us start with diagnosis and management of STIs. What happens when you suspect or diagnose a client with STIs?
58. *R: Mmm, when a client comes, before he goes for procedure, and after he comes out of the HTS room and he has gotten his HIV test results, he goes for STI screening. He is checked if his penis has any infections because it may happen that the client two days before and he has contracted some STIs. So such a person is put on hold because we don’t want to create a wound where there is already an infection that means the wound will not heal easily. So we treat the infection first and he is given some medication and an appointment date when he can come back. When he comes back we check the infection if there is any improvement or we should continue with the treatment.*
59. I: Mmm
60. *R:Yea*
61. I: Okay. So as a health care provider what is your opinion on integrating STI services with Voluntary Medical Male circumcision services.
62. *R: I feel that this will benefit us a lot.*
63. I: Why do you say so? What connection is there between STI services and Voluntary Medical Male circumcision services?
64. *R: What happens is that before we circumcise anyone we need to screen him for STIs, so I feel that this integration will help to eradicate some of the short-cuts that happen during the process some of the times because it happens sometimes that you have too many clients and a few providers. So you just take the client from the HTC room straight to the theatre room to operate on him, jumping the screening process. This happens sometimes. So if the integration is done, it can act as a reminder to each and every provider doing the circumcision to not jump any of the steps.*
65. I: Mmm
66. *R: Sure.*
67. I: Okay. So what do you *do not like the integration of STI with* Voluntary Medical Male *Circumcision services?*
68. *R: [No response]*
69. I: *What could be a challenge with this integration*
70. *R: The challenge could be that…its men that come to the VMMC clinic for circumcision so sometimes the man can be diagnosed with STIs but his partner is not at the clinic, maybe she is home or he just had sex with a woman who is not his wife. So for the partner to be treated it becomes difficult.*
71. I: Okay. You know the clinic flow here at the clinic right?
72. *R: Yes.*
73. I: So how do you think STI services should be offered at the Voluntary Medical Male circumcision clinic? When and where should they be offered?
74. *R: Screening for STIs?*
75. I:Yes
76. *R: Aah, after…I think the clinic flow should be left as it is only that there should be more of a reminder so that these short-cuts can be avoided.*
77. I: Alright, what can be included apart from the reminder so that we eradicate the short-cuts?
78. *R: Mmm, what we can do…*
79. I: Mmm
80. *R: Let’s say the client has come out of the counseling room, there needs to be another room for screening because when a man comes for circumcision, he needs to go through the screening procedure first.*
81. I: Mmm
82. *R: So we need to emphasize much to the one who is doing the screening so that he stresses much on the screening.*
83. I: Okay, do you think there may be any concerns to this work of emphasizing or stressing the need for screening for STIs, reminders or the general integration?
84. *R: Aah no there cannot be any concerns.*
85. I: How about barriers? You said earlier on that sometimes you get overwhelmed with work…
86. *R: Yes the barriers can be there because we can have issues with attitude, human resource when we are under-staffed and that makes health care providers to involve short-cuts. Say for example a client has been registered at the reception and you take him to the HTC room and you do the testing, after that you need to do STI screening then maybe there are other clients who have come for a review, or the same provider needs to go to the theatre room for the procedures and after the procedures he needs to go into Post-op room.*
87. I: Mmm
88. *R: You see?*
89. I: Mmm, so that means the provider has too much work?
90. *R: Yes it means there is too much work and less human resource.*
91. I: Mmm.
92. *R:Yea*
93. I: So how can we deal with such a concern?
94. *R: That means we have to recruit more health care workers.*
95. I: Mmm, okay. Now let us talk about family planning.
96. *R: Mmm*
97. I: What happens when a client needs family planning methods. Say a client comes for circumcision and he comes with his partner who needs family planning or as a couple they want family planning.
98. *R: If that happens…at this clinic the only family planning methods that we offer are condoms.*
99. I: Mmm
100. *R: But on top of that we tell them as a couple that there are other methods like Loop, injectable, and where they can get such services like at (Name of clinic).*
101. I: Okay. So a health care provider, what is your opinion on integrating Family planning in circumcision services? SO that you don’t refer the couples to other clinics but you offer the services here at VMMC clinic?
102. *R: For that we were thinking that we could link up with other clinics so that when we find a client…because our policy is that we cannot offer other services apart from VMMC and condoms.*
103. I: Okay. What if the policy changes to say you can be able to provide these other services?
104. *R: That could be good.*
105. I: Okay.
106. *R: Yea.*
107. I: Why do you say so?
108. *R: It’s because some clients go home to other clinics just because we do not provide these other services, which makes others to not even go to the other clinics because they have to ask for a map to go there. They may ask “Where do you say the clinic is “and you can give the map which he doesn’t understand and that makes him not to go and look for the place. So it’s like the client came to the clinic but you were not able to provide the needed services so they get disappointed.*
109. I: Mmm, okay.
110. *R: Sure.*
111. I: What *is it that you would not like the integration of family planning in* Voluntary Medical Male *Circumcision Clinic?*
112. *R: What can bring a concern is that most of the times it’s women who get involved in family planning issues. So with the circumcision service that we provide here, it will be like more women will be coming instead of the men that we target [laughing].*
113. I: [Laughing]
114. *R: I hope you understand what I am trying to say?*
115. I: Yes I understand…
116. *R: It’s because normally when there is something happening its women who get carried away with it. As for us men we like procrastinating. Even if you can start an intervention right now, it will be mostly women who will come. For men it’s a real challenge. Even the circumcision service that we are doing, you can do lots of sensitizations but men just say “Aah, I will go later”. You can have a one week out-reach clinic but from Monday to Thursday you register low client turn up because some men have fears about whether they will come out alive from the theatre room. They wait to see the first man who gets circumcised on Monday to come for a review to have confidence that they will be alright after the procedure. So you find that from Monday to Thursday you have low client turn up but on Friday and Saturday you have a lot of clients because some of them were failing to come earlier due to fear [laughs].*
117. I: Okay so how do you think family planning services can be offered within Voluntary Medical Male circumcision clinics?
118. *R: I think we can… we can be talking to the men that come for VMMC about the benefits of family planning so they can talk to their loved ones when they go home because some people lack information about family planning or where they can access family planning services. There are other people even here in Malawi, in typical villages who have never heard about family planning. When you talk to them about family planning they are like “What is family planning? I have never heard about that!” So if you give such people enough information they are able to make good decisions.*
119. I: Okay. So apart from the concern which you mentioned that more women would be coming to the VMMC clinic is there any other concern or barrier concerning this integration on your part as a provider?
120. *R: No there is none.*
121. I: Alright. So on the concern that there would be more women coming to the VMMC, what do you think can be done about it?
122. *R: I think on that we can have a restriction on the age group that comes so that we can put a control. We can say “women from this age range can come” so that those that have not reached that age do not come and those that have exceeded that age cannot come either.[laughing]*
123. I: [laughing]. Alright so let’s now talk about cervical cancer screening.
124. *R: Alright.*
125. I: What happens when a woman needs cervical cancer screening here at VMMC clinic?
126. *R: We don’t provide that service here but if a woman needs it then we just refer them up there.*
127. I: Has it ever happened that a woman came here looking for such a service?
128. *R: Yes it happens very often.*
129. I: Okay.
130. *R: It’s also because our clinic is close to the road so when people want to ask for information they find us first.*
131. I: Mmm
132. *R: If you can see this building was a maternity ward so some people still have that in mind and they think that the building is still being used as it was used in the past so we just refer them to the clinic up there.*
133. I: Okay. So as a health care provider, what is your opinion on integrating cervical cancer screening in Voluntary Medical Male Circumcision services?
134. *R: I feel that such integration would do us good because we tell men that VMMC protects their partners against cervical cancer.*
135. I: Mmm
136. *R:So if this integration is there then we can be telling them “from this room you need to go to that room to hear more about cervical cancer screening” and if the man has come with his partner then they can go together and she can get screened right there and then. In that way both of them can get the services they need when they go home.*
137. I: Okay, alright. Can there be any concern or barrier on this integration?
138. *R: No.*
139. I: There is none?
140. *R: No.*
141. I: How do you think is the best way to offer cancer screening within Voluntary Medical Male circumcision clinics?
142. *R: I think if there can be the screening machine and a health care provider who can be able to operate the machine and maybe a separate room where anyone who comes with a partner can get screened.*
143. I: Alright. Is it that this service is not available here because there is no machine or room which they can be doing the screening?
144. *R: The room can be provided as long as the machine and the one who will be operating it are available. But as for the time being the resources are not available.*
145. I: Okay, so where in the clinic flow can this screening for cervical cancer be included?
146. *R: I can just suggest that maybe when they come out of the HTC room. The men can be going for screening for male circumcision and if they came together with their partners then after HTC the partners can be going for screening for cervical cancer screening.*
147. I: Okay, so do you think are the barriers and concerns on this integration?
148. *R: Aah, no.*
149. I: There are concerns?
150. *R: No there are no concerns.*
151. I: Alright. Now let us talk about PrEP. Have you ever heard about PrEP?
152. *R: Yes.*
153. I: What do you know about PrEP?
154. *R: It’s a drug that you can take if you have been accidentally pricked by a syringe especially for us health care workers. So you take it within 72 hours.*
155. I: Okay, so what you have defined is PEP but now we want to talk about PrEP.
156. *R: Oh?*
157. I: Yes. PrEP is like PEP but they are a bit different because PrEP is an anti-HIV drug that keeps HIV negative people negative. Say for example you have never had sexual intercourse or you have sex with people. You can be taking this pill once a day and if you do that regularly it will effectively you from being infected even if you are exposed to HIV. So it’s a drug that keeps you negative even when you have been exposed to HIV.
158. *R: Okay.*
159. I: So have you now understood what PrEP is?
160. *R: Yes I have.*
161. I: So if PrEP was made available to HIV- men and women. Do you think you could advise your HIV negative clients to accept to take PrEP?
162. *R: I would firstly consider if there are any side effects because before we implement things we need to look at all angles. You can tell the person about it and he start thinking “Now that I am taking PrEP then I am 100 percent protected from HIV. I can be having unprotected sex the way I want” which can lead to other negative things. We also need to look at the availability of the drugs and where we can get it if we are out of stock.*
163. I: Mmm, okay. So can we say you are indifferent?
164. *R: Well, for me I think it can be hard.*
165. I: You would not encourage someone to be taking the drugs?
166. *R: No.*
167. I: Okay. What would be your concerns with this integration?
168. *R: My concern would be that when you tell people that they can take PrEP then they would be reckless with their lives thinking that they have protection against HIV.*
169. I: Okay. So let’s say powers to choose and integrate services in Voluntary Medical Male Clinics, what are the services that you would think of Integrate?
170. *R: I would choose screening for cervical cancer and family planning for integration.*
171. I: Why have you chosen screening for cervical cancer?
172. *R: It’s because the VMMC service that we provide is related to the cervical cancer so I feel that if we can be providing these two services together then it can be good.*
173. I: Okay. How about the family planning service?
174. *R: I have chosen the family planning service because we protect people by 60 percent here but we don’t provide other family planning methods like Loop and the like. When a person comes for family planning we refer them to other clinics but we don’t know whether they really go to access such services or whether they have been giving the services. It’s hard to follow up on that.*
175. I: So how do you think the cervical cancer screening and family planning can be offered at the clinic? In what way, at what time points and things like that?
176. *R: Umm, I think if you can talk to the management they can allocate a room where the services can be offered. If anyone asks about such services, then they can be referred to that room.*
177. I: Mmm.
178. *R: Yea.*
179. I: I thank you so much for taking your time to talk to me today.
180. *R: Yea.*
181. I: Your answers will be very helpful in improving the health service delivery at Voluntary Medical Male circumcision clinics.
182. *R: Alright.*
183. I: Before we close do you have anything that you wish to add?
184. *R: No there is nothing.*
185. I: Alright. Thank you for your time.
186. *R: I also thank you for talking to me as well. You know when it’s something knew you get nervous and do not know whether you would be able to answer but here I have been able to respond to all your questions.*
187. I: Yes thank you.
188. *R: Thank you.*
189. END

**D 43 STUDY**

**Date of Interview: 02 July 2018**

**Type of Participant: Provider**

**Interview Number: D-43-0002**

**Interviewer: C.L.**

**Total Interview Time: 25 minutes 17 seconds**

**Interview Summary:** **(from summary sheet)**

| **SERVICE TO BE INTERGRATED** | **THOUGHTS ON INTERGRATION** |
| --- | --- |
| Couple HIV Testing and Counseling | A good initiative which can enhance bonds in relationships. |
| STI Services | A good initiative which will help both partners to be treated whenever one is found with an STI. |
| Family Planning | The integration can work but it will be too much work for the providers. Integration should be piloted first. |
| Cervical Cancer Screening | A very good initiative as cervical cancer is connected to VMMC. Integration would help men to be coming for VMMC since women can be encouraging their partners to be coming for VMMC. |
| PrEP | A welcome idea since VMMC protects at 60% so PrEP would help to reach 100%. |
| Other Services | Thinks cervical cancer screening, PrEP and couple testing and counseling should be integrated with VMMC |

**Remarks:**

**Participant was relaxed and had no trouble understanding the questions. He seemed reserved when the issue of family planning came up and said it would be too much work for the providers.**

**Interview Text:**

1. I: Alright, so … thank you so much for taking your time, so that we have a talk
2. *R: Alright*
3. I: So as I explained, I would like to ask you some questions, concerning your opinion on certain services which you provide at this circumcision clinic … and also in addition, we would like to hear your opinion on including some services besides that service you already provide here, concerning circumcision.
4. *R: Okay*
5. I: so, there is no right or wrong answer on the questions which I am going to ask you
6. *R: Alright*
7. I: So maybe you have a question that you might need to ask me before we start
8. *R: ah no, there is no question. We can start*
9. I: Alright… fine…. So can you tell me how you are involved in the client care at this clinic?
10. *R: ehm… we, at this clinic, we are involved in….to be specific cleaning… so we deal more with the issues of IP, Infection Prevention.*
11. I: Okay fine, so… do your clients talk to you about the services which they want to receive at this circumcision clinic?
12. *R: yes, sometimes they talk to us. Maybe they want to know more, we could explain to them…ehm*
13. I: Give me an example… for the day when a client finds you
14. *R: I remember a certain time, I came Saturday, he found me, and that time a person working on data was not yet in at the reception. So he found me, and I explained to him, how it works, from where it starts and where it ends. Sometimes, it happens that you are at the reception and someone is asking, we could explain it to the person, how it works so that he can make the decision to be circumcised.*
15. I: Okay, fine… so let’s now talk about couple HIV testing at this clinic. What really happens if a man brings a spouse here at the Voluntary Medical Male circumcision clinic?
16. *R: Hmmm… often they do not come together, but individually for circumcision as a male and his blood got tested for HIV. It has not yet started coming as a couple to test for HIV.*
17. I: What do you think demotivates men to bring their partners here for HIV counselling and testing?
18. *R: … Hmm… maybe because the circumcision is for men, the women they could be like, aren’t this for men? It does not concern me… and so the men come here alone for circumcision and so they get their blood tested alone.*
19. I: Has a scenario ever happen, that a men came here with his partner? Even one men?
20. *R: hmmm yes…. It really happen*
21. I: Okay
22. *R: Yes, but it’s rarely…. Some time back it used to happen, they could come as a couple especially if it is a woman who has influenced a man for circumcision, so they could come as a family but if they are not influenced, they could come alone.*
23. I: so, what do you think can be done to make men bring their partners here for couple testing and counselling?
24. *R: Hmmm… this is really needed ….and what is needed to be done is to explain to them, the good thing of involving women on circumcision… then they could be coming as couple for circumcision as well as HIV testing at the same time. And it is possible. Most men also think, that is only for them and that’s why they come alone and women do not mind too. Maybe telling them the good things that the women should be there, they might be coming as couple. What is needed is just to tell them, the good things about coming as a couple.*
25. I: Okay fine… so you as a health care provider, what is your opinion on integrating couple counseling with Voluntary Medical Male circumcision services?
26. *R: It is really a good thing for them to be tested as a couple because (hmmm)... it can enhance their relationship within their family as they can be coming together and get tested. And the men have been circumcised and he is supposed to be like this for such period of time. So they can be having a clear updated information all together.*
27. *I: ehm… so, what do you think are the barriers and concerns on this integration*
28. *R: Hmmm…. In terms of barriers, I cannot say right now… but I think, it cannot be many… people can accept it well*
29. I: Okay, so what if there are barriers, what do you think can be done to overcome these concerns and barriers to couple counseling in Voluntary Medical Male circumcision services clinic?
30. *R: Hmmm… if barriers can be found, what is needed to be done, is to find the reason for such barriers and then you can find solutions to address the identified reason. So like, if there is any barrier, it can be overcome after analysis the focal problem. After that, we can address this.*
31. I: Okay, fine. Now I would like to discuss with you about sexual reproductive health services and Pills for HIV prevention: called pre-exposure prophylaxis. (PrEP). So this Sexual reproductive health includes services that promote good sexual health and reproduction. They include but not limited to family planning, cervical cancer screening sexual transmitted infection (STI) management, and many more. So today we will only discuss about family planning, diagnosis and management of STIs, Cervical cancer screening, and PrEP... We will look at each of these one by one. Let us start with STIs services or in other word, they say… screening for STIs.
32. *R: Okay*
33. I: Explain to me what happens if a client is suspected or diagnosed with an STI here?
34. *R: What happens here is that… if a person has been screened and is found with STI, at first, he need to be treated for the STI found. He receives a treatment for that STI, before circumcision.*
35. I: Okay, so you as a health care provider what is your opinion on integrating STI services with Voluntary Medical Male circumcision services?
36. *R: Hmmm, it may be found that, maybe the service for STI, like the way we do or there is another way? If they screen for STI, treatment is there already. If he is helped with the problem of STI, he can come for circumcision. But he cannot be circumcised without being treated. After enough treatment, he can then come for circumcision, but he receives treatment for STI.*
37. I: You only treat those who have come for circumcision?
38. *R: Yes. Only men*
39. I: Let’s take for example, this clinic is offering STI screening, as men can be coming with their partners or a boy can also be coming his girlfriend and if the boy has been found with an STI when he comes for circumcision, the girlfriend should also be treated for STI. What is your opinion about that?
40. *R: okay, it be a very good thing, in addition, I think people can welcome and accept it well. I remember a certain time I had some cases, were a man could come for circumcision and found with an STI. So as usual, when a man is found with an STI, he is supposed to come back with his partner for treatment. So they come as a couple to receive treatment. After a man is treated for an STI, he gets circumcised but in the process, both get helped as a couple. If this can come as a special programme for STI treatment, it could be a good thing… ehm it is a very good thing.*
41. I: Okay, fine… so what do you think are the barriers and concerns on this integration?
42. *R: Ehm… the big problem, could be for a woman to accept and take it, that his husband has been found with an STI, because it could be a thing which she was not expecting as his husband left for circumcision. But maybe, she could try to understand. If the programme could be special for STI, it could be a very nice program. Ehm... Because it could not be a new thing, as they could be coming for a special kind of treatment for a particular STI.*
43. I: Okay, like you a Health service provider, where do you think could be a problem, not only to the clients but to your part. Where do you think can be a problem?
44. *R: Hmmm… I don’t think there can be any problem because the drugs for STIs are available here. So there is no any problem concerning that.*
45. I: So what do you think should be done to address these barriers?
46. *R: Hmmm… on that part, there is a need to sit down and talk with level officers… It can be starting with level number two of HTC, since after HTC, that’s when we do have screening for an STI. So it can involve HTC and those who deals with screening before circumcision. Because what happens, it is just steps before circumcision, after HTC then screening and it is screening which makes a person known if he has an STI, and if he is found with an STI, he starts receiving the treatment of that STI. So it can involve HTC and screening.*
47. I: Okay, so do you think, there can be any concerns involving the integration of STI services?
48. *R: aaah, no. There is no any concerns or worries*
49. I: Okay, now let’s talk about family planning. What happens if a client needs a family planning method? For instance, a man has come with his female partner… so either a men or his female partner is asking you that I need to practice family planning, what happens?
50. *R: Hmmm… who have asked like a general question right?*
51. I: Yes, in general
52. *R: hahaha…. (Laughing) of course…. Okay, fine. Ehm, so they come as a couple, that they want family planning after they have agreed as a couple. So we see the method which they can take for family planning.*
53. I: Let me ask you in this way, for instance at this clinic, they can come a men and his female partner, either for circumcision and in the same process they also want to practice Family planning, what do you do?
54. *R: Okay since currently we don’t provide Family planning services, I can say, they might be helped by referring them where they do provide family planning services.*
55. I: As for you, this has never happened? Like that people come asking about family planning services?
56. *R: No, it has never happened here that people come asking about Family planning.*
57. I: So, you have explained that you do refer them?
58. *R: Yes, if they want to do family planning, you can go to this and that place.*
59. I: so, what if we integrate it here at this clinic as special service for family planning.
60. *R: Ehm... So that will be circumcision, STI screening and Family planning… okay fine, I think it can work well*
61. I: What is it that you like of the integration of Family planning with Voluntary Medical Male Circumcision Services? How is it connecting?
62. *R: Ehm… It is connected as when they agreed and come as couple for circumcision, it may be connected with that of circumcision and also Family planning won’t be bad to be included.*
63. I: Okay, but what could make you not to like integration of family planning in Voluntary Medical Male Circumcision Clinic?
64. *R: Aaah may be, because the work can be too much...Hahaha (Laughing)… at the same time you have to do this and that. The following cannot be possible and maybe the purpose of the project cannot be achieved because of that. So the problem can be in that part.*
65. I: So what do you think could be the best way of providing the Family planning services on the issue of circumcision?
66. *R: Hmmm you can just pilot it to see how it is working, then if it works, we can continue*
67. I: Okay, let’s talk about cervical cancer screening. Have you ever heard about that?
68. *R: Yes, I know it*
69. I: So what happens if a woman needs cervical cancer screening? If a woman finds you at this clinic
70. *R: Here we don’t provide such kind of services. So if she comes, we just refer her where she need to go to get a help. Because of now, we don’t screen for cervical cancer.*
71. I: what if we integrate cervical cancer screening here?
72. *R: that will be very good thing to do as one of the advantages for circumcision, helps women to prevent getting cervical cancer. So if it can be integrated in this project, it can be wonderful. And more women can surely understand the circumcision of their husbands, so that they can be encouraging men to go for circumcision. So that can be perfect …. hahaha [Laughing]*
73. I: So, what is it that you would not like about cervical cancer screening integration with Voluntary Medical Male Circumcision services?
74. *R: Aaah, maybe …my concerns could be …aaah but not that much, maybe staffing…maybe because of few staff, there can be some delays. But besides staffing, there is nothing to worry about.*
75. I: What could we do to deal with such kind of the concerns?
76. *R: what is needed is planning and recruiting more clinical staff.*
77. I: Do you think there is any barrier for integrating cervical cancer screening?
78. *R: Aaah no, I don’t think there can be any barriers.*
79. I: Now, let us discuss about PrEP. Have you ever heard about PrEP?
80. *R: PrEp?? Aaah … yes, quiet a lot.*
81. I: Okay, so what did you heard about PrEP?
82. *R: Hmmm… if am not mistaken, you are saying PrEP with R right? Can you just explain a bit about it? Maybe it is a different PrEP which I know.*
83. I: Okay, if we say PrEP is anti-HIV medicine that keeps HIV-negative people from being infected. So there is a single pill that is taken once daily, and if you take it regularly, it is highly effective at prevention people from being infected. While PEP, you take it after you have been exposed or injected at the moment.
84. *R: I do know PEP and not PrEP*
85. I: So have you understood?
86. *R: Yes, like a lot*
87. I: So, how can you explain about PrEP?
88. *R: PrEP is anti-HIV medicine that keeps HIV-negative people from being infected and there is a single pill that is taken once daily.*
89. I: Okay fine. If PrEP was made available to HIV men and women. Do you think you could advise your HIV negative clients to accept to take PrEP?
90. *R: Yes, like a lot because it will help those people not to take a virus but is it possible for everyone to be getting it?*
91. I: Yes, as long as he or she does not have a virus.
92. *R: It could be a good thing; it could be a good thing.*
93. I: What are your concerns?
94. *R: My concern could be that; some people may be careless like doing whatever they feel like doing because of it.*
95. I: Okay, assuming the PrEP is available, what is your opinion on integrating PrEP with Voluntary Medical Male circumcision services? Would you encourage clients to take it?
96. *R: It could be a welcome idea because circumcision helps to prevent HIV with 60% and integrating it with PrEP, there can be a great improvement with 100% achievement. It can be really welcome and people can be happy about it.*
97. I: How do you think PrEP be offered in Voluntary Medical Male Circumcision clinics?
98. *R: I think it can be offered to each client who come for last review of six weeks and it could be better if it is provided by HTC.*
99. I: Okay, let us talk about other services. If you were given powers to choose and integrate services in Voluntary Medical Male Clinics, what are the services that you would think of Integrate?
100. *R: Excluding what you said here?*
101. I: Yes, excluding what I said here or you can also include what I have already said here.
102. *R: Oh as for me, I could choose, integrating circumcision with cervical cancer screening, PrEP and I would also be happy if they come as a couple for circumcision as a family. I would be happy.*
103. I: why have you chose to be coming as a couple?
104. *R: I chose couple because there are prescriptions which are given to clients to follow, which is needed to be given to his wife as a family and they could be helping out one another in terms of what to do. If one is not doing it in the right way, he can be reminded by the partner. So I could be excited about that as a couple.*
105. I: Why have you also chose cervical cancer screening?
106. *R: Because one advantage of circumcision is to protect a woman from cervical cancer, therefore if screening is done here, it would be like we are achieving what we intended to achieve.*
107. I: So PrEP, why have you chose PrEP?
108. *R: Because you might find that, we say circumcision it helps to protect us from HIV with 60% and we say 40% it is about using condom but if we use PrEP, instead of a condom it can still work out.*
109. I: Okay, you are saying men should be coming here with his female partner, how will things be or work like at this clinic? Because there might be some concerns. How can it work like so that men should be coming here with his female partner?
110. *R: Men might be coming with their wife, and I do not think there might be any concerns. All the steps we talked about, from registration, screening...his wife will also be there, until it is done at procedure, then they go. Those who want, they come as a couple but what is needed is just to encourage them that it should always be like that.*
111. I: Okay, so where should cervical cancer screening be included?
112. *R: It can be placed under screening, as men is being screened for STI, before circumcision, maybe that’s when even a woman can also be screened for cervical cancer. Which means, they can be leaving with a good news at the end.*
113. I: Okay, you have explained clearly. Thank you for taking your time to discuss with me today. Your answers will be very helpful in improving the health service delivery at Voluntary Medical Male circumcision clinics. Before we close, do you have anything to say?
114. *R: I would just like to ask on the issue of PrEP you talked about, did you say the medicine are available or you are searching for it? Because this is my first time to hear about PrEP, I only know about PEP.*
115. I: The drugs are available but they have not yet started distributing.
116. *R: Okay, but are found here in Malawi?*
117. I: Yes, are available here in Malawi.
118. *R: Oh okay.*
119. I: Sure. So again, thank you so much for taking your time to speak with me.

**D 43 STUDY**

**Date of Interview: 14 August 2018**

**Type of Participant: Provider**

**Interview Number: D-43-0024**

**Interviewer: I.N.**

**Total Interview Time: 43 minutes 51 seconds**

**Interview Summary:** **(from summary sheet)**

| **SERVICE TO BE INTERGRATED** | **THOUGHTS ON INTERGRATION** |
| --- | --- |
| Couple HIV Testing and Counseling | Thinks its good development because there is a total participation on the wound care and on the counselling as well. |
| STI Services | Thinks it’s a good idea because when you are dealing with a man, circumcision, it’s all about men. Any complications that arise, in, in a man, has to be managed within a site. So, it’s good that the STI and the circumcision are done concurrent. |
| Family Planning | Thinks it is a good idea as it will lead into men empowerment since most men do not participate in family planning. |
| Cervical Cancer Screening | Thinks it’s a good idea as circumcision benefits more men but at the same time the women, they are also benefiting from directly.so, for women to be sure that they are cervical cancer free and they are willing to undergo screening, it’s good to do them at the spot [VMMCC] |
| PrEP | Like the idea of integrating PrEP with VMMC services but on a condition. PrEP can also have negative impact to the clients. |
| Other Services | Thinks all services should be integrated into VMMC as they fuse well |

**Remarks:**

**Participant was relaxed, confident, knowledgeable, explained clearly and in brief.**

**Interview Text:**

1. I: Aaah, thank you for taking the time to talk with me today. I would like to ask you some questions today about the way you feel and what you think about some issues related to the service you provide and how we can include other services in Voluntary Medical Male Circumcision clinics. There are no right or wrong answers to these questions. We would like to hear your opinion and your experiences in your own words. Aaah, do you have any questions before we begin?
2. *R:Aaah, no*
3. I: Okay…can you tell me how you are involved in the client care at this clinic?
4. *R: can you come again the question?*
5. I: can you tell me how you are involved in the client care at this clinic?
6. *R: Okay basically, I… aaah, we see, we see most of patients who come especially for circumcision. Yea, our role is to do the screening, we also sometimes participate in group counselling, like the client flow; they come from registration, the group counselling, then screening. Then screening to the procedure, the actual operation. And also participate in the review of the clients like post op checkup. And also manage STIs and we also refer clients for other services ART and other complications that we may not be handled here.*
7. I: Okay. Does your clients talk to you about how the services are provided here?
8. *R: Aaah, directly no, but sometimes we do conduct like a survey like we have a suggestion box, we could use as IQ we call it… [Not clear] continuous quality improvement. So, we do check the feedback that they do give out periodically but directly no.*
9. I: Yea but can you give me an example of a time that your client talked to you about the services he received here? Any example
10. *R: Any example?*
11. I: yea
12. *R: Okay, like the feedback?*
13. I: yea, like the feedback, about the services that a client received here at this clinic
14. *R: Okay, so far I haven’t got back a feedback that a client have given, aaah but most of them, they do appreciate the services, though sometimes, due to workload, we cannot absorb all the clients at once because most of them, they do come at once. For instance at this morning, there is no clients but most of them , they do come after ten, yea, so we are found to have more workload in the afternoon than in the morning, so that compromise time of stay and for their services.*
15. I: Okay. Now let us talk about partner HIV testing here at the Voluntary Medical Male circumcision clinic.
16. *R: ehm*
17. I: Tell me what happens if a man brings a spouse here at the Voluntary Medical Male circumcision clinic?
18. *R: Yea, it’s it’s a welcome development and they do participate and its done perfectly*
19. I: so, you have seen a man, bringing his own spouse?
20. *R: yes and we even encourage that those who are willingly, they can come together or even for STI, we also… we have been encouraging our clients to come with the partners.*
21. I: okay. What do you think are the motivators that make the men bring their spouses here for testing?
22. *R: Okay, aaah, basically, it’s aaah like one stop, most men, they don’t have, health seeking behavior, once they come here, they feel if they can do everything. Yea, so they are much comfortable. Just to be seen here, everything is done, then off they go.*
23. I: okay, so what are motivator there? What motivates them to bring the spouse?
24. *R: Ehm, its more confined service, confinement, yea, like we do… aaah, we do provide service which is directly to their needs. They just come here to get service that is related to male but also which is also linked to their spouses.*
25. I: Okay, for those who do not bring their partners, what do you think demotivates men to bring their partners here for HIV testing and counselling?
26. *R: Aaah, much we can’t tell because we haven’t come across like maybe, aaah cross examination on them but sometimes because there are more men, yea, so maybe we don’t have a place to keep them while they are waiting because there are more men. So if few women comes here it will be like…aaah the spouses are kind of maybe... yea. But then we also introduce the VIP service which at meantime is the… work policy HIV/AIDS work policy…aaah, this system, we were doing the clients, giving the services to the clients on their convenient time. So they could make a phone call and book when they want to be seen to be circumcised. Yea, that also gave men another advantage. They do come with their spouses those who are willing because it’s their board time.*
27. I: Okay. So what do you think can be done to make men bring their partners here for couple testing and counselling?
28. *R: Aaah, I think that can start with demand creation because of the awareness when imparting information to people to come for circumcision. If we also include that one during the sensitization period, I think they should be able to bring their spouses, yea... most of them, they should be able.*
29. I: Okay. What do you think on your own are the ways besides which are already implemented?
30. *R: Aaah that involving the partners, the spouses, aaah…. I think that’s the only … okay, it’s a matter of information that we give them. If we include to say, bring your partners, whether on the radios, whether we are using public addressing system or we are talking to the women at the antenatal or OPD, because we do capitalize on where the patients gather, the clients, anywhere within the hospital premises, in the schools, yea.. So all those places or in the gathering like through the chips, yea all the places, if we do tell them, come, bring with your partner, yea, I think they should be able to bring.*
31. I: Okay. You as a health care provider, what is your opinion on integrating couple counseling with Voluntary Medical Male circumcision services?
32. *R: sorry*
33. I: You as a health care provider, what is your opinion on integrating couple counseling with Voluntary Medical Male circumcision services? Which are already available
34. *R: yea, it is a good development, aaah, because there is a total participation on the wound care and on the counselling as well. But then, it also have a hiccup because it need more human resource because I don’t know whether couples maybe like we were to mix up couples, if maybe there are ten couples come together with their spouses, they do counselled, I think that’s not a problem but if you are targeting one individual, couple to be counselled separately, then I think that should be… should give us a workload. But if they can be coming as group, okay if they are many, they have come like couples and you counsel them, that shouldn’t be a problem because of the same information.*
35. I: Now I would like to discuss with you about sexual reproductive health services and Pills for HIV prevention: called pre-exposure prophylaxis. (PrEP) Sexual reproductive health include services that promote good sexual health and reproduction. They include but not limited to family planning, cervical cancer screening sexual transmitted infection (STI) management, and many more. Today we will only discuss about family planning, diagnosis and management of STIs, Cervical cancer screening, and PrEP. We will look at each of these one by one. Let us start with STI services. Explain to me what happens if a client is suspected or diagnosed with an STI here?
36. *R: Okay, if a client is diagnosed with an STI condition, aaah, we do manage the client and we also get the details and put in in STI register and we also cancel him on safe sex and also prevention of sexually transmitted diseases like HIV. We also undergo, for HIV test and at the same time, we also encourage him to bring a partner, whether he has multiple partners or single partner or all of them, we encourage to let them come here or if he cannot manage to bring them here, he can go to the nearest health area… we also give him a referral to give to the partner, STI referral, to give the partner to show any clinic if he may go to any other health facility.*
37. I: Okay, so you as a health care provider what is your opinion on integrating STI services with Voluntary Medical Male circumcision services?
38. *R: Aaah, it’s a good, it’s its opinion, I mean it’s good to integrate the two because when you are dealing with a man, circumcision, it’s all about men. Yea, so any complications that arise, in, in a man, has to be managed within a site. So, if, if, the client is managed at the site, and after all, we also tell them that, after seven days, after managing the STI condition, they should still come to get the service that they came for. Like okay, the client came for circumcision, and because they have been found with an STI condition, we can’t do that. So we treat him and we say, after seven days come back we do the circumcision. So, it’s good that the STI and the circumcision are done concurrent, yea.*
39. I: Okay. What is it that you do not like the integration of STI with Voluntary Medical Male Circumcision services? Why would you not accept the integration?
40. *R: Aaah, hmmm… no, I think there is no other reason why I would not accept, for accepting to be integrated*
41. I: Okay… [Turning over the papers] Hmmm… How do you think STI services should be offered at the Voluntary Medical Male circumcision clinic?
42. *R: Okay, one is privacy. Aaah, as I have said, men have tendency of low health seeking behavior. So, if they come for circumcision, most are actually, most of them, they may not even know that they have that condition. They just come. Yea, but because, this is specifically for men, yea, so they feel comfortable and it’s not even mixed up with any other clients and at the same time, the time, it’s not time consuming… they don’t have to queue yet they know they have a programme.*
43. I: Okay. So, where exactly? Here at Voluntary Medical Clinic should it be, put in terms of place… should it be within the hospital or outside or in the same building but the private room?
44. *R: At the circumcision?*
45. I: yea, for STI service, where should it be in terms of place?
46. *R: Aaah, it should be within the premise because, as I have talked about the client flow, yea, any client before going for the operation, theatre, here they undergo screening process. So during that screening process, it is where this client is diagnosed with the STI as a contradiction to circumcision. So, immediately after…. being found with the contradiction being STI, he is managed right there, rather than referring him to another section.*
47. I: So, what do you think are the barriers and concerns on this integration?
48. *R: Aaah, barriers… barriers are… ehm, okay, meantime, we are focusing on circumcision?*
49. I: yes
50. *R: And it may happen that maybe, after circumcising, or maybe those already circumcised, they would still maybe, get an STI, and they will still need to come and get treated. Yea, so, in that case, there is need just to … modify the client flow. To say maybe okay, those who have come for consultation, they have to go through this because we are capturing these clients from the screening but they may come just straight to say I have an STI, so those ones have to go straight to the STI. So, I think that one will also, like…ehm yea, human resource. Will increase human resource and… maybe to ladies, yea, to ladies as barrier, those who are maybe, they may not feel comfortable to be combined with men, if because ladies will not come for circumcision. They will just go straight for the service, they may feel that they are not part and parcel of men.*
51. I: Aaah, now let us talk about family planning. Explain to me what happens if a client needs a family planning methods?
52. *R: Aaah… family planning methods? Like here?*
53. I: yes, like here
54. *R: Aaah, it depends with the kind of family planning methods. Yea, for men, the family planning methods that we give here are condoms… yea and also abstinence. We do give, yea, we can counsel the client on types, I mean the methods they can choose. But we only have one method, which is condom and abstinence. Yea, but if it is a family, meaning that, come with a woman, then we don’t provide like these other methods, so we do refer them.*
55. I: So, you don’t conduct like vasectomy?
56. *R: Aaah, vasectomy no, we are not trained… yea, we don’t offer but we, the problem is like there is no package… yea, the… it’s not in the minimum package of VMMC, I think it’s not included. So if the policy can change to say, we put vasectomy as a minimum package, then I think that can be taken care of.*
57. I: Okay. As a health care provider, what is your opinion on integrating Family planning in circumcision services?
58. *R: Hmmm, yea… I think, it can bring like male empowerment involvement so to say, because most men do not take part in family planning methods and that’s why maybe vasectomy thing, it’s almost, the dead topic. Because men they don’t participate. Most participating in family planning are women. So, yea, I think it should be involved…*
59. I: why do you think most men do not like participating in family planning?
60. *R: Aaah, okay, I think it is the setup, because men don’t have a clinic. Men, you will find that in the hospital, you have, there is male or female ward, pediatric ward, labour ward… so three quarters of hospital setup are women, women, women, and kids. Men don’t have specific ward, its only VMMC clinic, which men can say this is our clinic. So because of that, and we don’t take opportunity to give information to, when they come for VMMC and family planning, they are also let out without participating.*
61. I: Okay. What is it that you would not like the integration of family planning in Voluntary Medical Male Circumcision Clinic?
62. *R: Aaah… it may be, time consuming. Like… hmm, for instance, when a man is only coming for circumcision and undergoes screening, counselling session of family planning, method choosing, yea, I think maybe they may fee bored. Yea but if we can include information in counselling, during the counselling session, should be a problem. But I think the only problem is time. Yea, I think they will take time to be assisted.*
63. I: yea. But you like the idea?
64. *R: The integration?*
65. I: yes
66. *R: yea, yea*
67. I: can you give me just one thing you like about this integration
68. *R: Okay, ehm, it’s a good idea and its one stop. One stop shopping, you come, you get everything. You get information about circumcision, you get information about family planning, yea... you get information about STIs, you also get information about…. Yea these three. So it’s like you come and it’s like, the health services, they will be like very optimist to utilize that time, to dish out the information. SO, it’s a good idea.*
69. I: Okay. How do you think family planning services can be offered within Voluntary Medical Male circumcision clinics?
70. *R: Ehm, I think, it’s just a way of devising a good client flow… yea. If they come for family planning method, no I should say, they go at the reception, aaah… they register there without classifying which area they had to go. And then, in the counselling session, all the general information is given and from there they split. Those who have come for family planning, they go that way, and then those who have come for circumcision, they go different way. Yea, I think like that kind of setup.*
71. I: Okay, so, do you think, it should be within the same building or outside?
72. *R: It should be the same building but just splitting the services. They should be like one roof.*
73. I: Okay. On the issue of barriers and concerns on this integration, what do you think are the barriers and concerns of this integration?
74. *R: Stigma. Stigma might come out like okay, to say those who are found with STI conditions, when they are going for treatment and then their fellow friend have seen them, they like okay, if you go that room, you have an STI condition but here, the good thing is that we combine the checkup, reviews, we remove the bandage, we do the seven days, 48 hours and six weeks check in the same room in the same room that we provide STI medication. Yea, so nobody they know that you have come for STI treatment, checkup… the stigma then is kind of bad.*
75. I: But what do you think should be done to address that concern of stigma?
76. *R: Stigma, aaah yea, the integration will kind of reduce the stigma because it’s in one room but you are getting all the services that you need, nobody can tell that you have gone that direction because of this… yea.*
77. I: Now let us talk about cervical cancer screening. Explain to me what happens if a woman needs cervical cancer screening?
78. *R: Like here or…?*
79. I: Like here
80. *R: Aaah, normally like Bwaila hospital, clients are being referred to antenatal, where they do provide screening.*
81. I: Okay. Aaah, do you have any experience about cervical cancer screening?
82. *R: Aaah no… I have not worked much in the screening department*
83. I: okay, or any knowledge, rumors, maybe you heard somewhere about cervical cancer, what happens
84. *R: Aaah, like actual procedure?*
85. I: yea like actual procedure when a woman need cervical cancer screening
86. *R: Aaah, okay, I think that much…. Am totally blank but what I know is that, those who are of age of child bearing, they do come and do, aaah they are eligible for cervical cancer screening. And there is also challenge that I have noted, most of studies or outreach that we conduct in several health centers, when disseminating information about circumcision, because cervical cancer is also a component of… as a benefit of circumcision. So, when we tackle that topic, women, they pose a lot of questions and they are ready to get the service but there is nobody to provide the service. If the system is integrated, the same providers that are providing for circumcision can also provide for cervical cancer screening, I think we can be home and dry, because there are so many women there and women are so willing to participate. But we don’t have the expertise within the VMMC. So it’s a good idea.*
87. I: so on your own opinion, if cervical cancer screening is included what would you like in this integration? What would you like about this integration?
88. *R: it’s the same, it’s like we are willing the same birds, men, women. Circumcision benefits more men but at the same time the women, they are also benefiting from directly… yea, so, for women to be sure that they are cancer free, cervical cancer free and they are willing to undergo screening, it’s good to do them at the spot… yea*
89. I: What is it that you would not like about cervical cancer screening integration with Voluntary Medical Male Circumcision services?
90. *R: Aaah… I don’t think there will be something that I will not like because most men who are coming for circumcision, most, they are married and also some are not married but they are actively involved in sexual intercourse, so they help each other, kind of.*
91. I: so, how do you think is the best way to offer cancer screening within Voluntary Medical Male circumcision clinics?
92. *R: The best way is the same of devising the client flow, client flow format… yea. So, it all starts from the same registration, all clients register. From there, they go for counselling session. So, counselling session, from counselling session they do the HIV test and then they split up to specific areas that they want to.*
93. I: Okay. What do you think are the barriers and concerns on this integration?
94. *R: Barriers will be like, those men will be coming without spouses, and maybe they will feel shy, seeing other women seeing them… yea. That’s the only barrier.*
95. I: Okay. So, what do you think should be done to address these concerns and barriers?
96. *R: I think with counselling, counselling they will be able to accept it.*
97. I: Okay. Now let us discuss about PrEP. Have you heard about this before?
98. *R: Yea*
99. I: can you tell me, how did you learn about this?
100. *R: Aaah, it’s a package on VMMC… yea and also… though we don’t provide it here, yea but under ART training. They do train people about PEP and also… I think… healthy talks, health educational topics I think, it’s… I have also heard about that as well*
101. I: so, what did you say PrEP is?
102. *R: Okay… PEP is Post Exposure Prophylaxis….yea. Actually it’s a medication given to somebody within the period of seventy two hours… yea. If maybe you are involved in unprotected sexual intercourse with somebody you don’t know the status, you are required to take the medication, within the seventy two hours period, for a period of a month yea.*
103. I: Okay. That’s PEP and not PrEP.
104. *R: aaah that’s PrEP?*
105. I: yes
106. *R: then am confusing*
107. I: yes, what you are saying its PEP and not PrEP. If you have never heard of PrEP
108. *R: I think I have never heard of it*
109. I: I will explain how the medicine works. PrEP is anti-HIV medicine that keeps HIV-negative people from being infected.
110. *R: That keeps?*
111. I: that keeps HIV-negative people from being infected. I can give you an example. For instance, a man might be negative, he might be knowing the status of his wife, that she is positive. After taking the PrEP, they can be having unprotected sex but without getting a virus.
112. *R: more less like a discordant couples?*
113. I: yes. You know the status that my partner is HIV positive but am negative, let’s be taking PrEP…yea. So, how does the PrEP works? There is a single pill that is taken once daily, and if you take it regularly, it is highly effective at prevention people from being infected. But you take it once daily and you take it regularly
114. *R: for life*
115. I: yea for life. In that way, a virus cannot be passed to your partner
116. *R: okay. Effective rate… does it have effective rate?*
117. I: Aaah, honestly am not sure about that you can talk to the doctors
118. *R: It is already on the market?*
119. I: Aaah, it’s not yet on the market but maybe soon might be available. But it has not yet started being released
120. *R: yea [smiling] I think it’s a good.*
121. I: But we just want your opinion. How do you think about that
122. *R: I think that will be good because there are so many discordant couples out there but they are burned from having unprotected sex because they are afraid of contracting virus from their partners… I think most discordant couples will be happy again.*
123. I: If PrEP was made available to HIV- men and women. Do you think you could advise your HIV negative clients to accept to take PrEP?
124. *R: Yea, yea yea… I think …. With good orientation, if the providers are trained and the government also has to include that one in the policy, yea I think we shouldn’t having any problem. And I don’t think people will reject it, it’s a good idea.*
125. I: What are the reasons you would encourage your clients to take PrEP?
126. *R: Reasons?*
127. I: yes
128. *R: okay, aaah, I think the reason is… the first indication is discordant couple. I think, if it’s meant for discordant couple, fine but there is anticipated abuse by those who we don’t know their status and they may substitute that one PrEP to PEP. So, it can prone to abuse if people are not well informed… yea*
129. I: How do you think PrEP be offered in Voluntary Medical Male clinics?
130. *R: Like the actual service?*
131. I: yea, like the actual service. How do you think should be offered here at Voluntary Medical Male Circumcision Clinic? Like in terms of place, time
132. *R: Okay… like because we circumcise HIV positive clients, yea so it’s possible that those with HIV positive and their partners with not HIV positive, I think they can be give them medication, I think the indicated clients can be those ones they have come for other services but…it can also be given at …..Clinic as well.*
133. I: Okay. Should it be given to only the clients or everyone?
134. *R: while that depends also on policy… on the effectiveness …yea, if given to everyone, on note of HIV prevention like this song of 90 90… yea, I think it’s not a problem. Because now we look at other factors like resistance, if there is no fear of drug getting resistance, then that should be a problem. Because it will be like flooding everywhere, given to everybody people will use it and then maybe it develop resistance to that individual after being exposed to several viruses after meeting and again it may encourage promiscuity, if it’s just given anyhow to anybody.*
135. I: Okay. So, what do you think should be done to address those concerns or barriers?
136. *R: Aaah… I think we need civic education, a lot of civic education. Need a lot of ground work both to the providers, I mean, health care people and the community out there*
137. I: Do you have any concerns or barriers on this integration of PrEP with Voluntary Medical Male Circumcision Clinic services?
138. *R: No… I think that one it has an entry point. There is no problem it can be.*
139. I: Let’s now talk to our last part on other services. If you were given powers to choose and integrate services in Voluntary Medical Male Clinics, what are the services that you would think of Integrate? Maybe some of the services which we just talked about which do you select? Which service will you integrate?
140. *R: Like the… ooh I think I have… all of them, they should be integrated.*
141. I: Explain to me what the reasons are for your choices
142. *R: Because… the services itself, its fusing to each other well. Combining them, it’s a one stop shopping, if you come, you have an opportunity to have an information of all the services that are pertaining to reproductive. It has a good entry point.*
143. I: Okay. So, how do you think these services should be offered in the clinic?
144. *R: It can be offered concurrent… yea. It can be offered concurrent as people come, they just go to specific areas… if am looking for circumcision, that is after getting the primary service of group counselling, HIV testing, then they split for specific screening.*
145. I: Thank you for taking your time to discuss with me today. Your answers will be very helpful in improving the health service delivery at Voluntary Medical Male circumcision clinics. Before we close, do you have anything to say?
146. *R: Just a question on… how soon or maybe if this is to be approved? How soon it may start?*
147. I: Aaah honestly, I can’t say the period but it will depend on how soon we have completed the survey yea, and after that if it has been approved, that’s when they will communicate… yea
148. *R: alright*
149. I: yea. Anything else
150. *R: No, no, no.*
151. I: Again, thank you so much for taking your time to speak with me
152. *R: you welcome.*
153. THE END

**D 43 STUDY**

**Date of Interview: 14 August 2018**

**Type of Participant: Service Provider**

**Interview Number: D-43-0025**

**Interviewer: I. N.**

**Total Interview Time: 48 minutes 33 seconds**

**Interview Summary:** **(from summary sheet)**

| **SERVICE TO BE INTERGRATED** | **THOUGHTS ON INTERGRATION** |
| --- | --- |
| Couple HIV Testing and Counseling | Things it’s a good idea. |
| STI Services | Thinks it’s a good idea despite that its already there |
| Family Planning | Thinks it’s a good idea as it will to copy or bring more men |
| Cervical Cancer Screening | Thinks it’s a good idea. |
| PrEP | Thinks it’s a good idea, as it will prevent negative people from being infected. |
| Other Services | PrEP and family planning |

**Remarks:**

**Participant was confident, open and relaxed. She emphasize much on capacity building and not just integration.**

**Interview Texts:**

1. I: Thank you for taking the time to talk with me today. I would like to ask you some questions today about the way you feel and what you think about some issues related to the service you provide and how we can include other services in Voluntary Medical Male Circumcision clinics. Aaah, there are no right or wrong answers to these questions. We would like to hear your opinion and your experiences in your own words. Do you have any questions before we begin?
2. *R: No*
3. I: Aaah, can you tell me how you are involved in the client care at this clinic?
4. *R: Okay, ehm… am VMMC provider. So, I do screening, providing the… VMMC, reviewing my clients… and other duty that will be assigned. I look at STIs, that’s when we are screening and… yea, any other conditions that comes with.*
5. I: Aaah, does your clients talk to you about how the services are provided here?
6. *R: yea, some do some don’t*
7. I: okay. Can you give me an example of a time that your client talked to you about the services he received here?
8. *R: ….*
9. I: anything
10. *R: anything?*
11. I: yea, any example
12. *R: okay… some do ask a lot on how the procedure is conducted, that’s the main factor because they really want to understand what we really do and how it is going to be performed and some focus much on they are going to perform like in terms of sex after we have done circumcision*
13. I: okay
14. *R: yea. Some they even reach up to the results like how, are they going to be productive or are they going to be getting the STIs again, HIV again after circumcision, that’s mainly what people talk of. Some do ask about complications like if you have late healing of the wound infection, what we are going to do. We discuss all that so that we array the anxiety and we reassure them of the, of what we are talking.*
15. I: okay.
16. *R: yea.*
17. I: Aaah, now let us talk about partner HIV testing here at the Voluntary Medical Male circumcision clinic. Tell me what happens if a man brings a spouse here at the Voluntary Medical Male circumcision clinic?
18. *R: mainly, we do screening to men only because that’s our focus*
19. I: okay
20. *R: yea. Had it been we integrate all those, we could also be involving the women. But, if the women says, I also want to be tested because it’s a clinic, it’s a hospital… yea we do provide the service. But if they won’t ask, we just think as any other guardian so we don’t do.*
21. I: okay
22. *R: yea*
23. I: But have you seen a man coming along with a partner?
24. *R: yea*
25. I: for HIV testing?
26. *R: yea*
27. I: okay. Aaah, what do you think are the motivators that make the men bring their spouses here for testing?
28. *R: okay, sometimes I feel like it’s the woman that persuade other guys to come for circumcision in the first place. So, they are also the ones that say I want to know my status, whatever thing. So some say, let’s do that because of that reason mainly, because we are also educating the client on women especially on the… VIP testing. So, most of them say, my husband go for circumcision, and I want to know my status or whatever thing, so most of men do for, it’s because of women themself*
29. I: okay
30. *R: yea*
31. I: Alright. Aaah, for those who do not bring their partners what do you think demotivates men to bring their partners here for HIV counselling and testing?
32. *R: I don’t really see as a demotivation because I feel like, we are not providing much information on the emphasis of bringing the woman to the VMMC clinic… yea. Had it been that we are also focusing on married men bringing their spouse or that have girlfriend or whatever partners to bring their relationship here, maybe I could have said there is that gap but our emphasis was, is men and if a man come to the clinic, we accept him. So I can’t really say it as a gap, had it been that we are given the information but the women are not coming, yea it could be a gap to me.*
33. I: okay
34. *R:yea*
35. I: Aaah, what do you think can be done to make men bring their partners here for couple testing and counselling?
36. *R: Aaah I think first and foremost thing is to give them information, that you need to bring your partner because most of times we share the information if the partner has STI*
37. I: okay
38. *R: yea, not HIV positive, not HIV negative but only because we also want to treat the partner so that they don’t re-infect each other. So I feel like giving information to the men, when we are sensitizing about VMMC, we can also include that information to encourage the men to be coming with their partners so that they should be having HIV testing together. I think that can be much easy. If they won’t be showing up, we are going to consider that this is a gap*
39. I: okay
40. *R: yea*
41. I: You as a health care provider, what is your opinion on integrating couple counseling with Voluntary Medical Male circumcision services?
42. *R: I didn’t get the first part of the question*
43. I: You as a health care provider, what is your opinion on integrating couple counseling with Voluntary Medical Male circumcision services? If we integrate couple HIV testing and counselling with already available services at circumcision clinics, aaah, what is your opinion about that integration?
44. *R: I feel it’s good because mostly we only test a man and when he is negative we assume everything is okay but we don’t know the status of the woman. So I feel it can be good if we incorporate that into what we are already doing because we are already doing the HIV testing but the challenge may be that we can now have congestion, if every man accept and feel it’s necessary… yea, it means we need to build our self, like in the time of service provision, we need to have more counsellors, we need to have yea but for the integration, it’s really good that we know the status of both people, that we should know how to handle them better*
45. I: okay
46. *R: yea*
47. I: What do you think are the barriers and concerns on this integration?
48. *R: I think the first is what I have already said that we can have an increased workload like already, am giving much example on STI thing because that’s what we do most… yea. Today you treat a man, tomorrow, the woman, the wife maybe the other partner also, the other partner also and they are females while we much focused on males. So, its quiet heavy on us but we do it. So, I believe that’s the main challenge that we can be facing. All we know, we are providing the service and it’s good that these people are being helped.*
49. I: okay fine. So, on that part of the challenge you have just explained, what do you think are the solutions or what do you think should be done to overcome the barrier you have just stated?
50. *R: I think if the integration is accepted, the better way to handle is just to increase the resources and to increase human resources again because we can’t say we should exclude these people while these people are also infected. I feel it’s good to integrate but look at human resource and the resources that we are going to have because let’s say in the STI thing, I have mentioned that we also treat females but mainly, when the female is lactating or is pregnant, we don’t give the same medication that we give to men. While the only medication that we have, will only targeting men, now will be telling that woman, go and buy this medication, which if we have all the resources, it will be easier for us because we also doubt that, is she really going to buy, is she really going this estate because we are not sure, you know Malawians that she is really going to buy medication but if they are going to consider the resources, the human resources and everything else, I think yea*
51. I: okay. Aaah, now I would like to discuss with you about sexual reproductive health services and Pills for HIV prevention: called pre-exposure prophylaxis (PrEP). Sexual reproductive health include services that promote good sexual health and reproduction. They include but not limited to family planning, cervical cancer screening sexual transmitted infection (STI) management, and many more. Today we will only discuss about family planning, diagnosis and management of STIs, Cervical cancer screening, and PrEP. We will look at each of these one by one. Let us start with STI services. Explain to me what happens if a client is suspected or diagnosed with an STI here?
52. *R: okay, we have our guide line… and mostly, most of us who went through medical nursing school, and are trained on job to provide STI services, we are trained and we are capable of providing the service. So according to the guideline we document and record our data that these are the STIs that we have seen this month.*
53. I: Okay. So what if a client is found that is with an STI, let’s take a client come for circumcision and in the whole process, he has been found with an STI, what do you do to that client?
54. *R: we treat the STI first.*
55. I: okay
56. *R: and give him the next appointment to come for circumcision. Some do come, some don’t*
57. I: Okay. Aaah, you as a health care provider what is your opinion on integrating STI services with Voluntary Medical Male circumcision services?
58. *R: This, because we have been doing this, I feel it’s really good that we are catching it there and then because as I have already said, we do give them the next appointment, some do come, some don’t. Some do come because they say I want circumcision because they are facing a problem not necessarily that they want a circumcision. That’s the people when we treat them, they feel am healed, they don’t come back. Some they understand the message, they say that, I did really want circumcision maybe with what they have said, and I should opt for circumcision to avoid these STIs. To me, I really feel it’s good but also being that we are providing medical services here, I feel we cannot just look at a disease and let it go, I feel that it’s very good that we do treat here, them.*
59. I: okay. On your own opinion, what is it that you do not like the integration of STI with Voluntary Medical Male Circumcision services?
60. *R: I would say the workload part*
61. I: ehm
62. *R: yea because when the client is normal, its either I would take that client to the procedure, that’s what am really doing here, yea but when I have found a client with STI, am going to leave everything that am going to do to treat that person first, which is a bit heavy because we are not doing referrals, everyone is trained is whatever we are doing, yea but we know were the risk and the benefits so yea, the benefits, is much good than what am saying.*
63. I: What is it that you like of the integration STI services with Voluntary Medical Male Circumcision services?
64. *R: I think I have already tackled that one, I have said that we are treating there and then*
65. I: but there might be something you like which on your own you like on the integration part with the combination of STI.
66. *R: I think the identification part because some are shy, I can say that some do come here while they are already circumcised to say that I have an STI, which is not really what they have to do, they should be going to the STI clinic but here everyone will be just considered as someone who just need medical circumcision, so people are afraid to come to us than going out there to an STI clinic and we are able to identify those conditions than just doing circumcision. We could have been doing to some people which are infected or we could have been sending them back which is not good.*
67. I: So how do you think STI services should be offered at Voluntary Medical Male Circumcision Clinic?
68. *R: hmmm… how?*
69. I: yes, in terms of place, should it be within the same building, or outside, should be like a clinic or within the same building but in the private room?
70. *R: ehm, yea I can say STI need privacy, so I feel like it should be offered in a different room though here I should say we do not combine but it’s a room that we do screen everyone, after they Identify that’s when we take the client to another room but had it been that we had STI special room maybe some could have been knocking straight in that room. Though I know these things, people don’t just show up and say I have an STI, they wait for us to tell them, you have an STI, Yea, but it is better that way to have a special room for an STI service.*
71. I: okay outside or within?
72. *R: within because the outside part, people don’t feel comfortable to say*
73. I: okay. Aaah, so, what do you think are the barriers and concerns on this integration?
74. *R: Aaah, maybe on space part. We are better here maybe because it’s big but to some other facilities, it’s small. So, yea… the space become the challenge for you take an STI client to special room… yea, maybe you just close the door and take the client right there… yea, the space is the challenge.*
75. I: so what do you think should be done to address that challenge?
76. *R: I would have say infrastructure but it’s a big thing you know*
77. I: yes
78. *R: yea [laughing]*
79. I: [laughing]
80. *R: so for me to address that problem, we just need to consider STI thing, which we do already consider but for us to address that problem, it’s really very big for the infrastructure part. Another, we may opt them to take clients up there which the client won’t feel comfortable, there are better treated here*
81. I: okay
82. *R: yea*
83. I: Aaah, now let us talk about family planning. Aaah, explain to me what happens if a client needs a family planning methods?
84. *R: a male?*
85. I: a male or female, whether it’s a male who wants vasectomy or any other methods of family planning. Do you know any methods of family planning?
86. *R: yes*
87. I: can you tell me some
88. *R: you mean all or that we do provide here*
89. I: any you know and even those you do provide
90. *R: we have depo, I mean we do provide at VMM clinic*
91. I: you can start with general, and from general you can start saying…
92. *R: okay, we have depo, the injected. We have the pills, we have the implants, we have loop [ISD], we have condoms, we have BTL and vasectomy but most of facilities don’t provide that*
93. I: Which methods do you provide here from the list you stated?
94. *R: mainly its condoms*
95. I: condoms?
96. *R: ehm*
97. I: okay, so what happens if a client needs the family planning methods, what do you do?
98. *R: mainly here, people don’t ask for family planning methods, a lot of them. Sometime, we do persuade that please have condoms for this purpose. We explain to them but mainly people don’t ask for family planning methods here, maybe they go somewhere else to seek for family planning methods. But for condoms we do give them and we do advise them to use condoms*
99. I: okay
100. *R: yea*
101. I: Aaah, so as a health care provider, what is your opinion on integrating Family planning in circumcision services?
102. *R: okay, because most of the planning methods that I have mention are used by females, I think that’s the reason why am saying that these guys don’t ask for... ehm, for the family planning methods. The other thing is, most of messages that we, health providers or those that do share information do give, will always target females, when it comes to family planning. The other challenge could be, the information that we are given is also less on the male part.*
103. I: okay
104. *R: yea*
105. I: What is it that you would not like the integration of family planning in Voluntary Medical Male Circumcision Clinic? If we combine or integrate, what do you think you would not like?
106. *R: if we combine, the challenge could be the space part because we are going to have more clients because a lot of women will be coming to our clinics and that will need us to be providing the services and maybe divert our focus from medical male circumcision part unless there is also human resource added to the doing that or maybe increase the number so that we may be focus on that, otherwise, there is no problem on us in providing that service because we are already doing that in other areas*
107. I: Okay, so what is it that you like of the integration of Family planning with Voluntary Medical Male Circumcision services?
108. *R: …*
109. I: you as a health care provider
110. *R: ehm*
111. I: what is your opinion on that, what would you like on integration of family planning with voluntary medical male circumcision services?
112. *R: okay, integration help us to provide the services under one roof, so I would feel like we are targeting to males and females both, so it would lessen the work or even maybe promote the acceptance of males in doing the family planning because sometimes these women, they just do come to the clinic for family planning when their husband do not really want them to be taking family planning. So, it will be easier for the clients or even to us to be to have I mean like a huge target yea. So I think, it could really be helpful if we can combine that we should be looking on other factors that I have already mentioned but it is not problem for us to be providing them because that’s what we do.*
113. I: okay, how do you think family planning services can be offered within Voluntary Medical Male circumcision clinics?
114. *R: okay, maybe when we are counselling like doing the medical male counselling, we could also combine the component of family planning like the vasectomy part because I feel like people in Malawi don’t do much vasectomy, I have seen few in the hospital setting, but… sometime I feel like we don’t really give these men such information to do the vasectomy or the benefits, people will think like, I want vasectomy, I won’t be performing in bed. That’s what people think. But I have already said that most of family planning methods information targets women, if at all we have a better way of conveying messages to men, some would have been accepting the methods. So I feel like when we are doing counselling, we can include that component of family planning to the males, if they accept, we can also be doing it in our clinics… yea*
115. I: what do you think in addition, barriers and concerns in this integration
116. *R: to the integration part, the barrier may be, if we are only doing circumcision, we have a lot of clients, our work is already yea, that high, if we also maybe the client wants to do vasectomy it means we take more much time, yea and most of us are not trained in doing the vasectomy part. Even If we are going to give the information, maybe we are not going to provide it and its few that are trained, and those that are trained, cannot be with us during the circumcision part, maybe they are in other facilities doing other jobs whatever other thing. So integration might have that short fall, if they are going to give information, it means we should have people trained to do the vasectomy part and I think we can yield results. So that’s the short fall that we may have. We can give information but what if people accepts, we are going to book them maybe at Kamuzu central wherever that we think people will provide the services not here. So, the integration should come when we know that we have capacity to do it. Like the STI it was easy for me to say, almost everyone is trained to provide the service, every provider is trained, so different from the vasectomy part. For the other family planning methods that target women, most of us are trained because we provide more of that but if we are really to target men, we need also to talk of information that will need to target men and I feel that’s where the gap is.*
117. I: Let us now talk about cervical cancer screening
118. *R: ehm*
119. I: Explain to me what happens if a woman needs cervical cancer screening?
120. *R: I have never seen one here but in the hospital setting, I don’t know if that’s what you want*
121. I: no, you can go ahead, you can explain what happens to the hospital setting, you have never seen?
122. *R: here at the clinic?*
123. I: yes
124. *R: yes. When they come, we book then on a date because the resources, room etc., so people are trained, like providers. So we screen them, and like I have said of other things, we document result, the clients get the results were the patient needs referral, we do refer whether chemotherapy, surgery, whatever, we communicate the client and refer to the other facility if it’s necessary. When the woman is well, we give her the next day of appointment so that should come, maybe after some years for screening again.*
125. I: okay
126. *R: that’s what we do*
127. I: so, as a health care provider, what is your opinion on integrating cervical cancer screening in Voluntary Medical Male Circumcision services?
128. *R: integration is good, but like I have said in other points, it means we need more people trained there, we need a room, because it’s something that you can’t do in open space, you need privacy, you need a couch, a special one that will help you to look in the women cervix and all those resources but otherwise, if we can have that capacity, the integration don’t have a problem because it’s all about providing services under one roof, yea.*
129. I: okay, so in terms of a room, you said there should be a room, where should the room be exactly? If we can locate maybe within a clinic, where should it be within a clinic, outside or?
130. *R: that one maybe outside*
131. I: why outside?
132. *R: personally I feel like here we are targeting men, so for the women to be coming, making their line there, they will be feeling shy and also that service, it doesn’t take us a lot of time, that women should be falling online with men or yea, it’s something that can do within but a room it can be this is where we are providing a service yea.*
133. I: So, What is it that you would not like about cervical cancer screening integration with Voluntary Medical Male Circumcision services?
134. *R: of course some men don’t like to be combine with females, so especially older ones, so I think that can be a challenge somehow. Maybe it may affect the flow of clients to the clinic to some that are not strong, they may feel that aaah, women come too but to some it’s okay yea. The only other challenge maybe, if we start this, we need what I have said, trained people, space, human resource, resources and everything. The resources here are not very much, maybe the couch part and everything but yea, if we have everything, they don’t require much things but yea, we need people that are competent to say this is cancer, yea*
135. I: so in summary, what do you think are barriers and concerns in this integration?
136. *R: combing all these*
137. I: no like cervical cancer screening
138. *R: ooh on the cervical cancer part?*
139. I: yes, what do you think are barriers and concerns
140. *R: the barriers are the ones that I have already said that maybe it may affect the client flow and the other one on human resource and yea. And resources, resources that we may need and space that we may also need. When women receive message about cervical cancer they do come and women have their own problems, when they come to our clinics, we also suspect that this one will have cervical cancer, so everyone is scared of cancer and when we tell them that come for screening, they don’t hesitate, they do come. And you might have that flow that people yea. That can be that challenge*
141. I: so what do you think should be done to address those challenges?
142. *R: I think training more people, providing the information as well because in our VMM clinics we don’t tell, we don’t counsel men to be telling their wives to go, I should not say come because we are not doing that service, to go for VIA but most of times we give information to the female. The female are the ones that go and do the VIA thing, iii the circumcision thing but also be telling the men to tell their wife and can be really help for those women and if we can also have the space to have those resources, we can address that problem which I have said that we don’t have space, we don’t have those resources and human resources whatever thing, we can overcome such problems*
143. I: okay, now lets us discuss about PrEP. Have you heard about this before?
144. *R: yes*
145. I: what did you heard about PrEP?
146. *R: I have actually learnt*
147. I: How did you learn about PrEP?
148. *R: [laughing] through trainings, through ART trainings*
149. I: okay, so can you tell me anything about PrEP?
150. *R: Its pre-exposure prophylaxis , they provide ART to those that feels at risk of being infected, that’s 72 hours of time, that they have, yea been exposed*
151. I: Aaah, what you have just explained is PEP
152. *R: ooh okay*
153. I: but PrEP
154. *R: Oh sorry, PrEP is a new thing to me*
155. I: so, if you have not heard about PrEP, I will explain how the medicine works. PrEP is anti-HIV medicine that keeps HIV-negative people from being infected. For instance I can give you an example, there is a man who is negative but his wife is positive and he knows that my wife went for HIV testing and she is positive, am negative. They can still be having sex but while he is taking PrEP, a man without contracting a virus but there is a condition. So this PrEP, there is a single pill that is taken once daily, and if you take it regularly, it is highly effective at prevention people from being infected.
156. *R: okay, that’s a new thing to me*
157. I: now how do you feel about PrEP? If somebody ask you what PrEP is, what can you tell that person?
158. *R: not much because I really know how it works. You have just told me in brief but I don’t know how effective it is*
159. I: If PrEP was made available to HIV- men and women. Do you think you could advise your HIV negative clients to accept to take PrEP?
160. *R: yes, if at all I know the results, if I know everything yea, why allowing someone being of risk of contracting HIV when you know that there is a medication that prevent him*
161. I: so, what are the reasons you would encourage your clients to take PrEP?
162. *R: in order for him to stay strong and health for a long time*
163. I: okay. If PrEP becomes available, what is your opinion on integrating PrEP with Voluntary Medical Male circumcision services?
164. *R: I think if PrEP will be available it would help us a lot because if we found a negative or a positive person, we would have an interest to know the status of the other person so that we should be able to give PrEP to one of these clients, the one with different status*
165. I: okay. So if your client is negative would you encourage him to take it?
166. *R: if my client is negative, I would want to know the status of the other one because I would not encourage a negative client to be taking PrEP while the other one is also negative. But I should know that these partners have different status, that’s when I can provide, that’s why I said at first that it will need me to know the status of the other one, whether positive or negative, so that I should know when exactly to give PrEP.*
167. I: Okay, how do you think PrEP be offered in Voluntary Medical Male clinics?
168. *R: if I meet a client who already know that the wife is positive and he is negative, yea like we do with the other service, I think we can also do the education, counsel client and give him PrEP and tell him what to do next while on PrEP*
169. I: Where should it be, within the clinic, outside the clinic?
170. *R: I think within the clinic because as we do with other conditions, we do meet in treats, I think it’s the same with PrEP*
171. I: aaah, what do you think are the concern and barriers to integrating PrEP in Voluntary Medical Male services?
172. *R: I think in this case the barriers would be mainly on ascertaining the status of the other partner because mostly to those that are negative, because mostly, unless we are trained, we know better that’s when we can be focused to say even this one is negative, my wife can be positive but as of now, I cannot value the status of the other one, that’s where the gap may be but if I know better even if I found you negative I can be interested to also know, this one is negative but what about the partner*
173. I: so, what do you think should be done to address that barrier?
174. *R: I think this one, should be a training, we should know. If we know that will be easy for us to identify and we can be giving you data by now if we knew that there is this thing. I don’t know if my friends really knows but to me it’s a new thing. If we are really given this information we know, we can be much interested in the status of both the partners, so we can having the partners right away and treating them with PrEP yea with no hesitance*
175. I: let’s now go to our last part on other services. If you were given powers to choose and integrate services in Voluntary Medical Male Clinics, what are the services that you would think of Integrate?
176. *R: like from the list that you have asked me?*
177. I: yea from the list and if there is any other services you would like to integrate in Voluntary Medical Male Clinics?
178. *R: okay, we already doing STIs, I think even though I don’t know much, PrEP wouldn’t be that very much difficult, like how we treat the STIs because it’s a small, small thing… yea but for the others, they need a lot of time, a lot of resources for you to sit and say we are combining this into the VMM clinics. But for the other, maybe the vasectomy part, family planning I should say, if at all we would have the capacity, I think this would have been a window opportunity to be catching males because this is where males comes most if we may compare with services that we may come to the hospital for, I should say men.*
179. I: yea. So how do you think these services should be offered in the clinic?
180. *R: I think if we have trained people, it can be very easy for them to be providing circumcision and vasectomy at the same time because I have seen clients that do come for circumcision and they wants went for vasectomy and its q quiet hectic to say today I come for this, tomorrow but if we were also trained in such procedures it could have been easier for us and for the other services that I have already said. yea it’s a bit easier that those required skilled person to do*
181. I: Aaah, Thank you for taking your time to discuss with me today. Your answers will be very helpful in improving the health service delivery at Voluntary Medical Male circumcision clinics. Before we close, do you have anything to say?
182. *R: Ehm not much but may be when you were looking at those things that people mention, maybe has much emphasis capacity building part, we should be capable to provide those services and not just saying we are combining the services, maybe increasing the capacity that we have and also the resources part and everything that will need but otherwise the combination is not bad especially where the VMMC have a lot demand like here and other districts, we can have good outputs, we can yield results because mainly men will be coming and men are the ones that don’t really seek health…yea , so I can say the integration is good but the capacity should be linked into everything so that we can yield the result than advertising where we cannot do.*
183. I: okay
184. *R: yea*
185. I: Again, thank you so much for taking your time to speak with me
186. *R: welcome*
187. THE END

**D 43 STUDY**

**Date of Interview: 14 August 2018**

**Type of Participant: Service Provider**

**Interview Number: D-43-0026**

**Interviewer: I.N.**

**Total Interview Time: 32 minutes 18 seconds**

**Interview Summary:** **(from summary sheet)**

| **SERVICE TO BE INTERGRATED** | **THOUGHTS ON INTERGRATION** |
| --- | --- |
| Couple HIV Testing and Counseling | Thinks it is a good idea and it can save time for those who want to visit these services, as it can be cheap, if they come together. |
| STI Services | It works better |
| Family Planning | Thinks it is welcoming idea but if integrated vasectomy and VMC it might not work |
| Cervical Cancer Screening | Thinks integration is helpful as it will be like killing two birds with one stone |
| PrEP | Thinks it will increase the number of people coming for service |
| Other Services | None |

**Remarks:**

Participant was open and confident as he could explain more. All services to be integrated were appreciated as good.

**Interview Texts:**

1. I: Thank you for taking the time to talk with me today. I would like to ask you some questions today about the way you feel and what you think about some issues related to the service you provide and how we can include other services in Voluntary Medical Male Circumcision clinics. There are no right or wrong answers to these questions. We would like to hear your opinion and your experiences in your own words. Do you have any questions before we begin?
2. *R: I don’t have any questions, continue*
3. I: can you tell me how you are involved in the client care in this clinic
4. *R: Am the provider I provide the service of circumcision so it starts with examination of the patient that is if he is suitable for the procedure and giving of the advice on what should be followed and then seeing that the patient is going out without any complication and the procedure has been done and also the treatment has been given and the advice to the client*
5. I: Okay does your client talk to you about how the services are provided here?
6. *R: Mostly they do ask questions because they are not aware of what they are going through so they would rather try to gain information from us the provider and they start with on asking on the fear they have concern the vim and we give them information on that*
7. I: can you give me one example of what one client said to you, anything
8. *R: Feedback some have been given back mostly when you meet the wife at home and have a chart of the client you had they most appreciate that they are safe from cervical cancer and they are not experiencing the old problem like the accretion of the penis and is most of the hygienic that the appreciate mainly from the women side concern the husband that they have done circumcision*
9. How about the client himself does he say anything to you?
10. *R: They also say that at first they were firing that experienced more pain and cut the glands and they end up realizing that it’s a simple procedure.*
11. I: now let’s talk about partner HIV testing here at voluntary male medical circumcision clinic tell me what happens if a man brings a spouse here at voluntary male medical circumcision clinic
12. *R: we are always happy because we normally encourage that, if they are married men they should bring their wife, so that the women should get the advice so that they are able to take care of the wounds, when they come here they are treated as VIP because they have done right thing, in a rare occasion is when a woman can refuse, but mostly both of them go for testing. Because we are interested in them man, we allow the man to have the procedure first.*
13. I: so have you seen a man coming here with his spouse for HIV testing
14. *R: I haven’t seen one here, but where I used to be I have seen several come.*
15. I: what do you think are the motivators for men to being their spouse here for VMC?
16. *R: firstly, it shows that the couple they do the counsel thing together and it’s a family that they are closer to one another because it’s rare for family members to discuss issue that involves sexuality, so we will take it a step, that the family is free to talk about sex in their family and make a decision as a couple*
17. I; for those who do not bring their partners what do you think makes men not to bring their spouse for their circumcision
18. *R: most of me do not realize that women are part and parcel of the VMMC, so we need to give them that information that encourage them to bring their spouses*
19. I: what did you say needs to be done?
20. *R*: *We need to motivate them or and use public awareness messages on the importance of couple testing and counselling because it comes as a responsibility of the family, there was this guy who said the wife was pregnant went for testing then found negative. So she told the husband to come for testing as well, he had to call the wife and told her he is negative and I will get circumcised and they seemed happy, so most of them don’t have that information*
21. I: so you as a healthy care provider what is your opinion on integrating couple counselling with voluntary male medical circumcision services
22. *R: it is a good idea and it can save time for those who want to visit these services, it can be cheap, if they come together they get the advice and go and practice because everyone has heard for themselves. They can be able to ask questions, so it should be encouraged, it can be a good outcome*
23. I: what do you think are some of the barriers and concerns of this integration?
24. *R: the first is cultural, our culture feels like, women are sidelined in the family, secondly like men are not free to discus with women, but when only women the information about cervical cancer it’s when men are coming in to do circumcision because women can easily discuss about cervical cancer and I have seen men coming because of that, they say my wife was concerned with cervical cancer that’s why I have come, this is what is happening as a culture, men do not discuss and women do discus.*
25. I: what do you think can be done to overcome those concerns or barriers?
26. *R: it’s like more of encouraging women for instance here when a young boy comes to the clinic for circumcision aged 12-14, they ae normally brought by the women in the houses, so we need to emphasize on public awareness even when we are teaching about VMMC so that these women would also encourage their men to come for testing and circumcision. We need to intensify the messages going out to the people*
27. I: now I have to discuss with you and sexual productive health and pills for HIV prevention called pre exposer prophylaxis (PEP) sexual productive health include services that promote good sexual health and reproduction ,they include and not limited to family planning ,cervical cancer screening , sexual transmitted management and many more , today we will only discuss about family planning , diagnosis and management of STI, cervical cancer screening and prep, we will look at each one of these one by one , let us start with STI services , explain to me, what happens when a client is suspected or is diagnosed with an STI infection here
28. *R: normally it’s a policy on emphasizing much that one who is having an STI should go for counselling after having been tested, you rule out the condition of the patient, in case he is found to be negative then we go straight away to treat the patient with STI and you give them the message on prevention and how to take the pills, we also give them a receipt to come for treatment, if he is found positive the client is treated and is told to go collect ARVs immediately*
29. I: you as a health care provider, what is your opinion on integrating STI services with voluntary male medical circumcision services
30. *R: it works better, I have seen a number of men coming and getting VMMC but they end up getting treatment of STI and they encourage them to go home, finish the treatment and come back for VMMC, it works better that way and most of them tell their spouses to come and get treated, so it should be included as well*
31. I: what is it that you do not like with the integration of STI and voluntary male medical circumcision services?
32. *R: there is nothing, because one will treat the other, if you do VMC it will reduce the risk getting the STI, so the same thing when we treat someone with STI, then also make that person to be suitable for VMMC, so there should be complimenting one another.*
33. I: how do you think STI services should be offered at voluntary male medical circumcision clinic?
34. *R:it should be offered right away after screening is done, when you are screening someone, found they have a problem you cancel them and give them the treatment and you are sure thy will come back for the services*
35. I: what do you think are the barriers and concerns of STI services and voluntary male medical circumcision services?
36. *R: the concerns are that, some would rather just come for VMMC and for them to understand that they have an STI they don’t like to be screened for that, they would just like to come for VMMC, that is the worry, some end up going back without getting VMMC*
37. I: what do you think should be done there to address these concerns and barriers
    *R: it should start with mobilizing the clients and we should be clearing out these messages, saying if you go for VMMV there is a number of few issues you meet like be screening of STI and get the treatment and don’t go straight away for a procedure after you get the treatment and also to prevent their spouses at home and the use of condoms, these should be aired out right away.*
38. I: do you think these services of STI should be offered within the same building of the clinic or outside in a private room
39. *R:* *it should be given within the facility that VMMC is done to reduce the hours of somebody staying out or to reduce the distance because you cannot come to VMMC and move to another office, hence someone may be lazy to be moving from one office to the other, hence it should be under one room*
40. I: let us look at family planning, explain to me, what happens if the client’s needs a family planning method
41. *R: if it it’s a man, he is referred to a facility where they can get a vasectomy or e book for them for surgery and if it’s a female, we just refer them as well to a facility where they provide family planning services*
42. I: as a health care provider what is your opinion of integrating family planning in a male circumcision survives
43. *R:it can be a welcoming idea, even though it’s rare for men but if you integrate vasectomy and VMMC it might not work but we provide condoms which is the family planning method and for women , mainly to work better ,if it’s there already it can be used to serve females who have escorted their spouse to get VMMC so they can also be getting the family planning methods, it will also save the time spend at the hospital, since it will be here, maybe we can also win a lot of women to bring their spouse to the e clinic since they also will be getting the services*
44. I: what is it that you will not like in the integration of family planning and voluntary male medical circumcision services?
45. *R: I wouldn’t like it in the sense that women are just coming are just coming on their own to get family planning service only, that would not work better because VMMC would be now for family planning , you re much aware that most hospitals women are there now and them ,3 quarter are women, so it can be monopolize it to be for females since women respond better to the hospital than men, it should be integrated but is should work for the couple that comes for the service and we give them the information for family planning, if they are willing they can get family planning services as well*
46. I: how do think family planning should be offered in a voluntary male medical circumcision clinics
47. *R:in the sense that they come for council about VMMC and go for screening, where the woman also go, is where we put the drugs, upon finishing screening we give a change to the whole family to make a decision, but condoms are always there to give to clients to use after healing*
48. I: you mean they should be offered in the same building
49. *R: yes, it should be offered in the same building*
50. I: what do you think are other challenges with the integration of family planning and voluntary male medical circumcision services?
51. *R: it will need an extra expertise in terms on vasectomy, even human resource to increase the number of people available for the service, for those family planning method that are cheap, they can just be done within without the need for an expert, we can start with those that are cheap and simple to be done, while looking further to which other ways can we bring an expert of human resource for the expensive one.*
52. I: what do you think should be done to address those challenges?
53. R*: we should start with simple methods, like use of condoms and loop, we can also have orientations of one week or so*
54. I: let’s talk about cervical cancer screening, explain to me, what happens if a woman needs cervical cancer screening
55. *R: normally they would not ask because they know it’s for male, VMMC, in case they come, we can refer them to where they offer such services*
56. I: as a health care provider what is your opinion on integrating cancer screening and voluntary male medical circumcision services
57. *R:the right thing to be done , as I said easier on, women are pushing men to come for circumcision because of cervical cancer, we are taking something to make men to come for VMMC, so I feel if we go out with the message to say, men keeps the papiroma virus which starts cancer , if you can bring your wife, we can be able to screen them, hence we can have a huge number and give them the services right away, the other thing is nowadays , they just organize to go for cervical cancer, but the service that the women want now is to go for regular checkup since it has brought fear and they don’t have much knowledge about it, combining will be like killing two birds with one stone.*
58. I: what is it that you will not like with cervical cancer screening and voluntary male medical circumcision services?
59. *R: women respond better to the hospital than men, so we are still having problems to convince men to come for circumcision while it will take women seconds to be convicted, it can also scare men away, to say when you go there you will be seen by women after circumcision, hence there would be a need for privacy for those couples who do come, but I fear the dominance of women taking over.*
60. I: what do you think is the best way of offering these services together?
61. *R: maybe it should be offered to those who have come as a couple*
62. I: where exactly in the building
63. *R: it can be in the same ways we do the screening in the same room for both men and women so it can be convenient enough, we screen a man and see he doesn’t have the problem and check the woman if she doesn’t have the problem as well, because treating a man without treating a woman is like we have done nothing*
64. I: what do think are the barriers and concerns in this integration?
65. *R: the messages being given out that men are carriers of the virus that causes cervical cancer but not giving out the information of where they can get the screening for cervical cancer and management, so the concern is that the message you are giving should be right to the people, hence we need to give the information that is right, it’s like we are getting the demand but we do not have the response*
66. I: what do you think, these barriers can be addressed? Or be overcome
67. R: *change the mindset of people through social behavior change messages concerning cervical cancer and male circumcision that these are one and should be done concurrently*
68. I: now let’s talk about PREP, have you heard of it before
69. *R: yes, I have*
70. I: what have you heard about prep?
71. *R: these are vaccines or pills given to somebody to prevent them from getting HIV aids virus*
72. I: how did you learn about this?
73. *R: I got it from news but I did not get all the information of how it is done*
74. I: is it given to someone who’s status is already known or not?
75. *R: yes, to the one whose status is already known that she or he is negative, so when he or she have sex with someone with HIV positive they should not to get the virus*
76. I: how does it work how is it taken in terms of duration, for how long that person should be taking PrEP
77. *R: that one, am not much aware of how long and how often it should be taken*
78. I: do you think these pills are readily available
79. *R: yes, on the news they said Malawi is one of the countries to be given to*
80. I: if PrEP would be made available to negative men and women do you think you can advise your HIV negative clients to take PrEP
81. *R: yes, we should understand that the practice of having sex from different partners and if there you never know when you going to have sex with some who is positive, so its proper for someone to be given PrEP*
82. I: if PrEP becomes available, what is your opinion on integrating prep and voluntary male medical circumcision services?
83. *R: I would be worried on the message which will be given out to people, it can be a source of infections depending on the message that you are giving to people, for instance, in the past felt that if they have done circumcision they are not prone to get the HIV virus, so it took time for the message to be changed to the better, so that people should get the concept of VMC against HIV virus, the same thing should be applied when advocating for the drug, so the information should be good to give to the people and emphasize much on prevention than going out just because you have the drug that can prevent it*
84. I: so you would encourage your clients to take it
85. R: yes, I would
86. I: on what reasons would you do that?
87. *R: as already said, based on the life of someone, u never knew, when someone is found negative but looking at her or gigs lifestyle you can encourage them to take the drug*
88. I: so how do you think prep should be offered in a voluntary male medical circumcision clinic?
89. *R: Soon after finding that someone is negative and has gone through VMMC, they can be fit to be given to them*
90. I: so it should be given who has been found negative or anybody else
91. *R: mostly on the preventive part, but if you put that it should complement the VMMC it can be better, because we will be doing the right thing at the right time*
92. I: so what do you think are he concerns and barriers o integrating PrEP and voluntary male medical circumcision services?
93. *R: I don’t think there’s much worry, but we can expect a boom in the number of people coming for the services, so we need to have enough resources in order to meet the demand which is going to come and both for human resources and supply of prep and the providers of the procedure*
94. I: now let’s talk about the other part which is other services. If you were given a chance to choose in a voluntary male medical circumcision services, what are the series you would choose to integrate?
95. *R: STI, VMC, and family planning*
96. I: Explain to me the reasons why you have chosen these services
97. *R:I will start with STI is like the same, if someone is found positive and has an STI, has to get treatment to achieve the 90 90 campaign at the same time the spouse should come at get the treatment, while on family planning it will be like saving the same course, the woman will be safe by getting information on cervical cancer and STI, and she may get the cure right away, for the man himself, if the women doesn’t have it he need to get VMC to complement each other, so it will be convenient and easy and cheap in terms of time spend at the hospital moving from one door to the other*
98. I: How do you think these services should be offered in the clinics?
99. *R: so far they will need that those who goes to still clinic which is normally at pod level need to have all this knowledge and skills, even the drugs should be in that room so that these services can be offered right away there*
100. I: thank you for taking your time to talk with me today, your answers will help in improving health service delivery at a voluntary male medical circumcision clinic, before we close do you have anything to say
101. *R: No*
102. I: Thank you once again
103. THE END.

**D 43 STUDY**

**Date of Interview: 14 August 2018**

**Type of Participant: Provider**

**Interview Number: D-43-0027**

**Interviewer: I.N.**

**Total Interview Time: 30 minutes 42 seconds**

**Interview Summary:** **(from summary sheet)**

| **SERVICE TO BE INTERGRATED** | **THOUGHTS ON INTERGRATION** |
| --- | --- |
| Couple HIV Testing and Counseling | Thinks male medical circumcision services is an integration already and it’s just matter of adding something on it. |
| STI Services | Thinks it is good because it will be like killing two birds with one stone |
| Family Planning | Think it a good idea to integrate family planning in a male circumcision clinic to the men, though it’s going to be a big challenge, because many men are not involved in family planning |
| Cervical Cancer Screening | Thinks it’s going to be difficult rather, having men circumcised here and on the other side having cervical cancer screening , the process that takes place I don’t think it’s going to be easy, |
| PrEP | Think integration is very important because it’s difficult to convince people to be living together as a discontent couple and be using condoms, as its difficult foot there couples to be using condoms, so it’s better to use PrEP |
| Other Services | PrEP and family planning |

**Remarks: Participant was open, relaxed and a bit of shy.**

**Interview text**

1. I: Thank you for taking the time to talk with me today. I would like to ask you some questions today about the way you feel and what you think about some issues related to the service you provide and how we can include other services in Voluntary Medical Male Circumcision clinics. There are no right or wrong answers to these questions. We would like to hear your opinion and your experiences in your own words. Do you have any questions before we begin?
2. *R: hmmm… aaah, no*
3. I: can you tell me how you are involved in the client care in this clinic
4. *R: I think am involved in several ways, just to mention a few. am involved in screening of patience, when I say screaming of patients I mean, as you know we are doing voluntary male medical circumcision we have to screen them weitherr those who have come for circumscision are fit for circumcision, we do screen them for wither they have deformities, we do screen them whether they have sexual transmitted infections and in due course we do treat them whenever we find them to have those sexually transmitted infection*
5. I: does your client talk to you about how the services are provided here?
6. *R: yes, some group gave us feedback*
7. I: can you give us an example of a client who talked to you about the services he received here
8. *R: I should say I have received a number of feedbacks from client especially when they come for review, when their wounds are healing, the way they are progressing they do give us feedback.*
9. I: can you give me one example of what one client said to you, anything
10. *R: there was this one gentleman, who received male circumcision about a week ago, when I met him in the review room, then he was like thanking, he was like “thank you guys you have done a nice job, it was not even painfully, my wound is not even infected “*
11. I: now let’s talk about partner HIV testing here at voluntary male medical circumcision clinic tell me what happens if a man brings a spouse here at voluntary male medical circumcision clinic
12. *R: a male bringing a spouse at this clinic is very rare and I should say I have never met nay couple*
13. I: what do you think makes men not to bring their spouse for their circumcision?
14. *R:am not sure about the reason behind, maybe it’s about attitude, about how men regard circumcision, they take it as a taboo for when they want to go visit a male circumcision clinic*
15. I: so you haven’t seen a male a man coming with his wife or partner for HIV testing at voluntary male medical circumcision clinic
16. *R: yes*
17. I: for those who do not bring their partners, what do you think demotivates men to bring their partners for HIV testing and counselling
18. *R: as I have already said, I think is a matter of attitude, they think coming to a male voluntary circumcision is not for the woman, it’s not for the couple, the only women we see here are those who bring their little children for circumcision*
19. I: what do you think can be done to make men bring their partners here for couple testing and counselling
20. *. R: I think sensitization is very important, if we sensitize these people that it’s possible to can accompany your husband to a male circumcision clinic, there would a change to that attitude*
21. I: so you as a healthy care provider what is your opinion on integrating couple counselling with voluntary male medical circumcision services
22. *R: You know male medical circumcision services is an integration already it’s just matter of adding something on it, you know we do screening of STIs as I have already said and deformities, some clients were born with deformities, they did not go for surgery we recognize them and send them for surgery that is part of integration, so if we include this I don’t think it will be a problem and it’s something that Is possible*
23. I: what do you think are some of the barriers and concerns of this integration?
24. *R: of course I should say cultural beliefs, circumcision mainly deals with private parts, so culturally will be talking private parts publicly it becomes a problem of now because of the culture beliefs*
25. I: what do you think can be done to overcome those concerns or barriers?
26. *R; we have to sensitize those people*
27. I: now I have to discuss with you and sexual productive health and pills for HIV prevention called pre exposer prophylaxis (PEP) sexual productive health include services that promote good sexual health and reproduction ,they include and not limited to family planning ,cervical cancer screening , sexual transmitted management and many more , today we will only discuss about family planning , diagnosis and management of stis ,cervical cancer screening and PrEP, we will look at each one of these one by one , let us start with sti services , explain to me, what happens when a client is suspected or is diagnosed with an sti infection here
28. *R:let’s say someone has come here for circumcision and we have diagnosed him with an sti infection or disease, we council him, making him realize what kind of a disease he is having , how that dieses is contracted, how that disease can be spread to partners and we also sensitize him on ways to prevent contracting that disease , treatment and importance of adhere treatment and the importance of treating the sexual partners and telling that client on the complication that may result because of the infection*
29. I: do you still go ahead with the procedure or the process of circumcision or you send him back after giving him advice
30. *R: normally we treat, send him back, and review after one week if we are satisfied that all is ok we go through with the procedure*
31. I: you as a health care provider, what so your opinion on integrating sti services with voluntary male medical circumcision services
32. *R: I think it’s good*
33. I: why do you think it’s good?
34. *R: you know someone has come for circumcision, during the course of screening, he is diagnosed and treated, and it will be killing two birds with one stone*
35. I: what is it that you do not like on the integration of voluntary male medical circumcision services?
36. *R: as of now I haven’t seen anything bad about it*
37. I: how do you think sti services should be offered at voluntary male medical circumcision clinic?
38. *R: maybe we should just find a means of treating these couples, as partners, because we are talking of men coming here diagnosed with stis but where is the partner, she is at home, we tell the client to go and tell the wife to go to a clinic, at least if we were able to call them here and treat them*
39. I: in the process of circumcision where do you think these sites of stis services should be put?
40. *R: in the process of circumcision, I think during the time of screening*
41. I: but should it be within the same building at the same premise or outside in a private room
42. *R: it can be in the same room, if we are able to convince women to accompany their spouses, where there is that group cancelling, both men and women together in the group cancelling and after screening the woman will be waiting for the husband, after screening the man I s diagnosed with an sti, goes back to the wife, telling her, I have got his problem, we have been advised to get treatment, let’s go and get treatment*
43. I: what do you think are the barriers and concerns of sti services and voluntary male medical circumcision services?
44. *R: I will still go with the cultural beliefs; we are so much ashamed*
45. I: what do you think shoulder done there to address these concerns and barriers
46. *R: let’s go out there, reach the people, sensitize them, and understanding little by little*
47. I: let us look at family planning, explain to me, what happens if the client’s needs a family planning method
48. *R: the only family planning method that is being offered here is the use of condoms, but it’s indirect*
49. I: so you don’t offer any other family planning method apart from distribution of condoms
50. *R: yes*
51. I: as a health care provider what is your opinion of integrating family planning in a male circumcision services
52. *R: I think it a good idea to integrate family planning in a male circumcision clinic to the men, though I know it’s going to be a big challenge, because many men are not involved in family planning*
53. I: what do you think need to bed one there
54. R*: the best thing that could be done is to include women participation in voluntary male medical circumcision services, that is during the group counseling, education on family planning would be tackled as well*
55. I: what is it that you will not like in the integration of family planning and voluntary male medical circumcision services?
56. *R: I don’t think of anything*
57. I: how do think family planning should be offered in a voluntary male medical circumcision clinics
58. *R: Men are very reluctant to be involved in family planning services but there are methods that concerns men, so it’s a minor surgical procedure, if we sensitize this men, some are already willing hence we can also sensitize them about circumcision*
59. I: beside the challenge you mention earlier, what do you think are other challenges with the integration of family planning and voluntary male medical circumcision services
60. *R: maybe, space, space where these people can be accommodated and the services being offered, it can act as a barrier*
61. I: Any concern
62. *R: an only a provider*
63. I: Yes, as a provider what are your concerns if you intergret family planning and voluntary male medical circumcision services
64. *R: on my part as a provider maybe is expertise*
65. I: what don’t we take it generally? Let’s take it you are just a human being, from the way things are, maybe we combine family planning services and voluntary male medical circumcision services, what can be a worry?
66. *R: it can may result in a drop out of some men, as I have already said, men leave family planning services to men*
67. I: what do you think should be done to address those challenges?
68. *R: there is a lot to be done, let’s talk to these men, that family planning is not for women only, men should also take part in family planning services*
69. I: how do you think this can be done, in what ways?
70. *R: when these men are here for circumcision, during group cancelling, we have to include that topic of family planning, as we are teaching them about sexually transmitted diseases, lets also tackle family planning issues and the importance of integration, they have come here for circumcision at the same time, they save time, instead of coming here this week for circumcision the other week for family planning method*
71. I: let’s talk about cervical cancer screening, explain to me, what happens if a woman needs cervical cancer screening
72. *R: there is a woman in the village who wants service cancer screening, they do go to clinics where they provide those services*
73. I: what if a woman comes here, maybe found you, I need cervical cancer screening, what happens?
74. *R: we do refer them to where those services are provided*
75. I: as health care provider what is your opinion on integrating cancer screening and voluntary male medical circumcision services
76. *R: it’s going to be difficult rather, having men circumcised here and on the other side having cervical cancer screening, the process that takes place I don’t think it’s going to be easy,*
77. I: how do you think that can be solved or be best offered here at a circumcision clinic?
78. *R: I think two clinics can be run parallel to each other, circumcision clinic as well as cervical cancer screening, but not in the same building*
79. I: what do think are the barriers and concerns in this integration?
80. *R: privacy, what is involved in cervical cancer screening is where a woman is stripped naked, her legs pulled upwards, to many women it’s going to be very difficult for them to be screened where man are circumcised*
81. I: what do you think, these barriers can be addressed? Or be overcome
82. *R: providing these women at least with a room where they are going to be comfortable, separately from where men will be receiving their circumcision*
83. I: do you like the idea of integrating cervical cancer screening and voluntary male medical circumcision services
84. *R: the actual screening and voluntary male medical circumcision, I don’t think they should be interpreted, but these two go hand in hand, so awareness campaigns can be conducted that during circumcision processes, we can also be telling these women to go for cancer screening, otherwise the integration of cancer screening and male circumcision is going to be difficult*
85. I: now let’s talk about PrEP, have you heard of it before
86. *R: I think am hearing it for the first time*
87. I: if you have near heard about PrEP I will explain how the medicine works, PrEP is an anti Hiv medicine that keeps Hiv negative people from being infected, there is a single pill that s taken once dairy an if you take it regularly it is highly effective
88. *R: so what does PrEP mean?*
89. I: PrEP is an anti HIV medicine
90. *R: no as it is* PrEP*? Is it an abbreviation*?
91. I: yes, it’s an abbreviation for pre exposure prophylaxis, let me give you an example of how it works if a man is Hiv negative and the woman is Hiv positive and both know their statuses, thy can be taking this PrEP and be having unprotected sex and not get infected as long as they are following required prescriptions. It’s taken once dairy and regularly, it’s different but many people confuse it with pep where you don’t take it once but you take it diary. Now how do you feel about PrEP?
92. *R: it is very important*
93. I: if PrEP would be made available to negative men and women do you think you can advise your HIV negative clients to take PrEP
94. *R: yes, I would*
95. I: why would you tell them that?
96. *R: because I understand it’s difficult for you to convince them to be living together as a discordant couples and be using condoms, you know it’s difficult for the couples to be using condoms, so it’s better to use PrEP*
97. I: if becomes PrEP available, what is your opinion on integrating PrEP and voluntary male medical circumcision services?
98. *R: that has to be integrating, it is very important*
99. I: so you would encourage your clients to take it
100. *R: yes, it I very important*
101. I: to be specific what kind of clients, would it be everyone else or those who has been found positive or those who are negative
102. *R: those who have been found positive, at least convince the to bring their partners, get tested if we have that thing of discordance then we can give them the PrEP*
103. I: so how do you think PrEP should be offered in a voluntary male medical circumcision clinic?
104. *R: through HIV screening*
105. I: in the same building or outside
106. *R: the way we provide sexual transmitted management*
107. I: so what do you think are he concerns and barriers to integrating PrEP and Voluntary male medical circumcision services?
108. *R: many people cannot trust it*
109. I: what do think should be done to deal with the challenge
110. *R: it should be taken to the masses, they have to understand what it is and how people can use this PrEP effectively.*
111. I: now let’s talk about the other part which is other services. If you were given a chance to choose in a voluntary male medical circumcision services, what are the series you would choose to interpret?
112. *R: I think I would choose to integrate this thing of* PrEP *we have just discussed, family planning issues*
113. I: Explain to me the reasons why you have chosen these two services
114. *R: I have taken simple services that would not be difficult for people to understand*
115. I: How do you think these services should be offered in the clinics?
116. *R: they should be offered where, one has gone through screening, testing and counselling, let’s say someone is found positive and we encourage him to bring his partner for a test, if the partner is found negative we offer* PrEP *and group cancelling we can be telling them about family planning methods that are offered here I think that will make things easy.*
117. I: thank you for taking your time to talk with me today, your answers will help in improving health service delivery at a voluntary male medical circumcision clinic, before we close do you have anything to say
118. *R: NO*
119. I: Thank you once again
120. *R: You are welcome*
121. THE END

**D 43 STUDY**

**Date of Interview: 15 August 2018**

**Type of Participant: Provider**

**Interview Number: D-43-0028**

**Interviewer: I. N.**

**Total Interview Time: 31 minutes 16 seconds**

**Interview Summary:** **(from summary sheet)**

| **SERVICE TO BE INTERGRATED** | **THOUGHTS ON INTERGRATION** |
| --- | --- |
| Couple HIV Testing and Counseling | Thinks it’s good to integrate because it will help combat HIV |
| STI Services | Think it’s very good to proceed with the integration |
| Family Planning | Thinks it’s a very good, it may help clients to have their opinion on family planning |
| Cervical Cancer Screening | It may be a good idea, because all those we are combating or have a good outcome as it could take care of the clients |
| PrEP | assuming it is 100% effective from preventing people, would like the integration |
| Other Services | STI screening, PrEP and cervical cancer screening |

**Remarks:**

**Participant was relaxed, confident and open. There was some doubts on the effectiveness of PrEP.**

**Interview Text:**

1. I: Thank you for taking the time to talk with me today.
2. R: Okay, thank you
3. I: I would like to ask you some questions today about the way you feel and what you think about some issues related to the service you provide and how we can include other services in Voluntary Medical Male Circumcision clinics. There are no right or wrong answers to these questions. We would like to hear your opinion and your experiences in your own words. Do you have any questions before we begin?
4. R: aaah, no
5. I: can you tell me how you are involved in the client care in this clinic
6. *6. R:first of all when the client is registered we go to screening , where we ask most of the questions related to male circumcision and sexually transmitted diseases which sometimes can affect the outcome of the make circumcision so make sure that everything we do should come clean and avoid the infection of the would after circumcision*
7. I: does your client talk to you about how the services are provided here?
8. R*: yes, some they do some they don’t, because some of the information dispersed to the guardians they just hear from so they might miss or not be familiar with it*
9. . I: can you give me one example of what one client said to you, anything
10. *R: at a certain time, one particular client came, he was complaining , he was delayed, we did not help him at a previous time that he came, but ere were ding other services of reviewing other patients while he was waiting ,so he was just complaining about that.*
11. I: now let’s talk about partner HIV testing here at voluntary male medical circumcision clinic tell me what happens if a man brings a spouse here at voluntary male medical circumcision clinic
12. R*: it depends with the councilor of the service provider, because this also one of the way of preventing HIV, so if one brings here we welcome them, mostly those people who are HIV positive are included because we test them and give them the treatment*
13. I: have you ever seen a man coming with his partner
14. *R: here no, we have not seen anyone, but those we are found with sits ae advised to come with their partners so some have come with them*
15. I; What do you think are the motivators that makes the men not to bring their spouse for their circumcision
16. *R: its fast, privacy and most of the time we maintain their confidentiality, that why they chose to come here*
17. I: for those who do not bring their partners, what do you think demotivates men to bring their partners for HIV testing and counselling
18. *R:it’s because , the information we give to the client is about male circumcision , so we have a lot of males here so maybe most of them are shy to bring their partners*
19. I: what do you think can be done to make men bring their partners here for couple testing and counselling
20. *R: it’s the matter of giving correct information and encouraging to come for testing since its one way of HIV prevention*
21. I: so you as a healthy care provider what is your opinion on interesting couple counselling with voluntary male medical circumcision services
22. *R: it is good, the couple can come and test because our aim is to reduce the infection so if the male client comes and is tested positive we initiate a council on treatment of aids to reduce the viral load so the integration can help to combat HIV*
23. I: what do you think are some of the barriers and concerns of this integration
24. *R:the most concerns and barriers would be site, where we going to do the test because there will be a lot of children and other guardians who will escort these kids or clients , so some of the clients may opt be happy to see these people to be around when they are being tested.*
25. I: what do you think can be done to overcome those concerns or barriers?
26. *R: I think we can create a private room away from the guardians and the clients that are young so that the couple can be cancelled*
27. I: so the room should be outside or inside?
28. *R: it can be either outside or inside but I prefer outside because it can a private room where people cannot be seen*
29. I: now I have to discuss with you and sexual productive health and pills for HIV prevention called pre exposer prophylaxis (PrEP) sexual productive health include services that promote good sexual health and reproduction ,they include and not limited to family planning ,cervical cancer screening , sexual transmitted management and many more , today we will only discuss about family planning , diagnosis and management of stis ,cervical cancer screening and prep, we will look at each one of these one by one , let us start with sit services , explain to me, what happens when a client is suspected or is diagnosed with an STI infection here
30. *R: first of all we ask the client if he has a prior acquaintance so that we can confirm that this is a sti, if we find that it’s a sti w give counselling and we don’t proceed we the procedure we take the patient to another room for counselling and treatment*
31. I: you as a health care provider, what is your opinion on integrating STI services with voluntary male medical circumcision services
32. *R: recently we have discovered that most clients come here to get the STI treatment through male circumcision, it has been hang because people are treated fast, they have the privacy and they are told to come with their spouse if they are found with the STI*
33. I: What is it that you don’t like in the integration of voluntary male medical circumcision services and STIs
34. *R: I don’t think there is anything that I don’t lie, I think it’s very good to proceed with the integration*
35. I: so you like the integration
36. *R: yes*
37. I: so what is it that you do not like with the integration of STIs and voluntary male medical circumcision services?
38. *R: clients are treated very fast and people come with confidence with their spouse, we treat them with no problems at all*
39. I: how do you think sti services should be offered at voluntary male medical circumcision clinic
40. *R: it should be offered to everyone who has STIs*
41. I: in the process of circumcision where do you think these sites of stis services should be put?
42. *R: the same way we are doing, when we are doing screening, we test for stis, then we take them to another room where we give treatment, people may not notice what we are doing because they will still be thinking that we are doing the usual VMC counseling.*
43. I: what do you think are the barriers and concerns of sti services and voluntary male medical circumcision services?
44. *R: because people come here for circumcision and you tell them there is sti, some are not happy, but after we council them, after a week or o they come for vmc counselling*
45. I: what do you think should be done there to address these concerns and barriers
46. R: I think the concern that people have is the period that we give them and we tell them to bring their spouse most of them don’t want to bring their spouse so we them to go to a separate hospital and get treatment
47. I: what if we bring a separate room, like outside, how do you see it?
48. *R:I don’t think that would be okie, the way we are doing we just combine the services because people will not discriminate them that they are having the stis, they will just see them as getting the same services that we provide as vmc , having a separate room, that will not help*
49. I: let us look at family planning, explain to me, what happens if the client’s needs a family planning method
50. *R: usually we refer them to a government or a private hospital to receive family planning, but we distribute condoms to clients that come here*
51. I: as a health care provider what is your opinion of integrating family planning in a male circumcision services
52. *R: yes it’s very good, it may help clients to have their opinion on family planning*
53. I: what is it that you will not like in the integration of family planning and voluntary male medical circumcision services?
54. *R:it it will depend with the male circumcision we are going to offer, but usually offer condoms to male , but having other integration cannot really help us*
55. I: how do think family planning should be offered in a voluntary male medical circumcision clinics
56. *R:I wa thinking maybe if during the post walk and counselling, we should offer the services and especially to those above 14 years so that they can have an option even if we are just giving out condoms , since we just condoms it can be a good idea to start off there*
57. I: what if it involves vasectomy for men and family planning for their female partners to be integrated here
58. *R:I don’t think that would be good, because to have vasectomy needs someone to be mature, and having family planning for females I don’t think some male would be coming to have this family planning, so I think is just okie to stick with one thing , but to combine it can work*
59. I: so in terms of that, if this kind of family planning involves vasectomy for male and other female’s family planning method, how can it work?
60. *R: If that can happen that there is an integration of the two at once, the possibility is that we can have two rooms that the men should go to the other room and the females the other rooms, because if we have one room some will be shy and not come to the clinic*
61. I: the rooms should they be in the same building or outside in a separate building
62. *R: we can have the same building, no problem*
63. I: what do you think are other challenges with the integration of family planning and voluntary male medical circumcision services?
64. *R: the barriers would be many clients more especially men, the possibility would be most men would Shum from the clinic, because they wouldn’t want to be seen coming out of a family planning clinic*
65. I: what do you think should be done to address those challenges?
66. *R: to do an intensive counselling and give out information and maintain privacy even if I tis the same room, but the privacy should be there to keep the clients comfortable*
67. I: let’s talk about cervical cancer screening, explain to me, what happens if a woman needs cervical cancer screening
68. *R: usually we refer them to a district hospital or health center, because we don’t offer them here*
69. I: as health care provider what is your opinion on integrating cancer screening and voluntary male medical circumcision services
70. *R: it may be a good idea, because all those we are combating or have a good outcome it could take care of the clients, but this process of cancer screening and VMMC may differ, so I don’t think it may work if we combine these services unless if we have these sites outside, the other room outside and the other room inside, so that the services are separated*
71. I: What is it that you will not like about cervical cancer screening integrating with voluntary male medical circumcision services?
72. *R: cancer screening and vmc, the other one it is mostly targeting those with Hiv testing, while the other one is just cancer, these two may work in palarel, to combine these services, I don’t think it may work*
73. I: What do you think is the best way to offer cancer screening and voluntary male medical circumcision services?
74. *R: I think have separate room because cervical cancer nowadays is very overwhelming, so we have to deal with them in the other room than the initial room*
75. I: do you like the idea of the integration
76. *R: yes I do*
77. I: what do think are the barriers and concerns in this integration?
78. *R: men will shun away from female clients to be on the same line to go for vmc or for council I don’t think many men would come*
79. I: what do you think, these barriers can be addressed? Or be overcome
80. *R: have a separate room, side and outside, and initiate counseling to the clients so that they are aware of what is happening*
81. I: now let’s talk about PREP, have you heard of it before
82. *R: yes*
83. I: if you have heard about prep, please tell me what you know about prep.
84. *R: this is a pre exposer prophylaxis, it is a treatment given to a client who has been exposed to HIV, or there is a risk of having HIV within 72 hours*
85. I: How did you learn about this
86. *R: in school, workshops, trainings*
87. I:oky, that is prep and not prep, so if you have not heard about prep I will explain how it works ,prep is an anti Hiv medicine that keeps Hiv negative people from being infected , there is a single pill that is taken once dairy an if you take it regularly it is highly effective at preventing people from being infected .the difference with pep is that prep is taken whale you know the status of your sexual partner, so you know that your wife is positive and you want to be having unprotected sex with her, you take prep to nor get the virus It’s taken once dairy and regularly , pep where you take it once when you are exposed. Now how do you feel about PREP?
88. *R: to me, I don’t think it’s good*
89. *I: you saying, they should the drug while you know the status of your partner who has the virus and you do not have the virus, but you are not 100% sure of how the drugs work, some may have the side effects, some would miss the dosage*
90. I: if prep would be made available to negative men and women do you think you can advise your HIV negative clients to take pre
91. *R: to me, no*
92. I: why would you tell them that
93. *R: of Couse you know we might prevent it, but we are not 100% sure that it will work, even the 0.something percent can impact on someone and get HIV*
94. I: assuming it is 100% effective from preventing people from being infected if you take it regularly, so what if is really effective
95. *R: then that is very good*
96. I: so being 100% sure, would you encourage your clients to take PrEP?
97. *R: in this case yes, I would encourage them*
98. I: what are the reasons?
99. *R: they will not get HIV, they will be safe they can have any spouse they want without any problem*
100. I: if prep becomes available, what is your opinion on integrating prep and voluntary male medical circumcision services?
101. *R: it would be good because VMMC is 60% and this one 100%, then we will achieve the 90 campaign I this country*
102. I: so you would encourage your clients to take it
103. *R: yes*.
104. I: so how do you think prep should be offered in a voluntary male medical circumcision clinic?
105. *R: I think soon after the procedure in post stop where we give panado, and we can give out the drug*
106. I: so what do you think are he concerns and barriers o integrating prep and voluntary male medical circumcision services?
107. *R: since this is new, we will have so many questions and worries, they are not sure what will happen if thy take it, so it’s a matter of ensuring them*
108. I: what do think should be done to deal with the challenge
109. *R:I think, the first thing is to teach them , to know what exactly how this drug works and how to achieve taking this drug regularly, giving them much information and counseling, they can have a good reason to take the drug*
110. I: now let’s talk about the other part which is other services. If you were given a chance to choose in a voluntary male medical circumcision services, what are the series you would choose to interpret?
111. R: the first one would be the STI. The second one would be prep and the third one should be on cervical cancer screening
112. I: Explain to me the reasons why you have chosen these services
113. *R: STI and VMC work hand in hand, so we are going opt achieve a god outcome, if they come for VMC screening and STI, we screen and treat them, for prep, since we are dealing with vmc, our aim is to reduce the infection rate, so prep will have an addition effect, in preventing HIV. On vmc and cancer screening, this may work hand in hand because cervical cancer is killing most of the women, so those people who are HIV infection may have cancer screening, hence we are reducing the infection of cervical cancer by screening men who contribute in making woman have cervical cancer*
114. I: How do you think these services should be offered in the clinics?
115. *R: STI and VMC should be serviced in the same room, prep and vmc can be in the same room, cervical cancer and vmc should be in separate room*
116. I: thank you for taking your time to talk with me today, your answers will help in improving health service delivery at a voluntary male medical circumcision clinic, before we close do you have anything to say
117. I: Thank you once again
118. THE END

**D 43 STUDY**

**Date of Interview: 15 August 2018**

**Type of Participant: Service Provider**

**Interview Number: D-43-0029**

**Interviewer: I.N.**

**Total Interview Time: 52 minutes 02 seconds**

**Interview Summary :( from summary sheet)**

| **SERVICE TO BE INTERGRATED** | **THOUGHTS ON INTERGRATION** |
| --- | --- |
| Couple HIV Testing and Counseling | Thinks its good development because the two services are like twins and created for the same purpose. |
| STI Services | Supports the idea since they both aim at one thing |
| Family Planning | Doesn’t support the idea, since these services are not related hence it’s good to only focus on related services |
| Cervical Cancer Screening | Dislikes the development, feels there is no link to VMMC |
| PrEP | Like the idea of integrating PrEP with VMMC services since they also provide condoms which does the same work |
| Other Services | Thinks STI services should be integrated into VMMC as they fuse well |

**Remarks:** The participant was a bit shy but open and explain as much as possible.

**Interview Texts:**

1. I: Thank you for taking the time to talk with me today. I would like to ask you some questions today about the way you feel and what you think about some issues related to the service you provide and how we can include other services in Voluntary Medical Male Circumcision clinics. There are no right or wrong answers to these questions. We would like to hear your opinion and your experiences in your own words. Do you have any questions before we begin?
2. *R: no lets proceeded sir*
3. I: Can you tell me how you are involved in the client care at this clinic?
4. *R: yeah, am a provider, that’s the main duty, apart from being a provider, I also do some pre and post counseling. Pre counseling that’s before the service, that’s voluntary male medical circumcision and post that’s after the client has received the service, voluntary male medical circumcision.*
5. I: Does your clients talk to you about how the services are provided here?
6. *R: yes, most of the times they do, if they were satisfied even if they were not satisfied they talk about it*
7. I: Can you give me an example of a time that your client talked to you about the services he received here?
8. *R: yeah, they are so men times, but most of them they do come to say like thank you. That what I was expecting from you is what I received, like appreciating, a few come saying that they are facing some little complications, as you know surgery may come along with other issues. So it is taking time to heal, yeah like its swollen, feeling pain, some of the complaints. but mostly they do come and appreciate for the services*
9. I: Now let us talk about partner HIV testing here at the Voluntary Medical Male circumcision clinic. Tell me what happens if a man brings a spouse here at the Voluntary Medical Male circumcision clinic
10. *R: though I haven’t met so many clients bringing their spouse, most come alone, but some do come together, just a few. But when they come, it’s like as usual a guardian. Only the man who is taken care of, undergoes the process we do, testing, the counseling, partially with counseling maybe they can be together the wife, as how to take care the wound but when it comes to testing only the man is involved*
11. I: So you said some do come with their spouses, what do you think are the motivators that make the men bring their spouses here for testing
12. *R: maybe looking at the issue of HIV, am just thinking, they may like killing two birds with one stone when they come together maybe, that what I think motivates them to come together*
13. I: For those who do not bring their partners what do you think demotivates men to bring their partners here for HIV counseling and testing
14. *R: I still feel that HIV still stands like, when one is tested and found positive, the issue of stigma and the likes, and still even married people some do not wish their statutes to be known to even their partners maybe that can be one factor that demotivates others to bring their partners when they come for the service,*
15. I: What do you think can be done to make men bring their partners here for couple testing and counseling?
16. *R: I feel first thing looking at the majority of people we deal with, if something can be put in, in terms like, maybe a certain fee, I mean an, like allowance that like appraising somebody those who brought their spouse, can be a transport to bring them back home, or receive something maybe that can encourage some, I feel so to my part*
17. I: You as a health care provider, what is your opinion on integrating couple counseling with Voluntary Medical Male circumcision services?
18. *R: aaah looking at voluntary male medical circumcision services and couple testing, as one or twins, two things are together because we cannot leave the other aside, as the whole purpose is to minimize the HIV infection, so taking these two going together can also be another way of promoting that prevention of HIV, as they do with pregnant mothers I have heard that they encourage also men to accompany them during pregnant. Same can also be apply in these services, and collectively at least we can have more people knowing their status, even of each other the transport will be there at the any maybe that the issue of preventing new infections can be achieving. That is if they are tested positive and even faithful to one another and to couple this can also be enforce as they will know their status at once from point zero, rather than one should know his and her status then after the long run the other one should also know their status. When it is done together they will rather start the journey together if found positive, if found negative they would be counseled accordingly, so continue with their marriage life*
19. I: What do you think are the barriers and concerns on this integration?
20. *R: they maybe, one, men have different responsibility from women, some are very busy they work up and start thinking of searching money, women also some busy searching money, so with that to make ends meet or to meet their daily needs maybe a challenge that the two whole parent to come for only this they feel that they will lose out. Maybe one should remain to search food for the kids at home, the other one to go for that, then just leaving everything. I was saying when you asked what could be done to motivate men maybe something to say if we leave all our things to be there and be on the queue, while on the queue we go on for testing or whatever, maybe we will at least gain something for the time we spend there. But minus that aaah I feel still more one problem”*
21. I: So what do you think can be done to overcome the concerns and barriers that you have just stated?
22. *R: yeah sure, I feel aaah.., maybe these people can be considered in a unique way, unique way such that when they come they should not queue or we should minimize their waiting time before service. How? that will be I hope the one running the program will know how they can do that, or what measures will they take in order to minimize the time of these when they come to access the service and any other motivating factors can be put in. on my side the ones I already mention, one transportation, two they should not queue for a long time waiting for the service they can be seen just within the first30 minutes they are they, they should see that they are helped, even starting time of work it should not be later than 7:30 and not whatever*
23. I: Now I would like to discuss with you about sexual reproductive health services and Pills for HIV prevention: called pre-exposure prophylaxis (PrEP). Sexual reproductive health includes services that promote good sexual health and reproduction. They include but not limited to family planning, cervical cancer screening sexual transmitted infection (STI) management, and many more. Today we will only discuss about family planning, diagnosis and management of STIs, Cervical cancer screening, and PrEP. We will look at each of these one by one. Let us start with STI services. Explain to me what happens if a client is suspected or diagnosed with an STI here?
24. *R: yeah, when a client comes, assessed, get his assessment and found or suspected to have an STI, he is referred to another room where further management is done thus having confirmed the diagnosed of STI, will be treated accordingly, so what happens then he is given another appointment just in two weeks and thus after finishing the complete course of STI confirmed* I: You as a health care provider what is your opinion on integrating STI services with Voluntary Medical Male circumcision services.
25. *R: I do support that this should not be detached this should move together as the whole purpose is just to prevent STIs including HIV, so having gonorrhea, or genital diseases that will promote even the transition of HIV so integrating them will combat the spread of both HIV infection as well as this behavior that may lead somebody to contract STIs*
26. I: What is it that you do not like the integration of STI with Voluntary Medical Male Circumcision services?
27. *R: it’s like you add more work, and sometimes because of that, privacy at one point may not be okay, because as per usual if its usual that people may see that from that consultation room one goes to this one then if he is okay then to the circumcision room, so when its seen that they have not passed through that others might suspect like what’s going on with this one. Eventually maybe one can feel like at that door one is treated with this disease, unfortunate thing also we do not circumcise them immediately we circumcise then after STI treatment, so may delay others, that is bringing in more workload*
28. I: To be specific what is it that you like of the integration STI services with Voluntary Medical Male Circumcision services***?***
29. *R****:*** *yeah, I do support much because integrating these two, is helping us to fully prevent STIs, not only targeting HIV but these other STIs like genital diseases which may promote even the spread of f HIV and hen addressing this part and the other part I feel like we are almost 90% combating the spread*
30. I: So how do you think STI services should be offered at the Voluntary Medical Male circumcision clinic?
31. *R: yes, to avoid missing them because when we refer them somewhere we may not know because we may not accompany them walking with them to that place, we may say go to Bwaila or the other hospital so we may miss that one and the spread may proceed .so treating them right away will ensure us that we minimize the spread of the infection*
32. I: So in terms of place at the VMMC where do you think these STI services should be offered?
33. *R: it should be offered under the same roof, however maybe it should also look into other staff, allocation should not be by chance but we should expect them that we will meet them, so because we have expected them we will properly organizing on how we can handle them so this will minimize the risk of delays and interrupting other services*
34. I: What do you think are the barriers and concerns on this integration
35. *R: yah barriers and concern you know some may have STI and feel it’s okay because they have stayed for long but if they are seeking VMMC I feel like if they are sent back they have some of the services like you know they will discourage others may be, few may feel important if they Have received the other service, some are maybe resources.*
36. I: What do you think should be done to these concerns and barriers? Address
37. *R: I think proper planning between these two they have to be planned the same the other one should not be underrated yah and implemented they may be such barriers and people may not feel neglected*
38. I: Now let’s go to family planning. Explain to me what happens if a client needs a family planning methods (vasectomy for men and family planning for female partners?
39. *R: at the moment they are referred to the other clinic where they get these services*
40. I: As a health care provider, what is your opinion on integrating Family planning in circumcision services
41. *R: Of course I dint see a direct link on family planning and VMMC though that is the same client we are dealing with it may bring benefit to the client if those services are offered at the same place and time though to me I don’t think there is direct like however family planning is important because it will minimize the traveling*
42. I: So you like the idea?
43. *R: No not very brilliant it’s just that we will minimize the time*
44. I: What is it that you would not like the integration of family planning in Voluntary Medical Male Circumcision Clinic?
45. *R: I see family planning as not directly like aaah helping in this issue of the main purpose of VMMC and STI management and also looking at incorporating this program like too many may at the end may not at the end may found that all these programme are not done well so sometimes it’s good just to focus on one thing I feel it’s good to focus on one area that’s what I feel*
46. I: How do you think family planning services can be offered within Voluntary Medical Male circumcision clinics?
47. *R I feel because the same client may need that and aaah this service is centered like the battle field is like we are targeting one area as far human reproductive system is* *concerned it’s like the working ground is the same it’s like we are not missing the target I feel they are in the same family*
48. I: Where should it be offered?
49. *R: It can be an attached department to these two aaah coz we mainly yah men mat bring their wives as they will be searching VMMC services so the man may be found with STI so they will be treated together. They can be in the same building but different compartment*
50. I: What do you think are the barriers and concerns with this integration?
51. *R: concerns aaah and barriers may be what I may suggesting at the first place we may not have the two as at the end we may be receiving only a man for VMMC of his goes and may be having the building to accommodate all those it will be challenge because we are already having problems to incorporate all HIV activities at art clinic like we may need more money and have a building to accommodate that and privacy and confidentiality that will be the challenges*
52. I: What do you think should be done to address these concerns and barriers?
53. *R: I feel they should be quick is still feel proper planning and timing can contract those challenges am foreseeing and if they sit properly and plan and look at the resources in terms of human and a material planning can help us*
54. I: so let us look at cervical cancer screening. Explain to me what happens if a woman needs cervical cancer screening?
55. *R: normally we refer that woman where that service is offered*
56. I: As a health care provider, what is your opinion on integrating cervical cancer screening in Voluntary Medical Male Circumcision services?
57. *R: yes, that a welcome idea and we are looking at a man bringing a wife and a wife looking for family planning and via however I have seen some hospital offering those services and they are doing it well the challenge is they offer it at different buildings far away here at Bwaila that will be a welcome idea but it will need proper planning and human resources*
58. I: What is it that you would not like about cervical cancer screening integration with Voluntary Medical Male Circumcision services?
59. *R: it’s like we are loading ourselves with different services at one point and normally for us to achieve and for these to be properly done they may need a biggest establishment it will depend this facility should grow to another level to be able to offer that services as the recommendation not just that we wanted to and not sustaining them it all goes with timely planning.*
60. I: How do you think is the best way to offer cancer screening within Voluntary Medical Male circumcision clinics?
61. *R: the best way is to make the environment conducive they have to feel to go and visit the clinic the environment should be attractive that will make women to accompany their husband the environment has to be attractive like the reception and how the services are being offered so the client will call more so we have to make ourselves better first before we start and can be done*
62. I: So in terms of place where should it be put?
63. *R: It should not be attached to VMMC it can have separate but not very far so boys should not the women we have to achieve confidentiality.*
64. I: What do you think are the barriers and concerns on this integration?
65. *R: Yah it’s like space we may think we have big vision but you find that there is no space for that like we might not have space for another wing aaah even the resources themselves aaah it may require to run to and from to find them since they may be limited if the government takes over it can be sustainable but still space and financial may be the challenge*
66. I: So what do you think should be done to address these concerns and barriers?
67. *R: yah I still feel with money like money can do things that we think it’s impossible like I was saying of space aaah some people can build in air like people can build upstairs 2 human resource the motivated one they come and ensure sustainability of that*
68. I: Now let us discuss about PrEP. Have you heard about this before?
69. *R: yes*
70. I: please tell me what you know about PrEP?
71. *R: like they just take the pills like I will not take condoms so just use pills to protect myself I healed like these are like the barrier like the virus will already with the guns like the virus cannot penetrate its iron gate and a thief cannot enter*
72. I: What’s the difference between PrEP and PEP?
73. *R: PrEP the terminology it’s before exposure and after exposure it’s you have already protected yourself before the enemy and pep it’s like the enemy has given you the punches and you are just protecting the severity of the punches*
74. I: In terms of duration how long do you take it?
75. *R: only one month*
76. I: According to this PrEP is anti-HIV medicine that keeps HIV-negative people from being infected. There is a single pill that is taken once daily, and if you take it regularly, it is highly effective at prevention people from being infected. Now how do you feel about PrEP?
77. *R: PrEP is good than pep good in the sense that they may not be such issue as delay it’s like the pep is giving the chances that you may delay to take the drugs so with prep I feel like issues of delays are not there of course you have taken the pill and you are ready to face the challenge and the protection is already in you*
78. I: If PrEP was made available to HIV negative men and women. Do you think you could advise your HIV negative clients to accept to take PrEP?
79. *R: yes*
80. I: What are the reasons you would encourage your clients to take PrEP?
81. *R: not just taking them without any exposure or unexpected exposure and mot exposure are anticipated you know where am going am going to do this and may take condom and safety may not be 100% and advice HIV negative client to have prep*
82. I: If PrEP becomes available, what is your opinion on integrating PrEP with Voluntary Medical Male circumcision services? Would you encourage clients to take it?
83. *R: after we give them condoms after everything is okay and even though others are married as you know life differs we still give them prep so I will encourage my client to take it.*
84. I: How do you think PrEP be offered in Voluntary Medical Male clinics?
85. *R: what we are dealing here we are aiming at a free nation and VMMC at its own its playing a part and condoms counseling and sometimes all these may fail and prep it’s like we are finishing it that this negative client may be protected*
86. I: What time?
87. *R: after check like when the wound is healing like give them condoms however 6 weeks is very far so second review will do us good we may advise them like apart from condoms PrEP is also available*
88. I: In terms of place?
89. *R: It has to be in the same room like in the same room with condoms*
90. I: What do you think are the concern and barriers to integrating PrEP in Voluntary Medical Male services?
91. *R: may be the one like skills knowledge like its difficulty to find one person to be integrated in these all skill and service s so the skill may be the challenge*
92. I: What do you think should be done to address these concerns and barrier?
93. *R: I still having enough resources that can enable us to have structures to accommodate these service and enough skills*
94. I: Let us look on other services. If you were given powers to choose and integrate services in Voluntary Medical Male Clinics, what are the services that you would think of Integrate?
95. *R: Aaah VMMC STI & VIA can make good combination then followed by family planning,*
96. I: Reason for your choices
97. *R: much as the services offered are targeting one system both male and female reproductive system so integrating them we are not missing the point*
98. I: How do you think these services should be offered in the clinic?
99. *R: they should be some demarcation and aaah we will be seeing different ages and should be treated accordingly in VMMC they are both adults and young 9ones and in via we only see women and family planning it’s both men and women*
100. I: Thank you for taking your time to discuss with me today. Your answers will be very helpful in improving the health service delivery at Voluntary Medical Male circumcision clinics. Before we close, do you have anything to say?
101. *R: No just thanking you*
102. I: Again thank you for taking time to speak with me.
103. THE END.

**D 43 STUDY**

**Date of Interview: 15 August 2018**

**Type of Participant: Service Provider**

**Interview Number: D-43-0030**

**Interviewer: I.N.**

**Total Interview Time: 50 minutes 29 seconds**

**Interview Summary:** **(from summary sheet)**

| **SERVICE TO BE INTERGRATED** | **THOUGHTS ON INTERGRATION** |
| --- | --- |
| Couple HIV Testing and Counseling | Thinks its good development because there is a total participation on the wound care and on the counselling as well. |
| STI Services | Thinks it’s a good idea because they are closely related |
| Family Planning | He was confused and at first said they don’t offer family planning service at VMMC. But he think it’s a good idea |
| Cervical Cancer Screening | Thinks it’s a good idea as circumcision benefit men and since they is encouragement to be bring couples during VMMC so cervical cancer screening will draw women to be coming with their husband to VMMC |
| PrEP | Does not Like the idea of integrating PrEP with VMMC services but on a condition. PrEP can be provided to those at risk. |
| Other Services | Thinks all services should be integrated into VMMC as they link up at one point in time |

**Remarks:**

**Participant was relaxed, confident and open though he could contradict himself with the answers provided.**

**Interview Text:**

1. I: Aaah, thank you for taking the time to talk with me today. I would like to ask you some questions today about the way you feel and what you think about some issues related to the service you provide and how we can include other services in Voluntary Medical Male Circumcision clinics. There are no right or wrong answers to these questions. We would like to hear your opinion and your experiences in your own words. Aaah, do you have any questions before we begin?
2. *R: Aaah, no*
3. I: Okay…can you tell me how you are involved in the client care at this clinic?
4. R: *yaah I think they are number of sections we are involved in first is screening of the patients to check if they are legible to do circumcision*s and then we do the procedures and after the procedures 48 hours we review them and after 7 days we reviews them and we are supposed to be involved in other sections like HIV testing but they are other guys who are doing 5hat and again we do treat clients who are found with STI we treat them if they are found with STI then later after14 days after they are cured so we are also involved in STI clinic
5. I: Okay. Do your clients talk to you about how the services are provided here?
6. *R: mostly the don’t especially if you ask them questions*
7. I: Yea but can you give me an example of a time that your client talked to you about the services he received here?
8. *R: yes, especially if they experience a problem it’s when they talk if doesn’t they don’t talk*
9. Any example
10. R: *yah like today I meet two guys ahhhmmh who complained about wound infections and they wanted how they could be given an advice on how to Aaah Aaah how to they could continue aaa with the wound care*
11. I: Okay. Now let us talk about partner HIV testing here at the Voluntary Medical Male circumcision clinic.
12. *R: ehm*
13. I: Tell me what happens if a man brings a spouse here at the Voluntary Medical Male circumcision clinic?
14. *R: what happen is I think yaah yaah it’s not most of the times Aaah a client bring a spouse but if he does it’s a good thing because circumcision involves both of them because the client is done circumcision he has to abstain for 6 weeks and if that information is given to both of them it assist in the management of wound and also mmh a mmh the partner mean the ahh the married if he is a wife she also gets information about cervical cancer because circumcision helps to reduce cancer of the cervix so I think it’s good a client to bring the client they are number of benefit both of the can get so it worth to come together*
15. I: okay. What do you think are the motivators that make the men bring their spouses here for testing?
16. *R: the motivator mmh I think that question I won’t be able to answer because most of the times they come alone they don’t bring their spouse*
17. I: Okay, for those who do not bring their partners, what do you think demotivates men to bring their partners here for HIV testing and counselling?
18. *R: unfortunately, I have not asked the client that question I think it’s because of fear that if they are positive maybe their relationship won’t proceed and if the results turns to be nice may be they will later they may bring the partner for testing may be both but have never asked the client that question.*
19. I: Okay. So what do you think can be done to make men bring their partners here for couple testing and counselling?
20. *R. since I think it’s very difficult Aaah yaah to ask a client to bring the partner because VMMC is a voluntary thing so if you want to be done VMMC alone without a spouse I think I won’t be easy to convince him to bring the spouse unless maybe during Aaah message during Aaah mobilization if they start yaah giving information that clients Aaah Aaah clients yaah should give them information that clients must bring the spouse during circus ion maybe during that mobilization maybe it would work but to tell them to bring their spouse after they have already reported at the clinic for it* *VMMC won’t be possible*
21. I: Okay. You as a health care provider, what is your opinion on integrating couple counseling with Voluntary Medical Male circumcision services?
22. *R: yaah that’s a good thing but it won’t be easy because we usually have client of different ages others are around 30s others are younger other couples may not feel comfortable to get that information in environment where we have kids I think the man will feel unconfutable but in principle a good thin*
23. I: So you like it
24. *R: Yaah that a good thing*
25. I: So what can be done to overcome the concern that you have stated
26. *R: mmh may be here at Bwaila maybe they do separate them may be other in separate room kids in other separate room but in other centers they just mix may be because of space and in those centers may we have to find more space so that kids should get the HIV service separately from adults maybe in that way the adults may be not feeling unconfutable to bring their spouse during circumcision and whether also get hive services*
27. I: Now I would like to discuss with you about sexual reproductive health services and Pills for HIV prevention: called pre-exposure prophylaxis. (PrEP) Sexual reproductive health include services that promote good sexual health and reproduction. They include but not limited to family planning, cervical cancer screening sexual transmitted infection (STI) management, and many more. Today we will only discuss about family planning, diagnosis and management of STIs, Cervical cancer screening, and PrEP. We will look at each of these one by one. Let us start with STI services. Explain to me what happens if a client is suspected or diagnosed with an STI here?
28. *R: at* *VMMC clinic first we treat and send back the client and advice the client to come back after 14 days if he is better we will proceed with the circumcision and he is still not well we continue with the treatment*
29. I: Okay, so you as a health care provider what is your opinion on integrating STI services with Voluntary Medical Male circumcision services?
30. *R: that’s a good thing because booth they go together or if the patient is found with STI you can’t operate on them so we postpone until the client gets better so they g o together*
31. I: Okay. What is it that you do not like the integration of STI with Voluntary Medical Male Circumcision services? Why would you not accept the integration?
32. *R: nothing*
33. I: Okay… [Turning over the papers] Hmmm… How do you think STI services should be offered at the Voluntary Medical Male circumcision clinic?
34. *R: yes they should be one block otherwise if you refer the client to another I think the client may not turn back for* *VMMC he may get the treatment but not come back for VMMC*
35. *But what time should that service be offered*
36. *Yah should be the same day as soon as you diagnose the STI*
37. I: So, what do you think are the barriers and concerns on this integration?
38. *R: mmh barriers mmh doors open …. other site they may not have enough drugs for STI sometimes you refer the patient to another clinic which is not good other sites may be shortage of staff the same staff will treat the patient the same staff will offer some time we turn to have a large turn to other times its gets difficult to combine both patients at the same time sometimes it very difficult so shortage of staff may be the barrier*
39. I: what should be done
40. *R: it depends where they have shortage of staff you increase the staff and the drugs increase the drugs*
41. I: Aaah, now let us talk about family planning. Explain to me what happens if a client needs a family planning methods?
42. *R: mmh as I have already it’s not always to have a couple so in may experience I have not, meet a couple, one wanting VMMC and another one wanting family planning mmh.*
43. I: So what if a man comes for vasectomy what can you say or how can you deal with that?
44. *R: Aaah, I think family planning methods in circumcision I think from my experience I have never meet a couple wanting a family planning method in VMMC so I won’t be answer correctly*
45. *Let’s take it as a clinic*
46. *First we do VMMC and refer him back after he gets well we ask him to come back for other services he wanted first we do VMMC we can’t combine both because we want mage both wound I think it’s a burden to give the patient 2 wounds*
47. *I: What family planning method do you offer so far*
48. *R: I will lie to you. I don’t know it’s just condoms*
49. When do you provide condoms?
50. *Are given at 48 hours*
51. I: Okay. As a health care provider, what is your opinion on integrating Family planning in circumcision services?
52. *R: … yaah that’s a good thing because after circumcision we advise the patient to abstain for 6 weeks and after 6 weeks we provide condoms for ten months so it’s a good thing*
53. *R: nothing*
54. I: how do you think family planning methods should be provided within the clinic
55. R: *it depends because so family\planning methods are procedure but It may need a large space because other family planning methods may involve females and u can’t combine females and male is in the same room but with these spaces it won’t be possible and more staff*
56. In terms of place where should it be
57. The same building but different rooms
58. But when should the client receive these services
59. R: *if they are simple like condoms pills ye I as they can be given the same day on the day of circumcises even those involve the procedure the issue is pace and staff*
60. What do you think are other barriers?
61. *R mmh I mentioned space and staff mmh I think in the meantime that’s all unless maybe the staff may need more information and skills and may need more money*
62. I: But what do you think should be done to address that concern of stigma?
63. *R: resources that’s I mean staff money and space so the resources should be increased*
64. I: Now let us talk about cervical cancer screening. Explain to me what happens if a woman needs cervical cancer screening?
65. *R: Like here or…?*
66. I: Like here
67. *R: Aaah, we have not done that maybe at Bwaila hospital, clients are being referred to antenatal, where they do provide screening.*
68. *Assuming a woman has come at VMMC and she want cervical cancer screening*
69. *R I would refer her to Bwaila but not in VMMC block*
70. I:so as health care provider what is your opinion on integrating cervical cancer screening and *VMMC*
71. *R: yaah its good thing because Aaah at one point they are linking because we are saying VMMC to a certain extent it protects cervical cancer but to offer those services at one block that not a simple thing Aaah women need resources like rooms equipment’s trainings but it’s a good hang because they are liking each other*
72. *They have to be in separate rooms because VMMC we deal with male genital and cc we deal; with female genital so we can’t combine 2 different people in one room that is not a good thing because when we are doing these we need to provide privacy so combining booth of them in one room that bad thing*
73. I: so on your own opinion, if cervical cancer screening is included what would you like in this integration? What would you like about this integration?
74. *R:*
75. I: What is it that you would not like about cervical cancer screening integration with Voluntary Medical Male Circumcision services?
76. *R: Aaah mixing the services in one room is that I don’t like*
77. I: so, you would like if it’s on the separate room
78. R: yah even in the same building but different room.
79. I: Okay. What do you think are the barriers and concerns on this integration?
80. *R: yaah equipment and staff and space.*
81. I: Okay. So, what do you think should be done to address these concerns and barriers?
82. *R: pouring more resources*
83. I: Okay. Now let us discuss about PrEP. Have you heard about this before?
84. *R: Yea I have*
85. I: can you tell me; how did you learn about this?
86. *R: Aaah I learned about pep in may hive training*
87. I: so, what is the difference between pep and prep
88. *R: Aaah I did HIV training long time ago around 2012 so I haven’t done any training on HIV someone of these things so I tend to ix them up but I think its Aaah they are not very different from each other*
89. I: I will explain how the medicine works. PrEP is anti-HIV medicine that keeps HIV-negative people from being infected. That keeps HIV-negative people from being infected. I can give you an example. For instance, a man might be negative, he might be knowing the status of his wife, that she is positive. After taking the PrEP, they can be having unprotected sex but without getting a virus.
90. I: If PrEP was made available to HIV- men and women. Do you think you could advise your HIV negative clients to accept to take PrEP?
91. *R: yes, if they are exposed I would advise them*
92. I: What are the reasons you would encourage your clients to take PrEP?
93. *R: I think if the couple like one is positive and the other one which is negative but in a couple who are negative would not encourage them*
94. I: if PrEP was made available, what is your opinion on integrating prep with *VMMC*?
95. *R: it’s not very directly linked unless eeeh unless if it’s a couple where one is negative and the other one is positive in that case prep can be offered but it’s not directly linked*
96. I: Okay. So you are not supporting the integration?
97. *R: No am not because VMMC protects from HIV with 60% and we encourage use of condoms so with those two things I think it’s enough with the two thing and prep as you have already said it’s just increased the risk of resistance I think there is no use of Aaah combining PrEP and VMMC unless if there is a scenario where one is positive and the other is negative*
98. I: What if your client has been found negative and the wife is positive at home
99. *R: Aaah I can provide and the client has to take that for life*
100. I: So you don’t think it is important to provide your client with PrEP?
101. *R: No but I don’t like complete integration because we can be providing it for a life so it’s better to integrate it to a clinic where they will be full seen for life*
102. I: how can the problem be solved
103. *R: yaah we will be having the drugs but just temporary*
104. I: Let me ask you this question what is the main reason for providing *VMMC*
105. *R: It protects from HIV and hygiene and cancer*
106. I: So on HIV part of 60 % but prep protect 100% what if we combine the one with 100% and 60% how do you see it
107. *R: The only problem is compliance PrEP is taken for life but he is not sick so at some time he may be missing the dosage so the PrEP thing should be for temporarily just to protect myself just for one-time thing the problem is resistance.*
108. I: So what should be done to avoid that?
109. *R: Unless the client assured to take that for life*
110. I: Let’s now talk to our last part on other services. If you were given powers to choose and integrate services in Voluntary Medical Male Clinics, what are the services that you would think of Integrate?
111. *R: some of the services which we just talked about which do you select like STI condoms and youth friendly*
112. I: Explain to me what the reasons are for your choices
113. *R: they are linking up in one way or another*
114. I: Okay. So, how do you think these services should be offered in the clinic?
115. *R: mmh in terms of place they can be offered in the same block*
116. I: Thank you for taking your time to discuss with me today. Your answers will be very helpful in improving the health service delivery at Voluntary Medical Male circumcision clinics. Before we close, do you have anything to say?
117. *R: May be the questions on family planning and it may be seen as I was contradicting myself so am not sure how some of these answers will assist you in your stud because I was contradicting myself*
118. I: Aaah it happens but they are no problem.
119. THE END.

**D 43 STUDY**

**Date of Interview: 15 August 2018**

**Type of Participant: Service Provider**

**Interview Number: D-43-0030**

**Interviewer: I.N.**

**Total Interview Time: 50 minutes 29 seconds**

**Interview Summary:** **(from summary sheet)**

| **SERVICE TO BE INTERGRATED** | **THOUGHTS ON INTERGRATION** |
| --- | --- |
| Couple HIV Testing and Counseling | Thinks its good development because there is a total participation on the wound care and on the counselling as well. |
| STI Services | Thinks it’s a good idea because they are closely related |
| Family Planning | He was confused and at first said they don’t offer family planning service at VMMC. But he think it’s a good idea |
| Cervical Cancer Screening | Thinks it’s a good idea as circumcision benefit men and since they is encouragement to be bring couples during VMMC so cervical cancer screening will draw women to be coming with their husband to VMMC |
| PrEP | Does not Like the idea of integrating PrEP with VMMC services but on a condition. PrEP can be provided to those at risk. |
| Other Services | Thinks all services should be integrated into VMMC as they link up at one point in time |

**Remarks:**

**Participant was relaxed, confident and open though he could contradict himself with the answers provided.**

**Interview Text:**

1. I: Aaah, thank you for taking the time to talk with me today. I would like to ask you some questions today about the way you feel and what you think about some issues related to the service you provide and how we can include other services in Voluntary Medical Male Circumcision clinics. There are no right or wrong answers to these questions. We would like to hear your opinion and your experiences in your own words. Aaah, do you have any questions before we begin?
2. *R: Aaah, no*
3. I: Okay…can you tell me how you are involved in the client care at this clinic?
4. R: *yaah I think they are number of sections we are involved in first is screening of the patients to check if they are legible to do circumcision*s and then we do the procedures and after the procedures 48 hours we review them and after 7 days we reviews them and we are supposed to be involved in other sections like HIV testing but they are other guys who are doing 5hat and again we do treat clients who are found with STI we treat them if they are found with STI then later after14 days after they are cured so we are also involved in STI clinic
5. I: Okay. Do your clients talk to you about how the services are provided here?
6. *R: mostly the don’t especially if you ask them questions*
7. I: Yea but can you give me an example of a time that your client talked to you about the services he received here?
8. *R: yes, especially if they experience a problem it’s when they talk if doesn’t they don’t talk*
9. Any example
10. R: *yah like today I meet two guys ahhhmmh who complained about wound infections and they wanted how they could be given an advice on how to Aaah Aaah how to they could continue aaa with the wound care*
11. I: Okay. Now let us talk about partner HIV testing here at the Voluntary Medical Male circumcision clinic.
12. *R: ehm*
13. I: Tell me what happens if a man brings a spouse here at the Voluntary Medical Male circumcision clinic?
14. *R: what happen is I think yaah yaah it’s not most of the times Aaah a client bring a spouse but if he does it’s a good thing because circumcision involves both of them because the client is done circumcision he has to abstain for 6 weeks and if that information is given to both of them it assist in the management of wound and also mmh a mmh the partner mean the ahh the married if he is a wife she also gets information about cervical cancer because circumcision helps to reduce cancer of the cervix so I think it’s good a client to bring the client they are number of benefit both of the can get so it worth to come together*
15. I: okay. What do you think are the motivators that make the men bring their spouses here for testing?
16. *R: the motivator mmh I think that question I won’t be able to answer because most of the times they come alone they don’t bring their spouse*
17. I: Okay, for those who do not bring their partners, what do you think demotivates men to bring their partners here for HIV testing and counselling?
18. *R: unfortunately, I have not asked the client that question I think it’s because of fear that if they are positive maybe their relationship won’t proceed and if the results turns to be nice may be they will later they may bring the partner for testing may be both but have never asked the client that question.*
19. I: Okay. So what do you think can be done to make men bring their partners here for couple testing and counselling?
20. *R. since I think it’s very difficult Aaah yaah to ask a client to bring the partner because VMMC is a voluntary thing so if you want to be done VMMC alone without a spouse I think I won’t be easy to convince him to bring the spouse unless maybe during Aaah message during Aaah mobilization if they start yaah giving information that clients Aaah Aaah clients yaah should give them information that clients must bring the spouse during circus ion maybe during that mobilization maybe it would work but to tell them to bring their spouse after they have already reported at the clinic for it* *VMMC won’t be possible*
21. I: Okay. You as a health care provider, what is your opinion on integrating couple counseling with Voluntary Medical Male circumcision services?
22. *R: yaah that’s a good thing but it won’t be easy because we usually have client of different ages others are around 30s others are younger other couples may not feel comfortable to get that information in environment where we have kids I think the man will feel unconfutable but in principle a good thin*
23. I: So you like it
24. *R: Yaah that a good thing*
25. I: So what can be done to overcome the concern that you have stated
26. *R: mmh may be here at Bwaila maybe they do separate them may be other in separate room kids in other separate room but in other centers they just mix may be because of space and in those centers may we have to find more space so that kids should get the HIV service separately from adults maybe in that way the adults may be not feeling unconfutable to bring their spouse during circumcision and whether also get hive services*
27. I: Now I would like to discuss with you about sexual reproductive health services and Pills for HIV prevention: called pre-exposure prophylaxis. (PrEP) Sexual reproductive health include services that promote good sexual health and reproduction. They include but not limited to family planning, cervical cancer screening sexual transmitted infection (STI) management, and many more. Today we will only discuss about family planning, diagnosis and management of STIs, Cervical cancer screening, and PrEP. We will look at each of these one by one. Let us start with STI services. Explain to me what happens if a client is suspected or diagnosed with an STI here?
28. *R: at* *VMMC clinic first we treat and send back the client and advice the client to come back after 14 days if he is better we will proceed with the circumcision and he is still not well we continue with the treatment*
29. I: Okay, so you as a health care provider what is your opinion on integrating STI services with Voluntary Medical Male circumcision services?
30. *R: that’s a good thing because booth they go together or if the patient is found with STI you can’t operate on them so we postpone until the client gets better so they g o together*
31. I: Okay. What is it that you do not like the integration of STI with Voluntary Medical Male Circumcision services? Why would you not accept the integration?
32. *R: nothing*
33. I: Okay… [Turning over the papers] Hmmm… How do you think STI services should be offered at the Voluntary Medical Male circumcision clinic?
34. *R: yes they should be one block otherwise if you refer the client to another I think the client may not turn back for* *VMMC he may get the treatment but not come back for VMMC*
35. *But what time should that service be offered*
36. *Yah should be the same day as soon as you diagnose the STI*
37. I: So, what do you think are the barriers and concerns on this integration?
38. *R: mmh barriers mmh doors open …. other site they may not have enough drugs for STI sometimes you refer the patient to another clinic which is not good other sites may be shortage of staff the same staff will treat the patient the same staff will offer some time we turn to have a large turn to other times its gets difficult to combine both patients at the same time sometimes it very difficult so shortage of staff may be the barrier*
39. I: what should be done
40. *R: it depends where they have shortage of staff you increase the staff and the drugs increase the drugs*
41. I: Aaah, now let us talk about family planning. Explain to me what happens if a client needs a family planning methods?
42. *R: mmh as I have already it’s not always to have a couple so in may experience I have not, meet a couple, one wanting VMMC and another one wanting family planning mmh.*
43. I: So what if a man comes for vasectomy what can you say or how can you deal with that?
44. *R: Aaah, I think family planning methods in circumcision I think from my experience I have never meet a couple wanting a family planning method in VMMC so I won’t be answer correctly*
45. *Let’s take it as a clinic*
46. *First we do VMMC and refer him back after he gets well we ask him to come back for other services he wanted first we do VMMC we can’t combine both because we want mage both wound I think it’s a burden to give the patient 2 wounds*
47. *I: What family planning method do you offer so far*
48. *R: I will lie to you. I don’t know it’s just condoms*
49. When do you provide condoms?
50. *Are given at 48 hours*
51. I: Okay. As a health care provider, what is your opinion on integrating Family planning in circumcision services?
52. *R: … yaah that’s a good thing because after circumcision we advise the patient to abstain for 6 weeks and after 6 weeks we provide condoms for ten months so it’s a good thing*
53. *R: nothing*
54. I: how do you think family planning methods should be provided within the clinic
55. R: *it depends because so family\planning methods are procedure but It may need a large space because other family planning methods may involve females and u can’t combine females and male is in the same room but with these spaces it won’t be possible and more staff*
56. In terms of place where should it be
57. The same building but different rooms
58. But when should the client receive these services
59. R: *if they are simple like condoms pills ye I as they can be given the same day on the day of circumcises even those involve the procedure the issue is pace and staff*
60. What do you think are other barriers?
61. *R mmh I mentioned space and staff mmh I think in the meantime that’s all unless maybe the staff may need more information and skills and may need more money*
62. I: But what do you think should be done to address that concern of stigma?
63. *R: resources that’s I mean staff money and space so the resources should be increased*
64. I: Now let us talk about cervical cancer screening. Explain to me what happens if a woman needs cervical cancer screening?
65. *R: Like here or…?*
66. I: Like here
67. *R: Aaah, we have not done that maybe at Bwaila hospital, clients are being referred to antenatal, where they do provide screening.*
68. *Assuming a woman has come at VMMC and she want cervical cancer screening*
69. *R I would refer her to Bwaila but not in VMMC block*
70. I:so as health care provider what is your opinion on integrating cervical cancer screening and *VMMC*
71. *R: yaah its good thing because Aaah at one point they are linking because we are saying VMMC to a certain extent it protects cervical cancer but to offer those services at one block that not a simple thing Aaah women need resources like rooms equipment’s trainings but it’s a good hang because they are liking each other*
72. *They have to be in separate rooms because VMMC we deal with male genital and cc we deal; with female genital so we can’t combine 2 different people in one room that is not a good thing because when we are doing these we need to provide privacy so combining booth of them in one room that bad thing*
73. I: so on your own opinion, if cervical cancer screening is included what would you like in this integration? What would you like about this integration?
74. *R:*
75. I: What is it that you would not like about cervical cancer screening integration with Voluntary Medical Male Circumcision services?
76. *R: Aaah mixing the services in one room is that I don’t like*
77. I: so, you would like if it’s on the separate room
78. R: yah even in the same building but different room.
79. I: Okay. What do you think are the barriers and concerns on this integration?
80. *R: yaah equipment and staff and space.*
81. I: Okay. So, what do you think should be done to address these concerns and barriers?
82. *R: pouring more resources*
83. I: Okay. Now let us discuss about PrEP. Have you heard about this before?
84. *R: Yea I have*
85. I: can you tell me; how did you learn about this?
86. *R: Aaah I learned about pep in may hive training*
87. I: so, what is the difference between pep and prep
88. *R: Aaah I did HIV training long time ago around 2012 so I haven’t done any training on HIV someone of these things so I tend to ix them up but I think its Aaah they are not very different from each other*
89. I: I will explain how the medicine works. PrEP is anti-HIV medicine that keeps HIV-negative people from being infected. That keeps HIV-negative people from being infected. I can give you an example. For instance, a man might be negative, he might be knowing the status of his wife, that she is positive. After taking the PrEP, they can be having unprotected sex but without getting a virus.
90. I: If PrEP was made available to HIV- men and women. Do you think you could advise your HIV negative clients to accept to take PrEP?
91. *R: yes, if they are exposed I would advise them*
92. I: What are the reasons you would encourage your clients to take PrEP?
93. *R: I think if the couple like one is positive and the other one which is negative but in a couple who are negative would not encourage them*
94. I: if PrEP was made available, what is your opinion on integrating prep with *VMMC*?
95. *R: it’s not very directly linked unless eeeh unless if it’s a couple where one is negative and the other one is positive in that case prep can be offered but it’s not directly linked*
96. I: Okay. So you are not supporting the integration?
97. *R: No am not because VMMC protects from HIV with 60% and we encourage use of condoms so with those two things I think it’s enough with the two thing and prep as you have already said it’s just increased the risk of resistance I think there is no use of Aaah combining PrEP and VMMC unless if there is a scenario where one is positive and the other is negative*
98. I: What if your client has been found negative and the wife is positive at home
99. *R: Aaah I can provide and the client has to take that for life*
100. I: So you don’t think it is important to provide your client with PrEP?
101. *R: No but I don’t like complete integration because we can be providing it for a life so it’s better to integrate it to a clinic where they will be full seen for life*
102. I: how can the problem be solved
103. *R: yaah we will be having the drugs but just temporary*
104. I: Let me ask you this question what is the main reason for providing *VMMC*
105. *R: It protects from HIV and hygiene and cancer*
106. I: So on HIV part of 60 % but prep protect 100% what if we combine the one with 100% and 60% how do you see it
107. *R: The only problem is compliance PrEP is taken for life but he is not sick so at some time he may be missing the dosage so the PrEP thing should be for temporarily just to protect myself just for one-time thing the problem is resistance.*
108. I: So what should be done to avoid that?
109. *R: Unless the client assured to take that for life*
110. I: Let’s now talk to our last part on other services. If you were given powers to choose and integrate services in Voluntary Medical Male Clinics, what are the services that you would think of Integrate?
111. *R: some of the services which we just talked about which do you select like STI condoms and youth friendly*
112. I: Explain to me what the reasons are for your choices
113. *R: they are linking up in one way or another*
114. I: Okay. So, how do you think these services should be offered in the clinic?
115. *R: mmh in terms of place they can be offered in the same block*
116. I: Thank you for taking your time to discuss with me today. Your answers will be very helpful in improving the health service delivery at Voluntary Medical Male circumcision clinics. Before we close, do you have anything to say?
117. *R: May be the questions on family planning and it may be seen as I was contradicting myself so am not sure how some of these answers will assist you in your stud because I was contradicting myself*
118. I: Aaah it happens but they are no problem.
119. THE END.

**D 43 STUDY**

**Date of Interview: 15 August 2018**

**Type of Participant: Service Provider**

**Interview Number: D-43-0031**

**Interviewer: I.N.**

**Total Interview Time: 37 minutes 44 seconds**

**Interview Summary :( from summary sheet)**

| **SERVICE TO BE INTERGRATED** | **THOUGHTS ON INTERGRATION** |
| --- | --- |
| Couple HIV Testing and Counseling | Thinks it’s a good idea since it will helps to reduce complication that happens due to women provoking men into intercourse before 6 weeks since here they will both receive the counseling about the duration together. |
| STI Services | Likes the idea since couples will be able to access both services at one place saving them time of travelling to different hospitals |
| Family Planning | Also thinks it is a good idea as couples will be accessing these two services at one place also. |
| Cervical Cancer Screening | Likes the idea very much because it is uncircumcised men who causes cervical cancer to women so it’s good for both of them to understand this together |
| PrEP | Like the idea of integrating PrEP with VMMC services since negative partners will be protected from the virus but has concerns that men will start cheating on their wives due to the assurance that they can’t get HIV if they use the drug. |
| Other Services | Thinks all services should be integrated into VMMC as they fuse well |

**Remarks:** The participant was open and could explain more.

**Interview Texts:**

1. I: Thank you for taking the time to talk with me today. I would like to ask you some questions today about the way you feel and what you think about some issues related to the service you provide and how we can include other services in Voluntary Medical Male Circumcisionclinics. There are no right or wrong answers to these questions. We would like to hear your opinion and your experiences in your own words. Do you have any questions before we begin?
2. *R: no, there are no questions*
3. I: Can you tell me how you are involved in the client care at this clinic?
4. *R: aaah, I do the counseling and I do also provide the service provision*
5. I: Does your client talk to you about how the services are provided here?
6. *R: some do, some don’t*
7. I: Can you give me an example of a time that your client talked to you about the services he received here
8. *R: aaah, yeah, especially the adults, aaah, many worry, to them the duration which they stay after the circumcision., yeah because most of the men feel the six weeks of abstinence, is a long duration, so some they do indulge in sex activities before the six weeks, so mostly worry about the duration itself.*
9. I: Now let us talk about partner HIV testing here at the Voluntary Medical Male circumcision clinic. Tell me what happens if a man brings a spouse here at the Voluntary Medical Male circumcision clinic
10. *R: aaah, okay, aaah, if they are together, we do offer the service to both of them*
11. I: Have you ever seen a man coming with their spouse for HIV testing?
12. *R: yeah, yes*
13. I: What do you think are the motivators that make the men bring their spouses here for testing?
14. *R: the motivator? I think it depends on how they received the information of VMMC, because in counseling some do also emphasis on the need to bring the partner, so it depends on where they hear the information of the service, so that’s why some bring their partners*
15. I: What do you think for some of the needs for those clients, so they should be bringing their partners?
16. *R: I think aaah. To prevent the stress when men have when they just hear it from here from the hospital, aaah its better if we emphasis on the aaah, we take this as one key massages we can be offering to the men*
17. I: For those who do not bring their partners what do you think demotivates men to bring their partners here for HIV counseling and testing
18. *R: mostly it’s just because they are shy, most of the men are shy*
19. I: So what do you think can be done to make men bring their partners here for couple testing and counseling?
20. *R: aaah I think I said I said in acertain statement that we just need to give them the right information, yeah so that they can even make their own informed choice*
21. So you as a health care provider, what is your opinion on integrating couple counseling with Voluntary Medical Male circumcision services?
22. *R: aaah, I think yeah that one can be good because it will also help us to reduce some of the effects which other man gets, aaah since they will be with their spouse and when they’re in their homes the spouses will not provoke these men, but when a man just come alone, without their spouse, when there at home they are provoked, and making them sometimes they are erect causing complications like bleeding, so yeah*
23. I: So what do you think are the barriers and concerns on this integration?
24. *R: mmm, I think mostly is the culture in our setup, as you no married men, despite being a family mostly they are not able to see each other’s private parts, even in their homes they tend to switch off lights, they can’t bath together. So others think it’s a taboo for them to be circumcised while their spouse is there*
25. I: And about HIV testing and counseling as a couple?
26. *R: aaah, as a couple is very important, very important. When they are together, whether one is positive or both are positive, we give the information together so they can even encourage each other*
27. I: You talked about barriers, so what do you think can be done to overcome these concerns and barriers to couple counseling in Voluntary Medical Male circumcision services clinic?
28. *R: sensitization is what is needed*
29. I: Now I would like to discuss with you about sexual reproductive health services and Pills for HIV prevention: called pre-exposure prophylaxis (PrEP). Sexual reproductive health includes services that promote good sexual health and reproduction. They include but not limited to family planning, cervical cancer screening sexual transmitted infection (STI) management, and many more. Today we will only discuss about family planning, diagnosis and management of STIs, Cervical cancer screening, and PrEP. We will look at each of these one by one. So let us start with STI services. Explain to me what happens if a client is suspected or diagnosed with an STI here?
30. *R: aaah, yeah, since we do the examination, when we find one with STIs, we don’t even offer the service on that day, we first treat the client, and we require the client to bring their partner so they that they should also be treated for STLs. That’s what happens*
31. I: So you as a health care provider what is your opinion on integrating STI services with Voluntary Medical Male circumcision services?
32. *R: aaah, yeah they will be… when these services are integrated, we will have the chance to kill one bird aaah two birds with one stone. They will be no need for them to come here then to another clinic they will be assisted here, this can be one importance*
33. I: What is it that you do not like the integration of STI with Voluntary Medical Male Circumcision services?
34. *R: I think these two they go together*
35. I: So you like the idea
36. *R: yes, the idea of integration*
37. I: How do you think STI services should be offered at the Voluntary Medical Male circumcision clinic?
38. *R: I feel the STI drugs be available, be always available in the clinic*
39. I: So where, if we talk of place, where do you think it should be offered a Voluntary Male Medical clinic?
40. *R: I think we have got...we have a treatment room here, so when we find such client we treat them there in the treatment room*
41. I: Is it a private room?
42. *R: Yeah, is specifically for treatment and people should be treated in a private room*
43. I: What do you think are the barriers and concerns on this integration
44. *R: the barriers? Aaah, the only barriers are that aaah… most of the client when they come for the VMMC, some think when they have done the circumcision, hats the end of it, so they came like running away from the truth that they got STIs, so they want circumcision yet in true sense they got STIs, so since we screen them, we just find that ooh this one got STI, so like a barrier? What can I say? In terms of integration, if we combine these services, I don’t think they can be barriers to that*
45. I: So there are no any barriers?
46. *R: no*
47. I: So you like the idea?
48. *R: yes, that one is okay*
49. I: So lets us talk about family planning. Explain to me what happens if a client needs a family planning methods for instance men maybe wants vasectomy and other family planning methods for females?
50. *R: aaah. We refer them to other clinics, that’s what happens, if they want vasectomy, mostly we talk of BLM*
51. I: But do you have like one or two family planning methods that you offer?
52. *R: yeah, short term we do offer condoms*
53. I: As a health care provider, what is your opinion on integrating Family planning in circumcision services?
54. *R: hmmm, I think it can also be a good idea, now we are thinking of men bringing their spouse, this is the time we can also take a chance to offer the services to the spouse. The man will be getting circumcised while the partner will benefit from family planning, since they have escorted each other. Instead of referring to another clinic they will be assisted here.*
55. I: What is it that you would not like the integration of family planning in Voluntary Medical Male Circumcision Clinic*?*
56. *R: aaah, I don’t think there is anything that you can’t like on that one, I feel is one of the good ideas*
57. I: How do you think family planning services can be offered within Voluntary Medical Male circumcision clinics, like in terms of place, in the same place?
58. *R: it can be offered within the same building so much the better, because it can minimize the movement, when we say go to that building, you lose this one. Some of these people are shy anyway, if it is in the same building so much the better. It should be offered the time the time you are providing the service to the man, is the time you should also be assisting the woman.*
59. I: What do you think are the barriers and concerns with this integration?
60. *R: hmmm, I think the only barrier which can be there, maybe in terms of spacing, yeah that can be the only barrier because previously we couldn’t think of such services in the future. In case we use, like here we have got I think about two theatres but both are always full, here they will be another one so space can be challenge*
61. I: What do you think should be done to address these concerns and barriers?
62. *R: I think if we can just identify another room, if it can be possible, for the family planning issue, because otherwise as I was thinking that when we are assisting this one here, the other one will get in that room for privacy*
63. I: Now let us talk about cervical cancer screening. Explain to me what happens if a woman needs cervical cancer screening?
64. *R: yeah in fact, there is their clinic*
65. I: When you say their clinic what do you mean?
66. *R: mmmh there okay is a room where they do the screening, the visual, inspection using acetic acid, in thatroom is the room where the procedure is done in there, for cancer screening,*
67. I: So as a health care provider, what is your opinion on integrating cervical cancer screening in Voluntary Medical Male Circumcision Services?
68. *R: very, very, important, because most of these cervical cancers, it is us men who transmit the cancer to women, because the virus is with us men, so screening with VMMC I think they can really go together, this one is very, very, vital, I like it very much, because it’s us men who keeps the virus that cause cancer to these women*
69. I: What is it that you would not like about cervical cancer screening integration withVoluntary Medical Male Circumcision services?
70. *R: for this one I cannot have any dislike.*
71. I: But how do you think is the best way to offer cancer screening within Voluntary Medical Male circumcision clinics?
72. *R: ehm.., the only challenge I see maybe is because of spacing but if we could have a room for screening within, I think that can be important, within the same building special for cervical cancer screening.*
73. I: But in term of time?
74. *R: time?*
75. I: Time during maybe according to circumcision procedure when should this be done for instance we have come as a couple, should a man be there when screening for cervical cancer
76. *R: It can be a good idea for a man to be there, because sometimes it is us men who dothe screening so to avoid suspicions it’s a good idea for men to be available when doing the screening men.*
77. I: What do you think are the barriers and concerns on this integration?
78. *R: aaah... the concerns can be like I was saying , some of the providers who screen are men, so if you brought in your beautiful wife hahaha and you leave her in a room with a male service provider, so jealousy kicks in, you may not feel good so that can be bad.*
79. I: What do you think should be done to address these concerns and barriers?
80. *R: I think much which is needed is sensitization, we need to sensitize more, we need to give more information to these people, not just… on top of these other things we need to explain what is required when you want to do a certain procedure and you need to inform them that all medical practitioners whether male or female we do one thing. So yeah. Sensitize more*
81. I: Now let us discuss about PrEP. Have you heard about this before?
82. *R: yeah*
83. I: So tell me what you know about PrEP
84. *R: PEP is given to a client when one suspect that let say he slept a person whom he doesn’t know, maybe the condom burst or maybe they didn’t even put on a condom, but they don’t know each other so we offer PEP.But before offering PEP we do the testing to confirm whether one is positive or negative*
85. I: So that’s PEP not PrEP have you ever heard about PrEP?
86. *R: no*
87. I: I will explain how the medicine works. PrEP is anti-HIV medicine that keeps HIV-negative people from being infected. There is a single pill that is taken once daily, and if you take it regularly, it is highly effective at prevention people from being infected.
88. I: Now how do you feel about PrEP?
89. *R: aaah.., it’s a good one, it’s a good one, yeah.*
90. I: If PrEP was made available to HIV negative men and women, do you think you could advise your HIV negative clients to accept to take PrEP
91. *R: hmmm…yeah, I think so*
92. I: What are the reasons you would encourage your clients to take PrEP?
93. R: *since the virus cannot be transferred.*
94. If PrEP becomes available, what is your opinion on integrating PrEP with Voluntary Medical Male circumcision services?
95. R: *I think that one can be of good use since we are talking of partner involvement, we never know the outcomes during test, once the results are out, maybe one is positive and the other is negative, this is the good time to offer them PrEP as a couple. It can be good.*
96. I: So you would encourage the client to take PrEP
97. *R: yes, I could encourage client*
98. I: How do you think PrEP be offered in Voluntary Medical Male clinics?
99. *R: I think during the… aaah during after the testing when you counsel them after the whole process of counseling, I think that’s the high time you can give the PrEP.*
100. I: What do you think are the concern and barriers to integrating PrEP in Voluntary Medical Male services?
101. *R: hmmm. The only barrier which can be there its aaah. It will depend on how they got the information, because once they understand that when you take this drug you may not get the virus, most people may go like crazy going out with different women especially we men. That’s the only barrier. I can’t get the virus because I take this. That can be our main barrier*
102. I: What do you think should be done to address these concerns and barrier?
103. *R: I think during the counseling section, we need to emphasis that this is to protect them...Since they know the status of each other…aaah it’s not a certificate to be going out with different men or women otherwise they will contract the virus*.
104. I: Let us go to our last part which involves other services. If you were given powers to choose and integrate services in Voluntary Medical Male Clinics. What are the services that you would think of Integrate?
105. *R: aaah will start with the PrEP, this one can be integrated. Hmmm the what? cervical cancer screening, even the family planning. Yeah and any other services*
106. I: What other services?
107. *R: I think these are main ones, we do a lot of tests, diabetes*
108. Explain the reasons for your choices.
109. *R: I talked of PrEP, PrEP since it will …. It will prevent cross the infections of the partners, so is of paramount important. As for cancer screening is of paramount because I said it’s us male who transmit the virus, so it will be of paramount for women to know whether they already have cervical cancer. The family planning, it will reduce the movement of these couples, from… maybe instead of doing other things, today to family planning tomorrow to screening that can also help. The STIs also the same they will get the treatment at one place.*
110. I: How do you think these services should be offered in the clinic?
111. *R: like for PrEP I said after you have done the counseling and they have understood, that’s the time to offer the PrEP. The screening I think before doing the procedure of circumcision, I think the man and the woman can be together in the screening room, they can be together, and yeah, they can be together. And at the same time I think if you have family planning, if it can be combined with the cervical cancer screening right away. Yeah to reduce the movement, that one can be welcomed idea*
112. I: Thank you for taking your time to discuss with me today. Your answers will be very helpful in improving the health service delivery at Voluntary Medical Male circumcision clinics. Before we close, do you have anything to say
113. *R: hmmm, of course yeah, for me as a person I have to admit that if this can be integrated we can improve the services of VMMC, it can reach many, because even the spouses they will be accompanying each otherto the clinic because they can also benefit, if it can materialize it can be of benefit*
114. I: Anything else?
115. *R: anything else no. I just wish this should be a success.*
116. I: Again, thank you so much for taking your time to speak with me.
117. THE END.

**D 43 STUDY**

**Date of Interview: 16 August 2018**

**Type of Participant: Service Provider**

**Interview Number: D-43-0032**

**Interviewer: I.N.**

**Total Interview Time: 56 minutes 33 seconds**

**Interview Summary :( from summary sheet)**

| **SERVICE TO BE INTERGRATED** | **THOUGHTS ON INTERGRATION** |
| --- | --- |
| Couple HIV Testing and Counseling | Thinks it is a very important concept to be done because when they receive the service at VMMC, there is a period when the couple need to follow to facilitate wound healing and prevent other STI’s |
| STI Services | It is very good because it will be one way of motivating the clients |
| Family Planning | It may bring confusion because circumcision itself is a wider concept and then family planning is as well a huge concept so it will end up confusing the clients. |
| Cervical Cancer Screening | It is very important, because VMMC prevent cervical cancer and there are all the facility that provides the cervical cancer screening within vicinity so it would be easy to integrate. |
| PrEP | Don’t like the integration |
| Other Services | None |

**Remarks:** The participant was open, confident and knowledgeable.

**Interview Texts**:

1. I: I would like to ask you a few questions concerning what you think and feel about the services you provide and how we can include other services in voluntary male circumcision clinics. Aaah…. There are no right or wrong answers to these questions…we would like to hear your own opinion and experiences in your own words. Do you have any questions before we begin?
2. *R: No. I don’t.*
3. I: okay. So can you tell me how you are involved in the client care VMC clinic?
4. *R: hmmm am involved in several sercives….so as a VMC provider…. I am a VMC provider. In VMC, there are several areas we tackle, mainly I do circumcision, after circumcision I take care of clients before and after them until I see they are okay to leave.*
5. I: So does your clients talk to you about how the services are provided here?
6. *R: Yes. Soon after circumcision, we take them in the recovery room and there we ask them how the procedure went on and most of them respond that they feel happy and do not regret doing the circumcision. Myself have never come across a situation whereby a client complained about the procedures I did to them.*
7. I: so can you give me an example whereby a client talked to you about the service he received here?
8. *R: aaaahah… that was we call it post procedure exit interview…it was the time the patient was at the recovery and I asked him how the procedure went. And he said he was happy because he was a young man whom among his cycle of friends he was the only one who was not circumcised so he was feeling out of place each time his friends were talking about it and he felt good that finally he was one of them.*
9. I: now let us look at the couple HIV testing and counseling in integration with VMMC services. Now let us talk about partner HIV testing here at voluntary male circumcision clinics. So tell me what happens when a man brings his partner during male circumcision?
10. *R: Aaaahah…. During my time I haven’t come across such a situation and for that question the VMMC counsellors would be in a better position to answer.*
11. I: but have you seen any man bringing their spouses here at VMMC?
12. *R: no I haven’t seen anyone. Maybe because am new here.*
13. I: but what do you think are the motivators that makes men bring their spouses?
14. *R: aaaahah it’s difficult for me to bring that. But most men shun that most of the times they don’t they are encouraged to do that only a few individuals do but mostly they shun away from doing that. It’s different with the way it’s done in antenatal clinics. Here men choose to be reserved maybe because of their behaviors.*
15. I: but those who do not bring their partners, what do you think demotivates them to bring their spouses for HIV testing and counselling here?
16. *R: Most of the men are afraid to know their status….to be sure, so it might be because of how they have been behaving their style of living so most of them are afraid to go for an HIV test.*
17. I: but what do you think can be done to motivate men to bring their spouses here for an HIV test and counseling in integrating with VMMC?
18. *R: In most of messages, we include the essence of integrating couple counselling in HIV testing with VMMC when giving awareness. We give out the information that women when they accompany their spouses its good step to undertake because it gives them the rare chance to know their status and plan for the future.*
19. I: but you as a service provider. What is your opinion on integrating couple counselling with voluntary male circumcision services?
20. *R: it is a very important concept to be done because when they receive the service here, there is a period when the couple need to follow to facilitate wound healing and prevent other STI’s and in addition we tell them to stay at least six weeks before sexual intercourse and hygienically to take care of himself so they wife needs to be involved in all these stages to understand all these requirements. Plus, as you know circumcision does not hundred percent protect people from contracting HIV and AIDS so we encourage them to continue using condoms during sex to protect themselves and as well this message needs to be delivered to both partners.*
21. I: what do you think are the barriers and concerns on the integration of HIV testing and VMMC couple counselling?
22. *R: I think culture because men just don’t feel comfortable and their life styles they are afraid if I bring my partner and found positive. it would be hard for them to accept.*
23. I: so what do you think can be done to overcome the barriers you have just mentioned?
24. *R: I think one to one counselling…. Bringing information to clients on one to one basis. For instance, approaching families and deliver the information to them so that they should become aware of what happens when they come for VMMC and together see the essence of being involved in the services.*
25. I: so now let’s talk about sexual reproductive health services and pills for HIV prevention which are called pre-exposure prophylaxis {PrEP.). Sexual reproductive services include services that promote good sexual healthy, and reproduction. They include but not limited to family planning, cervical cancer screening, sexually transmitted infections managements. Today we will only discuss; family planning, diagnosis and management of STIs cervical cancer screening and Prep. We will look into each one of these. So let us start with STI services. Explain to me what happens if a client is suspected or diagnosed with STIs?
26. *R: Normally, the VMMC procedure include STI management, before client goes for circumcision and when is found with an STI we manage the STI and suspend the circumcision and put the client on treatment for two weeks and we tell them to come back to redo the test. When the test is negative we schedule them for circumcision. We do it right here.*
27. I: So you as a healthy care service provider, what is your opinion on integrating STI services with VMMC services?
28. *R: to make it like one stop service yes. It is very good because it doesn’t make sense for clients to come here for circumcision and then we tell them to go up there to receive the STI services. This will be one way of motivating the clients so this idea is very good.*
29. I: What is it you do not like about integrating STI services and VMMC?
30. *R: To me I can’t day I don’t like it…. Go ahead with it it’s a very good idea.*
31. I: but how do you think STI services should be offered at the VMMC clinics?
32. *R: no to its juts okay, because clients are seen from a provider in the screening room where all the assistance or services are done there. Clients feel comfortable to receive the services here in a private room without indicating on the door that this is an STI screening room and it doesn’t consume much time of the provider.*
33. Interviewer: What do you think are the barriers with this integration?
34. *R: I don’t think there is any problem. Because you clients advise each other sometimes to come and get tested based on the services received. So to me I see no barriers unless if the clients would voice out on their own. I think the clients benefit a lot from this strategy.*
35. I: okay. So now let’s talk about family planning, explain to me what happens when a client needs a family planning service here?
36. *R: One of them is condom use. But I have never come across a male client asking for a family planning service but normally we give information. We give services like information, pills to prevent early pregnancies, how condoms are used and others.*
37. I: so do you have those family planning pills here?
38. *R: here at VMMC we provide other services like protective healthy services but pills are not provided from here?*
39. I: so if somebody comes for vasectomy or if a female comes asking for a family planning loop what do u do?
40. *R: we usually refer them to a hospital because here VMMC clinic is solely for circumcision services so me as a VMMC provider cannot do vasectomy or provide loop because that is not in our jurisdiction.*
41. I: But what is your opinion on integrating family planning services with male circumcision services?
42. *R: like I said, it has to start from the grassroots. Provide information to the community because mostly men do not like to give the family planning services to their women when they come here. So we need to give information to the communities so that they are able to make choices because knowledge is power.*
43. I: what would you not like about this integration of male circumcision and family planning?
44. *R: It may bring confusion because circumcision itself is a wider concept and then family planning is as well a huge concept so it will end up confusing the clients.*
45. I: So how do you this family planning services can be offered within the voluntary male medical circumcision services?
46. *R: I just feel like I said, if we integrate these services it has to be on a greater stage because these concepts are wider so the challenge is that in terms of implementations these two concepts are not easy to deliver but in terms of information delivery they are very similar. And also remember that it depends on what you are prioritizing because these are two departments though these concepts. E.g. cancer goes together with VMMC because one of the purposes of VMMC is to prevent cancer. So these things really go together. E.g. women are the good customers of reproductive health services like cancer and family planning. Giving women information about VMMC benefits for sure women will influence their spouses to come for VMMC services. So if we integrate these services people will really benefit a lot.*
47. I: Now on the same issue of family planning, where within the VMMC would this family planning be offered, within the same place, same building, and time?
48. *R: hhhhmm it would be under the same roof but different rooms. Like what we have in the governments set ups you use the same door to get to under five. VMMC, family planning.*
49. I: so when should a man receive this family planning service…. before circumcision, after circumcision or within the circumcision process?
50. *R: after. It depends on what method you want to use because normally it’s done after the procedure. But for vasectomy and others. anytime is okay for a client to go and receive the services.*
51. I: so what did you say are the challenges, concerns or barriers of this integration of VMMC and family planning?
52. *R: ehm I didn’t say much on barrier. But I said these two things look different on implementation at the same time they go together for instance if you talk about VMMC at Reproductive healthy women will absorb it.*
53. I: so what do you think are the barriers of this integration?
54. *R: hmmm it depends on strategic planning. I can’t say because it hasn’t been incorporated yet so this time around I don’t have the answers to that one.*
55. I: okay. So let us now look at cervical cancer screening. So explain to me, what happens when a woman comes to do cervical cancer screening here?
56. *R: No on that one I have never came across here because as I said usually men do not bring their spouses when coming here. So to know much about that I don’t think I will have the right answer.*
57. I: but you as a healthy care service provider… What is your opinion regarding integrating cancer screening with VMMC?
58. *R: it is very important, because we have already talked of the benefits of VMMC which of them is to prevent cervical cancer and then we have all the facility that provides the cervical cancer screening within our vicinity so it would be easy to integrate.*
59. I: but what would you not like about the VMMC integration with cervical cancer screening?
60. *R: well, I don’t think I can say I don’t like something about this integration. It is important.*
61. I: but if we integrate these services, how do you think the best way would be to deliver these two services together?
62. *R: In my opinion, I feel it won’t do good to combine within the concept of VMMC to bring in cervical cancer screening. But if you would say how important is it that these women go cancer screening in relation to VMMC but for voluntary screening I think it should not be done under this same roof.*
63. I: so should we say you don’t like the idea of integrating cancer screening within the VMMC clinics?
64. *R: no the idea is fine because you talking of the same thing here. The tricky part is on the benefits of VMMC on cervical cancer prevention. So these things they go together so if this idea comes out it’s a good one. It will help to bring information to people on VMMC and cervical cancer prevention.*
65. I: so within the VMMC service…where do you think the cancer screening should be offered? Within the same room, outside or some private room?
66. *R: it should be offered in a private room because you need to consider privacy of the clients because some do not want to be known plus it would be good for partners because you may choose to do it together with your partners plus this would encourage couples.*
67. I: so what do you think are the barriers to this integration?
68. *R: the same thing with culture. Men do not usually want to mix with women. Always men want privacy very few challenges to say it’s okay but most are reserved.*
69. I: so how do you think these barriers can be resolved?
70. *R: of course through awareness. People should be aware and be able to absorb the information and be able to make choices.*
71. I: no let us discuss about PrEP. Have you ever heard about this concept of PrEP? before?
72. *R: is it PEP or PrEP?*
73. I: it PrEP not PEP. So you haven’t heard about this concept before?
74. *R: yes.*
75. I: okay so I will explain it to you. PrEP is an HIV medicine that keeps HIV negative people from being infected.
76. *R: but then you call it PEP…Post Exposure that’s why I asked if its PEP not PrEP. I think I know it...is it pre exposure for HIV?*
77. I: Yes. It keeps HIV negative people from being infected.
78. *R: yes, but us we normally use PEP that’s what is available here this PrEP have only seen it in literature.*
79. I: This PrEP is a pill that is taken once a day and if taken according to the instructions there are high chances of an individual not Contracting HIV. I would give you an example, if I know the status of my wife that she is HIV positive and am negative but I want unprotected sex I would be taking PrEP regularly on daily basis without me contracting the virus. So having explained that in this way, how do you feel about PrEP?
80. *R: by the way I have to know first, how far has PrEP been used in the world and has it been used on a wider range? Because it depends on the ones who have piloted it and effective it has been.*
81. I: the drug would only be effective if you follow the right procedure i.e. I think it would become very effective if inly taken as instructed by the physicians it works well.
82. *R: But I asked this question because if this comes on market for everyone’s use I believe it has been piloted on the wider range and am sure they have tried so to believe its effective we need that information.*
83. I: as of now I wouldn’t say that I would be able to give you that information but am sure it is there but what I know at this moment is what I just explained to you. What is it? How it works and so forth.
84. *R: I can’t say, PrEP has to be used unless it is scientifically effective and how long I will be taking PrEP. Will it be taken for sexual pressure only or it’s a lifetime drug like antiretroviral drug? Because this drug is different with PEP which is taken continuously for 28 days. My question is how long?*
85. I: But assuming it’s scientifically proven with no side effects and its effective and it works well. What would you say about this drug?
86. *R: Me I would love to remain with PEP for post exposure and condom use for sexual pressure.*
87. I: so what are your concerns if PrEP would be made available to HIV negative couples with HIV positive partners, would you encourage your clients to take PrEP?
88. *R: hmmm I don’t think I would because I have to know and now this is the moment where you need both partners to come for the testing and counsel them and then let them make their choices.*
89. I: so let’s say that a couple came for an HIV test together and one of them is negative while the other is positive. Would you encourage them to take the drug?
90. *R: this is why we always seek consent from the clients. the choice is to them to make but I would just provide the information required for them to make choices.*
91. I: would you encourage your clients to take PrEP?
92. *R: I don’t think so.*
93. I: why, what’s your concern?
94. *R: I think I can’t give a proper answer for that one because I think the drug is still under pilot now.*
95. I: so now let us talk about other services. If you would be given chance to integrate other services into VMMC clinics, what other services would you integrate into the VMMC clinics?
96. *R: because men run away from the services, I would add family planning, cervical cancer and STI management integration.*
97. I: can you give me the reasons for your choices?
98. *R: in family planning for instance vasectomy, u know current fertility is high and men don’t like family planning methods so if this becomes integrated in the VMMC clinics it would help to collect the current trend of population growth in the families. On the issue of cervical cancer, would help because it would give us a true reflection of how serious this issues are in relation to VMMC and it will be done on the wider focus because people listen to us a lot because they are more afraid of HIV than HIV. Men will appreciate the essence of VMMC in relation to cervical cancer prevention and the date generated would help to be aware how important it is to integrate the services.*
99. I: so how do you think these services should be delivered at the VMMC clinics?
100. *R: you should departments within the building, same entrance but different rooms so that screening of cancer and VMMC should be done together as you have suggested.*
101. I: okay. Thank you for taking your time to talk to me today. Your answers will be very helpful in improving the health service delivery at the Voluntary Male Medical Circumcision clinics. Before we close the interview, do you have anything to say?
102. *R: yeah I just want to say that the information given they will take the information provided and the way these questions have been structured it will help to improve delivery services. Because currently couple management is very poor in service delivery where by you see many couples getting the services on separate occasions so these questions will really be helpful.*
103. I: again thank you so much for your time and allowing to speak with me.
104. *R: thank you very much.*
105. THE END.

**D 43 STUDY**

**Date of Interview: 23 August 2018**

**Type of Participant: Service Provider**

**Interview Number: D-43-0039**

**Interviewer: I. N.**

**Total Interview Time: 33minutes 42 seconds**

**Interview Summary:**

| **SERVICE TO BE INTERGRATED** | **THOUGHTS ON INTERGRATION** |
| --- | --- |
| Couple HIV Testing and Counseling | Thinks its good idea because there is a total participation on the man’s care and on the counseling as well. |
| STI Services | Thinks it’s a good development because when, circumcision is for men. |
| Family Planning | Thinks it is a good idea as it will lead into men empowerment since most men do not participate in family planning. |
| Cervical Cancer Screening | Thinks it’s a good idea as circumcision benefits more men but at the same time the women, they are also benefiting from directly.so, for women to be sure that they are cervical cancer free and they are willing to undergo screening, it’s good to do them at same place. |
| PrEP | Like the idea of integrating PrEP with VMMC services |
| Other Services | Thinks all services should be integrated into VMMC but It should be offered depending on the likes of the client because it is good to what the clients like |

**Remarks:** Participant was open and confident.

**Interview Texts**:

1. I: Thank you for taking the time to talk with me today. I would like to ask you some questions today about the way you feel and what you think about some issues related to the service you provide and how we can include other services in Voluntary Medical Male Circumcision clinics. There are no right or wrong answers to these questions. We would like to hear your opinion and your experiences in your own words.
2. I: Do you have any questions before we begin?
3. *R: Aaah no*
4. I: Can you tell me how you are involved in the client care at this clinic?
5. *R:* *Aaah mainly am voluntary male medical circumcision provider* that’s how am chiefly involved here
6. I: what services do you provide?
7. *R: Here we provide circumcision as a major and wound care you know after circumcision we give those 48 hours so we also care for the wound sometimes will be urgent events we also take care of them*
8. I: Aaah Does your clients talk to you about how the services are provided here?
9. *R****:*** *Aaah since we have just started as a new site of course we are getting some feedback that the services are good yaah people are coming up with good feedback about the services.*
10. I: Aaah so can you give me an example of a time that your client talked to you about the services he received here?
11. *R...Aaah hmmm I can’t be specific on that one but however we have had sometimes when we call for review so there we happen to meet some clients of whom were offered services and they would say my wound is healing and getting better yaah so those are the services that they talk about*
12. I: Now let us talk about partner HIV testing here at the Voluntary Medical Male circumcision clinic. Aaah tell me what happens if a man brings a spouse here at the Voluntary Medical Male circumcision clinic
13. *R: okay Aaah we usually yaah u know when the man gets circumcised what happens we counsel the couple together that they should refrain from having sex since if they are to be involved or in touch in sex it means the wound the man would be traumatized and the wound will not be healed faster it will compromise the treatment*
14. I: What do you think are the motivators that make the men bring their spouses here for testing?
15. *R:* *Yah u know most of the time these things they go together when a man brings a woman it means the message will be sent will be like conveyed to the wife right there as compared to give a man a note to bring the spouse later on after the service when he brings the wife together then the counseling will take place right there.*
16. I: For those who do not bring their partners what do you think demotivates men to bring their partners here for HIV counseling and testing?
17. *R:* *Yah most of the times some men are not aah are not ahha like comfortable or they don’t tell their wife about the truth about themselves, their status so they feel like when they bring their partners in the facility and happen to be found that the status is not that good then it will compromise their relationship like end the marriage so that’s what happens.*
18. I: Yah ok what do you think can be done to make men bring their partners here for couple testing and counseling?
19. *R:* *Yah that is very important we are supposed to tell them the truth and give them the facts to what’s the benefit of having both of them tested because they will know their status so we need to tell them the actual results than just tell the other side of it without telling them the benefits of the service*
20. I: Okay you as a health care provider, what is your opinion on integrating couple counseling with Voluntary Medical Male circumcision services.
21. *R: You know it, it’s, it’s very important because the coming in aaah of male circumcision to some extent as we know it reduces the effects or the, the gravity of HIV infection to the I mean from woman to man so it also helps that they should know to what will happen later on because when they come together they will give us the actual feedback so it helps*
22. I: So you like the idea?
23. *R: The idea is perfect it reduces the impact the gravity of infection*
24. I: What do you think are the barriers and concerns on this integration?
25. *R: Aaah hmmm the barriers most of the times the barrier will be sensitization for the things to happen these people they need to be sensitized because there are a lot of misconception when we talk about circumcision so we need to cub all they misconception so that they should know the actual facts on VMC and give them the right information to myself the barrier as of now is information*
26. I: So what do you think can be done to overcome these concerns and barriers to couple counseling in Voluntary Medical Male circumcision services clinic?
27. *R:* *Hay I think that the partakers the ones taking part in programs like the program like VMC and HIV these people should work together in giving information to the mass so that people should know what it is all about they should use the radios posters and others so that the info should reach each and every aaah household*
28. I: Now I would like to discuss with you about sexual reproductive health services and Pills for HIV prevention: called pre-exposure prophylaxis (PrEP). Sexual reproductive health includes services that promote good sexual health and reproduction. They include but not limited to family planning, cervical cancer screening sexual transmitted infection (STI) management, and many more. Today we will only discuss about family planning, diagnosis and management of STIs, Cervical cancer screening, and PrEP. We will look at each of these one by one. Let us start with STI services. Explain to me what happens if a client is suspected or diagnosed with an STI here?
29. *R:*  *Okay aaah as a matter of fact most of the time we do not come across on this but then we also have them so first of all when we are doing VMC we screen the client when we screen the client and client happen to have STI we first of all treat the STI we give them the treatment depending on the severity of STI and we give them another date to come so yaah that’s what we do when somebody finds with STI*
30. I: You as a health care provider what is your opinion on integrating STI services with Voluntary Medical Male circumcision services?
31. *R:* *Yah yaah hmmm it is very important and I would urge that it should be the norm that STI treatment and VMC they should go together because we cannot provide VMMC see to somebody who is having STIs so it’s a simple reason because if someone is found with STI is that the STI will compromise the wound healing so I think it’s better that we combine these two things since they work hand in hand*
32. I: What is it that you do not like the integration of STI with Voluntary Medical Male Circumcision services?
33. *R:* *Come again...currently I don’t have anything that I don’t like its good*
34. I: So you like the idea of integration?
35. *R: because if we integrate the client will be receiving required services at that particular time*
36. I: How do you think services have to be offered at the VMMC clinic?
37. *R: The services have to happen during screen Aaah client should be treated specifically to the causative agent of that particular STI and if found with STI after treat and confirmed that the client is fine then we can agree on another date to come for* circumcision.
38. I: But in terms of place where should it be offered?
39. *R: Aaah I think the infrastructure will matter because at the clinic we will have the personnel who will be screening and after found with STI be referred to another point to get the treatment there the client will be told what to do next but it can be the same facility but different department*
40. I: What are the barriers?
41. *R: The barriers can be you know these issues partnering with still people receive it as shameful so they will not come aaah they come when things are getting worse so I think it will go back to the issue of information so people have to be told of the signs and symptoms like someone have UD I think information is the main barrier*
42. I: How best the barrier can be addressed?
43. *R: I think they should also be personnel who will be counseling because counseling is the focal point of these issues as I said they have to be told of the signs and symptoms of the diseases like say if you see you’re the urethra is discharging like abnormal discharges so that kind of information will help them*
44. Let us talk about family planning. Explain to me what happens if a client needs a family planning methods (vasectomy for men and family planning for female partners?
45. *R:* *We have designated rooms which are labeled like family planning services so they come direct t that room and tell us what they want so the straight come to that particular room.*
46. I: Have you ever come across a man seeking vasectomy?
47. *R: Only once*
48. I: So how was he helped?
49. *R: Aaah am not a provider of vasectomy so I received the man and I referred the man to the right room*
50. I: As a health care provider, what is your opinion on integrating Family planning in circumcision services?
51. *R: It will help to bridge the gap between the two so it will help to bridge the gap which has been there because there have been some miss understanding in between so it will bridge that gap in fact I would love to see all be doing things together*
52. I: What is it that you would not like the integration of family planning in Voluntary Medical Male Circumcision Clinic?
53. *R:* *There is nothing I can not like*
54. I: How the service should be offered?
55. *R: The very important thing is to have well trained people who know what to do we need to have these within the facilities*
56. I: In terms of place should it be placed?
57. *R: In terms of place it should be within the clinic but it has to be like in the other room but within the structure.*
58. I: Why not outside?
59. *R: You know when privacy have been compromised things don’t work so privacy have to be offered*
60. I: According to the circumcision process when should the service be provided?
61. *R: We will first offer circumcision services and give the man usually after six weeks for family planning services however will give the man a backup so I will still offer the family planning services with a lot of caution*
62. I: What do you think are the barriers and concerns with this integration?
63. *R:* *The barrier would be is that the information is not reaching the mass very well so I think the main barrier is that we should have the people well informed*
64. I: Explain to me what happens if a woman needs cervical cancer screening?
65. *R:* *Usually we have designated services where women will come for screen of cervical cancer it is well labeled so they come direct to the room current we do not offer these services at this department*
66. I: But do you offer this service?
67. *R: No we don’t*
68. I: So what do you do if the woman come for screening?
69. *R: The woman is referred to another service*
70. I: As a health care provider, what is your opinion on integrating cervical cancer screening in Voluntary Medical Male Circumcision?
71. *R: Yah it is very important cervical cancer screening will assist women to have a health life so the nation will be health as well so I suggest that it have to be done right here where VMMC is done*
72. I: What is it that you would not like about cervical cancer screening integration with Voluntary Medical Male Circumcision services?
73. *R:* *Nothing*
74. I: What is it that you like of the integration cervical cancer screening with Voluntary Medical Male Circumcision services?
75. *R: Every thing*
76. I: How do you think is the best way to offer cancer screening within Voluntary Medical Male circumcision clinics?
77. *R: Since cervical cancer chiefly deals with women and VMC deals with men I would love if cervical cancer is somewhere where VMC is done to avoid compromising privacy and comfort ability of these two parties so if it can be provided within the hospital but not where circumcision is done*
78. I: What do you think are the barriers and concerns on this integration?
79. *R: The barriers could be counseling because women also sometimes don’t know how their body works so information should be provided on how often screening should be done so information is the main barrier some come with advanced cervical cancer because they don’t know what to do.*
80. I: So what do you think should be done to address these concerns and barriers?
81. *R: I think in the outreaches where we go there should be personnel to sensitize women on cervical cancer like give them leaflets, cards well written and women read and understand how often they have to visit the hospital*.
82. I: Now let us discuss about PrEP. Have you heard about this before?
83. *R:* *I haven’t heard prep no but pep yes but I can relate it that it relates to HIV*
84. I: If you have not heard about PrEP, I will explain how the medicine works. PrEP is anti-HIV medicine that keeps HIV-negative people from being infected. There is a single pill that is taken once daily, and if you take it regularly, it is highly effective at prevention people from being infected. Now how do you feel about PrEP?
85. *R: well I think that’s a good development since I have never come across this one*
86. I: If PrEP was made available to HIV negative men and women. Do you think you could advise your HIV negative clients to accept to take PrEP?
87. *R: Yaah that’s obvious because*
88. I: If PrEP becomes available, what is your opinion on integrating PrEP with Voluntary Medical Male circumcision services?
89. *R: it is quite important. People with misconception will benefit from prep*
90. I: Would you encourage clients to take it?
91. *R. yes*
92. I: How do you think PrEP be offered in Voluntary Medical Male clinics?
93. *R: I think it should be given to every person like the way we do with HIV testing*
94. I: Within the clinic?
95. *R: Yes, within the clinic*
96. I: What do you think are the concern and barriers to integrating PrEP in Voluntary Medical Male services?
97. *R:*  *As of now I don’t know if there are any barriers but if there are no side effects then its fine*
98. I: What do you think should be done, if there are side effect, so to address these concerns and barrier?
99. *R:* *If there are side effects we can do as with ART and with this prep we can do the same*
100. I: Let us go to the last part which is Other Services. If you were given powers to choose and integrate services in Voluntary Medical Male Clinics, what are the services that you would think of Integrate? What are the services?
101. *R: Family planning, VMC, STI, Youth friendly services*
102. I: Can you Explain to me what the reasons are for your choices.
103. *R: Because it will be convenient to people because it will get rid of referrals and when someone gets out of the door will have received all the services*
104. I: How do you think these services should be offered in the clinic?
105. *R: It should be offered in a way aaah depending on the likes of the clients sometimes it’s good to what the clients like*
106. I: Thank you for taking your time to discuss with me today. Your answers will be very helpful in improving the health service delivery at Voluntary Medical Male circumcision clinics. Before we close, do you have anything to say?
107. *R: Thank you too*
108. THE END.

**D 43 STUDY**

**Date of Interview: 23 August 2018**

**Type of Participant: Service Provider**

**Interview Number: D-43-0040**

**Interviewer: I.N.**

**Total Interview Time: 40 minutes 43 seconds**

**Interview Summary:** **(from summary sheet)**

| **SERVICE TO BE INTERGRATED** | **THOUGHTS ON INTERGRATION** |
| --- | --- |
| Couple HIV Testing and Counseling | Thinks it’s good but testing itself is private service but so a consent is needed from another partner |
| STI Services | Thinks it’s a welcomed idea but it delays the process of circumcision because first the client is treated and given time to do circumcision sometimes few clients return |
| Family Planning | Thinks it is a good idea and it will lead into men and women participate in family planning. |
| Cervical Cancer Screening | Thinks it’s a good idea as circumcision is for men so cervical cancer screening will at the same time help women. |
| PrEP | Said the idea is good but would not encourage people to take it because its exposing them to the drugs which when they get HIV some other drugs may not work |
| Other Services | Thinks all services should be integrated into VMMC because they are similar and they can be done together |

**Remarks**: Participant was open, confident and relaxed.

**Interview Texts:**

1. I: thank you for taking the time to talk with me today. I would like to ask you some questions today about the way you feel and what you think about some issues related to the service you provide and how we can include other services in Voluntary Medical Male Circumcision clinics. There are no right or wrong answers to these questions. We would like to hear your opinion and your experiences in your own words. Do you have any questions before we begin?
2. *R: Aaah no questions you can proceed*
3. I: Can you tell me how you are involved in the client care at this clinic?
4. *R: okay am involved directly in providing the service because am service provider but also I do counseling*
5. I: what service do you provide besides counseling?
6. *R: The surgical procedure and counseling*
7. I: Does your clients talk to you about how the services are provided here?
8. *R. Some talk and some don not talk and some clients are in pain so they can’t express their views*
9. I: Can you give me an example of a time that your client talked to you about the services he received here
10. *R: I remember one time we had a client of course he came for VMMC and he had some STI so we first treated the disease and we rescheduled next time to be do so he appreciated he was treated for STI*
11. I: Now let us talk about partner HIV testing here at the Voluntary Medical Male circumcision clinic. Tell me what happens if a man brings a spouse here at the Voluntary Medical Male circumcision clinic
12. *R: First of all, testing itself is private service but if you get a consent I can have my partner with me we do it*
13. *So you have come across a situation where a man brings his spouse?*
14. I: What do you think are the motivators that make the men bring their spouses here for testing for those who do not bring their partners
15. *R; Motivating factors one it’s to know their status Aaah I don’t know maybe love and love because if they come together it means they agreed*
16. I: what do you think demotivates men to bring their partners here for HIV counseling and testing
17. *R; One is fear the know maybe they were not protecting themselves so coming for testing they think they may be found HIV positive*
18. I: What do you think can be done to make men bring their partners here for couple testing and counseling?
19. *R: I think when doing demand creation for VMC we can include messages for goodness of knowing the status*
20. I: You as a health care provider, what is your opinion on integrating couple counseling with Voluntary Medical Male circumcision services?
21. *R. Of course as of now we are going there its being integrated because VMC our entry point is HIV testing but integrating all these services it can be good.*
22. I: What do you think are the barriers and concerns on this integration?
23. *R: May be resources we may not have enough resources to offer certain services*
24. **I:** What do you think can be done to overcome these concerns and barriers to couple counseling in Voluntary Medical Male circumcision services clinic?
25. *R: May to lobby for funds from well wishers*
26. I: Now I would like to discuss with you about sexual reproductive health services and Pills for HIV prevention: called pre-exposure prophylaxis. (PrEP) Sexual reproductive health include services that promote good sexual health and reproduction. They include but not limited to family planning, cervical cancer screening sexual transmitted infection (STI) management, and many more. Today we will only discuss about family planning, diagnosis and management of STIs, Cervical cancer screening, and PrEP. We will look at each of these one by one. Explain to me what happens if a client is suspected or diagnosed with an STI here?
27. *R: We do examination so you find maybe he has features point to maybe to genital ulcer disease or urethra discharge so we have treatment scheme for different diseases*
28. I: You as a health care provider what is your opinion on integrating STI services with Voluntary Medical Male circumcision services?
29. *R: Yah it’s a welcome idea*
30. I: Why would you like it?
31. *Aaah some of the clients some are diagnosed with STI of course if you found a client with STI is a contraindication so we give treatment and wait for 14 days*
32. I: What is it that you do not like the integration of STI with Voluntary Medical Male Circumcision services?
33. *. R: hmmm of course it delays the process of circumcision because we first treat and we give some time to do circumcision sometimes few clients return it demotivates*
34. I: What is it that you like of the integration STI services with Voluntary Medical Male Circumcision services?
35. *R: Yes*
36. I: How do you think STI services should be offered at the Voluntary Medical Male circumcision clinic?
37. *R: We have to reduce time and distance referring to another level it’s like time wastage and making some patient get bored and go*
38. I: So where do you think these services should be offered?
39. *R It should be within the same facility but a private room*
40. I: What do you think are the barriers and concerns on this integration?
41. *R: may be lack of resources*
42. I: So what do you think should be done?
43. *R: train more staff who can be handling STIs and robbing for funds*
44. I: Now let’s talk about Family planning explain to me what happens if a client needs a family planning methods (vasectomy for men and family planning for female partners**?**
45. *R: I haven’t faced that challenge*
46. I: So what kind family planning do you offer here?
47. *R: We only provide barrier methods and condoms*
48. I: As a health care provider, what is your opinion on integrating Family planning in circumcision service?
49. *R: It’s a good integration but it will be difficult to get clients like female clients like irreversible family planning methods and injectable because mostly the clients we get age group from 10 -20 most of them are not married*
50. I: But what do you think should be done?
51. *R: … It is difficulty because we don’t get clients who are old but*
52. I: you like the idea of integration?
53. *R: Yes I do*
54. **I:** How do you think family planning services can be offered within Voluntary Medical Male circumcision clinics?
55. *R: … We do follow ups then this period we tell them not to have sexual intercourse because the wound is not healed and we do not offer condoms but after six weeks it’s when they can use condoms so that should be the best time to offer the services*
56. I: do you see the barriers and concerns with this integration?
57. *R: It’s the same issue of maybe low staff and lack of resources*
58. I: How can you address those
59. *R: The funds should be there and training more staff*
60. I: aaah lets go to Cervical cancer screening. Explain to me what happens if a woman needs cervical cancer screening?
61. *R: We do not offer that service here because at Bwaila it’s strictly VMMC so they are referred to Bwaila DHO*
62. I: As a health care provider, what is your opinion on integrating cervical cancer screening in Voluntary Medical Male Circumcision. Would you like the idea?
63. R: Yes, we can integrate
64. *I: What is it that you would not like about cervical cancer screening integration with* Voluntary Medical Male *Circumcision services?*
65. *R. Nothing*
66. I: What is it that you like of the integration cervical cancer screening with Voluntary Medical Male Circumcision services? You just like the idea
67. *R. Everything*
68. I: How do you think is the best way to offer cancer screening within Voluntary Medical Male circumcision clinics?
69. *R: We can start with those that come with their partners considering issues of privacy*
70. I: In the same room?
71. *R: it should be in separate rooms where all the resources are there*
72. I: What do you think are the barriers and concerns on this integration?
73. *R: I think lack of knowledge to service providers because there are not the same because there few people who know how to do cervical cancer screening*
74. I: What do you think should be done to address this barrier?
75. R: Orienting staffs on how to do the services
76. I: How can the staff be oriented?
77. R: through trainings can be on job or special *packages*
78. I: Now let us discuss about PrEP. Have you heard about this before?
79. *R. I don’t know about it I know about pep*
80. I: These are different so I will explain how prep works. PrEP is anti-HIV medicine that keeps HIV-negative people from being infected. There is a single pill that is taken once daily, and if you take it regularly, it is highly effective at prevention people from being infected. Now how do you feel about PrEP? Have you understood a bout PrEP?
81. *R: It feels good.* Are they the same
82. I: Aaah no they differ prep is taken before
83. I: If PrEP was made available to HIV- men and women. Do you think you could advise your HIV negative clients to accept to take PrEP?
84. *R: No I would not*
85. I: what are your concerns?
86. *R: You are exposing them to the drugs which when they get HIV some other drugs may not work*
87. I: Let us talk about other services. If you were given powers to choose and integrate services in Voluntary Medical Male Clinics, what are the services that you would think of Integrate? What are the services
88. *R: VMMC, HTC, Family planning and STI*
89. I: Explain to me what the reasons are for your choices.
90. *R: These are directly linked so integrating them won’t be a problem*
91. I: How do you think these services should be offered in the clinic?
92. *R: Within the facility we can have separate rooms*
93. I: Thank you for taking your time to discuss with me today. Your answers will be very helpful in improving the health service delivery at Voluntary Medical Male circumcision clinics. Before we close, do you have anything to say?
94. *R: no*
95. THE END.

**D 43 STUDY**

**Date of Interview: 23 August 2018**

**Type of Participant: Service Provider**

**Interview Number: D-43-0041**

**Interviewer: I.N.**

**Total Interview Time: 30 minutes 32 seconds**

**Interview Summary:** **(from summary sheet)**

| **SERVICE TO BE INTERGRATED** | **THOUGHTS ON INTERGRATION** |
| --- | --- |
| Couple HIV Testing and Counseling | Thinks its good development because there is a total participation on the wound care and on the counseling as well. |
| STI Services | Thinks it’s a good idea because when you are dealing with a man, circumcision, it’s all about men. Any complications that arise, in, in a man, has to be managed within a site. So, it’s good that the STI and the circumcision are done concurrent. |
| Family Planning | Thinks it is a good idea as it will lead into men empowerment since most men do not participate in family planning. |
| Cervical Cancer Screening | Thinks it’s a good idea as circumcision benefits more men but at the same time the women, they are also benefiting from directly.so, for women to be sure that they are cervical cancer free and they are willing to undergo screening, it’s good to do them at the spot [VMMCC] |
| PrEP | Like the idea of integrating PrEP with VMMC services but on a condition. PrEP can also have negative impact to the clients. |
| Other Services | Thinks all services should be integrated into VMMC as they fuse well |

**Remarks:** Participant was open and confident.

**Interview Texts:**

1. I: Thank you for taking the time to talk with me today. I would like to ask you some questions today about the way you feel and what you think about some issues related to the service you provide and how we can include other services in Voluntary Medical Male Circumcision clinics. There are no right or wrong answers to these questions. We would like to hear your opinion and your experiences in your own words. Do you have any questions before we begin?
2. *R: No*
3. I: Can you tell me how you are involved in the client care at this clinic?
4. *R: Aaah on the client care most especially I do provide the actual circumcision to patients and also assessment of the patient if they are fit enough for circumcision most especially we call it preventive care for the patient yah so I do the assessment as well as the procedure as well as the post assessment I mean so after the procedure we provide care to the patient*
5. I: Does your clients talk to you about how the services are provided here?
6. *R: Yah but not more often they do ask some questions patterning the procedure itself so we answer them the question according to what the ask*
7. I: Can you give me an example of a time that your client talked to you about the services he received here?
8. *R: Aaah an example can be like last week one of the client asked for how long will I be assisted for how long will I heal after circumcision that was on Wednesday so I told him its six weeks he just have to observe the wound clean it and if there are problems he has to contact us*
9. I: Now let us talk about partner HIV testing here at the Voluntary Medical Male circumcision clinic. Tell me what happens if a man brings a spouse here at the Voluntary Medical Male circumcision clinic?
10. *R: Aaah it’s a welcomed thing whenever when a client brings a partner because we want all of them to know their status*
11. I: What do you think are the motivators that make the men bring their spouses here for testing?
12. *R: Well most of the times what motivate them is if they want to know their status they just want to know their wife’s status this is a couple at least if they are negative they want to be sure that the wife is negative two so what motivates them is they just to know they just want to have knowledge about their status about the couple.*
13. I: For those who do not bring their partners what do you think demotivates men to bring their partners here for HIV counseling and testing?
14. *R: Yah may be they are afraid that if am positive so the wife will know that am positive that motivates them to bring their partners*
15. I: What do you think can be done to make men bring their partners here for couple testing and counseling?
16. *R: Yah the best thing is all about education to the clients they should know the importance of bringing the wife when they are having the circumcision*
17. I: But how do you thing this education can be given?
18. *R: a… it’s all about community sensitization think we need to go in the communities aaah whenever they mobilize and spreading the message about circumcision they should also emphasize on the inclusion of the partner when coming for the procedure.*
19. I: You as a health care provider, what is your opinion on integrating couple counseling with Voluntary Medical Male circumcision services?
20. *R: Well aaah as provider it will help because aaah hmmm aaah caring for patients its ehm Aaah we should combine their whole. family care is the best other than caring for one within you aaah a couple they are the ones that live in the house so testing one part I dint feel we are helping the other part at least if we test all of them it will be the best*
21. I: What do you think are the barriers and concerns on this integration?
22. *R: Well the barriers can be aaah you know we say is only for men so even women say I know circumcision is for men so why should I go there yaah it’s all about attitude for these women that it’s for men*
23. I: What do you think can be done to overcome these concerns and barriers to couple counseling in Voluntary Medical Male circumcision services clinic?
24. *R: Sensitization telling them the importance of the service the service to both mainly the HIV testing the importance of it to both the male and female sensitizing the community and the family about that I think we will then have appositive result at the end couples can began to get the services*
25. I: Now I would like to discuss with you about sexual reproductive health services and Pills for HIV prevention: called pre-exposure prophylaxis (PrEP). Sexual reproductive health includes services that promote good sexual health and reproduction. They include but not limited to family planning, cervical cancer screening sexual transmitted infection (STI) management, and many more. Today we will only discuss about family planning, diagnosis and management of STIs, Cervical cancer screening, and PrEP. We will look at each of these one by one. Let us start with STI services. Explain to me what happens if a client is suspected or diagnosed with an STI here?
26. *R: Okay well when the client is diagnosed with an STI Aaah we usually take that patient provide the treatment and we provide a note to say to the partner to say if I think this one should be given to the partner we give a not to give to the partner if he is having multiple partner we gave a not to all partners that they should come so that we should check them as well*
27. I: You as a health care provider what is your opinion on integrating STI services with Voluntary Medical Male circumcision services?
28. *R. Well aaah to my opinion its very good aaah because we will also detect STI it’s like killing two birds at once we are providing the treatment and STI*
29. I: What is it that you do not like the integration of STI with Voluntary Medical Male Circumcision services
30. *R: Aaah ha-ha what I don’t like about aaah its whenever we treat an STI patient most of them we send them back we don’t provide the service the same day we wait for them to heal and in so doing we miss some the client they don’t come back*
31. I: What is it that you like of the integration STI services with Voluntary Medical Male Circumcision services?
32. *R.. Yes*
33. I: How do you think STI services should be offered at the Voluntary Medical Male circumcision clinic?
34. *R: Aaah at least we need to have like a room for STI to see whenever we observe and STI client has to have its own room and should be viewed more often let’s take we have given treat for seven days and give another review date at least to be sure that the client has taken the treat and healed the services has to be provided when the client has healed*
35. I: What do you think are the barriers and concerns on this integration?
36. *R: The barriers can be people who provide the service you will find that we might be 8 people we might be the same people doing management of STI*
37. I: What do you think should be done to address these concerns and barriers?
38. *R: We need to have enough personnel other for VMC and other for STI management*
39. I: Let us talk about Family planning. Explain to me what happens if a client needs a family planning methods (vasectomy for men and family planning for female partners?
40. *R: It’s quite rear for male to look for vasectomy but male condoms so the best services we provide male condoms we don’t conduct vasectomy*
41. I: As a health care provider, what is your opinion on integrating Family planning in circumcision services?
42. *R: Well aaah it can be an added advantage aaah because other come for those family planning services so we will be having a lot of people for family planning as well as VMC so we will be catering for more services so we will have a lot of clients coming for it will be more helpful*
43. I: What is it that you would not like the integration of family planning in Voluntary Medical Male Circumcision Clinic?
44. *R: Work overloaded u will be doing VMC, family planning is big all they are, all services in family planning*
45. I: What is it that you like of the integration of Family planning with Voluntary Medical Male Circumcision services?
46. *R: everything*
47. I: How do you think family planning services can be offered within Voluntary Medical Male circumcision clinics?
48. *R: aaah it’s all about bringing couples, about couples coming for the services more especially women because in Malawi we leave these services to women more especially family planning but if we can have couple and sensitize the we can be providing good services to them I feel we need to couples need to come and get the services not just the individuals like male for VMMC*
49. I: In terms of place where should it be place?
50. *R: It should be outside the clinic of course we are integrating but we need a compartment for family planning*
51. I: What do you think are the barriers and concerns with this integration?
52. *R: The barrier can be location this Bwaila is like people are used to say this is for VMMC they can be like, should I go to (Name of Hospital) they just provide VMC yet there is family y planning*
53. I: What do you think should be done to address these concerns and barriers?
54. *R: Locate family planning and VMC close to each other but with different structure s as well as I think the information to people about family planning and VMMC*
55. I: Let us talk about cervical cancer screening. Explain to me what happens if a woman needs cervical cancer screening?
56. *R: Well it’s not easy usually we just refer them to Bwaila for screening yah because we don’t conduct that screening here*
57. I: As a health care provider, what is your opinion on integrating cervical cancer screening in Voluntary Medical Male Circumcision?
58. *R: Well aaah in the first place it can be very difficult for people to get used because VMC is concerned about men getting circumcised but if we can integrate by a by the can get used because Bwaila is center for Lilongwe and we can get more clients*
59. I: What is it that you would not like about cervical cancer screening integration with Voluntary Medical Male Circumcision services?
60. *R: What I cannot like about is I feel sorry about the altitude of the people they say the man who goes for VMMMC and now we are including female I think it can be a challenge for women they cannot come we cannot expect many women to come so why should we combine. To address it it’s all about campaigning the importance of it to tell them the importance of communicating the two if male is coming for VMC the female should also come for ccs if we can campaign about that we can have a positive thing*
61. I: What is it that you like of the integration cervical cancer screening with Voluntary Medical Male Circumcision services?
62. *R: If we can campaign say man should be coming for VMMC and say women coming for via if a man is coming should take his wife I think that’s the best time to do this*
63. I: What do you think are the barriers and concerns on this integration?
64. *R: It’s all about the allocation of a room where via can be done*
65. I: What do you think should be done to address these concerns and barriers?
66. *R: yah within Bwaila itself because at Bwaila we don’t offer VMMC we also offer other services if we can get one structure for via I think it can work it can be outside the clinic*
67. I: Now let us discuss about PrEP. Have you heard about this before?
68. *R: Yes*
69. I: If you have heard about PrEP, please tell me what you know about PrEP? How did you learn about this?
70. *R: Said it’s a combination Aaah I heard that one at art they of drugs that are given to someone before doing an act that is exposed to get HIV*
71. I: How do you feel about this?
72. *R: It is good it’s also has its disadvantages it can be abused by people I feel so it’s good because you will be protected*
73. I: If PrEP was made available to HIV negative men and women, do you think you could advise your HIV negative clients to accept to take PrEP?
74. *R: Yes, prevention is better than cure I would advise them to take it*
75. I: What are the reasons you would encourage your clients to take PrEP?
76. *R: Aaah they will meet high risk like situations*  we might go to occasion and they want to indulge in sex so at least if they have been protected previously I think they would stand a better chance
77. I: If PrEP becomes available, what is your opinion on integrating PrEP with Voluntary Medical Male circumcision services? Would you encourage clients to take it?
78. *R: Well I think aaah the whole incense of circumcision is to prevent someone getting HIV so if prep is added it’s a win-win situation*
79. I: So you would encourage your client to take it?
80. *R: Yah I will encourage them*
81. I: How do you think PrEP be offered in Voluntary Medical Male clinics?
82. *R: It’s not easy to provide it but it has to be it has been aaah given to those to keep the drugs and be given the reason why they are taken the prep not to just be distributed to anyone. In terms it has to be within the structures of VMC for control it can be given during the counseling stage*
83. I: What do you think are the concern and barriers to integrating PrEP in Voluntary Medical Male services?
84. *R: Yah the barriers can be people can prefer to get prep than VMC that can be a barrier so they will say VMC will do the same as prep I would prefer getting the prep*
85. I: What do you think should be done to address these concerns and barrier?
86. *R: I feel we need to emphasize more on the importance of both Its through whenever the client has come for any service has to be told about the availability of prep and the importance of VMMC not just to say those client has come for VMC and they should have told about prep even if its malaria it has to be told about prep and VMC*
87. I: Let us talk about other services. If you were given powers to choose and integrate services in Voluntary Medical Male Clinics, what are the services that you would think of Integrate? What are the services?
88. *R: First service can be provision of art because whenever the patient has to be tested before undergoing the procedure and we also find others who are HIV positive and instead of treating them we refer them so that if this client is positive right away he should be treated Others like prep*
89. I: Explain to me what the reasons are for your choices
90. *R: Well whenever a client has gone to the hospital she expects a lot more especially me whenever they have chance to go to hospital it’s time to tell them about other services and do assessment and offer treatment and when a client is going for VMC and we will have a chance to check STI and give information about family planning information about prep the main reason is that this client has to have knowledge about all these service* and the services
91. I: How do you think these services should be offered in the clinic?
92. *R: Aaah hmmm it’s a matter of having good structures that can offer these services within VMC because it’s all about allocating the room for VMMC this is a room for prep this is a room for family planning and when the client is going for one service he client has to undergo through all the services*
93. I: Thank you for taking your time to discuss with me today. Your answers will be very helpful in improving the health service delivery at Voluntary Medical Male circumcision clinics. Before we close, do you have anything to say?
94. *R: No*
95. THE END.

**D 43 STUDY**

**Date of Interview: 23 August 2018**

**Type of Participant: Service Provider**

**Interview Number: D-43-0042**

**Interviewer: I.N.**

**Total Interview Time: 46 minutes 35 seconds**

**Interview Summary:**

| **SERVICE TO BE INTERGRATED** | **THOUGHTS ON INTERGRATION** |
| --- | --- |
| Couple HIV Testing and Counseling | Thinks its good idea because there is a total participation on the man’s care and on the counselling as well. |
| STI Services | Thinks it’s a good development because when, circumcision is for men. So, it’s good that the ST same time I and the circumcision are done at |
| Family Planning | Thinks it is a good idea as it will lead into men empowerment since most men do not participate in family planning. |
| Cervical Cancer Screening | Thinks it’s a good idea as circumcision benefits more men but at the same time the women, they are also benefiting from directly.so, for women to be sure that they are cervical cancer free and they are willing to undergo screening, it’s good to do them at same place. |
| PrEP | Like the idea of integrating PrEP with VMMC services Because it will help men to maintain their HIV negative status but it have to be fully checked and sensitization before it starts |
| Other Services | Thinks all services should be integrated into VMMC but It should be offered depending on the likes of the client because it is good to what the clients like |

**Remarks: Participant was relaxed, open and confident.**

**Interview Texts:**

1. I: Thank you for taking the time to talk with me today. I would like to ask you some questions today about the way you feel and what you think about some issues related to the service you provide and how we can include other services in Voluntary Medical Male Circumcision clinics. There are no right or wrong answers to these questions. We would like to hear your opinion and your experiences in your own words. Do you have any questions before we begin?
2. *R: aaah no*
3. I: Can you tell me how you are involved in the client care at this clinic?
4. *R: Am a service provider*
5. I: Does your clients talk to you about how the services are provided here?
6. *R: Some clients do some not*
7. I: Can you give me an example of a time that your client talked to you about the services he received here
8. *R.: Like one time I had a 14 years old boy he came with a mum he also had a problem of which he applause the circumcision he had a condition which we call phimosis so it was the first time the fore skin was removed to see the gland*
9. I: Now let us talk about partner HIV testing here at the Voluntary Medical Male circumcision clinic. Tell me what happens if a man brings a spouse here at the Voluntary Medical Male circumcision clinic?
10. *R: If a man brings a woman we test them both because the counseling we give before vmmc so the supposed to listen together to what will happen after the wound*
11. I: What do you think are the motivators that make the men bring their spouses here for testing?
12. *R: Our outreach programs because for six weeks some spouses will not understand what’s going on with the man so the community mobilization we encourage married men to come with the partner.*
13. I: For those who do not bring their partners what do you think demotivates men to bring their partners here for HIV counseling and testing?
14. *R.: Fear because most men they dominate the decision making so they don’t even care how the other partner will feel after circumcision.*
15. I: What do you think can be done to make men bring their partners here for couple testing and counselling?
16. *R: I think it has to start in the community because we do community circumcision sensitization to strengthen the systems to say those that are married have to come with their partners*
17. I: You as a health care provider, what is your opinion on integrating couple counseling with Voluntary Medical Male circumcision services.
18. *R: I think it’s a good one because much as we are bringing me to vmmc and we are doing other services like STI and HIV testing.*
19. I: What do you think are the barriers and concerns on this integration?
20. *R. Most men like will come from work and make decision on their own and could not tell their wives that they are coming for vmmc*
21. I: What do you think can be done to overcome these concerns and barriers to couple counseling in Voluntary Medical Male circumcision services clinic?
22. *R: Sensitization in communities and tell them that wives can also come and tell them that we provide privacy for both of them.*
23. I: Now I would like to discuss with you about sexual reproductive health services and Pills for HIV prevention: called pre-exposure prophylaxis. (PrEP). Sexual reproductive health includes services that promote good sexual health and reproduction. They include but not limited to family planning, cervical cancer screening sexual transmitted infection (STI) management, and many more. Today we will only discuss about family planning, diagnosis and management of STIs, Cervical cancer screening, and PrEP... We will look at each of these one by one. Let us start with STI services. Explain to me what happens if a client is suspected or diagnosed with an STI here?
24. *R: If the client is suspected of having an STI we give them counseling and we give them treatment and write a referral to bring their partner and we gate their contacts to remind them*
25. I: You as a health care provider what is your opinion on integrating STI services with Voluntary Medical Male circumcision services
26. *R: I think it’s very good*
27. I: What is it that you do not like the integration of STI with Voluntary Medical Male Circumcision services?
28. *R: Aaah may be when you have someone they are not coming for vmmc and you are doing the screening and you find that they have an STI and you tell them you can’t do vmmc and they don’t return and don’t come back.*
29. I: What is it that you like of the integration STI services with Voluntary Medical Male Circumcision services?
30. *R: But if you have given them treatment it’s okay but it is disappointing*
31. I: How do you think STI services should be offered at the Voluntary Medical Male circumcision clinic?
32. *R: Aaah I think in the screening room within the clinic it shouldn’t be different rooms everything has to happen in the same room*
33. I: What do you think are the barriers and concerns on this integration?
34. *R: They have to go to other room for the medicine that’s the barrier for integration and if the medicines are in the same room like you screen and give them treatment right away*
35. I: Explain to me what happens if a client needs a family planning methods (vasectomy for men and family planning for female partners?
36. *R. I haven’t come across someone wanting vasectomy. Here we only provide condoms for other family planning methods we don’t do.*
37. I: As a health care provider, what is your opinion on integrating Family planning in circumcision services
38. *R: It is very good to integrate*
39. I: What is it that you would not like the integration of family planning in Voluntary Medical Male Circumcision Clinic?
40. *R: I think some men prefer to find ladies that are providers and have a congestion and some men will feel shy to mix will ladies*
41. I: How do you think family planning services can be offered within Voluntary Medical Male circumcision clinics?
42. *R: First we should have to sensitize the community and have readily available family planning services.*
43. I: in terms of place where should it be done?
44. *R: In terms of place I think within the clinic and in the screen room*
45. I: What do you think are the barriers and concerns with this integration?
46. *R: Some men would not want to see a lot of women at circumcision place*
47. I: What should be done?
48. *R: It’s a matter of educating the people in a way that they understand it batter*
49. I: Explain to me what happens if a woman needs cervical cancer screening?
50. *R: I have never come across that …we refer them to Bwaila clinic*
51. I: As a health care provider, what is your opinion on integrating cervical cancer screening in Voluntary Medical Male Circumcision?
52. *R: It’s a good idea*
53. I: What is it that you would not like about cervical cancer screening integration with Voluntary Medical Male Circumcision services?
54. *R: If we are encouraging couples and it is also good because most females don’t go we go when things are very bad so I think integrating it will be fine since we will be educating them at the same time*
55. I: What is it that you like of the integration cervical cancer screening with Voluntary Medical Male Circumcision services?
56. *R: I think time that’s what I will not like*
57. I: How do you think is the best way to offer cancer screening within Voluntary Medical Male circumcision clinics?
58. *R: If there is an establishment and start screen our women and maybe counseling them if they need further help and offer the treatment right away than referring them to Bwaila*
59. I: What do you think are the barriers and concerns on this integration?
60. *R: Time could not want to spend the whole day and congestion of women at vmmc clinic and some women will not come because the clinic is for men*
61. I: What do you think should be done to address these concerns and barriers?
62. *R: Education is very important in this case*
63. I: Now let us discuss about PrEP. Have you heard about this before?
64. *R: Yes*
65. I: If you have heard about PrEP, please tell me what you know about PrEP?
66. *R: It’s in the 2016 guidelines I heard it is encouraging people to be moving around*
67. I: Now how do you feel about PrEP?
68. *R: It should not be given to everyone it has to be given to key population like sex workers*
69. I: If PrEP was made available to HIV negative men and women. Do you think you could advise your HIV negative clients to accept to take PrEP?
70. R: Only to those at risk like prostitute I would advise them to take prep
71. I: Why you would encourage your clients to take PrEP?
72. *R: Because it will help them to maintain their HIV negative status*
73. I: If PrEP becomes available, what is your opinion on integrating PrEP with Voluntary Medical Male circumcision services?
74. *R: It is a good idea but it has to full checked and sensitization before it starts needs to be done*
75. I: Would you encourage clients to take it? Or do you think PrEP be offered in Voluntary Medical Male clinics?
76. *R: It should be done in consultation room because its where you interact and know better your client.*
77. I: What do you think are the concern and barriers to integrating Prep in Voluntary Medical Male services?
78. *R: Only give to key population or those at risk I think some people will be sent back*
79. I: What do you think should be done to address these concerns and barrier?
80. *R: There should only emphasizes that these key populations are getting prep through education*
81. I: Now Let’s talk about Other Services If you were given powers to choose and integrate services in Voluntary Medical Male Clinics, what are the services that you would think of Integrate?
82. *R: Initiation of ART & VMMC*
83. I: Explain to me what the reasons are for your choices
84. *R: These are rerated and have to be done together*
85. I: How do you think these services should be offered in the clinic?
86. R: *In the test room and if they are tested positive they can start ART right away*
87. I: Thank you for taking your time to discuss with me today. Your answers will be very helpful in improving the health service delivery at Voluntary Medical Male circumcision clinics. Before we close, do you have anything to say?
88. *R: No just want to thank you*
89. THE END.

**D43 STUDY**

**Date of Interview: 10 September 2018**

**Type of Participant: Service Provider**

**Interview Number: D-43-0051**

**Interviewer: I.N.**

**Total Interview Time: 17 minutes** 59 **seconds**

**Interview Summary:** **(from summary sheet)**

| **SERVICE TO BE INTERGRATED** | **THOUGHTS ON INTERGRATION** |
| --- | --- |
| Couple HIV Testing and Counseling | Thinks its good development because everything people will be coming to get everything |
| STI Services | Thinks it’s a good idea because you can’t do VMC with a client who is sick hence they are given treatment |
| Family Planning | Thinks it is a good idea because people will be coming to get whatever the service they want because the moment you refer them they don’t go to the services anymore |
| Cervical Cancer Screening | Thinks it’s a good idea because as we are building family planning we need to integrate cervical cancer so that when a woman comes she should get everything at once |
| PrEP | Like the idea of integrating PrEP with VMMC services because those that are negative then they get them so that they can maintain their negative status |
| Other Services | Thinks supposed to have family planning, the STI |

**Remarks:** Participant was relaxed and confident. She was able to explain more details but in shot time because she was in rush.

**Interview Text:**

1. I: Thank you for taking the time to talk with me today. I would like to ask you some questions today about the way you feel and what you think about some issues related to the service you provide and how we can include other services in Voluntary Medical Male Circumcision clinics. There are no right or wrong answers to these questions. We would like to hear your opinion and your experiences in your own words. Do you have any questions before we begin?
2. R: No
3. I: Can you tell me how you are involved in the client care at this clinic?
4. *R: I am a provider so we give them the information about circumcision and the importance of circumcision and the procedures that goes so that the client should know the procedure before going in the room*
5. I: Does your clients talk to you about how the services are provided here?
6. *R: sometimes they do, they give us feedback, and after they are done they give us feedback*
7. I: Can you give me an example of a time that your client talked to you about the services he received here
8. *R: when the client came back for review, he was excited there are rumors outside about how circumcision is done especially about the pain, and that you can’t even walk back home, he went back home alright, came back and safely remove the bandage*
9. I: Now let us talk about partner HIV testing here at the Voluntary Medical Male circumcision clinic. Tell me what happens if a man brings a spouse here at the Voluntary Medical Male circumcision clinic
10. *R: when a man brings a spouse we always acknowledge and thank them for bringing because when they come together they get the information together and the testing they will do together so it brings that unity between the family and unity between us because we are able to provide the service to the couple*
11. I: What do you think are the motivators that make the men bring their spouses here for testing?
12. *R: it always starts at home, it depends with how the relationship is with the wife and the information, like how is the information is dispersed, it starts at home, when there is good communication at home they always do things together*
13. I: For those who do not bring their partners what do you think demotivates men to bring their partners here for HIV counselling and testing
14. *R: Sometimes men are skeptical about their status, so what they want to do is to calm themselves first not many men are not courageous because of how they have been behaving*
15. I: What do you think can be done to make men bring their partners here for couple testing and counselling?
16. *R: as already said it starts at home, and us as providers, the information, we need to bring out that information, the importance in the community, so people should know the importance of couple testing*
17. I: You as a health care provider, what is your opinion on integrating couple counseling with Voluntary Medical Male circumcision services.
18. *R: integration is very good because when you do VMC people just think is the clinic for men, but when you do everything people will be coming to get everything, whether family planning, art, testing, STIs, like here, when someone Is HIV positive he is referred to Bwaila, of course it’s near but when somebody comes he has to get everything and not be referred outside, so the integration is important.*
19. I: What do you think are the barriers and concerns on this integration?
20. *R: maybe the staffing, to have the expert in both services, and the organization because it all needs money, so integration other services will need money*
21. I: What do you think can be done to overcome these concerns and barriers to couple counseling in Voluntary Medical Male circumcision services clinic?
22. *R: It starts with the organization planning, talking to the donors and tell them the importance maybe they can raise the funds to do the integration*
23. I: Now I would like to discuss with you about sexual reproductive health services and Pills for HIV prevention: called pre-exposure prophylaxis (PrEP). Sexual reproductive health includes services that promote good sexual health and reproduction. They include but not limited to family planning, cervical cancer screening sexual transmitted infection (STI) management, and many more. Today we will only discuss about family planning, diagnosis and management of STIs, Cervical cancer screening, and PrEP. We will look at each of these one by one. Let us start with STI services.
24. I: Explain to me what happens if a client is suspected or diagnosed with an STI here?
25. *R: we counsel, then treatment*
26. I: You as a health care provider what is your opinion on integrating STI services with Voluntary Medical Male circumcision services.
27. *R: it’s important because you can’t do VMC with a client who is sick who has an STI so when somebody comes here we do examination and when he has STI we give them treatment, then we proceed with the procedure later on after treatment*
28. I: What is it that you do not like the integration of STI with Voluntary Medical Male Circumcision services?
29. *R: there is nothing I don’t like because it helps in health*
30. I: How do you think STI services should be offered at the Voluntary Medical Male circumcision clinic?
31. *R: the way we offer them?*
32. I: yes
33. *R: the client comes for screening, when we do the screening and he has the STI we go for the treatment, we have another room for treatment so that there is privacy there*
34. I: so you wouldn’t want to change it?
35. *R: no it’s okay*
36. I: What do you think are the barriers and concerns on this integration
37. *R: mostly what happens when someone has a STI is that he doesn’t come again*
38. I: Let us talk about family planning. Explain to me what happens if a client needs a family planning methods (vasectomy for men and family planning for female partners?
39. *R: we refer them because we can’t offer the services*
40. I: As a health care provider, what is your opinion on integrating Family planning in circumcision services
41. *R: it is important because as I said, let people come and get whatever the service they want because the moment you refer them they don’t go to the services anymore because when they come they want that services at that time so it’s important*
42. I: What is it that you would not like the integration of family planning in Voluntary Medical Male Circumcision Clinic?
43. *R: there is nothing that I wouldn’t like*
44. I: How do you think family planning services can be offered within Voluntary Medical Male circumcision clinics?
45. *R: the thing is we need space of that family planning and VMC, so it means we need to spend something for training medical people, the need for fresher course so it’s an added cost*
46. I: should be outside or inside?
47. *R: it should be in as I said when somebody comes let that somebody get what he wants*
48. I: What do you think are the barriers and concerns with this integration?
49. *R: maybe the ladies will not be comfortable to come, because VMC is for male circumcision, so the turn up will not be as much as possible, but we need to send recent information to the community to change their mindset*
50. I: Let us talk about cervical cancer screening**.** Explain to me what happens if a woman needs cervical cancer screening?
51. *R: we refer because we don’t do it here*
52. I: As a health care provider, what is your opinion on integrating cervical cancer screening in Voluntary Medical Male Circumcision Services?
53. *R: as we are building family planning we need to integrate cervical cancer so that when a woman comes she should get everything at once*
54. I: so you like the integration?
55. *R: yes*
56. I: What is it that you would not like about cervical cancer screening integration with Voluntary Medical Male Circumcision services?
57. *R: there is nothing I will not like it because it provides the services to the people when they come*
58. I: How do you think is the best way to offer cancer screening within Voluntary Medical Male circumcision clinics?
59. *R: it means it means another space another trained stuff since people have to be trained so it is costly* I: when a client come like a couple, what’d you think these services should be offered?
60. *R: when they come as a couple we always give information and the services we offer so that they make the decision*
61. I: What do you think are the barriers and concerns on this integration?
62. *R: People may not be coming, they will be afraid, we Malawian are afraid to be scanned especially we women*
63. I: What do you think should be done to address these concerns and barriers?
64. *R: the information dispersion in the community*
65. I: Now let us discuss about PrEP. Have you heard about this before?
66. *R: I have*
67. I: If you have heard about PrEP, please tell me what you know about PrEP?
68. *R: it is an emergency that we give in case of middle pricking or splash*
69. I: do you take before you get injured
70. *R: The one we use here is after injury, somebody has got a splash that when we give them*
71. I: that’s PEP and that’s not PrEP
72. *R: so what is PrEP?*
73. I: If you have not heard about PrEP, I will explain how the medicine works. PrEP is anti-HIV medicine that keeps HIV-negative people from being infected. There is a single pill that is taken once daily, and if you take it regularly, it is highly effective at prevention people from being infected. Now how do you feel about PrEP?
74. *R: it’s good to have it, but we don’t have it*
75. I: yes, it’s not available. If PrEP was made available to HIV negative men and women do you think you could advise your HIV negative clients to accept to take PrEP?
76. *R: we will, unless we tell the importance*
77. I: What are the reasons you would encourage your clients to take PrEP?
78. *R: Not to be exposed not to be infected*
79. I: If PrEP becomes available, what is your opinion on integrating PrEP with Voluntary Medical Male circumcision services? Would you encourage clients to take it?
80. *R: because we do HIV testing, so people they come they are negative then they get them so that they can maintain their negative status*
81. I: How do you think PrEP be offered in Voluntary Medical Male clinics
82. *R: Yes*
83. I: What do you think are the concern and barriers to integrating PrEP in Voluntary Medical Male services
84. *R: it should be a free service and people should not pay*
85. I: in terms of place?
86. *R: it should be in the HTC where they do the HIV testing*
87. I: what kind of people should be given PrEP
88. *R: the high risk ratio group from 14 to 49 because they are sexually active*
89. I: What do you think should be done to address these concerns and barrier?
90. *R: there is need for a lot of education, people may think they are getting ARVs so we need to explain to them what prep is what it does.*
91. I: Let us talk about other services. If you were given powers to choose and integrate services in Voluntary Medical Male Clinics, what are the services that you would think of Integrate?
92. *R: family planning, the STI*
93. I: anything else?
94. *R: no, only those we have talked about*
95. I: Explain to me what the reasons are for your choices.
96. *R: because those concerns, when one comes as a couple we offer the services same with the STI*
97. I: How do you think these services should be offered in the clinic?
98. *R: in terms of what?*
99. I: timing
100. *R: Monday to Friday*
101. I: place
102. *R: the privacy, we will have the rooms demarcated for screening, STIs.*
103. I: Thank you for taking your time to discuss with me today. Your answers will be very helpful in improving the health service delivery at Voluntary Medical Male circumcision clinics. Before we close, do you have anything to say?
104. *R: No, thank you*
105. I: Again, thank you so much for taking your time to speak with me.

**D43 STUDY**

**Date of Interview: 10 September 2018**

**Type of Participant: Service Provider**

**Interview Number: D-43-0052**

**Interviewer: I. N.**

**Total Interview Time: 27 minutes 02 seconds**

**Interview Summary:** **(from summary sheet)**

| **SERVICE TO BE INTERGRATED** | **THOUGHTS ON INTERGRATION** |
| --- | --- |
| Couple HIV Testing and Counseling | Thinks its good development because women will be able to know their health status |
| STI Services | Thinks it’s a good idea because both are sexual and when somebody has been circumcised, chances of getting STI are minimal |
| Family Planning | Thinks it is a good idea because it’s a chance for someone who doesn’t know about family planning to know about it |
| Cervical Cancer Screening | Thinks it’s a good idea because cervical cancer is brought by men, if they can be brought at the clinic for screening it can be better |
| PrEP | Do not Like the idea of integrating PrEP with VMMC services because it’s like encouraging that there should be having sexual immorality |
| Other Services | Thinks supposed to have cervical cancer screening and family planning |

**Remarks:** Participant was confident and open but had negative attitude with PrEP.

**Interview Texts:**

1. I: Thank you for taking the time to talk with me today. I would like to ask you some questions today about the way you feel and what you think about some issues related to the service you provide and how we can include other services in Voluntary Medical Male Circumcision clinics. There are no right or wrong answers to these questions. We would like to hear your opinion and your experiences in your own words. Do you have any questions before we begin?
2. *R: No*
3. I: Can you tell me how you are involved in the client care at this clinic?
4. *R: I work as a surgical for VMC procedures, so mostly I work as a provider that is like a surgeon*
5. I: Does your clients talk to you about how the services are provided here?
6. *R: yes*
7. I: Can you give me an example of a time that your client talked to you about the services he received here
8. *R: normally when a client comes here they request a provider to give a council so whenever there is a problem that’s when they come to ask how the procedure was ad how to deal with the problem*
9. I: can you give me an example when your client talked to you anything specific
10. *R: Of course there was this other one who called me that he had a problem with passing out urine the problem was that during the time of the wound with a plaster it was too tight so I just advised him that he should loosen the plaster and be able to pass out urine then he will be ok*
11. I: Now let us talk about partner HIV testing here at the Voluntary Medical Male circumcision clinic. Tell me what happens if a man brings a spouse here at the Voluntary Medical Male circumcision clinic
12. *R: normally here we test the partners as they come individually, and this is done by the councilors and not by us*
13. I: What do you think are the motivators that make the men bring their spouses here for testing
14. *R: in fact, men can be bringing their spouses for testing because when they come here maybe they have already heard from friends that there is testing and when you go with your partner you are treated their so some do bring their partners and they get tested and if they are found positive we still circumcise them but the canceler advise them that they should go to the clinic where they provide ARV services*
15. I: For those who do not bring their partners what do you think demotivates men to bring their partners here for HIV counselling and testing
16. *R: maybe lack of information that they do not know that they are supposed to bring their partners, it’s not because they don’t want but because they don’t know so if they are made aware they can be assisted and even during cancelling, it can be better because we tell them to abstain from sex for 6 weeks so it can assist.*
17. I: You as a health care provider, what is your opinion on integrating couple counseling with Voluntary Medical Male circumcision services.
18. *R: I think it’s a good idea to interpret because women will be able to know their health status*
19. I: What do you think are the barriers and concerns on this integration
20. *R: the barriers I can say is lack of awareness, the second one is ignorance and sometimes especially working class people it will be very difficult to bring their partners*
21. I: What do you think can be done to overcome these concerns and barriers to couple counseling in Voluntary Medical Male circumcision services clinic?
22. *R: I think there should be an advocacy for that, awareness especially when they come here, we should encourage them to bring thee partners*
23. I: Now I would like to discuss with you about sexual reproductive health services and Pills for HIV prevention: called pre-exposure prophylaxis (PrEP). Sexual reproductive health includes services that promote good sexual health and reproduction. They include but not limited to family planning, cervical cancer screening sexual transmitted infection (STI) management, and many more. Today we will only discuss about family planning, diagnosis and management of STIs, Cervical cancer screening, and PrEP. We will look at each of these one by one. Let us start with STI services. Explain to me what happens if a client is suspected or diagnosed with an STI here?
24. *R: if a client has been diagnosed with an STI, automatically we treat them here if he comes alone we tell them to bring their partner for the same treatment*
25. I: You as a health care provider what is your opinion on integrating STI services with Voluntary Medical Male circumcision services.
26. *R: the integration is good because both are sexual and when somebody has been circumcised, chances of getting STI are minima than the one who is not circumcised*
27. I: *What is it that you do not like the integration of STI with Voluntary* Medical Male *Circumcision services?*
28. *R: there is nothing*
29. I: How do you think STI services should be offered at the Voluntary Medical Male circumcision clinic? In terms of place and time
30. *R: time there is no problem because we have got rooms that whenever someone has been screened and he is suffering from STI, there is another room for treatment and we give them the appropriate date to come again for the procedure, I think that’s the best way to do it, after treatment you tell them to come after a week or two weeks to view him, if he is okay automatically we circumcise him*
31. I: What do you think are the barriers and concerns on this integration?
32. *R: in fact, I can say that there is no much barrier because we combine them, before circumcision we do physical examination to know if he is okay if he is we circumcise him, so I don’t see any barrier since they are just incorporated*
33. I: Let us talk about family planning. Explain to me what happens if a client needs a family planning methods (vasectomy for men and family planning for female partners)?
34. *R: if that issue arises we cancel them and tell them to go where they will get the services, for example (Name of clinic) or anywhere where they offer these services*
35. I: As a health care provider, what is your opinion on integrating Family planning in circumcision services
36. *R: this is the time whereby we have a chance that when a client comes, maybe he doesn’t know about family planning, we take advantage of that he has arrived at the clinic, we also have to tell them about the family planning services and how he can prevent getting STIs, so I think this is the right forum and platform where we can also be assisting the client*
37. I: What is it that you would not like the integration of family planning in Voluntary Medical Male Circumcision Clinic?
38. *R: especially under children, who haven’t reached 18 years I think it’s like its limited we cannot talk to them about family planning, but those who are a bit older are the ones who we can tell about family planning services*
39. I: What is it that you like of the integration of Family planning with Voluntary Medical Male Circumcision services? How do you think family planning services can be offered within Voluntary Medical Male circumcision clinics?
40. *R: there should be an agreement that these services can be provided, most of us are nurses, technicians, so we have gone family planning services and we know how to provide them without any problem*
41. I: should they be offered under the same roof or outside?
42. *R: I think it should be inside, there are several rooms for family planning, there is vasectomy pills, injections, condoms, so if they are simple, they can be provided in the treatment room like injection or giving pills or inserting nail plant, but for the surgical procedure like vasectomy needs a special place or theater for that.*
43. I: when a client who came for circumcision also want family planning, how does it work?
44. *R: for his wife or him*
45. I: for him, maybe he wants vasectomy
46. *R: as of now we can just refer, we can do the circumcision then refer to the clinic where they offer vasectomy*
47. I: What do you think are the barriers and concerns with this integration?
48. *R: I think there can be a barrier for those who are not trained in family planning, it’s like they don’t know about family planning and give the services about family planning*
49. I: What do you think should be done to address these concerns and barriers?
50. *R: if there is need for the integration, those who are not trained should be trained on family planning*
51. I: Let us talk about cervical cancer screening. Explain to me what happens if a woman needs cervical cancer screening?
52. *R: we don’t let ladies for these services because they know this is for VMMC so they don’t come and ask for these services*
53. I: As a health care provider, what is your opinion on integrating cervical cancer screening in Voluntary Medical Male Circumcision services
54. *R: I think it can be better because* cervical cancer is brought by men, if they can be brought here for screening it can be better, we can be encouraging these women to bring their husbands for circumcision to avoid being infected with cancer
55. I: How do you think is the best way to offer cancer screening within Voluntary Medical Male circumcision clinics? In terms of place and time
56. *R: there can be a room special for that and they can be a special trained person who is trained in screening for cervical cancer*
57. I: What do you think are the barriers and concerns on this integration?
58. *R: the barriers is that if you are not trained in screening then it’s difficult to perform*
59. I: What do you think should be done to address these concerns and barriers?
60. *R: I have told you that, if this need to be incorporated then the ones who are supposed to be in the clinic are supposed to be trained*
61. I: Now let us discuss about PrEP. Have you heard about this before?
62. *R: Yes*
63. I: If you have heard about PrEP, please tell me what you know about PrEP?
64. *R: whenever you have been exposed to infectious blood and that you have been tested that you are negative and the blood you have contaminate then you have to take the drunk to avoid catching the virus*
65. I: How did you learn about this?
66. *R: we leaners when we do training. Each and every training we are told how to deal with people who have been exposed to any fluid*
67. I: is it taken before exposer or before exposer?
68. *R: it is taken after exposure; you have to take it within 72 hours*
69. I: that is pep, post exposure prophylaxis and prep is pre exposure prophylaxis.
70. *R: so what is PrEP?*
71. I: If you have not heard about PrEP, I will explain how the medicine works. PrEP is anti-HIV medicine that keeps HIV-negative people from being infected. There is a single pill that is taken once daily, and if you take it regularly, it is highly effective at prevention people from being infected. Now how do you feel about PrEP?
72. *R: that one is bad because you already know that this one is already infected and I will not catch it so you never know what will happen*
73. I: If PrEP was made available to HIV negative men and women, do you think you could advise your HIV negative clients to accept to take PrEP?
74. *R: no*
75. I: If No: what are your concerns?
76. *R: I think that one is like encouraging that there should be having sexual immorality and the best way is to abstain if you know that you are negative, you should not play with fire when you know that this one is positive*
77. I: Let us talk about other services. If you were given powers to choose and integrate services in Voluntary Medical Male Clinics, what are the services that you would think of Integrate?
78. *R: cervical cancer screening is the best and even family planning*
79. I: beside the services we talked about, any other you think of integrating
80. *R: checking for malaria, like somebody who is shivering you get to wonder what is happening to him, so when you can integrate, not those who are suffering from malaria should come here but for us, maybe when we suspect that this one has got sugar we can check, we got the thermometer*
81. I: Thank you for taking your time to discuss with me today. Your answers will be very helpful in improving the health service delivery at Voluntary Medical Male circumcision clinics. Before we close, do you have anything to say?
82. *R: No*
83. I: Again, thank you so much for taking your time to speak with me.
84. THE END.

**D43 STUDY**

**Date of Interview: 10 September 2018**

**Type of Participant: Service Provider**

**Interview Number: D-43-0053**

**Interviewer: I.N.**

**Total Interview Time: 28 minutes 10 seconds**

**Interview Summary:** **(from summary sheet)**

| **SERVICE TO BE INTERGRATED** | **THOUGHTS ON INTERGRATION** |
| --- | --- |
| Couple HIV Testing and Counseling | Thinks its good development because they are encouraging women of vulnerable groups so it can be a way to bring women here |
| STI Services | Thinks it’s a good idea because it’s a male environment so men will be comfortable getting the services at a VMC clinic |
| Family Planning | Thinks it is a good idea because men must be involved in all decisions so as taking a leading role in the family at household level, that would increase the uptake of family planning method. |
| Cervical Cancer Screening | Thinks it’s a good idea because women getting cancer screening on the same day as the husband is getting circumcised it will be time saving for them |
| PrEP | Like the idea of integrating PrEP with VMMC services because many men of multiple sexual partners can be using VMC as an entry point to get prep |
| Other Services | Thinks supposed to have family planning |

**Remarks:**

Participant was open, calm and confident.

**Interview Texts:**

1. I: Thank you for taking the time to talk with me today. I would like to ask you some questions today about the way you feel and what you think about some issues related to the service you provide and how we can include other services in Voluntary Medical Male Circumcision clinics. There are no right or wrong answers to these questions. We would like to hear your opinion and your experiences in your own words. Do you have any questions before we begin?
2. *R: No*
3. I: Can you tell me how you are involved in the client care at this clinic?
4. *R: to begin I work here as a VMC provider, this means that am involved in too many stages of the service which include, group education, screening of clients to certify eligible to go for circumcision I am also involved in the actual procedure, and follow ups and anything in between, sometimes we find clients with STI we also treat them*
5. I: Does your clients talk to you about how the services are provided here?
6. *R: yes, normally they are supposed to give us feedback after interviews so that they can come with their opinions on how they feel in terms of how we are providing the service*
7. I: Can you give me an example of a time that your client talked to you about the services he received here
8. *R: am new here, this is my second week so far I can’t say much but where I was I was also in a program providing VMC.*
9. I: you can even say whatever you discussed with your client where ever you were.
10. *R: where I was working before I remember one client said the service was quiet okay but he wasn’t okay with other attitude he saw with some providers, so he spoke about it, we held a meeting and discussed to find who was at fault.*
11. I: Now let us talk about partner HIV testing here at the Voluntary Medical Male circumcision clinic**.** Tell me what happens if a man brings a spouse here at the Voluntary Medical Male circumcision clinic?
12. *R: actually that’s what we encourage, we encourage our clients to bring their spouses so that they can have counselling so when they come here they walk together to the testing room where they receive counselling and they get tested*
13. I: What do you think are the motivators that make the men bring their spouses here for testing?
14. *R: the environment itself, plus also some men feel like it’s fair to bring their spouses for testing unlike going to KCH and the man should come here, so they prefer coming together*
15. I: For those who do not bring their partners what do you think demotivates men to bring their partners here for HIV counselling and testing?
16. *R: in my experience, some of the men that do not want to bring their partners, most of the time is because they already know their status, while others they know that they have been promiscuous so they are afraid that maybe the results will be positive and the partner will be angry at them so they prefer to come alone*
17. I: What do you think can be done to make men bring their partners here for couple testing and counselling?
18. *R: I would say if we prioritize people who come as a couple, maybe let’s say, we have 10 clients, 2 couples, then you prioritize the couple’s first so that they can receive the service earlier*
19. I: You as a health care provider, what is your opinion on integrating couple counseling with Voluntary Medical Male circumcision services.
20. *R: I think it’s a welcoming idea, because like in VMC we are encouraging women of vulnerable groups so that can be a way to bring women here, out their women think they are not supposed to come here, but it’s an issue that affect them, so they should come and hear for themselves because we don’t only offer VMC here but also we include HIV testing, STI screening and we can receive messages about cervical cancer so it’s a good idea to come for couple counselling.*
21. I: What do you think are the barriers and concerns on this integration?
22. *R: like I said earlier, women believe that VMC is just about men so I will start with the men, maybe some will be feeling some kind of ashamed to come here and meet women who has come with their partners, maybe it can also affect the services negatively, while some men may feel like it’s not right to be seen by women that they are coming to get the service*
23. I: What do you think can be done to overcome these concerns and barriers to couple counseling in Voluntary Medical Male circumcision services clinic?
24. *R: I think it’s all about information, when they come for group education, so what they can do is to separate couples those who have come as individuals should not do the education as a group*
25. I: Now I would like to discuss with you about sexual reproductive health services and Pills for HIV prevention: called pre-exposure prophylaxis (PrEP). Sexual reproductive health includes services that promote good sexual health and reproduction. They include but not limited to family planning, cervical cancer screening sexual transmitted infection (STI) management, and many more. Today we will only discuss about family planning, diagnosis and management of STIs, Cervical cancer screening, and PrEP. We will look at each of these one by one. Let us start with STI services. Explain to me what happens if a client is suspected or diagnosed with an STI here?
26. *R: at the facility we offer VMC as a package which includes STI screening so if a client is found with an or seen with symptoms of STIs are treated according to the guidelines and we make sure we have the necessary drugs for the STI, if we do not have the drugs we refer them to another facility*
27. I: You as a health care provider what is your opinion on integrating STI services with Voluntary Medical Male circumcision services.
28. *R: We all know that one of the barriers for men to receive care is that men do not like going to get help when they are sick because they are afraid of what the society will think so the VMC clinic is like their home ground, they feel comfortable to come here to receive STI services because it’s a male environment so I think it’s good that we do STI screening here*
29. I: What is it that you do not like the integration of STI with Voluntary Medical Male Circumcision services?
30. *R: so far the only set back is that some clients when you treat them for STI, this is a VMC clinic, so men are supposed to come here for VMC sometimes it happens that you treat a client for STI they don’t come back to come and do VMC, so it’s like you have lost a client, sometimes they come here and hear the message you treat them for STI, sometimes they report back to still get circumcised so it depends on the messages that they get out there, so if we only treat STI this can turn into an STI clinic and not VMC clinic.*
31. I: How do you think STI services should be offered at the Voluntary Medical Male circumcision clinic?
32. *R: the current set up is fine cause mainly STIs are treated in the screening room, that’s where we do legibility testing so when they are found with an STI they are treated because we keep a stock of drugs so currently it’s okay*
33. I: What do you think are the barriers and concerns on this integration
34. *R: so I will be just repeating what I already said*
35. I: can you just repeat what you said in a few sentences
36. *R: it can lead to maybe congestion here at the facility if people think that all we do is provide STI screening and treatment that means we will be having stock outs of the drugs that we keep here*
37. I: What do you think should be done to address these concerns and barriers?
38. *R: we can address this by encouraging that the clients we see that at the facility still come for the main service that we are offering that is VMC, it’s not compulsory but if they feel that it’s necessary because we all know that VMC reduces the chance of getting and STI*
39. I: Now let us talk about Family planning. Explain to me what happens if a client needs a family planning methods (vasectomy for men and family planning for female partners?
40. *R: currently we do not provide the service, we just refer them where they can get the service*
41. I: As a health care provider, what is your opinion on integrating Family planning in circumcision services
42. *R: I think it’s actually a good idea because men in our society must be involved in all decisions, they are the head, so if we involve them in family planning am sure that it will help us to address some of the barriers sometimes women may decide to come and receive family planning, but if they discuss it with the man but if they hear about it when they come for VMC it means they will be able to take a leading role in the family at household level, it will also increase the uptake of family planning method.*
43. I: How do you think family planning services can be offered within Voluntary Medical Male circumcision clinics?
44. *R: we can have a separate room within the VMC site for family planning so that if someone want family planning service we will not refer to another facility we will offer them here and it will save time*
45. I: What do you think are the barriers and concerns with this integration?
46. *R: space, because like at this facility almost every room is already occupied by a particular service, but maybe we can make other rooms, but we have limited space here*
47. I: What do you think should be done to address these concerns and barriers?
48. *R: like we do outside, if we do not have enough space we can have tents so that we can still provide the service because a family planning clinic doesn’t need much space*
49. I: Let us talk about cervical cancer screening. Explain to me what happens if a woman needs cervical cancer screening?
50. *R: just like I said with family planning, we just refer to facilities where we know they will get the service*
51. I: As a health care provider, what is your opinion on integrating cervical cancer screening in Voluntary Medical Male Circumcision Services
52. *R: for me I think it also be a very great idea because as I said earlier on we are encouraging women involvement in VMC so they should also see that they will gain something by coming here not only escorting the spouse but they can get HIV testing, cancer screening on the same day it will be time saving for them*
53. I: How do you think is the best way to offer cancer screening within Voluntary Medical Male circumcision clinics? Within or outside
54. *R: we can also create a separate space, if it’s possible we can combine with family planning facility like what they do in other clinics to save space and human resources since it will need more stuff*
55. I: What do you think are the barriers and concerns on this integration?
56. *R: I would say stuff as well as the space*
57. I: What do you think should be done to address these concerns and barriers?
58. *R: training of more stuff cause like here we have the stuff but am not sure that all of us are trained, like me I am not trained in cervical cancer screening and I don’t think my colleagues have been trained in cervical cancer so we will need training.*
59. I: Now let us discuss about PrEP. Have you heard about this before?
60. *R: yes*
61. I: tell me what you know about PrEP?
62. *R: I just know that it is pre exposure prophylaxis, that it can be taken before actual exposure to HIV*
63. I: How did you learn about this?
64. *R: I just see posters they are posted everything*
65. I: do you know how the medicine works
66. *R: not really*
67. I: I will explain how the medicine works. PrEP is anti-HIV medicine that keeps HIV-negative people from being infected. There is a single pill that is taken once daily, and if you take it regularly, it is highly effective at prevention people from being infected. Now how do you feel about PrEP?
68. *R: it has to be taken once dairy*
69. I: yes
70. *R: like ARVs, for life*
71. I: yes, but he difference is this is taken by those who are negative
72. *R: but the composition is like ARVs*
73. I: yes, so if you take it regularly its highly effective, now how do you feel about PrEP
74. *R; I don’t know, because people who are HIV positive still find it hard to stick to the regimen to take the pills on a daily basis so I don’t know if someone who is not infected to be taking the pills faithfully every day.*
75. I: you can have a partner who has a negative spouse to be taking PrEP
76. *R: so it’s for discordant couples*
77. I: exactly you can be taking the medicine as a couple or as a family
78. *R: is it just for discordant?*
79. I: no, it was just an example
80. I: If PrEP was made available to HIV negative men and women. Do you think you could advise your HIV negative clients to accept to take PrEP?
81. *R: yes, especially if they know that they are high risk clients like those who are sexually active and have multiple partners, I would encourage them to take PrEP*
82. I: If PrEP becomes available, what is your opinion on integrating PrEP with Voluntary Medical Male circumcision services? Would you encourage clients to take it?
83. *R: though I don’t have supporting data, but am sure that many men of multiple sexual partners, so VMC can be used as an entry point to get prep here since they are comfortable*
84. I: So you would encourage them to take them
85. *R: yes*
86. I: How do you think PrEP be offered in Voluntary Medical Male clinics?
87. *R: it can be offered like in the same way that we offer condoms here, we have a norm stat at every stage here, at HIV testing, follow ups and screening, I think it’s important to incorporate prep at every stage of VMC, maybe the client may not accept it at the clinic where they offer it, but they may accept it at a place they are comfortable in, so I would encourage prep at every stage of the process*
88. I: What do you think are the concern and barriers to integrating PrEP in Voluntary Medical Male services
89. *R: work load maybe of course right now we don’t have a lot of clients but normally the corridors are congested and you have many clients, so I don’t know if we will be providing multiple services at once on top of VMC we are providing, I don’t know, does it need a trained personnel who can provide*
90. I: on that issue I do not have the information
91. *R: because it’s not everyone who is supposed to prescribe ARVs, so I don’t know if we can need someone to prescribe prep, so we might need training as well*
92. I: What do you think should be done to address these concerns and barrier?
93. *R: training of stuff of what prep is and what it does*
94. I: Let us talk about other Services. If you were given powers to choose and integrate services in Voluntary Medical Male Clinics, what are the services that you would think of Integrate?
95. *R: the ones we have already discussed, especially family planning those are the only services I could think of*
96. I: no extra addition
97. *R: not really*
98. I: Explain to me what the reasons are for your choices.
99. *R: because we are still far behind with family planning here in Malawi, we already have the issue of over population, so it will do us better to interpret family planning in VMC*
100. I: How do you think these services should be offered in the clinic?
101. *R: having a separate room for the provision of the service and trained stuff*
102. I: Thank you for taking your time to discuss with me today. Your answers will be very helpful in improving the health service delivery at Voluntary Medical Male circumcision clinics. Before we close, do you have anything to say?
103. *R: No*
104. I: Again, thank you so much for taking your time to speak with me.
105. THE END

**D 43 STUDY**

**Date of Interview: 18 September 2018**

**Type of Participant: Provider**

**Interview Number: D-43-0058**

**Interviewer: I.N.**

**Total Interview Time: 31 minutes 25 seconds**

**Interview Summary:(from summary sheet)**

| **SERVICE TO BE INTERGRATED** | **THOUGHTS ON INTERGRATION** |
| --- | --- |
| Couple HIV Testing and Counseling | A good opportunity for couples to test together. Partner can motivate you to get VMMC. |
| STI Services | Thinks STI services would help more men to get VMMC.Fears the VMMC clinic would be crowded |
| Family Planning | The integration is a welcome idea only that it should only be offered to partners of VMMC clients |
| Cervical Cancer Screening | Feels cervical cancer screening is needed for couples that present with STI infections so the integration is a good idea |
| PrEP | A good idea as this integration would help to fight HIV better. |
| Other Services | Thinks TB screening could also be integrated with VMMC |

**Remarks:**

**Participant was very open-minded, gave straight forward responses to questions and he was curious about PrEP.**

**Interview Text:**

1. I: You are most welcome here, and thank you for taking the time to talk with me today.
2. *R: Thank you.*
3. I: I would like to ask you some questions today about the way you feel and what you think about some issues related to the service that you provide and how we can include other services in Voluntary Medical Male Circumcision (VMMC) clinics.
4. I: There is no right or wrong answers to these questions. We would like to hear your opinion and your experiences in your own words. Do you have any questions before we begin?
5. *R: No, I don’t have any questions.*
6. I: Ok.
7. *R: Sure.*
8. I: Alright, can you tell me how you are involved in the client care at this clinic?
9. *R: As VMMC care provider.*
10. I: Ok. But to be specific, what do you mean when you say VMMC provider? Which services do you provide?
11. *R:I provide services like, at start, following the procedures for the whole process such as being involved in screening the clients to see if they are fit for the procedure. And when they are fit for the procedure, we proceed with them for the circumcision procedure. I am also responsible for circumcising them. After all the procedures, I am also responsible for doing the reviews and check if they can really go home. So, after discharging them, whenever they come for the reviews, I am also responsible for doing the reviews.*
12. I: Ok. Do your clients talk to you about how the services are provided here?
13. *R: No.*
14. I: So you have never met a situation where a client expressed how the services are conducted or how the circumcision process takes place?
15. *R:I at once talked to a certain guy who expressed gratitude on how I circumcised him. But then, I have never had an opportunity to hear from the clients on the services that were provided by my colleagues. So all I know is about the clients that I offered services only.*
16. I:Alright, now let us talk about partner HIV testing here at the Voluntary Medical Male circumcision which is under couple testing and counseling. Tell me what happens when a man brings his spouse to the VMMC clinic?
17. *R: When a man brings a spouse at the VMMC Clinic, they are counselled on the importance of HIV testing. From the experience that I have, I saw a couple that had sexually transmitted infections. So when we called for the partner to come, they both went for HIV testing, and they were all tested.*

*.*

1. I: For those that bring their partners here what do you think motivates them to do so?
2. *R: I think what motivates them is the counsel that we give them about the importance of VMMC, because the focus is mainly on the prevention of HIV, and so they get motivated to call their spouses to come here so that they can be tested as well.*
3. I: Ok.
4. *I: Sure..*
5. I: And what do you think is the reason some men don’t bring their partners at the VMMC clinic for HIV counselling and testing?
6. *R: I think there is nothing in particular that we can say demotivates them, it’s just about their personal choices that they make because when we counsel them, most men talk about getting tested, but most of them don’t talk about bringing their spouses here, they are not willing to do so. So I don’t exactly what issues prevent them from bringing their spouses here.*
7. I: But in your opinion, what do you think prevents men from bringing their spouses here for HIV counselling and testing?
8. *R:But I suppose that most men know that women usually have access to HIV testing services. For example, pregnant women are supposed to be tested for HIV when they want to start antenatal care. I think that’s what make men to avoid bringing their spouses here because they assume that their spouses were tested already.*
9. I: Ok.
10. *R: Sure.*
11. I: So what do you think needs to be done so that men can be coming along with their wives for HIV testing as a couple?
12. *R: I think that is our responsibility. Whenever we go into the villages to inform them about the importance of VMMC, we also need to ask them to come with their spouses the way we ask women who come for antenatal to bring their spouses as well. So whenever we are asking them to come for VMMC, we need to ask them to come together.*
13. I: Ok!
14. *R: Sure.*
15. I: So, you, as someone who encourages men to get circumcised, what is your opinion on integrating couple HIV counseling with Voluntary Medical Male circumcision services?
16. *R: I think it’s a good initiative because when they come as a couple, it helps them to plan effectively, unlike when they come independently, a man knows is status, but a woman doesn’t know hers, it sometimes brings confusion to the family. They need to come together, so both can know their statuses.*
17. I: Ok!
18. *R: Sure.*
19. I: What do you think are the barriers and concerns on this integration?
20. *R: Most men say they are busy people at work, and that they only ask for a few hours from their work places to come here. So since they have a busy schedule, it can he challenging to ask their spouses to come here with them. The other barrier is that when we go out in the villages asking the clients to come, they come here in large numbers and when we start counselling each of them individually, it becomes time consuming to be done with one client, and that on its own is a barrier as well.*
21. I: Are there any concerns besides the barriers that you have mentioned?
22. *R: No, there are no any other barriers.*
23. What do you think can be done to overcome these barriers that you mentioned to couple counseling in Voluntary Medical Male circumcision services clinic*?*
24. *R: I think to overcome these barriers, we need to have more HIV Counsellors and more rooms for providing such services. Currently, we have only 2 staff, and considering the number of clients that come here, I don’t think its proportionate.*
25. I: Ok.Now I would like us to discuss about sexual reproductive health services and Pills for HIV prevention: called pre-exposure prophylaxis. (PrEP) Sexual reproductive health include services that promote good sexual health and reproduction. They include but not limited to family planning, cervical cancer screening sexual transmitted infection management cervical, condom distribution and many more. Today we will only discuss family planning, diagnosis and management of Sexual transmitted infection management cervical screening and PrEP. We will look at each of these one by one. Let us start with STI services.
26. *R: Ok.*
27. I: Explain to me what happens when a person is suspected or diagnosed with STIs?
28. *R: When a person is diagnosed with STIs, we treat them first. After treating the, we give them a notification slip which they are supposed to present to their sexual partners, and when their sexual partners come, we examine them as to whether or not they have the sexually transmitted infections.*
29. I: Alright.
30. *R: Sure.*
31. I: You as a health care provider, what is your opinion on integrating STI services with Voluntary Medical Male Circumcision services?
32. *R: It is a good service that needs to be integrated, and that’s what we are currently doing. So for that one, I can say that it is already integrated.*
33. I: Despite the fact that it is already integrated, what is it that you don’t like about the integration of STIs with VMMC?
34. *R: There is nothing that I don’t like about the integration.*
35. I: Ok.
36. *R: Yes.*
37. I: And what in particular do you like about the integration?
38. *R: It is good because when we are doing the screening, we cannot circumcise someone who has an active STI. The STI has to be treated first. |So the integration is good because it saves time, we do the screening, we find the infection, we treat it first rather than if the service was being accessed somewhere else, it could have been demotivating people, to say, I have to go again to this place. \Some of them could be running away, they could never show up. So the integration helps to provide care to the clients.*
39. I: Do you like the way STI services are being offered here at the VMMC?
40. *R: Yes.*
41. *I: Ok.*
42. I: What do you think are the barriers and concerns on this integration of STI services with Voluntary Medical Male circumcision services despite the fact that it is already in place?
43. *R:I think the concerns are to do with staffing, we sometimes receive a lot of clients who do not match with the number of providers here. We need to attend the clients here, we also need to be at the theatre.*
44. I: So what do you think can be done to address such concerns?
45. *R: During the mass campaigns, the need to have more providers has to be pointed out, and that the providers should be able to share the tasks.*
46. I: Ok!
47. *R: Yes.*
48. I: Okay. Now I want us to talk about family planning. Tell me what happens when a person comes to the VMMC clinic to access family planning? For instance, a man comes for vasectomy, or other family planning methods for his partner, what happens?
49. *R: When the clients come here for family planning methods, we usually refer them to Bwaila hospital.*
50. I: Ok.
51. *R: Yes.*
52. I: As a health care provider, what is your opinion on integrating Family planning in circumcision services?
53. *R: That is going to be good because we are also encouraging couple counselling, and again, when we have done the circumcision, we ask the couple to abstain from sex which is also another method of family planning and it is going to help in family planning to our clients who come here for VMMC.*
54. I: So you like the intergration?
55. *R: Yes.*
56. I: What is it that you would not like the integration of family planning in Voluntary Medical Male Circumcision Clinic?
57. *R:I think what I cannot like is about having large volumes of clients like maybe the clients that have come simply for family planning and not VMMC service. I think I there is any integration with family planning it should be provided only to those clients that come for VMMC not just any client who just comes for family planning because if we let it open it means we will be having large volumes of clients. So we might shift from providing VMMC to family planning.*
58. I: Okay. In terms of space, where do you think family planning service can be offered within VMMC clinic?
59. *R:Space can always be available, even the treatment room can be used to provide family planning service to the clients since most of these services don’t need complicated procedures.*
60. I: Say if a client comes for VMMC and needs vasectomy, when do you think this family planning service can be received by this client?
61. *R: I think he can get the service once the wound has started healing so that we shouldn’t compromise the healing process of the circumcision wound.*
62. I: Okay, what if they come as a couple?
63. *R:If they come as a couple then they can access it after counseling; that is after they have been taught about the benefits and dangers of the procedure.*
64. I: Okay.
65. *R: Yes*
66. I: Okay. What do you think could be the barriers or concerns on family planning and Voluntary Medical Male circumcision integration?
67. *R: The barriers can still be same as the barriers that we have during campaigns that we have a large volume of clients leading to high workload. But then when there is no campaign there won’t be any problem because there is usually not many clients.*
68. I: How can we deal with such concerns or barriers?
69. *R: I think during the time that have large volume of clients we need more staff. So there is need for more part-time that can cover the gaps that are created during that time*
70. I: Okay. Now let’s talk about cervical cancer screening. Tell me what happens when someone wants to get cervical cancer screening?
71. *R: When a woman needs screening we always refer them to Bwaila Hospital for the service*
72. I: As a health care provider, what is your opinion on integrating partner cervical cancer screening with Voluntary Medical Male circumcision services
73. *R: It’s going to be a good initiative because sometimes there are clients that present with issues say the husband has presented with an STI and the woman presents with bleeding after sexual intercourse which can be subject to screening for cervical cancer o check if it’s cancer or not.*
74. I: Do you like the integration?
75. *I: Yes a lot.*
76. I: What is it that you would not like about this integration of cervical cancer screening and VMMC?
77. *R: I think the issue is still that the service should be offered to couples where by the husband has come for VMMC and not for the general public who just come simply for cervical cancer screening. Or it could be clients who have done VMMC and have come for screening or they have just come with their wives who need cervical cancer screening.*
78. I: Okay.
79. *R: Sure.*
80. I: And how do you think this service can be offered in the VMMC clinic?
81. *R: I think the best way is to offer the service to those that have come as a couple and those that have an STI and have come for treatment because their partner has an infection. That’s the best way.*
82. I: In terms of space where exactly within the VMMC clinic can this service be offered?
83. *R: I think the same treatment room can be used.*
84. I: What do you think are the barriers and concerns for this integration?
85. *R: The concern could be the same one of workload whenever we are doing campaigns for more clients to come since the treatment room is also used for clients who have come for check-up.*
86. I: Okay.
87. *R: Yes, so space as well as workload can be some of the barriers.*
88. I: What do you think could be done there to deal with such barriers?
89. *R: I think during campaigns there should be part-time staff to help and also find another room within the clinic where cervical cancer screening could be done. I think that could also help.*
90. I: Okay. Now let us talk about PrEP. Have you ever heard about PrEP?
91. *R:Yes*
92. I: Can you tell me anything you have ever heard about PrEP?
93. *R: You are saying PrEP or PEP?*
94. I: PrEP
95. *R: Not PEP?*
96. I: No, PrEP.
97. *R: No I have never heard about PrEP. I know of PEP.*
98. I: Okay what is PEP?
99. *R: It’s a Post Exposure Prophylaxis. Maybe someone has had a needled prick from some suspected to be HIV positive, or maybe during sexual intercourse the condoms bursts and the other individual is HIV positive, then the one who is negative gets tested for HIV and if they are HIV negative then they start treatment to help them not to get infected*
100. I: Oh okay. So PrEP is Pre Exposure Prophylaxis.
101. *R: Oh okay.*
102. I: Yes, so PEP is “Post” while PrEP is “Pre”, that is before you get exposed. So I will explain how the medicine works. PrEP is an anti-HIV drug which helps to keep HIV-negative people negative. There is a single pill that one needs to take every day to prevent HIV infection. If taken daily, it is highly effective at keeping HIV negative people from being infected.
103. *R: Oh okay.*
104. I: So how do you feel about PrEP?
105. *R: I have gotten the meaning but I have so many questions.*
106. I: Mmm
107. *R: Is it given the same way anti-retroviral therapy is given or the way PEP is give that is to say there is a specific people or time to take the pills*
108. I: I think they have explained here that there is a single pill that’s take once daily, and it has to be taken regularly for it to be effective. So it’s like you keep on taking the pills.
109. *R: So you can even take it for life?*
110. I: Yes, you can take it for life.
111. *R: So it’s just the same as taking ARVs?*
112. I: I think so [chuckling]
113. *R: It’s my first time to hear about this.*
114. I: Okay, so if PrEP was made available to HI negative men and women, would you advise your clients to take PrEP?
115. *R: Yes, I can advise them if they are sexually active.*
116. I: Why would you advise your clients to be taking PrEP?
117. *R: Since you have said the use of PrEP is to prevent HIV infection, I think I can advise them to take it because it will help them not to get infected with HIV.*
118. I: Mmm. If PrEP become available, what is your opinion on integrating PrEP with Voluntary Medical Male circumcision services?
119. *R: I think I can accept the integration since the meaning of this program is about preventing people from HIV infection. So with the coming of PrEP I think I can still encourage that there should be that integration because it can even motivate mote clients to come knowing that after getting VMMc they will get PrEP.*
120. I: So you like the integration?
121. *R: Yes.*
122. I: Would you encourage your clients to take it?
123. *R: Yes I can encourage them to take it as long as I have understood the benefits of PrEP.*
124. I: Mmm. How do you think PrEP would be offered in this clinic?
125. *R:I think it should be offered to those that are sexually active and not to anybody.*
126. I: Okay, why do you think that way?
127. *R:It’s because I think those that are sexually active are at a higher risk of getting HIV than those that are not sexually active.*
128. I: What do you think would be the concerns about integrating PrEP into VMMC?
129. *R: I don’t think there can be any concerns because the service will be offered as part of care just the same way we offer them condoms when they come for VMMC. So PrEP can also be offered to them as part of care if they wish.*
130. I: Let’s go to the last part of this discussion which is other services. If you were given powers to choose and integrate services in Voluntary Medical Male Clinics, what are the services that you would think of Integrating?
131. *R: How many? Should I list all or...*
132. I: I mean other services maybe…
133. *R: Like those that have not been mentioned here?*
134. I: Yes.
135. *R: The other services that I would like to be integrated can be maybe medical care because sometimes there are some medical problems[door knock]. They can be offered medical service like TB screening which can also be integrated in the VMMC clinic because sometimes they come with such problems.*
136. I: You have mentioned about TB screening, what other services would you think of integrating with VMMC?
137. *R: Aah, no it’s only that one.*
138. I: Only TB.
139. *R:Yes*
140. I: Why have you chosen TB screening?
141. *R: It’s because TB is also common among those that are 20 years and above because of the risky behavior they engage in like smoking which puts them at a higher risk of contracting TB. So that’s why I have chosen TB.*
142. I: How do you think that service can be offered in the VMMC clinic?
143. *R: It can be offered during screening time. When they are doing the screening they can also ask some questions that will show if the patient may have signs and symptoms of TB before doing the procedure*
144. I: Thank you very much for your time. Your answers will be very helpful in improving the health service delivery at VMMC clinics.
145. *R:Mmm, thank you.*
146. I: Before we close, is there anything more you would like to say?
147. *R: I just have…maybe am just curious about the PrEP that you have mentioned. Is it that it’s currently available in the country or there is just a plan to make it available?*
148. I: Currently it’s not available that’s why we have asked “if it becomes available” which means it’s not yet available. But let’s hope it becomes available soon.
149. *R: Okay fine.*
150. .I: Okay. Thank you very much for talking to me today.
151. *R: Thank you.*

END

**D 43 STUDY**

**Date of Interview: 18 September 2018**

**Type of Participant: Provider**

**Interview Number: D-43-0060**

**Interviewer: B.M.**

**Total Interview Time: 36 minutes 43 seconds**

**Interview Summary:(from summary sheet)**

| **SERVICE TO BE INTERGRATED** | **THOUGHTS ON INTERGRATION** |
| --- | --- |
| Couple HIV Testing and Counseling | Thinks it’s very good idea if we integrate as will be able to assist many problems at once, like holistic approach |
| STI Services | It is good and very important |
| Family Planning | It is good because it is like having many services under one umbrella body |
| Cervical Cancer Screening | Think it is important because will reduce cervical cancer |
| PrEP | It is good idea because it will give chances to both parties or people who might be exposed to HIV virus. |
| Other Services | ART |

**Remarks:**

**Participant was very open-minded, gave straight forward responses to questions and he was confident.**

Interview Text:

- - 1. I: Thank you for taking the time to talk with me today. I would like to ask you some questions today about the way you feel and what you think about the service you provide and how we can include other services at voluntary medical male circumcision clinics. There are no right or wrong answers to these questions. We would like to hear your opinion and your experiences in your own words. So do you have any questions before we start?
    2. *R: no, you can go ahead*
    3. I: so, can you tell me, Aaah, how you are involved in the services in the circumcision clinics, rather you are working as who?
    4. *R: thank you… so much. Am a… VMMC service provider and am much involved in the services, whereby we eeh, we assist clients holistically right away from where, ehm, from the community where these people live. We have to convince them, so that they should understand the importance of voluntary medical male eehm, circumcision, so that eehm, we give them, chances to choose.*
    5. I: okay.
    6. *R: Whether to make a decision to come for the service or they can be given, time to think over it or to consult.*
    7. I: okay
    8. *R: After we are also involved at receiving them at our center. So we also screen them holistically for STIs, HIV and fitness to undergo procedure*
    9. I: okay. So, you are like involved in screening the participants
    10. *R: yea. We are involved in screening. We are also involved in examine if the client is fit for the procedure, there after I also operate and post-operative as well. So we work in all fields, in our areas.*
    11. I: so, do you talk to or participants talk to you about how the services are provided here?
    12. *R: yes. Some clients talk to us.*
    13. I: can you give me an example?
    14. *R: yes. Aaah, okay. I have been operating on some clients whom when they come to receive the services are afraid. They say, we thought it is as painful as other people say but have you finished the procedure? We ask to say, yes, we have done, aaah, only this, let me go and convince my friends because we have been afraid and we are coming for the procedure.*
    15. I: Aaah now let us talk about other services. Let us talk about HIV testing and counselling. We will talk about HIV testing here at VMMC with regards with partner involvement, that is partner HIV testing and counselling. Tell me, what happens if a man brings spouse here at VMMC clinic?
    16. *R: yea, since the services involving male, but when that male have been found with HIV, we ask that client to bring the partner, so that they can both benefit, even if during screening, we found that the client have STIs, then we sent that client, we give the treatment for the client, then we give a slip to give a partner to bring the partner to be treated.*
    17. I: okay, so they only bring them after you tell them, or they bring them voluntarily when they are coming for initial service.
    18. *R: there are some who come with their spouses when they are coming for VMMC. Some, they don’t come. They bring after*
    19. I: Okay. So what do you think motivates the men that bring partner here? What do you think are the motivators?
    20. *R: just thinking that maybe could be our environment, is really good, that we have got places where the partners can stay while the male, husband is being done, procedure and how we receive clients. I hope they are happy, with us.*
    21. I: okay. Now, so what do you think, does others not to bring partners, what do you think could be demotivates?
    22. *R: it could… Aaah, am not sure but am just thinking could be maybe lack of knowledge. They think male voluntary, male circumcision, since the name says male, its only male partner, male clients who come there but we have no limits for the partners to bring their partners*
    23. I: okay
    24. *R: for the clients to bring their partners*
    25. I: okay, so what do you think we can do to make men bring partners here for couple counselling and testing?
    26. *R: yea, we have to emphasize more on sensitization campaign that will help us to catch more because when many male bring their partners we may discover that, some of them might be tested positive, so they can benefit and they can also disseminate the good news of VMC to others*
    27. I: So, you as a health care provider what is your opinion on integrating this couple counselling and testing with VMMC services.
    28. *R: I see to it to be a very good idea if we integrate, then we will be able to assist many problems at once, like holistic approach*
    29. I: okay. So, in your opinion what could you think could be the barriers and concerns that this integration of couple counselling and testing could bring out?
    30. *R: yea, there might be many but just to mention few, could be distance, whereby most of them they just walk to assess the services despite that we are arrange for the outreach but we have not reached many areas. There are few areas that we go and it depends on the availability of funds, donor and other factors. And could also be cultural values of that community because there are some cultures up to now, they have not accepted the circumcision. Could be time, when the circumcision is done because most campaigns are done during holidays it seems as if it is targeting only the students, yet there are other people who are also married, so during that period if we can do more campaigns, we can also increase the number of clients coming. The final could be lack of knowledge. There is knowledge gap of the benefits of all the importance of circumcision.*
    31. I: okay, so what should we do? With all the factors you have mention, what do you think we should do?
    32. *R: if we can add more outreach clinics, only if … can volunteer themselves to assist. If for example, (name if sponsor), we might have one donor but if more done can come, that we are going to fund outreach clinics only, so that you can add more clinic days. Then other, if we can do more sensitization campaigns on the importance of circumcision, so that other cultures should be diluted.*
    33. I: okay. Now I would like to discuss with you about sexual reproductive services and pills for HIV prevention called Pre exposure Prophylaxis or PrEP in short. Sexual reproductive Health include services that promote good sexual health and reproduction. They include but not limited to family planning, cervical cancer screening, sexual transmitted infections, management and many more. Today, we will only discuss about family planning, diagnosis and management of STIs, cervical cancer screening and PrEP. We will look at each of this one by one. Let us start with STI services. Explain to me, what happens if the client is suspected or diagnosed with an STI here at your clinic?
    34. *R: okay. When a client’s usually they are being at bring here at our (Name of sponsor) VMC. Clients are being captured with STI during screening. These clients come here for the procedure, circumcision. So, after they have already tested HIV, then they go to the screening whether they are fit for procedure, where we are screening other problems including STIs. So when we found out that this client has STI, the procedure is being abandoned, until the client is healed … from this problem… we refer to treatment room, where we give treatment for the STI free of charge*
    35. I: okay, is the treatment room within the circumcision clinic or outside?
    36. *R: within our building but next door. Then, if that client has got a partner, we give the slip to bring the partner. When the partner also comes, is being tested and treated. But HIV testing is not forced, it’s voluntary.*
    37. I: okay. So as you as a health care provider, what is your opinion on this integration of STI services within VMMC services?

1. *R: it’s good and very important because it catch and we treat the clients who was at home, unknowingly that he is also affected by the partner. And the partner who have come for one service benefits from treatments.*
2. I: okay. So, what is it that you do really like about this integration of STI and VMC?

*R: sorry, may you come again.*

1. I: What is it that you like about this integration of STI and voluntary medical circumcision?
2. *R: yea, the part that I like most, the clients they come to seek other services, they also get treated for the hidden problems. So, to me, this is very good.*
3. I: okay. Is there anything that you would say, you don’t really like about this integration?
4. *R: Aaah… no*
5. I: okay. So, how do you think STI services should be offered at VMMC clinic? I know you are offering those services but is there any other opinion, this STI services could be offered.
6. *R: yea, they are offered but we need more terms and updates with new guidelines of course we have but it’s not to everybody. If more briefing on STIs can be added, I think it could be of higher standard, then produce quality.*
7. I: Okay, so what do you think are the barriers or concerns of this integration? Of STI to VMMC
8. *R: The only barrier is that, the same room that we use for VMC clients, is the same room we use for treatment, for those who have diagnosed with STIs, so we need to have another room to avoid interrupting your service. Should have another room for clients found with STIs, so that they should be treated.*
9. I: so are there anything to add to address these barriers?
10. *R: I think no… no, I have talked enough*
11. I: okay, now let us talk about family planning. Explain what happens if a client needs family planning services, whether vasectomy for males or … family planning service
12. *R: aaah we refer them to Bwaila district hospital because we are only targeting on VMMC cases but those with STIs, the benefit because we need to assist them but for other services, we are not offering.*
13. I: okay, so you as a healthy care provider, what is your opinion if family planning was integrated in VMMC services
14. *R: aaah I don’t know if there can be other projects can be added like family planning as another project, then it can be good but if it can be incorporated in the VMMC project, it can be good as well*
15. I:it can be good?
16. *R: because like having many services under one umbrella body*
17. I:is there anything that you would like of integration this integration of family planning and VMMC services?
18. *R: [laugh]… yes, of course that will be increasing more to the VMMC but it can be of benefit whereby both partners like a husband seeks services of circumcision, brings the wife, both can benefit as they can be encouraging one another like one trip, having so many services offered to them*
19. I: so, what do you think would not interest you in integrating family planning with VMMC?
20. *R: most men can start shunning away from accessing the services because they will know that once my neighbor has seen me coming for VMMC, then she already knows that circumcised since our culture its new to circumcision.so they may be shy, seeing the wife of the neighbor seeing that am circumcised.*
21. I: so if family planning has been integrated with VMMC, how should the service be offered here at VMMC clinic?
22. *R: that’s a good question. That this, am not saying it’s bad to integrate family planning with VMMC, its good but they should be at separate buildings despite that both can come at the same facility but at different rooms so that the women should go and access their services and these men should not be seen by these women when they go for their services*
23. I: okay, so what do you think are the barriers and concerns about this integration?
24. *R: I think the barriers are the ones which I have said that the men can be shy as of now, they are open enough, they are happy… of course we have a barrier that some men do not like to be circumcised by female providers, they shun away. But only those who have really needed the procedure, are the ones who go ahead.*
25. I: okay, so what should we do to address these concerns so that both male and spouses they benefit through VMMC while we are also taking care of what you have also said
26. *R: I think if we can do more sensitization campaigns, this will help so that we can impart knowledge from both parties so that they can know the benefits of integrating the service.*
27. I: okay, so let us now talk about cervical cancer screening. Explain what happens when a women need cervical cancer screening here at VMMC?
28. *R: aaah since we don’t offer, this service, its only VMMC, so we refer them where they can access cervical cancer screening*
29. I: okay, so you as a healthy care provider what is your opinion on integrating cervical cancer screening and VMMC?
30. *R: yea, it’s important because if we can be doing this because the aim, one of the aim, reason of doing vmmc, is to reduce cervical cancer. So if a husband brings his wife, and come for vmmc, can you check my wife for cancer, then we can screen many because now three thousand male circumcise, so if we screen, about that figure, at least we can catch many who have not reached advanced stage of cervical cancer.*
31. I: okay, what is it that would not interest you about this integration? If there is any
32. *R: aaah no, it will interest me, only that we will need to add more trainings either to the same staff or to add more staff, we can be doing that project.*
33. I: okay, what is it that you like of this integration if we happen to integrate?
34. *R: yea, I like this because we can reduce the advancement of cervical cancer because it will be identified at an early stage because it can easily be cured.*
35. I: okay, so imagine about this integration, how best should we offer services of such integration at VMMC
36. *R: as I have already said, we can encourage males to bring their partners. Boys who are not married but have got girlfriends, should also bring them. Any of age bearing girls or women, of child bearing age*
37. I: so where should we provide? Within the clinic or where?
38. *R: within clinic at a separate room that because also girls may shun away, run away that they are shy that they might be shy but we can offer at a separate room.*
39. I: what do you think will be the barriers of this integration of cervical cancer screening and VMMC?
40. *R: the barrier could be that this service is offered next door, then the males will not be interested, we will see a dropout of numbers coming for vmc because they will be feeling shy with girls or women. so if we can separate, either the whole or there could be demarcation, that these they cannot be seeing one another.*
41. I: okay fine. Now, let us talk or discuss about PrEP. Have you ever heard, about PrEP before?
42. *R: yes*
43. I: what have you heard about PrEP?
44. *R: Yea, PrEP, is the group of ARVS or type of ARVS…. Anti-retroviral therapy given to a person who have not yet exposed but is prone to be exposed to HIV virus either health sector or in one way or another*
45. I: okay now, if PrEP becomes available, what is your opinion if we can integrate PrEP with VMMC
46. *R: yea, PrEP will be good because it will give chances to both parties or people who might be exposed to HIV virus, yea, I accept it.*
47. I: okay, if PrEP becomes available, how do you think should be offered in VMMC?
48. *R: yea, I feel like should be offered to healthy workers who are going to operate on a client who has been found with HIV positive during testing of HIV if they are going to operate on that client, healthy workers should take PrEP because there are injuries or there might be injuries*
49. I: okay. PrEP and PEP, they are the two different things?
50. *R: yes*
51. I: PrEP is Pre-Exposure Prophylaxis, which mean someone take drug before they are infected. PEP it’s when they are exposed, so they are really not to get infected. So I was asking that if PrEP that drug which they get the negative ones, becomes available, how should be offered in the Vmmc or how should be integrated in the VMMC?
52. *R: I was answering that, PrEP because if you are going to work, you have to put on a shield or bullet proof.so PrEP, will act as a bulletproof because you are going to operate on the person who is infected.so you need to be protected, get a shield because anything can happen, you can get a bullet or not. So it’s the same, as you are going to operate on this patients, you may be injured or not*
53. I:so would you encourage your clients who come for vmmc to take PrEP? If it becomes available
54. *R: I would encourage if healthy worker is positive because they can also be infected*
55. I: okay. What about clients who just come for vmmc and they are negative, would you encourage those people to take PrEP?
56. *R: I think no, I would not encourage it because unless the provider is infected, so that’s when we can give clients. If that provider, is open enough, can give the client who he is going to operate on. But if he is negative, anyway, window period is there*
57. I: what about those clients who are at high risk?
58. *R: for high risk clients, they should take PrEP*
59. I: would you encourage your clients who come for VMMC to take PrEP?
60. *R: hmmm its 50;50 because for high risk, you don’t whether it is at window period, even if you get PrEP, it will not work because it is already exposed. But the client who have tested, are negative, but the high risk, yes, it will be good for them to avoid getting HIV virus.*
61. I:so how do you think PrEP should be offered at VMMC?
62. *R: I hope it should be offered by trained provider after HIV testing and tested HIV negative and is open enough to tell whether the high risk.*
63. I: okay. Do you think there are any barriers or concerns if we integrate PrEP with VMMC?
64. *R: yes, my concern is that we might develop blood resistance because if you are not infected you take the drug. When you will be infected, the drug is not in your body, the virus, might be resistance. if you start taking the dosage of ARVS it will not work properly because your body is already full of [laugh]the virus. But if research has already been done, proving that PrEP work as no drug resistance, then it’s good to take because the virus that will be coming in your body, it will find already drug there, so it will not be able to penetrate, fine and good.it will be good as well[laugh]*
65. I: this drug is taken really daily for it to be effective, so would you encourage people to be taking PrEP?
66. *R: It will depend because it may take two days, if you don’t have a reason taking drugs, you may not adhere to the instruction on how to take drugs, unless you have a reason on why you are taking a drug. So PrEP, it might be taken without any purpose so people will not be adherent. they cannot finish days but PEP, it gets finished because you know that you have exposed but if you are not being exposed, it’s difficult to finish the dosage.*
67. I: so what is your opinion on integrating PrEP with VMMC?
68. *R: yea, it should be integrated, I like that but only to the people who are vulnerable, not to everybody because to those who are at high risk, yes agree to that and not anyhow, there should be rules guiding on PrEP.*
69. I: OK. Thank you. So if you were given powers to choose and integrate services, in vmmc clinics, what services would you include which we have not discussed here?
70. *R: ehm, we have talked STIs, Family planning, cervical cancer, PrEP and part of PEP*
71. I: Of course yes
72. *R: [laughing] we can include ART clinic within VMMC*
73. I: would you explain the reasons why you choose ART services?
74. *R: because the clients, because we are testing, why are we testing because we want to find out whether our client is positive or not, if found positive, what’s next? We refer, why referring? We are saying holistic approach outlines holistically*
75. I: so how do you think this service should be offered within
76. *R: within our clinic as we do with STI. If we found someone with STI during screening, we treat, why not HIV if drugs could be available, we are providers, we can provide the drugs as wells as long as the trainings, updates and orientations are being offered as well*
77. I: okay.is there any other service that you would think of?
78. *R: [laughing] as of now, no. Maybe if I can be given time*
79. I: okay, thank you very much for taking your time to discuss with me today. Your answer will be very helpful in improving the health services delivery at voluntary medical male circumcision clinics but before we close, do you have anything to say?
80. *R: aaah am just happy that am one of the interviewers, what I have said here will add value on what my friends have already said. They might have already said the things that I have also said. But still more, if two, three people say the same, that thing it carries more weight. So I appreciate also to keep me as one of the interviewee*
81. I: thank you
82. *R: and you are most welcome. Next time you may also contact me, am readily available*
83. I: ok
84. *R: my phone numbers, I have already given to my friend*
85. I: ok again thank you so much for taking your time
86. *R: thank you.*

THE END.
