# Supplementary material for: Opinions on integrating couple counselling and female sexual reproductive health services into Voluntary Medical Male Circumcision services in Lilongwe, Malawi
Source: PLoS One. 2022 Sep 9;17(9):e0273627. doi: 10.1371/journal.pone.0273627 (PMC9462804; doi:10.1371/journal.pone.0273627)
Supplement: S2 File — (DOCX) [file pone.0273627.s002.docx]

**D 43 STUDY**

**Date of Interview: 29 June 2018**

**Type of Participant: Peer or Clinic Aid**

**Interview Number: D-43-0009**

**Interviewer: C.L.**

**Total Interview Time: 21 minutes 40 seconds**

**Interview Summary:** **(from summary sheet)**

| **SERVICE TO BE INTERGRATED** | **THOUGHTS ON INTERGRATION** |
| --- | --- |
| Couple HIV Testing and Counseling | Thinks most men do not know they can bring their partners for testing and counseling but he thinks the integration is a good idea only that men can have fears that their wives will learn of their promiscuity. |
| STI Services | Thinks it’s a good idea to integrate because both partners will get treatment if one is infected. Diagnosis should be done in HTC room. |
| Family Planning | The integration would help the next generation to know about family planning. A separate office should be dedicated for the service. |
| Cervical Cancer Screening | Thinks it’s a good idea. Partners should take advantage of the time men go for review at the VMMC to access the cervical cancer screening. |
| PrEP | Would encourage promiscuity. |
| Other Services | No idea |

**Remarks:**

**Participant was relaxed and had no trouble understanding the questions. He seemed reserved when the issue of PrEP came up and didn’t even want to comment much on it.**

1. I: Thank you for taking the time to talk with me today. I would like to ask you some questions today about the way you feel and what you think about some issues related to the service you provide and how we can include other services in Voluntary Medical Male Circumcision clinics.
2. *R: Okay.*
3. I: There is no right or wrong answer to these questions. We would like to hear your opinion and your experiences in your own words. Do you have any questions before we begin?
4. *R: Ah, no. You can start.*
5. I: Okay. What role do you play in the provision of Voluntary Male Medical Circumcision?
6. *R: The services that we get at the VMMC clinic is a good one. Like for me the time that I came to be circumcised, it was in 2012 because I was having problems when having sex with my wife. When you are getting older the foreskin bruises when you are having sex and for you to have sex again the next day you think twice. But after you have done circumcision nothing like that happens and you enjoy married life.*
7. I: Mm… do you talk to your friends about Voluntary Male Medical Circumcision?
8. *R: Yes. Since I have ever done VMMC then when we are in a group and one of us complains about his foreskin and sex life I tell him “Go for Voluntary Male Medical Circumcision”. But others especially of the Chewa tribe usually say “Why should I do that?” But I encourage them “Think about how you feel when you are having sex with your wife, what happens?” and they say “I always have bruises and I wonder why that happens. Sometimes I think maybe it’s because she has just finished her menstruation”. So I tell them “No, it’s not that. It’s because your foreskin has grown old so it needs to be removed.”*
9. I: Okay, so how many people have you motivated to come for VMMC?
10. *R: So far I have motivated five who have come for VMMC at (Name of hospital) including my three sons.*
11. I: Okay so give a discussion that you had with one of these people to the point that he was convinced to come for VMMC.
12. *R: [Name withheld]*
13. I: How did you motivate him?
14. *R: He told me about the challenge that he was having which was the same one that I was having. So when I told him to come to Bwaila for VMMC he came and six or seven weeks later he came to say “You have really helped me. Everything that you said was true and now things are okay with me.”*
15. I: Okay. Have you ever seen a man bring his partner to the VMMC clinic?
16. *R: Ah… the ones I used to see were women with their children and not with their husbands but that was in 2012. But mostly men are encouraged to go for VMMC by their wives because when women are talking they tell each other how things go with their husbands and some can envy and wish their husbands were like that.*
17. I: Mh…
18. *R: So most men that come here it’s because they have been motivated by their wives.*
19. I: Okay. But you have never seen anyone come with his wife?
20. *R: No, I have never seen that.*
21. I: Alright I wanted to talk about couple testing and counseling at VMMC clinics. What do you think makes men fail to bring their partners to the VMMC clinic?
22. *R: Most men…ah… when coming to the VMMC clinic, most men do not know that they can also bring their wives. Most of the men refuse when they are told to test for HIV which shows that they don’t have the confidence in themselves to find out their status. But when they are coming for VMMC they don’t have any idea about the whole process.*
23. I: Mmm.
24. *R: Yes.*
25. I: That means they don’t know. So what can be done so that the men can be bringing their partners to the VMMC clinic?
26. *R: There should be sensitizations telling men that when coming to the VMMC clinic they should be bringing their wives along.*
27. I: Mm, so if you knew that you could bring your wife at the VMMC clinic at the time when you came for VMMC, would you have come with your wife?
28. *R: We could have come together only that at the time that I came here she was at work. So usually I could drop my kids to school then take them home after they knocked off. And that day was a Friday so I just dropped the kids at school then went straight here. Otherwise there is nothing wrong with taking my wife here.*
29. I: So what is your opinion about men bringing their partners to the VMMC clinic for couple testing and counseling?
30. *R: I think there is nothing wrong with that. [phone ringing]*
31. I: What is your opinion about integrating couple testing and counseling into VMMC clinic?
32. *R: Most men have fears because they know they lead risky life styles and for their wives to know their status, they are not happy.*
33. I: What makes them afraid?
34. *R: It’s what I have said that they know how promiscuous they are.*
35. I: So what can be done to address such fears so that the men bring their partners to the clinic?
36. *R: They need to be sensitized.*
37. I: What message should they be given?
38. *R: They should be told that when coming to the clinic they should come with their wives, that there will be testing and counseling not only for the man but for both.*
39. I: Mm... Alright. Now I would like to discuss with you about sexual reproductive health services and Pills for HIV prevention: called pre-exposure prophylaxis. (PrEP)
40. *R: Okay*
41. I: Sexual reproductive health includes services that promote good sexual health and reproduction. They include but not limited to family planning, cervical cancer screening sexual transmitted infection (STI) management, and many more. Today we will only discuss about family planning, diagnosis and management of STIs, Cervical cancer screening, and PrEP…We will look at each of these one by one. Let us start with STI diagnosis and management.
42. *R: [Clears throat]*
43. I: What happens when a man is suspected or diagnosed with STIs at the VMMC clinic?
44. *R: If they diagnose someone with an STI they are not supposed to conduct the circumcision procedure on him.*
45. I: They are not supposed to circumcise him?
46. *R: No, until he gets healed because if they do the procedure on him the infection can cause other serious infections and in the end people will start saying medical male circumcision is bad.*
47. I: Mmm… so as a peer what is your opinion about integrating STI diagnosis and management into VMMC clinic?
48. *R: it’s very important because then both of them will be aware of the situation. Sometimes one partner can have an STI while the other not. So if it’s known for the one that is infected then both of you get the treatment.*
49. I: Mh…
50. *R: And that will do well for both of you.*
51. I: Okay, but what concerns and barriers can there be for this type of integration?
52. *R: The challenge is that you women have no problems telling men about things but when a man tries to tell you something, it becomes an issue “Where did you hear that from, who were you with…” and so on, [chuckling]*
53. I: Mh…
54. *R: So that’s the difference between men and women. You women you can tell men anything and men won’t get irritated wit it but for a man to tell you the same thing, it irritates you.*
55. I: So that means if a man tells his wife to say “My wife let’s go to the VMMC clinic so we can be screened for STIs”, the wife can…
56. *R: She can be irritated.*
57. I: Okay, so how can we address such a concern since what the integration is about is for men to be coming with their partners to get screened?
58. *R: The best way to deal with it is for the men to be telling their wives explicitly what they will be doing at the VMMC clinic so that she knows what she will be doing there instead of hiding these things.*
59. I: Mmm. So what do you think is the best way to offer STI services at the Voluntary Medical Male Circumcision clinics?
60. *R: As I said earlier on that there needs to be civic education so that the people should know before they get here. This is so because if they are already here and you tell them about this integration then they can just tell you “let me visit the gents first” and can never come back.*
61. I: Okay, so let’s say the sensitization has been conducted and people know about the integration. Where can this STI diagnosis and treatment be conducted?
62. *R: In the HTC room.*
63. I: In the HTC room?
64. *R: Yes, first station.*
65. I: Right in the VMMC clinic?
66. *R: Yes, but they should be doing it at the first station, the HTC room.*
67. I: Who should be conducting the screening for STIs? Should it be the same person who does HIV testing and counseling?
68. *R: No there should be two in the room so that one does the HIV testing and counseling and the other diagnosis for STIs.*
69. I: Okay. Who should access this STI service? Should it just be anyone or just those coming for VMMC?
70. *R: Everyone who needs the service.*
71. I: What do you think are the barriers and concerns on this integration of STI services with Voluntary Medical Male circumcision services?
72. *R: There cannot be any barriers or concerns because people need the service.*
73. I: Okay, now let’s talk about family planning. Have you ever heard anything about family planning?
74. *R: Yes. I know that it prevents unwanted pregnancies.*
75. I: Okay, so in your opinion, what happens when a couple wants family planning service at the VMMC? When we are talking about family planning we mean for both men and women.
76. *R: There are different types of family planning; one is when you need space between two births while the other is when you feel your children are enough. So I don’t know which one you want.*
77. I: Okay, so I am actually talking about both. Maybe you have ever heard other men at the VMMC clinic talking about family planning for their wives. So what happens when a man needs family planning service?
78. *R: I have never heard anyone talk about that so I can’t say how they are assisted.*
79. I: Mmm
80. *R: Like for me it’s my wife who got Tubal Ligation and that’s the type of family planning that we do.*
81. I: As a peer what is your opinion on integrating Family planning in circumcision services
82. *R: Yea that would help the next generation to know what they are doing and they would know that if we do this then the end result is that. Right now they just come here and go without knowing anything about family planning.*

I: So how do you think family planning services can be offered within Voluntary Medical Male circumcision clinics?

1. *R: I think if you spared a separate office where you can be offering the family planning service.*
2. I: Okay, when can the family planning service be offered?
3. *R: Before circumcision.*
4. I: Before circumcision?
5. *R: Yes, you can include it before the procedure because when they are done with the procedure they cannot come for the service…*
6. I: Mh…
7. *R: … you should include it before at check-up, because the clients come for reviews so they can be offered family planning before the reviews.*
8. I: Okay so who should be able to access these services? Should it just be a couple that comes for VMMC or who?
9. *R: Those that have come for VMMC because let’s say a man alone for VMMC, so you can tell him that when coming to review he should come with his partner.*
10. I: What would be the barriers or concerns for this integration?
11. *R: There cannot be any barriers or concerns because with the way things are with this generation, it’s just supposed to be like that.*
12. I: Mh…
13. *R: It was hard in the late 80s or early 90s because people were saying “Are they saying I should only have three children? No!” But now things have changed.*
14. I: Now let’s talk about screening for cervical cancer. Tell me anything you know about screening for cervical cancer.
15. *R: I have ever heard about it but I don’t really know what it is.*
16. I: Okay, cervical cancer is a type of cancer that attacks the cervix of women and some clinics screen women to check if they are at risk of having it or not.
17. *R: Mmm*
18. I: Have you ever heard people talk about it at the VMMC clinic?
19. *R: Aah no, I have never heard about it.*
20. I: Okay so as I said some clinics screen women to check if they are at risk or if they have it and if the diagnosis is early enough they can stop it from spreading. So what is your opinion on integrating cervical cancer screening in Voluntary Medical Male Circumcision services?
21. *R: Cancer is now a big threat maybe because of the food that we eat. So we need to have cancer clinics everywhere to assist people. Otherwise people will just be dying.*
22. I: Mmm okay. What is it that you do not like about this integration?
23. *R: There is nothing I don’t like about it. There cannot be any concerns because that means you will spare another office for the cervical cancer screening.*
24. I: You said they need to spare a separate office for cervical cancer screening. What else can be done to better offer this service?
25. *R: When you spare an office then you can be conducting all the procedure there and you can decide what kind of treatment you can give to someone who is found to have the disease.*
26. I: Who should be screened?
27. *R: Cervical cancer attacks women…*
28. I: Mmm
29. *R: And women don’t go for VMMC [laughing]*
30. I: Yes, that’s where we want to know how they can be coming.
31. *R: The issue is like I have said that they need to come together. Like for STI diagnosis we said they should come together for review so that the woman can take advantage of the man going for review and she goes for cervical cancer screening.*
32. I: Should the screening be happening while the man is there?
33. *R: They can talk about it while they are together then privately they can screen the woman.*
34. I: If it’s your wife being screened would you feel shy to be there while she is being screened?
35. *R: No not that I would be ashamed but just giving her some respect*
36. I: What do you think are the barriers and concerns on this integration?
37. *R: I don’t think there can be any barriers or concerns unless the person isn’t aware of what is happening. How do we go to the labor ward?*
38. I: Mh…
39. *R: We go to the labor ward and watch while your baby is being born.*
40. I: Have you ever gone to the labor ward?
41. *R: Yes, I have, my last born child [name withheld], I was there when he was born.*
42. I: Mmm. You have said earlier that men need to respect their wives by not being there when they are being screened and now…
43. *R: Yes, I was saying that for the sake of those people that are uncivilized because we have people who are civilized while others are not. So those that are not civilized want more privacy while the one who is civilized feels that it’s his wife so there is no problem.*
44. I: Okay, so it should be a choice whether one wants to be there during the cervical cancer screening or not?
45. *R: Yes.*
46. I: Okay. Have you ever heard anything about PrEP?
47. *R: No, you tell me.*
48. I: PrEP is anti-HIV medicine that keeps HIV-negative people from being infected.
49. *R: Mmm*
50. I: There is a single pill that is taken once daily, so it’s like you take one pill today and take another tomorrow and if you take it regularly, it is highly effective at prevention people from being infected.
51. *R: [silence]*
52. I: So if you are to tell someone what PrEP is, what can you tell them?
53. *R: Don’t you think that is encouraging people to be promiscuous?*
54. I: [laughing] Okay that means you have concerns, what are your concerns?
55. *R: Ah, no that is encouraging promiscuity.*
56. I: Say your concerns.
57. *R: No, it’s not a concern but you will be encouraging people to be having unprotected sex anyhow. When they are at parties they will just say “I am taking PrEP so I can do whatever I want “.*
58. I: If PrEP was available, do you think it’s necessary for it to be made accessible to HIV-negative men and women?
59. *R: Yea it can be made accessible to them but that’s encouraging bad behavior.*
60. I: It’s encouraging bad behavior…?
61. *R: Yes, and I don’t like the drug.*
62. I: You don’t like it?
63. *R: No.*
64. I: Would you encourage a friend to take the drug?
65. *R: No he needs to protect himself and not rely on that drug.*
66. I: Why can’t you encourage your friend to take it?
67. *R: It’s because if I encourage him to take the drug then it will be a habit and if the drug is no longer available and he gets infected then it will be bad.*
68. I: Okay. Now let’s talk about other services. We have talked about PrEP, couple testing and counseling, STI services, family planning and screening for cervical cancer. What other services would you choose to be integrated with VMMC if you were given the powers to choose?
69. *R: No, I was thinking that for medical circumcision to work well you need to encourage men to come for medical circumcision when their wives have just given birth. Let’s say a woman is pregnant and has gone into labor, that’s the right time for the man to go for VMMC so that at six weeks both of them can be ready to have sex. There might be some problems when a man gets medical circumcision at a time when the woman is okay because the first week the woman can understand that you have a wound but the next two or three weeks she can take it no more even if you play with her, she cannot be satisfied and can look for sex elsewhere. That is why most people have sex at four weeks*
70. I: Mmm, so what other services do you think can be integrated with VMMC service?
71. *R: I have no idea.*
72. I: You have no idea?
73. *R: No, maybe if I could come back then I can be able to think and answer.*
74. I: Alright. Thank you for taking your time to discuss with me today. Your answers will be very helpful in improving the health service delivery at Voluntary Medical Male circumcision clinics.
75. *R: Mm.*
76. I: Maybe you have something to say before we close?
77. *R: Ah, what I wanted to say is what I have already said that when a woman is going into labor then the man should take that opportunity to go for VMMC. That was things will be better because each of them will be waiting for the partner to get better.*
78. I: Alright thank you for your time

END

**D 43 STUDY**

**Date of Interview: 18 August 2018**

**Type of Participant: Peer or Clinic Aid**

**Interview Number: D-43-0014**

**Interviewer: I.N.**

**Total Interview Time: 46 minutes 55 seconds**

**Interview Summary:(from summary sheet)**

| **SERVICE TO BE INTERGRATED** | **THOUGHTS ON INTERGRATION** |
| --- | --- |
| Couple HIV Testing and Counseling | Integration is good but can make some men uncomfortable to come to the VMMC clinic |
| STI Services | Thinks STI services would help to reduce the number of infections. |
| Family Planning | Providers will be able to clear myths and misconceptions about family planning. |
| Cervical Cancer Screening | Feels cervical cancer should not be integrated with VMMC |
| PrEP | Not a good idea to integrate with VMMC because these are two different services. |
| Other Services | Thinks people need sensitization. |

**Remarks:**

**Participant was very open-minded. However, he started contradicting himself and started saying contrary to what he said on the onset.**

**Interview Text:**

1. I: You are most welcome here, and thank you for taking the time to talk with me today.
2. *R: Thank you.*
3. I: I would like to ask you some questions today about the way you feel and what you think about some issues related to the service you receive here and how we can include other services in Voluntary Medical Male Circumcision (VMMC) clinics.
4. *R:Mh…*
5. I: There is no right or wrong answers to these questions. We would like to hear your opinion and your experiences in your own words. Do you have any questions before we begin?
6. *R: Yes, I have a question.*
7. I: Go ahead.
8. *R: Is it right for a man to come with his partner at the VMMC?*
9. I: Well, that’s one of the things that we are going to discuss today, and of course that’s one of the questions that it is you who are supposed to answer.
10. *R: Ok.*
11. I: Alright. What role do you take in Voluntary Male Medical Circumcision services?
12. *R: I encourage them about VMMC and also how important VMMC is especially in the prevention of STIs and cervical cancer which is affecting a lot of people in this country.*
13. I: Are you circumcised yourself?
14. *R: Yes.*
15. *I*: When were you circumcised?
16. *R: In 2001.*
17. I: Ok.
18. *R: Sure.*
19. I: So how in particular do you encourage your peers? How do you work with Bwaila Health Centre?
20. *R: This is what we do; I work with my colleague and we do go in villages, and find boys, say, they are playing football, and then we ask them to come, and we mobilize them, and then we explain to them on the importance of VMMC such as the prevention of STIs, HIV in particular, and also to prevent cervical cancer which is affecting the lives of many women and this helps. So when a man is circumcised, women are in a good position to prevent contracting cervical cancer.*
21. I: So when they are convinced, do you bring them here?
22. *R: Our policy is that when a boy is convinced that he will participate, he is supposed to give us their phone numbers. Besides, we are supposed to communicate with their parents before they can come here. The parents also need to discuss as a family, women need to tell their husbands on the development, and if both the parents and the boy agrees, we make a phone call and send our vehicle to come and get the boy and assist him accordingly. When he is circumcised, we also bring him back to his home and keep on visiting him for the next 3 days.*
23. I: Ok!
24. *R: Yes, we come to check on him, and for three days, we visit him so as to loosen the bandages that were stuck on him and also to remind him on the counsel that he was told when he came at the VMMC; that they are supposed to cut a bottle and put salt water therein and dip their organ there for some 15 minutes, after which they can take it off from the bottle, and they need to repeat the process in the evening. When they stop feeling pain, it means that the wound has healed, and we don’t even remove the stitch, it falls down on its own.*
25. I: Mm… Ok!
26. *R: Yes.*
27. I: So can you give me an example of a day when you talked to a friend about VMMC and how he responded? What you actually talked about.
28. *R: I talked to someone, but he is married. He says when he compares the level of cleanliness back then before he came for VMMC and after he came, he says it is healthier now than before because before he got circumcised, he could produce a certain smell after sex especially before he could go for shower, but nowadays, that problem is no longer there.*
29. I: Mm, okay!
30. *R: Yes.*
31. I: Alright. Let’s now talk about couple testing and counseling. Tell me what happens when a man brings his partner to the VMMC clinic? Have you ever seen a man coming with his partner at VMMC?
32. *R: So many men come with partners.*
33. I: So what happens when they come up with their partners?
34. *R: When a man comes with his partner…. we actually have our rules here, for he doesn’t just come, but meets a doctor who screens HIV. So when the man has come with a partner, and the partner is interested to go for HIV test, we tell them both that they can go for testing as a couple.*
35. I: Ok.
36. *R: Yes.*
37. I: For those that bring their partners here what do you think motivates them to do so?
38. *R: I think it’s because of the prevention of serious STIs like the ones that I mentioned, especially cervical cancer. So, most people encourage their wives to come for that because of STIs.*
39. I: Alright. So have you ever seen other men who do not come with their partners at the VMMC?
40. *R: There are men who don’t love their marriages and they do go at the clinic alone without going with their partners.*
41. I: Mm, what do you think is the reason such men don’t bring their partners at the VMMC clinic?
42. *R: I think such men have fears of being tested positive because for a man who has no doubts about his status cannot fail to take along with them their wives at the VCT for HIV testing.*
43. I: Apart from suspecting that they can be tested positive, is there any reason that you think can prevent men from coming with their wives at the VMMC?
44. *R: Yes, there can be another reason. There are cases where a man can be married for years and can have older children who knows what is happening and in such cases, they can be reluctant to go for VMMC because they can feel ashamed since their little ones can notice that they have been circumcised. So they can just hide it from their wives so that they should not know.*
45. I: Mh, ok!
46. *R: Yes. Those are some of the reasons that can affect others.*
47. I: So what do you think needs to be done so that men can be coming along with their wives for HIV testing as a couple?
48. *R: Well, what happens is that when a person comes at the clinic, we ask him if he has a wife, and when they say that they have a wife, we ask them if it is possible for them to come with the wife. If they say that the wife cannot come, we tell them that it is ok since they are already at the VMMC, and we tell them that we will assist the accordingly, but after we help them we tell them that they should inform their wives so that they should go to any nearest health centre for HIV testing.*
49. I: Ok!
50. *R: Sure. In addition to that, when someone is tested HIV positive, he is never circumcised. We ask them to leave.*
51. I: Why are they not allowed to get circumcised?
52. *R: They are asked to leave especially when their immunity is very low. So they are asked to meet a doctor who can help them with the medicines that can help them to regain their immunity. So they are given a time frame and are told by what month they are supposed to come at the VMMC. There are several boys in the villages whom we have left aside.*
53. I: Really?
54. *R: Yes.*
55. I: So, you, as someone who encourages men to get circumcised, what is your opinion on integrating couple HIV counseling with Voluntary Medical Male circumcision services?
56. *R: Well, it becomes difficult especially for older men who are married. They feel uncomfortable to come here at the VMMC. But we still do encourage them so that they should come here and undergo the process. Some just do feel ashamed because they have older daughters, and when they see him, for we do come with them as they are coming from the clinic, and so if they see that their dad is getting off from the car, they become surprised: “Where is Dad going?” Our vehicle is very famous, you see, it has posters all over it indicating that it is for VMMC, and so fathers are afraid of that since they know that their children are grown ups and they will notice that their father has been circumcised. You see?*

*[BOTH LAUGHING]*

1. I: Ok!
2. *R: Sure.*
3. I: But do you personally believe that it is possible to integrate couple HIV counseling with Voluntary Medical Male circumcision services?
4. *R: Yes, it is possible.*
5. I: How can this be possible?
6. *R: It can be possible because it can help in other institutions as well where people could have gone for HIV testing, so it will be like we are on the other hand assisting, doing both things at once.*
7. I: Mh?
8. *R: Yes.*
9. I: Mm... What do you think are the barriers and concerns on this integration?
10. *R: I cannot clearly know the barrier, because there are several organizations and so I cannot tell what help they can provide, maybe they cannot want that arrangement of allowing a couple to come for HIV testing, depending on the way they do their job since several organizations come…*
11. I: Several organizations come at the VMMC?
12. *R: Yes.*
13. I: Really?
14. *R: Yes.*
15. I: They are not from a single organization?
16. *R: No.*
17. I: Ok.
18. *R: Sure.*
19. I: So what you are saying here is that there can come an organization that can refuse to do that?
20. *R: Yes, they can refuse. For instance, the organization that we now have refuses boys who are 9 years and less. They want those who are 10 years and above. Those ones can be circumcised. But those who are 9 years and below are rejected.*

*It also happens that there are other organizations who assist those who are 9 years old, even those who are 8 years. But for those ones to assist such young ones, they advise that the boys should come with their parents.*

1. I: Mh...
2. *R: Yes.*

I: Ok. What do you think should be done to overcome these concerns and barriers to couple counseling in Voluntary Medical Male circumcision clinic since there are different organizations that come here, some accept, others refuse? What can we do to ensure this disagreement isn’t there?

1. *R: To overcome such barriers, we need to sensitize them the way we tell them about HIV, and they should be able to know what to do in their families. We need to ask them the way we screen cervical cancer and other small STIs, we really need to tell them to test for everything.*
2. I: Ok, alright. Thank you very much.
3. *R: Okay.*
4. I: Now I would like us to discuss about sexual reproductive health services and Pills for HIV prevention: called pre-exposure prophylaxis. (PrEP)Sexual reproductive health include services that promote good sexual health and reproduction. They include but not limited to family planning, cervical cancer screening sexual transmitted infection management cervical, condom distribution and many more. Today we will only discuss family planning, Sexual transmitted infection management cervical screening and PrEP. We will look at each of these one by one. Let us start with STI services.
5. *R: Ok.*
6. I: Of course that means that we are excluding HIV ….
7. *R: Yeah, these other small STIs…*
8. I: Yes.
9. *R: Ok.*
10. I: Tell me what happens when a person is suspected or diagnosed with STIs?
11. *R: If a person is suspected or diagnosed with, say, syphilis, he is sent back home because we can’t circumcise such a person…. or we firstly give him treatment so the infection can go away then he can come back for VMMC.*
12. I: If someone has syphilis, do you circumcise him on the same day, or?
13. *Ok, we just give him treatment in form of drugs and after he completes the dosage, we ask him to come back for VMMC.*
14. I: Why do you send back or firstly treat the person with syphilis or any STI before VMMC, why don’t you just circumcise him on the same day?
15. R: Our aim is to deal with the infection first before circumcision because if we circumcise the person before we treat him, the infection will still be there and in addition to that, the wound will take long to heal.
16. I: Alright.
17. *R: Sure.*
18. I: As a peer, what is your opinion on integrating STI services with Voluntary Medical Male Circumcision services? That is, a couple can come, not only for VMMC, it can even mean girls coming, what’s your opinion?
19. *R: I think that’s a welcome idea so we can be helping both sides.*
20. I: Why do you say so?
21. *R: Am saying so because if we do not take that initiative, we will not reduce the spread of the STIs, they will keep on spreading.*
22. I: Mh. What is it that you like about this integration?
    1. *R: I like the integration because it will help reduce the spread of STIs, and also men and women will have health lives.*
23. I: Ok.
24. *R: Yes.*
25. I: How about what you don’t like about the integration – for each story has both sides, advantages and disadvantages?
26. *R: I view it positively.*
27. I: Ok.
28. *R: Yes.*
29. I: So how do you think is the best way to offer STI services at the Voluntary Medical Male Circumcision clinics? At what time point and where should the service be offered?
30. *R: Before men are circumcised, they are sensitized by girls like you in a certain room. When we are sensitizing them, we explain to them about the processes that take place here including where they will go after the sensitization process is over. We tell them that they will be tested for HIV and even those who have other diseases like heart problems, we also ask them to explain at that point in time so that we should help them first before they are circumcised.*
31. I: So you feel this is the best time?
32. *R: Yes, because sensitization can take up to 1 hour and 30 minutes.*
33. I: How about the fact that there will also be the presence of girls, and some can

bring their wives, don’t you think this can embarrass them?

1. *R: No, they never get embarrassed!*
2. I: So what do you think is the best room that they can be screening the STIs?
3. *R: We go in the hospital building, there is a special room that is used to screen, we do not do the screening right here at the VMMC. VMMC is separate from the screening department and those who do the screening are people different from those at the VMMC.*
4. I: Ok.
5. *R: Yes.*
6. I: So you prefer they should be screened right there as it happens, but that there should be a separate room for that?
7. *R: Right at the VMMC?*
8. I: Yes.
9. *R: No, they should keep on doing the screening at the place that they screen!*
10. I: Ok.
11. *R: Sure.*
12. I: Mm. So what am saying is, much as we know that there are other services like HIV screening at the VMMC, but then, how can it be like if there can be a hospital with a sub clinic that will concentrate on the STIs so that even those people who do not come for VMMC can as well have an opportunity to attend. So my question is, should the screening be the way things are like at the moment, or there should be a special way of doing it?
13. *R: I think there should be a special way because those who come for VMMC are seasonal patients who come for a period and then they disappear after some time, while those who have STIs cannot be waiting for the VMMC to be working for them to be assisted, they need to be assisted urgently and we really need to ask for support on that so that the providers should be available at the place yearly, but not just coming for a few months, say, 6 months, then off they leave, no! Sometimes they skip years, they can come this year, but not come the following year. It should note happening like that. They should be available full time the way the hospital works – all the times!*
14. I: Who exactly do you think needs to go and receive the services in such a clinic? Do you think there are some who are not supposed to be there to have access to STIs’ treatment?
15. *R: I think it should not be just everyone, it should be those who are like 18 years and above.*
16. I: Why have you chosen those who are 18 years and above?
17. *R: Those are the ages who know everything that happens in the body of a person.*
18. I: Ok.
19. *R: Sure.*
20. I: What do you think are the barriers and concerns on this integration of STI services with Voluntary Medical Male circumcision services?
21. *R: [SILENT]*
22. I: You well know the VMMC Clinic?
23. *R: Yes.*
24. I: So what do you think can be the concerns if we integrate STI services with VMMC?
25. *R: There can be a concern. Those people come when there are VMMC staff, which means if the providers are gone, it can then be difficult because those who come to seek help can come and can find no one at the clinic and that’s what worries me. So that’s why I mentioned at a certain point in time that we should be assisted even if the VMMC providers are not there.*
26. I: Ok?
27. *R: Yes.*
28. I: Any other concern?
29. *R: Yes, there are some women who don’t encourage their husbands to go for VMMC. Some men need to be forced to do VMMC. They need to be told the reason of going VMMC. Women are in a good position to do that if they want to have a bright future.*
30. I: Ok!
31. *R: Yes.*
32. I: Okay. Now I want us to talk about family planning. Tell me what happens when a person comes to the VMMC clinic to access family planning? Has this ever happened?
33. *R: Male or female?*
34. I: Either sex, it can be male or female.
35. *R: Do men also do family planning?*
36. I: Vasectomy, ever heard of it?
37. *R: Yes, but don’t fully understand what actually happens.*
38. I: And so you have never heard anyone at the VMMC Clinic talking about family planning?
39. *R: Yes, mention.*
40. I: What actually do they say?
41. *R: They only talk about using condoms. We of course hear that there is another family planning method for men, but we have not yet known what exactly it is.*
42. I: You don’t know it?
43. *R: No.*
44. I: Alright. There are different family planning methods for women. There is also a family planning method for med known as vasectomy, so a man never impregnates a woman. But for women, they use pills, depo, implant. So what is your opinion on integrating Family Planning services in VMMC?
45. *R: I think the methods can be good, but about family planning itself, I think it depends on how you as a family has agreed on what to do, depending on how you see things, it’s a personal issue.*
46. I: Ok, so what do you think is the advantage of integrating Family Planning services in VMMC?
47. *R: I think families can be going on well so that a family cannot have so many children while they don’t have resources to take care of the children.*
48. I: What is it that you would not like about integrating Family Planning services in VMMC?
49. *R: I think I would not like the fact that these providers are here for a short time and they give a misconception to people that the VMMC offer the family planning services, and so when the providers are gone, people will have nowhere to do that. That is the disadvantage since the VMMC will come integrating both services and once they are gone, that will be very adverse and it will be a draw back to the people.*
50. I: Okay. What do you think is the best way to provide the family planning services at the VMMC clinic? When and where should these services be provided?
51. *R: These same VMMC people?*
52. I: Yes, in case they provide family planning services, at what time should they be offering such services?
53. *R: Sorry to ask, but you mean when a man and a woman comes?*
54. I: Yes, in case you have come at the VMMC Clinic, and you want to access family planning services, where can you prefer to get such services? Should they be provided by those who welcome you, or those who do surgery, screening? What should be happening?
55. *R: I think we should meet the one in the hospital whose department concentrates on family planning only.*
56. I: So should the doctor be from VMMC or should the doctor be someone different who does provide other specialist services in a particular department?
57. *R: It should be a separate specialist doctor in a particular department.*
58. I: Ok.
59. *R: Sure. He should not take part in the VMMC, those of the VMMC are supposed to be different personnel.*
60. I: How do you think is the best way to offer family planning services within Voluntary Medical Male circumcision services clinics in case we have integrated the two?
61. *R: I think what can be done is that for those who want to do family planning, they need to go as a family at the clinic, because the first step is that a couple needs to agree first because it is not possible for this just to happen by coincidence, a couple needs to agree on the matter of family planning first. If you reach a consensus, you can then go for a family planning method of your choice if the family planning doctors are available at the VMMC.*
62. I: Ok.
63. *R: Sure.*
64. I: Mm... So which room should those offering family planning be?
65. *R: Those for family planning should be in their own separate room, and also those for VMMC should be in their own separate room as well, because there are totally different services.*
66. I: Okay. What do you think could be the barriers or concerns on family planning and Voluntary Medical Male circumcision integration because you have a couple of times said that family planning and VMMC are different things?
67. *R: Well, the reason I think we cannot combine the two is that VMMC deals with minimizing these minor STIs, and also it shows that you are civilized people, while family planning is something that you as a couple agrees on. But for VMMC, it is something that you just need to so that you should avoid contracting STIs, but for family planning, a couple agrees on which ear to do family planning.*
68. I: Do you think it is good to integrate the two?
69. *R: I think that cannot be good.*
70. I: Ok?
71. *R: Yes.*
72. I: Okay. Now let’s talk about cervical cancer screening. Tell me what happens when someone wants to get cervical cancer screening?
73. *R: I have never come across such cases. I just have ever heard about it only. If a woman is interested to be screened of cervical cancer, she just decides to do so.*
74. I: Ok, but how about at the VMMC Clinic, do you see such cases?
75. *R: Of course people talk about it especially on the transmission of cervical cancer only.*
76. I: As a peer, what is your opinion on integrating partner cervical cancer screening with Voluntary Medical Male circumcision services
77. *R: That cannot be a good thing because there can be different things because those doing cervical cancer screening will be different from those doing VMMC.*
78. I: So don’t you think it is possible to integrate the two?
79. *R: No, I don’t think so. For example, a doctor who deals with TB concentrates on TB only, those who deals with surgeries do surgeries only, that’s so totally different.*
80. I: Have you ever gone with your partner at the VMMC Clinic?
81. *R: No, but I have ever gone with her at the VCT.*
82. I: You went to VCT?
83. *R: Yes.*
84. I: Ok, so let’s assume that you want to go with your partners at the VMMC Clinic for HIV screening, can you go with her?
85. *R: I can’t go along with her.*
86. I: Why?
87. *R: It’s because at VMMC Clinic they concentrate on circumcision so I can just go where I know I can find VCT services.*
88. I: Ok I asked that question because earlier on, you said that you encourage men to come with their partners.
89. *R: Well, it is true that when a man comes, he goes to VCT, but it is not intentional, no. it’s the VMMC Clinic that requires a man to be tested first for HIV before circumcision so that we should his level of immunity first before we start circumcising him. So, if the man wishes, he can go along with his wife then.*
90. I: So are both of them tested?
91. *R: Am not quite sure, all I know is that the one who is to be circumcised is the one that is tested. That is why I said that the doctor who conducts the VMMC cannot do the STIs’ screening, they only test those who come for VMMC.*
92. I: The question was about having a specialist, say, in screening STIs, and bring him at the VMMC Clinic, and also a specialist in VMMC placed in the VMMC Clinic so that when the man is coming for VMMC Services, his wife should also be screened of cervical cancer, for instance. Do you understand that?
93. *R: Yes.*
94. I: So I don’t necessarily mean here that we should discharge the duties of a doctor who conducts the VMMC so that he should do the other services, no, that’s not what we mean here. Your answer should concentrate on adding extra services in the VMMC Clinic. We are not discharging anyone of his duties, we are just talking about adding an extra service right within the VMMC Clinic.
95. *R: It can all depend on what the organizers can prefer, or the way we can ask them. So it can be up to them to accept, or to deny about these other extra services.*
96. I: Do you think integrating cervical cancer services with VMMC can be good, and if good, how so?
97. *R: As I earlier on said, there is no advantage because these providers come and go and people will lack their services once they are gone*.
98. I: Ok.
99. *R: Sure.*
100. I: Okay. Now let us talk about PrEP. Have you ever heard about PrEP?
101. *R: If I am not mistaken, I heard that they are medicines that one can take after having unsafe sex with a woman…. they say, you take the medicines for a period of some 14 days or more, something like that. SO once you complete the dose, you are never found positive.*
102. I: Oh okay. But the one that you have explained is PEP, the medicines that someone takes when he/she had unsafe sex with a positive person. But here, we are talking about PrEP. They are a bit similar, but they work differently. So PrEP is an anti-HIV drug which helps to keep HIV-negative people negative. There is a pill that one needs to take every day to prevent HIV infection. It works if one is HIV negative and you want to prevent contracting HIV. It’s different from PEP, PEP is taken when you have unsafe sex and you are afraid of contracting HIV. But for these ones, we just take for a long period, and if you are following the procedures, you can’t get infected. Now with this explanation, how do you feel about PrEP? What’s your view on the medicines?
103. *R: I think they are good medicines, but then, can’t they cause any disorder in the body seeing you are just taking the medicines and yet you are not sick, because for someone like me here, am not sick? So I can’t just take any medicines when I don’t have any problems. Can’t they cause any disorder?*
104. I: Well, I think the right answer for that question can be found if we consult the doctors, for now, our answer is: we don’t know whether or not they cause disorders. But assuming they don’t cause any disorders, what can be your answer to the question?
105. *R: I think they are good medicines.*
106. I: Really?
107. *R: Yes.*
108. I: Ok. Would you encourage someone to be taking the pills?
109. *R: Of course, I can encourage them.*
110. I: Why?
111. *R: It’s because…well, if am to be honest, the best way for me to encourage my friends is on a condition that I see the medicines producing positive results to those who use them. But apparently, I don’t know anyone who has ever used them, and so I can’t promise to say they are good to someone. It’s only when I see how they work.*
112. I: Alright. If PrEP become available, what is your opinion on integrating PrEP with Voluntary Medical Male circumcision services?
113. *R: I think that cannot be a good idea as I earlier on said, that the VMMC Clinic is sponsored by NGOs, and these are not stable people. They can be here today, and tomorrow you find that they are gone. So if at first, you received the medicines from these VMMC people, and once they are gone, you will not complete the dose and that will be a challenge to you. I prefer that these medicines should be found at a stable place where anyone who would like to use them should have that direct access to get them.*
114. I: So your opinion is that PrEP should be there, but at a stable place, and not at the VMMC?
115. *R: Exactly.*
116. I: Mh... How do you think PrEP would be offered in this clinic, assuming the NGOs at VMMC are a stable organization? Who should be providing, and to who and at what time?
117. *R: I think there will be a special doctor to be offering such medicines, and not the VMMC people.*
118. I: Mm... SO should they be given to anyone who comes at the VMMC?
119. *R: They can’t afford to offer to anyone who comes at the VMMC Clinic. I think they can only manage to offer to those who have the desire to receive at the health centre.*
120. I: What do you think can be the right time to offer PrEP for those who come at the VMMC Clinic?
121. *R: Though we can make it clear to them that offering PrEP is not part of our services, I would still prefer to educate them about this at the time that we counsel them on the STIs and HIV, the time we inform them about the reasons that they need to come for VMMC. I think that’s the best time for us to explain to them about it.*
122. I: Apart from the fact that you have mentioned that NGOs can go at any time, what do you think can be the other barriers to PrEP?
123. *R: The other barrier can be that there can be some people who can refuse to take the medicines because they are not sick.*
124. I: How can you help such people if, say, you had an opportunity to use the medicines, and you see that they have no impacts? How can you help such people?
125. *R: If I had that opportunity to use them, and then I saw to it that they had no impact, I would go and sensitize the people in the villages the way the health care people does. I can make sure I create some time to inform people especially those whom I chat with about.*
126. I: If you were given powers to choose and integrate services in Voluntary Medical Male Clinics, what are the services that you would think of Integrating?
127. *R: I can go for HIV testing.*
128. I: Mh. As a couple, or just individually? There are times that men are told to come with their wives, and there are also times that even a person like me can just come and go for the test. Which one would you prefer?
129. *R: I think for the one who wants to be tested. I don’t think someone who doesn’t want to get circumcised can just go to the VMMC Clinic and say “I want to be tested for HIV.” I think that one cannot be allowed.*
130. I: Why have you chosen HIV testing out of all the services that are there?
131. *R: I have chosen HIV testing because that’s the only way one can know his status.*
132. I: Ok.
133. *R: Sure. Anyone can at any time just go to the health center for HVI testing. When you know your status, you help yourself to have a health life.*
134. I: Ok, alright.
135. I: Thank you very much for your time. Your answers will be very helpful in improving the health service delivery at circumcision clinics.
136. *R: Mmm, thank you.*
137. I: Before we close, is there anything more you would like to say?
138. *R: My question is; what will be the outcome of what we have enlightened to one another here?*
139. I: The outcome will be that they will consider what you have mentioned here and they will do according to your opinions regarding the services. If possible, they will add extra services at the VMMC Clinic.
140. *R: Ok!*
141. I: Yes, the services that they will provide will be the same ones that we were discussing here. So, you might wonder why a couple will come at the VMMC for HIV testing, but that will all be the result of what we have discussed.
142. *R: That is, a result of our discussions and pleas?*
143. I: Yes, exactly.
144. *R: Ok.*
145. I: Sure. Thank you very much.

END

**D 43 STUDY**

**Date of Interview: 10 August 2018**

**Type of Participant: Peer or Clinic Aid**

**Interview Number: D-43-0015**

**Interviewer: I.N.**

**Total Interview Time: 43 minutes 50 seconds**

**Interview Summary:** **(from summary sheet)**

| **SERVICE TO BE INTERGRATED** | **THOUGHTS ON INTERGRATION** |
| --- | --- |
| Couple HIV Testing and Counseling | Thinks it is a good idea but he thinks on its own it cannot work. Also think, this can work better, if antenatal services could be integrated at VMMC. |
| STI Services | Thinks it’s a good idea |
| Family Planning | Thinks it is a good idea. Thinks men are far more behind than female on this part, therefore it is necessary to be integrated at VMMC. |
| Cervical Cancer Screening | Thinks it is a good idea, especially if they could be going as a couple, so that if a problem is found, could be solved at once. |
| PrEP | Thinks it is a good idea. Would prevent spread of a virus |
| Other Services | Couple HIV testing and counselling, Cervical cancer screening and PrEP. Also thinks that integrating antenatal services at VMMCC, would be a good thing. |

**Remarks:**

**Participant was confident, rush in responding but provided short information. He could explain with a number of real life example but could lost a track or necessary points and got confused of his own statement, like he was not knowing. He needed a further push to explore more ideas, though he was much knowledgeable.**

**Interview Text:**

I: Thank you for taking the time to talk with me today.

*R: Yes.*

1. I: I would like to ask you some questions today about the way you feel and what you think about some issues related to the service you provide and how we can include other services in Voluntary Medical Male Circumcision (VMMC) clinics.
2. *R: Yes.*
3. I: There are no right or wrong answers to these questions.
4. *R: Yes.*
5. I: We would like to hear your opinion and your experiences in your own words. Do you have any questions before we begin?
6. *R: Mh… no.*
7. I: No question?
8. *R: Yes.*
9. I: Yes, can you tell me how you are involved in the client care at this clinic?
10. *R: Am involved through the issue of circumcision. Before you get circumcised, they firstly take you for blood test. If you are find without infection after getting your blood tested, that is when, you get circumcised. But if you are found with infection that is when you are not allowed to proceed for circumcision.*
11. I: So what is your role?
12. *R: Yes.…my role…. ehm[ confused]*
13. I: or we can say, what do you do, or which part do you take in issues of circumcision?
14. *R: My role is to influence people for circumcision. Because circumcision is one part were… your penis remains hygiene… second to know your blood status…after circumcision, you cannot contact HIV virus easily because you have circumcised. But we are not saying that, through that way you cannot get diseases as through the same way, some people end up contracting diseases.*
15. I: Have you circumcised?
16. *R: Yes.*
17. I: when did you circumcised?
18. *R: Yes.…20…July, 2013.*
19. I: what made you to go for circumcision?
20. *R: Ah, what made me is, you can get diseases when, your penis is not circumcised*
21. I: Mh…
22. *R: Yes…. Like gonorrhea, syphilis… ehm*
23. I: Okay. Do your peers talk to you about how the services are provided here?
24. *R: Yes, we could talk*
25. I: What do you talk about? Can you give me an example?
26. *R: The issue of circumcision, that it better is youth do not hesitate for circumcision because even this was written in the bible, as at first we used to take circumcision as only applying for Muslims but as of now, since people are aware, after knowing bible, they now know, that this is not only for Muslims… ehm.*
27. I: Does your peers ever talk to you about the services which are provided here?
28. *R: They did tell me, that circumcision clinic, is a good clinic because most of people, we were not aware about that.*
29. I: Okay, but what did they say concerning the services which they received at this clinic?
30. *R: On that part, they did not tell me.*
31. I: Now let us talk about partner HIV testing here at the Voluntary Medical Male circumcision clinic
32. *R: Yes.*
33. I: Tell me what happens if a man brings a spouse here at the Voluntary Medical Male circumcision clinic?
34. *R: They want to know about their health status, whether they do not have HIV virus. If they do not have HIV virus, they are supported to protect themselves. If one of the partners is found with a virus, they are not supported to separate but their relationship should continue and they should be using condoms… yea.*
35. I: What do you think are the motivators that make the men bring their spouses here for testing?
36. *R: Sometime, there might be trust issues between them. That my partner you are careless. Yes… others, they do want to know their status, what is my blood status because if you have been found with a virus, in good time, you are supposed to start taking drugs, in good time. While if you hesitate, you might found that, you have created another problem…yea.*
37. I: What do you think demotivates men to bring their partners here for HIV counselling and testing?
38. *R: Some do feel embarrassed. They do feel embarrassed to come with their partners. Some people hide, they could go to the hospital alone without their partner knowing as he or she might be knowing that she engaged in some promiscuous ways, maybe she has a virus while the man is not with a virus. Some men do not trust their wife and if the wife is found with a virus that becomes the end of marriage.*
39. I: Okay.
40. *R: Yes.*
41. I: What do you think can be done to make men bring their partners here for couple testing and counselling?
42. *R: We need to do campaigns, encouraging people to go to hospital for HIV testing.*
43. I: Okay. You as a peer, what is your opinion on integrating couple counseling with Voluntary Medical Male circumcision services?
44. *R: It is good that, as men are going for circumcision, they should be coming together with their partner.*
45. I: Why do you think in that way?
46. *R: Ah because…. men may not be willing for that but when his partner wants, but nowadays is it common that, when a woman is expecting, men are supposed to escort her for antenatal services and most men feel shy like I cannot go to antenatal clinic with her. The other way which we can do is, when men are going for circumcision, if they can put antenatal services there, he cannot get demotivated because it is already there.*
47. I: Okay
48. *R: Yes.*
49. I: What do you think are the barriers and concerns on this integration?
50. *R: Mh… There can be a number of barriers because others cannot get used to go to such kind of the place. It depends with your conscious that I should go and do this, yea… and this can be a barrier or follow up.*
51. I: What do you think, are other barriers or concerns for this service to be integrated at this clinic of circumcision?
52. *R: This cannot work because it may need people to think critically and accept it in their heart that, this thing should really go there.*
53. I: Ah, what do you think can be done to overcome these concerns and barriers to couple counseling in Voluntary Medical Male circumcision services clinic?
54. *R: We need to analyze thoroughly that, will people like this thing? will they like it or not… yea*
55. I: What ways do you think we need to follow there on analyzing, which ways do we need to follow or what should we do?
56. *R: Motivate people using circumcision method.*
57. I: Now I would like to discuss with you about sexual reproductive health services and Pills HIV prevention: called pre-exposure prophylaxis. (PrEP). Sexual reproductive health includes services that promote good sexual health and reproduction. They include but not limited to family planning, sexual transmitted infection management, cervical cancer screening, Condom distribution and many more. Today we will only discuss about family planning, diagnosis and management of STIs, Cervical cancer screening, and PrEP. We will look at each of these one by one. Let us start with STI services.
58. *R: Yes.*
59. I: Explain to me what happens if a client is suspected or diagnosed with an STI here?
60. *R: If happens that, he did not protect himself.*
61. I: So, what happens there? Do they continue with circumcision process or how does it go?
62. *R: No. If he has been found with STI, he does not get circumcised.*
63. I: What do they do?
64. *R: They send him back home as he is not supposed to get circumcised.*
65. I: Does the person found with STI sent home just like that or he receive some services?
66. *R: He just sent home just like that*
67. I: Or even without being treated like given drugs?
68. *R: Ah… they give him treatment of drugs because it depends with the person, because I remember 2013, when I came for HIV testing, that time I came for circumcision, a certain person was found with HIV and he did not even have time to sit down as he run away.*
69. I: Oh?
70. *R: Yes, by the time, they were calling out his name, he was not there…. yes, many people who realize that, they have been indulging in bad ways which could lead into contracting diseases, they got frightened. Even if you are found with a virus, you think two, three times like who did I have sex with… yea*
71. I: So, let us take you have been found with gonorrhea, do they just send you home?
72. *R: No, they gave you treatment of drugs.*
73. I: They gave you drugs?
74. *R: Yes, then they explain to you the whole process*
75. I: What happens if you are healed? Do you come back for circumcision or you continue staying at home?
76. *R: If you are healed, that is all, you just stay home*
77. I: Don’t they allow you to come back for circumcision?
78. *R: Ah, if you get healed, they allow you to come for circumcision.*
79. I: They come?
80. *R: Yes.*
81. I: You as a peer, what is your opinion on integrating STI services with circumcision services?
82. *R: Ah, on this one, screening…. It is good that….. [lost]*
83. I: Should I repeat the question?
84. *R: Yes*
85. I: You as a peer, what is your opinion on integration, like here at circumcision clinic, they should integrate STI services, do you think it is a good thing or not?
86. *R: It is a good thing*
87. I: Okay, what do you think about that?
88. *R: On that part…ehm... I think it is a good thing if they put in that place, after you agreed with people from circumcision clinic after communicating with each other, that is it possible to change, that all should be working together? The time a person is coming for circumcision and integrating that, so it could be a good thing. But also, it is not good for integration because you know other people do feel shy.*
89. I: But as to you, how do you see it?
90. *R: As for me, I do see it as a good thing.*
91. I: Ah, what is it that you would like about the integration of diagnosis and management STIs with Voluntary Medical Male Circumcision services?
92. *R: Ah, that part of screening, is what I think, it is good.*
93. I: Is it good?
94. *R: Yes.*
95. I: In what way, is it good?
96. *R: It is good, if a person understands… understands, you see the advantage and disadvantage of it. Yea.*
97. I: Okay.
98. *R: Yes.*
99. I: Alright. So what is it that you would not like the integration of diagnosis and management STIs with Voluntary Medical Male Circumcision services?
100. *R: Ah… the circumcision...*
101. I: Yes, that they should integrate on circumcision, STI screening on this circumcision clinic, what is your opinion? What are the things that you would not like on that one?
102. *R: No… on that part… [laughing]*
103. I: Let me ask you in this way, do you like it or not?
104. *R: I don’t like…, I do like*
105. I: You like it?
106. *R: Yes.*
107. I: In what way?
108. *R: In the way that, if they can officially declare and put in place, men’s way, on sexual issues with circumcision clinic [was not clear what to say] … is one way which can help us to be people who understands clearly.*
109. I: Yes, which means there is nothing which you do not like right?
110. *R: Yes.*
111. I: So, how do you think STI services should be offered at the Voluntary Medical Male circumcision clinic?
112. *R: Sometimes, it is not good and sometimes it is good.*
113. I: Okay, so you said that, you think you like the idea that here, at circumcision clinic, they should integrate STI services?
114. *R: Yes.*
115. I: How do you think this service should be offered, at what time when you come for circumcision?
116. *R: The time after circumcision, it is a time when be offering that, the time men is coming from circumcision. After circumcision as he is about to be leaving for home, that is when he should be screened for STIs.*
117. I: After being circumcised as he is about to start off home?
118. *R: Since he has already been screened that he does not have infections after circumcision, he is supposed to do that.*
119. I: Okay. So where do you this STI services should be offered? Like within voluntary medical clinic, private room or outside
120. *R: It is supposed to be a private room …ehm*
121. I: Like within the VMMC or outside?
122. *R: No within…*
123. I: In the clinic?
124. *R: Yes.*
125. I: What do you think are the barriers and concerns on this integration?
126. *R: There are a lot of barriers, others will not accept that it is not supposed to be like that. Others do accept that, this is truly good, if they can integrate, things can work well. Because a person does feel shy, if he has made a decision that I want to do that, for example me, if I went for blood test, I could tell like other people, I do not shy even If a woman is there, or if other women has come, they can test me on their presence but I cannot feel shy, because I know about my body.*
127. I: So how do you think that concerns or to say barriers can be addressed?
128. *R: How can it be addressed?*
129. I: Yes.
130. *R: We can address it if people agree, that this is how it is supposed to work like…yea*
131. I: Like how? How do you mean by that agreement?
132. *R: Eh… other questions [laughing]*
133. I: No, just answer them in the way you think like, it is not that it is write or wrong, but what you think
134. *R: The concerns are there, because we people, we are not that much aware. So we can say that, yes or no. So we cannot*
135. I: So how can we address the concerns? You, yourself, you did explain about concerns, how can we address them? Like the concern of shyness, how can you address it?
136. *R: The concern of shyness, we can address it because if we can explain to people, they cannot be having shy because they may be knowing that the things which we are doing is not supposed to be of secret. In world, there is nothing of secret, yea.*
137. I: Who is supposed to be explaining to people?
138. *R: You, the doctors and the person, you need to talk with a person.*
139. I: Alright, so let us discuss about family planning. Can you explain to me, what happens if a client needs a family planning methods? Or have you ever heard about family planning?
140. *R: Ah, I heard about family planning*
141. I: How does it work?
142. *R: Family planning depends on, if a woman is not practicing family planning, she is bearing children without space, sometimes that women die, because if a woman, this year have a child, next year another child and the other year child, her body grows unhealthy… yes. That’s why, they do say that, we men we need to be doing family planning, yes,*
143. I: So, can you explain to me, what happens if a man need family planning? How does it work?
144. *R: If a man needs family planning, he is supposed to agree with his partner, like “wife go and do injection method for family planning or husband, if he need family planning method, he should be using whether condoms, so that his wife should not get pregnant early.*
145. I: Which are the methods that you do know about family planning? I have heard you mentioning some, can you just repeat?
146. *R: family planning methods include issue of injections, some get pills… yea, that’s all.*
147. I: Other methods
148. *R: Others, using condoms… yes.*
149. I: Okay, is there any other which you do know?
150. *R: Ah, the other method? Aaah no, that’s all*
151. I: You as a peer, what is your opinion on integrating family planning in circumcision services?
152. *R: They should integrate?*
153. I: Yes.
154. *R: Most of men, we do not know, because [yawning], when they say family planning, people think, think that men does not need family planning and women also do not, especially men. Men, we are the one who are far behind than women… yea*
155. I: Okay, so you, what is your opinion on integration the service of family planning with circumcision services?
156. *R: It is one way which can help us, we as youth… yea*
157. I: What is it that you would like about the integration of Family planning with Voluntary Medical Male Circumcision services?
158. *R: It is one way that, at the men’s way, they can put a room so that by the time men can be doing circumcision, the women should also be there, so that she can help with her husband, that they have chosen a right method of family planning. Because, there are other people, men or women, they do not even know family planning. So that will take us, that those in a family should agree to do this this this, so that it can take us to be people, who can realize easily.*
159. I: So when this should be offered?
160. *R: The time when we are doing circumcision*
161. I: When a man is doing circumcision, his partner should also be there?
162. *R: Yes.*
163. I: Within the same clinic [VMMCC] or outside?
164. *R: Outside or even inside… let us just say outside*
165. I: The clinic should be outside [for family planning services]?
166. *R: Yes.*
167. I: What is it that you would not like about the integration of family planning with Voluntary Medical Male Circumcision services?
168. *R: Mh… there are a lot which can make a person not to support integration.*
169. I: Can you mention two or three?
170. *R: Some feel shy on a group, like to be together*
171. I: Like a couple?
172. *R: Yes…* they do feel shy like ‘’ should we go to the same place together? No,… yea
173. I: Other thing, shyness, other thing….?
174. *R: Shyness, fear, scared*
175. I: Other thing?
176. *R: Laziness. Laziness of going to the clinic to do that [family planning]…yea*
177. I: Mh, how do you think family planning services would be offered within Voluntary Medical Male circumcision clinics?
178. *R: By the time when we are going for antenatal services, when a woman for instance let us use an example of a woman, nowadays it is common that, when a woman is pregnant, she is supposed to be going to the hospital with her husband, yea… so that is the time when we can offer it or when we are going for circumcision… yea, we can pass through a private room… yea*
179. I: What do you think are the barriers and concerns with the integration family planning and Voluntary Medical Male circumcision services?
180. *R: There are a lot of barriers*
181. I: Like what barriers?
182. *R: Others may not accept, like “no, circumcision, antenatal clinic should not be integrated, and what.*
183. I: Mm…. we are talking about circumcision clinic here
184. *R: Yes.*
185. I: Does circumcision also offer antenatal services?
186. *R: No.*
187. I: Yes, so we are talking about circumcision clinic
188. *R: Yes, of circumcision, others do not accept it.*
189. I: Why do you think; they do not accept?
190. *R: Ah, shyness… others it is because of shyness itself.*
191. I: What do you think, need to be done so that those concerns or barriers you have mentioned need to address?
192. *R: We need to think deeply about that, so that it can be addressed.*
193. I: So what are other ways which can be followed… to address these concerns?
194. *R: The issue is about alerting …. If people know, they may not be afraid.*
195. I: If you say, alerting what do you mean?
196. *R: Alerting that people should know.*
197. I: Alright. Can you give me an example of how you can do that?
198. *R: [Laughing]*
199. I: For instance
200. *R: I can give you an example that, the way we do for circumcision, the only way which makes a person not to be afraid is to alert that “men, women, let us go the hospital, to know about our blood status, to know about cervical cancer” yea, that is the only way we can alert people or using yellow vans (car), people can know fast*
201. I: Okay…
202. *R: Yes.*
203. I: So let us talk about cervical cancer screening for female partners. Explain to me what happens if a woman needs cervical cancer screening?
204. *R: They want to know about their body status because if the cervical cancer is not noticed earlier, the results you get sick or even die…. yea*
205. I: So you as a peer, what is your opinion on integrating cervical cancer screening for female partners with Voluntary Medical Male Circumcision services?
206. *R: It can help that, people we need to realize earlier when our wife or she is expecting and she is feeling pains in the body, we need to rush to the hospital so that the doctor can screen her in time because a person can be just saying that am having stomach pains while something bad has happened, maybe cervix is not well. So what is needed is that, the earlier they notice of it, they need to be given drugs in time so that she gets healed…yea*
207. I: So, what is it that you would like about partner’s cervical cancer screening integration with Voluntary Medical Male Circumcision services?
208. *R: I would like it because I have known the good and bad thing of it*
209. I: Okay, what is it that you would not like about the integration of partner cervical cancer screening with Voluntary Medical Male Circumcision services?
210. *R: Ah… as for me there, there is nothing, which can let me take time not to let it be integrated in circumcision. It is supposed to be integrated, so that people can know.*
211. I: How do you think is the best way to offer cancer screening within Voluntary Medical Male circumcision clinics?
212. *R: It is one way where we can have a private room while we are going for circumcision. In private room or outside, so that people should recognize it fast…yea*
213. I: So, if you bring your spouse, how will it be working like, in terms of time?
214. *R: The time that we have done circumcision, that’s when we are supposed to or before circumcision, we should start from there… screening a female partner, then a man should go there [circumcision]… yea*
215. I: What do you think are the barriers and concerns about integrating partner cervical cancer screening and Voluntary Medical Male Circumcision services?
216. *R: There cannot be any barrier because most of people can be aware*
217. I: Aware of what?
218. *R: the bad effects of the disease*
219. I: Alright. What do you think should be done to address these concerns and barriers?
220. *R: There cannot be any barriers because many people are aware.*
221. I: Okay. Now let us discuss about PrEP. Have you heard about this before?
222. *R: Ah, no. I have never heard.*
223. I: You have never heard?
224. *R: Ah, hearing about it, I do but here and there*
225. I: PrEP? What do you hear about it? What do they say?
226. *R: That people should be taking PrEP, like a person with HIV positive, before he is found with HIV, he should be taking it*
227. I: The person with HIV, should be taking PrEP?
228. *R: Before he is found with HIV virus*
229. I: After testing?
230. *R: After testing but found negative*
231. I: He should be just taking PrEP?
232. *R: Yes.*
233. I: When should he be taking PrEP? Like every day or ….
234. *R: Ah, not everyday*
235. I: But what time?
236. *R: eh…the time….*
237. I: You said he should be taking it, but what time? After having sex or…
238. *R: After having sex.*
239. I: With a person what status?
240. *R: Who he is suspecting that, this one has it*
241. I: After sex, he should take it?
242. *R: Yes.*
243. I: What you have just mentioned is not PrEP but PEP.
244. *R: PEP?*
245. I: Yes, they say PEP.
246. *R: Okay*
247. I: Because PrEP, aaah, is anti-HIV medicine that keeps HIV-negative people from being infected. There is a single pill that is taken once daily, and if you take it regularly, it is highly effective at prevention people from being infected.
248. *R: Okay.*
249. I: So, how do you feel about PrEP?
250. *R: It is good that people should be taking it, maybe the other is HIV positive, so he need to be taking it.*
251. I: If PrEP was made available to HIV negative men and women. Do you think you could advise your HIV negative peers to accept to take PrEP?
252. *R: Yes, it has to be available*
253. I: Mm…. Ehm, how do you feel in your heart about PrEP? Or the way you have heard here, how can you explain about PrEP?
254. *R: PrEP?*
255. I: Yes, if a person has to ask you about PrEP, what is it?
256. *R: Is a medicine which is taken once daily*
257. I: Is it malaria medicine?
258. *R: [Laughing] that prevent from contracting HIV virus*
259. I: Ah, what are the reasons you would encourage your clients to take PrEP?
260. *R: Mm… the reason… is …. ehm*
261. I: Because we have talked that PrEP is ant-HIV medicine, you do take it when you know that my partner has a virus but you cannot get it
262. *R: Yes.*
263. I: So, what reasons do you have to encourage your clients to take PrEP?
264. *R: Because most of times, women might have a virus, so this can help her partner not to get a virus.*
265. I: If PrEP becomes available, what is your opinion on integrating PrEP with Voluntary Medical Male circumcision services?
266. *R: It is good because people will realize quickly because by the time their partner or has been found with HIV, they are supposed to be taking it so that one cannot get that virus.*
267. I: Mm… Would you encourage clients to take PrEP?
268. *R: Yes, I would like that and I will be alerting that people should be receiving such kind of medicine, it should prevent the spread of disease in the body, so that they could not get a virus from his wife or her husband.*
269. I: How do you think PrEP would be offered here at Voluntary Medical Male Circumcision Clinic?
270. *R: In a private room but within VMMCC there, at a time when a man has not been found a virus, he is supposed to go and receive, so that he can be taking it, when his wife has been found with a virus, he is supposed to be taking it… yea.*
271. I: What do you think could be the concern and barriers to integrating PrEP Voluntary Medical Male Circumcision services?
272. *R: Others may not be willing to accept it*
273. I: Why would they not be willing to accept it?
274. *R: Ah, because we do not know, other they do not accept like I cannot take that, but it is a good way by taking it as when you are taking it you might be able to get a virus from your partner who is positive or even you if you have it, your wife who is negative, might not be able to get it.*
275. I: So they might not be willing accept it?
276. *R: Yes.*
277. I: So that is like barrier and concerns?
278. *R: Yes.*
279. I: Anything else, what do you think are other barriers? Besides not willing to accept to take PrEP.
280. *R: [Laughing] I do not have any more answers*
281. I: Okay, what do you think should be done to address these concerns and barrier?
282. *R: There is a need to do research*
283. I: Concerning what?
284. *R: Campaign that PrEP should be taken*
285. I: On a research, like what? What questions would you like to be included in it? Like what?
286. *R: Analysis thoroughly…*
287. I: Thoroughly in terms of what?
288. *R: Mm….*
289. I: Or what need to be researched about PrEP?
290. *R: Just asking people, that could they accept it? Or not*
291. I: Okay. Aaah… so let us talk about other services. If you were given powers to choose and integrate services in Voluntary Medical Male Clinics, what are the services that you would think of to Integrate? For example, from others which we have already discussed
292. *R: Like cervical cancer screening, HIV [couple testing] and PrEP. That is what I can choose.*
293. I: Okay. Why could you choose for cervical cancer screening?
294. *R: Because many people we need to realize that…..to know about our body status*
295. I: Okay, you have also chosen PrEP, why?
296. *R: For PrEP, because I want that, if we have been found with a virus, that also our partner should not get a virus that can make us happy.*
297. I: You also chose couple HIV testing. Why have you chosen it?
298. *R: Because most of people, are far behind, they do not want to go to the hospital for testing on their own. But when you are going for circumcision, you need to be tested before you proceed for the circumcision.*
299. I: So, just on repeating, how should the service of cervical cancer screening be offered at this clinic?
300. *R: The service of cervical cancer screening is supposed to be offered, if within a family, a wife is found with a problem, they have to agree with her husband at a time they are going to the hospital, whether a husband is going for circumcision, he is also supposed to go there and here the results of his wife.*
301. I: During screening?
302. *R: Yes.*
303. I: So about PrEP, how should it be offered at a circumcision clinic?
304. *R: It is supposed to be offered by a doctor but also we, ourselves, we also need to pay more attention on it.*
305. I: Okay, so about couple HIV testing
306. *R: On couple HIV testing*
307. I: How should this service be offered?
308. *R: This service should be offered without taking sides because many time, we men, we do not like going alone for HIV testing even when a campaign for HIV testing starts “They say, there has come people who test blood” but people do not go. But we should not deny but to know the status within our body, the way we are. So it is good to integrate the service there, so that people can realize about it quickly.*
309. I: Okay. Aaah, thank you for taking your time to discuss with me today. Your answers will be very helpful in improving the health service delivery at Voluntary Medical Male circumcision clinics. Before we close do you have any question?
310. *R: Ah, I do not have a question.*
311. I: you do not have?
312. *R: Yes…*
313. I: Alright.
314. *R: Yes.*

**D 43 STUDY**

**Date of Interview: 10 August 2018**

**Type of Participant: Male Index Participant**

**Interview Number: D-43-0016**

**Interviewer: I.N.**

**Total Interview Time: 43minutes 59seconds**

**Interview Summary:** **(from summary sheet)**

| **SERVICE TO BE INTERGRATED** | **THOUGHTS ON INTERGRATION** |
| --- | --- |
| Couple HIV Testing and Counseling | A good opportunity for couples to test together. Partner can also benefit by getting cervical cancer screening. |
| STI Services | Thinks STI services would help people to know their status and start treatment once they have infections. |
| Family Planning | The integration would make it easier for people who need family planning at the VMMC clinic. |
| Cervical Cancer Screening | Feels integration will help men to know whether their partners have cervical cancer or not before getting VMMC. |
| PrEP | A good idea as this integration would help to fight HIV better. Only concerned that people would not protect themselves. |
| Other Services | Thinks family cervical cancer screening and PrEP are the best for the integration. |

**Remarks:**

**Participant was relaxed and had a good understanding of the services. He asked questions whenever he didn’t understand.**

**Interview Text:**

1. I: Thank you for taking the time to talk with me today.
2. *R: Alright.*
3. I: I would like to ask you some questions today about the way you feel and what you think about some issues related to the service you receive here and how we can include other services in Voluntary Medical Male Circumcision (VMMC) clinics.
4. *R: Alright.*
5. I: There is no right or wrong answers to these questions. We would like to hear your opinion and your experiences in your own words. Do you have any questions before we begin?
6. *R: No I don’t have any questions.*
7. I: Alright. What role do you take in Voluntary Male Medical Circumcision services?
8. *R: I talk to friends the benefits of VMMC and sometimes they listen to me or not depending on where they come from because some people come from areas where they don’t do circumcision and it can be difficult for them to make a decision to go for VMMC on their own.*
9. I: What message do you give them about VMMC?
10. *R: We tell them the benefits of VMMC that it reduces risk of cervical cancer and promotes cleanliness.*
11. I: Mm… okay, you mentioned cervical cancer, what other infections are reduced by VMMC?
12. *R: HIV and STIs.*
13. I: Mm… okay, so what happens when the person accepts?
14. *R: We show him where VMMC is done and tell him not to be afraid when he gets there because some people fear the injections just because they were told that you get 10 at once which is not true.*
15. I: Mm... Give me an example of a person that you convinced to get VMMC.
16. *R: It was [name withheld]*
17. I: Mm... What did you say to him?
18. *R: I told him that there is a new circumcision that is done at the clinic and it is different from the ones we do at the villages because when you go to the clinic to get VMMC you go back home the same day. Also depending on how far away your home is from the clinic, they may provide you with transportation. So he accepted then later went for VMMC. Sometime later the wound healed and he came and told me that I helped him.*
19. I: Let’s now talk about couple testing and counseling.
20. *R: Couple testing and counseling?*
21. I: Mmm. Tell me what happens when a man brings his partner to the VMMC clinic?
22. *R: Firstly, it’s a few men who can be open enough to bring their wives to the VMMC clinic because they will feel shy. It’s a bit different from younger people who are not married.*
23. I: Would you bring your girlfriend?
24. *R: Mm… that is hard because…maybe she may accept but the way I see it I don’t think she can accept.*
25. I: At the time that you did VMMC, did you bring your partner?
26. *R: No, I did not.*
27. I: Why is that?
28. *R: It’s because I didn’t have one at that time.*
29. I: So let’s say you had one at the time, would you bring her?
30. *R: If she accepts…?*
31. I: Yes, say she proposes that you bring her.
32. *R: If she says so then I can bring her because it’s not like she will be there when the real things will be happening.*
33. I: What would motivate you to bring your partner?
34. *R: It’s because some women say they can’t have sex with a man who didn’t do VMMC…*
35. I: What may be her aim for coming to the VMMC clinic?
36. *R: She may want to see for herself that you have done it because she may have loved you just because you told her that you want to do VMMC, and may want to encourage you to do it.*
37. I: What do you think makes men not to bring their partners at the VMMC clinic for HIV testing?
38. *R: They feel ashamed to bring their wives because some women are too talkative and may start telling their friends about this issue which may bring ridicule on the man seeing that he got VMMC at an old age.*
39. I: Mm... what do you think should be done for men to bring their partners at the VMMC clinic?
40. *R: They need to know their status so they can plan their future.*
41. I: Alright, you mentioned that some men may feel shy to bring their partners so what can be done so that they don’t feel shy?
42. *R: What can be done is telling the women to keep confidentiality because some men fail to bring them to such issues for fear that the partners will disclose it. So they need to be confidential so they can plan their lives with their husbands.*
43. I: Alright. What is your opinion on integrating couple HIV counseling with Voluntary Medical Male circumcision services?
44. *R: I think it’s a good idea because and it will benefit the couple because if they test for HIV and the woman screens for cervical cancer, they will be protecting themselves. Also when the man gets VMMC they need to be open to each other.*
45. I: Mh. What do you think are the barriers and concerns on this integration?
46. *R: There can be a barrier because HIV testing is a confidential issue which some people want to be kept private. Now if you bring your girlfriend for couple testing and counseling and she knows your status then later your breakup, those results will be out and that cannot be good.*
47. I: How can we address such a concern?
48. *R: If funding is there, they should employ some staff who will be sensitizing people to keep confidentiality. If one of the couples is found to be HIV positive they do not need to discriminate against him or disclose his status to other people*
49. I: Mm... Okay
50. *R: Sure.*
51. I: I would like us to talk about sexual reproductive health services and Pills for HIV prevention: called pre-exposure prophylaxis. (PrEP) Sexual reproductive health includes services that promote good sexual health and reproduction. They include but not limited to family planning, cervical cancer screening sexual transmitted infection management cervical, condom distribution and many more.
52. *R: Okay.*
53. I: Today we will only discuss family planning, Sexual transmitted infection management cervical screening and PrEP. We will look at each of these one by one. Let us start with STI services.
54. *R: Mh…*
55. I: Tell me what happens when a person is suspected or diagnosed with STIs?
56. *R: It becomes a problem for such a person to get VMMC because it may happen during the surgery that the provider cuts himself and that can expose him to infections. So the providers may choose to put those that have STI aside and help those that don’t have STIs first, then later he can do the surgery on the ones with the STIs.*
57. I: Do they give any reason why they put them aside?
58. *R: No they don’t.*
59. I: As a peer, what is your opinion on integrating STI services with Voluntary Medical Male Circumcision services?
60. *R: For everyone?*
61. I: Yes, for everyone. Right now they provide STI services only for those that come for VMMC, right?
62. *R: Yes.*
63. I: Now we are talking about integration where STI services will be offered along with the VMMC service.
64. *R: Well it’s a welcome idea because people will know their status. If they are found to be positive they will start taking treatment while those that are found to be negative will get counseled on how they can prevent the infection.*
65. I: Mh. What is it that you don’t like about this integration?
66. *R: What I don’t like about VMMC or about the STIs?*
67. I: About the integration of VMMC and STI services.
68. *R: The challenge can be supplies because sometimes they tell you they don’t have supplies like scissors and turn back other people until they get another supply. So sometimes it becomes difficult for some people to build that confidence again and go back to the clinic when they are told that the clinic has run out of anesthesia. They may have been exposed to a lot of talk about VMMC which is there in the communities. SO it’s an issue which the government needs to work on.*
69. I: What should be done to deal with such a challenge?
70. *R: Whenever we as peers find people who want VMMC we need to inform the clinic first so that they can be prepared. So the clients can be split in groups so that no one has to be turn back due to supplies. Rather than the clients just going there anyhow only to be told that there is no anesthesia at the clinic.*
71. I: Okay, how about for those that only want STI services? Should they also be split into groups?
72. *R: Can you repeat the question?*
73. I: You said for those that come for VMMC should be split into groups so I am now asking for those that will be coming only for STIs, should they also specify the number of people to come to the clinic in a day?
74. *R: I am not sure if they can run out of the test kits like for HIV testing, for them to specify the number of people. Maybe if it’s drugs then they can specify because they can run out.*
75. I: Okay. So when I talk about STIs I don’t mean HIV only but I mean all the other STIs. At the time when you got VMMC were you screened for STIs?
76. *R: Yes, they did.*
77. I: Not just HIV testing but screening if your penis did not have infections or if you did not have infection.
78. *R: Yes, they screened me.*
79. I: Okay so that’s what I mean that some people can just be coming specifically for STI screenings and not VMMC.
80. *R: Oh okay.*
81. I: So what do you think can be the barrier to this type of integration?
82. *R: I think there will be too much workload because there will be two services being provided which is that of STI services and VMMC. It would be best if they screen first then do the VMMC.*
83. I: Mh. So you mentioned that supplies may run out and now you have said there can be too much workload. So what can be done to deal with these challenges?
84. *R: The government needs to put much effort for this to work so that when one is screened for STIs and found to be infected he should be given treatment right away and if possible he can get VMMC at the same time.*
85. I: Okay. Now I want us to talk about family planning. Do you know anything about family planning?
86. *R: I know that it’s a way of postponing the next birth of a child and limiting yourself to a number of children which you can be able to take care of.*
87. I: Tell me what happens when a person comes to the VMMC clinic to access family planning?
88. *R: I have never come across such an issue.*
89. I: You have never come across this?
90. *R: No.*
91. I: As a peer what is your opinion on integrating Family planning in Voluntary Medical Male circumcision services/clinic? So that clients at the VMMC clinic can be able to access family planning.
92. *R: I think that that will help those that come for family planning at the VMMC clinic to be able to access it. It can happen that a man goes to the VMMC clinic and gets family planning then go home and tell his wife that he has gotten family planning, depending on how long they want to use family planning.*
93. I: What is it that you would not like about integrating Family Planning services in VMMC?
94. *R: The problem can be that it’s only men that go to the VMMC clinic so if women want family planning then it can be difficult.*
95. I: Why can it be difficult?
96. *R: There are some women who are shy and for such to come to the VMMC clinic and ask for family planning, it can be difficult. There are some women though who can easily go and get such services even at the VMMC clinic together with their partners.*
97. I: So how can we deal with the challenge that women can feel shy?
98. *R: by creating a separate space at the VMMC clinic where women can secretly go for family planning while their partners do the VMMC instead of providing the service where men can see them because that can make them feel shy.*
99. I: So you have said men should be separated from women. So at which time point in the VMMC process possible?
100. *R: The right time is when they just arrive at the clinic. As men go for VMMC the woman can be shown the other room where family planning is being offered.*
101. I: Mh... So there should be a separate room and they should be offered at the time when men go for VMMC procedure?
102. *R: Yes.*
103. I: Who should be offered family planning?
104. *R: Every woman that comes to access family planning should be offered the service.*
105. I: Should it be women who come with their partners at the VMMC clinic or just any woman?
106. *R: Any woman.*
107. I: Okay. Let’s go back a bit to the STI services. You mentioned the right time for providers to be offering family planning. How about for STIs? What is the best way to offer it?
108. *R; I think for that they should spare a special day like on a Friday where they can be offering the STI services for couples to be coming*
109. I: So family planning should be offered along with VMMC while STI services should be offered on a special day?
110. *R: Yes, so if possible they can come as couples or by themselves. How about that?*
111. I: Why do you feel it should be offered on a special day?
112. *R: It’s because some women may be busy with other things on other days but they can spare just one day to visit the clinic with their partners.*
113. I: So you want it to be special day so they can come with their partners?
114. *R: Yes.*
115. I: Who should be allowed to get screening for STIs at the VMMC clinic?
116. *R: It should be everyone who wants to be screened.*
117. I: Okay. Now let’s talk about cervical cancer screening. You earlier on explained that VMMC reduces the risk of cervical cancer. Now tell me what happens when someone wants to get cervical cancer screening?
118. *R: They go to a place where cervical cancer screening is done.*
119. I: What kind of places?
120. *R: Like at government or private hospitals.*
121. I: As a peer, what is your opinion on integrating partner cervical cancer screening with Voluntary Medical Male circumcision services
122. *R: I feel like it’s very important because when a man wants to get VMMC it’s because he wants to prevent his partner from cervical cancer. So VMMC on its own does not prevent cervical cancer if it (cancer) was already there, it’s only from the time the man gets VMMC forward. So if the woman gets screened before the man gets VMMC it can help even if she is found out to have the cancer because she can be put on treatment. If she doesn’t have it then VMMC will be able to prevent it. This is better than a man just doing VMMC without knowing whether the partner already has it or not. There have been several cases where women have cervical cancer and yet their partners did VMMC. So I feel this is very important.*
123. I: What is it that you would not like about this integration?
124. *R: That this integration should not be done?*
125. I: Yes.
126. *R: No I like the idea.*
127. I: Mmm… what could be the barriers to this integration?
128. *R: I feel maybe some women may feel shy but that cannot be a real issue if there can be a separate building where the screening is done*
129. I: Okay. So when is the best time to offer this service?
130. *R: The right time is when the men go into surgery. That’s the best time of screen the women for the cervical cancer*
131. I: Alright. So who do you think should be screened for cervical cancer at the VMMC clinic?
132. *R: It should be everyone that comes for the service because this disease can attack any woman.*
133. I: Okay. Now let us talk about PrEP. Have you ever heard about PrEP?
134. *R: No I have never heard about PrEP..*
135. I: Oh okay. So PrEP is an anti-HIV drug which helps to keep HIV-negative people negative. There is a pill that one needs to take every day to prevent HIV infection.
136. *R: Oh okay.*
137. I: So how do you feel about PrEP?
138. *R: Okay…so like anyone who wants unprotected sex can just take the pills?*
139. I: Yes.
140. *R: So I feel like… can you repeat the question?*
141. I: How do you feel about PrEP?
142. *R: Okay I don’t really like it because if people are told that if you take these pills they won’t get infected even if they have unprotected sex, it encourages sex. So I feel it’s not a good drug.*
143. I: [Laughing] now do you think it’s necessary for PrEP to be made available to HIV-negative men and women?
144. *R: Yes, I feel it’s necessary, even though I said I don’t like it. I say it’s necessary because this generation is a weird one where people love sex. So it’s necessary for such people to be protected and be given these pills.*
145. I: Would you encourage someone to be taking the pills?
146. *R: Yes, I can encourage them.*
147. I: What reasons would you have to encourage someone to take them pills?
148. *R: It’s because there are some people even my friends who say they cannot manage to stay a week without sex, some who love unprotected sex and others who have un consented sex or have sex because they are drunk which makes them not to think about protection. I can encourage such people because it can protect them from infection.*
149. I: Mh. If PrEP becomes available, what is your opinion on integrating PrEP with Voluntary Medical Male circumcision services?
150. *R: Would you repeat the question?*
151. I: Mh... If PrEP becomes available, what is your opinion on integrating PrEP with Voluntary Medical Male circumcision services?
152. *R: I think I can be very happy if they do such an integration because sometimes VMMC protects people from HIV infection, so adding that percentage of protection with PrEP means it can increase the protection to 100%.*
153. I: Mh. How do you think PrEP would be offered in this clinic?
154. *R: It can be offered to those that have done VMMC. They can be asked after the procedure if they love unprotected sex with women and be given the pills to be taking daily*
155. I: Mh...
156. *R: Yes, so after the surgery they can ask if you have any interest in taking PrEP. That is the best time. If they don’t love unprotected sex, then they cannot be taking the pills*
157. I: What would be your concern if people are taking PrEP?
158. *R: My concern would be the same That I mentioned that people will be having unprotected sex and since most men that come for VMMC are aged 25 and below who have not yet been married, then family planning may be difficult because people will want to have unprotected sex. This will render our family planning message useless because they will be saying they have taken PrEP and won’t need to protect themselves*
159. I: How do you think we can address such concern?
160. *R: I think people need to be warned that the pills prevent HIV infection not pregnancies. So we can tell them that they can be taking PrEP and still use condoms if they are not married because PrEP reduces the risk of HIV, condoms can break and expose on to HIV but if you are using both then even if the condom breaks then you cannot be infected. This in a way promotes family planning using condoms and HIV prevention at the same time.*
161. I: Mh…
162. *R: Sure*
163. I: If you were given powers to choose and integrate services in Voluntary Medical Male Clinics, what are the services that you would think of Integrating?
164. *R: I can opt for cervical cancer screening because the situation is so pathetic for women. Cervical cancer screening and VMMC are connected because VMMC reduces the risk of cervical cancer in women. So if there is funding I can support this screening because they are connected.*
165. I: Mh…
166. *R: I also like PrEP to be integrated because it is also preventing HIV infection just like VMMC which has a certain percentage that protects against HIV. So these two can support each other if integrated*
167. I: Thank you very much for your time. Your answers will be very helpful in improving the health service delivery at circumcision clinics.
168. *R: Mm, thank you.*
169. I: Before we close, is there anything more you would like to say?
170. *R: I feel we have already talked about a lot of things.*
171. . I: Okay. Thank you very much for talking to me today.
172. *R: Thank you.*
173. END

**D 43 STUDY**

**Date of Interview: 10 August 2018**

**Type of Participant: Male Index Participant**

**Interview Number: D-43-0017**

**Interviewer: I.N.**

**Total Interview Time: 29 minutes 51 seconds**

**Interview Summary:** **(from summary sheet)**

| **SERVICE TO BE INTERGRATED** | **THOUGHTS ON INTERGRATION** |
| --- | --- |
| Couple HIV Testing and Counseling | A good opportunity for couples to test together and know each other’s status. |
| STI Services | Thinks STI services would help people to get treatment early. |
| Family Planning | The integration is not necessary. |
| Cervical Cancer Screening | Feels cervical cancer is a dangerous disease so integration is very important. |
| PrEP | A good idea as this integration would help women to get treatment for cervical cancer early. |
| Other Services | Thinks PrEP is the best for the integration. |

**Remarks:**

**Participant was relaxed and had a good sense of humor. He easily understood the questions and was very open-minded.**

**Interview Text:**

1. I: So thank you for taking the time to talk with me today.
2. *R: Thank you.*
3. I: I would like to ask you some questions today about the way you feel and what you think about some issues related to the service you promote here and how we can include other services in Voluntary Medical Male Circumcision (VMMC) clinics.
4. *R: Alright.*
5. I: There is no right or wrong answers to these questions. We would like to hear your opinion and your experiences in your own words. Do you have any questions before we begin?
6. *R: No I don’t have any questions.*
7. I: Alright. What role do you take in Voluntary Male Medical Circumcision services?
8. *R: I talk to young men about the benefits of VMMC.*
9. I: What is it specifically about VMMC that you tell them?
10. *R: I tell them that VMMC helps to reduce the risk of infections in the country.*
11. I: Mm, okay, can you give me an example of a person whom you were able to talk to about VMMC?
12. *R: Okay I talked to [name withheld] about the benefits of VMMC*
13. I: What did you say were the benefits?
14. *R: That it helps to reduce infections in the country.*
15. I: What type of infection?
16. *R: Like cervical cancer, HIV and AIDS and also it helps in the cleanliness of a person.*
17. I: Okay so what happens when you convince someone like that? Do they come to the VMMC clinic on their own?
18. *R: No we escort them to the VMMC clinic.*
19. I: Mm, okay. Now let’s talk about couple testing and counseling.
20. *R: Okay.*
21. I: Tell me what happens when a man brings his partner to the VMMC clinic?
22. *R: It helps them to start treatment together if they are both found to be positive, and to protect themselves and be faithful to each other if they are found HIV negative.*
23. I: Mm, okay. Why did you not bring your partner?
24. *R: No.*
25. I: Why did you not bring your partner?
26. *R: [No response]*
27. I: Do you have a partner?
28. *R: No.*
29. I: Let’s imagine you had a partner, would you have brought her here?
30. *R: Yes, I could have encouraged her to come with me and brought her if she accepted.*
31. I: At the time when you got circumcised, did you know that you could bring your partner to the VMMC clinic for couple testing and counseling?
32. *R: No I didn’t know.*
33. I: How about now?
34. *R: Now I know that I can bring her.*
35. I: Have you ever seen a man bring his partner here at the VMMC clinic?
36. *R: Yes, on the day that I came for VMMC I saw a man bring his partner here but since he was someone I just saw here and never had a chance to talk to him, I didn’t know why he brought his partner here or what they have been told.*
37. I: In your opinion do you think it’s a lot of men that come with their partners here or it’s just a few?
38. *R: It’s a few men.*
39. I: It’s a few men?
40. *R: Yes.*
41. I: For those that bring their partners here what do you think motivates them to do so?
42. *R: They want to know each other’s status*
43. I: Mh…
44. *R: I believe so.*
45. I: What do you think makes men not to bring their partners here?
46. *R: I think its fear of being found HIV positive and losing their partner in the process, and shyness for some.*
47. I: [Laughing] So what do you think can be done to make men bring their partners for couple counseling and testing?
48. *R: Sensitizing them about the benefits of couple testing and counseling.*
49. I: What are your thoughts about bringing in your partners for HIV testing at Voluntary Medical Male circumcision clinic
50. *R: I think it’s good because everyone needs to know his partner’s status*
51. I: Alright. What is your opinion on integrating couple HIV counseling with Voluntary Medical Male circumcision services?
52. *R: Can you repeat the question?*
53. I: What is your opinion on integrating couple HIV counseling with Voluntary Medical Male circumcision services? So that men ca be bringing their partners for couple testing and counseling.
54. *R: I think it’s a good idea because it will help both partners to know each other’s status*
55. I: Mh... What do you think are the barriers and concerns on this integration?
56. *R: [pause for a moment] I don’t think there can be any barriers or concerns.*
57. I: I would like us to talk about sexual reproductive health services and Pills for HIV prevention: called pre-exposure prophylaxis. (PrEP) Sexual reproductive health include services that promote good sexual health and reproduction. They include but not limited to family planning, cervical cancer screening sexual transmitted infection management cervical, condom distribution and many more.
58. *R: Okay.*
59. I: Today we will only discuss family planning, Sexual transmitted infection management cervical screening and PrEP. We will look at each of these one by one. Let us start with STI services.
60. *R: Mm.*
61. I: When I say STIs I don’t mean HIV but other STIs. Do you know them?
62. *R: Yes, it’s syphilis, gonorrhea and so on.*
63. I: Yes, such STIs. Tell me what happens when a person is suspected or diagnosed with STIs?
64. *R: If a person is suspected or diagnosed with syphilis or gonorrhea, he does not get VMM C because if he does the wound heals slowly. So they give him treatment for the STI first then tell him to come later after the infection is done.*
65. I: At the VMMC clinic, do they offer STI screening for everyone that comes?
66. *R: No it’s just those that come for VMMC.*
67. I: As a peer, what is your opinion on integrating STI services with Voluntary Medical Male Circumcision services?
68. *R: Can you repeat the question?*
69. I: As a peer, what is your opinion on integrating STI services with Voluntary Medical Male Circumcision services?
70. *R: It will help people to know their status.*
71. I: remember I mentioned that STIs here refer to the other infections apart from HIV. So what is your opinion on integrating STI services to the VMMC clinic?
72. *R: It will help people to get treatment early if they are diagnosed early as well.*
73. I: Mh. What is it that you don’t like about this integration?
74. *R: [No response].*
75. I: You know how things work at the VMMC clinic right? So what do you not like about integrating STI services in the VMMC clinic?
76. *R: It will make the clients that come for VMMC to take too much time there before going home because the providers will be busy with those that come for the STI services instead of concentrating on performing the surgeries.*
77. I: So how do you think is the best way to offer STI services at the Voluntary Medical Male Circumcision clinics? At what time point and where should the service be offered?
78. *R: [no response]*
79. I: When is the best time to offer STI services at the VMMC clinic.
80. *R: They should offer STI screening at the time they do HIV testing*
81. I: Mh. They should do it right in the HTC room?
82. *R: Yes.*
83. I: Okay, thank you. You mentioned that what you do not like about this integration is that clients will be delayed at the VMMC clinic. What other concerns are there for this integration?
84. *R: I don’t understand.*
85. I: What are the concerns or barriers for this integration.
86. *R: It’s the same one that clients will be delayed because providers are busy with the other people.*
87. I: Okay. Now I want us to talk about family planning. Tell me what happens when a person comes to the VMMC clinic to access family planning?
88. *R: I have never come across such an issue*
89. I: As a peer what is your opinion on integrating Family planning in Voluntary Medical Male circumcision services/clinic?
90. *R: I think it can be good because it will help women to be on family planning.*
91. I: What is it that you would not like about integrating Family Planning services in VMMC?
92. *R: The problem can be that there will be too much work of the providers if they are to serve both clients that come for VMMC and family planning at the same time.*
93. I: How can we deal with such concern or barrier?
94. *R: I feel these should be separate services. There should be one space offering VMMC and another offering family planning.*
95. I: So they should not combine?
96. *R: I don’t think they should combine.*
97. I: Okay. Now let’s talk about cervical cancer screening. You mentioned that VMMC reduces the risk of cervical cancer in women right?
98. *R: Yes*
99. I: Tell me what happens when someone wants to get cervical cancer screening?
100. *R: Like how the screening is done?*
101. I: No just what happens or where they get the screening.
102. *R: I don’t know which hospital they go to but I just know that they go for cervical cancer screening.*
103. I: As a peer, what is your opinion on integrating partner cervical cancer screening with Voluntary Medical Male circumcision services
104. *R: I feel it can help women to get treatment earlier if they are diagnosed early.*
105. I: What is it that you would not like about this integration?
106. *R: [No response].*
107. I: How do you think your peers can view this?
108. *R: It can help more men to get VMMC.*
109. I: Mm, it can help men to get VMMC. What about what you don’t like about this?
110. *R: Long waiting hours for those that come for VMMC and too much workload for providers.*
111. I: Mm, how can we deal with such a barrier?
112. *R: Building more blocks where they can be offering VMMC on one side and cervical cancer screening on the other.*
113. I: Okay. Who should be screened for cervical cancer?
114. *R: Women who come with their men for VMMC.*
115. I: Alright. How about those that come alone?
116. *R: It should be everyone that comes for the service*
117. I: For family planning you said they should build another building. Now there are so many clinics that offer family planning. So when you said they should build another space do you mean they should increase the number of buildings at the VMMC clinic or build a completely new clinic for family planning?
118. *R: They should just increase the number of rooms at the VMMC clinic and offer family planning right there.*
119. I: So when should they offer family planning at the VMMC clinic?
120. *R: Whenever a woman comes with his partner to the VMMC clinic.*
121. I: And when should it be in the clinic flow? After they are done with the surgery or when they just come in or what time?
122. *R: When they test for HIV that’s when they should screen for STIs and offer family planning.*
123. I: How about screening for cervical cancer? When should it be done?
124. *R: they should do it at the end of everything.*
125. I: Okay so imagine you have come with your partner to the VMMC clinic. When is the best time for your partner to get cervical cancer screening so you can still get VMMC?
126. *R: Say you have done HIV testing and screening for STIs. While the man goes into surgery the woman can go for cervical cancer screening.*
127. I: Okay. Now let us talk about PrEP. Have you ever heard about PrEP?
128. *R: Yes.*
129. I: What do you know about it?
130. *R: That’s it’s a drug that is given to a raped girl or anyone who has had unprotected sex in order for them not to be infected with HIV.*
131. I: Oh okay. So what you have described is PEP. They sound alike but they are different because there is an “r” on PrEP. PrEP is an anti-HIV drug which helps to keep HIV-negative people negative. There is a pill that one needs to take every day to prevent HIV infection. It is almost the same as PEP only that with PrEP you have to take the pills daily.
132. *R: Oh okay.*
133. I: So how do you feel about PrEP?
134. *R: Like…?*
135. I: Do you like it or not?
136. *R: I feel like it’s a good drug because it can reduce the number of HIV infections in the country?*
137. I: [laughing] Now do you think it’s necessary for PrEP to be made available to HIV-negative men and women?
138. *R: Yes, I feel it’s necessary.*
139. I: Would you encourage someone to be taking the pills?
140. *R: Yes, I can encourage them.*
141. I: What reasons would you have to encourage someone to take them pills?
142. *R: I can tell them that this drug will help to boost your antibodies which will fight against the infection so they don’t get infected*
143. I: Mh. If PrEP become available, what is your opinion on integrating PrEP with Voluntary Medical Male circumcision services?
144. *R: It can be good because VMMC only reduces the risk of infection by 60% so with PrEP the risk can be better reduced [laughing].*
145. I: Mm... Okay it can reduce the risk. How do you think PrEP would be offered in this clinic?
146. *R: It can be offered to those that have finished their supply of PrEP.*
147. I: Mm... So let’s say for those that are just starting on the drugs. When should they be offered PrEP?
148. *R: After they have done HIV testing and they are negative then they can be offered the pills.*
149. I: Should they get the pills right in the HTC room?
150. *R: Yes.*
151. I: What would be the concern or barriers for this integration?
152. *R: Some people may accept it while others cannot.*
153. I: Why would other people not accept it?
154. *R: It’s because they may be having unprotected sex and not take the pills for some time, so when they get infected they can be angry but it’s all because they were not taking the pills regularly.*
155. I: So how can we deal with such a concern?
156. *R: Encouraging them to be taking the pills daily so they don’t get infected.*
157. I: Now let’s talk about other services. If you were given powers to choose and integrate services in Voluntary Medical Male Clinics, what are the services that you would think of Integrating?
158. *R: I can opt for providing PrEP for those that are HIV negative.*
159. I: Mm, why have you chosen PrEP?
160. *R: [laughing]It’s one of the methods which the youth can be using if they don’t have condoms readily available.*
161. I: SO they can just be taking PrEP instead?
162. *R: Yes*
163. I: Alright. Thank you very much for your time. Your answers will be very helpful in improving the health service delivery at circumcision clinics.
164. *R: Mm, thank you.*
165. I: Before we close, is there anything more you would like to say?
166. *R: I feel we have already talked about a lot of things.*
167. . I: Okay. Thank you very much for talking to me today.
168. *R: Thank you.*

END

**D 43 STUDY**

**Date of Interview: 10 August 2018**

**Type of Participant: Peer or Clinic Aid**

**Interview Number: D-43-0018**

**Interviewer: I. N.**

**Total Interview Time: 34 minutes 45 seconds**

**Interview Summary:** **(from summary sheet)**

| **SERVICE TO BE INTERGRATED** | **THOUGHTS ON INTERGRATION** |
| --- | --- |
| Couple HIV Testing and Counseling | Thinks it’s a good idea. Will help to reduce conflicts between partners which comes when there are some doubts. |
| STI Services | Thinks it’s a good idea. Will be provided with things to protect themselves |
| Family Planning | Thinks it’s a good idea. Will help reduce the rate of children and protect from some diseases. |
| Cervical Cancer Screening | Thinks it’s a good idea. Will reduce the rate of women with cervical cancer screening. |
| PrEP | Thinks it’s a good idea. It will reduce the spread of HIV Infection. |
| Other Services | Cervical cancer screening and PrEP |

**Remarks:**

**Participant was a bit shy and needed a lot of clarifications for questions. He saw no obstacles or concerns with integration of all services with VMMCC services.**

**Interview Texts:**

1. I: So… Thank you for taking the time to talk with me today.
2. *R: Yes.*
3. I: I would like to ask you some questions today about the way you feel and what you think about some issues related to the service you provide and how we can include other services in Voluntary Medical Male Circumcision (VMMC) clinics
4. *R: Yes.*
5. I: There are no right or wrong answers to these questions
6. *R: Yes.*
7. I: We would like to hear your opinion and your experiences in your own words. Do you have any questions before we begin?
8. *R: No, there is no question.*
9. I: Can you tell me how you are involved in the client care at this clinic?
10. *R: We do take part on encouraging our friends for circumcision, its benefit at school.*
11. I: Aaah... does your peers talk to you about how the services are provided here?
12. *R: They do talk to us.*
13. I: Can you give me an example of a time that your Peer talked to you about the services he received here?
14. *R: Yes*
15. I: Give me an example of what they said concerning the service they received at circumcision clinic.
16. *R: Yes, they received clothes and also drinks of different kinds*
17. I: okay… before circumcision or after?
18. *R: After circumcision*
19. I: Okay but with how the circumcision service works, like complication with how they were circumcised…
20. *R: No, there is no any complication which they said.*
21. I: Okay. Have you circumcised?
22. *R: Yes.*
23. I: When?
24. *R: I circumcised last year*
25. I: What happens for you reach the point of circumcision? How did it go?
26. *R: We went to the clinic. We were given a form. We signed it. They get us tested. After being tested, we went to receive circumcision.*
27. I: Ehm… so, let us talk about HIV testing as a couple at circumcision clinic. As a man is coming for circumcision, his female partner should also be coming along in the process, as they do test blood right [for HIV]?
28. *R: Yes, they do test blood*
29. I: Yes, so tell me, what happens if a man brings a spouse here at the Voluntary Medical Male circumcision clinic?
30. *R: Okay, when a man brings his spouse here, it works well*
31. I: How does it work like?
32. *R: Okay… mh….*
33. I: Feel free, what happens, how it works like, if a man is coming for blood test like HIV. Have you ever seen a man coming with his partner for testing here at circumcision clinic?
34. *R: Mm… no.*
35. I: You haven’t seen any?
36. *R: Yes.*
37. I: Or did you heard somewhere, how it works?
38. *R: No I haven’t*
39. I: You haven’t
40. *R: Yes.*
41. I: Mm…. What do you think are the motivators that make the men bring their spouses here for testing?
42. *R: That…. They do want that, both of them should health status… without doubting.*
43. I: Without doubting?
44. *R: Yes, without doubting.*
45. I: What do you think demotivates men to bring their partners here for HIV counselling and testing?
46. *R: Okay… they want that, if they have been found with HIV, they shouldn’t tell their wife, they should hide it.*
47. I: They should hide it right?
48. *R: Yes.*
49. I: What do you think can be done to make men bring their partners here for couple testing and counselling?
50. *R: What is needed is to encourage them*
51. I: To encourage them in what way?
52. *R: Encouraging both of them to be knowing their status so that there is no one found doubting the other.*
53. I: How do you think we should encourage them?
54. *R: Okay… hmmm…*
55. I: Or how should we do it to encourage them? Just give me an example of how you think we can encourage them
56. *R: In my own thinking, conducting meeting were parents or guardians have been called and explain to them.*
57. I: Mm, you as a peer, what is your opinion on integrating couple counseling with Voluntary Medical Male circumcision services?
58. *R: Repeat the question…*
59. I: You as a person who influence others to come and do circumcision, what is your opinion on integration, because we want to integrate couple HIV testing here at circumcision clinic, so about that service of testing HIV as a couple, what do you think? How do you see it?
60. *R: It is a good idea*
61. I: It is a good idea?
62. *R: Yes.*
63. I: Why do you see it as a good idea?
64. *R: Okay… mm… the question has destructed me.*
65. I: You have said that integration couple HIV testing, as a man is coming for circumcision, his female partner should also be coming as they test for blood, that should be done together as a couple, you said it is a good idea.
66. *R: Yes.*
67. I: So why do you think it is a good idea?
68. *R: It is a good idea that both of them should be knowing their health status, if one has been found with a virus, should start the treatment right away that both should be protecting themselves but also to protect his wife.*
69. I: Okay. Mh…. What do you think are the barriers and concerns on this integration?
70. *R: What?*
71. I: What do you think are the barriers and concerns on this integration? Barriers or concerns which you see… can come
72. *R: Ah, I don’t see any concerns or barrier*
73. I: Aaah, Now I would like to discuss with you about sexual reproductive health services and Pills HIV prevention: called pre-exposure prophylaxis. (PrEP). Sexual reproductive health includes services that promote good sexual health and reproduction. They include but not limited to family planning, sexual transmitted infection management, cervical cancer screening, Condom distribution and many more. Today we will only discuss about family planning, diagnosis and management of STIs, Cervical cancer screening, and PrEP. We will look at each of these one by one. Let us start with STI services. So, explain to me what happens if a client is suspected or diagnosed with an STI here?
74. *R: Okay, if a person is found with STI, they do not circumcise that person and he go back home.*
75. I: He go back home?
76. *R: Yes.*
77. I: So, doesn’t he receive any service?
78. *R: No. He doesn’t the service pertaining to circumcision*
79. I: He doesn’t receive?
80. *R: Yes.*
81. I: Do they gave him drug treatment relating to the diseases found?
82. *R: Yes, they do give him drug treatment.*
83. I: If he went home, do they call him back for circumcision or he went for good?
84. *R: He come back*
85. I: He come back?
86. *R: Yes*
87. I: You as a peer what is your opinion on integrating STI services with circumcision services? That it should be like they have included, when you come for circumcision, so that they should also be screening you for STI and helped if found. What do you think about that?
88. *R: Our opinion, it is good.*
89. I: Is it good?
90. *R: It is good*
91. I: You saying that it is good, what are the things which have made you say it is good or why do you think it is good?
92. *R: Okay, because …. Hmmm... the question [laughing]*
93. I: Ehm, here we were saying, they want to integrate STI services in the process of circumcision services, as you come for circumcision, they should also be screening you and give you STI service
94. *R: Yes.*
95. I: So, you have said it is a good thing
96. *R: Yes.*
97. I: So, I have asked that, why do you see it as a good thing of integrating STI services
98. *R: Okay, it good for saying that when you are found with STI, they will be providing us things for protection.*
99. I: Things for protection like what for example?
100. *R: Okay, like condoms.*
101. I: Ah, what is it that you would not like the integration of diagnosis and management STIs with Voluntary Medical Male Circumcision services?
102. *R: Ah nothing there.*
103. I: Nothing?
104. *R: Nothing.*
105. I: How do you think STI services should be offered at the Voluntary Medical

Male circumcision clinic?

1. *R: The question [repeat]…*
2. I: How do you think STI services should be offered at the Voluntary Medical Male circumcision clinic? You know the circumcision process right? As you come for circumcision what is the first thing that you do?
3. *R: First, they give us a paper to sign*
4. I: After signing, then
5. *R: Then, you go for circumcision*
6. I: Then…
7. *R: They gave us a prescription paper*
8. I: Prescription paper, then
9. *R: Ah, mm….*
10. I: You go home?
11. *R: Yes, we go home, then we come back for checkup with doctors*
12. I: In the circumcision process, where do you think STI services should be offered?
13. *R: Okay… hmmm*
14. I: Where does it have to be place? On registration, screening for diseases, during

circumcision, after circumcision? Where exactly?

1. *R: Okay, on registration*
2. I: During registration that’s where STI should
3. *R: Screened first for STI…*
4. I: Then…
5. *R: [Laughing]…*
6. I: Okay, should this service, be provided within the VMMCC, within VMMCC

but in a private room or outside?

1. *R: Within VMMCC but in private room.*
2. I: Why in the same clinic and not outside that maybe we should be going outside
3. *R: Okay, for the sake of confidentiality.*
4. I: If it is outside, does it mean it will not be privacy?
5. *R: [Laughing] no, everyone will know.*
6. I: Okay, will know that, you are going to be screened for STI?
7. *R: Yes, diseases.*
8. I: Okay. What do you think are the barriers and concerns on this integration?
9. *R: There is no barrier.*
10. I: Nothing...
11. *R: Yes.*
12. I: Ah, let us talk about family planning. Have you ever heard about family planning?
13. *R: Yes.*
14. I: What did you heard about family planning?
15. *R: Okay, I heard about women family planning and men family planning*
16. I: Okay. How does women family planning works like?
17. *R: They go to the hospital and receive an injection for family planning*
18. I: Okay. How does men do family planning
19. *R: They also go to the hospital and receive an injection for family planning*
20. I: To, explain to me what happens if a client needs a family planning methods? How does it work?
21. *R: There, I have never inquired how it works*
22. I: Ah, you as a peer, what is your opinion on integrating Family planning in circumcision services? …Not that they should only conducting circumcision but also providing family planning service. How do you see it… what would you like about that?
23. *R: Okay, it is a good idea to integrate… family planning*
24. I: Why do you think it is a good idea?
25. *R: Okay, we see it as a good idea because…. Hmmm…hmmm…*
26. I: Or how would you like it to integrate family planning services
27. *R: We would like it because… it would help to reduce different kind of diseases*
28. I: Diseases, in what way?
29. *R: Which you get through sexual intercourse*
30. I: If you are doing family planning does it mean you cannot get STI?
31. *R: Mh, you can get… mm…*
32. I: Or when they say family planning, how do you understand it?
33. *R: Family planning, I see it as having a limitation in number of children to have.*
34. I: Yes.
35. *R: So that the number of children should not increase*
36. I: So, those, if they have to integrate family planning that men should be able to do family planning and women should also do family planning here at circumcision clinic, you said it is a good thing right?
37. *R: Yes, it is a good thing*
38. I: Yes, so how is it good that men should be receiving family planning at this clinic? In what way
39. *R: Okay, it is good because … ehm [laughing] that question*
40. I: You have explained about family planning, so I asked you, if family planning has to be integrated at circumcision clinic, you said it is a good thing, so I said, it is good in what way because when a person you are saying that it is good, there is a reason for saying why it is good. So why is it good?
41. *R: It is good because people should not be having more children, they should be having a limit for having children.*
42. I: Okay, as we were saying in circumcision process, where should this service placed? When a person come for circumcision, where should it be offered?
43. *R: Of family planning?*
44. I: Yes and when?
45. *R: Mm…. The time when we are getting circumcised*
46. I: The time when you are being circumcised, that’s when you should be receiving family planning services?
47. *R: Yes…*
48. I: Alright. What do you think are the barriers and concerns with the integration family planning and Voluntary Medical Male circumcision services?
49. *R: There is no barrier.*
50. I: There is no barrier…
51. *R: Yes.*
52. I: So the integration can just work well?
53. *R: Yes.*
54. I: Let us discuss about cervical cancer screening for female partners.
55. *R: Yes.*
56. I: Explain to me what happens if a woman needs cervical cancer screening?
57. *R: Mh, that I have never enquired [laughing]*
58. I: Or have you heard about cervical cancer screening?
59. *R: No, I just heard but I have never asked how it works*
60. I: What did you heard?
61. *R: I was just hearing, there is cancer, cancer of cervix, that makes women not to deliver*
62. I: Mm, so as a peer, what is your opinion on integrating cervical cancer screening for female partners with Voluntary Medical Male Circumcision services?
63. *R: It is good… because it helps protect from different diseases*
64. I: Diseases like what?
65. *R: HIV and AIDS and the cancer*
66. I: Mm…. So, what is it that you would not like about the integration of partner cervical cancer screening with Voluntary Medical Male Circumcision services?
67. *R: Which can resist us?*
68. I: Which you would not like
69. *R: We will be having fear that if they screen us… let me just say that many people do have fear that, if they go to a place where they do screen and found with diseases which you get through sexual intercourse, if they send them back, they do feel bad, that their friend will be laughing at them that they have been sent back, their friends will be laughing that, that one has diseases. So many people do feel bad*
70. I: Mh, but as what we are talking about here, it involves cervical cancer screening, yea, if we integrate this service, what will you make not to like integration like at this clinic, they should not integrate this service. What is it?
71. *R: Ah, nothing.*
72. I: Nothing.
73. *R: Yes.*
74. I: How do you think is the best way to offer cancer screening within Voluntary Medical Male circumcision clinics?
75. *R: Repeat the question*
76. I: How do you think is the best way to offer cancer screening within Voluntary Medical Male circumcision clinics? What are the best way for offering this service?
77. *R: Mh,…the question is difficult*
78. I: Or we should say, this service, when should it be offered in circumcision process, where should it be placed? When you come for circumcision, so that women could also be screened for cervical cancer, when should it be offered?
79. *R: The time people are being circumcised*
80. I: The same time men are being circumcised and the women should also be screened?
81. *R: Yes.*
82. I: So where should it be placed?
83. *R: Okay… the place should not be where circumcision is taking place but somewhere else.*
84. I: Somewhere else?
85. *R: Yes.*
86. I: Where a woman should be screened?
87. *R: Yes.*
88. I: In the within clinic or outside?
89. *R: No, within the clinic.*
90. I: Okay, but in different room
91. *R: Yes, different room*
92. I: Mm… what do you think are the barriers and concerns about integrating partner cervical cancer screening and Voluntary Medical Male Circumcision services?
93. *R: There are no concerns.*
94. I: Or barriers?
95. *R: There are no barriers.*
96. I: Ah, let us now talk about PrEP. Have you ever heard about PrEP?
97. *R: No.*
98. I: Or PEP…?
99. *R: Aaah, I just heard about the words*
100. I: what do you hear?
101. *R: PEP, I don’t have a clear history about it*
102. I: so just heard about PEP or PrEP…?
103. *R: Yes. So I do ask to my friends.*
104. I: So, if you have never heard about PrEP, I will explain how the medicine works. PrEP is anti-HIV medicine that keeps HIV-negative people from being infected. If you don’t have HIV and you taking PrEP, you cannot get a virus if you slept with some who is positive. There is a single pill that is taken once daily, and if you take it regularly, it is highly effective at prevention people from being infected. So, if a person asks you about PrEP, how can you explain it?
105. *R: Okay, are medicine that protect, even if you slept with someone who is positive not to get it.*
106. I: Okay. Now, how do you feel about PrEP? Or If PrEP was made available to HIV- men and women. Do you think you could advise your HIV negative peers to accept to take PrEP?
107. *R: No.*
108. I: It should not be available
109. *R: Yes.*
110. I: It should be found to only people with HIV…
111. *R: HIV.*
112. I: Why?
113. *R: [Laughing]… No, it should be available to people who are negative.*
114. I: only? Or I should ask that, who should receive the medicine?
115. *R: It should be available to all.*
116. I: Those with HIV and those without?
117. *R: Ah, but it should be to those who do not have… HIV [negative]…*
118. I: Those without HIV?
119. *R: Yes.*
120. I: Alright. Aaah, what are the reasons you would encourage your clients to take PrEP?
121. *R: Ah, the first reason of taking this medicine is that, if you take this medicine and you slept with someone who is positive, you cannot get the disease.*
122. I: Alright…
123. *R: You cannot get disease.*
124. I: Another reason?
125. *R: Another reason, it is because it will help to reduce the spread of HIV infection*
126. I: Addition?
127. *R: No that’s all.*
128. I: If PrEP becomes available, what is your opinion on integrating PrEP with Voluntary Medical Male circumcision services?
129. *R: Repeat…*
130. I: If PrEP becomes available, what is your opinion on integrating PrEP with Voluntary Medical Male circumcision services? What do you think, if PrEP has to be available and integrate it with service provided at circumcision clinic?
131. *R: It will be a good thing.*
132. I: So, how do you think PrEP would be offered here at Voluntary Medical Male Circumcision clinic?
133. *R: Ah, it should be offered to anyone who have received circumcision*
134. I: Everyone who have received circumcision?
135. *R: Yes.*
136. I: Mh, who should be providing this service?
137. *R: Doctors.*
138. I: Those who provide medicine?
139. *R: Yes, or even nurse.*
140. I: What do you think could be the concern and barriers to integrating PrEP Voluntary Medical Male Circumcision services?
141. *R: Nothing.*
142. I: Nothing?
143. *R: Yes.*
144. I: Mhm… [clearing throat]. Let us now talk about other services. If you were given powers to choose and integrate services in Voluntary Medical Male Clinics, what are the services that you would think of to Integrate?
145. *R: Cervical cancer screening.*
146. I: Only that?
147. *R: And PrEP.*
148. I: Any other service?
149. *R: No that’s all*
150. I: You have chosen cervical cancer screening and PrEP?
151. *R: Yes.*
152. I: Why have you chosen those two? Explain to me.
153. *R: PrEP, will help to reduce the spread of HIV but also not to get HIV infection.*
154. I: While cervical cancer screening
155. *R: Okay, will help more women not to get cervical cancer, it going to be reduced*
156. I: Ah, how do you think these services would be offered in the clinic? The services of cervical cancer screening and PrEP, how should it be offered? Let us start with how PrEP should be offered at circumcision clinic
157. *R: It should be offered when people are being circumcised*
158. I: When are being circumcised?
159. *R: Yes.*
160. I: Before circumcision or after circumcision?
161. *R: After circumcision.*
162. I: Just after circumcision?
163. *R: Yes.*
164. I: So, about… cervical cancer screening, how should this service be offered?
165. *R: Anytime*
166. I: anytime?
167. *R: Yes.*
168. I: Thank you for taking your time to discuss with me today.
169. *R: Yes.*
170. I: Your answers will be very helpful in improving the health service delivery at Voluntary Medical Male circumcision clinics.
171. *R: Yes.*
172. I: Do you have a question?
173. *R: There is a question.*
174. I: Yes, ask…
175. *R: We would like to know; does circumcision protect 100%?*
176. I: 100% from HIV?
177. *R: Yes…*
178. I: *No, it is not 100% because it happens that you did circumcision and you are having unprotected sex. It happens that during sex, there were some friction and both of you have been injured, and there is a blood contact. If your partner has a virus, she can give it to you.*
179. *R: Which mean if you slept unprotected with someone who is positive without bruises you cannot get a virus?*
180. I: Mh… there am not sure but it depends, but your life is at risk, you can get a virus. When they say circumcision, it doesn’t mean you cannot get diseases, you can still get.
181. *R: Okay. That’s the only question.*
182. I: Thank you.

*END*

**D 43 STUDY**

**Date of Interview: 10 August 2018**

**Type of Participant: Peer or Clinic Aid**

**Interview Number: D-43-0019**

**Interviewer: I.N.**

**Total Interview Time: 39 minutes 31 seconds**

**Interview Summary:** **(from summary sheet)**

| **SERVICE TO BE INTERGRATED** | **THOUGHTS ON INTERGRATION** |
| --- | --- |
| Couple HIV Testing and Counseling | Thinks it’s a good idea. It will reduce the spread of HIV/AIDS. |
| STI Services | Thinks it’s a good idea to integrate because it can help people not to put their life at risk. |
| Family Planning | Thinks it is a good idea. It will reduce the rate of new born babies from different diseases. |
| Cervical Cancer Screening | Thinks it’s a good idea. It will benefit both men and women. |
| PrEP | Thinks its good idea. Also thinks it will protect people from STIs. |
| Other Services | PrEP, Condoms and Couple HIV testing. |

**Remarks:**

**Participant was relaxed, confident, and knowledgeable. He was going of point and needed amplifications.**

**Interview Texts**:

1. I: Ah, thank you for taking the time to talk with me today. I would like to ask you some questions today about the way you feel and what you think about some issues related to the service you provide and how we can include other services in Voluntary Medical Male Circumcision (VMMC) clinics. There are no right or wrong answers to these questions. We would like to hear your opinion and your experiences in your own words. Do you have… any questions before we begin?
2. *R: No question.*
3. I: [Clearing throat] … Can you tell me how you are involved in the client care at this clinic?
4. *R: Okay, for encouraging other people about the good thing of circumcision and the benefit of circumcision and the good results which may come after circumcising*
5. I: Ah, does your peers talk to you about how the services are provided here?
6. *R: They could talk*
7. I: Maybe when they come for circumcision, can you give me an example of a time that the Peer talked to you about the services he received here?
8. *R: By the time he received circumcision service, he was able to tell the good thing of circumcision by saying that circumcision has helped protect them from different diseases than before they got circumcised as they could see some blisters at the head of their penis, but since circumcision, it has not happened again.*
9. I: Are you circumcised?
10. *R: Yes, am circumcised*
11. I: What happened to be circumcised? When did you circumcise?
12. *R: I circumcised in 2011, august.*
13. I: Did you came alone or you were told by someone to come?
14. *R: I was encouraged by my friend who has also done the thing [circumcision], and I saw it as a good thing… I should say like that*
15. I: Upon reaching here, how did it go to be circumcised?
16. *R: Before circumcision, they take me for blood testing, they want to see your blood status whether you are positive or negative from AIDS diseases. So they take me and I was found negative. Then I was allowed to go for circumcision because when you are found to be positive, sometimes it happen that they do not allow you for circumcision.*
17. I: Alright, [clearing throat], let us talk about Couple HIV testing
18. *R: ehm*
19. I: Man and woman who has come to test for HIV as a couple when they come at circumcision clinic. So, tell me what happens if a man brings a spouse here at the Voluntary Medical Male circumcision clinic?
20. *R: What happens is… when a man come with his partner, they do encourage one another. First before they come they do encourage one another for blood testing so that they could know their immunity in their bodies. By also wanting to be encouraging one another in the issues of their relationship. So when they come here, they encourage one another for testing to know how their body immunity is working like… for the sake that one may have diseases and can pass to the partner. So they want to know that, in case one knows that has infection, can find ways of how to protect themselves or health service providers, they could guide you find ways of how you can protect yourself so that the other cannot get infection.*
21. I: Okay, have you ever seen a man coming at a circumcision clinic with his partner?
22. *R: Yes, I have seen it*
23. I: Okay, what do you think are the motivators that make the men bring their spouses here for testing?
24. *R: By wanting to know their immunity system but also what happens when there are some trust issues, which makes them to come together and test for HIV and AIDS disease.*
25. I: What do you think demotivates men to bring their partners here for HIV counselling and testing?
26. *R: By not trusting each other in a family*
27. I: What do you think can be done to make men bring their partners here for couple testing and counselling?
28. *R: Ah, by introducing programs which can encourage families on issues of testing HIV and AIDS and other forms of print Medias were people could be able to read that they can come and test for HIV and AIDS disease.*
29. I: You as a peer, what is your opinion on integrating couple counseling with Voluntary Medical Male circumcision services?
30. *R: can you repeat the question*
31. I: Mm… you as a peer, who help other to come here and do circumcision right?
32. *R: Yes.*
33. I: What is your opinion on integrating couple counseling with Voluntary Medical Male circumcision services? How do you see it? Or what do you think?
34. *R: On issue of circumcision?*
35. I: Yes, and they have to integrate couple HIV testing, how do you see it?
36. *R: Okay, after testing, a man, even if he has done circumcision, he can still get HIV, so there is still need to integrate by encouraging them that even though you have circumcised, it does not mean you have to be reckless, sleeping with other people unprotected. And also you need to follow the prescription given during circumcision.*
37. I: So here they want to integrate a service for couple HIV testing, a man and his spouse coming for testing, what is your opinion on integrating that service at circumcision clinic? How do you welcome it?
38. *R: It is a good thing because that service can help by saying that diseases should not spread*
39. I: In what way?
40. *R: The question is difficult*
41. I: Or how can the disease not spread if the man is coming with his spouse at circumcision clinic?
42. *R: Oh, in the way that they can be people who encourage and trust one another when doing things*
43. I: Ah what do you think are the barriers and concerns on this integration?
44. *R: The concern is that when they come as a couple for testing, if one is found to be positive, the other is negative, it is a time when things, when family complications can start*
45. I: Mm, what do you think can be done to overcome the concern you mention so that people can be coming as a couple for testing at circumcision clinic?
46. *R: There is need to come up with strategies that can help encourage people on issue when one is found with AIDS and the other do not have. There is a need to encourage them as it can happen that one has AIDS and the other do not have, it can happen that the family can still be there for a long time, the other partner without AIDS and not sharing it, by giving them guidance on strategies to follow so that they cannot share the disease.*
47. I: Can you mention one of the strategies for instance
48. *R: strategies…?*
49. I: Which should be put in action. Which can be used to address this concern
50. *R: Okay, we can give them advice by saying that, that is not the end of life and also saying that if you are found with AIDS it does not mean that the family should end, it should not make family to end. They should also give them things which they can use to protect themselves by the time when one is found to be negative and the other positive for the fear that they might share the disease between them.*
51. I: Okay. Now I would like to discuss with you about sexual reproductive health services and Pills HIV prevention: called pre-exposure prophylaxis. (PrEP). Sexual reproductive health includes services that promote good sexual health and reproduction. They include but not limited to family planning, sexual transmitted infection management, cervical cancer screening, Condom distribution and many more. Today we will only discuss about family planning, diagnosis and management of STIs, Cervical cancer screening, and PrEP. We will look at each of these one by one. Let us start with STI services as a service which they want to integrate. So explain to me what happens if a client is suspected or diagnosed with an STI here?
52. *R: Ah, when a person come for circumcision, by being suspected or to say that he has been found with STI, they could counsel him, by the time he has been found with infection, they could tell him to go and receive treatment of the STI found with, but he also sends back home.*
53. I: Alright. You as a peer what is your opinion on integrating STI services with circumcision services?
54. *R: Okay. If a person got circumcised this help not to get AIDS diseases. It just helps, it doesn’t mean that by the time you have circumcised you cannot get AIDS, and it just reduce to say that you can get AIDS disease.*
55. I: Okay, if we integrate at circumcision clinic, that people could be able to be screened for STIs and given treatment, how do you see it? Would you like the idea?
56. *R: I would like it because aaah by the time you have integrate at a place where people receive circumcision or to say treatment, it can really help them. It can help people not to put their life at risk.*
57. I: Okay. What is it that you would not like the integration of diagnosis and management STIs with Voluntary Medical Male Circumcision services?
58. *R: For forcing someone to go for circumcision and also for forcing someone to… be ... able to test… for diseases. This could make a person to have worries in with his body*
59. I: Okay. How do you think STI services should be offered at the Voluntary Medical Male circumcision clinic?
60. *R: By having a place or people who can be giving counselling to people who came for test, about the good thing for testing but also for strategies which can be followed when found with diseases and also which can be followed when do not have diseases so that can live a protected life.*
61. I: Where exactly do you think this clinic should be situated at circumcision clinic? Within the clinic, outside, private rooms or how?
62. *R: It is supposed to be within the clinic because by the time you are being circumcised, it happens that there is a problem to say doctor or nurse. This can help as his colleague doctors with necessary experience can help the doctor or that nurse of the problem committed on duty like when he is conducting circumcision procedure. Even for that person, it can be easy to take him to the hospital and you can be just moved to the other compartment within the clinic, that being far.*
63. I: So, when is the person supposed to screen for STI in the circumcision process?
64. *R: By the time when he has tested but before circumcision, that is when he is supposed to be screened*
65. I: Okay. What do you think are the barriers and concerns on this integration?
66. *R: Ah… the barriers are there*… can you ask again
67. I: the barriers are there, what can the barriers be on integrating STI services?
68. *R: Ah, the barriers is that, there are other people who when heard about a thing, they do not have a heart of accepting it or to tell others. There are other people who have hearts that when a thing has been put in place, they do not like it to keep on working.*
69. I: So, what do you think should be done to address these concerns and barriers?
70. *R: Encourage one another about the good thing of this integration.*
71. I: How can you encourage each other?
72. *R: Through different programs in areas… where we are staying… by encouraging them about this thing. But also through different trainings which can be conducted to let people know the benefit of integration.*
73. I: Yes, okay. Let us talk about family planning. Explain to me what happens if a client needs a family planning methods?
74. *R: When a person is in need of family planning method, is supposed to go to the hospital. Before going to the hospital, it is necessary to first know about the goodness of family planning by the time is in a marriage or not.*
75. I: Mm... when they say family planning, what does it mean?
76. *R: Ah, family planning… is one of the strategies… which were put in place to or we can say… it is a way whereby a person can take time without having children.*
77. I: So, there are ways for family planning right, others say methods of family planning. Which methods do you know or which ways?
78. *R: Ah, I know family planning method of using… pills. But there is also another way of family planning of using condom, by the time you are having sexual intercourse. Because condom is one way of protection that a woman should not conceive.*
79. I: So, as a peer, what is your opinion on integrating Family planning in circumcision services?
80. *R: Ah, on the integrating family planning, this will help that we should be able to prevent the number of people who might be having infections as new born babies. If might be found that, those people if they do not know family planning method, children might be getting pregnancy. By the time of getting pregnancy, both children might have STIs. So, this may make the rate of when a woman is pregnant and when the child is born, it becomes difficult not to be born with disease. So it can make the rate of diseases to increase.*
81. I: So, would you like it? [integration]
82. *R: Yes, I would like it.*
83. I: Mh. What is it that you would not like about the integration of family planning with Voluntary Medical Male Circumcision services?
84. *R: What did you ask?*
85. I: At first, I asked you, what you would like on integration of family planning with circumcision services.
86. *R: Okay.*
87. I: So you were explaining like the goodness of it right?
88. *R: Yes.*
89. I: And also if you can like…
90. *R: And I have accepted…*
91. I: Now here am saying, what is it that you would not like about the integration of family planning with Voluntary Medical Male Circumcision services?
92. *R: Nothing.*
93. I: How do you think family planning services would be offered within Voluntary Medical Male circumcision clinics?
94. *R: On the men side, need to encourage men to use condom by the time they are having sexual intercourse, is one way. On the side women side, through coming to receive methods of family planning but also giving the medicine or services which can be able to help them on issues of family planning.*
95. I: So, when do you think this service should be offered in the circumcision process
96. *R: By the time when a person has chosen that he can receive family planning*
97. I: Mm, if a man has come for circumcision, when do you think he can receive this service?
98. *R: By the time he has recovered [healed]*
99. I: Mm… What do you think are the barriers and concerns with the integration family planning and Voluntary Medical Male circumcision services?
100. *R: My worries is that, this can promote youth to be having sexual intercourse sometimes unprotected sex but also between married partners, this can promote a behavior that, a man can be able to do other things like sexual intercourse on other side with the backup that he has received family planning, saying that he cannot impregnate, meaning that he cannot be caught.*
101. I: What do you think should be done to address these concerns and barriers?
102. *R: Encouraging men, that by the time they are receiving family planning or when they are following it, it is not time to be reckless, having unprotected sex with people who are not in marriage or not his wife. And also women, it’s not time to feel free and start sleeping with different people.*
103. I: Ah, okay. Let us now talk about cervical cancer screening for female partners. Explain to me what happens if a woman needs cervical cancer screening?
104. *R: I don’t know*
105. I: Have you heard about cervical cancer screening?
106. *R: Yes, I have heard*
107. I: What did you heard? As they say cervical cancer
108. *R: Okay, cervical cancer… damage of cervix. That’s what I heard*
109. I: As a peer, what is your opinion on integrating cervical cancer screening for female partners with Voluntary Medical Male Circumcision services?
110. *R: Cervical cancer for women?*
111. I: Yes.
112. *R: It can help in the way that…aaah, by the time they have been found with cancer, they can be helped in good time. And also by screening cervical cancer, it will them to know how their cervix is. But also on men circumcision, this help to reduce the chances of getting diseases by the time they are having sexual intercourse.*
113. I: *What is it that you would not like about the integration of partner cervical cancer screening with* Voluntary Medical Male *Circumcision services?*
114. *R: Mm… nothing*
115. I: How do you think is the best way to offer cancer screening within Voluntary Medical Male circumcision clinics?
116. *R: [laughing] ask again*
117. I: How do you think is the best way to offer cancer screening within Voluntary Medical Male circumcision clinics? Or we can say, when is a good time for this service to be offered in circumcision process.
118. *R: By the time men has go for circumcision, they should be able to motivate their partners for screening cervical cancer*
119. I: When should this happen according to the circumcision phases?
120. *R: When they are going for testing*
121. I: That’s when a woman should also be going for screening
122. *R: That’s when should be going for screening*
123. I: Both of them together or
124. *R: Both of them together. Since they are like family, they are supposed to be together when doing things*
125. I: So does the clinic supposed to be within circumcision clinic or outside?
126. *R: It is supposed to be at different places*
127. I: Why different places?
128. *R: Because screening cervical cancer is something different and circumcision is also different. So these things are supposed to be at different places and also because the work is different.*
129. I: So if a man brings his spouse, how will it be working like?
130. *R: If a man brings his spouse like*
131. I: Like where will they be going first? If clinics will be separate and not in one place, how will it be?
132. *R: They will start going to the women side for cervical cancer screening then they will be going together for circumcision*
133. I: Ah, what do you think are the barriers and concerns about integrating partner cervical cancer screening and Voluntary Medical Male Circumcision services?
134. *R: There are a lot of barriers for having inadequate resources for cervical cancer screening and also for having inadequate tools used for circumcision, when a man is being circumcised.*
135. I: Mm… What do you think should be done to address these concerns and barriers?
136. *R: On the first concern about inadequate resources for cervical cancer screening, can be addressed by working together as we contribute money to buy tools which can be used to screen women for cervical cancer and also for tools used for circumcision.*
137. I: Assuming all equipment’s are available for cervical cancer screening and at circumcision, the equipment’s are also available, what can be the concerns about integration of these two?
138. *R: The problem can be there for some people not understanding the issue of cervical cancer screening and circumcision. It is not everyone who can accept it*
139. I: Okay fine. So, the problem of not accepting it, how can we address it?
140. *R: By motivating them about the good thing of screening but also motivating them the good thing about circumcision for men.*
141. I: Now, let us talk about PrEP. Have you ever heard about PrEP?
142. *R: Yes.*
143. I: If you have heard about PrEP, please tell me what you know about PrEP?
144. *R: PrEP is medicine that help a person prevent from getting disease by the time they are having unprotected sexual intercourse.*
145. I: How did you learn about this?
146. *R: I heard about this through other people who know about this, who were able to use these medicine.*
147. I: How do you feel about this?
148. *R: It is a good thing because you can prevent diseases by the time you are having sexual intercourse but also the bad thing about these things, can promote youth or youth on sexual behaviors, because they will be thinking that if I slept with this one, I will be safe.*
149. I: What you have mention is PEP
150. *R: PEP right*
151. I: But there is PrEP. Have you ever heard about PrEP?
152. *R: I have never heard of PrEP*
153. I: Okay, if you have not heard about PrEP, I will explain how the medicine works. PrEP is anti-HIV medicine that keeps HIV-negative people from being infected. There is a single pill that is taken once daily, and if you take it regularly, it is highly effective at prevention people from being infected. Now, how do you feel about PrEP?
154. *R: It is good*
155. I: It is good?
156. *R: Yes…*
157. I: If PrEP was made available to HIV- men and women. Do you think you could advise your HIV negative peers to accept to take PrEP?
158. *R: It is necessary*
159. I: It is necessary in what way?
160. *R: This could protect men and women from contracting STIs because are medicine that help protect from getting STI.*
161. I: If PrEP becomes available, what is your opinion on integrating PrEP with Voluntary Medical Male circumcision services?
162. *R: When men received circumcision, that’s when he is supposed to receive the medicine [PrEP]. But also after circumcision, he is needed to be counselled about the medicine [PrEP], the goodness of this medicine.*
163. I: Would you encourage clients to take PrEP?
164. *R: Yes*
165. I: Why?
166. *R: Because I could be one of them helping people to be protected from STI*
167. I: How do you think PrEP would be offered here at Voluntary Medical Male Circumcision clinic?
168. *R: By the time when men has come for circumcision and also when they come with their wives for instance antenatal clinic or when she is expecting, that’s when they can be coming to be counselled and receive these medicine.*
169. I: But like circumcision clinic, what time could PrEP be given?
170. *R: By the time they have come for circumcision as a couple, they are supposed to receive these medicine.*
171. I: Which kind of people are supposed to receive PrEP?
172. *R: Those who have circumcised and those that have not circumcised.*
173. I: What do you think could be the concern and barriers to integrating PrEP Voluntary Medical Male Circumcision services?
174. *R: The barrier is that other people they cannot trust those kind of medicine*
175. I: Why do you think in that way that it cannot be trusted? It cannot be trusted in what way?
176. *R: That one is difficult*
177. I: Or I should ask that, what reasons can make them not to trust the medicine?
178. *R: Some people cannot trust those medicine on the cultural beliefs of parents but also religious beliefs, they cannot accept to receive these medicine*
179. I: What do you think should be done to address these concerns and barrier?
180. *R: Need to establish organization that can motivate or print Medias that can be able to write the goodness of using these medicine.*
181. I: Alright, let us go to other services. If you were given powers to choose and integrate services in Voluntary Medical Male Clinics, what are the services that you would think of to Integrate?
182. *R: The other services by motivating boys or men who have not yet circumcised*
183. I: On other services which we have already talked about like PrEP…
184. *R: I can choose PrEP*
185. I: Any other? That what you can choose to integrate
186. *R: The other service is using condoms when having sexual intercourse*
187. I: Is there any other service?
188. *R: Couple HIV testing*
189. I: So you have chosen condoms
190. *R: PrEP, couple HIV testing, that’s all.*
191. I: Why have you chosen about condoms?
192. *R: Because condom are the things which cannot be hard to find but also are the things which are found in many places*
193. I: You have also mentioned of PrEP right?
194. *R: Yes*
195. I: Why have you chosen PrEP? To integrate with…
196. *R: PrEP is not difficult to follow when you have chosen it*
197. I: You also mentioned the last one, what was it?
198. *R: Testing [couple]*
199. I: Yes couple testing
200. *R: Couple testing will help as a family, since all will be protected after knowing their status.*
201. I: How do you think the services you have chosen should be offered at the clinic, starting with condom
202. *R: Condom should be offered when a man has come for circumcision and also by encouraging them that when they come here at the clinic, they should be getting condoms, so that they can be using them at the time they want to have sexual intercourse.*
203. I: When they are leaving for home?
204. *R: Yes.*
205. I: So for PrEP, how should this service be offered at this clinic or when?
206. *R: Okay, maybe you should just change at the clinic*
207. I: Yes, how should it be offered at this clinic?
208. *R: By the time a man is coming for circumcision or when he has just volunteered to just come and get the medicine.*
209. I: Okay, how about couple HIV testing. How should this service be offered at the clinic?
210. *R: By the time when a person has accepted it for testing and also by the time when he comes as a couple for cervical cancer screening.*
211. I: Thank you for taking your time to discuss with me today. Your answers will be very helpful in improving the health service delivery at Voluntary Medical Male circumcision clinics.
212. *R: Thank you.*
213. I: Before we close, do you have anything to say?
214. *R: No.*
215. I: Again thank you so much.
216. *THE END*

**D 43 STUDY**

**Date of Interview: 11 August 2018**

**Type of Participant: Male Index Participant**

**Interview Number: D-43-0020**

**Interviewer: I N**

**Total Interview Time: 38 minutes 50 seconds**

**Interview Summary:** **(from summary sheet)**

| **SERVICE TO BE INTERGRATED** | **THOUGHTS ON INTERGRATION** |
| --- | --- |
| Couple HIV Testing and Counseling | A good opportunity for couples to test together. |
| STI Services | Thinks STI services would benefit those that live far away from normal hospitals to be able to access the service at the VMMC clinic which may be closer to their homes. |
| Family Planning | An advantage for peers to talk more to clients about family planning |
| Cervical Cancer Screening | Men will know whether their partner has the cancer before they get VMMC.. |
| PrEP | A good idea as this integration would help to fight HIV better. Only concerned that people would not protect themselves. |
| Other Services | Thinks cervical cancer screening and PrEP are the best for the integration. |

**Remarks:**

**Participant was relaxed and had a good sense of humor. He easily understood the questions and was very open-minded.**

**Interview Text:**

1. I: Thank you for taking the time to talk with me today.
2. *R: Thank you.*
3. I: I would like to ask you some questions today about the way you feel and what you think about some issues related to the service you receive here and how we can include other services in Voluntary Medical Male Circumcision (VMMC) clinics.
4. *R: Alright.*
5. I: There is no right or wrong answers to these questions. We would like to hear your opinion and your experiences in your own words. Do you have any questions before we begin?
6. *R: No I don’t have any, but maybe at the end of this discussion.*
7. I: Alright. What role do you take in Voluntary Male Medical Circumcision services?
8. *R: I talk to men or parents of boys who have not done VMMC the importance of VMMC. We tell them like we were told at the time of our recruitment that VMMC is very important because it prevents cervical cancer for one’s partner and also that it reduces one’s risk of HIV infection by 40 percent, and not 100 percent. It does not encourage people to have unprotected sex once they get VMMC.*
9. I: Can you give me an example of a day when you talked to a person about VMMC and how he responded?
10. *R: I can give an example of the guardians of the children which we talk to. If we tell them the importance of VMMC that it protects from cancer for their partners to be and they understand such issues, they tell us to take the children to the VMMC clinic. So we take them and once they get circumcised then we bring them back to their homes.* ***01:42***
11. I: Mmm okay, can you give me another example of an older person whom you were able to talk to about VMMC?
12. *R: I can remember four or six older people within the ages of 20 and 25 whom I was able to convince to get VMMC within the two weeks that I worked with the VMMC staff.*
13. I: Mmm okay. Now what specifically do you tell older people for them to be convinced to get VMMC?
14. *R: Okay. Firstly, if it’s a youth it becomes easy because I am also a youth. I tell him that I was also afraid to get VMMC because people say VMMC does not serve for any purpose but I have done it last year. So I tell the youth that before you get VMMC you have to clean your foreskin every time because it holds some dirt. Even if you haven’t bathed for three to four hours such dirt accumulate in the foreskin so much that if you want to have sex you can contract HIV or cause some infections. So I tell them that for me such is not an issue and my penis is always clean because of VMMC.*
15. I: Mh.
16. *R: So they get convinced when they hear that I got VMMC last year. Some people believed that VMMC is connected to Satanism because they say when they cut the foreskin they use it for Satanism. So I told them that it’s not true because once a person gets VMMC the foreskin is gotten rid of while we see and it is not used for any satanic purposes.*
17. I: Mh.
18. *R: Sure.*
19. I: Lets now talk about couple testing and counseling. Tell me what happens when a man brings his partner to the VMMC clinic?
20. *R: We encourage partners when we talk about VMMC with them that they should come together when the man is coming for VMMC. But at the VMMC clinic we only conduct HIV testing on the one that needs VMMC and not both partners because VMMC is not a centre for HIV testing or other diseases. We just want to know if there will not be any complications if the man gets VMMC and that’s why we test him for some infections.*
21. I: Have you ever seen a man bring his partner here at the VMMC clinic?
22. *R: For the two weeks that I have worked with VMMC I haven’t seen any man bring his partner*
23. I: They did not bring their partners?
24. *R: No, and that is one of the challenges that we face that women don’t want to come with their partners.*
25. I: So it’s women who don’t want to escort their partners?
26. *R: Mh…*
27. I: Haven’t there been any cases where it’s the man who doesn’t want to bring his wife?
28. *R: Yes, some women have complained that their partners don’t want to come for VMMC for fear that people will laugh at them since they are already too old. SO we meet such cases where the woman is willing but the partner is not.*
29. I: For those that bring their partners here what do you think motivates them to do so?
30. *R: I think it’s because of other benefits that we tell them. We tell them that once a man gets VMMC, they as a couple will have longer sex and that excites women who in turn encourage their partners to come for VMMC. We also tell them that a woman whose partner is not circumcised is at risk of developing cervical cancer and that also encourages women to motivate and escort their partners to the VMMC.*
31. I: Alright. What is your opinion on integrating couple HIV counseling with Voluntary Medical Male circumcision services?
32. *R: I think it’s a good idea because if you test one partner while the other partner is outside waiting, they may falsify the HIV results to the partner since she was not there while the man was being tested. But if they said “we will conduct couple testing and counseling on those that have come with their partners” and they test both, it can help them to know how best to protect themselves if they are negative or how best to live their lives if they are positive. Even if the man will not get VMMC but they will start treatment early.*
33. I: Mh, what do you think should be done for men to bring their partners at the VMMC clinic?
34. *R: What can be done is that there should also be outreach clinics where they can be tested as couples and both will know each other’s status. While if you test one only, the woman will feel less important and will be unlikely to escort the partner. So it would be good if at the centres they test both partners if they come together.*
35. I: So what is needed are the outreach centres?
36. *R: Yes, so they can test both partners.*
37. I: Mh. What do you think are the barriers and concerns on this integration?
38. *R: If people know that both partners will get an HIV test when they come together at the VMMC clinic, then the numbers for those that get VMMC while married will be reduced because some men already know that they may be infected and wouldn’t want their partners to know and may prefer to not even come to the VMMC clinic. Where as if they were to go alone they would feel confident that if they are found HIV positive their partner will not know and they are negative they will still get VMMC.*
39. I: How can we address such a concern?
40. *R: People need to be sensitized and be told that HIV is real even if we can deny it. What is needed is for a person to accept if they have it than saying you don’t have it when you do. It’s also important for one to know his status than die not knowing. When you get tested early and you start treatment, you can live longer while if you don’t you can die early. If you know you have HIV you can know how best to live a normal life and live longer. Some people die during accidents when they are HIV negative while someone who is HIV positive can live a long life because he takes good care of himself. So telling them all of this can get rid of the concerns.*
41. I: Mh....
42. *R: Sure.*
43. I: I would like us to talk about sexual reproductive health services and Pills for HIV prevention: called pre-exposure prophylaxis. (PrEP) Sexual reproductive health include services that promote good sexual health and reproduction. They include but not limited to family planning, cervical cancer screening sexual transmitted infection management cervical, condom distribution and many more.
44. *R: Okay.*
45. I: Today we will only discuss family planning, Sexual transmitted infection management cervical screening and PrEP. We will look at each of these one by one. Let us start with STI services.
46. *R: Mm…*
47. I: Tell me what happens when a person is suspected or diagnosed with STIs?
48. *R: If a person is suspected or diagnosed with syphilis or gonorrhea, firstly he is given treatment so the infection can go away then he can come back for VMMC.*
49. I: What happens when he is found to have HIV?
50. *R: When he has HIV he is counseled on how to take care of himself by taking treatment but he cannot do VMMC.*
51. I: Why is it that he cannot do VMMC?
52. *R: For someone who has HIV, his immunity is compromised so if he is to get VMMC it’s like you are adding another infection. So we want to protect him from that.*
53. I: As a peer, what is your opinion on integrating STI services with Voluntary Medical Male Circumcision services?
54. *R: I think that’s a welcome idea because some people live very far away from hospitals while VMMC has outreach clinics in the communities. So if they integrate VMMC with STI management then it can reduce the number of STI infections and people will take care of themselves because they will know at an early stage and get treatment for the STIs.*
55. I: Mh. What is it that you don’t like about this integration?
56. *R: What I don’t like is that this is VMMC, so I think it would be best if we could concentrate on this service first so that those men and boys that are not yet circumcised could do so rather than telling them about these additional services that will also be at the centres.*
57. I: Mh.
58. *R: Say you talk to the guardians or parents about all these services and you find that the boy doesn’t get VMMC, the boys will be afraid that their parents will automatically know they have STIs. While if we don’t involve this integration and they don’t get circumcised the boys can have a better excuse to give to the parents. [laughing].*
59. I: So how do you think is the best way to offer STI services at the Voluntary Medical Male Circumcision clinics? At what time point and where should the service be offered?
60. *R: I feel like after sensitization has been done and the people have come to the clinic, the first thing to think about is the person’s privacy. This is so because most people feel shy when it comes to these issues. I can give an example of the VMMC centres that I have been to: they just use a cloth as a blind between one room and the other and sometimes the cloths are not big enough to cover the spaces. So some people are very shy to the extent that they fail to get VMMC. So with the STI services it cannot be right for these services to be in one room, not even with the blinds because some people may be hearing what they are saying in the STI room. Imagine other people hearing “we have diagnosed you with gonorrhea…” So in short what’s needed is people’s privacy so that no one else can be able to know their illness.*
61. I: Oh okay. So what do you think can be done so that this privacy is met at the centres?
62. *R: So I feel like there should be more than one room where they can be offering this STI service so that there should be one patient at one time in one room and what happens there stays private, like the way we are talking here.*
63. I: Mh. So at what time point should this service be offered in the VMMC clinic flow?
64. *R: I dint understand that question. You mean we should be skipping one step?*
65. I: No, I mean within the VMMC clinic flow, when should STI services be offered?
66. *R: I feel like there should be a partition so that anyone coming for STI services should follow a different flow from the ones that come for VMMC. Every flow should have their own reception and all the other things that are needed. Like those that come for STI services they should have their own reception and rooms for the service while those that come for VMMC they should have their own reception, counseling room, HTC room and the surgery. I feel it will be better if there is such a partition at the VMMC clinic.*
67. I: What do you think is the benefit of partitioning?
68. *R: This will reduce workload for the providers at the clinic because they will know that this group of people is here just for STI services while the other group is for VMMC. This is because some of the people that will be visiting the clinic are those that already did VMMC, so if they queue with those going for VMMC it would delay the process.*
69. I: Okay, thank you. You mentioned that what you do not like about this integration is that these are two different services and it will be good to concentrate on VMMC What other concerns and barriers do you think are there for this integration??
70. *R: The other concern that may arise is that when men learn that there will be such service at the VMMC clinic, they may think twice before visiting the clinic for fear that they may be found with infections. They may start to reason “I have been without VMMC all my life; why doesn’t my wife have cervical cancer? Why don’t I have HIV?” Some people may just want to make it look bad by saying “Why did God create us with the foreskin? If he knew the foreskin was bad he could have created us already circumcised”. SO some people may give such excuses so they don’t do VMMC.*
71. I: Mh, so how do you think we can address this concern?
72. *R: Ah…I think we can address this by… when people are going for sensitizations in the communities, they need to be very serious when talking to men about such issues. I feel it can be good when employing people, to go out for sensitization to look at the ages of the audience. It cannot be good for a young person aged 20 and below to sensitize older men about VMMC and tell them that there will be removing of the foreskin. You know older people need respect and so the ages of those employed to reach such people should be considered. Some young people can be employed to sensitize the youth but among the group there should also be some older people to reach the older men.*
73. I: Okay. Now I want us to talk about family planning. Tell me what happens when a person comes to the VMMC clinic to access family planning?
74. *R: If a person comes to the clinic seeking family planning we give him the information since these are related. We tell him the benefits of family planning based on how expensive life is nowadays. We tell him that nowadays for our children to be well educated we need to send them to expensive schools so you cannot have too many children when you cannot afford to pay for their education. Child spacing needs to be considered as well because of the same reason. So we give them all that information and the disadvantages of not using family planning, then we tell them the different types of family planning methods that are available and offer them condoms. If they want the other methods, we refer them to other hospitals to access them.*
75. I: Mm... So that means if a person wants other methods of family planning other than condoms you refer him to other clinics?
76. *R: Yes*
77. I: As a peer what is your opinion on integrating Family planning in Voluntary Medical Male circumcision services/clinic?
78. *R: I think that is necessary because it can be an advantage for us to be able to talk more about family planning since it is part of medical care same as VMMC. Some people don’t get sick that often so they don’t have the chance to know issues like that of family planning. Some get sick and buy drugs from pharmacies instead of coming to the hospital. So there is a need for the providers to have all the relevant information to give to people once they get a chance to meet them at the clinic.*
79. I: What is it that you would not like about integrating Family Planning services in VMMC?
80. *R: The problem can be the same as I earlier on explained that some couples don’t get along while others get along so for those that don’t get along don’t escort each other to the clinic and when one goes to the clinic and gets such information he may not see the information as important. Some men may not feel comfortable to be told about family planning.*
81. I: Why is that?
82. *R: Most men feel like they need a lot of respect and that they already know things. So they cannot be receptive to new information. Like the issue of family planning they my say “I already know about family planning. If I bear a lot of children I will take care of them”, when if you can give enough information they may be able to change.*
83. I: Okay. What do you think is the best way to provide the family planning services at the VMMC clinic?
84. *R: I feel the best family planning method is implanon.*
85. I: Mh…
86. *R: That is because condoms are not 100% reliable, they can break at any time and expose the woman to risk of pregnancy.*
87. I: You mentioned for STI service that there should be partitions between those that are coming for VMMC and those that are coming for STI services only. Now how best should it be done with the family planning?
88. *R: I think the information should be given earlier in the process then the service should be offered along with the VMMC only that it should be offered after one is done with VMMC. They should be shown a room where they can access the methods within the building.*
89. I: Mh. So there should be a separate room and they should be offered after VMMC.
90. *R: Yes*
91. I: How about those that are only coming for family planning and not VMMC?
92. *R: They should be allowed to access the service. They can go to that room and explain what they are looking for.*
93. I: Okay. What do you think could be the barriers or concerns on family planning and Voluntary Medical Male circumcision integration?
94. *R: Culture and religion can be some of the barriers to this integration because there are some cultures and religions which use one scripture from the Bible to say “We were told to fill the earth just like grains of sand” So telling them to use family planning is like telling them to commit a sin. They feel like if God doesn’t want us to have a child then he can block my wife so she doesn’t conceive. But if He has allowed my wife to conceive then let it be. So that can be a barrier from culture and religions.*
95. I: How can we deal with such concerns or barriers?
96. *R: It’s by telling them the benefits of family planning because nowadays for one to have too many children is not healthy. We see people who have some money and have two children only but are not able to afford the children’s education. So giving them such examples and telling them the dangers of child bearing to the health of the woman can help. Even if it’s a car and every time you overload it, then it cannot last long. The same applies to women who give birth every now and again: they may have problems with the uterus or their general health. It can happen that one of the pregnancies can bring complications or even maternal death. Child bearing is not something one gets used to.*
97. I: Okay. Now let’s talk about cervical cancer screening. Tell me what happens when someone wants to get cervical cancer screening?
98. *R: Honestly I have never come across such cases in the two weeks I have worked here.*
99. I: Okay. But you know that women get cervical cancer screening right?
100. *R: Yes, that I know.*
101. I: As a peer, what is your opinion on integrating partner cervical cancer screening with Voluntary Medical Male circumcision services
102. *R: I feel like it’s very important because when a man wants to get VMMC it’s because he wants to prevent his partner from cervical cancer. So VMMC on its own does not prevent cervical cancer if it was already there, it’s only from the time the man gets VMMC forward. So if the woman gets screened before the man gets VMMC it can help even if she is found out to have the cancer because she can be put on treatment. If she doesn’t have it then VMMC will be able to prevent it. This is better than a man just doing VMMC without knowing whether the partner already has it or not. There have been several cases where women have cervical cancer and yet their partners did VMMC. So I feel this is very important.*
103. I: What is it that you would not like about this integration?
104. *R: That this integration should not be done?*
105. I: Yes.
106. *R: The issue is still going back to couples. If you talk about this issue to parents that their girls should also get screened before they get married it can be easier for them to send their children for screening. While married people procrastinate. They feel that they will be screened at the time when they give birth. They can feel shy to come to the VMMC clinic where their children go for VMMC. So the thought of being seen there makes them feel shy.*
107. I: Mm, they feel shy.
108. *R: Yes, they may feel like people will automatically know that they are going there for cervical cancer screening.*
109. I: Mh, how can we deal with such a barrier?
110. *R: I feel the best way to deal with this is by telling the chiefs to gather their people in the communities so we can sensitize them because right now all we do is go door to door and talk to people about VMMC not the whole community at once. We need to give this information to the chiefs first so they can understand the situation and be able to gather their people. Knowledge is power: of course some people may say “it’s my life, I can do whatever I want with it!” but we tell them “Yes this is your life but if you don’t get screened now and you are diagnosed with this cancer at a later stage, it becomes difficult for one to get healed. You can only get treatment outside this country but it’s very expensive and you can die while waiting to go there or soon after you reach there.” So some people can be motivated to go and get screened regardless of what people can say once they know.*
111. I: Okay. So when is the best time to offer this service?
112. *R: The right time is when they…because this is a serious illness. You find that a woman goes for antenatal clinic or to give birth and she is diagnosed with it. So I feel this should be urgent. When they check blood sugar and BP they should tell men who have come with their partners to go to the screening room.*
113. I: Alright. So who do you think should be screened for cervical cancer at the VMMC clinic?
114. *R: It should be everyone that comes for the service because this disease can attack any woman. Even if she comes for other services like HIV testing, she should also be screened for cervical cancer.*
115. I: Why do you say so?
116. *R: It’s because this is a serious illness which if not treated with urgency will cost a person’s life, unlike HIV which you can delay for some days. That’s why I am saying whenever a woman comes she should be screened.*
117. I: Okay. Now let us talk about PrEP. Have you ever heard about PrEP?
118. *R: No I have never heard about PrEP.*
119. I: Oh okay. So PrEP is an anti-HIV drug which helps to keep HIV-negative people negative. There is a pill that one needs to take every day to prevent HIV infection.
120. *R: Oh okay.*
121. I: So how do you feel about PrEP?
122. *R: Okay…so like anyone who wants unprotected sex can just take the pills?*
123. I: Yes.
124. *R: So I feel like… can you repeat the question?*
125. I: How do you feel about PrEP?
126. *R: Okay I don’t really like it because if people are told that if you take these pills they won’t get infected even if they have unprotected sex, it encourages sex. So I feel it’s not a good drug.*
127. I: [laughing] Now do you think it’s necessary for PrEP to be made available to HIV-negative men and women?
128. *R: Yes, I feel it’s necessary, even though I said I don’t like it. I say it’s necessary because this generation is a weird one where people love sex. So it’s necessary for such people to be protected and be given these pills.*
129. I: Would you encourage someone to be taking the pills?
130. *R: Yes, I can encourage them.*
131. I: What reasons would you have to encourage someone to take them pills?
132. *R: It’s because there are some people even my friends who say they cannot manage to stay a week without sex, some who love unprotected sex and others who have un consented sex or have sex because they are drunk which makes them not to think about protection. I can encourage such people because it can protect them from infection.*
133. I: Mh... If PrEP become available, what is your opinion on integrating PrEP with Voluntary Medical Male circumcision services?
134. *R: Would you repeat the question?*
135. I: Mm… If PrEP become available, what is your opinion on integrating PrEP with Voluntary Medical Male circumcision services?
136. *R: I think I can be very happy if they do such an integration because sometimes VMMC protects people from HIV infection, so adding that percentage of protection with PrEP means it can increase the protection to 100%.*
137. I: Mh. How do you think PrEP would be offered in this clinic?
138. *R: It can be offered to those that have done VMMC. They can be asked after the procedure if they love unprotected sex with women and be given the pills to be taking daily*
139. I: Mh.
140. *R: Yes, so after the surgery they can ask if you have any interest in taking PrEP. That is the best time. If they don’t love unprotected sex, then they cannot be taking the pills*
141. I: What would be your concern if people are taking PrEP?
142. *R: My concern would be the same That I mentioned that people will be having unprotected sex and since most men that come for VMMC are aged 25 and below who have not yet been married, then family planning may be difficult because people will want to have unprotected sex. This will render our family planning message useless because they will be saying they have taken PrEP and won’t need to protect themselves*
143. I: How do you think we can address such concern?
144. *R: I think people need to be warned that the pills prevent HIV infection not pregnancies. So we can tell them that they can be taking PrEP and still use condoms if they are not married because PrEP reduces the risk of HIV, condoms can break and expose on to HIV but if you are using both then even if the condom breaks then you cannot be infected. This in a way promotes family planning using condoms and HIV prevention at the same time.*
145. I: Mh…
146. *R: Sure*
147. I: If you were given powers to choose and integrate services in Voluntary Medical Male Clinics, what are the services that you would think of Integrating?
148. *R: I can opt for cervical cancer screening because the situation is so pathetic for women. Cervical cancer screening and VMMC are connected because VMMC reduces the risk of cervical cancer in women. So if there is funding I can support this screening because they are connected.*
149. I: Mh…
150. *R: I also like PrEP to be integrated because it is also preventing HIV infection just like VMMC which has a certain percentage that protects against HIV. So these two can support each other if integrated*
151. I: Thank you very much for your time. Your answers will be very helpful in improving the health service delivery at circumcision clinics.
152. *R: Mm… thank you.*
153. I: Before we close, is there anything more you would like to say?
154. *R: I feel we have already talked about a lot of things.*
155. I: Okay. Thank you very much for talking to me today.
156. *R: Thank you.*

END

**D 43 STUDY**

**Date of Interview: 11 August 2018**

**Type of Participant: Peer or Clinic Aid**

**Interview Number: D-43-0021**

**Interviewer: I.N.**

**Total Interview Time: 41 minutes 02 seconds**

**Interview Summary:** **(from summary sheet)**

| **SERVICE TO BE INTERGRATED** | **THOUGHTS ON INTERGRATION** |
| --- | --- |
| Couple HIV Testing and Counseling | Thinks this integration is very necessary. It will help relationships work well. |
| STI Services | Thinks it’s a good idea though it is already integrated. |
| Family Planning | Things it’s a good idea. It will to reduce congestion in other clinics and reduce population growth |
| Cervical Cancer Screening | Thinks it’s a good idea but would be better if it stands on its own as separate. |
| PrEP | Thinks it is a good idea. Will reduce the rate of people infected with HIV. |
| Other Services | PrEP, STI services, couple HIV testing and counselling. |

**Remarks:**

**Participant was relaxed, open and happy. He like the idea of integration though he does not like other services to be included in circumcision like family planning and cervical cancer screening.**

**Interview Texts**:

1. I: [Clearing throat] Thank you for taking the time to talk with me today. I would like to ask you some questions today about the way you feel and what you think about some issues related to the service you provide and how we can include other services in Voluntary Medical Male Circumcision (VMMC) clinics.
2. *R: Okay.*
3. I: There are no right or wrong answers to these questions.
4. *R: Okay.*
5. I: We would like to hear your opinion and your experiences in your own words. Do you have any questions before we begin?
6. *R: No. I will be just waiting for your questions so that I can answer*
7. I: Ah, can you tell me how you are involved in the client care at this clinic?
8. *R: The service which we are involved in implicates going to the villages and mobilizing the children for circumcision*
9. I: Does your peers talk to you about how the services are provided here?
10. *R: Yes, they could talk.*
11. I: Can you give me an example of a time that your Peer talked to you about the services he received here?
12. *R: Yes, like a friend who I came with, he explained that the doctors welcomed him well and also by the time, before circumcision and after circumcision, he experienced something different.*
13. I: The things which he experienced different, what do you mean?
14. *R: [laughing] yea, the time which he wasn’t circumcised, issues like of sexual intercourse, firstly, foreskin was disturbing him and secondly, he was feeling the pleasure of sexual intercourse than at first.*
15. I: Okay. Now let us talk about partner HIV testing here at the Voluntary Medical Male circumcision clinic. Tell me what happens if a man brings a spouse here at the Voluntary Medical Male circumcision clinic?
16. *R: Okay, when a man brings his spouse, we do get both of them and start giving them advice, before counselling. We start counselling before receiving treatment right, then after counselling them, that’s when they do receive treatment. The aim of counselling is that, maybe when we are counselling them, we should change their mindset. There are other people, upon seeing their partners, after testing, if the partner is found infected with diseases, they could leave their partners right there on a chair like putting a blame on a doctor, and leave. So, we want such kind of scenario to get reduced.*
17. I: Have you ever seen a man coming with his spouse?
18. *R: Yes, like a lot [laughing]*
19. I: More do come?
20. *R: Yes*
21. I: But there are men who does come alone. Have you ever seen a men who come alone?
22. *R: Yes, people come alone*
23. I: What make such kind of men not to come with their spouses to come and test for HIV?
24. *R: firstly, it might be that they don’t have confidence in themselves that they might be found with a virus. So once you are found with a virus while your spouse is on your side… mostly, you think that, I will be found with a disease and not my partner, so things will not end well. It also happens with way you do behave sexually like the way I do (sleeping around) and I should go with my partner? No, it’s better if I go alone first. If I see it that am okay, that is when I can gather courage to come with my partner.*
25. I: Mm. Aaah, what do you think are the motivators that make the men bring their spouses here for testing?
26. *R: Firstly, it is love but also they want to know the status of a woman, also how their status is, so that when they are doing things, they should be doing it freely. ehm*
27. I: What do you think can be done to make men bring their partners here for couple testing and counselling?
28. *R: The main thing is to send information through phones, even radio, explaining things which can motivate them to come with their spouses for testing at the place like here. It’s not a sin [laughing] don’t give up easily.*
29. I: [Laughing] okay. You as a peer, what is your opinion on integrating couple counseling with Voluntary Medical Male circumcision services?
30. *R: This service, I do see it as very necessary, not because am a peer but to health side. This service is really necessary because once you are both found positive, you may clearly understand each other and do things open.*
31. I: Okay. Aaah, what do you think are the barriers and concerns on this integration?
32. *R: Ah, there is no barriers here*
33. I: Or which you see blocking this integration
34. *R: Ah, am not seeing the barrier, unless when there is misunderstand but also maybe when there are inadequate materials. Those things can be barriers*
35. I: What do you think can be done to overcome these concerns and barriers to couple counseling in Voluntary Medical Male circumcision services clinic?
36. *R: Working together on what is needed at that place but also understanding each other, discussing things that it should be like this but also doing things together as one.*
37. I: Okay. Now I would like to discuss with you about sexual reproductive health services and Pills HIV prevention: called pre-exposure prophylaxis. (PrEP). Sexual reproductive health include services that promote good sexual health and reproduction. They include but not limited to family planning, sexual transmitted infection management, cervical cancer screening, Condom distribution and many more. Today we will only discuss about family planning, diagnosis and management of STIs, Cervical cancer screening, and PrEP. We will look at each of these one by one. Let us start with STI services. Explain to me what happens if a client is suspected or diagnosed with an STI here?
38. *R: Okay, a person who has come for circumcision and has been screened and found with a virus, that person is not supposed to be circumcised. He is supposed to be send back home*
39. I: Mm, so don’t they give him any service?
40. *R: The service is supposed to be given. The service get provided…*
41. I: Then after receiving the service and sent back home, does they come back or they don’t allow him to come back?
42. *R: They are supposed to come back because the service which they are given is supposed to drug treatment were they are supposed to take drugs of HIV and AIDS which add immunity in the body. So they are supposed to be coming and receive drugs and also getting condoms so that when they got home, it happens that a person you might say let me go somewhere and test something different [other women], they should be using things like that.*
43. I: Okay. Aaah, you as a peer what is your opinion on integrating STI services with circumcision services?
44. *R: On screening?*
45. I: Yes, or we should say how do you think or we should say what is your mind, that at circumcision clinic, they should be doing screening and give STI services?
46. *R: The service, you mean what?*
47. I: I mean that, if a person, you have screened and you have been found with STIs, so they should give you treatment so that it should be like you are being treated at circumcision clinic. How do you see it, if we integrate that?
48. *R: The treatment like he should be allowed to be circumcised?*
49. I: No… that you should receive drug treatment, they should help you like in terms of counselling, like a hospital where they will be offering such kind of services.
50. *R: Yes, that is needed and it is also a very good idea because in other clinics since they are like separate, so it founds that there are a lot of people. So to be standing on line [queue], others are found to have other things to do [work. If we can integrate this service to circumcision, such kind of scenarios, people will be reduced because other people will be receive the services here. That ideas, is very good.*
51. I: What is it that you would not like the integration of diagnosis and management STIs with Voluntary Medical Male Circumcision services?
52. *R: There is no point for disagreeing there. As for me, I just appreciate that the ideas which they have brought are good. But there is no point for disagreeing as their points sounds good. It is good that people should be receiving services at the place where they have been found with virus as they come for circumcision. Those who have been found, they should be willing to be helped by given treatment. It is a good idea*
53. I: How do you think STI services should be offered at the Voluntary Medical Male circumcision clinic?
54. *R: Screening?*
55. I: Yes. How do you think are the good ways for screening and offering STI services when a person is found at circumcision clinic?
56. *R: I don’t understand the question*
57. I: If we integrate STI services, with the circumcision process, where should this service be offered? When should it be offered?
58. *R: Okay, by the time when a person is found with infection, you need to give him medicine, which he can start with and tell him dates. There is a need to build a room where those found with STI, they should be receiving the service besides circumcision. They need to be coming and receive services while others are also being helped in the other room [those for circumcision].*
59. I: Okay, within the same clinic?
60. *R: Yes.*
61. I: And not outside?
62. *R: Not outside*
63. I: Okay…
64. *R: With outside room, you can be spotted by people passing and you might be shy*
65. I: [Laughing] that’s true. What do you think are the barriers and concerns on this integration?
66. *R: There is no barriers*
67. I: Or any one concerns which may come out
68. *R: [laughing] the concern maybe that other people might be found with a virus, they could be like,, I came for circumcision and yet they are telling me to go there to receive treatment, aaah, I cannot go. Others, you can tell them, and they can agree that we will be coming to get medicine but upon going, they run away without being found again.*
69. I: What do you think should be done to address these concerns and barriers? Like what you said that people might run away, if they have been found with STIs.
70. *R: When a person is found with a virus, you need to give him hope [strengthening words] like it happens, you need to be strong, this is not the end of life. You can give him words which can give him hope but not when you meet him at the road, starting to laugh at him, pointing fingers with your friends at him no, you need to talk at him like a relative, a person who you know him well. Not starting laughing and pointing fingers at him, so those kind of people, they do have worries, like they do point fingers at me and fade my image that makes things like this not to work.*
71. I: Okay. Now let us talk about family planning as one of the services which we would like to integrate with circumcision services. Explain to me what happens if a client needs a family planning methods whether a man or a woman?
72. *R: First thing, they need to talk about it with the spouse that, I need family planning method, so it depends on whether the man will agree with it. If he agrees, they need to come and receive the treatment.*
73. I: Mm, what methods of family planning do you know? Whether for men or women. Do you know that men also do family planning?
74. *R: [Laughing] no*
75. I: Men also do family planning
76. *R: Oh [laughing] yea I know*
77. I: You know right?
78. *R: Yes…*
79. I: You know what and what?
80. *R: Okay, using condom, is like the other way of family planning. Since the sperms do not reach a women and it cannot do any process. That is the side of men*
81. I: Okay, how about women side?
82. *R: On women side, they can go for injection at the hospital but also using female condoms of care, is the other way of family planning.*
83. I: How about other methods of family planning which you know? If there are any other besides that of condoms
84. *R: I don’t any other*
85. I: That of injections don’t you know it
86. *R: Of injection I have already explained it*
87. I: Oh you have already explained
88. *R: Yes. Injection, they could do that*
89. I: Other methods are depo-, loop, they could block
90. *R: Yes, block way*
91. I: Ah, as a peer, what is your opinion on integrating Family planning in circumcision services?
92. *R: Family planning, really need to be integrated with circumcision because people fail to access like how could we do family planning. But at a time when people could found family planning through circumcision way, many people could know that when they go for circumcision, there is also things about family planning there. Which means many people will be coming, it will be that many people who were lacking access to this kind of service, they found it.*
93. I: Mm, would you like it?
94. *R: Yes, I would really like it. In this world the population is increasing and the jobs are rarely found [laughing]*
95. I: [Laughing]
96. *R: We need to be decreasing so that the jobs can be found*
97. I: Yes, we are really increasing. What is it that you would not like about the integration of family planning with Voluntary Medical Male Circumcision services?
98. *R: Okay… like on the side of women, firstly, circumcision involves only men, women don’t circumcise. So once the meeting site for people who come for family planning and those for circumcision, I see that it can bring a certain problem; Men might be having shy like there will be women and they will be knowing obviously that we are going for circumcision [laughing], this can discourage people*
99. I: Okay. So how do you think family planning services would be offered within Voluntary Medical Male circumcision clinics?
100. *R: Family planning services need to be located at its own place*
101. I: And not within the same clinic?
102. *R: Yes. Or it might be outside the clinic, just putting a tent because it seems that, family planning is not too involving than circumcision.*
103. I: Mm. Alright. What do you think are the barriers and concerns with the integration family planning and Voluntary Medical Male circumcision services?
104. *R: Family planning with circumcision?*
105. I: Yes…
106. *R: There is no barrier.*
107. I: Mm… let us talk about cervical cancer screening for female partners. Explain to me what happens if a woman needs cervical cancer screening?
108. *R: If she doubts herself of having cancer, she need to explain it to her partner like those one like am not feeling well in my body at this part of the body, I need to go to the hospital, so that they can screen me well. Or not even explaining to your man, because your life, need you to take care of it yourself. It might be found that the man is refusing her to go to the hospital, while she is not feeling well. You need to go to the hospital alone and meet a nurse. The nurse is supposed to screen you and if you are found with cancer, you need to be treated… yea*
109. I: So, what is cervical cancer or how does it get detected? So that a woman knows that she has a cancer or what causes it?
110. *R: On that part, I don’t know clearly*
111. I: You are not following?
112. *R: Yes…*
113. I: So, as a peer, what is your opinion on integrating cervical cancer screening for female partners with Voluntary Medical Male Circumcision services?
114. *R: Concerning cervical cancer and concerning integration, can you ask again?*
115. I: Yes. Aaah, cervical cancer screening integrating together with circumcision services, how do you see it? What would you like on this integration?
116. *R: Okay, it is really very exciting since women go to the hospitals to screen for such kind of diseases, so it’s like you are reducing the workload from such kind of hospitals since it gets congested with people. So as you have to start this, it will be like other people will be coming here, thereby reducing the problem of congestion. This could be very helping.*
117. I: Okay. Ah, what is it that you would not like about the integration of partner cervical cancer screening with Voluntary Medical Male Circumcision services?
118. *R: nothing, that deserve to be integrated. It is necessary that while the other has circumcised and other get screened, knowing the status in his body. While you are receiving treatment after circumcision, a person might be thinking I have to do certain things to my wife but without knowing that she is on a treatment. But after integrating, they might be knowing that I came with my wife, she have been found with cervical cancer, she is on a treatment right now, and we have to stop doing this kind of things. It helps*
119. I: Okay. How do you think is the best way to offer cancer screening within Voluntary Medical Male circumcision clinics?
120. *R: For those people who came together, it’s not good that… [Laughing] aaah but on that part, they also need to find a private room with a clinic and not outside, since it involves operation, it needs a good and protected place.*
121. I: Okay. When should it be offered?
122. *R: It is needed to be found at a time when that person has been found with that problem. The service need to be provided*
123. I: Okay like in circumcision process, when the man is going for circumcision, as he is being screened for STI or where? How is it supposed to be?
124. *R: This is supposed to be at the time when a man is going for circumcision and integrating that, can work well*
125. I: When screening woman, does a man need to be right there? Or as a women is doing their own thing, a man should also be doing their own thing?
126. *R: [Laughing] there should be that as a man is doing his own thing, a woman should also do her own thing… to be done together, one can be curious to be seeing the partner, it can be disturbing doctors to do their work well*
127. I: Okay. What do you think are the barriers and concerns about integrating partner cervical cancer screening and Voluntary Medical Male Circumcision services?
128. *R: Ah no*
129. I: Nothing?
130. *R: yes. The concern may be to the women.*
131. I: To women, what is the concern? The way you think
132. *R: Because the cancer can find her when she doesn’t even have a child or have one child while she needed two, it is not a good thing, that is a barrier*
133. I: So, what do you think should be done to address these concerns and barriers?
134. *R: That concern can be addressed by giving her treatment. Explaining to her like be strong, we know your plans were not like this but since it has happened, we will not let you get sick when you need something, maybe something you want, cannot be found because you are like this, giving something of hope that’s all, and giving right treatment like medicine…yea*
135. I: Alright. Aaah, let us now discuss about PrEP. Have you ever heard about PrEP?
136. *R: No. That I will heard from here*
137. I: Or PEP
138. *R: No.*
139. I: No?
140. *R: Yes…*
141. I: If you have not heard about PrEP, I will explain how the medicine works
142. *R: Mh…*
143. I: PrEP is anti-HIV medicine that keeps HIV-negative people from being infected. For instance, a man might be negative and his wife positive. He knows the status of his partner, as he takes PrEP, a women cannot give a virus to him
144. *R: Okay*
145. I: But for this to work, there is a single pill that is taken once daily, and if you take it regularly, it is highly effective at prevention people from being infected. If someone ask you what PrEP is, what can you say?
146. *R: Okay, PrEP is a medicine that help protect people so that they don’t share HIV*
147. I: PEP is a medicine which you take after having unprotected sexual intercourse with a person who you doesn’t know his status before 72 hours, three day right. But PrEP you take it regularly and you might be knowing that my partners status is positive and you can be having unprotected sexual intercourse without sharing a virus, if you are following the prescriptions.
148. *R: Okay*
149. I: If PrEP was made available to HIV- men and women. Do you think you could advise your HIV negative peers to accept to take PrEP?
150. *R: That is really necessary because it will help reduce the rate of people who might be getting diseases [HIV]. While he is taking the medicine, he will be protecting himself, so he can’t find a chance to get… yea, that is necessary*
151. I: If PrEP becomes available, what is your opinion on integrating PrEP with Voluntary Medical Male circumcision services?
152. *R: If PrEP become available, it is not supposed to be given only to people who are in marriages even youths need to be given since the youth could be using it since they also don’t want to get the disease, everyone need to take care of his life. So to my youths, need to be given such kind of medicine*
153. I: Would you encourage clients to take PrEP?
154. *R: Yes, I would like that.*
155. I: Okay
156. *R: Even the first person to use it, it can be me [laughing]*
157. I: [Laughing] that could be something really good. Okay, alright. How do you think PrEP would be offered here at Voluntary Medical Male Circumcision clinic?
158. *R: For the person who has come for circumcision, after circumcision, need to explain to him about these medicine like you came, we have circumcised you but there is also this medicine of PrEP which protect a person if you take it in a right way, you might be sleeping with a person who does have a virus but without you getting it. The giving counselling, he is interested, he can be given the medicine for free.*
159. I: Okay. What do you think could be the concern and barriers to integrating PrEP Voluntary Medical Male Circumcision services?
160. *R: On this part, there is no barriers because this medicine seems that it is really good and everyone need to take care of his life, its only few who have been circumcised and they may be refusing to get the medicine*
161. I: Those who maybe refusing, what do you think could make them refuse?
162. *R: Some pretend to be innocent on face as they will be like “what is that for, we don’t like it” but worrying somewhere else, I could have taken that thing*
163. I: How could you help those kind of people?
164. *R: Highlight them by explaining, we see with the way the person looks, telling them that you should not just say because maybe you are saying that in front of us but upon going somewhere you will be regretting, it is better if you get it now. Explain to him about how the medicine works, maybe it can make him interested to get the medicine*
165. I: Alright. Let us talk about other services. If you were given powers to choose and integrate services in Voluntary Medical Male Clinics, what are the services that you would think of to Integrate? Maybe even what we mentioned earlier like family planning, PrEP, cervical cancer screening, couple HIV testing, STI services. What can you choose to be integrated with circumcision services?
166. *R: Firstly, couple HIV testing. Secondly, PrEP. Thirdly of screening but that concerning cervix, I think it should be on its own*
167. I: Okay. Explain to me what the reasons are for your choices
168. *R: Okay, others seems if they integrated it can be easy but to the part of cervical cancer, treatment seems like a bit difficult. That’s why I put those on separate so that it does not get integrated with others*
169. I: So, for those services which you have chosen, would you give me the reasons for each, why it should be integrated with circumcision services
170. *R: Okay like of PrEP, most youth, came for circumcision firstly, they want to be protected as for circumcision, and it is not easy to get the disease. Yes you do get the disease but not that easily than not circumcising. It need to be together with PrEP but also couple HIV testing, since you know the status of a woman and a man, it can also give you confidence that, I should circumcise, my wife is well. If she is found with diseases, you say I should circumcise though she has diseases but I can still be doing what I want because on the side of circumcision, to get diseases is not easy, it is like you are being protected.*
171. I: Other? You chose three if not four
172. *R: I chose PrEP, couple HIV testing and counselling, what was the other*
173. I: Cervical cancer screening… that is what you rejected
174. *R: Yes, I rejected of cancer*
175. I: Family planning?
176. *R: Family planning need to be integrated*
177. I: Why family planning?
178. *R: Because… [Laughing] family planning is good. Circumcision should be integrated because by the time a person circumcise… [laughing] this is difficult*
179. I: It is difficult?
180. *R: Yes. It could have been on its own. It is difficult for me to integrate it*
181. I: So it is not supposed to be integrated?
182. *R: Yes.*
183. I: The other one is STI services. Have you chosen it or?
184. *R: STI service and circumcision?*
185. I: Yes.
186. *R: I have chosen it*
187. I: Why do you think it is good?
188. *R: It is good because we can say that, that thing is already integrated because a person before you get circumcised, you start being tested and then circumcision itself. That am seeing it being integrated already*
189. I: Okay
190. *R: Since it protects a person to be tested before circumcision. After circumcision, it helps as it tells nurses that this person is infected and is not supposed to be circumcised. But if it is not integrated, they might not be knowing as they could be just saying that he has come, let us circumcise him, found that they are worsening the situation for those with diseases*
191. I: So, how do you think these services would be offered in the clinic? Like PrEP
192. *R: like PrEP, couple HIV testing and counselling and circumcision, I think like they have to go through the same way because firstly, you will begin with testing the person. After testing you will know the status of that person. You will do your own procedures like you do. You will start with testing then circumcision, after circumcision that’s when you will give him some advice and telling him that there is this medicine. It is simple to integrate these*
193. I: Okay. Alright. How about screening STI? Oh that has been integrated already right?
194. *R: Yes.*
195. I: So, thank you for taking your time to discuss with me today.
196. *R: Thank you…*
197. I: Your answers will be very helpful in improving the health service delivery at Voluntary Medical Male circumcision clinics. Before we close, do you have anything to say?
198. *R: right now I will start with PrEP, since I have heard it from here. I will explain to my fellow youth and even eider people so that they could know that, this medicine is coming soon. To the circumcision, they have integrated things like this, even the other which has been explained, will be told so that people are aware about this*
199. I: So this, since it is a research, we want to hear people’s opinion first. We haven’t been confirmed to be like that yet
200. *R: So, it needs not to be told first*
201. I: yes. This is a research which we want to hear your mind, if many people will accept this integration as a good thing, that’s when we will integrate
202. *R: Okay.*
203. I: Is there any other question?
204. *R: There is no question. I should just thank that you welcome us well, we have learnt other things which we were not knowing*
205. I: Again thank you so much
206. *THE END*

**D 43 STUDY**

**Date of Interview: 11August 2018**

**Type of Participant: Male Index Participant**

**Interview Number: D-43-0022**

**Interviewer: I N.**

**Total Interview Time: 27 minutes 49 seconds**

**Interview Summary:** **(from summary sheet)**

| **SERVICE TO BE INTERGRATED** | **THOUGHTS ON INTERGRATION** |
| --- | --- |
| Couple HIV Testing and Counseling | Important service if both partners are to be protected. |
| STI Services | Can work together with VMMC because one will get VMMC and STI treatment on one visit. |
| Family Planning | The integration is possible for those that want family planning. |
| Cervical Cancer Screening | Whole purpose of VMMC is to reduce risk of cervical cancer so these services are connected. |
| PrEP | A good idea as this integration would provide full protection. |
| Other Services | Thinks PrEP is the best for integration. |

**Remarks:**

**Participant was relaxed and had a good sense of humor. He easily understood the questions and was very open-minded.**

**Interview Text:**

1. I: Okay, so thank you for taking the time to talk with me today.
2. *R: Alright.*
3. I: I would like to ask you some questions today about the way you feel and what you think about some issues related to the service you promote here and how we can include other services in Voluntary Medical Male Circumcision (VMMC) clinics.
4. *R: Alright.*
5. I: There is no right or wrong answers to these questions. We would like to hear your opinion and your experiences in your own words. Do you have any questions before we begin?
6. *R: No I don’t have any questions.*
7. I: Alright. What role do you take in Voluntary Male Medical Circumcision services?
8. *R: When our friends get VMMC they prevent HIV infection.*
9. I: Do you talk to your friends about VMMC?
10. *R: Yes I do.*
11. I: What do you tell them?
12. *R: I tell them that VMMC helps to reduce the risk of infections.*
13. I: When did you get VMMC?
14. *R: It was last year.*
15. I: So from last year to this day what has been your role at the VMMC clinic?
16. *R: I have been working there at the VMMC clinic.*
17. I: Okay so what type of work were you doing at the VMMC clinic?
18. *R: I go into villages and get children to VMMC clinic.*
19. I: So how were you getting them?
20. *R: By approaching their parents first and if they consent we would then take the children and tell them not to be afraid and explain the benefits of VMMC. Then we would take them to the VMMC clinic and stay with them there so they wouldn’t be afraid. Then we would take them to the HTC room where they would have HIV testing then check their blood pressure. SO once that is done we would take them to the surgery room where they would tell them to take off their clothes and be given anesthesia on the penis. They would then lie down and put their hands behind their head and the provider would do the surgery.*
21. I: Mm… okay, what VMMC benefits were you telling to your friends?
22. *R: That VMMC enhances hygiene: one doesn’t need to clean his penis every time and again. Also it reduces the risk of HIV infection when having sex.*
23. I: Mm… what are other benefits?
24. *R: If one has not done VMMC he is at a high risk of infections and also transmitting cervical cancer to his partner. So VMMC reduces that risk.*
25. I: Mm… okay. Now let’s talk about couple testing and counseling.
26. *R: Okay.*
27. I: Tell me what happens when a man brings his partner to the VMMC clinic?
28. *R: It happens but it’s only the man who gets HIV testing because he is the one who gets VMMC since a woman cannot get VMMC. So maybe it can be at another clinic where they can both get tested as a couple.*
29. I: Mm… okay but right now they only test the man?
30. *R: Yes, they only test the man and not the woman.*
31. I: For those men that bring their partners here what do you think motivates them to do so?
32. *R: They want to know each other’s status and want to protect their families from infections*
33. I: Mm…
34. *R: I believe so.*
35. I: What do you think makes men not to bring their partners here?
36. *R: I think its fear of being found HIV positive depending on how they have been leading their lives.*
37. I: Why do they have fear?
38. *R: They think that they have HIV while their partner doesn’t. So they are afraid that if they are both tested together and he is found HIV positive while his partner negative then he can lose his partner. But it’s important for both to go and get tested together and accept whatever outcome there maybe. If one is found positive and the other negative they also need to accept that.*
39. I: [Laughing] So what do you think can be done to make men bring their partners for couple counseling and testing?
40. *R: It’s up to organizations like you to do a research and see what can be the best way to get men to come with their partners.*
41. I: At the time when you came for VMMC did you bring your partner?
42. *R: No I didn’t because I am not married [laughing].*
43. I: How about a girlfriend?
44. *R: That one I have.*
45. I: So did you bring her at the time you got VMMC?
46. *R: No, I didn’t but we went and had couple testing and counseling at another hospital.*
47. I: Alright. What is your opinion on integrating couple HIV counseling with Voluntary Medical Male circumcision services?
48. *R: That is very important if both couples are to be protected.*
49. I: Mm… What do you think are the barriers and concerns on this integration?
50. *R: I don’t think there can be any barriers or concerns because this is all about everyone’s future.*
51. I: Don’t you think your friends can be concerned with this?
52. *R: No I don’t think they would be concerned.*
53. I: I would like us to talk about sexual reproductive health services and Pills for HIV prevention: called pre-exposure prophylaxis. (PrEP) Sexual reproductive health include services that promote good sexual health and reproduction. They include but not limited to family planning, cervical cancer screening sexual transmitted infection management cervical, condom distribution and many more.
54. *R: Okay.*
55. I: Today we will only discuss family planning, Sexual transmitted infection management cervical screening and PrEP. We will look at each of these one by one. Let us start with STI services.
56. *R: Mh…*
57. I: When I say STIs I don’t mean HIV but other STIs.
58. *R: Yes, like syphilis, gonorrhea and so on.*
59. I: Yes such STIs. Tell me what happens when a person is suspected or diagnosed with STIs?
60. *R: That becomes a problem if one wants to have VMMC. So he is given treatment and told to wait then alter they perform the surgery.*
61. I: Do they do the surgery on the same day?
62. *R: No it takes some days.*
63. I: Why is it that they don’t do the surgery on the same day?
64. *R: they fear that the infection can be enhanced because of the VMMC wound.*
65. I: At the VMMC clinic, do they offer STI screening for everyone that comes?
66. *R: No it’s just those that come for VMMC.*
67. I: As a peer, what is your opinion on integrating STI services with Voluntary Medical Male Circumcision services?
68. *R: For that I can only ask organizations to see how they can help so that people can also be coming to get HIV testing at the VMMC clinic.*
69. I: Okay so now I am asking what is your opinion on integrating STI services with Voluntary Medical Male Circumcision services?
70. *R: I think that can be good because these can work together.*
71. I: Can you elaborate on that?
72. *R:These can work together because you can firstly get VMMC then get HIV testing and that means you have prevented infections.*
73. I: Remember I mentioned that STIs here refer to the other infections apart from HIV. So what is your opinion on integrating STI services to the VMMC clinic?
74. *R: It will help people to get treatment early if they are diagnosed early as well and that will help us as young people to be protecting each other*
75. I: Mm... What is it that you don’t like about this integration?
76. *R: There is nothing I don’t like about this.*
77. I: Okay, you know how things work at the VMMC clinic right?
78. *R: Yes…*
79. I: So how do you think some men can feel about this?
80. *R; I feel some men can accept it while others can be embarrassed to come to the clinic so that can be a problem.*
81. I: Why would they feel embarrassed?
82. *R; It’s because we are different people, some people feel shy when they are in a group of other people or when they see a member of the opposite sex they fail to do things or speak.*
83. I: Okay so the way you know your friends, do you think they can be comfortable to come to the clinic?
84. *R: I think there just needs to be a partition between where men go and where women and girls go.*
85. I: But not provide the service in one place?
86. *R; No.*
87. I: So how this should be offered at the VMMC clinic?
88. *R: I think it can all go well together.*
89. I: I am asking because you said there should be a partition.
90. *R: It’s because some people feel embarrassed depending on the infections that they have so that’s why I felt that there should be a partition.*
91. I: So how do you think is the best way to offer STI services at the Voluntary Medical Male Circumcision clinics? At what time point and where should the service be offered?
92. *R: Time?*
93. I: Yes when the best time to offer STI is services at the VMMC clinic. You earlier on mention the clinic flow so now I want to know what the best time in that clinic flow is.
94. *R: They should offer STI screening at the time someone goes for VMMC.*
95. I: Mm... They should do it each time a person comes for VMMC?
96. *R: Yes.*
97. I: They should do it in the surgery room?
98. *R: No in the HTC room.*
99. I: What are the concerns or barriers for this integration.
100. *R: Some people can have concerns considering their present sexual behaviors. They may have had sex with a girl whom they are not sure of her status so they may have fears to come for screening thinking that they may be infected.*
101. I: How can we deal with such concerns?
102. *R: You can be sensitizing us and motivating us to have no fear of coming to the clinic and accessing such services*
103. I: Okay. Now I want us to talk about family planning. Tell me what happens when a person comes to the VMMC clinic to access family planning?
104. *R: Like a man?*
105. I: Whether a man or a woman but seeking family planning services. Have you ever encountered such situations?
106. *R: Yes.*
107. I: How did you help such a person?
108. *R: Ah, I have never faced such a situation only cases where men want VMMC.*
109. I: You have never faced such a situation?
110. *R: No.*
111. I: You have never even heard what happens when a man or woman wants family planning?
112. *R: No.*
113. I: So there are different family planning methods some of which are for men and others for women. And for women there are also different methods.
114. *R: That I know.*
115. I: As a peer what is your opinion on integrating Family planning in Voluntary Medical Male circumcision services/clinic? So that men and their partners can be coming to access these methods.
116. *R: I think it’s possible for those that want to be on a method.*
117. I: So you say it’s a good thing?
118. *R: Yes, it’s very important.*
119. I: What is it that you would not like about integrating Family Planning services in VMMC?
120. *R: The problem can be there if some people start family planning before they get married.*
121. I: What concerns or barriers can be there for this integration?
122. *R: Some men cannot accept it because say you have done VMMC but you are not yet married and they tell them about family planning. Such men can refuse to get family planning because they may want to have children first.*
123. I: How do you think family planning can be offered at the VMMC clinic?
124. *R: they can be told about family planning at the time they are in surgery.*
125. I: They should tell him at the time he is in surgery when he is thinking about the surgery?
126. *R: [Laughing] I think that can be a problem. So maybe if they tell him after he has healed.*
127. I: Why do you say so?
128. *R: It’s because by then he may have been over the worries and thoughts about VMMC and he can then concentrate on the family planning message.*
129. I: Okay. So what do you think are the barriers and concerns for this integration?
130. *R: I don’t think there can be any concerns or barriers.*
131. I: Okay. Now let’s talk about cervical cancer screening. You mentioned that VMMC reduces the risk of cervical cancer in women right?
132. *R: Yes.*
133. I: Tell me what happens when someone wants to get cervical cancer screening?
134. *R: For that I don’t…*
135. I: You have never been faced with such a case.
136. *R: I have never been faced with such a case but I know that women get infected if their partners who have not done VMMC.*
137. I: As a peer, what is your opinion on integrating partner cervical cancer screening with Voluntary Medical Male circumcision services
138. *R: I think it’s a good idea because for men who are not circumcised, their penises are harbor infections which can cause cervical cancer in the women. So the whole purpose of VMMC is to reduce the risk of cervical cancer.*
139. I: What is it that you like about this integration?
140. *R: It can be good because it can make us men to be clean.*
141. I: Okay, how will that be since it’s women who get screened for cervical cancer?
142. *R: Then that can be another way.*
143. I: The question is trying to say: say for example you have come for VMMC together with your partner so that she gets cervical cancer screening as you get VMMC. So if this scenario is really possible, what would you like about it?
144. *R: I can be happy because I can get VMMC and she can get cervical cancer screening. This can help because both of us will get a service when we come here.*
145. I: What is it that you would not like about this integration?
146. *R: There is nothing I wouldn’t like about it.*
147. I: How should screening for cervical cancer be done?
148. *R: They should do it at the time when the man goes into surgery.*
149. I: Not that you should go together.
150. *R: Yes, there can be two different ways of doing things.*
151. I: Why would you not like to go in together?
152. *R: It can also be possible that both partners go in together because just like in HTC they can opt to go in together or go in one by one.*
153. I: So who should be screened for cervical cancer?
154. *R: Like between boys and girls?*
155. I: No, like what kind of women should be screened for cervical cancer?
156. *R:[No response]*
157. I: Should it be any woman or they should select a certain group of women to screen?
158. *R: It should be every woman that comes for the service.*
159. I: Okay so what do you think can be the barriers for this integration?
160. *R: I don’t think there can be any barriers.*
161. I: No barriers?
162. *R: No.*
163. I: Won’t there be any concerns from men?
164. *R: No I don’t think there can be any concerns from them.*
165. I: Okay. Now let us talk about PrEP. Have you ever heard about PrEP?
166. *R: Yes.*
167. I: What do you know about it?
168. *R: That’s it’s a drug that you can take if you have had unprotected sex with a girl whom you know has HIV.*
169. I: Oh okay. So what you have described is PEP. There are two of them, one is called PEP and the other PrEP.
170. *R: R: Oh so I know PEP.*
171. I: They sound alike but they are different because there is an “r” on PrEP. PrEP is an anti-HIV drug which helps to keep HIV-negative people negative. There is a pill that one needs to take every day to prevent HIV infection. It is almost the same as PEP only that with PrEP you have to take the pills daily. So how do you feel about PrEP?
172. *R: How I feel about PrEP?*
173. I: Yes
174. *R: What did you say is the prescription for the pills?*
175. I: You take a pill every day.
176. *R: What if you don’t want to have sex?*
177. I: There is nothing like “I don’t feel like having sex today so I won’t take the pills” No. Whenever you have started taking the pills you need to continue taking them. On the day that you have sex with someone you will be already protected.
178. *R: Don’t they have side effects?*
179. I: We don’t know about that but let’s just assume that they don’t have any side effects.
180. *R: But they protect you?*
181. I: Yes they do.
182. *R: I feel it’s a good thing.*
183. I: Why do you say so?
184. *R: It’s because it can protect you from infection.*
185. I: What reasons would you have to encourage someone to take them pills?
186. *R: I can tell them that this drug will protect him from being infected*
187. I: [laughing] Now do you think it’s necessary for PrEP to be made available to HIV-negative men and women?
188. *R: Yes I feel it’s necessary because if these pills are given to someone who has done VMMC then he can be fully protected*
189. I: Would you encourage someone to be taking the pills?
190. *R: Yes, I can encourage them.*
191. I: Mm... If PrEP become available, what is your opinion on integrating PrEP with Voluntary Medical Male circumcision services?
192. *R: It can be good because it will protect people from infections.*
193. I: Mm... Okay it can reduce the risk. How do you think PrEP would be offered in this clinic?
194. *R: It can be offered to those that have done VMMC*
195. I: Mm... So they should be giving to those that have done VMMC?
196. *R: Yes, they should give to those that have done VMMC only.*
197. I: Why is that?
198. *R: It’s because if they give to those that have not done VMMC they can still infect women with some infections like with cervical cancer. Of course they cannot infect them with HIV but as for cancer it is possible.*
199. I: What would be the concern or barriers for this integration?
200. *R: I don’t think there can be any barriers*
201. I: Now let’s talk about other services. If you were given powers to choose and integrate services in Voluntary Medical Male Clinics, what are the services that you would think of Integrating?
202. *R: I can opt for VMMC, then providing PrEP for those that are HIV negative.*
203. I: Mm, why have you chosen PrEP?
204. *R: [laughing]It can help protect people from HIV*
205. I: So they can just be taking PrEP instead?
206. *R: Yes*
207. I: Alright. Thank you very much for your time. Your answers will be very helpful in improving the health service delivery at circumcision clinics.
208. *R: Mm, thank you.*
209. I: Before we close, is there anything more you would like to say?
210. *R: I feel we have already talked about a lot of things.*
211. .I: Okay. Thank you very much for talking to me today.
212. *R: Thank you.*

END

**D 43 STUDY**

**Date of Interview: 11 August 2018**

**Type of Participant: Peer or Clinic Aid**

**Interview Number: D-43-0023**

**Interviewer: I.N.**

**Total Interview Time: 30 minutes 16 seconds**

**Interview Summary:** **(from summary sheet)**

| **SERVICE TO BE INTERGRATED** | **THOUGHTS ON INTERGRATION** |
| --- | --- |
| Couple HIV Testing and Counseling | Things it’s a good idea as everyone will be knowing his status |
| STI Services | Thinks it’s a good idea as you will be knowing that everything is in its place without STIs |
| Family Planning | Think it’s a very good idea. Most men do have shy to get family planning because that issue is taken as for women. So if we integrate, in circumcision went more men, this could not be difficult for men to get family planning |
| Cervical Cancer Screening | Thinks it’s a very good idea. |
| PrEP | Thinks it’s a good idea as it will protect one’s life |
| Other Services | PrEP, cervical cancer screening and family planning |

**Remarks:**

**Participant was confident, open and easily understand the questions. He literary had no knowledge about PrEP.**

1. I: Thank you for taking the time to talk with me today. I would like to ask you some questions today about the way you feel and what you think about some issues related to the service you provide and how we can include other services in Voluntary Medical Male Circumcision (VMMC) clinics
2. *R: Mm…*
3. I: There are no right or wrong answers to these questions
4. *R: Mm…*
5. I: We would like to hear your opinion and your experiences in your own words
6. *R: Alright*
7. I: Do you have any questions before we begin?
8. *R: There is no question*
9. I: Can you tell me how you are involved in the client care at this clinic?
10. *R: I do take part since as I walk, I could explain the goodness about circumcision but also because when you circumcise, you can give a woman cancer disease. So, we could also explain this issue to the women that if a man goes for circumcision, the good thing is that, he cannot give a woman cervical cancer but also the man himself remains hygiene. That’s how I do explain, when I meet with people*
11. I: Okay. Aaah, so when you explain to people, what is your intension? So that they could come to the hospital or?
12. *R: I explain that, this issue of circumcision, we went to the hospital but also women should go to the hospital for cervical cancer screening due to coming of this cancer*
13. I: Does your peers come to the hospital?
14. *R: like a lot*
15. I: Ah, does your peers talk to you about how the services are provided here?
16. *R: Yes, they also appreciate so much like you helped us a lot, even I do stay well with my wife. She thank me that, it is really good.*
17. I: Ah, can you give me an example of a time that a Peer talked to you about the services he received here?
18. *R: Example of how they received the service?*
19. I: Yes.
20. *R: When a person can he explained, he undresses and tell me like nowadays when I do sleep with a women, things works well because I do live with them without cuts as before after sex I could found with bruises but now it is not happening. That is what they appreciate so much*
21. I: Mh. So, let us talk about partner HIV testing. That is one of service we would like to integrate at circumcision clinic. Yea, so now let us talk about partner HIV testing here at the Voluntary Medical Male circumcision clinic. Tell me what happens if a man brings a spouse here at the Voluntary Medical Male circumcision clinic?
22. *R: When a man come her for circumcision, they get their blood tested and they get their BP tested and Diabetes. That happens when they come for circumcision.*
23. I: Mh.
24. *R: Yes. And they explained the good things of doing this*
25. I: Have you ever seen a man bringing his spouse here?
26. *R:I have seen them coming with their spouse*
27. I: Ah, what do you think are the motivators that make the men bring their spouses here for testing?
28. *R: There are other men who we do explain to their wives who do take part in explaining to their husbands for circumcision. Most times we could talk to women and so they do explaining those problems to men. So they could escort them… yea*
29. I: But have you ever seen a man bringing his spouse for HIV testing here at circumcision clinic?
30. *R: Ah, I have never seen them. They have never come together*
31. I: What do you think demotivates men to bring their partners here for HIV counselling and testing?
32. *R: Maybe it is shyness from men to explain to their wives. Maybe that’s why they do not come together*
33. I: Okay. What do you think can be done to make men bring their partners here for couple testing and counselling?
34. *R: There is a need to recruit more volunteers so that when we are explaining to people about circumcision, we should also explain that when you are going you should take your spouse to test for blood because this issue was never integrated, we were only talking about circumcision and family planning. If we integrate this issue, people will be coming together*
35. I: You as a peer, what is your opinion on integrating couple counseling with Voluntary Medical Male circumcision services?
36. *R: This could be good, because everyone will be knowing his status than only a man knowing, which mean it could be good thing yea*
37. I: Mh. Alright. What do you think are the barriers and concerns on this integration?
38. *R: maybe transportation since children we could get them in cars, maybe if have big cars, those people can be coming without problems. Or going in the villages and place close to the circumcision places, could also help.*
39. I: Mh, which means the concern there can be
40. *R: Distance of the place as we are doing at (Name of location), some come from (Name of location), it’s very far for a person to reach there. So people look at it as far. So if we could put close [circumcision clinics]*
41. I: Mh, alright. Now I would like to discuss with you about sexual reproductive health services and Pills HIV prevention: called pre-exposure prophylaxis. (PrEP). Sexual reproductive health include services that promote good sexual health and reproduction. They include but not limited to family planning, sexual transmitted infection management, cervical cancer screening, Condom distribution and many more. Today we will only discuss about family planning, diagnosis and management of STIs, Cervical cancer screening, and PrEP. We will look at each of these one by one. Let us start with STI services. Explain to me what happens if a client is suspected or diagnosed with an STI here?
42. *R: Ah, if he came here and found with this disease, they go to… they first look at his immunity, that how low it is, whether we are going to circumcise him or not. If they see that, their immunity is well, they could get circumcised. That’s how it is*
43. I: So if the immunity is not well
44. *R: The service providers do fix a way which they know by giving, whether they know so that your immunity is in its place and circumcised when the immunity is in its place*
45. I: Mh. So do they send him back or what happens?
46. *R: By that time when it is at Kang’oma, they could send them to a big hospital, so that they could see what they have to done.*
47. I: Mh. Hmmm… You as a peer what is your opinion on integrating STI services with circumcision services? What would you like?
48. *R: What I would like, is that it could be a good thing because everything you will be knowing that everything is in its place. I don’t have that kind of the diseases, they have also screened me, and everything is also well. That could also be very good thing*
49. I: Okay. Does that mean you agree with integration?
50. *R: I agree with it. It is very good thing*
51. I: What is it that you would not like the integration of diagnosis and management STIs with Voluntary Medical Male Circumcision services?
52. *R: What I would not like is that the offices should just separate, this for circumcision and the other one for screening what is needed to be tested. The offices should be like separate but that is really good*
53. I: So when should this service offered?
54. *R: That service should be offered the same time because a person come from home to receive treatment. That is needed to be received that time*
55. I: Mh…
56. *R: Yes.*
57. I: So like in the circumcision process, like you have come for registration, has been screened, has circumcised. When should this be placed?
58. *R: Maybe after receiving the circumcision, they can look for other things and given treatment then going home*
59. I: Like after circumcision, then that is when they will be screening?
60. *R: Yes…*
61. I: If has STIs?
62. *R: Yes…*
63. I: But the hospital should be
64. *R: The same one like the way it is when we just arrived that at circumcision, they go and test for BP, diabetes… it is found that at the end they got circumcision. It should be the exact way*
65. I: It should be the same way?
66. *R: Yes. That when they start, testing here and finally circumcision, there is no problem*
67. I: But in separate rooms?
68. *R: Separate rooms*
69. I: Okay. Aaah, what do you think are the barriers and concerns on this integration?
70. *R: The concern is that when we want to tell people, we could only pass through big roads only while we don’t reach small roads to reach people well. Those cars used for spreading information pass through big roads, other areas, do not get reached. That is the concern which is found.*
71. I: Okay. So what do you think should be done to address these concerns and barriers?
72. *R: This concern can be addressed as we who do this job of mobilizing others, we should people who are serious with this work so that we could reach were cars do not reach, explaining to them well about this issue for instance right now others don’t know that they have opened a period for circumcision. We are reaching them that did they open because they haven’t been reached*
73. I: Alright, let us talk about family planning. Explain to me what happens if a client needs a family planning methods
74. *R: If a person need a family planning method, goes to the hospital and explain that I need a family planning method*
75. I: Mh…
76. *R: Yes…*
77. I: What family planning methods do you know?
78. *R: There is loop. So there is that of pills, which you could be just protecting yourself… yes*
79. I: Mh... How about men?
80. *R: Men they do also have family planning method*
81. I: Mh. What method is that?
82. *R: That method I have just forgotten it but it is there*
83. I: Okay. Alright, so as a peer, what is your opinion on integrating Family planning in circumcision services?
84. *R: There since most men we do have shy to get family planning because that issue we take as for women. So if we integrate, in circumcision went more men, this could not be difficult for men to get family planning*
85. I: Mh…
86. *R: Yes.*
87. I: So what is it that you would like about the integration of Family planning with Voluntary Medical Male Circumcision services?
88. *R: What I would like since at male circumcision goes more men, I would like this because it can be good*
89. I: Mh…
90. *R: Yes…*
91. I: What else would you like?
92. *R: What I would also like is that right now what is needed, more men should be reached so that they should know about this issue, so that they can be going for circumcision and family planning*
93. I: Okay
94. *R: Yes*
95. I: What is it that you would not like about the integration of family planning with Voluntary Medical Male Circumcision services?
96. *R: Mm… maybe if people are not given enough information, it could be difficult for people to know*
97. I: Mh…
98. *R: Yes.*
99. I: Mh… How do you think family planning services would be offered within Voluntary Medical Male circumcision clinics?
100. *R: Here, its advantage is what we are looking now of thanking that people have started moving, spreading information that people will receive family planning at that place, circumcision at that place that I see it that it has started well because I have been hearing that people will receive at that school, at that hospital. I have seen it that it has started well*
101. I: Where should family planning place in circumcision process?
102. *R: This issue should be put right there were they are spreading about family planning. They are spreading about family planning and about family planning they are not. So it is like it is different there but these need to be integrated because I have been trying in the houses that they have now started circumcision but also family planning go to the hospital. Women receive it well because before we start explaining to them about circumcision we start with family planning issues because we know that if we start with family planning, they will be disappointed. After explaining about family planning, cervical cancer then we talk about circumcision, so women receives those things well and explain to the man. As a result, we go in the morning and get a man for circumcision. That’s the good thing*
103. I: Like at the circumcision clinic, when could this service be offered?
104. *R: At the clinic.*
105. I: After a man circumcising or before circumcising or where should it be place?
106. *R: This needs first a man has to get circumcised, after explaining everything to him after arriving that there is this this this… family planning, there is circumcision, so how do you copy with it. The man will accept them, circumcise, receive and going home. That means we have done those things without a problem.*
107. I: Should it be offered within the clinic or outside?
108. *R: This should not be within the same clinic, because the doctors get tied up with work, so it’s like people see as a wastage of time, then they get discouraged to go and get family planning. Where circumcision is being done should not be combine with others, as family planning should also be somewhere else.*
109. I: What do you think are the barriers and concerns with the integration family planning and Voluntary Medical Male circumcision services?
110. *R: My concern is that they will be slow in terms of working and other people get mad like they have get us from home and look on how they are working, they are too slow and this is a concern as a person can say am rushing for something else to what I came here for and what is happening is different. That is our concern and we do start our work late after finding that the car are not there, found that we are just staying at the road from 8 up to 12, people haven’t eaten anything. That is what people just say that we are going back [home]. And we do worry a lot like we do work for nothing*
111. I: What do you think should be done to address these concerns and barriers?
112. *R: This need us and those we are working together we should agree on what to do, when we give them a phone call, that we have found people, and come at a good time to pick them or rushing where the clinic is placed so that they reach in a good time. This will help us who mobilize that what we explain to people is really happening.*
113. I: Let us talk about cervical cancer screening for female partners. Explain to me what happens if a woman needs cervical cancer screening?
114. *R: After reaching the hospital?*
115. I: Yes.
116. *R: Their cervix is supposed to be screened for cancer so that they can receive treatment. If they don’t have, they get told that your cervix is fine, go back. That is how it works*
117. I: As a peer, what is your opinion on integrating cervical cancer screening for female partners with Voluntary Medical Male Circumcision services?
118. *R: That because as I have explained after meeting a woman with the aim of meeting a man, we start with explaining the good thing about family planning, the cancer which is found, so the woman influences her husband. I also believe that when going to that place, they will go together. So it cannot be difficult to be done.*
119. I: What is it that you would not like about the integration of partner cervical cancer screening with Voluntary Medical Male Circumcision services?
120. *R: Maybe because women may be feeling shy since there are men at that place, so maybe they might be having shy because of issues like that. So there is just need to make it clear, how we can put in distance between men and women, it should a small difference so that they should not feel shy*
121. I: So, how do you think is the best way to offer cancer screening within Voluntary Medical Male circumcision clinics?
122. *R: The screening is just happening in the clinics but the way they do with family planning is not happening, they just explain about receiving medicine for family planning but screening is done in the hospitals, so if there can be a chance that were they are walking people should also be screened that they could not take long distances going to the hospitals, that could be of help.*
123. I: Okay but like here since we are talking of this service to be at circumcision clinic
124. *R: Yes.*
125. I: At this clinic…?
126. *R: Yes.*
127. I: Where should this service be offered here if we integrate? Offered when?
128. *R: Time when, it could happen that this issue of cervical cancer, is screened by a man since it screened to a women, it could happen that a man is receiving circumcision there while a women is also receiving her service*
129. I: At the same time?
130. *R: Yes since a woman is just escorting while the man is receiving circumcision, the woman is also screened for cervical cancer*
131. I: should it be the same clinic or outside?
132. *R: It could be the same clinic but maybe after making separate place for women and men so that men should not be shy or women should not be shy*
133. I: Ah, what do you think are the barriers and concerns about integrating partner cervical cancer screening and Voluntary Medical Male Circumcision services?
134. *R: My concern is that when there will not be a clear strategy on integration of men and women, it could happen that others will be feeling shy, but there has to be a place where people could be explained clearly about this issue, then women can be go their side and so do men, so it could be that they will not be feeling shy or women should have their place where they would be counselled about this and so do men, that could be of help. They could not be feeling any shy*
135. I: What do you think should be done to address these concerns and barriers?
136. *R: This concern can be addressed by health officials that they could make orderly place, were people could not feel shy when going for screening*
137. I: Okay. Now let us discuss about PrEP. Have you heard about this before?
138. *R: I have ever heard about PrEP but I don’t understand what it means*
139. I: What did you heard about PrEP?
140. *R: Aren’t PrEP family planning?*
141. I: No, it is not family planning
142. *R: What is it?*
143. I: PrEP is anti-HIV medicine that keeps HIV-negative people from being infected. I can give you an example, a man might be having a virus while his wife not, if the women is taking PrEP, she cannot get a virus from her husband. There is a single pill that is taken once daily, and if you take it regularly, it is highly effective at prevention people from being infected.
144. *R: What I heard is that which a person take when is pregnant, so that cannot have a baby. Maybe it is that one and it is a bit different*
145. I: We can say that is it a bit different. So, if a person ask you what PrEP is, what can you tell him?
146. *R: I can answer that are medicine which you take which helps not get disease while your partner has a disease through sexual intercourse*
147. I: Alright. If PrEP was made available to HIV- men and women. Do you think you could advise your HIV negative peers to accept to take PrEP?
148. *R: Those who doesn’t have?*
149. I: Yes.
150. *R: Those who doesn’t have could be difficult to take them since they are already well but those who have or one of you have, should be taking*
151. I: What are the reasons you would encourage your clients to take PrEP?
152. *R: I can encourage them, it is a good news because you can protect your partner so that cannot get any disease, so it is a very good news*
153. I: If PrEP becomes available, what is your opinion on integrating PrEP with Voluntary Medical Male circumcision services?
154. *R: And also this issue is very important because it is one which is helping someone’s life. So this one should be integrated on circumcision and the explanations which people will have about these pills is very good.*
155. I: Would you encourage clients to take PrEP?
156. *R: Very much and this issue is very good for influencing people to go for circumcision and also to receive different medicines. That could be one way of copying people*
157. I: What do you think could be the concern and barriers to integrating PrEP Voluntary Medical Male Circumcision services?
158. *R: My concern is that when we explain this to people, in those places it will be happening in the right time but if it will not be happening in the right time, people question as that what you taught is not happening, that’s our concern*
159. I: What do you think should be done to address these concerns and barrier?
160. *R: We who do mobilize people and health service providers we should be working together, that could be very good thing*
161. I: At circumcision clinic when do you think PrEP should be offered? Or where
162. *R: This issue could be at the same clinic, other place which could be built through which after people have been explained they will be going to receive there*
163. I: Like for men who have come for circumcision
164. *R: Men who have come for circumcision after explaining to them, they will be going to receive, feeling relieved as doing three things at once*
165. I: After circumcision or before
166. *R: After circumcision because after they have been explained about this issue*
167. I: Okay. Now let us talk about other services. If you were given powers to choose and integrate services in Voluntary Medical Male Clinics, what are the services that you would think of to Integrate?
168. *R: I could integrate family planning, but also receiving pills of PrEP, this one is very good to be integrated. Yea but also cervical cancer screening. It is very good, it needs to be included at this place*
169. I: Three?
170. *R: Three…*
171. I: Explain to me what the reasons are for your choices like PrEP, cervical cancer screening and family planning
172. *R: These services as for me especially PrEP, one a person will be protected from his family. From there we have to go to cervical cancer screening, which mean everything for her will be in its place while in family planning, it is very good because you have found it without difficulties, that I would like*
173. I: So, how do you think these services would be offered in the clinic? Like PrEP, family planning and cervical cancer screening
174. *R: I hope health specialist can make clear strategies like how we receive other medicine like we could put a person here, after receiving here… in order. It can work without problems*
175. I: On your own, how do you think PrEP should be offered?
176. *R: PrEP could be offered on a private place, where when a person go, cannot be seen because this issue it is supposed be secret. So this could be at a place where other people cannot notice that you have received these medicine*
177. I: How about family planning?
178. *R: Family planning, the same because others feel shy for others to know just like circumcision we could take people like we are going for a chat, they don’t want people to know that they have gone there. If you don’t approach them well instead of accepting that thing, they do refuse it. You can explain in a group and call boys aside, explaining clearly and you will find that a mother who was refusing accept it*
179. I: So how about cervical cancer screening?
180. *R: It’s just the same*
181. I: It’s just the same. So thank you for taking your time to discuss with me today. Your answers will be very helpful in improving the health service delivery at Voluntary Medical Male circumcision clinics. Before we close, do you have anything to say?
182. *R: Yes, I do have. As for me, am just appreciating that, government have think well about this because we do this job, maybe just recruited and posted there but they don’t ask us the problems which we do face or which can help them, it does not happen. I see it as a big opportunity to be called, asked… on that am thankful, it should continue so that we could meet time to time so that we could explain our concerns which can help health services and that am very thankful*
183. I: Thank you. Again thank you so much
184. *THE END*

**D43 STUDY**

**Date of Interview: 17 August 2018**

**Type of Participant: Peer and clinic aides**

**Interview Number: D-43-0033**

**Interviewer: I.N.**

**Total Interview Time: 39 minutes** 00 **seconds**

**Interview Summary:** **(from summary sheet)**

| **SERVICE TO BE INTERGRATED** | **THOUGHTS ON INTERGRATION** |
| --- | --- |
| Couple HIV Testing and Counselling | Thinks it is very important, because circumcision protects men from contracting HIV so if couples for HIV testing together, they could know their status together and follow instructions properly. |
| STI Services | Like the integration |
| Family Planning | Thinks it is a good thing because after circumcision you become protected from diseases while with family planning you are not fully protected. |
| Cervical Cancer Screening | Thinks it is a good thing because when a person is circumcised he is protected from other diseases and has protected the woman from cervical cancer. |
| PrEP | Thinks it’s a good thing because if you cannot manage to abstain, you can choose PrEP to protect yourself from the infection |
| Other Services | None |

**Remarks:** Participant was relaxed, calm and knowledgeable.

**Interview Texts:**

1. I: Thank you for taking your time to talk to me today, I would like to ask you some questions on how you feel and the services you received here and how we can include other services in the voluntary male circumcision clinics. There are no right or wrong answers to these questions. We would like to hear your experiences in your own words. Do you have any questions before we begin?
2. *R: no. I don’t have.*
3. I: Can you please tell me what role do you play in the services that are offered here at the VMMC clinic?
4. *R: what we basically do is that we go into the communities to inform people especially young men on the benefits of circumcision and the dangers of not getting circumcised. We try to inform their parents so that one day they may make a decision to send their children to the VMMC clinic for circumcision.*
5. I: with your friends, do you ever talk about the services offered at this clinic?
6. *R: Yes. They are really able to talk.*
7. I: would you please tell me an example of the moment whereby your friend talked to you about the service they received here?
8. *R: here was a certain time when a man that we reached out to in the community came for circumcision, so he got interested that when you are circumcised you are half way protected from AIDS.*
9. I: Now let us talk about HIV couple testing at this clinic. Tell me what happens when a man brings a wife here at the voluntary male medical circumcision clinics?
10. *R: when a man brings their spouse here, what happens is that because here we do circumcision, so we mostly deal with men, so we test only the husband for HIV and we don’t test the woman and when they are infected we don’t circumcise them.*
11. I: have you ever seen a man coming with a woman for testing?
12. *R: so I will lie on that one. I have never seen any.*
13. I: what do you think makes men not to be bringing their spouses for HIV testing?
14. *R: maybe largely it would be shyness to bring their spouses because if they are going together, they test their blood so they are usually afraid that when the other partner knows they are infected it won’t be good. They would rather keep that secret from their spouse.*
15. I: what do you think should be done to make men start bringing their spouses here for HIV couple testing?
16. *R: if a centre is to be established where by these services will be provided in separate rooms. Firstly, partners would be going together for the HIV testing and then the man will proceed to the circumcision room. This is because if the tests are done separate they may not be able to tell each other about their status.*
17. I: so in your opinion, what are your thoughts about bringing your spouses to the VMMC clinics for HIV testing?
18. *R: this is very important, because firstly, circumcision already protects men from contracting HIV so if couples for HIV testing together, they know their status together and follow instructions properly.*
19. I: but what do you think are the barriers and concerns of this integration?
20. *R: maybe when they come here for HIV test, because when a person is found positive they don’t proceed with the circumcision because by this time their body system to fight infections is weak so creating a wound would make the matters worse.*
21. I: what do you think should be done to resolve that concern?
22. *R: what’s important is to have one Centre with various departments that would be able to offer these services. So that they can be making choices to come just for HIV test or circumcision in these different centres.*
23. I: y centres you one clinic separated into rooms or establishing them outside?
24. *R: I think these should be rooms because it requires privacy so it can’t work outside because like the issue of HIV testing will just need a separate room within the clinic so that people should do the tests and then proceed with the circumcision or not.*
25. I: o where within the circumcision process do you think this integration should take place?
26. *R: I think the time that the couple has come for an HIV test, this is the best time they can decide whether to do circumcision or not. In instances where they test negative they would go ahead with the circumcision to remain protected.*
27. I: kay fine. Now I would like us to discuss about sexual reproductive health services, pills for HIV prevention called pre-Exposure Prophylaxis. sexual reproductive health includes services that promote good sexual health and reproduction they include but not limited to family planning, cervical cancer screening, Sexually Transmitted Infections management and condom distribution and many more. Today we will only discuss about family planning, cervical cancer screening, Sexually Transmitted Infections management and PrEP. We will look into each one of these one by one, so let’s start with STI services. Explain to me what happens when a person is being suspected or has been diagnosed with STIs?
28. *R: when a person is suspected or been diagnosed with STIs at this clinic, that person is not eligible to undergo the circumcision process because at this stage his body becomes weak so for instance if he is diagnosed with Gonorrhoea they are supposed to take medication and recover then come back for the circumcision.*
29. I: So you as a health service provide support personnel, what is your opinion on integrating STI screening within the VMMC clinics?
30. *R: here because you have been circumcised, you become circumcised. The penis if not circumcised, the foreskin keeps a lot of things and then you risk infecting a woman with diseases when having sex and you can even infect women with cancer.*
31. I: so what things are you not comfortable with regarding the integration of STI management and VMMC services?
32. *R: what I can say is that male circumcision is a good programme which can be used to reduce the number of people contracting the HIV virus and STIs and also you can still contract the disease, so what I don’t agree with is that even after circumcision you still need to be using condoms.*
33. I: so we should say that you are okay with the idea of integrating these services?
34. *R: yes, I agree to this idea.*
35. I: what do you think are the best ways of providing the STIs services here at the VMMC clinic?
36. *R: maybe just encouraging people to come for circumcision and explain to people that when you are circumcised you become protected from diseases and the dangers of not being circumcised. Because even during sex those that are circumcised take longer to ejaculate during sex and that brings satisfaction to women.*
37. I: okay. But in terms of time and place within this clinic, how should these services be offered here?
38. *R: in terms of time, I think it should be done when a person has come for HIV test that’s the best time to do the circumcision and place should be of some sort of privacy because people are shy to be seen in such places.*
39. I: what do you think are the barriers and concerns of this integration?
40. *R: the only little concern that can be there is that because before a person undergoes circumcision they first test their blood, people may be shy since when they test positive and don’t proceed to the circumcision room people will be able to make a judgment about their status.*
41. I: so how do you think that concern can be dealt with?
42. *R: stablishing a room that can offer the STI screening together with the circumcision service so that people shouldn’t be able to make judgments.*
43. I: let us now talk about family planning, what have you heard about family planning?
44. *R: I have heard that there are a lot of methods that women use such as in plant, condoms.*
45. I: okay. Vasectomy, ever heard about vasectomy?, men also do family planning.
46. *R:no I have never heard about that one.*
47. I: explain to what happens when a person wants a family planning method here?
48. *R: since this is a circumcision clinic, when such people come here we just advise them on the available family planning methods and also we give them condoms after circumcision which is a family planning method but then we refer them to another hospital for such a service.*
49. I: so you as some health service provider support personnel, what is your opinion on integrating family planning within the VMMC services?
50. *R: if they integrate these two it will be a good thing. So I should explain, this is because after circumcision you become protected from diseases while with family planning you are not fully protected.*
51. I: so you are okay with this integration?
52. *R: no I don’t really agree.*
53. I: what do you think are the best ways of providing family planning services at the VMC clinic?
54. *R: the best way is to explain to the clients both the man and the woman and let the make a choice of the method they would like to use because condos are not hundred percent but to make a decision like of in plant that will take time before expiry.*
55. I: like in terms of place, where should this family planning be offered?
56. *R: family planning should be offered when a person has come, done the HIV test, results negative, and do the circumcision but that should be done when they have come as a couple.*
57. I: but in what place, this same building or?
58. *R: it should be in a separate room that people can openly talk about the services.*
59. I: what do you think are the barriers and concerns to this integration?
60. *R: the barrier that can be there is that if a person has done a family planning method but has not been circumcised they are still at risk of being infected of HIV and also to pass the cervical cancer to their loved ones.*
61. I: how do you think this concern can be dealt with?
62. *R: before you do family planning, test them first to know their status and also and the husband should now do th4e circumcision and then make a choice for family planning method.*
63. I: so this integration are you okay with it or not?
64. *R: I agree to it a little bit because, when you are circumcised you become protected and the woman is also protected with family planning.*
65. I: how let us look at cervical cancer screening, what do you know about cervical cancer?
66. *R: what I can explain is that when a woman is coming to the clinic for cancer screening, they are advised to make sure their vagina is clean because they insert certain tools and materials during screening. Like cotton which is first immersed in spirit to wipe the vagina and then another cotton swab that is first immersed in vinegar that is now used to determine the cancer availability based on the colour changes of the cotton swab.*
67. I: have you ever seen or you just heard?
68. *R: just a little bit I have ever seen because my brother is a nurse.*
69. I: explain to me, what happens here when a person wants to do cervical cancer screening?
70. *R: when a person has come for blood test is the same time that they do cancer screening here and even when they have come for family planning.*
71. I: but then in the case of this clinic, have you ever seen a woman requiring cancer screening?
72. *R: o I have never seen.*
73. I: alright. So in your own opinion, what do you think about the integration of cervical cancer screening within the VMMC clinic?
74. *R: what I can say is that when a person is circumcised he is protected from other diseases and has also protected the woman from cervical cancer. When you are not circumcised, the penis may store some dirty things which may cause the cancer in women.*
75. I: so if we are to integrate these two services, how do you look at that issue?
76. *R: that can be a very good thing.*
77. I: what can make you not to want this integration?
78. *R: ah on this one, I don’t there is something I don’t like about this because they will all be protected maybe the risk will only come on family planning.*
79. I: how do you think cancer screening service should be offered here in terms of time and place?
80. *R:in terms of place a separate room should be established for the screening and circumcision we already have that room and then the best time is when they have undergone the blood test.*
81. I: what do you think can be the concerns and barriers to this integration?
82. *R: if you circumcised you are protected as well as when you have done the cancer screening but if you did not do family planning it becomes a challenge. Sometimes men are shy to bring their wives during circumcision and women may also not be comfortable to come with their husbands.*
83. I: how do you think these barriers or concerns can be resolved?
84. *R: we should just continue encouraging people to be coming for circumcision and cancer screening because with circumcision you are protected and also cancer screening helps the woman to know how they are in their body and helps in early treatment commencement.*
85. I: how let us talk about PrEP. Have you ever heard about PrEP?
86. *R: o.*
87. I: okay. So I will explain to you how it works. It is an abbreviation that means Pre-Exposure Prophylaxis. It is a drug that is taken by an HIV negative person so that it should protect the person from contracting HIV even if this person has sexual intercourse with an HIV positive person. It is usually confused with PEP but this is PrEP. Now how do you understand about PrEP?
88. *R: The way I have heard, I think it’s a good thing because if you cannot manage to abstain, you can choose PrEP to protect themselves from the infection.*
89. I: is it important to make PrEP available to people without HIV?
90. *R: Yes, it is necessary.*
91. I: so what reasons can you encourage people with?
92. *R: first, those that cannot manage sex with condoms, they can be taking PrEP so that they remain protected.*
93. I: if PrEP is to be made available, what is your opinion on integrating it within the VMMC service?
94. *R:it is a good thing, because even circumcision you can still contract infections, so PrEP will be very good even when you travel without a condom but you have met woman to have sex with and you taking PrEP you can still be safe.*
95. I: so you would be interested to tell people to be taking PrEP?
96. *R: Yes. Because there a lot of people who have not circumcised but they keep on sleeping with women without condoms even though it may lead to improper family planning.*
97. I: So that will be the concern to this integration?
98. *R: Yes, because once people start taking PrEP they will only be thinking that they won’t contract the infections yet they will just be making babies in the process.*
99. I: so what do you think should be done to end this concern?
100. *R: I think we will need to encourage people to be taking PrEP together with family planning methods to avoid unplanned pregnancies.*
101. I: So how do you think PrEP can be offered here at the circumcision clinic?
102. *R: I think this should be offered when you have come for circumcision, you have been tested negative and they should be told that even after circumcision you can contract the disease so they need to be taking PrEP.*
103. I: So in terms of place within the VMMC clinic, where should PrEP be provided?
104. *R: I think in another room where the counselling is done.*
105. I: let us look at the other part which is other services, if you are given the power to integrate other services to the VMMC services, what are the services you would think of integrating?
106. *R: I would integrate family planning and PrEP.*
107. I: These two only? Tell me the reasons for these choices.
108. *R: if you do family planning and using PrEP at the same time you are circumcised, you are safe from all critical issues and I should add cervical cancer screening to the list.*
109. I: so what do you think this service should be offered at the clinic?
110. *R: should be offered in the way that after the blood test, cervical cancer screening for the woman and the man does the circumcision, after the circumcision they go in another room for counselling for family planning so by this time they should have received all the services.*
111. I: thank you so much for taking your time to discuss these things today, your answers will be helpful in improving the health service delivery at circumcision clinics. Before we close, do you have anything to say.
112. *R: So I have exhausted everything.*
113. I: thank you for your time once again.
114. *R thank you very much.*
115. THE END.

**D43 STUDY**

**Date of Interview: 17 August 2018**

**Type of Participant: Peer and clinic aides**

**Interview Number: D-43-0034**

**Interviewer: I. N. Total Interview Time: 40 minutes 49** **seconds**

**Interview Summary:** **(from summary sheet)**

| **SERVICE TO BE INTERGRATED** | **THOUGHTS ON INTERGRATION** |
| --- | --- |
| Couple HIV Testing and Counseling | Integration of HIV couple testing with the VMMC services in the clinic is very helpful. |
| STI Services | This is a very good thing and it helps because when a person doesn’t know their health status so this is like you killing two birds with one stone |
| Family Planning | Think it is very important to integrate because to some people after circumcision they think that they have reduced the chances of contracting the virus disregarding condom use without thinking of pregnancy |
| Cervical Cancer Screening | Thinks it should be integrated because they are similar, the reason that men go for circumcision is because they want to protect women from cervical cancer so the department of circumcision should have another service for cervical cancer screening |
| PrEP | Thinks it will help because when people have done circumcision they will no longer be using condoms so I think this integration should happen in the clinics to protect such people from contracting the virus |
| Other Services | None |

**Remarks:** Participant was relaxed, confident and knowledgeable.

**Interview Texts:**

1. I: Thank you so much for sparing your time to talk to me today. I want to ask you a few question on the services you provide and your opinions on any additional services onto the services that is provided in Voluntary Male Medical Circumcision clinics. There is no right or wrong answers to these questions. We want to hear your understanding and opinions. Do you have any questions before we begin the interview?
2. *R: No. I don’t have any question.*
3. I: Would you please tell me the role that you play at this circumcision clinic?
4. *R: As we live we know that there are other people who haven’t yet done the circumcision so we reach out to them with information about the benefits of circumcision and the dangers and we encourage them to come to the clinic for the circumcision.*
5. I: Are your friends able to talk to you about the service that is offered at this clinic?
6. *R: Yes. They are able to ask questions like how does it go when you go to the clinic, you know people are afraid of circumcision and they have a lot of issues that discourages them but we encourage them that the pain for the wound are temporary but the benefits are long term.*
7. I: Would you please tell me an example of a situation whereby your friend ever talked to you about the service they received here?
8. *R: There was a time when my friend came for circumcision, he explained that the procedure is very good even hygiene wise as a man even if you don’t take a shower your penis is always clean so for him after circumcision he saw that hygienically it was a good thing to do.*
9. I: Okay fine. Now let us talk about going for an HIV test as a couple. This is one of the services that they are planning to integrate here at the VMMC clinic. So that couples would be able to come together and do the HIV test as a family. Explain to me, what happens when a husband brings his wife here at VMMC clinic?
10. *R: What I know from the time I came, when a person comes with their partner, they only do blood test for only the person undergoing the circumcision not the other partner this is because when they test to see your status that’s when the decision is made whether to circumcise you or not.*
11. I: But have you ever seen a man coming with his wife as a couple here?
12. *R: Yes, but this time around, the wife just escorted the husband so she didn’t proceed to do the test with the husband.*
13. I: What do you think makes men to bring their spouses for HIV testing?
14. *R: Partly, we would say it’s love or no having doubts about their status that they would allow their spouses test for HIV together with them.*
15. I: So what do you think makes men not to bring their spouses here for HIV test?
16. *R: It would be the opposite of that I just said, when you have doubts about your status when you start to think of the things you have been doing you may be thinking that it is possible that you can be infected.*
17. I: So what do you think can be done to make men able to come with their spouses for couple HIV testing?
18. *R: What should be done is that all these people should be given counselling so that when they all come they should be tested together not only one person so that everyone should be able to know his or her status.*
19. I: You as a service provider, what do you think about the integration of HIV couple testing on to the already available service of VMMC? We understand in the VMMC clinics they just provide circumcision service, now there is this new idea of integrating HIV couple service onto this already existing service, so what’s your opinion on this idea?
20. *R: Integration of HIV couple testing with the VMMC services in the clinic is very helpful because the time when a person has been tested and either diagnosed with or not with HIV. Those that test negative, when they go back after circumcision they should continue taking care of themselves because it’s not like after circumcision you become hundred percent protected from HIV there are still percentages of contracting the virus.*
21. I: What do you think the concerns or barriers would be of this integration?
22. *R: The barriers to this integration would be because it is not possible to do the circumcision whilst the wife is there since the room for HIV testing is different with where the circumcision is done. The other challenge would be if one tests positive the other partner may be frustrated.*
23. I: So what do you think can be done to resolve those concerns?
24. *R: To resolve those concerns, we should establish another place that the couple should be coming together for testing as a couple and that when a man comes with his wife for circumcision they should be told everything that happens at the clinic so that they should be prepared.*
25. I: Now I want us to discuss sexual and reproductive health and pills that protect from HIV which are known as PrEP. sexual and reproductive health includes among others, family planning, cancer screening, condom distribution, STIs management etc. Today we will only discuss issues around family planning, cancer screening, STIs management as well as PrEP. We will discuss each one of these issues in detail. So let us start with the issue of sexually transmitted infections. Please explain to me what happens when a person is suspected or has be diagnosed with STIs here at the VMMC clinic?
26. *R: When a person comes and has been diagnosed with STIs for instance syphilis, they send you back home with treatment and you are advised to come back when you have fully recovered. But usually when you have been diagnosed with STISs they don’t continue with the circumcision.*
27. I: So you as health care service provider support personnel, what do you think about the integration of STIS screening within the VMMC clinic?
28. *R: This is a very good thing and it helps because when a person doesn’t know their health status so this is like you killing two birds with one stone because you came for circumcision and then you have been given the chance to know your status so if diagnosed positive you start taking medications immediately thus you have been helped.*
29. I: So what things don’t you agree with this integration?
30. *R: one thing I don’t agree with this integration is the screening of only the husband because is the one doing the circumcision and leaving the woman not screened rather it should be made that both partners should be tested because it is possible to disclose the results of one partner to the wife yet the husband doesn’t know the status of his wife and the second one is that when a person has been tested and you have been diagnosed with STIs and then you go back home and come back I think it is better just to proceed with the circumcision and then give the person treatment to go and never come back again.*
31. I: So what do you think are the best ways of providing the STI screening here at the VMMC clinic?
32. *R: They should first screen blood to know the status in the laboratories with tools and equipment to assist the testing and another thing…*
33. I: But then let’s just consider the place and time when the STI screening can be offered within the circumcision process?
34. *R: There should be a special place where the screening should be done and also circumcision should be done in another department or room and in terms of time after testing they should immediately give you treatment and send you home to recover. It should be done in a private room because it is not possible for the woman to go into the circumcision room since there are other men there.*
35. I: What barriers do you think can exist with the integration of STI screening and management with VMMC?
36. *R: Some people are afraid to come because when they come they will be tested for blood so they are usually afraid because they are not ready to know their status.*
37. I: So how do you think this barrier can be resolved?
38. *R: I think they just need guidance to be told that it is a good thing to know their status and that when they have been diagnosed with the STIs they will be give3n treatment and when they are fully recovered they can come back for the circumcision.*
39. I: Now let us talk about family planning. Have you ever heard about family planning?
40. *R: Yes.*
41. I: What have you heard about family planning?
42. *R: Family planning is when a person has decided that when they have sex like for women should not get pregnant for instance in plant inserted that they should not bear children.*
43. I: What family planning methods do you know?
44. *R: There is in plant, another one is it kangaroo?... pills, and another one on the hand which women take.*
45. I: which method for men do you know?
46. *R: I know this one…. what do they call it? vasectomy which cuts the semen passage.*
47. I: So explain to me what happens when a person comes here seeking for the family planning service?
48. *R: When a person comes he meets with the responsible people for such services, so you tell them the method of your choice of family planning and they explain to you the benefits and side effects of each method until you make a choice of the family planning method of your choice.*
49. I: So you as health care service provider personnel, what do you think about the integration of family planning services within the VMMC clinics?
50. *R: I think it is very important to integrate because to some people after circumcision they think that they have reduced the chances of contracting the virus disregarding condom use without thinking of pregnancy.*
51. I: So do you agree with this integration?
52. *R: Yes, it is very good.*
53. I: But what can make you not to want this integration?
54. *R: The challenge is that men think that family planning is for women like for example most men would not be willing to go do vasectomy so they would rather let the wife go for it because it is another process.*
55. I: But do you agree to this integration?
56. *R: Yes.*
57. I: What do you think are the best ways of providing these family planning services here at the VMMC clinic?
58. *R: I think pills or in plant but also to men I think because it is only one method they can do vasectomy as well as condom use to prevent unwanted pregnancies.*
59. I: But in terms of place and time, how do you think the services should be offered?
60. *R: There has to be a separate place so that when a person comes from the circumcision room they should be going into the family planning rooms not providing the service before the circumcision.*
61. I: What do you think are the concerns and barriers to the integration of family planning services within the VMMC clinics?
62. *R: Most people are afraid of these methods and also place where these services are offered.*
63. I: So what do you think should be done to resolve these conflicts?
64. *R: Special places should be established where the family planning services should be offered and also place for circumcision should be separate.*
65. I: So now let us look at cancer screening for women. Please explain to me what happens when women comes to this clinic seeking for cervical cancer screening?
66. *R: When a woman comes for cervical cancer screening, they meet responsible doctors and they explain to them that they want to do screening for cervical cancer and they the doctors conduct the screening.*
67. I: In these circumcision clinics, do they screen for this cancer?
68. *R: No I have never seen that they do the screening.*
69. I: So you as health service provider support personnel, what’s your opinion about integrating cervical cancer screening within he VMMC clinics?
70. *R: These services should be integrated because they are similar, the reason that men go for circumcision is because they want to protect women from cervical cancer so the department of circumcision should have another service for cervical cancer screening.*
71. I: But what can make you not to want this integration to take place?
72. *R: There I should not lie, there is nothing that can make me not to want this integration.*
73. I: So what do you think are the best ways of providing the cervical cancer screening services in the voluntary male medical circumcision clinics?
74. *R: Men should be encouraging women to come screening while medical practitioners should be using proper equipment to screen out the presence of cancer cells.*
75. I: What about place, where should it be offered?
76. *R: It should be offered on a separate place not where the circumcision is taking place because cervical cancer is for women and circumcision is for men.*
77. I: So what do you think would be the barriers and concerns of integrating cervical cancer screening within the VMMC clinics?
78. *R: Men may not be willing to places like these that’s why men usually came alone at the clinics.*
79. I: How do you think these concerns should be resolved?
80. *R: I think en that have undergone the circumcision should be given counselling so that when they go back they can encourage their spouses to come and do the cervical cancer screening.*
81. I: Now let us talk about PrEP, have you ever heard about PrEP?
82. *R: Yes.*
83. I: What have you heard about PrEP, can you tell me what you know about PrEP?
84. *R: About PrEP, that time when I was in school some girl was raped so if the person that raped her was HIV infected, if this girl takes PrEP it will help not to let the virus multiply itself. But I haven’t heard much about PrEP but I know that it’s a drug that protects a person from contracting HIV.*
85. I: That one is PEP which post exposure when you have already been in contact while PrEP is Pre-Exposure Prophylaxis so I should explain to you about PrEP. This PrEP is a pill that is taken once a day and if taken according to the instructions there are high chances of an individual not Contracting HIV. I would give you an example, if I know the status of my wife that she is HIV positive and am negative but I want unprotected sex I would be taking PrEP regularly on daily basis without me contracting the virus. So having explained that in this way, how do you feel about PrEP?
86. *R: The way I feel about PrEP is that for a person that cannot be able to abstain from sexual intercourse, they can take the pill to protect themselves.*
87. I: Is it necessary to make PrEP available to men and women who don’t have HIV?
88. *R: Yes. It is supposed to be made available because we can’t be sure about their movements just to be safe if they have sex with an HIV positive person.*
89. I: So if PrEP is to be made available, what is your opinion about integrating PrEP within the VMMC clinics?
90. *R: My opinion is that this pill will help because when people have done circumcision they will no longer be using condoms so I think this integration should happen in the clinics to protect such people from contracting the virus.*
91. I: So you might be interested to encourage people to take PrEP?
92. *R: Yes, like a lot.*
93. I: So how do you think PrEP can be provided here at the VMMC clinic?
94. *R: Okay. PrEP should be made available in its own procedure to everyone who wants it whether you have done circumcision or not there has to be another department offering these services so that workload shouldn’t be too much.*
95. I: So should this service be offered outside or inside the same building?
96. *R: it should be done outside in a private room.*
97. I: But for people who have come for circumcision, where within the circumcision process should PrEP be offered?
98. *R: For those who have come for circumcision it will be up to their choice to receive the pill or not but it can be very important that everyone should be receiving PrEP if they have done circumcision.*
99. I: okay. What do you think are the concerns or barriers to this integration?
100. *R: People may not be interested to take the drugs because of pride.*
101. I: What do you think we can deal with this barriers?
102. *R: The best way is to give them guidance on the importance of this drug so that they should have the interest to take the drug.*
103. I: So now let us talk about other services. If you would be given chance to integrate other services into VMMC clinics, what other services would you integrate into the VMMC clinics?
104. *R: Most of the services we have already discussed here, PrEP and cervical cancer screening and family planning services but the major one should be this one for PrEP.*
105. I: Can you give me the reasons for your choices? Why PrEP?
106. *R: PrEP because when people have done circumcision they think that they are now protected from HIV and they go back have unprotected sex with women so PrEP will be very important to protect them.*
107. I: What about family planning service?
108. *R: Family planning, is good because if the husband comes for circumcision they get excited and when they go back they just have unprotected sex yet risking the probability of giving their wives unplanned pregnancies.*
109. I: And the last one, why cervical cancer screening?
110. *R: Cancer screening is very important because these things are related, cancer usually comes because of an uncircumcised man so if it is integrated it will be very helpful.*
111. I: So these three things you have mentioned; how do you think should be offered at this clinic?
112. *R: I think they should be offered on a separate place.*
113. I: Thank you for taking your time to talk to me today. Your answers will be very helpful in improving the health service delivery at the Voluntary Male Medical Circumcision clinics. Before we close the interview, do you have anything to say?
114. *R: Yes. What I can say is that this study in a clinic like this one, the things we have discussed should be made available and most of young men should be encouraged to come for circumcision.*
115. I: Okay. Thank you for taking your time to talk to me once again
116. *R: Thank you.*

*END*

**D 43 STUDY**

**Date of Interview: 03 July 2018**

**Type of Participant: Peer or Clinic Aid**

**Interview Number: D-43-0035**

**Interviewer: I. N.**

**Total Interview Time: 26 minutes 12 seconds**

**Interview Summary:** **(from summary sheet)**

| **SERVICE TO BE INTERGRATED** | **THOUGHTS ON INTERGRATION** |
| --- | --- |
| Couple HIV Testing and Counseling | A good opportunity for couples to test together. Partner can motivate you to get VMMC. |
| STI Services | Thinks STI services would help more men to get VMMC. Fears the VMMC clinic would be crowded |
| Family Planning | The integration would make it easier for men to have access to family planning since most of them do not know a lot about family planning. |
| Cervical Cancer Screening | Feels cervical cancer is a dangerous disease so integration is very important. |
| PrEP | A good idea as this integration would help to fight HIV better. Only concerned that people would not protect themselves. |
| Other Services | Thinks family Planning services and PrEP are the best for the integration. |

**Remarks:**

**Participant was relaxed and had a good sense of humor. He easily understood the questions and was very open-minded.**

**Interview Text:**

1. I: Thank you for taking the time to talk with me today.
2. *R: Mmm*
3. I: I would like to ask you some questions today about the way you feel and what you think about some issues related to the service you receive here and how we can include other services in Voluntary Medical Male Circumcision (VMMC) clinics.
4. *R: Alright.*
5. I: There is no right or wrong answers to these questions. We would like to hear your opinion and your experiences in your own words. Do you have any questions before we begin?
6. *R: No I don’t have any, but maybe at the end of this discussion.*
7. I: Alright. What role do you take in Voluntary Male Medical Circumcision services?
8. *R: I search for boys and young men in the communities who haven’t done VMMC so they can do it at the hospital.*
9. I: Can you give me an example of a day when you talked to a person about VMMC and how he responded?
10. *R: I tell them that when they do VMMC it helps reduce their risk of STI infections, for women it also protects against cervical cancer. So I ask the parents for their consent so that their young boys can get VMMC. When they consent I take them to the VMMC clinic.*
11. I: Do you ever talk to your friends about VMMC?
12. *R: Yes I do, a lot.*
13. I: Mm… okay, can you give me another example of an older person whom you were able to talk to about VMMC?
14. *R: I tell them that for a married person, VMMC helps to prevent cervical cancer because when you have VMMC your penis is clean.*
15. I: Lets now talk about couple testing and counseling. You come to this VMMC clinic right?
16. *R: Yes*
17. I: Okay so tell me what happens when a man brings his partner to the VMMC clinic?
18. *R: If a man comes with a partner he firstly goes to the reception and introduces the partner and the reason why they came. So the doctor there at the reception checks for eligibility and if you are eligible you are allowed to get VMMC.*
19. I: What is the role of the woman at the clinic?
20. *R: Her role is to take care of you.*
21. I: Don’t they test her as well?
22. *R: They test them both.*
23. I: They test her for HIV?
24. *R: Yes, and usually they also test the man.*
25. I: Have you ever seen partners getting couple testing at the VMMC clinic?
26. *R: Yes for several times.*
27. I: For those that bring their partners here what do you think motivates them to do so?
28. *R: It’s because it bonds them when they know their status together.*
29. I: Have you ever had couple testing and testing at the VMMC clinic?
30. *R: Yes, I did VMMC here at Bwaila clinic and I came with my wife.*
31. I: For those men that don’t bring their partners, what do you think makes them not to bring their partners to the VMMC clinic?
32. *R: It maybe because of negligence because we all think differently.*
33. I: What do you mean when you say “we all think differently”?
34. *R: Everyone has a right to choose whether he brings his partner or not so if one doesn’t want to bring his partner, you can’t force him to come for VMMC. Those of us that want to are the ones that come.*
35. I: What makes them not to want to?
36. *R: I don’t know what happens for them to act like that.*
37. I: Mm, what do you think should be done for men to bring their partners at the VMMC clinic?
38. *R: What can be done is that there should also be outreach clinics where they can be offering VMMC as well because some people don’t want to come here because of distance and transport costs. So the outreach clinics can help people not to spend money just to get VMMC.*
39. I: Right now the clinic is too far?
40. *R: Yes for some people to come here it costs them money. While if it’s close they can just walk.*
41. I: Alright. What is your opinion on integrating couple HIV counseling with Voluntary Medical Male circumcision services?
42. *R: I think it’s a good idea because both of you will know each other’s status and that strengthens the bond between the couples because there are no longer secrets. Once you know your status you can be able to plan your future.*
43. I: Mh. What do you think are the barriers and concerns on this integration?
44. *R: There cannot be any barriers.*
45. I: Mh. How about for men, don’t you think they can have any concerns?
46. *R: No they can’t have any concerns.*
47. I: I would like us to talk about sexual reproductive health services and Pills for HIV prevention: called pre-exposure prophylaxis…
48. *R: PrEP?*
49. I: Yes, PrEP Have you ever heard about it?
50. *R: Yes.*
51. I: Okay so we will talk about it in a moment.
52. I: (PrEP) Sexual reproductive health includes services that promote good sexual health and reproduction. They include but not limited to family planning, cervical cancer screening sexual transmitted infection management cervical, condom distribution and many more. Today we will only discuss family planning, Sexual transmitted infection management cervical screening and PrEP. We will look at each of these one by one. Let us start with STI services.
53. *R: Mm…*
54. I: Tell me what happens when a person is suspected or diagnosed with STIs?
55. *R: If a person is suspected or diagnosed with STIs and wants VMMC is not supposed to get it*
56. I: What happens when one is found like that?
57. *R: They don’t perform the surgery on him. Say for example he has Bubo and they perform the surgery. This person will be in a lot of pain due to the Bubo as well as the surgery. So they wait for him to heal from the infection and later do the surgery.*
58. I: As a peer, what is your opinion on integrating STI services with Voluntary Medical Male Circumcision services?
59. *R: Can you repeat the question*
60. I: At the VMMC clinic right now they only screen STIs for VMMC clients. So the question is as a peer, what is your opinion on integrating STI services with Voluntary Medical Male Circumcision services?
61. *R: I feel it can be good.*
62. I: Why is that?
63. *R: it’s because the providers will know before performing the surgery that a person has an infection and that will help them to prescribe treatment so that when the infection is dealt with he can come back for VMMC.*
64. I: How about for their partners and others who may want to screen for STIs?
65. *R: It’s a good idea because it will reduce the cases of STIs in the Malawi.*
66. I: Mm... What is it that you don’t like about this integration?
67. *R: There is nothing I don’t like about this integration because this will help to reduce infections in this country.*
68. I: So how do you think is the best way to offer STI services at the Voluntary Medical Male Circumcision clinics? At what time point and where should the service be offered?
69. *R: I think the best way is by building VMMC clinics closer to the people.*
70. I: They should build VMMC clinics?
71. *R: Yes*
72. I: What then should be happening at those clinics?
73. *R: They should be providing VMMC, family planning, STI services and so on.*
74. I: Okay. So you know the clinic flow at the VMMC clinic. Like you said if a man comes he firstly goes to the reception…and so on. So in that flow when should screening for STIs be done so that things go smoothly at the clinic?
75. *R: I feel like in the morning that’s the best time they can do the STI screening then at around 9 or 10 they can start the VMMC procedures.*
76. I: That means they should not provide these two services at the same time?
77. *R: No they should not provide both at once because that will be too much work for them if they are t screen for STIs and do the surgeries at the same time.*
78. I: Mm, so they should do the screening in the morning?
79. *R: Yes.*
80. I: Who should be screened for STIs at the VMMC clinics?
81. *R: It should be men…and women as well because they may be both infected*
82. I: What other concerns and barriers do you think are there for this integration??
83. *R: Like what can make men fail to come or not?*
84. I: Yes, and what barriers can be for this integration to happen?
85. *R: There cannot be any barriers.*
86. I: There can be no barriers?
87. *R: No.*
88. I: As a peer you know the people you reach out to…
89. *R: Yes, I know the men I reach them. It happens that when you talk to someone about VMMC he gives an excuse that he is busy with work and we leave them. The next time we reach them they may promise to come another day and yet they don’t come. But if the clinic was close to their homes they would have made it.*
90. I: Okay. Now I want us to talk about family planning. Tell me what happens when a person comes to the VMMC clinic to access family planning?
91. *R: If a person comes to the clinic seeking family planning…like any method?*
92. I: Yes. Say they have come with a partner who wants family planning or it’s the man who wants a method.
93. *R: let me talk about the woman. When she comes for family planning she goes to the family planning clinic where she meets a nurse and is given information about the different family planning methods like pills, depo, Norplant and so on. So she chooses what type of method she wants without being forced.*
94. I: Mh. Does that happen at the VMMC clinic or other clinics?
95. *R: This happens at any government hospital.*
96. I: Okay, so my question was specifically asking from VMMC clinics. Say a person comes to the VMMC clinic seeking family planning, what happens?
97. *R: I have never heard of that.*
98. I: And you have never had such cases.
99. *R: No I have never met someone seeking family planning at the VMMC clinic.*
100. I: As a peer what is your opinion on integrating Family planning in Voluntary Medical Male circumcision services/clinic?
101. *R: I think that will work to the advantage of couples because it’s mostly men that come to the VMMC clinic, now if they integrate with family planning it means women will also have a chance of coming which will make marriages stronger.*
102. I: Mh, why do you say so?
103. *R: I am saying so because if they integrate with family planning it means you can tell your partner to escort you so that she accesses family planning as you get VMMC. And that will work.*
104. I: What is it that you would not like about integrating Family Planning services in VMMC?
105. *R: What about this?*
106. I: Like what you would not like about this integration.
107. *R: The problem here can be since women will also be coming to the clinic, some may feel embarrassed seeing circumcised men.*
108. I: Mh.
109. *R: So that can be a problem.*
110. I: Okay. What do you think is the best way to provide the family planning services at the VMMC clinic?
111. *R: I feel they should have a special room where they can be offering family planning would be better.*
112. I: Mh. What time should that be?
113. *R: I think at around 9 can be the best time.*
114. I: Okay say they have come together as a couple, at what point during the VMMC process should the woman get family planning?
115. *R: Say they have come at 10, then the nurse needs to prioritize this couple so that they are helped faster and get all the services.*
116. I: Okay. What do you think could be the barriers or concerns on family planning and Voluntary Medical Male circumcision integration?
117. *R: I don’t think there can be any concerns. Actually I feel this is a good thing because it will now become easier for men to negotiate with their wives to escort them. They will use family planning as an excuse for them to go together.*
118. I: Okay. Now let’s talk about cervical cancer screening. Tell me what happens when someone wants to get cervical cancer screening?
119. *R: If she comes to the VMMC clinic?*
120. I: Yes say she comes to the VMMC clinic seeking cervical cancer screening.
121. *R: When she comes she goes to the reception where she is told what to do like consulting with the nurse then the screening.*
122. I: Okay. Do they screen for cervical cancer at the VMMC clinic?
123. *R: No they don’t do it at the VMMC clinic.*
124. I: Now I was asking if the woman comes to the VMMC clinic.
125. *R: No I have never been faced with such a situation.*
126. I: As a peer, what is your opinion on integrating partner cervical cancer screening with Voluntary Medical Male circumcision services
127. *R: I feel like it’s very important because when a man comes with a woman who wants cervical cancer screening, he can be going into surgery while the woman gets screened. So I think it’s a good idea.*
128. I: Why do you say so?
129. *R: It’s because I do VMMC so that my wife can have good health. So if I bring her here and she also gets help then that is a good thing.*
130. I: Do you feel that there is a connection between VMMC and cervical cancer screening?
131. *R: Yes, there is a connection. If a man has done VMMC and has sex with a woman, there is no way that woman can have cervical cancer.*
132. I: What is it that you would not like about this integration?
133. *R: Some men cannot feel comfortable to bring their partners.*
134. I: Why do you say so?
135. *R: As men we talk about such issues. Some men can mock you to say “They did so and so to his wife!” And that can be humiliating.*
136. I: Mh, so how can we deal with such a problem?
137. *R: The men should have their own room where the surgeries are performed and women should have their own as well.*
138. I: Mh, they should not be screened for cervical cancer in the presence of their partners.
139. *R: Like a man there, another man there and a woman being screened there…? [Laughing]No! Not like that. Maybe if we say one should go to one room and the other in another room then that’s possible. You know at the hospital we meet people from different backgrounds so if these people are to be in the same room with your wife. It would be embarrassing.*
140. I: Okay. So when is the best time to offer this service?
141. *R: The right time is after 12 pm.*
142. I: Why have you chosen that time?
143. *R: It’s because the providers can be providing the other services in the morning then rest and start this screening.*
144. I: Like they should rest…?
145. *R: Yes, because they start working in the morning so in the afternoon they can rest and start the screening for cervical cancer.*
146. I: Alright. So who do you think should be screened for cervical cancer at the VMMC clinic?
147. *R: It should be nurses.*
148. I: I mean the women that come for the service.
149. *R: It should be everyone that comes for the service because this disease can attack any woman whether she comes with her husband or not.*
150. I: Why do you say so?
151. *R: It’s because every woman in the productive age needs to be screened for cervical cancer.*
152. I: What concerns or barriers can there be for this integration?
153. *R: Barriers like…*
154. I: Barriers that can prevent this from working.
155. *R: The barriers can be ideas from people. Some people may try to give suggestions on how this should be done.*
156. I: What kind of suggestions?
157. *R: Like some people may say” You should have let VMMC be and build another clinic for the cervical cancer screening”. Others may say “It would have been better if they partitioned the building so that VMMC is on one side and the other side cervical cancer screening”.*
158. I: Why would some people say that?
159. *R: It’s because they may think the VMMC clinic is very far away from their homes.*
160. I: How can we deal with such concerns?
161. *R: We need to sensitize the people and give them enough information. This will make work easier.*
162. I: Okay. Now let us talk about PrEP. Have you ever heard about PrEP?
163. *R: Yes, I know PrEP very well. It’s a drug that you take if you know that you had unprotected sex with a woman. So PrEP helps you not to be infected.*
164. I: Oh okay. So there are two different drugs, PEP and PrEP. PrEP is an anti-HIV drug which helps to keep HIV-negative people negative. There is a pill that one needs to take every day to prevent HIV infection. If taken regularly it can help to prevent HIV infection. While with PEP you just take the pills only when you feel like you have been exposed to HIV. For PrEP you take the pills daily regardless of exposure
165. *R: Oh okay.*
166. I: So how do you feel about PrEP?
167. *R: It’s a very good drug.*
168. I: Why do you say so?
169. *R: If one is HIV negative and takes PrEP he will be at ease rather than a person who is not taking PrEP who will always be one the look out not to be exposed to HIV*
170. I: [laughing] Now do you think it’s necessary for PrEP to be made available to HIV-negative men and women?
171. *R: Yes, I feel it’s necessary, even though I said I don’t like it. I say it’s necessary because this generation is a weird one where people love sex. So it’s necessary for such people to be protected and be given these pills.*
172. I: Would you encourage someone to be taking the pills?
173. *R: Yes, I can encourage them.*
174. I: What reasons would you have to encourage someone to take them pills?
175. *R: It’s because they are good drugs which can protect someone if he takes them*
176. I: Mh. If PrEP becomes available, what is your opinion on integrating PrEP with Voluntary Medical Male circumcision services?
177. *R: So that people can be getting PrEP at the VMMC clinic?*
178. I: Yes
179. *R: It’s good because that means after getting VMMC they will be giving you PrEP to take the pills everyday so that you don’t get infected.*
180. I: Mh. How do you think PrEP would be offered in this clinic?
181. *R: It can be offered to everyone starting from 8 am so that people can be going back to work.*
182. I: Mh. Not that they should only be offering to VMMC clients?
183. *R: No, that would be segregation.*
184. I: Everyone should be able to get PrEP…
185. *R: Yes, and when you do that you will be attracting more people to get VMMC*
186. I: What would be your concern if people are taking PrEP?
187. *R: I don’t have any concern because if the drugs are available and someone comes to the clinic, gets the pills then goes home to do his daily work, that is a good thing*
188. I: Mh…
189. *R: Sure*
190. I: If you were given powers to choose and integrate services in Voluntary Medical Male Clinics, what are the services that you would think of Integrating?
191. *R: I can opt for cervical cancer screening and PrEP.*
192. I: Mh. Why have you chosen cervical cancer?
193. *R: It’s because many women in the communities are fighting this disease so if it is integrated in the VMMC clinic then many women will come for screening. I also like PrEP because most of us will have a chance to get these pills.*
194. I: Okay, thank you very much for your time. Your answers will be very helpful in improving the health service delivery at circumcision clinics.
195. *R: Mh… thank you.*
196. I: Before we close, is there anything more you would like to say?
197. *R: I thank you because I have learnt some new things here. I will make sure that in a few days to come I should bring other men to get VMMC.*
198. .I: Okay. Thank you very much for talking to me today.
199. *R: Thank you.*

END

**D43 STUDY**

**Date of Interview: 21 August 2018**

**Type of Participant: Peer and clinic aides**

**Interview Number: D-43-0038**

**Interviewer: I. N.**

**Total Interview Time: 37 minutes** 47 **seconds**

**Interview Summary:** **(from summary sheet)**

| **SERVICE TO BE INTERGRATED** | **THOUGHTS ON INTERGRATION** |
| --- | --- |
| Couple HIV Testing and Counseling | Thinks it’s good because everyone will be able to know their partner’s status |
| STI Services | Thinks it’s good because some of the youths that are infected with STIs will be able to be circumcised and have a brighter future just like everyone else |
| Family Planning | Thinks it’s not right because family planning has its own wider services that can allow it to function as an independent service. |
| Cervical Cancer Screening | Thinks it is a nice and good idea because in this case the husband will be protected while at the same time the woman will not be at risk of suffering from cervical cancer. |
| PrEP | It is necessary so that people should be protected |
| Other Services | None |

**Remarks:** Participant was shy and felt uncomfortable. He was finding it difficult to understand the questions and even provided short answers.

**Interview Texts:**

1. I: Thank you so much for sparing your time to talk to me today. I want to ask you a few question on the services you provide and your opinions on any additional services onto the services that is provided in Voluntary Male Medical Circumcision clinics. There is no right or wrong answers to these questions. We want to hear your understanding and opinions. Do you have any questions before we begin the interview?
2. *R: No. I don’t have any question.*
3. I: Can you please tell me the role you play here at VMMC clinic?
4. *R: I encourage people to go for circumcision.*
5. Interviewer: are your friends able to talk you concerning the services that are offered here at VMMC clinic?
6. *R: Yes, they are able to do so.*
7. I: would you please give me an example of the time when your friends ever talked to you about the services they got at the VMMC clinic? I mean after undergoing the circumcision procedure here…you tell me what they said about the service they got.
8. *R: Okay, they were given Sobo squash, under wears and a drink.*
9. I: But based on the circumcision what did they tell you. about any changes they experienced after the circumcision service? Did they say anything?
10. *R: Yes. They were given counselling that when you have done circumcision you have 60%.*
11. I: 60% of what?
12. *R: Of preventing HIV virus*.
13. I: Okay fine. Now let us talk about going for an HIV test as a couple. This is one of the services that they are planning to integrate here at the VMMC clinic. So that couples would be able to come together and do the HIV test as a family. Explain to me, what happens when a husband brings his wife here at VMMC clinic?
14. *R: May you please come again?*
15. I: Okay maybe I should ask in this way, have you ever seen a man that came with his wife here at VMMC clinic?
16. *R: Yes. I have ever seen.*
17. I: So what happens when the husband brings his spouses at this clinic? You have said you have ever seen this happening here. Tell me what happened.
18. *R: They just escorted each other. The woman escorted the husband for circumcision.*
19. I: Was the woman going together with the husband in the different rooms that people go through in the circumcision process?
20. *R: No the woman was just outside.*
21. I: What do you think motivates men to bring their spouses to undergo an HIV test together?
22. *R: It is because of the trust that exists in the relationship.*
23. I: And what do you think makes men not to bring their spouses to do an HIV test together?
24. *R: They are afraid that if the test comes out positive for him, that will be the end of their marriage.*
25. I: Okay…hmm so what do you think should be done so that men should be bringing their spouses for an HIV test together?
26. *R: I think we should just be encouraging them.*
27. I: How can we encourage them?
28. *R: By telling them the importance of doing the HIV test together as a family maybe through radios.*
29. I: You as a service provider, what do you think about the integration of HIV couple testing on to the already available service of VMMC? We understand in the VMMC clinics they just provide circumcision service, now there is this new idea of integrating HIV couple service onto this already existing service, so what’s your opinion on this idea?
30. *R: I think that can be good.*
31. 31. I: Why do you think that can be a good idea?
32. *R: Because everyone will be able to know their partner’s status.*
33. I: What do you think would be the barriers to this integration?
34. *R: May you repeat the question?*
35. I: If they are to integrate HIV couple testing at VMMC clinic, what do you think would be the concerns or barriers?
36. *R: They may not be not being ready to hear the results together, especially the husband may not really be comfortable.*
37. I: What are other concerns?
38. *R: They may be worried to think that if they go together for the test at the clinic it will lead to the divorce of their marriage.*
39. 39. I: To the barriers you have mentioned, what do you think can be done to resolve them? You have said that the husband is afraid, divorce, so to resolve these fairs what do you think can be done?
40. *40. R: Just encouraging them to remain trust-worthy to each as a couple.*
41. 41. I: Now I want us to discuss sexual and reproductive health and pills that protect from HIV which are known as PrEP. Sexual and reproductive health includes among others, family planning, cancer screening, condom distribution, STIs management etc. Today we will only discuss issues around family planning, cancer screening, STIs management as well as PrEP We will discuss each one of these issues in detail. So let us start with the issue of sexually transmitted infections. Please explain to me what happens when a person is suspected or has be diagnosed with STIs here at the VMMC clinic?
42. *. R: When a person is suspected or has been diagnosed with STIs he becomes stressed over these results so it just depends on this person to accept his results.*
43. I: No I want to understand what happens to the circumcision procedure and everything here?
44. *. R: it all depends on the person; some they just accept that it has happened but some do not understand.*
45. . I: But here at the clinic after the tests…what happens?
46. *. R: You get you test results and know your health status.*
47. . I: Or should ask in this way, explain to me the procedure for circumcision when one arrives at the clinic…what procedures are taken?
48. *. R: You first receive counselling then they test your blood for HIV and then they ask you if you are ready to be circumcised then you get circumcised*.
49. . I: After the circumcision, do you immediately leave for home or you are told to go into another room?
50. *. R: You just go home.*
51. . I: Have you done circumcision yourself?
52. *R: Yes.*
53. I: When was that?
54. *. R: In 2015*
55. . I: So have you already forgotten?
56. *R: I did not forget but you know.*
57. I: Because this survey is supposed to be done with those that are aware of the issue. So are you really circumcised? If yes, then let’s proceed with the questions. When they have done the blood tests and you have been diagnosed with an STI, do they proceed to do the circumcision or they don’t?
58. *. R: It depends on the kind of STI you have been diagnosed with. If you have been diagnosed with infections like HIV they proceed to do the circumcision but if they diagnose your gonorrhea or syphilis they don’t conduct the circumcision on you.*
59. . I: So you as a service provider, what do you think about this integration of STI management here at VMMC clinic?
60. *. R: It’s good because some of the youths that are infected with STIs will be able to be circumcised and have a brighter future just like everyone else.*
61. . I: But what is it you don’t agree to about this integration?
62. *. R: Ah nothing.*
63. . I: What do you think are the best ways of delivering these services here at the VMMC clinic? Within the circumcision process, where should this integration be done?
64. *. R: That is based on those that have already been circumcised?*
65. . I: A person has come for circumcision, you have explained the procedure that is followed during the circumcision process… so let’s assume this integration has happened, where within the VMMC should the STI management be undertaken, and should it be done before, within or after the circumcision?
66. *. R: I think before circumcision because of certain other infections that does not allow the circumcision to proceed.*
67. . I: So what place would be ideal, should it be done within this building or outside or another private room?
68. *. R: It should be offered within this very same building.*
69. . I: What would be the concerns or barriers to this integration?
70. *. R: There cannot be barriers by I think it would consume more time to undergo through the whole circumcision process and this one as well.*
71. . I: When you are coming for circumcision, don’t they test you for STIs?
72. *. R: They test.*
73. . I: So what do you think that concern of time can be resolved?
74. *. R: Maybe the procedures should be done, the service providers should be increased so that the work should be done faster.*
75. . I: Now let us talk about family planning. Have you ever heard about family planning?
76. *. R: Yes.*
77. . I: What have you heard about family planning?
78. *. R: I heard they are drugs that a person takes to protect yourselves from pregnancy.*
79. . I: Do men practice family planning?
80. *. R: I didn’t understand clearly on that one so I can’t really say.*
81. . I: Men also do practice family planning. What family planning methods do you know?
82. *. R: Nor-plant only.*
83. . I: There are condoms, injection, pills etc. so would you please explain to me what happens if a person is seeking a family planning services at the VMMC clinic?
84. *. R: I don’t know.*
85. . I: You as a health service provide support staff, what do you think about the idea of integrating family planning services within the VMMC services?
86. *. R: I think it is not right to be so.*
87. . I: Why is that so?
88. *. R: Because family planning has its own wider services that can allow it to function as an independent service.*
89. . I: Is there anything you may like if it is to be integrated within the VMMC? Or I should ask in this way, what do you think are the best methods of delivering family planning methods in the VMMC clinics?
90. *. R. Mm…. I don’t know.*
91. . I. What do you think are the best ways of delivering family planning services in the VMMC clinics regarding place and time?
92. *. R. Anytime is okay to receive these services.*
93. . I. What about the place? Should it be inside, outside or in a private room?
94. *. R. It’s supposed to be inside. In a private room.*
95. . I. Do you think that there are any barriers or concerns on the integration of family planning and VMMC?
96. . R. *I don’t know on that one.*
97. . I. So now let us look at cancer screening for women. Please explain to me what happens when women come to this clinic seeking for cervical cancer screening?
98. . R. *When a woman has been diagnosed with cervical cancer screening?*
99. I. Explain to me what happens when a woman wants to do cervical cancer screening?
100. *R. Mm… I don’t know.*
101. I. But do you know cervical cancer?
102. *. R. No. I don’t know it*
103. . I. It’s a type of cancer that attacks women and it usually affects the cervix. When you came for circumcision, didn’t they explain to you how the cervical cancer begins?
104. *R: They explained but then it’s been long so I forgot.*
105. I: This cancer happens because of a man’s foreskin that keeps substances that are causatives of cervical cancer in women. In other words, uncircumcised men have the ability to pass this type of cancer to their loved ones. So you as a service provider, what do you think about integrating cervical cancer screening within the VMMC clinics?
106. *R: I think it is a nice idea because in this case the husband will be protected while at the same time the woman will not be at risk of suffering from cervical cancer. So I think it will be good.*
107. I: What is it you don’t like about this integration?
108. . *R: aaaahah nothing.*
109. I: What do you think are the best ways of delivering this service in the VMMC clinics in regard to place and time?
110. *. R: Mm… ah…. repeat the question I didn’t get it.*
111. . I: What do you think are the best ways of delivering the cancer screening service here at the clinic in regard to place and time, should it be done within this very same building, outside or in a separate room?
112. *. R: I think it should be done in a separate room.*
113. I: Why do you think it should be done in a private room?
114. *R: Because cervical cancer affects a private part of a woman so I think the screening should as well be done in a private room.*
115. I: The time within the circumcision procedure, what time should cancer screening within the circumcision process?
116. *R: I think during the evening.*
117. I: Why during evening/late hours?
118. *R: I think we should not say the evening I think any time since we are saying we can be doing it in a private room.*
119. I: What do you think can be the barriers of integrating the cervical cancer service within the VMMC clinics?
120. *R: Ah there are no barriers.*
121. I: Now let us talk about PrEP, have you ever heard about PrEP?
122. *R: Yes.*
123. I: Explain to me what you have ever heard about PrEP?
124. *. R: These are drugs that you take every day and they help to protect one from HIV.*
125. I: Ah…. how did you get to know the information about PrEP?
126. *R: I heard from a friend because he takes these drugs every day, one pill per day.*
127. I: So these drugs are available?
128. *R: Yes*
129. I: How does he access them?
130. *R: Ah I shouldn’t lie, I don’t know.*
131. I: So what is his intentions for taking these drugs every day?
132. *R: When he has sex with an HIV positive person, he does not contract the virus because of these drugs.*
133. I: Alright. Is it necessary to make PrEP available to men and women who don’t have HIV?
134. *R: Yes. It is necessary.*
135. I: Would you please tell me the reasons you would encourage people to take PrEP?
136. *R: So that they should be protected.*
137. I: If PrEP is to be available, what is your opinion on integrating PrEP within the VMMC clinic?
138. *R: May you repeat the question again?*
139. I: If PrEP is to be available, what is your opinion on integrating PrEP within the VMMC clinic?
140. *R: It will help a lot of people to be protected from the HIV virus that causes AIDS.*
141. I: So you are happy with this integration that you may even be interested to tell people about it?
142. *R: Yes.*
143. I: How do you think PrEP should be administered here at the VMMC clinic?
144. *R: It should be given to anyone who is interested at any time of their choice.*
145. I: What barriers do you think exists in integrating PrEP within the VMMC clinics?
146. *R: There are no barriers to this integration.*
147. I: Okay. Now let us talk about other services, if you have been given authority to add any other services to the VMMC clinics, what services would you add?
148. *R: You mean from the services you have explained?*
149. I: yes.
150. *R: I would choose the PrEP service.*
151. I: What other service would you choose?
152. *R: Cervical cancer screening.*
153. I: Is there any other service?
154. *R: No. only these two.*
155. I: Why have you chosen PrEP provision and cervical cancer screening?
156. *R: PrEP is good because it will help people to stay protected from HIV and AIDS on the other hand cancer screening is good because when the husband is circumcised he cannot give his loved one cervical cancer.*
157. I: How do you think these two services you have chosen should be offered here at the VMMC clinic? Like PrEP.
158. *R: Like I have already said, they can be offered anytime.*
159. I: What about the cervical cancer screening service?
160. *R: I think it’s just the same. They can be offered any time.*
161. I: Thank you so much for giving me your time today to discuss these issues regarding Voluntary Male Medical Circumcision clinics, your answers will help to improve the services that are offered within the VMMC clinics. Before we conclude the interview, do you have anything to say concerning the issues we have discussed today?
162. *R: No I don’t have anything to say.*
163. I: Thank you so much.
164. END.

**Peers and Clinic Aides interview guide**

**D 43 STUDY**

**Date of Interview: 24 August 2018**

**Type of Participant: Peer and clinic aides**

**Interview Number: D-43-0043**

**Interviewer: I.N.**

**Total Interview Time: 42 minutes 14 seconds**

**Interview Summary:** **(from summary sheet)**

| **SERVICE TO BE INTERGRATED** | **THOUGHTS ON INTERGRATION** |
| --- | --- |
| Couple HIV Testing and Counseling | Thinks its good development because it helps a person, the way he is living to know how they are and expect good things |
| STI Services | Thinks it’s a good idea because this helps a person who hasn’t been circumcised to see how he is so when he is found with a problem they can be able to help him out |
| Family Planning | Thinks it is a good idea because the two things are related and helps a person’s wellbeing |
| Cervical Cancer Screening | Thinks it’s a not good idea because it something that is hard at first since target is about circumcision so with cervical cancer screening there can be a separation, so women are not supposed to receive this help of cervical cancer testing at VMMC clinics and women are another part not receiving circumcision, so it doesn’t really concern them so it’s hard, and impossible |
| PrEP | Like the idea of integrating PrEP with VMMC services because these drugs are helpful because when one gets circumcised they expect that man to be protected, so when he receives one part of helping his health, he needs to get the other service that can help him not to get infected. |
| Other Services | Thinks family planning and HIV testing as a family and bringing of women for cervical cancer screening should be integrated |

**Remarks:** Participant was open, confident and provided as much information as possible.

**Interview Text:**

1. Thank you for taking the time to talk with me today. I would like to ask you some questions today about the way you feel and what you think about some issues related to the service you provide and how we can include other services in Voluntary Medical Male Circumcision (VMMC) clinics. There are no right or wrong answers to these questions. We would like to hear your opinion and your experiences in your own words. Do you have any questions before we begin?
2. *R: No.*
3. I: Can you tell me how you are involved in the client care at this clinic?
4. *R: I take part in searching and encouraging the youth to take part in VMMC*
5. I: How do you find them?
6. *R: We go deep inside the village and explain the goodness of circumcision*
7. I: Does your peers talk to you about how the services are provided here?
8. *R: My friends who I work with or the one I chat with?*
9. I: The ones you bring for circumcision?
10. *R: Not all of them, some of them talk about it*
11. I: Can you give me an example of a time that your Peer talked to you about the services he received here?
12. *R: For example, what he told me was that he was a witness that circumcision is a good thing and it helps a lot*
13. I: In what way?
14. *R: The way we were talking he was saying it helps at the time he is sleeping with a woman there is good protection and he can prevent diseases*
15. I: Now let us talk about partner HIV testing here at the Voluntary Medical Male circumcision clinic. Tell me what happens if a man brings a spouse here at the Voluntary Medical Male circumcision clinic?
16. *R: What happens to men?*
17. I: What happens when a man brings his spouse here for example when they come with the aim that the man will get circumcised, what happens?
18. *R: To the man?*
19. I: To both of them?
20. *R: I didn’t understand the question, the big thing is that the man has to get tested because he is the one who will get circumcised, for being a family and that they have come together, they have to know their health status*
21. I: Have you ever seen a man bringing his wife when he is coming for VMC and HIV testing?
22. *R: Yes, I have seen them and I know one of the couples personally*
23. I: What do you think are the motivators that make the men bring their spouses here for testing?
24. *R: There is a good expectation that they should know how their status are so that they should be able to depend on each for the rest of the marriage and know how to handle themselves*
25. I: What do you think demotivates men to bring their partners here for HIV counselling and testing?
26. *R: Most of the men are scared of being found positive, mostly it’s because of being doubtful about one self because of how they have been conducting themselves, so they fear that if they bring their wife for testing it will not end well for them.*
27. I: What do you think can be done to make men bring their partners here for couple testing and counselling?
28. *R: This issue is the one we are encouraging when we go in the villages it just need to encourage and motivate them and tell them how the world is operating and how they ae supposed to live their lives, getting HIV tested and its benefit*
29. I: You as a peer, what is your opinion on integrating couple counseling with Voluntary Medical Male circumcision services.
30. *R: I think getting tested for HIV as a family and other help we give to other people who come for VMMC, all the sides are important but I should say circumcision is being encouraged also for young men so that they should be aware of what is happening in the world when it comes to the family it means that we are also motivating them and encouraging them to come to the hospital when the man I coming for VMMC they should also get tested to know their health status*
31. I: You think it’s a good thing?
32. *R: Definitely it’s a good thing*
33. I: Why do you think it’s a good thing?
34. *R: I already said it a good thing that it helps a person the way you are living to know how you are and you expect good things when you know yourself*
35. I: What do you think are the barriers and concerns on this integration?
36. *R: Barriers can be maybe because of the way some people talk and how your spouse is talking, but there is a good thing that can be explained in different way, but the barriers can come in because of those partners that give us wrong interpretations*
37. I: What do you think can be done to overcome these concerns and barriers to couple counseling in Voluntary Medical Male circumcision services clinic?
38. *R: Because of counselling and teachings we give will motivate them not to have worries when people don’t know anything they have fear, they don’t know what to expect, but when you give them good information and they are confident nothing can stop them*
39. I: Now I would like to discuss with you about sexual reproductive health services and Pills HIV prevention: called pre-exposure prophylaxis. (PrEP). Sexual reproductive health includes services that promote good sexual health and reproduction. They include but not limited to family planning, sexual transmitted infection management, cervical cancer screening, Condom distribution and many more. Today we will only discuss about family planning, diagnosis and management of STIs, Cervical cancer screening, and PrEP. We will look at each of these one by one. Let us start with STI services. Explain to me what happens if a client is suspected or diagnosed with an STI here?
40. *R: It is an important information to those who are found positive*
41. I: Do they will circumcise him?
42. *R: There is enough privacy, we give advice which is very important and we don’t tell other people, we just tell him how he is supposed to live his life but we don’t tell other people*
43. I: But how is the person helped?
44. *R: He is helped by giving him other medicine that can help him in hi everyday life*
45. I: Let’s say the person has candidiasis
46. *R: When he is infected with these diseases, he is supposed to get help so that he can be safe and get better*
47. I: If the person is feeling better, doe he come for circumcision?
48. *R: When he is okay he is supposed to come back and get circumcised*
49. I: You as a peer what is your opinion on integrating STI services with circumcision services.
50. *R: This helps because it need a person who hasn’t been circumcised to see how he is so when he is found with a problem we can be able to help him out, if he has any disease, we help him up to the point that he feels better and he is ready to get circumcised*
51. I: What is it that you would not like the integration of diagnosis and management STIs with Voluntary Medical Male Circumcision services?
52. *R: When screening, because there are different thoughts by different people from different places, so sometimes some people are not treated alright, so there is need to see it through that somehow somewhere there is a problem*
53. I: How do you think STI services should be offered at the Voluntary Medical Male circumcision clinic?
54. *R: As already explained, he should get tested, if he is found with a problem, after he is treated and he feels better he can come for circumcision*
55. I: What do you think are the barriers and concerns on this integration
56. *R: mostly people are afraid, some people are born with fear so they get motivated when we counsel them there is a huge concern to those who are not able to get the information well when we are teaching them about HIV and circumcision, or they wish they could just one part when they think of getting circumcised and HIV testing, they would rather get one*
57. I: What do you think should be done to address these concerns and barriers?
58. *R: We should encourage them and we should advise them in a way that the people should truly understand about the services*
59. I: What about place, how is the service supposed to be given?
60. *R: This kind of help, it needs to be done at the hospital, in a private place*
61. I: Same hospital for VMC?
62. *R: Yes, it should be in the same room where you get VMC and STI*
63. Let us talk about family planning. Explain to me what happens if a client needs a family planning methods (vasectomy for men and family planning for female partners?
64. *R: Family planning is giving yourself space in giving birth, we help them in such a way that at first we see them, especially men if they want vasectomy, we explain to them about vasectomy, but the main way we follow is that we explain about family planning and we encourage them to get tested, since we look at circumcision we just receive them and just explain about family planning*
65. I: How do they provide this at VMMC?
66. *R: There is family planning that involves pills as well as injection*
67. I: That means they are giving it at VMMC?
68. *R: I did not understand well, but at VMMC the family planning method we give them is that they should be protecting themselves and not pills and injections, we just give them the way of protecting themselves like condoms*
69. I: As a peer, what is your opinion on integrating Family planning in circumcision services?
70. *R: It helps a lot because as you are providing VMMC and family planning, these are two things that are related that helps a person’s wellbeing hence its good cause combing the two work together*
71. I: What is it that you would not like about the integration of family planning with Voluntary Medical Male Circumcision services?
72. *R: Let me just say integrating VMMC and family planning for men it’s something that when we give men circumcision it’s also good to encourage them in family planning which I see somehow they are not supposed to take part in it*
73. I: How do you think family planning services would be offered within Voluntary Medical Male circumcision clinics?
74. *R: It’s the only way to prevent, especially condom*
75. I: Since family planning is not only about condoms we have nor plant, pills, injection, vasectomy and others , so when all these are integrated with *VMMC* , where should they be offering them same clinic , in a private room outside and the person who has come for *VMMC* when should he get the service of family planning
76. *R: The one who has gotten circumcised is supposed to get family planning method when he has been certified to be okay and receive circumcision*
77. I: What about place?
78. *R: It should be in the clinic*
79. I: What do you think are the barriers and concerns with the integration family planning and Voluntary Medical Male circumcision services?
80. *R: It can be because of how we have given space to the one who has received circumcision, it can happen that he can make other decision, maybe we were thinking of helping him in other ways, so some would not come back for those services*
81. I: What do you think should be done to address these concerns and barriers?
82. *R: There is need to be following them so that they should not be changing their minds so that we help them in every aspect*
83. I: Let us talk about cervical cancer screening. Explain to me what happens if a woman needs cervical cancer screening?
84. *R: What happens when a woman wants to get screened in cervical cancer they are supposed to meet those at the hospital, so what happens I, can you ask the question again*
85. I: Have you ever seen a woman coming here for cervical cancer screening?
86. *R: Yes, I have, but I don’t know what happens from there*
87. *I: So I was saying, what happens when a woman wants to get tested for cervical cancer here at a VMMC clinic*
88. *R: What happens?*
89. I: Or how does it work?
90. *R: When the woman gets to a VMMC clinic for cervical cancer screening, they start with testing*
91. I: They provide this service of cervical cancer testing?
92. *R: Some of these questions are confusing*
93. I: At a VMMC clinic?
94. *R: We don’t give them any help to women who want to get cervical cancer screening*
95. I: As a peer, what is your opinion on integrating cervical cancer screening for female partners with Voluntary Medical Male Circumcision services?
96. *R: It’s something that is hard at first because target we have is about circumcision so cervical cancer screening there can be a separation that can happen, women are not supposed to receive this help of cervical cancer testing here*
97. I: Why?
98. *R: Women are another part that are not receiving circumcision, it doesn’t really concern them so I think it’s hard, and it’s impossible*
99. I: What is it that you would like about partner’s cervical cancer screening integration with Voluntary Medical Male Circumcision services?
100. *R: If they happen to integrate o these issues, the help can be given depending on how it’s needed at that time*
101. I: In terms of place how should they be offered?
102. *R: I should be at the same clinic but they should divide the place where women can get help and the place where men can get circumcised*
103. I: When they come as a couple how do you think it can work, when the man has come for circumcision and the woman has come for cervical cancer screening?
104. *R: It means that we will divide them, divide the time, the man can get circumcised on his own and the woman get cancer screening on the other side but the same clinic*
105. *I: What is it that you would like about the integration of partner cervical cancer screening with* Voluntary Medical Male *Circumcision services?*
106. *R: Right now, I can say that it is a good idea but I already say that people receive things differently but now I like it*
107. I: What is it that you like?
108. *R: It good because right now it’s good to trust each other as a family, so coming together to get these services is good for the family*
109. I: What do you think are the barriers and concerns about integrating partner cervical cancer Screening and Voluntary Medical Male Circumcision services?
110. *R: There are some concerns because the woman can have their own thought that contradicts the man, so the concern is that when these people don’t agree to come together there can be some barrier*
111. I: What do you think should be done to address these concerns and barriers?
112. *R: it can be dealt with in the sense that those who have come here for services get to encourage other people to come and do the same*
113. I: Now let us discuss about PrEP. Have you heard about this before?
114. *R: prep?*
115. I: yes
116. *R: I have heard about prep before*
117. I: If you have heard about PrEP, please tell me what you know about PrEP?
118. *R: I heard that these drugs that are given to women who has been found with cervical cancer*
119. I: How did you learn about this?
120. *R: I heard about this from a friend who takes part in teaching about it*
121. I: We can say that’s another PrEP, but the PrEP am talking about is PrEP is anti-HIV medicine that keeps HIV-negative people from being infected. There is a single pill that is taken once daily, and if you take it regularly, it is highly effective at prevention people from being infected. Now, how do you feel about PrEP?
122. *R: I understand it very well because it helps one not to get HIV infection*
123. I: If PrEP was made available to HIV negative men and women. Do you think you could advise your HIV negative peers to accept to take PrEP?
124. *R: Yes it’s important because it helps those who don’t know their status*
125. I: We are talking about PrEP, not PEP, where you know the status of someone who is positive so that you don’t get infected. What are the reasons you would encourage your clients to take PrEP?
126. *R: The reason can be, when one has a problem and the other one knows, they have different expectations, if it’s a family and they think it’s okay for them to be positive then it not okay, but if they are using prep then it is helpful to the one who doesn’t have the virus*
127. I: If PrEP becomes available, what is your opinion on integrating PrEP with Voluntary Medical Male circumcision services? Would you encourage clients to take PrEP?
128. *R: Especially when the man has received circumcision, these drugs are helpful because when one gets circumcised, we expect that man to be protected, so when he receives one part of helping his health, he needs to get the other service that can help him not to get infected so it I really helpful to men*
129. I: You would love to tell people about receiving PrEP?
130. *R: Yes*
131. I: How do you think PrEP would be offered here at Voluntary Medical Male Circumcision clinic?
132. *R: It can be offered here when they get circumcised and if we see that there Is a problem in their family*
133. I: What do you think could be the concern and barriers to integrating PrEP Voluntary Medical Male Circumcision services?
134. *R: Concerns are there maybe because of those that are circumcised to be explaining about prep, like there are drugs that help to prevent getting HIV , some people would not like the information hence concerns are there*
135. I: What do you think should be done to address these concerns and barrier?
136. *R: There is need to end it when we meet these people, teaching them about the importance of the medicine and how they can live their lives from the tie they have been circumcised*
137. I: Let us talk about other services. If you were given powers to choose and integrate services in Voluntary Medical Male Clinics, what are the services that you would think of to Integrate?
138. *R: Combining in terms of VMMC and other services?*
139. I: Yes.
140. *R: We can be giving women encouraging women because it seems like we are only encouraging men only, so as a family, men should be telling their wives to be coming together, o that these women should not be a threat to their husbands*
141. I: From what we have discussed which ones you would choose
142. *R: I would love to integrate family planning and HIV testing as a family and bringing of women for cervical cancer screening and many other problems*
143. I: Explain to me what the reasons are for your choices.
144. *R: On all three I can say that, the goodness I see in choosing them is that, when you help a family it has a great expectation as a family not to have doubt from either side*
145. I: What about family planning, why did you choose family planning
146. *R: Because now days giving birth regularly are a risk to one’s life hence combining family planning is helpful to help in controlling birth rates.*
147. I: Why cancer screening?
148. *R: It’s like helping women to walk in the same line with the man*
149. I: How do you think these services would be offered in the clinic?
150. *R: All of these three can be offered in line with time and the days that have been set when they come together as a family, but helping each one individually the way they wanted*
151. I: What about the place in a VMMC clinic?
152. *R: The place should be there can be to ways let me talk about one way, it can be in the same clinic*
153. I: In the same room for circumcision or another room?
154. *R: Another room but in the same clinic*
155. I: What about those with cancer?
156. *R: It can be offered at the same clinic but another side, when we help the man, we should also make sure that the woman is also helped as a family so that they should be moving forward together as a family*
157. I: Thank you for taking your time to discuss with me today. Your answers will be very helpful in improving the health service delivery at Voluntary Medical Male circumcision clinics. Before we close, do you have anything to say?
158. *R: What I can say that by the time we are encouraging people to get circumcised, lest not get discouraged because it’s a good and a big project that needs to reach every part of Malawi so that even young ones should have a bright future*
159. I: Again thank you so much.

END.

**D43 STUDY**

**Date of Interview: 24 August 2018**

**Type of Participant: Peer and Clinic Aides**

**Interview Number: D-43-0044**

**Interviewer: I.N.**

**Total Interview Time: 32 minutes 56 seconds**

**Interview Summary:** **(from summary sheet)**

| **SERVICE TO BE INTERGRATED** | **THOUGHTS ON INTERGRATION** |
| --- | --- |
| Couple HIV Testing and Counseling | Says this would be good for he believes this will bring about couple unity and knowledge about each other’s health status. |
| STI Services | Says this will be good for people will get easy treatment in order to get better circumcision help. |
| Family Planning | Such an integration would bring about couple unity. |
| Cervical Cancer Screening | Would give women the chance to easily know how their cervix is. |
| PrEP | Believes this would help create a safer environment for those who are HIV negative. |
| Other Services | Says he would like the integration of cervical cancer screening and couple HIV testing together with male circumcision services. |

**Remarks:** The participant was not open and shy. This resulted into probing a number of questions for clarifications.

Interview Texts:

1. I: Thank you for taking the time to talk with me today. I would like to ask you some questions today about the way you feel and what you think about some issues related to the service you provide and how we can include other services in Voluntary Medical Male Circumcision clinics. There are no right or wrong answers to these questions. We would like to hear your opinion and your experiences in your own words. Do you have any questions before we begin?
2. *R: Questions will be given at the end.*
3. I: Can you tell me how you are involved in the client care at this clinic?
4. *R. I just get to find clients and bring them to this clinic. That’s what I do in order to not leave the work for a single person.*
5. I: Does your clients talk to you about how the services are provided here?
6. *R: Yes, they do talk to us.*
7. I. Can you give me an example of a time that your client talked to you about the services he received here.
8. *R: Yes, I could. The service was that when they come to a circumcision clinic they get to learn a lot of things and they are given guidance that once they have been circumcised they become protected one part from sexually transmitted infection. That one thing is what I find to be the most logical thing we get to talk about with the males that get to come to this male circumcision clinic.*
9. I: Now let us talk about partner HIV testing here at the Voluntary Medical Male circumcision clinic. Tell me what happens if a man brings a spouse here at the Voluntary Medical Male circumcision clinic?
10. *R: When a man comes to this circumcision clinic with his partner, he is not immediately circumcised but is first tested for HIV.*
11. I: Have you ever seen a man bring their spouse here at the clinic?
12. *R: Yes, I have ever seen.*
13. I: What do you think are the motivators that make the men bring their spouses here for testing?
14. *R: What motivates them is the need to be open with each other. That’s why they are strong enough to come get tested together with their spouse.*
15. I: For those who do not bring their partners what do you think demotivates men to bring their partners here for HIV counselling and testing
16. *R: What demotivates them is lack of openness in their marriage and doing things that their spouses are unaware of which is actually destroying the other person’s future.*
17. I: What do you think can be done to make men bring their partners here for couple testing and counselling?
18. *R: These men should be sensitized about the benefits of getting tested together with their spouses.*
19. I: You as a health care provider, what is your opinion on integrating couple counseling with Voluntary Medical Male circumcision services?
20. *R. I think it is a really good idea. Something worth so much. Because, from what I have already explained, when you come as a couple for testing you are aware of your body’s status for that if you were involved in indecent acts you start taking caution which means together as a couple you do things carefully.*
21. I: What do you think are the barriers and concerns on this integration?
22. *R: I don’t think there would be none, not even one. Because couples get to agree on the choice together.*
23. I: Which means if the integration was to occur there would be no barriers?
24. *R. There would be no problem. Because the service would not be undertaken at a similar place as to that of circumcision. Be it a room for the service to be undertaken.*
25. I: Now I would like to discuss with you about sexual reproductive health services and Pills for HIV prevention: called pre-exposure prophylaxis (PrEP). Sexual reproductive health includes services that promote good sexual health and reproduction. They include but not limited to family planning, cervical cancer screening sexual transmitted infection (STI) management, and many more. Today we will only discuss about family planning, diagnosis and management of STIs, Cervical cancer screening, and PREP. We will look at each of these one by one. Let us start with STI services. Explain to me what happens if a client is suspected or diagnosed with an STI here?
26. *R: That person does not get circumcised but is counselled and advised to follow instructions. The person gets circumcised after they get healed from the STI’s.*
27. I: You as a health care provider what is your opinion on integrating STI services with Voluntary Medical Male circumcision services.
28. *R: I see that to be good.*
29. I: What is it that you do not like the integration of STI with Voluntary Medical Male Circumcision services?
30. *R: There is nothing I do not like about it.*
31. I: What is it that you like of the integration STI services with Voluntary Medical Male Circumcision services?
32. *R****:*** *I take it as being good because the person found with STI’s is counselled to come back later on after the infections have healed.*
33. I: Do you like this integration*?*
34. *R. Yes I do like it.*
35. I**:** How do you think STI services should be offered at the Voluntary Medical Male circumcision clinic?
36. *R: There is a need that there should be a special room for this service.*
37. I. When should this service be offered to the clients? Before or after being circumcised?
38. *R. It should be given before circumcision.*
39. I: What do you think are the barriers and concerns on this integration
40. *R: I don’t believe there would be any barriers.*
41. I: Let us now talk about Family planning. What does family planning mean?
42. *R. When they say family planning it does not concern one person only but the two of you as a couple so that they should do it as one. And if a woman has given birth and after a while she wants to involve herself with family planning she has to tell her husband so that they make that decision together for if they do it as one it is a little better unlike if she gets to do it without telling the husband which is wrong.*
43. I. Do you provide family planning methods at a male circumcision clinic?
44. *R. No they do not.*
45. I: What is your opinion if they are to integrate male circumcision and family planning?
46. *R. Like I said if they were to integrate the two it would be that they would be undertaken in separate rooms.*
47. I: What is it that you would not like the integration of family planning in Voluntary Medical Male Circumcision Clinic?
48. *R: There would be no problem there.*
49. I: What is it that you like of the integration of Family planning with Voluntary Medical Male Circumcision services?
50. *R: You would be going there as a couple, there would be unity among couples.*
51. I: How do you think family planning services can be offered within Voluntary Medical Male circumcision clinics?
52. *R: It will depend on the time at which they have come for such an assistance.*
53. I: What do you think are the barriers and concerns with this integration?
54. *R: There wouldn’t be any barriers because people will go to where ever the want to get assistance.*
55. I: Let us talk about cervical cancer screening. Explain to me what happens if a woman needs cervical cancer screening?
56. *R: When they come to seek such a service, they get undressed and then are screened in their vaginal area.*
57. I. Do they give out this service at male circumcision clinics?
58. *R. No they do not do that. Only if there is a place specific for that at the clinic.*
59. I: As a health care provider, what is your opinion on integrating cervical cancer screening in Voluntary Medical Male Circumcision services
60. *R: It would be good that they would both be at the same place so that cervical cancer screening service can also easily be accessed.*
61. I: That means you like this integration?
62. *R. Yes I do.*
63. I: What is it that you would not like about cervical cancer screening integration with Voluntary Medical Male Circumcision services?
64. *R: There is nothing I would not like about it.*
65. I: What is it that you like of the integration cervical cancer screening with Voluntary Medical Male Circumcision services? How do you think is the best way to offer cancer screening within Voluntary Medical Male circumcision clinics?
66. *R: In the morning hours. The place to offer such a service should be well protected.*
67. I: Should the place be inside the clinic?
68. *R: The place should be inside the clinic but should be private.*
69. I: Is the husband supposed to be present during cervical cancer screening of their spouse?
70. *R. That would depend on the service provider allowing that the man should be present to see how the procedure works and how this cancer looks like.*
71. I: What do you think are the barriers and concerns on this integration?
72. *R: For me, there would be nothing that would act as a barrier to this but maybe others who would not like such an integration would create barriers.*
73. I: What do you think should be done to address these concerns and barriers?
74. *R: Conducting sensitization campaigns so that others should take part as well.*
75. I: Now let us discuss about PrEP. Have you heard about this before?
76. *R: No I have never heard about this.*
77. I: If you have not heard about PrEP, I will explain how the medicine works. PrEP is anti-HIV medicine that keeps HIV-negative people from being infected. There is a single pill that is taken once daily, and if you take it regularly, it is highly effective at prevention people from being infected. Now how do you feel about PrEP?
78. *R: I feel that PREP is good.*
79. I: If PrEP was made available to HIV negative men and women. Do you think you could advise your HIV negative clients to accept to take PrEP?
80. *R. Yes that would be possible.*
81. I: What are the reasons you would encourage your clients to take PrEP?
82. *R: Since PREP is a sort of protection from HIV for those who are negative so when they take these drugs their bodies become protected for that I would encourage others to not be afraid but to come to our clinic to receive council about this PREP.*
83. I: If PrEP becomes available, what is your opinion on integrating PrEP with Voluntary Medical Male circumcision services? Would you encourage clients to take it?
84. *R: Yes, I would encourage it. For once you get tested and you are found negative it would good council to receive such drugs time after time as a couple.*
85. I: How do you think PrEP be offered in Voluntary Medical Male clinics?
86. *R: Coming here to get such a service.*
87. I: When do you think PREP be administered in relation to male circumcision?
88. *R: A person should receive this PREP after getting circumcised.*
89. I: What do you think are the concern and barriers to integrating PrEP in Voluntary Medical Male services?
90. *R. I see no barriers here because these services have to be easy to access. Same clinic different rooms*
91. I: Let us talk about other services. If you were given powers to choose and integrate services in Voluntary Medical Male Clinics, what are the services that you would think of Integrate?
92. *R. Cervical cancer screening and HIV testing as a couple*
93. I: Explain to me what the reasons are for your choices.  *Cervical cancer screening?*
94. *R. This is good for women; it is good for them to know how they are rather being unaware thinking they are okay when actually they are not for this type of cancer is very dangerous.*
95. I. HIV testing as a couple?
96. *R. This is also good for you get to come for testing as a couple so that you both get to know your status. For that this would be good.*
97. I: How do you think these services should be offered in the clinic?
98. *R. these should be offered at the clinic in private rooms.*
99. I: Thank you for taking your time to discuss with me today. Your answers will be very helpful in improving the health service delivery at Voluntary Medical Male circumcision clinics. Before we close, do you have anything to say?
100. *R. It is just my concern to encourage you people to continue teaching us for no one knows all, you get to learn from others as well and we will keep on encouraging others who haven’t gotten circumcised to do so and for them to also get tested to know how they are not to think that they are already done for.*
101. I: Again, thank you so much for taking your time to speak with me.

END.

**D 43 STUDY**

**Date of Interview: 24 August 2018**

**Type of Participant: Peer and clinic aides**

**Interview Number: D-43-0045**

**Interviewer: I. N.**

**Total Interview Time: 37 minutes 50 seconds**

**Interview Summary:** **(from summary sheet)**

| **SERVICE TO BE INTERGRATED** | **THOUGHTS ON INTERGRATION** |
| --- | --- |
| Couple HIV Testing and Counseling | Says it is a good idea because couples will be able to know the status of their partner which will help prevent HIV transmission. |
| STI Services | Believes that this integration will help reduce the spread of sexually transmitted infections |
| Family Planning | Says this integration would create problems if there are not many health assistants. |
| Cervical Cancer Screening | Thinks such an integration would be beneficial for both males and females for it would help them know how their bodies are. |
| PrEP | Believes that this kind of integration will be beneficial for circumcised males for circumcision does not give a person total protection from HIV |
| Other Services | Believes that VMC should be integrated with PREP and HIV testing as a couple. |

**Remarks:** Participant was relaxed and explained clearly.

**Interview Texts:**

1. I: Thank you for taking the time to talk with me today. I would like to ask you some questions today about the way you feel and what you think about some issues related to the service you provide and how we can include other services in Voluntary Medical Male Circumcision clinics. There are no right or wrong answers to these questions. We would like to hear your opinion and your experiences in your own words. Do you have any questions before we begin?
2. *R: No I do not.*
3. I: Can you tell me how you are involved in the client care at this clinic?
4. *R: When organizations approach us so that we can convince others who can help us solve problems that we might have*
5. I: What can you tell us about your involvement with male circumcision?
6. *R: Since I am already circumcised, I approach others who have never been circumcised so that they too get circumcised in order to prevent different problems.*
7. I: When you say “to keep away from different problems” what exactly are these problems?
8. *R: Problems like sexually transmitted diseases which in truth are not one hundred percent avoidable but that we are somehow being protected from those diseases.*
9. I: Does your clients talk to you about how the services are provided here?
10. *R: yes, they do talk to us.*
11. I: Can you give me an example of a time that your client talked to you about the services he received here?
12. *R: Yes. I have this friend who was having problems before he got circumcised, he was experiencing pain when doing some activities, but after getting circumcised he’s found those activities easier to do.*
13. I: Now let us talk about partner HIV testing here at the Voluntary Medical Male circumcision clinic. Tell me what happens if a man brings a spouse here at the Voluntary Medical Male circumcision clinic?
14. *R: It happens that a person does not know the status of their body, maybe they keep on moving from one disease to the next, because of no knowledge about their body we search for assistance so that we go to the hospital in order to follow proper advice about how the body works for things to work our way.*
15. I: Have you ever seen a man bring their spouse here at the clinic?
16. *R: Yes, a lot of them.*
17. I: What do you think are the motivators that make the men bring their spouses here for testing
18. *R: It could be that there is lack of trust between the two, that maybe one partner keeps on being attacked by different diseases successively, so by trying to prevent those diseases from attacking the other person, they get to decide to go for testing to figure out the problem.*
19. I: For those who do not bring their partners what do you think demotivates men to bring their partners here for HIV counseling and testing
20. *R: Those men just do not have Self Love, because by not doing so the will not be able to know the status of their bodies.*
21. I: What do you think can be done to make men bring their partners here for couple testing and counseling?
22. *R: We ask that organizations should sensitize people in rural areas as well as those in urban areas encouraging them to take part in this so that problems like these should reduce in our country.*
23. I: You as a health care provider, what is your opinion on integrating couple counseling with Voluntary Medical Male circumcision services?
24. *R: I could take part in this, because a person having to come across such problems and taking no action on it is not logical, but they should follow procedures that will help take your life further.*
25. I: You as a health care provider, how do you receive integration of couple counseling with Voluntary Medical Male circumcision services?
26. *R: I receive it well.*
27. I: Why are you receiving it well?
28. *R: Because it will reduce the problems we are meeting.*
29. I: What are these problems you say you are meeting?
30. *R: Problems that involve transmission through sexual intercourse for not knowing each other’s status.*
31. I: What do you think are the barriers and concerns on this integration
32. *R: Barriers could be there depending on the people that we have asked help from and they do not want to help us. So this could be a barrier because we would have nowhere else to seek for such assistance.*
33. I: What do you think can be done to overcome these concerns and barriers to couple counseling in Voluntary Medical Male circumcision services clinic?
34. *R: On that, there’s a need to take part in encouraging those organizations so that they should be able to help give us assistance.*
35. I: When you say assisting you in finding help, what exactly is this help?
36. *R: Assistance in helping with cervical cancer*
37. I: How are you relating this male circumcision clinic to getting help for cervical cancer?
38. *R: I am saying that because if there could be a chance of pregnancy, it is possible to protect the unborn child so that the baby is free from any problem, or if a person has problems giving birth they’ll be able to receive help.*
39. I: Now I would like to discuss with you about sexual reproductive health services and Pills for HIV prevention: called pre-exposure prophylaxis (PrEP). Sexual reproductive health includes services that promote good sexual health and reproduction. They include but not limited to family planning, cervical cancer screening sexual transmitted infection (STI) management, and many more. Today we will only discuss about family planning, diagnosis and management of STIs, Cervical cancer screening, and PrEP. We will look at each of these one by one. Let us start with STI services. Explain to me what happens if a client is suspected or diagnosed with an STI here?
40. *R: What happens is that, when a person is found to have STI’s, is that some people do not know the status of their bodies, that they are unaware of how best they can take care of their bodies in order to live a healthier life.*
41. I: Now, a person has come for circumcision and it happens that he has STI’s such as gonorrhea and genital warts, how does it work out for them?
42. *R: In this situation, doctors are given work, because at the time this person wasn’t feeling well, he did not bother to go to the hospital to seek medical help, now you are going there because you have a problem, now you give doctors work to try to help you with your problem, but it works out eventually.*
43. I: So now, how does it work out for this person, is he sent back or does he get circumcised?
44. *R: No, he doesn’t get circumcised. But he is just given medicine to help with his STI’s, so that when he comes back later on he should get assisted after the infections are gone.*
45. I: You as a health care provider what is your opinion on integrating STI services with Voluntary Medical Male circumcision services.
46. *R: I see that it is a good method to let people know that they should lead a healthy life.*
47. I: So if it would happen that STI services have been integrated with male circumcision, how are you receiving such a development?
48. *R: I am receiving it well, but the place for providing services for STI’s should not stand together with this one for male circumcision.*
49. I: What do you mean exactly about the place being separate?
50. *R: It could be the same hospital, but then there should be a room separate from the one for male circumcision.*
51. I: How do you think STI services should be offered at the Voluntary Medical Male circumcision clinic?
52. *R: We cannot say the specific time for giving out this service because that is supposed to be decided on by those in charge.*
53. I: Believe I am to ask in this way, according to the circumcision follow out, when is it supposed to be given out?
54. *R: Before you get circumcised.*
55. I: Why should it be given before circumcision?
56. *R: So that you should get to hear some advice first, then you should receive treatment afterwards.*
57. I: What do you think are the barriers and concerns on this integration
58. *R: Barriers are to be there because of the large numbers of people or that there is less medical equipment.*
59. I: What do you think should be done to address these concerns and barriers?
60. *R: It is necessary that we should find friends that would be able to assist us in this.*
61. I: How are these people to help us?
62. *R: Finding friend’s like organizations and letting them know of the problems that we are facing here at the clinic.*
63. I: So you mean in terms of the equipment?
64. *R: Yes, in terms of the equipment.*
65. I: Let us talk about Family planning. Explain to me what family planning means?
66. *R: Family planning means that if a person has a child they should protect that a young child so that she/he grows and also that you should not get pregnant early.*
67. I: Which family planning methods do you know?
68. *R: Family planning methods that I know of is the Injection method.*
69. I: What about family planning methods for males?
70. *R: I do not know any.*
71. I: There is male vasectomy, implant, and injections as well, some take pills including condoms. *so do you give out family planning at this clinic?*
72. *R: No, we do not.*
73. I: Explain to me what happens if a client needs a family planning methods (vasectomy for men and family planning for female partners?
74. *R: They just to give out directions on where you get that treatment.*
75. I: As a health care provider, what is your opinion on integrating Family planning in circumcision services
76. *R: I see that there could be a problem there because for there to happen that a person is involved in doing two things it would create too much work which means you are supposed to give others that extra work for you to handle the other department.*
77. I: What is it that you would not like the integration of family planning in Voluntary Medical Male Circumcision Clinic?
78. *R: What I would like about it is that a person would not get assisted at both matters at the same time for it would be difficult to do so.*
79. I: What is it that you like of the integration of Family planning with Voluntary Medical Male Circumcision services**?**
80. *R: What I would like about it is that when a person goes to get help with family planning, and then you also get to decide that the way I am I should also go get circumcised you would get assisted in no time.*
81. I: How do you think family planning services can be offered within Voluntary Medical Male circumcision clinics?
82. *R: Finding people who are well abled to undertake this task while you are doing something else.*
83. I: Where do you think is the best place to give out family planning methods according to the follow up of male circumcision?
84. *R: These methods should not be given where male circumcision is undertaken.*
85. I: That is, it should be undertaken somewhere else?
86. *R: Yes it is supposed to be undertaken somewhere else.*
87. I: So where do you think this other place should be situated? In the same clinic or outside the clinic or inside the clinic but in a private place?
88. *R: In the same clinic but in a private room.*
89. I: Why should the place be situated in a private place?
90. *R: Because a person can go and get assisted without feeling insecure about being watched by others. But the activity should be private between the patient and medical practitioner.*
91. I: What do you think are the barriers and concerns with this integration?
92. *R: Barriers could be there because it would take up time for the clinic assistants for when you want to assist someone with family planning methods someone else would be wanting your assistance with male circumcision and one person cannot achieve all that.*
93. I: What do you think should be done to address these concerns and barriers?
94. *R: There is a need to get assistance in terms of money, which will be used to govern organizations that will hire other people to work as assistants.*
95. Explain to me what happens if a woman needs cervical cancer screening?
96. *R: It depends on how you feel in your body which will make you decide that you should go and get screened for cervical cancer.*
97. *I: Do you provide cervical cancer screening at your clinic?*
98. *R: No we do not.*
99. I: As a health care provider, what is your opinion on integrating cervical cancer screening in Voluntary Medical Male Circumcision services.
100. *R: That would be a problem because men and women cannot be assisted in one place.*
101. *I:* So what do you think should be done so that it should be like that?
102. *R: there is a need to find a special room for assisting these women in the same clinic.*
103. **I: Probes:** *What is it that you would not like about cervical cancer screening integration with* Voluntary Medical Male *Circumcision services?*

*R: nothing What is it that you like of the integration cervical cancer screening with* Voluntary Medical Male *Circumcision services?*

*R: what I would like about it is that a person would get assisted according to the help they seek.*

1. What do you think are the barriers and concerns on this integration?
2. *R: Barriers could be there because there are no tools to undertake cervical cancer screening.*
3. *I: So what if these tools were available?*
4. *R: there would be no barriers.*
5. I: What do you think should be done to address these concerns and barriers?
6. *R: since every time you are doing something you are always prepared I don’t think there would be any barriers.*
7. Now let us discuss about PrEP. Have you heard about this before? If you have heard about PrEP, please tell me what you know about PrEP?
8. *R: No I have never heard about prep.*
9. I: If you have not heard about PrEP, I will explain how the medicine works. PrEP is anti-HIV medicine that keeps HIV-negative people from being infected. There is a single pill that is taken once daily, and if you take it regularly, it is highly effective at prevention people from being infected. Now how do you feel about PrEP?
10. *R: PREP is good for it protects you from sexually transmitted diseases so that immunity in the body should be at its best.*
11. **I:** If PrEP was made available to HIV- men and women. Do you think you could advise your HIV negative clients to accept to take PrEP?
12. *R: No. PREP is not important to such people.*
13. *I:* Why is it not important?
14. *R: Because they haven’t come across this problem yet.*
15. I: Which problem is that?
16. *R: Sexually transmitted diseases.*
17. I: Have you really grasped how PREP works?
18. *R: Yes, I have. PREP works in people who have not yet been diagnosed with the disease.*
19. I: Those who are negative is it?
20. *R: No, those who are positive.*
21. I: now that I have explained how PREP works to you again, is it important that PREP be made available to people who are HIV negative?
22. *R: Yes, it is important because it will help reduce HIV.*
23. I: If No: what are your concerns? If yes, what are the reasons you would encourage your clients to take PrEP?
24. *R:* *It will help reduce HIV.*
25. I: If PrEP becomes available, what is your opinion on integrating PrEP with Voluntary Medical Male circumcision services? Would you encourage clients to take it?
26. *R: It is necessary to encourage it for male circumcision is not a highly trusted way that would prevent one from contracting diseases so it is necessary that one has to be aware of PREP so that when you are involved in some activities you should know that you have helped yourself. Yes, I would encourage clients to take it.*
27. I: How do you think PrEP be offered in Voluntary Medical Male clinics?
28. *R: In the same clinic.*
29. I: When do you think PREP be administered to a client?
30. *R: After circumcision.*
31. I: What do you think are the concern and barriers to integrating PrEP in Voluntary Medical Male services…
32. *R: Barriers would be there depending on the equipment we have.*
33. I: And if the equipment were there?
34. *R: There wouldn’t be any barriers.*
35. I: What do you think should be done to address these concerns and barrier?
36. *R: Encouraging people to assist us so that we also get to help others.*
37. I: How would you encourage these people?
38. *R: We would encourage them by, organizations that’s all. Those that would be able to assist us.*
39. I: Other Services: If you were given powers to choose and integrate services in Voluntary Medical Male Clinics, what are the services that you would think of Integrate?
40. *R: Giving out PREP and cervical cancer screening including HIV testing as a couple.*
41. I: Explain to me what the reasons are for your choices.*..* HIV testing as a couple...
42. *R: I would be glad because you would be able to know your status. I would choose that the service should be there, it should be available indeed.*
43. I: What about PREP?
44. *R: I have chosen PREP because PREP will help us in cases where you want to have sexual intercourse with someone yet you do not know their status so there is a need for PREP to be there so that we should be able to help ourselves so that we should not get into problems.*
45. I: You also chose cervical cancer screening, why have you chosen this one to be integrated with male circumcision?
46. *R: I don’t agree with that one.*
47. I: You are denying it now?
48. *R: Yes, because there is a need to find a separate room for that, but integrating them, things do not go well.*
49. I: How do you think these services should be offered in the clinic?
50. *R: Should be given by the doctors.*
51. I: How about places for such services?
52. *R: Places are to be divided up. Separate rooms for each service.*
53. I: Thank you for taking your time to discuss with me today. Your answers will be very helpful in improving the health service delivery at Voluntary Medical Male circumcision clinics. Before we close, do you have anything to say?
54. *R: No, I have nothing more to say.*
55. I: Again, thank you so much for taking your time to speak with me.

END**D43 STUDY**

**Date of Interview: 05 September 2018**

**Type of Participant: Peer and clinic aides**

**Interview Number: D-43-0047**

**Interviewer: I. N.**

**Total Interview Time: 36 minutes** 44 **seconds**

**Interview Summary:** **(from summary sheet)**

| **SERVICE TO BE INTERGRATED** | **THOUGHTS ON INTERGRATION** |
| --- | --- |
| Couple HIV Testing and Counseling | Thinks it is good and very joyous issue because these things enter in a person’s body in different ways but by the end of the day there is only one body. |
| STI Services | It is very good idea |
| Family Planning | It is very helpful because everything needs to go through a particular protocol. |
| Cervical Cancer Screening | It is very interesting since you have put together all the services rather than if they are to be provided separately |
| PrEP | It is necessary because it is a way of protecting |
| Other Services | None |

**Remarks:** Participant was relaxed, calm and knowledgeable. He was able to explain in details and clearly.

**Interview Texts:**

1. I: Thank you for taking your time to talk to me today, I would like to ask you some questions on how you feel and opinion on the services you provide here and how we can include other services in the voluntary male circumcision clinics. There are no right or wrong answers to these questions. We would like to hear your experiences in your own words. Do you have any questions before we begin?
2. *R: No. will be asking the question later on*
3. I: Alright fine. So can you tell me the role you play in the services provided here at the VMMC clinic?
4. *R: To my side the way I have seen circumcision, it is very good and when it was first introduced we received the idea with keen interest and I immediately came to the clinic to do circumcision and it is ideal for hygiene and am now living a happy life now that am married, there is some skin diseases on the penis on the man that you can transmit to the woman but since ma circumcised am living a happy life. That’s what I can tell you.*
5. I: Thank you. So like in the circumcision clinic, what role do you play?
6. *R: On the part of circumcision, I have taken the role of telling my friends to come for circumcision immediately. Circumcision is good and hygienic to everyone interested to come do circumcision so I tell all my friends to come for medical circumcision.*
7. I: Do you ever discuss with your friends how the services are provided here?
8. *R: Yes, we discuss that this circumcision is not like the circumcision that our friends do. This circumcision is very important in the sense that you first receive counseling and if you follow it you cannot have any problems. You should not take it for granted but you need to do circumcision when you have made up your mind.*
9. I: So your friends that you encourage to come for circumcision, are they able to talk to you about the services provided here?
10. *R: Yes. Almost three guys have agreed that they thought this program was difficult but it is a good program because of what we have been experiencing in the past regarding on the sores on the penis/private parts, we no longer experience such.*
11. I: Now let us talk about HIV couple testing at this clinic. Tell me what happens when a man brings a wife here at the voluntary male medical circumcision clinics?
12. *R: Taking his loved one to do screening for the door……. HIV testing is very good because you are free and you play a role in protecting your family and whatever comes you are able to know where it may come from.*
13. I: Have you ever seen a man coming to the clinic with his partner for HIV testing as a couple?
14. *R: No I have never seen. I would be the first one that my wife should come and experience the services at the hospital.*
15. I: What do you think makes men to bring their loved ones to the VMMC clinics?
16. *R: That happens because of the love between the couple and not keeping secrets from each other and that happens if you don’t hide anything from each other and you put God first before everything in your marriage.*
17. I: Okay. So men that do not bring their spouses to the clinic for HIV couple testing, what do you thinks makes them not to do so?
18. *R: It is just because of lack conscious but still I wish people could take part to come to the clinic and do the test with their loved ones.*
19. I: So what do you think should be done so that men start bringing their loved ones for couple HIV testing?
20. *R: I think we just need to choose a day and call people to a meeting to discuss with them the essence of doing that so that one day they can make a decision to come and do the testing of problems in a person’s body like cervical cancer. Coming with your loved one to the hospital is not a bad thing.*
21. I: So you as health service provider support personnel, what do you think about the integration of couple HIV testing with VMMC services?
22. *R: On that one I see it is good and it is a very joyous issue if these services are to be offered together because these things enter in a person’s body in different ways but by the end of the day there is only one body. If the disease enters body through the private parts, it is not possible that somewhere things will be fine so it is important we protect everywhere so that there are no possibilities of contracting diseases.*
23. I: What do you think can be the barriers and concerns to this integration?
24. *R: On this one there cannot be barriers because if it has been provided by God it becomes possible, first the person has to make a decision and know that where you are going there will be results. When you are stressed things don’t usually go the right way.*
25. I: Okay fine. Now I would like us to discuss about sexual reproductive health services, pills for HIV prevention called pre-Exposure Prophylaxis. sexual reproductive health include services that promote good sexual health and reproduction they include but not limited to family planning, cervical cancer screening, Sexually Transmitted Infections management and condom distribution and many more. Today we will only discuss about family planning, cervical cancer screening, Sexually Transmitted Infections management and PrEP. We will look into each one of these one by one, so let’s start with STI services. Explain to me what happens when a person is being suspected or has been diagnosed with STIs?
26. *R: The person becomes stressed because their body’s ability to fight infections gets weakened and by this time it is like they have received the news they never expected so they become stressed but if they are not stressed and able to follow the instructions from the clinic they live just as normal.*
27. I: So when a person has come for circumcision and has been diagnosed with sexually transmitted infections, what happens to them?
28. *R: They receive counselling regarding how they can protect themselves for the future since the STIs were not expected.*
29. I: But do they proceed with the circumcision if found with the STIs?
30. *R: On that part since everyone goes into the circumcision room alone and am not sure how it goes but like on my part I just received my results.*
31. I: Health providers never explained to you what may happen if you have been diagnosed with STIs?
32. *R: They just explained that they want to get the results for the test to understand whether a person is doing circumcision while infected with HIV or not and on my part I did.*
33. I: If we are to integrate STI screening within the VMMC clinics, what are your thoughts about this idea?
34. *R: I think it would be a very good idea.*
35. I: What would interest you the most about this integration?
36. *R: Because I have been protected from the infection before testing, so when I have been tested and receive the drugs it would not be easy for other infection to come in. like I have never tested and not aware on how I can protect myself.*
37. I: What things are you not okay with about the integration of STI screening within the circumcision clinics?
38. *R: It is goo to refrain from having multiple sexual intercourse with other women because it is only you that can take care of your life, if I have protected my spouses, she should as well protect my life in this way infections may not come.*
39. I: So on this integration of STI screening and circumcision, what can you not like?
40. *R: On that one all these services provided we need to follow one way, like we talk about……may please ask again that question?*
41. I: Ah we are talking about integrating STI screening and circumcision, what can you not like about it?
42. *R: I think I would agree to all these because there is family planning…….*
43. I: no we are talking about these two services now, screening of STIs and circumcision, what wouldn’t you agree with about this integration?
44. *R: I would be happy with both.*
45. I: How do you think this service can be provided here at the clinic?
46. *R: We can be having its special day, like today you provide one part of the service and another day the other part that can go well because all of them at once I don’t think it can work.*
47. I: So if a person has come for circumcision, this service should be provided where exactly, within the clinic, in a private space or outside the clinic?
48. *R: Circumcision service should be provided within the clinic in a private room not outside.*
49. I: Yes, that’s for circumcision, but for screening of STIs and management within the circumcision process, how should it be provided? Or what time, after circumcision or before?
50. *R: Before circumcision needs to be screened.*
51. I: Okay. So time or place for screening, how should it be done in terms of place and time?
52. *R: Within the circumcision clinic not outside because this process is very private it can’t be done anyhow.*
53. I: Are there any concerns or barriers to this integration?
54. *R: There are no barriers, I would say there are no concerns.*
55. I: Okay now let us talk about family planning. Explain to me what happens when a person wants a family planning method like vasectomy, what happens here?
56. *R: On the part of family planning, because now there are a lot of family planning methods. Some are using the loop method that works for five years or three years based on what the couple has agreed to do. Some will go foe vasectomy or tube legation if you have had enough children you may agree on the method to follow.*
57. I: So if a person wants family planning, how does it go at the circumcision clinic?
58. *R: On that part I have never been told regarding family planning during circumcision.*
59. I: As a health service personnel provider, what is your opinion on integrating family planning within the circumcision clinics?
60. *R: I think that is very helpful because everything needs to go through a particular protocol.*
61. I: So what can make you not to want the integration of family planning with male medical circumcision?
62. *R: Ah…on that one repeat again.*
63. I: What is it you can’t like about this integration of family planning within the circumcision clinic?
64. *R: I think it is very good to integrate if they have agreed it good.*
65. I: How do you think family planning should be offered at the circumcision clinic?
66. *R: It can be offered based on the agreement made between the two in a family like husband and wife as long as they choose a method to use and they are happy with it.*
67. I: So in terms of place, where should it be placed, inside the clinic, outside or in a private room within the clinic?
68. *R: It should be offered inside the clinic in a private room not outside because these methods needs privacy and outside can’t be good.*
69. I: So if a man comes for circumcision, what’s the best time to provide him with the family planning service?
70. *R: He should receive the service after the circumcision.*
71. I: What do you think are the barriers or concerns to this integration?
72. *R: I think there are no barriers or concerns as I have already said that if these services are to come we just need to follow through.*
73. I: Now let us talk about cervical cancer screening. Explain to me, what happens here when a woman wants to do cervical cancer screening?
74. *R: It is important for women to go and do cancer screening to know their status and receive counseling.*
75. I: So like in the circumcision clinics, how does it go when a woman seeks cancer screening services?
76. *R: On that part I can’t really know because women do their things about cervical cancer screening alone same as men on circumcision since you are able to know things based on what you are going through.*
77. I: You as health service provider support personnel, what do you think about the integration of cervical cancer screening within the circumcision clinics?
78. *R: The role I can play on this one is just to continue encouraging my friends so that whatever comes we really need to be part of it and it is important for them to come to the clinic to do all these procedures.*
79. I: So if we are to do this integration, what can make you happy most?
80. *R: It is very interesting since you have put together all the services rather than if they are to be provided separately.*
81. I: Ah what can make you not to want this integration of cervical cancer screening and circumcision service?
82. *R: On that one I don’t think you can never want as long as you have agreed with your partner.*
83. I: But to you, how do you look at it to integrate cervical cancer screening with circumcision clinic, is there anything you can’t like?
84. *R: It is a good method.*
85. I: How do you think this method should be provided? Or I should ask that at the circumcision clinic in terms of place and time, how can this integration be offered?
86. *R: In terms of place it should be inside and time should be anytime either morning or afternoon but not in the evening*
87. I: What do you think can be the barriers and concerns to this integration?
88. *R: Ah on that one I don’t think there can be any concerns because stress is not good and you don’t need to be stressful to accomplish.*
89. I: Are there any barriers?
90. *R: Maybe the barriers in the course of the integration but you can always do your best to work on them.*
91. I: How do you think these barriers or concerns can be dealt with?
92. *R: These concerns of the barriers can be resolved by discussing about it even for several days until you agree on one method to follow.*
93. I: Now let us talk about PrEP. So I will explain to you how it works. It is an abbreviation that means Pre-Exposure Prophylaxis. *It is a pill that is taken by an HIV negative person so that it should protect the person from contracting HIV even if this person has sexual intercourse with an HIV positive person. If taken properly it is effective in protecting you fully from contracting HIV. Now how do you understand about PrEP?*
94. *R: It is a good method regarding that if you have taken PrEP as the doctors are saying you will live as you have just said.*
95. I: So how important is it to make PrEP available to men and women without HIV?
96. *R: Yes, it is necessary.*
97. I: What reasons so do you have about…. you can encourage people to take PrEP?
98. *R: Yes, I can encourage them because it is a way of protecting oneself from STIs.*
99. I: If PrEP is to be made available, what is your opinion about the integration of PrEP provision within the circumcision clinic?
100. *R: I can be happy because we now have the drugs we never had access of its use.*
101. I: In terms of place and time, how do you think PrEP should be offered here at the circumcision clinic?
102. *R: Ah at the time you have come with your spouse for counseling at the clinic and it should be done inside the clinic and not outside.*
103. I: What do you think are the barriers and concerns to this integration?
104. *R: On that one, I don’t think there any barriers and it is better to agree to follow but barriers can come sometime based on how you have perceived it.*
105. I: Let us look at the other part which is other services, if you are given the power to integrate other services to the VMMC services, what are the services you would think of integrating?
106. *R: I would choose PrEP and other services that can be provided.*
107. I: Apart from the methods we have discussed, what other services do you think should be offered here?
108. *R: Ah other services, maybe if we can get them in the course of the integrations but for now I cannot say.*
109. I: So why have you chosen PrEP.
110. *R: PrEP because it is the one that is protective from HIV.*
111. I: Thank you so much for taking your time to discuss these things today, your answers will be helpful in improving the health service delivery at circumcision clinics. Before we close, do you have anything to say.
112. *R: What I can say is that I would try my best to encourage my friends that have not yet done circumcision to come at the clinic since it is hygienic and they should not be afraid that there are challenges and it also protect women from cervical cancer so I would encourage everyone to take part in circumcision I think these can be my last words.*
113. I: Thank you so much.
114. *R: Thank you.*

END.

**D43 STUDY**

**Date of Interview: 05 September 2018**

**Type of Participant: Peer and clinic aides**

**Interview Number: D-43-0048**

**Interviewer: I. N.**

**Total Interview Time: 41 minutes** 00 **seconds**

**Interview Summary:** **(from summary sheet)**

| **SERVICE TO BE INTERGRATED** | **THOUGHTS ON INTERGRATION** |
| --- | --- |
| Couple HIV Testing and Counseling | Thinks it’s a very good because it is good to do things together and receive counseling together whether you have tested positive or negative. |
| STI Services | Thinks it is important to have a special branch to assist with this STI management so that if these infections persist you can still be assisted |
| Family Planning | Like the idea because it can brought happiness in a family |
| Cervical Cancer Screening | The family will be as new and it would also allow the woman to know her status if she is infected with the cancer. |
| PrEP | Thinks it will facilitate the act of unprotected sexual intercourse |
| Other Services | None |

**Remarks:** Participant was open, relaxed and confident. He explained the answers clearly and in brief.

**Interview Text:**

1. I: Thank you for taking your time to talk to me today, I would like to ask you some questions on how you feel and opinion on the services you provide here and how we can include other services in the voluntary male circumcision clinics. There are no right or wrong answers to these questions. We would like to hear your experiences in your own words. Do you have any questions before we begin?
2. *R: Mm… no I don’t have.*
3. I: Can you tell me the role you play in the services provided here at the VMMC clinic?
4. *R: The role that I play is to encourage my friends who have not yet done male medical circumcision to come to the clinic and get circumcised.*
5. I: Do your friends talk to you regarding the services that are provided here?
6. *R: Yes, they are able to talk to me*
7. I: Can you please tell me an example where a friend ever talked to you regarding the service they received here?
8. *R: Yes, I can explain, there is………. some men that came for circumcision, they told me that they are shy but because of my encouragement they now tell me that they were just delaying for nothing since they thought the procedure is very painful and not necessary*
9. I: Now let us talk about HIV couple testing. Tell me what happens when a man brings a wife here at the voluntary male medical circumcision clinics?
10. *R: Ah because when you are leaving for circumcision you agree as a family so it is all happiness to do the circumcision because the woman is protected.*
11. I: What do you think makes men to come with their spouses for HIV testing?
12. *R: I think it is because of a woman’s happiness that she is protected after proper explanation to her about the benefits of HIV*
13. I: But what do you think makes men to bring their partner for couple HIV testing at the clinic?
14. *R: Because they want to develop trust between each other.*
15. I: so what do you think makes other men not to bring their spouses here at the clinic for couple HIV testing?
16. *R: Because of lack of trust based on how they behave.*
17. I: You as health service provider support personnel, what is your opinion about integration couple HIV testing within the circumcision clinics?
18. *R: I think it was very good because it is good to do things together and receive counselling together whether you have tested positive or negative.*
19. I: What barriers and concerns do you think can come in between this integration?
20. *R: Ah I don’t think there can be any barriers but maybe they can come in if the couple don’t understand each other properly on the benefits of circumcision but I think this is the way to since it’s like killing two birds with one stone whereby you protect your spouse from cervical cancer and also you are able to know your HIV status.*
21. I: What do you think should be done to deal with the barriers or concerns you have mentioned?
22. *R: I think people should really be encouraging about couple HIV testing because sometimes it becomes hard for man to explain convincingly to a woman the essence of circumcision as it is in the villages most women would start to think that if a man goes for circumcision it is because they are no longer interested in having sex with them no knowing it is good in that it protects them from cancer and the man gets protected too.*
23. I: Okay fine. Now I would like us to discuss about sexual reproductive health services, pills for HIV prevention called pre-Exposure Prophylaxis. sexual reproductive health include services that promote good sexual health and reproduction they include but not limited to family planning, cervical cancer screening, Sexually Transmitted Infections management and condom distribution and many more. Today we will only discuss about family planning, cervical cancer screening, Sexually Transmitted Infections management and PrEP. We will look into each one of these one by one, so let’s start with STI services. Explain to me what happens when a person is being suspected or has been diagnosed with STIs?
24. *R: They receive counselling of how they can take care of themselves. For instance if you have been diagnosed of gonorrhea or syphilis, they first assist you on the infection you have been diagnosed with then they circumcise you right away*
25. I: Do they do the circumcision if diagnosed with the infections or they first send you back to come again after recovery.
26. *R: But the way I noticed, they circumcise you right away but they also give you treatment for the infection you have been diagnosed with.*
27. I: So as health service provider support personnel, what is your opinion about the integration of STI screening and management within the circumcision clinic?
28. *R: I think it was important to have a special branch to assist with this STI management so that if these infections persist you can still be assisted*
29. I: So what issues do you agree with in this integration?
30. *R: Like I have said, taking us both as a couple HIV testing and if diagnosed with gonorrhea or syphilis we need to receive the counselling as a family like what used to happen in the past if I receive the medication on my own I will take the drugs as instructed but still my wife is still infected and also if the woman comes to the clinic with men they may get the chance of being screened of cancer at this moment so I think this will be a very good move.*
31. I: What things don’t you agree with the integration of STI screening and management within the circumcision clinics?
32. *R: Ah to me I don’t think there is something I don’t like about it*
33. I*:* In terms of place and time, how do you think this service can be offered at the VMMC clinic?
34. *R*: *In most village clinics which are very far away so it is better to have small branches in these areas so that people can be getting counselling close by to relieve people from walking long distance for this service to enable people access if just information on how circumcision works and enable people make decisions.*
35. I: So in clinics like this one, where exactly should this service be offered? Outside, inside the hospital or in some private space?
36. *R*: *I don’t think this issues should be a private matter for instance if you have come with your relations maybe but I think the best way is to have a special clinic so that whenever you are coming for circumcision everyone should be able to know that you are going for circumcision since you cannot stand on a queue to receive very different services.*
37. I: What do you think can be the barriers and concerns to this integration?
38. *R: Ah….to my side I think there are no concerns.*
39. I: Now let us talk about family planning. Explain to me what happens when a person has come to seek family planning services like vasectomy and tube legation?
40. *R: What happens is that, let me talk about women family planning services, for men we receive the news about this truly but for men I have never had time to understand how it works but I heard for men it weakens your sexual power so women may run away from you but for women after family planning service men still have the same sexual feeling as before*.
41. I: No you as health service provider support personnel, what do you think about the integration of family planning within the VMMC clinics?
42. *R: Mm… circumcision and family planning are two different things, I know circumcision is for people with 10 years and above but family planning maybe 25 years above based on what the couple has agreed so I see there is no concern because it is about the reproductive person and circumcision is for a person old enough to manage the wound so as I said we need a special clinic that can offer these services separately and the counselling is offered differently which can’t be provided anyhow. So you need to divide the clinic into circumcision room, family planning room since these services cannot be provided in the same room.*
43. I: But do you like this integration*?*
44. *R: Yes, I have liked it a lot because it has brought happiness in a family.*
45. I: How let us talk about cervical cancer screening. Explain to me, what happens here when a woman wants to do cervical cancer screening? Do they do cancer screening at circumcision clinics?
46. *R: Ah because I don’t know that women go but women don’t go to the circumcision so they have not started offering that service but if they are to start women may only get the chance of getting screened.*
47. I: What can make you happy most about this integration that women can be screened of cervical cancer?
48. *R: As I said already, when you put in place this service, the family will be as new because I can come for circumcision but I can never know whether she already has cervical cancer or not because if you are to put in place even the man can be screened for this cancer because it would allow the woman to know her status already since you not know if you have already infected her with the cancer*.
49. I: What can make you not to want this integration?
50. *R: Ah nothing.*
51. I: How do you think this service of cervical cancer screening should be provided in terms of time and place?
52. *R: In terms of place as I have already said a clinic like this should just be divided in different blocks to offers these distinct services.*
53. I: What do you think can be the barriers or concerns to this integration?
54. *R: Maybe if you don’t understand in the house because maybe you are stressed that you will be infected with HIV*
55. I: So what do you think can be done to deal with those concerns?
56. *R: They need to be provided with information, like I said about establishing the small local branches to help in providing information so that they make a choice but as of now because there are no clinics in the villages they come with the mobile vehicles that passes the information they may not get all the information and very few people like me came for circumcision so as of now my family is very good though I don’t know if my wife hasn’t yet been infected with the cancer since she has never been screened.*
57. I: Now let us talk about PrEP. Have you ever heard PrEP?
58. *R: No.*
59. I: So I will explain to you how it works. It is an abbreviation that means Pre-Exposure Prophylaxis. *It is a pill that is taken by an HIV negative person so that it should protect the person from contracting HIV even if this person has sexual intercourse with an HIV positive person. If taken properly it is effective in protecting you fully from contracting HIV. Now how do you understand about PrEP??*
60. *R: It protects a person from the virus?*
61. I: Yes, it is. For example if your wife is HIV positive and your negative so you want to be having sex, it is still possible to be doing so if you are taking PrEP properly. So how do you understand about PrEP?
62. *R: By PrEP you mean you can have without condom without contracting?*
63. I: Yes. PEP do you know PEP? It is taken after sexual intercourse within 72 hours without contracting the virus while PrEP it is taken before sexual intercourse and if taken properly you cannot contract the virus. So do you think it is necessary to make PrEP available to HIV negative men and women?
64. *R: Because being infected of any disease is not necessarily that you have been having immoral sexual activities so yeah these pills are good.*
65. I: What reasons do you have to encourage people to be taking PrEP?
66. *R: PrEP as I have already said not everyone gets infected through sex others are infected at birth so it may help to reduce divorce rates because of HIV and also it will allow the other person to remain healthy so as to take care of the kids if the other is dead.*
67. I: So if PrEP is to be made available, what is your opinion on integrating it within the VMMC clinics?
68. *R: But this PrEP can it not facilitate the act of unprotected sexual intercourse?*
69. I: That is your concern right?
70. *R: Yes*
71. I: Well, we will come to that later on sure, but was about to ask the barriers concerns so you have already answered the next question. So how do you think that concern can be addressed?
72. *R: That concern needs people to be taught because people can be doing it deliberately because taking the drugs because the other partner is infected is different from taking the pill and into a bar so it may promote unprotected sexual intercourse.*
73. I: Yes, it is possible, so what do you think can be done?
74. *R: I think it is important to teach people and we should not reach to the point of saying you cannot be protected by maybe we should be using the word as in condoms that it is not 100 percent so that people should not afraid at least we tell people it protects only 60 percent chance but it is not supposed to be discouraged because doing so will deprive other the chance to protect themselves if their spouse if infected.*
75. I: But do you like this integration?
76. *R: Yes, it is a good one because if we are to discourage we will deprive the majority their right to access the pill.*
77. I: So how do you think these drugs can be provided at a circumcision clinic?
78. *R: These drugs what is important as I have already said, there is need to have different rooms and it should be indicated on the door and this can be within the family planning but to a person who is infected but has no wife I think this process is not necessary since they merely decide to go and have sex but this PrEP is ideal for married people.*
79. I: So let us look at other services. If you are given the power to integrate other services to the VMMC services, what are the services you would think of integrating?
80. *R: I can choose to integrate; you mean in the hospital like how a person should be going through?*
81. I: Yes, in the circumcision clinics, if you have been given the power to choose the services to add, what other services would you add?
82. *R: Mm... eeehhh because throughout the discussion seems like you went deeper but my thinking is that because when we say this is a woman it starts at birth and cancer does not affect only those that are married or does it attack only the married ones?*
83. I: No it doesn’t.
84. *R: So I was thinking maybe if you can put a service where even small ladies can be tested before marriage so that at the point at which they be getting married they should be aware that they are free from the cancer. Because I have seen here that there is nothing about these girls here so that in the room that can be used to screen women in that same room these ladies can be helped in that same room at a certain age.*
85. I: But for the services we have already discussed like PrEP, STI screening and management for instance, what services would you choose to integrate in the circumcision clinics?
86. *R: Yes, but as I have said they need to have one place, most of these am interested with so I would say all of the issues we have discussed are very important.*
87. I: Thank you so much for taking your time to discuss these things today, your answers will be helpful in improving the health service delivery at circumcision clinics. Before we close, do you have anything to say?
88. *R: Ah what I can say about circumcision is to say thank you because ever since I lived even at our school they never come to talk about circumcision. All I ever know was those harmful traditional circumcision but this service previously we thought it is very painful but this is medical different from the traditional ones since it allows people to be doing their normal duties but this is helpful to myself.*
89. I: Thank you so much.
90. *R: Indeed.*

END.
