# Supplementary material for: Opinions on integrating couple counselling and female sexual reproductive health services into Voluntary Medical Male Circumcision services in Lilongwe, Malawi
Source: PLoS One. 2022 Sep 9;17(9):e0273627. doi: 10.1371/journal.pone.0273627 (PMC9462804; doi:10.1371/journal.pone.0273627)
Supplement: S3 File — (DOCX) [file pone.0273627.s003.docx]

**D 43 STUDY**

**Date of Interview: 26 June 2018**

**Type of Participant: Male Index Participant**

**Interview Number: D-43-0003**

**Interviewer: C. L**

**Total Interview Time: 36 minutes 31 seconds**

**Interview Summary:** **(from summary sheet)**

| **SERVICE TO BE INTERGRATED** | **THOUGHTS ON INTERGRATION** |
| --- | --- |
| Couple HIV Testing and Counseling | Could help prevent STIs because couples could agree to stay negative if they test together. |
| STI Services | Would help couples to be cautious not to get STIs. Thinks the screening room should be the same which is already there. |
| Family Planning | Did not have prior knowledge of family planning but still said clients who have done VMMC usually want unprotected sex so this integration would help their partners to get family planning and the men would not be looking for sex elsewhere. Service should be given to those that have been screened and have gotten VMMC. |
| Cervical Cancer Screening | Thought cervical cancer attacks both men and women! Thinks the integration is a good idea only that couples need to come together to the VMMC clinic. |
| PrEP | Indifferent about the integration of PrEP into VMMC service. |
| Other Services | Thinks cervical cancer screening and PrEP are the best to be integrated into VMMC |

**Remarks:**

**Participant was relaxed, quick to ask where he was unsure but did not have prior knowledge of family planning, PrEP and cervical cancer. He however freely expressed his opinion.**

**Interview Text:**

1. I: Thank you for taking the time to talk with me today. I would like to ask you some questions today about the way you feel and what you think about some issues related to the service you receive here and how we can include other services in Voluntary Medical Male Circumcision clinics. There are no right or wrong answers to these questions. We would like to hear your opinion and your experiences in your own words. Do you have any questions before we begin?
2. *R: So it’s all about our opinion?*
3. I: Yes, it’s all about your opinion. You will only say what you think.
4. *R: Ok.*
5. I: Sure. So the first question is; Do your ever talk to your care providers about how the services are provided here?
6. *R: Yes, we do talk.*
7. I: You do talk?
8. *R: Yes.*
9. I: Can you give me an example of what you talked when you came?
10. *R: Well, the first day that I came here was on Saturday. I was late, and they told me that I was late too. So they advised that I should come on Monday in the morning. But the same Saturday, they called me and explained to me how their processes take place. You know, previously people were intimidating us, so I took it as an opportunity to ask them how the whole process takes place. So they started explaining to me that “Male circumcision is a simple process. Firstly, we conduct an HIV test to know your status…” and then they also told me that when one has sexually transmitted infections such us gonorrhea, syphilis and the like, they don’t circumcise you, they rather give you the medicine to cure you, and after you are cured, they then circumcise you. That was just ok with me. I then came on Monday, and they conducted the tests, and they tested me for BP, Diabetes, gonorrhea, and the like, and then they told me that there was one last process to take place – circumcision. So they circumcised me.*
11. I: Ok.
12. *R: Sure.*
13. I: Alright, so I want us to talk about partner HIV testing here at the circumcision clinic. The first question is: have you ever brought your partner here at the circumcision clinic?
14. *R: No, I have never brought her here.*
15. I: No?
16. *R: No.*
17. I: Why did you not bring her here?
18. *R: I never thought about it.*
19. I: You never thought about it?
20. *R: No.*
21. I: Did they explain to you what happens when a man brings a partner at the Voluntary Medical Male circumcision clinic?
22. *R: No, they did not explain anything, and I did not ask them about it either.*
23. I: Ok.
24. *R: Yes.*
25. I: Or did you see anyone who brought a partner at the circumcision clinic?
26. *R: No, I saw no one.*
27. I: Ok. So why do you think is the reason men do not bring their partners at the Voluntary Medical Male circumcision clinic?
28. *R: I think they become nervous. The thing is, they can come with their partner but they fear a situation where they are tested HIV positive while the Partner is negative.*
29. I: Ok.
30. *R: Yes, it’s all about being afraid and ashamed.*
31. I: Ok.
32. *R: Yes.*
33. I: But how do you personally view that situation where you can bring a loved one when being tested for HIV?
34. *R: That is very important because firstly, for someone like me, I am very confident that I am negative, and it can be good if she as well can come here so that I can as well know her status.*
35. I: Ok.
36. *R: Yes.*
37. I: Alright, so what do you think can be done to make men bring their partners here for HIV testing and counselling?
38. *R: I think there should be civic education, they need to be taught about the advantages of going there with their partners because for someone like me, I never heard that we can come with our partners.*
39. I: You had never heard that you can come together?
40. *R: No, if I had heard, I could have come with her. There was no one to guide me in particular, I just came on my own for VMMC.*
41. I: Ok.
42. *R: Yes.*
43. I: Ok.
44. *R: Sure. But if they were travelling in different locations, teaching on how it is done, it could have been much better.*
45. I: Ok, you as Individual, what is your opinion on integrating couple HIV counseling with Voluntary Medical Male circumcision services?
46. *R: What do you mean?*
47. I: I mean when you come here, you need to come with your partner for HIV Testing, what is your opinion?
48. *R: It can help to prevent sexually transmitted infections because, let’s say, for example, if I come here for HIV testing with my partner and I have been tested negative, and she has also been tested negative as well, we can agree on one thing because both of us can be tested negative and there cannot be any need for us to go elsewhere looking for another partner because going elsewhere can lead to the contracting of sexually transmitted infections. So yes, it can help to prevent sexually transmitted infections.*
49. I: Ok. So do you think there are barriers and concerns on this integration?
50. *R: Barriers to the clients? Or…?*
51. I: Yes, to the clients, as well as to the providers. What can be the barriers to this integration?
52. *R: The barriers?*
53. I: Yes.
54. *R: I think the barriers can be, to those who work, lack of proper education to the people, and this can prevent the progress of the development, say, couples to go together at VMMC. But for the clients, the barrier can be in form of fear. If one has gone with a wife, for example, and has been tested HIV positive, and let’s assume I don’t know my status and I am not circumcise either, aware that a fellow was not circumcised when he was tested positive, and of course, while with his partner, I cannot go there with my partner as well, so that I should hear it first for myself.*

I&R: [Laughter]

1. *R: Because if you go together in such issues, even married people can get divorced because of such things. So to prevent things like divorces, men will opt to go alone. But if there can be civic education to sensitize people in such cases, it cannot be a hard thing.*
2. I; Mhhh. So it is because one can be afraid of divorce.
3. *R: Yes, fears of divorce.*
4. I: So what do you think we need to do to eliminate such worries?
5. *R: To eliminate?*
6. I: Yes.
7. *R: Its al about what I earlier own said – civic education only.*
8. I: Mh...
9. *R: There are some people who have never gone to school, and they are not aware of all the procedures that take place here, and you need to explain to them patiently on how it goes. Even those who stay in remote areas do not really know and understand about VMMC. Even myself, when I was coming here for VMMC, I learnt a lot of things. I was earlier on afraid. People were intimidating us. “You will be failing to walk!” “a circumcised man wears a chitenje (Wrapper cloth)!” “The circumcision wound can lead you to death…”*
10. I: mmmmm.
11. *R: I could have gone for VMMC already back then, but I was afraid. Of course there was a time when I heard on the radio about VMMC and I was inspired. And there was also this other time when there came a VMMC vehicle and I had an opportunity to ask some questions, and that is why I had made up my mind to come here for VMMC.*
12. I: Ok.
13. *R: Sure.*
14. I: Now I would like us to discuss about sexual reproductive health services and Pills for HIV prevention: called pre-exposure prophylaxis. (PrEP)
15. *R: PEP?*
16. I: PrEP. There is an “R”
17. *R: PrEP.*
18. I: Yes.
19. *R: Pills?*
20. I: Yes.
21. *R: They protect?*
22. I: Yes. They protect from HIV. I will explain to you in details later during our conversation. Sexual reproductive health includes services that promote good sexual health and reproduction. They include but not limited to family planning, cervical cancer screening sexual transmitted infection management cervical, condom distribution and many more. Today we will only discuss family planning, Sexual transmitted infection management cervical screening and PrEP. We will look at each of these one by one. Let us start with: screening and sexual transmitted infections. Explain to me, what you have ever learnt about STIs when you came to Voluntary Medical Male circumcision clinic?
23. *R: What have you said?*
24. I: Explain to me, what you have ever learnt about STIs when you came to Voluntary Medical Male circumcision clinic?
25. *R: Disadvantages, or what?*
26. I: Anything that they explained.
27. *R: Ok.*
28. I:Mm...
29. *R: Ok, firstly, I will talk about the disadvantages of VMMC….I rather mean STIs…*
30. I: …. STIs…
31. *R: The disadvantages are: (1) You can die. (2) You don’t look healthy and you cannot do a heavy work.*
32. I: Ok.
33. *R: Sure.*
34. I: What did they tell you would happen in case they suspect or actually test you positive for STIs?
35. *R: They told me that in that case, I will not do the VMMC.*
36. I: So did they test you for STIs?
37. *R: Yes, they did.*
38. I: So what is your opinion on integrating STI services with Voluntary Medical Male Circumcision services, so that both of you should be coming here so that the process can take place while you are together with your partner?
39. *R: As in like going together with her?*
40. I: Yes… [Laughter by both interviewer and respondent]
41. I: Screening this time happened to you only, but in case there is a programme to be screening both of you together for STIs while you are with your partner?
42. *R: That can be a good idea.*
43. I: Why?
44. *R: The fact that you will both come together can help that you should together prevent the STIs because in case both of you are negative as regards cervical cancer, or such STIs like gonorrhea, it can be good because even if you are not together, you will be cautious since you will know that you are negative and you would not want to do anything that can bring the STIs.*
45. I: How do you think is the best way to offer STI services at the Voluntary Medical Male Circumcision clinics?
46. *R: The best way?*
47. I: Yes. The best way to screen for STIs, as someone who has gone through all the processes, where do you think is the best way to screen for STIs in the VMMC process?
48. *R: I think the same places where they screen are already good.*
49. I: They are good?
50. *R: Yes. Because you need to qualify at such places if you are to be circumcised. VMMC is the last step in the process if you are tested negative in the other processes.*
51. I: Ok. So who do you think should be tested for STIs?
52. *R: Both.*
53. I: Both?
54. *R: Yes.*
55. I: Ok…
56. *R: Yes, because it will be kind of a couple.*
57. I: So should they screen anyone who comes, or how should it be like?
58. *R: They should be screening anyone who comes here to be assisted.*
59. I: What is it that you do not like integrating STI services in Voluntary Medical Male circumcision services?
60. *R: What?*
61. I: What is it that you do not like integrating STI services in Voluntary Medical Male circumcision services? Or is there anything that you did not like when they tested you? Assuming you were with your partner?
62. *R: Nothing.*
63. I: There is nothing that you do not like?
64. *R: Nothing. In fact, I liked it so much because at first, I thought that at VMMC, only HIV is screened, but I was amazed to see that I was also tested for Diabetes and BP and the like, and I was so happy because I knew my status as regards those diseases because I lack time to go screening for such diseases. We mostly come for such tests when it is already too late. For example, when I came, there was a man who was in front of me, and when he went for HIV screening, he fainted because his BP shot. They took off his shirt and started cooling him, imagine! Then when he was back to his senses, they brought him up here to the ward, and they gave him some medicine. So, when he had been given medicine, he went back to the VMMC, but they did not allow him to do the VMMC, they told him that such diseases prevent one from getting the VMMC.*
65. I: Mmmm.
66. *R: Yes, so to me, I noted that it was good they test us for such diseases, because that man could not have known about his status if he had not come here. So even though he was not circumcised, it helped him to know that he has a BP problem, something that he would not have otherwise known if he had not come for VMMC. And since they gave him medicine, it can help him to recover from that problem.*
67. I: Ok.
68. *R: Yes.*
69. I: What do you think are the barriers and concerns on this integration of STI services with Voluntary Medical Male circumcision services?
70. *R: Fear.*
71. I: Fear?
72. *R: Yes. They fear what will happen to their partners if they discover that they are positive.*
73. I: What can we do to eliminate such fears?
74. *R: My point still stands: Through civic education. People should be fully taught on the advantages of being screened and also the disadvantages of just be staying at home without knowing their status.*
75. I: Alright. So, let us now talk about family planning; Explain to me about anything that you know about family.
76. *R: Well, I don’t know much about family planning. I actually don’t know about what actually happens*
77. I: Family planning is all about methods that men and women do to prevent pregnancy, to prevent having a child. So a woman can do family planning, and a man can as well do family planning. It is possible for men to do family planning through vasectomy. There are also several methods for women such as DEPO, Loop, Norplant, condoms, pills. So individually, …….
78. *R: Wait a minute, does it mean that if you are doing family planning, you will never have a child?*
79. I: No.
80. *R: You can have children later on?*
81. I: Yes.
82. *R: It just helps at that particular time, that the woman should not get pregnant?*
83. I: Yes. But it also depends on the family planning method that you are using. Some methods are for longer periods, while others are for shorter periods. For example, pills are for a short time, but Depo helps for 3 months. The other family methods can be for years, up to 5 years. So, as an individual, what is your opinion on integrating Family planning in Voluntary Medical Male circumcision services/clinic?
84. *R: It can be very good because there are some people who when they do VMMC……they…they…. anyway, I will say it…*
85. I: Yes, you can say it its ok…
86. *R:. there are some people who when they go for VMMC, they think that they can just be having unsafe sex without a problem. There are cases when a person doesn’t want pregnancy, and then the partner wants to have unsafe sex. If you deny your partner of having unsafe sex, he may go elsewhere to outsource, say to a prostitute and pay her off after having sex. So, I would prefer that they should be told about family planning because it can then help.*
87. I: Alright. So, what is it that you would like about integrating Family Planning services in Voluntary Medical Male Circumcision?
88. *R: What?*
89. I: What is it that you would like about integrating Family Planning services in Voluntary Medical Male Circumcision? So because Family planning is offered in other departments, but not yet at the VMMC Clinic.
90. *R: I can like the fact that I can be enjoying with my partner, it will help because I will not be outsourcing because my partner has refused.*
91. I: Ok!
92. *R: Yes.*
93. I: How happy would you be to come and access the services?
94. *R: Yes, I can be very happy to access such a service.*
95. I: And what would make you not want integrating Family Planning services in Voluntary Medical Male Circumcision services? We are discussing about VMMC Clinic here.
96. *R: Yes.*
97. I: So what would make you not want integrating Family Planning services in Voluntary Medical Male Circumcision services?
98. *R: But to me, I think there is nothing that can let me down because I can be accessing family planning services.*
99. I: Ok. How do you think is the best way to offer family planning services within Voluntary Medical Male circumcision services clinics?
100. *R: I think it should be given to those who have been screened, and have of course been circumcised.*
101. I: But not everyone?
102. *R: No!*
103. I: Alright. What do you think could be the barriers or concerns on family planning and Voluntary Medical Male circumcision integration?
104. *R: I think people can have fears that they will never have children forever.*
105. I: How do you think these concerns and barriers be addressed?
106. *R: I will go back to what I already mentioned at some point in time….*
107. I: That’s ok.

[ALL LAUGHING]

1. *R: Civic Education.*
2. I: Ok. Let’s now talk about cervical cancer. Have you ever heard about cervical cancer screening?
3. *R: Yes, I have ever heard, but not in details as to the processes and what actually happens, no, I just know it by name.*
4. I: It really is a disease that affects women and in some clinics, they do the screening to find out if a woman has cervical cancer. As an individual, what is your opinion on integrating partner cervical cancer screening with Voluntary Medical Male circumcision services? Say, you come with your partner and she is screened for cancer?
5. *R: It can really help. In fact, last time I came, they said that they do screening for women. But I suppose they meant to us men, but in case we have gone at the VMMC Clinic with our partners, and both of us are screened, I think it can do us good, and it can also help us to be taking care of ourselves.*
6. I: What is it that would make you not want integrating cervical cancer screening with Voluntary Medical Male Circumcision?
7. *R: There is nothing that can let me down.*
8. I: Nothing?
9. *R: Nothing.*
10. I: Ok, so how do you think is the best way to offer Cervical Cancer screening services within Voluntary Medical Male circumcision clinics?
11. *R: To those who come for VMMC?*
12. I: Yes.
13. *R: I think it can be good if this service can be offered in the ward that they do VMMC, to help them know their status. They screen us before we are circumcised, and I remember I was screened for something like that.*
14. I: Ok.
15. *R: Yes.*
16. I: So before they are circumcised?
17. *R: Yes.*
18. I: Because only women are screened for cervical cancer…
19. *R: Only women?*
20. I: Only women.
21. *R: Not men?*
22. I: No.
23. *R: So you mean its only women who can suffer from cancer?*
24. I: Cervical cancer is for women only. There are different types of cancer, but cervical is for women only. Do men have the uterus?
25. *R: No.*

*[ALL LAUGHING]*

1. I: They don’t have. Its only women. Now, I want to ask you, since you said that you are screened of different things when you come here for VMMC, so I want to know, should the woman come for screening while the man is there?
2. *R: They are supposed to be together. It’s difficult to trust a person nowadays. One can be tested positive, but might not disclose it to the partner, and it can also happen that someone can tell you that she is negative and you might just answer “ok” but you can doubt because you will always say that I wasn’t there when she was been screened. But if you are together, you can accept the results.*
3. I: So what class of women do you think should be screened? Every woman or you have a preferred age range or a specific type of women?
4. *R: Every woman.*
5. I: Every woman?
6. *R: Yes, every woman should be screened.*
7. I: Ok. What do you think could be the barriers or concerns with partner cervical cancer screening and Voluntary Medical Male circumcision integration?
8. *R: Mmhhh*
9. I: Or rather I should say the barriers that can prevent men from bringing their partners at the VMMC Clinic?
10. *R: No, there isn’t.*
11. I: There isn’t?
12. *R: No.*
13. I: NOW Let us discus about PrEP. Have you heard about PrEP?
14. *R: No.*
15. I: I will explain it to you. PrEP are medicines that help to prevent HIV, they help in sense that a person who is HIV negative should not contract HIV. So they are medicines that when you take, you do not contract HIV. The dose is, a person swallows one pill per day. One pill per day, daily. And if you are following the instructions, this pill works very effectively in helping people prevent from getting HIV.
16. *R: For the rest of your life?*
17. I: Yes.
18. *R: You will be HIV negative?*

*[BOTH LAUGHING]*

1. I: You will be taking for the rest of your life if you are HIV negative and the whole aim is to help you prevent from getting HIV.
2. *R: So you can have intercourse with a person who is HIV positive, but you cannot contract?*
3. I: Yes, on a condition that you are following the instructions properly.
4. *R: Can it be possible that you can take as you are going to have sex with a partner?*
5. I: No, you need to be taking like every day, that’s then procedure. But not only when you want to have sex with a woman, no.
6. *R: [LAUGHING]*
7. I: Now, how do you feel about Prep
8. *R: It has advantages and disadvantages. The advantage will be that it can help a person not to get infected. But the main disadvantage is that you can have that mentality of being protected and as a result, you can just be having sex with several people. In case you are unlucky, and you are infected, you can start blaming those who gave you the medicines. You can tell them that you lied to me. And also being a person, it is possible for one to miss a day without taking the medicines, or you can be taking, but not procedural, and you can tell someone you want to have sex with that you do take the medicines and you cannot be infected, and yet you can end up being infected and this can affect one’s life.*
9. I: Ok. So what are your concerns as regards the availability of these medicines?
10. *R: My concern is that the availability of these medicines can result in the increase in the spreading of HIV/AIDS. Am saying that because it is not everyone who can manage to follow such instructions. To give an example, there are some people who drink beer. If those people are drunk, they can easily forget the dose. They can even completely forget to take the medicines.*
11. I: But you also at one point said that in case these medicines are found, it can as well be good….
12. *R: Of course yes.*
13. I: So people should be taking?
14. *R: Yes.*
15. I: If PrEP becomes available, what is your opinion on integrating PrEP with Voluntary Medical Male circumcision services?
16. *R: Come again?*
17. I: If PrEP becomes available, what is your opinion on integrating PrEP with Voluntary Medical Male circumcision services?
18. *R: My opinion is that…since when we go for VMMC, we are screened of every infection, and there is proof that we are negative, taking medicines like PrEP can help in that you cannot get infected with HIV/AIDS.*
19. I: How happy would you be to access them?
20. *R: I can be happy to have access to these medicines.*
21. I: Why?
22. *R: It’s because it can help me not to get infected.*
23. I: Ok. How do you think PrEP would be offered in this clinic?
24. *R: But they should not just be offered anyhow, but maybe only to those who have undergone VMMC because they know that they are all HIV negative.*
25. I: Mmm.
26. *R: So the medicines can help prevent the infections. It can be hard for them if they are offered to someone who already is HIV positive and is on ARV treatment. They can stop taking ARVs and start thinking that these medicines are better that ARVs, and that can pose a risk.*
27. I: Ok.
28. *R: Yes.*
29. I: You also mentioned that there can be worries if people are taking PrEP in that they can become careless. What do you think needs to be done to stop that in case it is happening?
30. *R: That is difficult.*
31. I: It is difficult?
32. *R: Yes, because there are some people who are slow to understand unless you teach them much often.*
33. I: Alright. If you were given powers to choose and integrate services in Voluntary Medical Male Clinics, what are the services that you would think of Integrating?
34. *R: On what we have discussed?*
35. I: Yes, on what we have discussed, but if there are other ways that can be done that we have not discussed here, you can equally mention them.
36. *R: Ways for these processes to take place……*
37. I: Yes…
38. *R: I think there should be a lot of working staff. Some should be spreading VMMC messages; some should be spreading about the disadvantages of not going for VMMC, or concerning cervical cancer, yes.*
39. I: Ok, but the objective of this question was; since we are discussing about the services that are being offered at the VMMC Clinic, but there are some services that are not offered at VMMC such as cervical cancer screening, offering PrEP that we discussed, screening of STIs while you are with your partner, HIV testing with your partner, and some other services that we have not even discussed here. Now, in case you have been given powers to choose a service that you think can best suit VMMC, what sort of services can you choose?
40. *R: Cervical cancer screening – I can make sure that it is there.*
41. I: Mmm.
42. *R: I can also see to it that PrEP is also there.*
43. I: Ok, so why have you chosen cervical cancer screening?
44. *R: Cervical cancer screening because before we are circumcised, there is a 90% chance that we can harbor the cancer viruses, so, when they are screening for cancer, it can help to know my status.*
45. I: Ok, and why have you chosen PrEP?
46. *R: I have chosen PrEP because since they screen us first at the VMMC, and then they circumcise us later on after they see that we are HIV negative, it can therefore help me not to get STIs.*
47. I: Alright. In case you were given powers to choose and integrate services in Voluntary Medical Male Clinics, how do you think PrEP and cervical cancer screening should be offered in the clinic?
48. *R: They should be offered to those people who have been tested negative, like those people who do come for VMMC for the first time.*
49. I: Thank you for taking your time to discuss with me today. Your answers will be very helpful in improving the health service delivery at circumcision clinics. Before we close, do you have anything to say?
50. *R: A few questions, though. The first question is: PrEP medicines, since you said that one needs to take for the rest of his life, don’t they have some negative effects?*
51. I: About that, if we can ask our colleagues the doctors, they can be in a position to answer us. [Laughter]
52. *R: Because it can be possible that even though it seems to be good, it can have some negative impacts.*
53. I: Yes, you are really right. So we will ask them and see what they are going to tell us.
54. *R: Secondly, the disadvantages of family planning – you mentioned about the use of the pills. What are the effects?*
55. I: That question as well will require the doctors to answer us.
56. *R: Ok.*
57. I: Sure.
58. *R: Those were my only questions.*
59. I: I really appreciate.
60. *R: Thank you.*
61. I: Yeah.

END

**D 43 STUDY**

**Date of Interview: 27 June 2018**

**Type of Participant: Male Index Participant**

**Interview Number: D-43-0004**

**Interviewer: C.L.**

**Total Interview Time: 27 minutes 35 seconds**

**Interview Summary:** **(from summary sheet)**

| **SERVICE TO BE INTERGRATED** | **THOUGHTS ON INTERGRATION** |
| --- | --- |
| Couple HIV Testing and Counseling | Reasonable because most men and women are unwilling to come to hospital so if one feel that it’s a good thing then he can motivate the partner. |
| STI Services | A good thing because STIs are not good so it’s better to get treatment and follow instructions. |
| Family Planning | A good idea. Set age limit to 25. |
| Cervical Cancer Screening | Good initiative. Women should be going for screening for cervical cancer at the time when men are going for screening for eligibility for VMMC |
| PrEP | A good initiative. Partners will be able to protect each other if there are trust issues between them. |
| Other Services | Thinks PrEP, couple counseling and testing and screening for cervical cancer should be integrated with VMMC. |

**Remarks:**

**Participant didn’t understand most of the questions and when probed he gave short answers. Even the answers that he gave did not make sense most of the times.**

**Interview Text:**

1. I: Thank you for taking the time to talk with me today. I would like to ask you some questions today about the way you feel and what you think about some issues related to the service you receive here and how we can include other services in Voluntary Medical Male Circumcision (VMMC) clinics. There are no right or wrong answers to these questions. We would like to hear your opinion and your experiences in your own words. Do you have any questions before we begin?
2. *R: Ah… there is no question*
3. I: Okay. Do your ever talk to your care providers about how the services are provided here?
4. *R: We could talk because some people do not want to go for circumcision and we have known the good thing about circumcision like reducing the chances of getting STIs.*
5. I: Can you give me an example of a time when you managed to talk to your health care provider about the services you received here? Did you ever ask questions?
6. *R: Yes, we could ask questions for instance, could a person who have been circumcised not get diseases? So they told me that, a person who has been circumcised cannot get diseases if he protects himself.*
7. I: Okay, now let us talk about partner HIV testing here at the circumcision clinic? Tell me what happens when a man brings in a spouse at the Voluntary Medical Male circumcision clinic
8. *R: Yes, men are supposed to be aware that he is going to get his blood tested for circumcision since nowadays there are trust issues, we think my partner is being unfaithful. So it is the one way of going together for HIV testing.*
9. I: Have you ever seen a man coming together with his female partner here for circumcision?
10. *R: Yes, I have seen it happening and it happens.*
11. I: How do you think is the partner involved in the services that men received here at the clinic?
12. *R: They want to witness that their husband has really circumcised and secondly they also want to test for HIV.*
13. I: Did you bring your wife the time you came for circumcision?
14. *R: No, I did not… because am not yet married.*
15. I: What do you think about bringing your female partner here at circumcision clinic?
16. *R: First of all, most men do not go with their wives; for example, during pregnancy checkups, because they think that is the duty or role of the women. But for coming together as a couple for circumcision, I think it is a good thing, firstly, for the wife to witness that “my husband has really been circumcised” and it is risky if the men come alone. Secondly, it is because once the women have seen that “my husband has been circumcised which means he is clean. I should also follow his footsteps and be clean.”*
17. I: Do you think many men come with their wives for circumcision?
18. *R: No, very few. But I do not know the real reason why they do not like to come with their wives or female partners. But I can come with my wife if I get married since your wife is like your own property or tool.*
19. I: Okay, you as Individual, what is your opinion on integrating couple HIV counseling with Voluntary Medical Male circumcision services
20. *R: This program, I think it is reasonable because many times men and women are unwilling to go to hospital. This will encourage them to be going to the hospital because if one feels something in his heart that this is important then he will encourage the other to go as well.*
21. I: What do you think should be done so that men bring their partners to couple counseling in Voluntary Medical Male circumcision clinic?
22. *R: We need some campaigns for the awareness in the villages, so that men and women together could take part.*
23. I: What do you think are the barriers and concerns on this integration?
24. *R: I do not think there could be any concerns because everyone has agreed on what needs to be done.*
25. I: I would like us to talk about sexual reproductive health services and Pills for HIV prevention: called pre-exposure prophylaxis. (PrEP). Sexual reproductive health include services that promote good sexual health and reproduction. They include but not limited to family planning, cervical cancer screening sexual transmitted infection management, cervical screening, condom distribution and many more. So, today we will only discuss family planning, Sexual transmitted infection management, cervical screening and PrEP. We will look at each of these one by one. Let us start with STI services. Explain to me, what you have ever learnt about STIs when you came to Voluntary Medical Male circumcision clinic?
26. *R: We learnt that you can protect yourself from STIs through using condoms even if one of you in a relationship is infected, we better be using condoms for protection.*
27. I: So…. what did the staff say would happen if you were found or suspected with STIs... before circumcision
28. *R: Before we get circumcised, they told us to go for blood testing and if we are found infected, they gave us information or advice which we need to follow, like to start treatment right away*
29. I: Now here we want to talk about STIs and not HIV. Think of syphilis and gonorrhea. Before you were circumcised, did they explained to you about STIs?
30. *R: Yes. But they were not explaining to us, what they were going to do to us if we were found infected.*
31. I: Okay…as an individual, what is your opinion on integrating STI services with Voluntary Medical Male Circumcision services?
32. *R: I think it is a good thing because STIs like syphilis and gonorrhea are not good diseases. It is better if we prevent it or if we have been given medicine, we better follow procedure to recover quickly.*
33. I: Fine, how do you think is the best way to offer STI services at the Voluntary Medical Male Circumcision clinics? Where could it be placed, as you know the clinic flow of the VMMC clinic. You know that after this room, you go to that room?
34. *R: Yes, the way I know clinical step for circumcision, you start with screening, then you go to the room for the advice and then you go for circumcision, then you go home. So I think by the time you go for HIV testing that’s when you should be tested for STIs. The doctor should also be testing everyone so that you can be aware about your health status.*
35. I: Okay, what do you think are the barriers and concerns on this integration of STI services with Voluntary Medical Male circumcision services?
36. *R: Some maybe shy, thinking that they will be found with STIs*
37. I: Ehm... what do you think should be done to address these concerns and barriers?
38. *R: educate them, that’s the only way, both good and bad things*
39. I: Let us talk about Family planning. Explain to me about anything that you know about family planning.
40. *R: About family planning, we all know that, it is important in the world but it works for a person who ever had children like two or three children. Some of the family planning I know, include that of pills and stiches. Yea*
41. I: As an individual, what is your opinion on integrating Family planning in Voluntary Medical Male circumcision services/clinic?
42. *R: Ah… that can help us... since you know firstly, the population is increasing and government is encouraging people to practice family planning. If you have two or three children, that should be a limit. You cannot have many children as you cannot be able to pay school fees or even to feed them. But family planning can help. I can be happy to be receiving this treatment at this type of clinic especially after being circumcised and it should be provided to people with the age of 25 and above.*
43. I: okay, what do you think could be the barriers or concerns on family planning and Voluntary Medical Male circumcision integration?
44. *R: I do not think, there could be any barriers or concerns.*
45. I: Okay, let us now talk about cervical cancer screening. Explain to me what you know about cervical cancer screening
46. *R: I just heard about it on a radio but I do not pay that much attention. I can’t even remember what I heard on the radio.*
47. I: Okay fine, it is a disease, cancer which attack cervix. As an individual, what is your opinion on integrating partner cervical cancer screening with Voluntary Medical Male circumcision services?
48. *R: I think it is a good thing because most women suffer with this disease even if she is pregnant she finds it difficult to give birth which lead into giving birth through operation. So it will be better if they can include that. Even If I get married, I can come here with my wife.*
49. I: What is it that would make you not want integrating cervical cancer screening with Voluntary Medical Male Circumcision?
50. *R: Unwillingness and lack of motivation from both men and women to come to the hospital.*
51. I: How do you think is the best way to offer Cervical Cancer screening services within Voluntary Medical Male circumcision clinics?
52. *R: When men are going for screening before they get circumcised, that’s when women can also get screened for cervical cancer.*
53. I: Okay, Alright, but what do you think could be the barriers or concerns with partner cervical cancer screening and Voluntary Medical Male circumcision integration?
54. *R: Some may accept it, while others may not due to lack of motivation.*
55. I: Alright, now let us talk about PrEP. Have you heard about PrEP?
56. *R: Yes, they said that, if you are negative and you have unprotected sex with infected person, and you have taken PrEP, you do not get HIV virus.*
57. I: Hmm... Okay, fine. So what you explained is PEP. PrEP is anti-HIV medicine that keeps HIV-negative people from being infected. There is a single pill that is taken once daily, and if you take it regularly, it is highly effective at prevention people from being infected. While PEP, you take if you have some doubts that you have get a virus.
58. *R: Okay*
59. I: Is it necessary to make PrEP available to HIV men and women?
60. *R: Yes*
61. I: Do you have any concerns if people are taking PrEP?
62. *R: No, there cannot be any concern*
63. I: If PrEP becomes available, what is your opinion on integrating PrEP with Voluntary Medical Male circumcision services? How happy would you be to access them?
64. *R: Yes. Because sometimes there maybe trust issues between a husband and wife. So to protect one another, they need to take it. They can access it at the time of receiving treatment like the way they give HIV drugs. They should be given to both men and women.*
65. I: Okay, what concerns would you have about people taking PrEP?
66. *R: I would not have any concerns.*
67. I: Let us assume, if you were given powers to choose and integrate services in Voluntary Medical Male Clinics, what are the services that you would think of Integrating?
68. *R: That of PrEP, couple HIV testing and cervical cancer.*
69. I: Why have you chosen PrEP?
70. *R: Because some men do not like to get their blood tested and taking drugs after they have found with HIV, it also becomes a problem. So to avoid that PrEP can help people to stay healthy.*
71. I: Okay, why have you chosen cervical cancer?
72. *R: This will help men to be aware of the disease and how they can protect their wives in the process of circumcision.*
73. I: What do you mean by that?
74. *R: They say VMMC reduces the risk of cervical cancer so if men are aware of this then more will come to access VMMC in order to protect their partners.*
75. I: Okay, thank you for taking your time to discuss with me today. Your answers will be very helpful in improving the health service delivery at circumcision clinics. Before we close, do you have anything to say?
76. *R: No*
77. I: Again, thank you so much for taking your time to speak with me.

END

**D 43 STUDY**

**Date of Interview: 27 June 2018**

**Type of Participant: Male Index Participant**

**Interview Number: D-43-0005**

**Interviewer: C.L.**

**Total Interview time: 28 minutes 31 seconds**

**Interview Summary:** **(from summary sheet)**

| **SERVICE TO BE INTERGRATED** | **THOUGHTS ON INTERGRATION** |
| --- | --- |
| Couple HIV Testing and Counseling | Thinks it’s good to bring a partner because she motivates the man. |
| STI Services | Thinks it’s a good idea but some men might fear that their friends would laugh at them if they are diagnosed with an STI. |
| Family Planning | Thinks it’s a good development but wishes if men got injection for family planning just like women. |
| Cervical Cancer Screening | Thinks it’s a good development but men need to talk to their partners about it so that they understand the importance of the screening. |
| PrEP | Would be happy to take PrEP. Thinks it should be offered together with the pain-killers that are offered after VMMC procedure. |
| Other Services | Thinks PrEP and Cervical Cancer are the ones he could choose to integrate with VMMC |

**Remarks:**

**Participant was at ease. He looked very happy when PrEP issue was introduced.**

1. I: So thank you for taking your time for us to talk today. I would like to ask you some questions today about the way you feel and what you think about some issues related to the service you receive here and how we can include other services in Voluntary Medical Male Circumcision (VMMC) clinics.
2. *R: Alright.*
3. I: There is no right or wrong answers to these questions.
4. *R:Mmm*
5. I: We would like to hear your opinion and your experiences in your own words. Do you have any questions before we begin?
6. *R: No there are no questions.*
7. I: There are none?
8. *R: No.*
9. I: Do you ever talk to your care providers about how the services are provided here?
10. *R: Talking to them?*
11. I: Yes about the VMMC service that they provide.
12. *R: Yes we talked with them especially we asked them questions about circumcision.*
13. I: What type of questions did you ask?
14. *R: Firstly, I asked about how they do the circumcision procedure because some people don’t come here since they don’t know how really they cut the foreskin. Like for me I am Lomwe by tribe so I already did traditional circumcision so I wanted to know the difference between the medical one and the traditional. So we asked them how they cut it so they said “ we inject you with an anesthesia then we cut the fore skin” and so on and so forth so we consented to that and they did the procedure.*
15. I: Mmm
16. *R: Sure.*
17. I: So now let’s talk about couple HIV testing and counseling here at VMMC clinic.
18. *R: Okay.*
19. I: Tell me what happens when a man brings a partner here at VMMC for HIV testing and counseling.
20. *R: They do that so that they should know each other’s status because sometimes we don’t trust our partners and sometimes it happens that people are married and one has HIV while the other doesn’t.*
21. I: Mmm
22. *R: Yea so that’s how it is.*
23. I: have you ever seen a man bring his partner here at VMMC clinic?
24. *R: Yes my friend brought his partner and I also brought mine and we got tested.*
25. I: So how was the partner involved in the services that men received here at the clinic?
26. *R: Ah, for me to come to a decision to come here for circumcision it was my partner who encouraged me to do so by saying “You know what, you need to go for medical circumcision”. So we discussed that as a couple and there was a slow problem on my part…*
27. I: Mm…
28. *R: Yea because you know we are young so when I would go out to have sex with other women and I use a condom, I would have boils on the foreskin which showed me that I was allergic to the rubber.*
29. I: Mmm
30. *R:Yea.*
31. I: Okay, so in your opinion, what are your thoughts about bringing in your partners for HIV testing at Voluntary Medical Male circumcision clinic.
32. *R: I was happy because it was my wife who initiated the move that I should do circumcision so I felt that if she was telling me to do it then it means it’s a good thing.*
33. I: Okay, so what are your thoughts about integrating couple counseling and VMMC?
34. *R: I think integrating these two… because we started with testing then when we came here they said they also do HIV testing so we agreed because sometimes you may get tested at one clinic and they tell you that you are negative while at another clinic they tell you that you are positive so I was happy to get tested again because the results may differ.*
35. I: Okay. So if they say there is a special program which integrates VMMC and couple testing and counseling so that men are encouraged to bring their partners here for couple testing, how would you feel about that?
36. *R: It’s a good move.*
37. I: It’s a good move?
38. *R: Yes.*
39. I: Don’t you have any concerns about it?
40. *R: No I don’t.*
41. I: How about concerns from other people?
42. *R: Maybe other people can have concerns because everyone knows how they lead their lives and maybe some may be aware of how reckless they have been with their lives, and sometimes it’s not only through sex that you contract HIV, sometimes it maybe because there was an accident and you were assisting the people without gloves and you had a cut and that way you can contract it. Other times it may be that the parents hid it from you.*
43. I: Okay so what would be the people’s concern?
44. *R: They may think that if they are found to be HIV positive in front of their partner then the marriage can end there so maybe they may not be willing to bring the partners.*
45. I: So how do you think we can deal with such concerns?
46. *R: By sensitizing the people and telling them the benefits of getting tested together and knowing your partner’s status.*
47. I: Okay. Let us now talk about sexual reproductive health services and Pills for HIV prevention: called pre-exposure prophylaxis. (PrEP) Sexual reproductive health include services that promote good sexual health and reproduction. They include but not limited to family planning, cervical cancer screening sexual transmitted infection management cervical, condom distribution and many more. Today we will only discuss family planning, Sexual transmitted infection management cervical screening and PrEP. We will look at each of these one by one. Let us start with Sexually transmitted infection management. Please explain to me what you have ever learnt about STIs when you came to Voluntary Medical Male circumcision clinic?

1. *R: What I have learnt is that just because I have done male medical circumcision then I need to just be having unprotected sex without using on a condom, no. We need to still use a condom.*
2. I: So what did the health care providers say would happen if they suspected or diagnosed you with STIs?
3. *R: Ah, they said we would not be circumcised.*
4. I: Why was that?
5. *R: They said it was because with the infection, the skin would give them problems when suturing, and sometimes there might be some pain and so on.*
6. I: Okay. Were you screened for STIs? Not just testing for HIV but screening for STIs.
7. *R: Ah, s for the screening they did not screen us.*
8. I: Okay so what is your opinion on integrating STI services with Voluntary Medical Male Circumcision services?
9. *R: I really wanted them to conduct all the procedures on me so that I could experience the benefits and be one of the people who would encourage other people out there.*
10. I: So if they integrate you would be glad?
11. *R: So very much.*
12. I: Okay so what is it that you do not like integrating STI services in Voluntary Medical Male circumcision services? We are not only talking about screening clients who come for VMMC but also their partners so they can be screened together. What is it that you would not like about this?
13. *R: I have no concerns about it.*
14. I: Do you think other people would have concerns?
15. *R: Some people may have concerns because other people think that if they are found to have the infections then they would be laughed at by their friends and that is what holds some people from coming here to access such services.*
16. I: Mm... So what do you think we can do to deal with such concerns so that people can still be coming?
17. *R: The way we can deal with this is by sensitizing them and telling them that they should not be afraid of the hospital.*
18. I: Mmm [interruption] Okay now let’s talk about family planning. Tell me anything that you know about family planning, anything that you know.
19. *R: It’s good to use family planning but other women say that some methods like the injectable or the one which is inserted is not good because it has side effects on some women to the point that they get sick frequently.*
20. I: Okay, so what family planning methods do you know apart from the ones which you have mentioned like the one they insert or the injectable?
21. *R: I can say the best family planning method is the injectable.*
22. I: So what do you think is the purpose of family planning?
23. *R: The purpose is for child spacing.*
24. I: Mmm okay. What is your opinion on integrating Family planning in Voluntary Medical Male circumcision services/clinic?
25. *R: The thing is most men leave family planning to women but the good thing can be that they can combine VMMC and family planning so that when men come for circumcision they should also learn about family planning. When it comes to antenatal clinics and other services it’s only the woman who goes there, if you tell the man to accompany the woman to antenatal clinic it’s only a few that really go.*
26. I: Mm, sure. Okay. So if they say family planning methods are integrated with VMMC, not just the message about family planning but the methods should also be available here…
27. *R: Yea that may be true but that depends on your agreement with your partner at home to say “my husband when do you want us to have another child after this one?” so you need to have an agreement because we hear that the family planning methods are different: some can be for up to three years, others four even five and so on. So it depends on your discussion with your wife.*
28. I: What is it that you would like about integrating Family Planning services in Voluntary Medical Male Circumcision?
29. *R: It would be good because it’s not good for us to just be having so many children when it is hard to take care of them. It’s not good for one to be pregnant this year, give birth next year then the other year you give another birth when you are not able to take care of the children. But when you are on a family planning method, you know that you will take care of your current child until he reaches a certain age.*
30. I: So how happy would you be to come and access the services with your partner?
31. *R: I would be very happy.*
32. I: Okay because when we talk about family planning it’s not necessarily just women that can take part in it…
33. *R: I know.[laughing]*
34. I: [laughing] Okay so how do you think is the best way to offer family planning services within Voluntary Medical Male circumcision services clinics?
35. *R: Like here…you know women use methods like…what do you call it? Norplant?*
36. I: Yes
37. *R: Yes, and others get injections right?*
38. I:Yes
39. *R: Yes. O it would also be good for us men to be having the injections as well to say that after an injection the man can have sex with a woman and not make her pregnant for so many years [laughing]*
40. I: [laughing] Oh! Men should also be getting injections?
41. *R: Yes they should also be having those.*
42. I: Okay, so we will see if that is possible. So what would make you not want integrating Family Planning services in Voluntary Medical Male Circumcision services
43. *R: No, on my part nothing.*
44. I: What do you think could be the barriers or concerns on family planning and Voluntary Medical Male circumcision integration?
45. *R: Nothing.*
46. I: Okay alright. Let’s talk about screening for cervical cancer Okay alright. Let’s talk about screening for cervical cancer. What do you know about screening for cervical cancer?
47. *R: Mm, I have never heard about that.*
48. I: you have never heard about it?
49. *R: No.*
50. I: Okay, when they are talking about VMMC what do they say VMMC protects from?
51. *R: We can say it’s those two things: one is infections and two it’s the cervical cancer.*
52. I: Okay that’s true. So medical circumcision reduces the risk of cervical cancer. SO when we talk about cervical cancer it’s a disease or a type of cancer which attacks the cervix of a woman and it kills a lot of people. So at other clinics they screen for this type of cancer and if they find or suspect that she has it they give her some treatment so that it doesn’t progress.
53. *R: Okay.*
54. I: So what is your opinion on integrating partner cervical cancer screening with Voluntary Medical Male circumcision services?
55. *R: It’s true. Like previously they were on a campaign that men should accompany their wives to the labor ward.*
56. I: Mmm
57. *R: So it’s a good development.*
58. I: So how is it connected with the cervical cancer?
59. *R: It’s because this cervical cancer it’s a disease, so we cannot say we as men should not get involved.*
60. I: Mmm
61. *R: So we need to be in agreement with the integration.*
62. I: What would make you not want integrating cervical cancer screening with Voluntary Medical Male Circumcision
63. *R: Ah no. they should do the integration.*
64. I: Okay. So since you have been through the clinic flow at this VMMC clinic, at what time point do you think they can include the screening for cervical cancer? At which point in the clinic flow?
65. *R: At the VMMC clinic?*
66. I: Yes.
67. *R: At the time when they are doing the procedure, when the man is in the theatre.*
68. I: At the time when the man is in the theatre?
69. *R: Yes.*
70. I: Okay so who should be screened?
71. *R: You can bring in your wife so that at the time they are operating on you then she should be screened.*
72. I: Okay, so that when the man is being operated on then the woman should be screened for cervical cancer?
73. *R: Yes yes.*
74. I: What do you think could be the barriers or concerns with partner cervical

cancer screening and Voluntary Medical Male circumcision integration?

1. *R: For me I don’t see any barrier.*
2. I: How about for the women?
3. *R: It might be difficult for some women but if it’s according to your agreement*

*then it should be difficult.*

1. I: Okay you said for some women it would be difficult, what would make it

difficult?

1. *R: There are some women who would think “Why should I go there and do*

*something like that?” You know sometimes women speak too much.*

1. I: Mmm.
2. *R: Sure.*
3. I: So how can we address this barrier for the women so that they can be coming?
4. *R: it’s easy to deal with it. Firstly, there needs to be agreement in the house to say*

“My wife, I want things to be so and so.” So when you go to the clinic it gets easier

since you had already discussed it.

1. I: Okay, so now let us talk about PrEP. Have you ever heard about PrEP?
2. *R: No.*
3. I: Okay then I will explain how the medicine works. PrEP is anti-HIV medicine that keeps HIV-negative people from being infected. There is a single pill that is taken once daily, and if you take it regularly, it is highly effective at prevention people from being infected.
4. *R: [silence]*
5. I: So if you are to tell someone what PrEP is, what would you say?
6. *R: It’s easy to explain to someone because you would have the drugs and show him and tell him “These are drugs that will prevent you from being infected. And the prescription is like this and that”.*
7. I: Mmm so the way I have explained, how many times does one take per day?
8. *R: Three times per day.*
9. I: Okay I said there is a pill…
10. *R: A pill.*
11. I: Yes, a pill you take every day once a day. So if you want to be protected for ten years then you take the pill fir ten years. If you want to be protected for your whole life, then you take it for your whole life every day. But you need to take it regularly. So if this medicine was available do you think it is necessary for this drug to be available to HIV-negative men and women?
12. *R: Yes, it is necessary.*
13. I: Why do you say it’s necessary?
14. *R: It’s because like us men we like to cheat and lie to our wives about it so if we*

*go and have sex out there then we can take the pill and protect ourselves*

1. I: Okay. Do you think there may be any concerns if people are taking these?

drugs?

1. *R: No, there cannot be any concerns.*
2. I: How happy would you be to access them?
3. *R: I would be very happy. I don’t know if my wife would be happy if I talk to her*

*about it.*

1. I: So what is your opinion on integrating PrEP with Voluntary Medical Male

circumcision services?

1. *R: If you are to tell men to say “you need to go to the hospital to do such and such”, it becomes difficult for them because they feel like they have other business to attend to. So at VMMC clinic it’s like it will be easy to find the men there and talk to them about these other services that are also there at the clinic instead of telling them to go and access such a service at a separate clinic because they will feel that maybe they will be late for work or similar excuses. SO it’s a good development to integrate these services.*
2. I: Mmm. So how do you think PrEP would be offered in this clinic?
3. *R: It would be the same clinic flow like they are doing right now because when they are done with the procedure they offer some pain killers to the men. So they can be offering the pills in the same way so that when the men go home they can be taking them.*
4. I: Okay. You mentioned that you do not have any concerns about people accessing PrEP…
5. *R: Mmm.*
6. I: Do you think other people can have concerns if this PrEP is available and given to HIV-negative men and women?
7. *R: Some people may have concerns to say “It’s not good that this drug should be available”*
8. I: Why would they say it’s not good?
9. *R: It’s because they might feel like it’s mandatory.*
10. I: Mmm. So how can we address such a concern?
11. *R: By telling them that there is nothing dangerous or difficult about this.*
12. I: Mmm. Okay. So if you were given powers to choose and integrate services in Voluntary Medical Male Clinics, what are the services that you would think of Integrating?
13. *R: The services that are there are quite understood.*
14. I: They are understood?
15. *R: Yes.*
16. I: So from the ones we have discussed here, which ones would you choose to integrate with VMMC services?
17. *R: The pills which we have talked about.*
18. I: PrEP?
19. *R: Yes PrEP…cervical cancer screening.*
20. I: Why have you chosen cervical cancer screening?
21. *R: It’s because cervical cancer is a painful disease to a woman and if a disease is such fatal, it needs to be prevented.*
22. I: Okay.
23. *R: Sure.*
24. I: How about PrEP, why have you chosen it?
25. *R: It’s because it would help to prevent HIV so that’s why I have chosen it.*
26. I: Alright, thank you for taking your time to talk with me today. Your answers will be very helpful in improving the health service delivery at circumcision clinics. But before we close, do you have anything else to say?
27. *R: No I have nothing else to say.*
28. I: How about a question?
29. *R: No, I have no question either. [laughing]*
30. I: Alright thank you for your time.
31. *R: Alright.*

END

**D 43 STUDY**

**Date of Interview: 27 June 2018**

**Type of Participant: Male Index Participant**

**Interview Number: D-43-0006**

**Interviewer: C. L.**

**Total Interview Time: 22minutes 17 seconds**

**Interview Summary:** **(from summary sheet)**

| **SERVICE TO BE INTERGRATED** | **THOUGHTS ON INTERGRATION** |
| --- | --- |
| Couple HIV Testing and Counseling | Partner acts as a motivation for a man to get VMMC service and this can be an opportunity for HIV testing. |
| STI Services | One can be killing two birds with one stone: get STI services as you go for VMMC service. |
| Family Planning | Good idea. Couples can use the opportunity of going for VMMC to get family planning services. |
| Cervical Cancer Screening | Good thing as one could be discouraged to travel all the way to (Name of Hospital) for screening. |
| PrEP | Good idea. Can protect discordant couples. |
| Other Services | Thinks cervical cancer screening and PrEP are the best to be integrated into VMMC |

**Remarks:**

**Participant was relaxed and showed that he had enough information about VMMC service. He could express his opinions and asked questions where he was unclear.**

**Interview Text:**

1. I: So… thank you for taking the time …to talk with me today. Feel free
2. *R: Thank you.*
3. I: I would like to ask you some questions today about the way you feel and what you think about some issues related to the service you receive here and how we can include other services in Voluntary Medical Male Circumcision (VMMC) clinics. There is no right or wrong answers to these questions. We would like to hear your opinion and your experiences in your own words. Do you have any questions before we begin?
4. *R: There is no question.*
5. I: There is no question? [Laughing…]
6. *R: Yes [Laughing…]*
7. I: so, do you ever talk to your care providers about how the services are provided here?
8. *R: Yes… yes, we could talk*
9. I: You could talk?
10. *R: Yes.*
11. I: Can you give me an example of a time when you managed to talk to your health care provider about the services you received here?
12. *R: We talked that, circumcision is voluntary as you are not forced to do it, you should do it by your own choice or free will. So before you get circumcised, they need to check your health status like HIV, BP, and even diabetes.*
13. I: Did you have a chance to ask them questions?
14. *R: Yes, I could ask them questions.*
15. I: Can you give an examples of questions you asked?
16. *R: The question which I asked was that, what are some of the bad things which can happen in the process of circumcision? And I was given the answer.*
17. I: Okay... Now let us talk about partner HIV testing here at the circumcision clinic, Bwaila right? First of all, tell me what happens when a man brings in a spouse at the Voluntary Medical Male circumcision clinic. Or did you ever seen a man coming with his partner here? Did you ever bring your partner here?
18. *R: Mh… what happens is that, they explain to a person, maybe how to take care of the circumcised wound, because one may forget, and can be reminded by the partner.*
19. I: How is the partner involved in the services that men received here at the clinic? ... Since this is a clinic for male circumcision right?
20. *R: To be exact, they are involved in…. motivating the husband and also to be helping in terms of the services which they have been given from hospital at home.*
21. I: Oh, okay, that’s true. So, did you ever bring your partner?
22. *R: Ehm, yes I did brought her before*
23. I: What motivated you to bring her in?
24. *R: I thought bringing her here would help as on my own I could be discouraged in terms of thinking and how to take care of my body but if she come along, she can be encouraging me.*
25. I: Ehm, so for those who do not bring their partners here, what made them not to?
26. *R: Maybe because, men if they don’t bring in their partners, they think partners are of no help*
27. I: In your opinion, what are your thoughts about bringing in your partners for HIV testing at Voluntary Medical Male circumcision clinic?
28. *R: I think it is a good thing, because it could be like an advantage or an opportunity for HIV testing*
29. I: You as Individual, what is your opinion on integrating couple HIV counseling with Voluntary Medical Male circumcision services?
30. *R: I think, it is a good thing, I welcome and accept it well, hundred percent. This is because each couple could know its status, as sometimes in the family, one partner may be unfaithful, maybe one get test his blood while other does not.*
31. I: What do you think are the barriers and concerns on this integration?
32. *R: There maybe concerns in the way that some men may not be willing to come together with their partners for blood test.*
33. I: What do you think should be done to overcome these concerns and barriers to couple counseling in Voluntary Medical Male circumcision clinic?
34. *R: That is where there is a need to sensitize and encourage them. Educate people in groups like it is in communities to be coming as a couple and that will help them to understand.*
35. I: Fine, now I would like us to discuss about sexual reproductive health services and Pills for HIV prevention: called pre-exposure prophylaxis. (PrEP). Sexual reproductive health includes services that promote good sexual health and reproduction. They include but not limited to family planning, cervical cancer screening sexual transmitted infection management cervical, condom distribution and many more. Today we will only discuss family planning, Sexual transmitted infection management cervical screening and PrEP. We will look at each of these one by one. We will start with STI services. So explain to me, what you have ever learnt about STIs when you came to Voluntary Medical Male circumcision clinic?
36. *R: We have learned that, if a person have unprotected sex, you can get STIs like Syphilis and gonorrhea*.
37. I: What did the staff say would happen if you were found or suspected with STIs?
38. *R: At that moment, you cannot be circumcised unless you have received the treatment and you are well, that is when you can be circumcised.*
39. I: So did they screen you for STIs?
40. *R: Yes they did.*
41. I: Okay, as an individual, what is your opinion on integrating STI services with Voluntary Medical Male Circumcision services?
42. *R: I think it could be a very good thing because it could be like killing two birds with one stone as some maybe hiding but as you go for circumcision you can also be screened for STI since some people do not think about STIs.*
43. I: So, how do you think is the best way to offer STI services at the Voluntary Medical Male Circumcision clinics?
44. *R: It should be placed where they do test or screen for diabetes or BP and after that, you should be given medicine for a particular STI diagnosis that you have and then you should come other day for circumcision.*
45. I: Okay, what is it that you would like about the integration of diagnosis and management of STI services in Voluntary Medical Male services?
46. *R: I can be happy about that because I could be knowing my health status and there can be nothing to hide, maybe in terms of shyness which could not be there, because I might have been told everything by then.*
47. I: Okay, what is it that you do not like integrating STI services in Voluntary Medical Male circumcision services?
48. *R: Ah nothing, everything about integration is all good.*
49. I: What concerns or barriers do you think can be there for this integration?
50. *R: No, there can be no barriers or concerns.*
51. I: Alright, let us talk about Family Planning. Explain to me about anything that you know about family planning. What do you know about family planning?
52. *R: Ah, what I know is that, on a family you can choose to have a space for the number of children, maybe because of the school fees, so you may choose to have a small number of children which you can manage to take care of. There are also methods which are used for family planning like the use of pills, condoms and injections, but I do not know the one used for men.*
53. I: Alright, as an individual, what is your opinion on integrating Family planning in Voluntary Medical Male circumcision services/clinic?
54. *R: That is the best idea, as most of people do not think about family planning, but integrating it here, it could be an opportunity for family planning services as you come for circumcision.*
55. I: Okay, so what would make you not want integrating Family Planning services in Voluntary Medical Male Circumcision Services?
56. *R: Ah, maybe time management could be a problem as you know here in town we have other things to be doing so for us to be spending too much time here it would not work for us.*
57. I: How do you think is the best way to offer family planning services within Voluntary Medical Male circumcision services clinics? Who is supposed to receive, when, where?
58. *R: This need special place like an office where people can be going after being circumcised.*
59. I: Okay, so what do you think could be the barriers or concerns on family planning and Voluntary Medical Male circumcision integration?
60. *R: Ah… no, there cannot be any concerns, especially for a person who think properly, it is only a good thing.*
61. I: So we will talk about cervical cancer screening... Explain to me what you know about cervical cancer screening
62. *R: Eh… what I know about cancer is that, it is a natural disease and it attacks private parts of both men and women. Circumcision helps to protect women from getting cervical cancer which starts from bacteria hidden at the foreskin of the penis.*
63. I: So as an individual, what is your opinion on integrating partner cervical cancer screening with Voluntary Medical Male circumcision services?
64. *R: This is really a good thing because on your own you cannot move and go at (Name of hospital) hospital for cancer screening, as you get discouraged but it should be integrated here since it is also a chronic disease , that could be a good thing.*
65. I: Alright, what is it that would make you not want integrating cervical cancer screening with Voluntary Medical Male Circumcision?
66. *R: Ah, nothing*
67. I: How do you think is the best way to offer Cervical Cancer screening services within Voluntary Medical Male circumcision clinics?
68. *R: I think by the time you are going for blood pressure and diabetes screening that is when you should also be screened for cervical cancer. They should also have the required or necessary tools or equipment’s for screening.*
69. I: What do you think could be the barriers or concerns with partner cervical cancer screening and Voluntary Medical Male circumcision integration?
70. *R: Hmmm maybe we can say lack of proper tools or equipment’s for screening but I doubt about any concerns if there can be.*
71. I: Alright, let us now discus about PrEP. Have you heard about PrEP?
72. *R: Hmmm… no*
73. I: Alright, I will explain how the medicine works. PrEP is anti-HIV medicine that keeps HIV-negative people from being infected. There is a single pill that is taken once daily, and if you take it regularly, it is highly effective at prevention people from being infected. If you will have to tell someone about PrEP, what can you say it is?
74. *R: PrEP is anti-HIV medicine that keeps HIV-negative people from being infected.*
75. I: That is true. So is it necessary to make PrEP available to HIV- men and women?
76. *R: It is necessary because sometimes in a family one may be unfaithful and can get a virus, but if one is getting PrEP can be protected.*
77. I: Alright fine. So If PrEP become available, what is your opinion on integrating PrEP with Voluntary Medical Male circumcision services? How happy would you be to access them?
78. *R: That is necessary for instance if you are found with HIV, you can get the medicine so that your partner can be taking it and be protected.*
79. I: How do you think PrEP would be offered in this clinic?
80. *R: The medicine should be given maybe before circumcision after screening and if you are not found with a virus.*
81. I: what concerns would you have about people taking PrEP?
82. *R: I think things cannot be working well in the families, as people may become careless due to depending on the medicine available.*
83. I: Eh, so what should be done to address this concern?
84. *R: There is a need give people the right information about these medicine, so that people in the family could understand.*
85. I: Okay, if you were given powers to choose and integrate services in Voluntary Medical Male Clinics, what are the services that you would think of Integrating?
86. *R: Service of PrEP and cervical cancer screening.*
87. I: why have you chose PrEP?
88. *R: PrEP can help to protect myself since nowadays in most marriages things are not working well as one may be unfaithful to the partner.*
89. I: why have you chose cervical cancer screening?
90. *R: It is not easy for me to go and get screened for cancer on my own but this is just like an advantage.*
91. I: Thank you for taking your time to discuss with me today.
92. *R: Thank you.*
93. I: Your answers will be very helpful in improving the health service delivery at circumcision clinics.
94. *R: Thank you.*
95. I: Before we close, do you have anything to say?
96. *R: Yes, there is a question. When did this research start and until when?*
97. I: Alright fine, we have started on Monday but we are going to do it until we reach the expected number. And if it has reached a maximum number of the required participants which is twenty, then we are going to close. But will be analyzing what people have been saying to help improve the health service delivery at circumcision clinics.
98. *R: Is the research only for those who have been circumcised?*
99. I: Yes only those that have been circumcised and health care providers.
100. *R: Okay*
101. I: Alright, thank you so much for your time.
102. *R: Thank you.*

END

**D 43 STUDY**

**Date of Interview: 27 June 2018**

**Type of Participant: Male Index participant**

**Interview Number: D-43-0007**

**Interviewer: C. L.**

**Total Interview Time: 26 minutes 21 seconds**

**Interview Summary**: **(from summary sheet)**

| **SERVICE TO BE INTERGRATED** | **THOUGHTS ON INTERGRATION** |
| --- | --- |
| Couple HIV Testing and Counseling | Thinks it’s a good idea to know each other’s status as a couple. |
| STI Services | Thinks it can help to know if partner has been promiscuous but people need to be sensitized about it. |
| Family Planning | The integration would help couples to access both services at one visit. |
| Cervical Cancer Screening | Thinks it’s a good idea. Partners will come to the VMMC clinic knowing that each will get at least one service. |
| PrEP | Thinks it’s a good idea but people would be surprised to hear that they can take drugs every day when they are not sick. More sensitization needed. |
| Other Services | Thinks cervical cancer screening, couple testing and counseling and STI services are the most important services to be integrated into VMMC |

**Remarks:**

**Participant was relaxed and had no trouble understanding the questions. He was open when giving his opinion and asked where he was not clear**.

1. I: Thank you for taking the time to talk with me today. I would like to ask you some questions today about the way you feel and what you think about some issues related to the service you receive here and how we can include other services in Voluntary Medical Male Circumcision (VMMC) clinics.
2. *R: Mh…*
3. I: There is no right or wrong answers to these questions.
4. *R: Okay.*
5. I: We would like to hear your opinion and your experiences in your own words. Do you have any questions before we begin?
6. *R: No there are no questions.*
7. I: You have no questions?
8. *R: No.*
9. I: Okay. Do you ever talk to your health care providers here at Voluntary Male Medical Circumcision clinic about the services that you receive?
10. *R: Yes we do.*
11. I: Can you give an example of what you talked to them about?
12. *R: When I came the counseled us on what is involved for one to be circumcised, the benefits of being circumcised and wound care. That motivated me to go through the procedure.*
13. I: Okay. Were you able to ask them questions about your concerns?
14. *R: Yes because at first I would just hear people say “ circumcision is not good” but when I talked to the health care providers they told me that it doesn’t take long for one to be normal again and yet you get protected for your entire life.*
15. I: Mm, alright. Now let us talk about couple testing and counseling. What happens when a man brings a partner at the VMMC clinic?
16. *R:[brief silence]*
17. I: If maybe you have ever seen or brought your partner
18. *R: No I have never brought my partner but she just told me.*
19. I: What did she say happens?
20. *R: She said “The way I see it, before we can start arranging for our marriage you need to go for circumcision”. She said that because we are not yet married but there is hope that we will be married and she fears cervical cancer.*
21. I: Mh…
22. *R: Yea so she said she wouldn’t want to develop such a disease so she said it would be better for me to do medical circumcision. So I agreed to what she said.*
23. I: Mm… Have you ever seen any man bring his partner here?
24. *R: No I have never seen.*
25. I: What made you not to bring your partner here?
26. *R: She is not here; she is in Blantyre.*
27. I: So let’s imagine that she was here, would you have brought her to the clinic?
28. *R: Yes and so easily because we both know what we are doing.*
29. I: Okay so if she had been here what would have been her involvement in the services that you get here?
30. *R: It would have been a motivation for me. She would have said like “Since you are already here, have no fear. Things will be okay”.*
31. I: Okay. So your opinion, what are your thoughts about bringing in your partners for HIV testing at Voluntary Medical Male circumcision clinic
32. *R: It’s a good idea because when I came here they tested me for HIV. So it can be good if you came together with your partner so that they test you together and you know your status and that will be good because your will know each other’s status and if one partner has HIV then you will know how you can help one another.*
33. I: Mh. So what is your opinion on integrating couple HIV counseling with Voluntary Medical Male circumcision services? So that men are encourage to bring their partners for couple testing and counseling?
34. *R: As I said there is nothing wrong with that.*
35. I: So do you think there can be any concerns regarding this integration?
36. *R: I don’t think so because if you have already agreed to go for couple testing and counseling then it’s not an issue.*
37. I: Mh, there cannot be any concerns for other people?
38. *R: No, I don’t see any reason for concern.*
39. I: Okay so what do you think makes men not to bring their partners?
40. *R: Ah it might be that they don’t know that you can bring a partner to the medical circumcision clinic.*
41. I: Okay, so now I want us to talk about **s**exual reproductive health services and Pills for HIV prevention: called pre-exposure prophylaxis. (PrEP)
42. *R: Mh…*
43. I: Sexual reproductive health includes services that promote good sexual health and reproduction. They include but not limited to family planning, cervical cancer screening sexual transmitted infection management cervical, condom distribution and many more**.**
44. *R: Okay*
45. I: Today we will only discuss family planning, Sexual transmitted infection management cervical screening and PrEP. Sexually transmitted infection management involves screening for STIs just like screening for cervical cancer.
46. *R: Okay*
47. I: We will look at each of these one by one. Let us start with screening and management of STIs.
48. *R: Okay.*
49. I: What have you learnt about STIs when you came to the VMMC clinic?
50. *R: They told me that if you have an STI then you cannot undergo medical circumcision because sometimes it happens that the STI has developed into a blister which is producing puss, so they cannot circumcise such a person until the infection is treated and the wound heals.*
51. I: What did the health care providers say would happen to you if they suspected or diagnosed you with an STI?
52. *R: If they diagnosed you with the STI they said they turn you back until the wound heals then you can come back and after assessing that’s when they can do the circumcision.*
53. I: So did they screen you for the STIs?
54. *R: Yes they did.*
55. I: They did?
56. *R: Yes.*
57. I: what is your opinion on integrating STI services with Voluntary Medical Male Circumcision services?
58. *R: It’s a good development because when you come here together you will know whether your partner has been promiscuous or not and you will know how to protect each other.*
59. I: Mh, okay.
60. *R: Sure.*
61. I: So how do you think is the best way to offer STI services at the Voluntary Medical Male Circumcision clinics?
62. *R: Like…?*
63. I: Like how best can this STI service be offered here at VMMC so that people can be accessing them?
64. *R: Oh for people to access them?*
65. I: Yes.
66. *R: Okay I think sensitizing people in the villages and telling the people that if a man is suspecting an infection then he needs to go to the hospital and seek medical circumcision services.*
67. I: Mh…
68. *R: Yes because the people need to be sensitized first before they come to the…*
69. I: To the clinic?
70. *R: Yes before they come to the VMMC clinic.*
71. I: Okay. So where within the VMMC clinic flow should the STI screening be included? Since you have gone through the whole clinic flow and you know how it is.
72. *R: Yes, I know.*
73. I: So where should STI screening be included?
74. *R: At the very beginning of the whole process that is when they should do the screening.*
75. I: That’s screening for the STIs?
76. *R: Yes, they should do the screening before you undergo any other procedure because you can go through the other processes and tell you later that you have an infection. Say for example they have already circumcised you and you just realize later that your wound is not healing, so you can be blaming the health care workers and can think that medical male circumcision is a bad idea.*
77. I: Alright. SO what is it that you may not like about this integration?
78. *R: Ah I cannot have any concerns on my part.*
79. I: Even if there should be couples coming?
80. *R: No, I have no concerns.*
81. I: Okay. Now let’s talk about family planning. Tell me anything you know about family planning.
82. *R: Family planning…. ah but it’s just a few things that I know.*
83. I: Yes, that’s okay.
84. *R: I know there are different methods of family planning which include natural method…others use pills, and if it’s men some use condoms*
85. I: Mm…
86. *R: Mm, that’s if they don’t want to have more children. If it’s a woman she can be on a family planning method and if they don’t want children anymore then the man can have vasectomy, I think.*
87. I: Mh…
88. *R: Sure.*
89. I: So what is your opinion on integrating family planning into Voluntary Male Medical Circumcision clinic?
90. *R: I think it’s a good way of controlling population growth*
91. I: Mmm
92. *R: Because then you can be planning as to how many children you want to have.*
93. I: Mm…
94. *R: Sure*
95. I: So what is it that you like about integrating family planning into Voluntary Male Medical Circumcision clinic?
96. *R: It will help because you will know that once you come here for circumcision and you get family planning then things will be fine at home.*
97. I: What is it that you do not like about this integration? Remember we are talking about family planning which both men and women can take part in.
98. *R: Ah no there is nothing I don’t like about the integration. The problem is that there is overpopulation nowadays and if this continues then things cannot be good. So I see nothing wrong with this integration.*
99. I: Alright. SO what do you think is the best way to offer family planning in Voluntary Male Medical Circumcision clinics?
100. *R: Like when I came… I think the first step is sensitizing the people about the whole process that happens here while they are home so that they should be aware of what they will be doing here at the clinic because it may happen that someone comes to the clinic seeking family planning but does not know that there is also Voluntary Male Medical circumcision that is offered here, so it may affect him when he goes home. But when leaving home, he should know that he is coming here for voluntary Male Medical Circumcision but he will also access family planning services.*
101. I: Mm…
102. *R: Yea.*
103. I: Where or at what time point should they offer the family planning at the clinic?
104. *R: they should do that together at the same time…*
105. I: Which time point?
106. *R: Maybe when you are switching from one room to the other they can be sensitizing you concerning family planning methods that are there.*
107. I: That is before going to theatre for procedure?
108. *R: Yes.*
109. I: Okay, so you said you have no concerns concerning this integration, but how do you think people would view this integration between family planning and Voluntary Male Medical Circumcision?
110. *R: Ah every person is different and the way we perceive or understand issues is also different so some people may feel that this integration is not a good development and can say “I only came here for Voluntary Male Medical Circumcision, why are they telling me about family planning?”*
111. I: Mm…
112. *R: Yea.*
113. I: So how can we deal with such an attitude?
114. *R: As I said earlier on what needs to be done is sensitization about what is involved in medical circumcision clinic and about family planning so that when they come here they already know which services they will be coming here for.*
115. I: Mm... Okay, now let’s talk about screening for cervical cancer.
116. *R: Mm…*
117. I: What do you know about cervical cancer?
118. *R: It’s a cancer that mostly attacks the cervix of women whose partners have not done Voluntary Male Medical Circumcision. So I heard it’s a serious disease.*
119. I: Mm, okay. So this type of cancer can be screened to check if a woman has it or not. So what do you think about integrating cervical cancer screening into Voluntary Male Medical Circumcision?
120. *R: I don’t see any problem with that.*
121. I: Okay what would you like about this integration?
122. *R: When we come with our partners we will know that we are coming to the clinic for Voluntary Male Medical Circumcision but also screening for cervical cancer.*
123. I: Okay. How would you be pleased to come with your partner to access these services?
124. *R: I would be very pleased to come with her.[laughing]*
125. I: What is it that you would not like about this integration which would make you not to come with your partner to access the services?
126. *R: there is nothing that would stop me from coming and with the way things have changed nowadays, we need to be moving with the world*
127. I: Okay. So how best can this cervical cancer screening be offered at the clinic?
128. *R: People get information through different means and for some to get information it takes time because they have no access to a radio, TV, newspaper. So there needs to be brief presentations which can reach the people in the villages maybe using village clinics as platforms to give such information. Such information can reach people even if they have no access to the internet or know nothing about the internet.*
129. I: Mh…
130. *R: So with the proper channel and sufficient information, people can be able to understand the integration.*
131. I: Mm..
132. *R: Sure.*
133. I: So what do you think can be the concerns or barriers against this integration?
134. *R: We cannot run away from concerns or barriers because whenever there is a new development happening, people will always oppose it. Say for example when I came I only wanted to do the medical circumcision, right?*
135. I: Mm…
136. *R: So for someone to come and hear about the cervical cancer issue they might think “Ah they have started offering cervical cancer screening at the VMMC clinic! I won’t go back there because I only wanted VMMC and here they were telling me about family planning, cervical cancer. I don’t want anything to do with family planning, all I want is VMMC”. So some people may think of not coming back.*
137. I: Mm, okay. So how can we address such concerns so that people don’t feel like “I only wanted VMMC, why are they telling me about cervical cancer?”
138. *R:]laughing] It’s like I said that if the people are sensitized on how the integration of VMMC and cervical cancer and family planning then when going to the VMMC clinic they will have an open mind knowing that when they come to the VMMC clinic they will get messages about family planning and also cervical cancer.*
139. I: Mh…
140. *R: So they will prepare their minds and know what happens here at the clinic unlike someone from the village who doesn’t know anything and when he comes here he will just hear “After this we want to screen you for cancer or something” so it will come to them as something they were not expecting.*
141. I: Alright so apart from sensitizing them in the communities what can be done at the clinic so that they can be able to come?
142. *R: If there could be some space here, I don’t know…maybe if there was some designated healthcare providers who could be stationed here to give more information about the integration to the clients that come here whether they have just for the VMMC or other services, they need to know about all the services that are available.*
143. I: Mh.
144. *R: Sure.*
145. I: Alright, lets now talk about PrEP. Tell me anything you know about PrEP.
146. *R: I have ever heard but I don’t really know what it is.*
147. I: Okay, what is it that you heard about it?
148. *R: I cannot specifically say but I just heard the term. I don’t even know what it is or what it is related to.*
149. I: Okay. So when we say PrEP we are talking about medicine that keeps HIV-negative people negative. There is a pill which is supposed to be taken one pill per day and every day. If one takes the pill regularly as per prescription, then he can be effectively protected from HIV.
150. *R: Okay.*
151. I: Yes but you need to take the pill regularly.
152. *R: Alright.*
153. I: So how do you feel about PrEP?
154. *R: You said it’s a drug that you take to prevent HIV infection.*
155. I: Okay, so do you think it’s necessary for this medicine to be available to HIV-negative men and women?
156. *R: [chuckling] it would be surprising to someone who has never taken the drug or has never heard of it. It’s like telling someone who is HIV-negative to be taking ARVs [laughing]*
157. I: [laughing] Okay
158. *R: Yes, so it can be something strange and surprising to say “How is that possible that I am not infected and I should be taking such drugs?”*
159. I: Mh…
160. *R: But it’s a good development because it can prevent people from being infected. I feel that it’s very helpful.*
161. I: Mh…
162. *R: Mm… that’s according to what you have said about it.*
163. I: If PrEP was available, what is your opinion about integrating PrEP into Voluntary Male Medical Circumcision?
164. *R: I feel that it’s a good development…*
165. I: How is it a good development?
166. *R:…that when people are told about PrEP and those that are HIV negative can be taking the drugs while those that are positive…maybe something else can be done about them.*
167. I: Mm, how best can this PrEP be offered at the VMMC clinic?
168. *R: I think they can be offering the drugs right there at the VMMC clinic…if there is some space then they can utilize it to for the distribution.*
169. I: Okay, at what time points can PrEP be offered?
170. *R: I think before one starts the whole process of VMMC.*
171. I: Before the process?
172. *R: Yes.*
173. I: And before they screen or test you for anything?
174. *R: Yes.*
175. I: So what happens if one is found HIV positive after being given the drugs?
176. *R: [laughing] then that can be a problem. But they can just give back the drugs because they won’t need them.*
177. I: What concerns would you have if people are receiving PrEP?
178. *R: Mmm, every new development faces opposition…*
179. I: Mh…
180. *R: Especially for someone who is not sick…because if one is sick and is taking drugs he has faith that one day he will be healed but for one to be taking drugs without being sick, some would wonder “What will those drugs be doing in my body?”*
181. I: Mh…
182. *R: It’s different from a vaccine which you are given to prevent some kind of disease but for you to be taking pills every day when you are not sick, some would wonder what the drugs will be doing in their bodies.*
183. I: Mm, so what needs to be done to address such concerns?
184. *R: I think it’s the same thing that I talked about earlier on to say people need to be sensitized about all these services. They need to be told what PrEP is and what it does so they would know that when they come to the VMMC clinic they will find PrEP.*
185. I: Mm, okay.
186. *R: Sure.*
187. I: If you were given powers to choose and integrate services in Voluntary Medical Male Clinics, what are the services that you would think of Integrating?
188. *R: I would choose all the services that you have talked about because they are beneficial to the people that can access them.*
189. I: Okay, so I wanted you to list the ones which you feel can be integrated with VMMC.
190. *R: Okay, like you have talked about cervical cancer screening. That is very important for people to know and have access to.*
191. I: Mh…
192. *R: And the other services which you mentioned like…what were the other services?*
193. I: Couple testing and counseling,…
194. *R: Yes, couple testing and counseling and STI management is also important so that people should know when coming that they will have access to such services.*
195. I: Why have you chosen couple counseling and testing?
196. *R: They say that VMMC does not protect someone by 100 percent, so you cannot say now that you are circumcised then you will just be having unprotected sex, no. But firstly you need to know whether you have HIV or not before you get VMMC so that when you are HIV positive then you need to know how you can lead a positive life, say “Now that I am positive, how best can I protect my partner?” That way the couple can be using condoms for protection.*
197. I: mmm, okay. Why have you chosen cervical cancer?
198. *R: It’s because that cancer affects women who stay innocently at home when men are busy putting their lives at risk and yet they are not circumcised. So everything ends with the woman and that’s why people need to be sensitized about cervical cancer and how dangerous it is.*
199. I: Okay. So thank you for taking your time to talk with me today. The answers that you have given will be very helpful in improving the health service delivery at circumcision clinics. But before we close, is there anything you want to say?
200. *R: Yes, I just want to thank the VMMC procedure that has been put in place because most of us, like me I was just so afraid and had so many questions about what VMMC is all about. So a lot of things are said about it and people say “VMMC is not good “but I have seen that it’s a good thing because they have advised me on what I should be doing or how I should take care of my wound and the benefits of VMMC. So it’s something precious to me.*
201. I: Mh...
202. *R: Sure*
203. I: Alright thank you for your time and for talking with me today.
204. *R: Alright, thank you.*
205. END

**D 43 STUDY**

**Date of Interview: 26 June 2018**

**Type of Participant: Male Index participant**

**Interview Number: D-43-0008**

**Interviewer: C.L.**

**Total Interview Time: 37 minutes 14 seconds**

**Interview Summary:** **(from summary sheet)**

| **SERVICE TO BE INTERGRATED** | **THOUGHTS ON INTERGRATION** |
| --- | --- |
| Couple HIV Testing and Counseling | A good way to get rid of trust issues |
| STI Services | A good idea because one needs to know if they have an infection before circumcision. |
| Family Planning | Didn’t know anything about it but thinks it’s a good idea because it can improve a woman’s health. |
| Cervical Cancer Screening | A good idea but clients need to come as couples |
| PrEP | Could prevent people from infection if integrated with VMMC |
| Other Services | Thinks cervical cancer screening and PrEP are the best to be integrated into VMMC |

**Remarks:**

**Participant was relaxed and had no trouble understanding the questions. He did not have any information regarding family planning which restricted his answers to the questions.**

**Interview Text:**

1. I: Thank you for taking the time to talk with me today. I would like to ask you some questions today about the way you feel and what you think about some issues related to the service you receive here and how we can include other services in Voluntary Medical Male Circumcision (VMMC) clinics. There is no right or wrong answers to these questions. We would like to hear your opinion and your experiences in your own words. Do you have any questions before we begin?
2. *R: Ah, there is no question.*
3. I: Alright. Do your ever talk to your care providers about how the services are provided here?
4. *R: Yes we do*
5. I: What did you talk about?
6. *R: At first we could just hear from yellow vans about circumcisions, but I came here and they explained to me. I came back the following day, after making my decision for circumcision and I was tested for HIV and other STIs, and it was all good until I was circumcised. Every procedure was completed.*
7. I: Were you able to ask questions?
8. *R: Yes, I was asking them questions because it was depending as they could say you might be feeling pain before you wake in the morning, and I was like “ but naturally, we men, usually in the morning, when we are about to wake up, our penis elects automatically, so how could we be helped? And I was told to be drinking water as a remedy, and it will come back to normal on its own.*
9. I: Alright, so now let us talk about partner HIV testing here at the circumcision clinic. Did you ever bring in your partner for HIV testing here at a clinic?
10. *R: Yes, I did but it was not at this circumcision clinic. It was at (Name of hospital). I did not bring in my partner here because it was way back and now she is my ex-girlfriend [Laughing…]. We broke up for her own known reasons.*
11. I: Can you come together with your partner or wife assuming you are married?
12. *R:* *Yes, I can because it is necessary as I cannot just say am fine on my own without my partner knowing also her status, which will be like destroying myself.*
13. I: Alright. What can motivate you to bring her in here at circumcision clinic?
14. *R: I came here for circumcision and my blood was tested and follows all the process required. She is supposed to be, so that she can witness and believe what I went through. If I come alone, it will be like am hiding something else from her, but we need to come together and hear whatever we want to, together.*
15. I: Okay. Have you ever seen a man bring in his spouse at circumcision clinic?
16. *R: No*
17. I: Did they explain anything concerning coming with your partner?
18. *R: Yes, they did. They said, it is okay to come with your partner for blood testing.*
19. I: In your opinion, what are your thoughts about bringing in your partners for HIV testing at Voluntary Medical Male circumcision clinic?
20. *R: Like I said, the issue is to be strong, if you are bringing in your partner, she should realize that what you are doing is good and that you want to know your body is safe.*
21. I: What made men not to bring their partners here?
22. *R: sometimes, the thing is that they do not believe in themselves. Sometimes it is fear but what need to be done, it is just to gain courage and come with your partner.*
23. I: What do you think can be done to make men bring their partners here for HIV testing and counselling?
24. *R: About this issue, what is needed is empowerment and to spread information so that others could also hear about it just like we did. To say the truth, I did not know at first we needed to be coming with our partners for blood testing until we were told, and if they are told too, this can work.*
25. I: Okay, so you as Individual, what is your opinion on integrating couple HIV counseling with Voluntary Medical Male circumcision services?
26. *R: It is a good thing as for instance in most families there is trust issues. For example if I can come here alone for circumcision, my partner will ask me why I came alone and even I get my blood tested, I would still be questioned, But it is good to be coming together as everyone knows the status of the partner and if your status is good, you know how to protect your partner.*
27. I: What do you think are the barriers and concerns on this integration?
28. *R: it can result into marriage break ups due to found with different HIV status. So people may be scared with that.*
29. I: What do you think should be done to overcome these concerns and barriers to couple counseling in Voluntary Medical Male circumcision clinic?
30. *R: what is needed there is counselling. Just like they did there, they welcomed, explained to us, how we can do it and how we can take care of ourselves, it is just the same.*
31. I: Now I would like us to discuss about sexual reproductive health services and Pills for HIV prevention: called pre-exposure prophylaxis (PrEP).Sexual reproductive health include services that promote good sexual health and reproduction. They include but not limited to family planning, cervical cancer screening sexual transmitted infection management cervical, condom distribution and many more. Today we will only discuss family planning, Sexual transmitted infection management cervical screening and PrEP. We will look at each of these one by one. Let us start with STI services. Explain to me, what you have ever learnt about STIs when you came to Voluntary Medical Male circumcision clinic?
32. *R: what I have learned is to protect myself. If I wouldn’t have been here, I could have been doing things blindly as a result it could have destroyed me. They have clearly explained to me how to protect myself to prevent such STIs.*
33. I: What did they explain to you about STIs?
34. *R: They did not explained*
35. I: What did the staff say would happen if you were found or suspected with STIs?
36. *R: They just said it is complicated. Let us take for example, if you are found with an STI and you have been circumcised, you can be feeling pain and not because of the* *circumcision but the infection. This could result in discouraging people for circumcision.*
37. I: As an individual, what is your opinion on integrating STI services with Voluntary Medical Male Circumcision services?
38. *R: It’s a good idea because you need to know the problems which you have before circumcision. After counselling you do have a choice.*
39. I: How do you think is the best way to offer STI services at the Voluntary Medical Male Circumcision clinics?
40. *R: Before circumcision that is when you need to be screened for STIs.*
41. I: What do you think are the barriers and concerns on this integration of STI services with Voluntary Medical Male circumcision services?
42. *R: The concern might be there, in terms of fear of being found with an STI.*
43. I: What is it that you would like about the integration of diagnosis and management of STI services in Voluntary Medical Male services?
44. *R: if the child is using abusive language, let him because he does not know what he is saying. If we come together and we have been found with STI, if we went home, we will not be quarrelling or arguing. You will be even living a happily life in your relationship.*
45. I: So what do you think should be done to address these concerns and barriers?
46. *R: What is needed is counselling. Explain to others how it works.*
47. I: Let us talk about Family Planning. Explain to me about anything that you know about family planning
48. *R: I cannot lie; I do not know anything about family planning. But I remember the methods like nor plant, pills, injection….*
49. I: As an individual, what is your opinion on integrating Family planning in Voluntary Medical Male circumcision services/clinic?
50. *R: That could help as a family in terms of family development and also helps to improve the health of women.*
51. I: What is it that you would like about integrating Family Planning services in Voluntary Medical Male Circumcision?
52. *R: What I would like is that, I could be knowing what to do because you have already said that they should be coming as a family. They should be all knowing time for having a child and time for spacing.*
53. I: What would make you not want integrating Family Planning services in Voluntary Medical Male Circumcision services?
54. *R: There are some beliefs that some may not be willing to go for family planning. Some people do not accept who they are for example after every two years, you might be losing the wife and your life becomes miserable.*
55. I: Okay. How do you think is the best way to offer family planning services within Voluntary Medical Male circumcision services clinics?
56. *R: Let us say they have come together as a family for circumcision, they should be having a choice. This should be after screening and before circumcision.*
57. I: What do you think could be the barriers or concerns on family planning and Voluntary Medical Male circumcision integration?
58. *R: I do not think there could be any concern because it depends on your own choice.*
59. I: So… let us discuss about cervical cancer. Explain to me what you know about cervical cancer screening
60. *R: I heard about cervical cancer in a class and it was being taught by our teacher but not in brief.*
61. I: Okay, so, as an individual, what is your opinion on integrating partner cervical cancer screening with Voluntary Medical Male circumcision services?
62. *R: That could be a good thing if they go as couple.*
63. I: What is it that would like about integrating cancer screening with Voluntary Medical Male Circumcision services?
64. *R: we have done about circumcision and we have completed.*
65. I: What is it that would make you not want integrating cervical cancer screening with Voluntary Medical Male Circumcision?
66. *R: it could be misunderstanding between two people as a family because one may want to go together while the other may want to go alone because of fear.*
67. I: How do you think is the best way to offer Cervical Cancer screening services within Voluntary Medical Male circumcision clinics?
68. *R: It is just the same as it is for blood testing, and should be explained after she can make a choice but it should be in a private place. A person should be screened anytime she arrives and depending on the programme.*
69. I: What do you think could be the barriers or concerns with partner cervical cancer screening and Voluntary Medical Male circumcision integration?
70. *R: It could be intimidating the women thinking about how she will give birth if she has been found with cancer.*
71. I: How do you think these concerns and barriers be addressed?
72. *R: If you have been found with cancer, a person should be given a treatment and comforted.*
73. I: Ehm... Now let us discus about PrEP. Have you heard about PrEP?
74. *R: No*
75. I: PrEP is anti-HIV medicine that keeps HIV-negative people from being infected. There is a single pill that is taken once daily, and if you take it regularly, it is highly effective at prevention people from being infected.
76. *R: But my question is, are you supposed to be taking it every day when you are sleeping with a person who you do not know his status?*
77. I: No
78. *R: But you just have to be taking it daily?*
79. I: Yes. But is it necessary to make PrEP available to HIV- men and women?
80. *R: it is necessary because the disease which has affected the whole world, it is this one. So for those who cannot manage to abstain, can be greatly helped.*
81. I: What concerns would you have about people taking PrEP?
82. *R: If your body gets used to the medicine, it can have some effects.*
83. I: If PrEP become available, what is your opinion on integrating PrEP with Voluntary Medical Male circumcision services? How happy would you be to access them?
84. *R: this could help to protect a person if circumcision is combine with PrEP.*
85. I: How do you think PrEP would be offered in this clinic?
86. *R: After circumcision, that’s when it can be offered. The person should be explained and make a decision later whether to take or not.*
87. I: What concerns would you have about people taking PrEP?
88. *R: It can result in to a certain behavior as they start to be careless*
89. I: What should be done to address these concerns?
90. *R: Need to educate people on how to use PrEP… and its prescription.*
91. I: If you were given powers to choose and integrate services in Voluntary Medical Male Clinics, what are the services that you would think of Integrating?
92. *R: PrEP, Cervical cancer…*
93. I: Why have chosen PrEP?
94. *R: That is a medicine and the disease which everyone fears nowadays is HIV.*
95. I: Why have you chosen cervical cancer screening?
96. *R: This protect family*
97. I: How do you think these services should be offered in the clinic?
98. *R: Those should only be offered to a person who have come for circumcision. Everyone who have been tested for HIV, should be the one given PrEP. Cervical cancer, women should have power to go with her husband for screening.*
99. I: Okay. Thank you for taking your time to discuss with me today. Your answers will be very helpful in improving the health service delivery at circumcision clinics. Before we close, do you have anything to say?
100. *R: No*
101. I: Again, thank you so much for taking your time to speak with me.
102. *R: Thank you.*

END

**D 43 STUDY**

**Date of Interview: 26 June 2018**

**Type of Participant: Male Index Participant**

**Interview Number: D-43-0003**

**Interviewer: C.L.**

**Total Interview Time: 36 minutes 31 seconds**

**Interview Summary:** **(from summary sheet)**

| **SERVICE TO BE INTERGRATED** | **THOUGHTS ON INTERGRATION** |
| --- | --- |
| Couple HIV Testing and Counseling | Integration would provide a good opportunity for couples to know each other’s status. Women can be motivated for sound care if they come together with their men for VMMC. |
| STI Services | Would help couples to be cautious not to get STIs. Thinks the screening room should be the same which is already there. |
| Family Planning | Integration would open up another opportunity and raise family planning awareness for men to easily access family planning and |
| Cervical Cancer Screening | Partners could take advantage of coming with their partners at The VMMC clinic and get cervical cancer screening. Thinks some men may not feel comfortable with women at the clinic. |
| PrEP | VMMC would be an avenue for awareness for PrEP. |
| Other Services | Thinks cervical family planning and PrEP are the best to be integrated into VMMC |

**Remarks:**

1. I: So, I welcome you to our discussions today. And I thank you for taking the time to talk with me today. I would like to ask you some questions today about the way you feel and what you think about some issues related to the service you receive here and how we can include other services in Voluntary Medical Male Circumcision (VMMC) clinics. So, there is no right or wrong answers to these questions. We would like to hear your opinion and your experiences in your own words. Do you have any questions before we begin?
2. *R: Ah, no.*
3. I : There is no question
4. *R: No.*
5. I: Alright fine. So my first question is ….do you ever talk to your care providers about how the services are provided here?
6. *R: Here?*
7. I : Yes
8. *R: Ehm, the first day, they explained and open a floor for questions*
9. I: Okay.
10. *R: Yes.*
11. I: So what did you ask?
12. *R: I asked that, how long the process take to be well.*
13. I: how did they respond?
14. *R: It take seven day for the stitches to be removed*
15. I : Okay
16. *R: Yeah… but it also depends how you are taking care of yourself by following the given prescriptions, and with that all works well.*
17. I: Okay. Did they talk to you about anything which maybe brought a concern to you?
18. *R:they also said that, it reduces HIV contraction with sixty percent and you can easily take good care of your private parts*
19. I: Okay fine. So let us talk about partner HIV testing here at the circumcision clinic, like a family together. Did you ever seen a man coming with his wife or partner for circumcision?
20. *R:Ah… no, I have not seen them*
21. I: You have never seen a man coming with his partner?
22. *R: Yes*
23. I: Did you bring in your spouse when you came?
24. *R: Ah, no, I did not [Laughing…]*
25. I: [Laughing …], why not?
26. *R:It was just fear and out of that, I just made a decision on my own as I did not talk about it with my partner*
27. I: you did not talk about it?
28. *R: Yes*
29. I: Alright, so…
30. *R: But she was encouraging me to go for circumcision. But with fear… [Laughing]*
31. I: what makes fear?
32. *R: A certain fear just comes... [Laughing.] Like that there is a possibility that you can be found with diseases.*
33. I : Okay.
34. *R: Yes, also maybe because to my perception, I used to see circumcision as a painful experience, so, those are the fears which prevented me from coming.*
35. I: Ehm okay, so… did you know that you can come for circumcision and test for HIV together with your partner?
36. *R: Ah, that I did not know*
37. I : You did not know
38. *R: Yes I did not know*
39. I: Let us take as if you knew, could you have come together with your wife?
40. *R: If I could have known, I could have come with her because she was the one encouraging me to go for HIV testing but I was reluctant. But if I knew, it could have been easier.*
41. I : Okay.
42. *R: Yes*
43. I :In your opinion, what are your thoughts about bringing in your partners for HIV testing at Voluntary Medical Male circumcision clinic
44. *R: I think,… it can be a good opportunity to know their standing*
45. I : How do you think people may welcome and accept it, you being in a group of men
46. *R: [Laughing] Ah, for us men, mostly it is fear which can go in the negative side but it depends maybe others can accept it, depending on their background*.
47. I: Okay, when you say background, what do you mean?
48. *R:I can say that, you are exposed to unprotected sex before getting engaged with the person you with and that brings fear*
49. I: Can those encourage or discourage to come in with your partner?
50. *R: mostly it is obstacle as I cannot say they do it alone as there some who are courageous but it is an intimidation thing.*
51. I: Okay, so what is your opinion on integrating couple HIV counseling with Voluntary Medical Male circumcision services?
52. *R: Mm… maybe it can help in a way that, like they say in English, killing two birds with one stone, it can really help.*
53. I: Okay, who can be helped?
54. *R: Both of them in the family*
55. I: Mmm, but what do you think are the barriers and concerns on this integration of HTC for couples?
56. *R: As for me, I think it is an advantage in terms of care because I started passing out pus on my wound, maybe because of care, as I was applying salt, how I was applying it and the amount and cleaning. I think a woman can be helping you out the process of hygiene in taking care of the wound.*
57. I: Okay, now I would like us to discuss about sexual reproductive health services and Pills for HIV prevention: called pre-exposure prophylaxis. (PrEP). So if we say, sexual reproductive health includes services that promote good sexual health and reproduction. They include but not limited to family planning, cervical cancer screening sexual transmitted infection management cervical, condom distribution and many more. So today we will only discuss family planning, Sexual transmitted infection management cervical screening and PrEP. We will look at each of these one by one. Let us start with STI services. Explain to me, what you have ever learnt about STIs when you came to Voluntary Medical Male circumcision clinic?
58. *R: Ah, of course what I have learned is that, before you get circumcision there are a number of thing which can bring complications because on upper urethra, if you are not circumcised, the opening get exposed on the upper, where it is a vulnerable place where you can contact HIV/ AIDS. So by closing it, you prevent HIV by sixty percent.*
59. I: Mmm, so how about other diseases like sexual transmitted diseases. What diseases did they mentioned?
60. *R: They also said that, ah… I think, he just generalizes like that sexually transmitted diseases get reduced.*
61. I: Okay, so what did the staff say would happen if you were found or suspected with STIs?
62. *R: Ah they said that, there is a treatment that can prevent you from proceeding for circumcision. They said it depends on extent of your immunity.*
63. I: Mmm, okay so if your immunity has gone down what happened?
64. *R: Then you cannot proceed, because that prevent …[coughing], it delays your healing process*
65. I: Okay, so if you have been found with STI and your immunity is high can them…
66. *R: yes they said that there is a possibility that you can be circumcised*
67. I: okay, what is your opinion on integrating STI services with Voluntary Medical Male Circumcision services? First of let me ask you, did they get you screened for STI?
68. *R: Yes they did*
69. I : Okay
70. *R: I think it could be a good thing as couple because most times you can stay without being noticed as it is a normal thing, without knowing. But it is a good thing as you can know*
71. I: Okay, so how do you think is the best way to offer STI services at the Voluntary Medical Male Circumcision clinics?
72. *R: I think ,… it can depend but I think the best way is to have awareness, as you are coming, you should be bringing in your partner as a couple after coming willingly. It should be offered before you get circumcised.*
73. I: Okay can you explain the process of circumcision after a person arrived until he gets circumcised.
74. *R: You enter first, then you get briefed, then HIV test and STDs, then they ask you for the consent of HIV testing. Then they test your blood and wait for fifteen minutes. Then after that if you are found to be negative, you proceed to the other stage of circumcision.*
75. I: So according to the process which you have explained, when is a good time as a couple to be screened for STIs?
76. *R: Should be combined with HIV testing*
77. I: Okay fine, what is it that you would like about the integration of diagnosis and management of STI services in Voluntary Medical Male services?
78. *R: As a family, that means you have been protected because it can be that, on your family it has been a while since you had sex and one can be a carrier while you can pass through screening while the wife can still be a carrier.*
79. I : Mh…
80. *R: So by that process, you both remains on the same ground*
81. I: so, what is it that you do not like integrating STI services in Voluntary Medical Male circumcision services?
82. *R: [Laughing], it could be possible that one can be a carrier and the other can be well. You might be found to be disadvantaged, and it can be complicated as wife can be a carrier and not you.*
83. I: Okay, so what do you think should be done to address these concerns and barriers?
84. *R: Hmmm… there is no way but the only way is through counseling, giving them viable way*
85. I: Alright, let us talk about Family Planning. Explain to me about anything that you know about family planning
86. *R: Family planning is like, hmmm…. birth control, after having sexual intercourse, they use it before seventy-two hours to prevent birth.*
87. I : Okay, what are other methods because you have just explained about pill
88. *R: Mmm… other methods, a women can be … am not clear about it but it is a process, they do inject her that … that after certain years she could not be giving births.*
89. I: Okay fine. Like you have explained, family planning is birth control and there are different methods. The one you explained first, we call emergence pill where a person take it if she does not want to get pregnancy after having unprotected sex. But there are also other pills which a women need to be taking every day not to get pregnant. There is also an injection method as you explained it, they call it Depo but there is also another which they do inject on the shoulder and they call it in-plant. There is also loop, and there are a lot of different family planning methods. There is another method for men whom they call it Vasectomy. And so if we say family planning, we do not mean only women but also men as couple. As an individual, what is your opinion on integrating Family planning in Voluntary Medical Male circumcision services/clinic?
90. *R: Mm… I think it can also be a good idea because you are opening up options for people. Like myself, I rarely come to the hospital and about circumcision was new to me. So if it can be integrated, it can help to bring awareness more especially to us men.*
91. I: Mmm, what do you think could be the barriers or concerns on family planning and Voluntary Medical Male circumcision integration?
92. *R: Mmm, on this one, there is nothing*
93. I: Nothing. But how do you think is the best way to offer family planning services within Voluntary Medical Male circumcision services clinics?
94. *R: Mmm that one I think… it can be because we do have a number of days for checkup, I think maybe it can be that, you can be briefed before circumcision beside circumcision, there are other options like family planning, that can be available but if you can opt to do that, you can be coming during checkups because I doubt if one may want this service on the first day.*
95. I: Do you think this integration should be in the circumcision clinic or separate?
96. *R: even if it is in the same building because I see the advantage of awareness which is one of the ways, of bringing awareness to men.*
97. I: Okay so what do you think could be the barriers or concerns on family planning and Voluntary Medical Male circumcision integration?
98. *R: The agreement can be there between a husband and wife because one maybe that he or she still want to be having children and doing it on your own it could be a problem. You can make a decision whether to come together or not. The only problem is agreement.*
99. I: How do you think these concerns and barriers be addressed?
100. *R: Having a talk about it…*
101. I: Okay, should they be talking on their own or we should be involved?
102. *R: you can be involved, explaining to them how it can help them because where there is knowledge, there is power, and they will be able to make decision.*
103. I: Okay fine. Let us talk about cervical cancer screening. Explain to me what you know about cervical cancer screening.
104. *R: I heard people talking about it but it wasn’t in details. About cervical cancer screening, they were advising women to go for checkups*
105. I: Okay, as an individual, what is your opinion on integrating partner cervical cancer screening with Voluntary Medical Male circumcision services?
106. *R: Mm… I don’t know whether … but I think… if we come with a partner, it could be an advantage but some men could not be comfortable because men are sensitive with women. But if you come with your wife, it could be an advantage.*
107. I: what is it that would like about integrating cervical cancer screening with Voluntary Medical Male Circumcision services?
108. *R: [Laughing] nothing*
109. I: Nothing. So with the process of circumcision, how do you think they could be screening for cervical cancer?
110. *R: Ah, that could a situation when you come with your partner, and by the time you go for circumcision, that’s when a women can be going for cervical cancer screening.*
111. I: Who should be screened?
112. *R: Anyone who is willing*
113. I: Can’t it complicate anything?
114. *R: No.*
115. I: Alright what do you think could be the barriers or concerns with partner cervical cancer screening and Voluntary Medical Male circumcision integration?
116. *R: Mostly could be peoples opinion as they could not take it seriously thinking that women will also be there*
117. I: Okay, how do you think these concerns and barriers be addressed?
118. *R: I think there is a need to clear things by making them aware*
119. I: Okay, let us talk about PrEP. Have you heard about PrEP?
120. *R: Can we it is that medicine for birth control right*
121. I: No, PrEP is anti-HIV medicine that keeps HIV-negative people from being infected. There is a single pill that is taken once daily, and if you take it regularly, it is highly effective at prevention people from being infected. While PEP, you take it after you have been exposed or slept with someone who you do not know his blood status. So how can you explain about PrEP?
122. *R: Mm… I can say, PrEP is another way which can help you after having unprotected sex with a person who has HIV. It can prevent.*
123. I: so from the way you have heard, is it necessary to make PrEP available to HIV- men and women?
124. *R: [Laughing]… Yes, there is advantage and disadvantage as it can brings quarrels like you are prepared that you are going to sleep with someone else. So the trust can be a problem but the advantage is that if it has happened but I do not advice it as I do not know its side effects, whether it can boost or not or whether to be talking while you are in a marriage. But sometimes it can help, so am at the middle.*
125. I: If PrEP becomes available, what is your opinion on integrating PrEP with Voluntary Medical Male circumcision services? How happy would you be to access them?
126. *R: It is a good way because as I said earlier, it is one of the avenues for awareness and helping, that’s why for people like me, we did not think over it for coming here, it was just a one-time decision and yet we have now known about PrEP. I could be happy to receive it, as just from prevent, everyone would like to take it.*
127. I: Okay, how do you think PrEP would be offered in this clinic?
128. *R: Were they do brief, that is where it could be offered.*
129. I: So it should be given to whom?
130. *R: You be getting it as a couple or individually.*
131. I: What concerns would you have about people taking PrEP?
132. *R: Some people may take it as escape code. Some people maybe careless due to the presence of these drugs.*
133. I: What should be done to address these concerns?
134. *R: emphasize more when you are briefing people about PrEP.*
135. I: If you were given powers to choose and integrate services in Voluntary Medical Male Clinics, what are the services that you would think of Integrating?
136. *R: Ah, nothing besides PrEP and Family planning. I think many people may opt for PrEP and Family planning.*
137. I: Thank you for taking your time to discuss with me today. Your answers will be very helpful in improving the health service delivery at circumcision clinics. Before we close, do you have anything to say?
138. *R: No*
139. I: Again, thank you so much for taking your time to speak with me.

END

**D 43 STUDY**

**Date of Interview: 02 July 2018**

**Type of Participant: Male Index Participant**

**Interview Number: D-43-0011**

**Interviewer: C. L.**

**Total Interview Time: 31 minutes 02 seconds**

**Interview Summary:** **(from summary sheet)**

| **SERVICE TO BE INTERGRATED** | **THOUGHTS ON INTERGRATION** |
| --- | --- |
| Couple HIV Testing and Counseling | A good idea only that men need to be sensitized. Trust issues and secrets can prevent men from bringing their partners to the clinic. |
| STI Services | Good initiative only that it can depend on how open people are in their relationships. Would prefer service to be offered at the beginning of the VMMC clinic flow |
| Family Planning | The integration would make it easier for men to have access to family planning |
| Cervical Cancer Screening | A good initiative only that privacy issues for men need to be considered. |
| PrEP | A good option only has issues with taking the drug daily. |
| Other Services | Thinks screening for cancer in men and PrEP are the best choices to integrate with VMMC |

**Remarks:**

**Participant was relaxed and had a good sense of humor. He expressed his feelings freely except for when we discussed Screening for Cervical cancer. But later he was able to freely talk about it after probing.**

**Interview Text:**

1. I: Thank you for taking the time to talk with me today. I would like to ask you some questions today about the way you feel and what you think about some issues related to the service you receive here and how we can include other services in Voluntary Medical Male Circumcision (VMMC) clinics. There is no right or wrong answers to these questions. We would like to hear your opinion and your experiences in your own words.
2. *R: Okay.*
3. I: Do you have any questions before we begin?
4. *R: No.*
5. I: You have no questions?
6. *R: No.*
7. I: Okay so do you ever talk to your care providers about how the services are provided here?
8. *R: Yes I do.*
9. I: Can you give me an example of a time when you managed to talk to your health care provider about the services you received here?
10. *R: An example of…?*
11. I: Like what you talk about or the questions you ask when you come for the service?
12. *R: Oh, like when you come you start with the HTC room and they tell you what VMMC is all about: that’s it’s about removing the foreskin. Then after HIV testing you go into the screening room where they screen for STIs or ulcerations on the foreskin or any signs that you may have that can make you not eligible for the process. Then you sign a consent form which reads that there may be some adverse events during or after the procedure because it’s a surgery.*
13. I: Mmm
14. *R: Yes that’s that.*
15. I: What questions did you ask about VMMC?
16. *R: I asked how VMMC helps one to be hygienic and also in the consent form they wrote somewhere that during the procedure they can mistakenly cut the penis. So that made me afraid and I wanted to know how that can happen.*
17. I: So what did they say?
18. *R: About the issue that they can cut the penis they said it can be either you as a client or if something goes wrong during the surgery and that’s on them as providers. It can happen that the surgery goes on well but you as a client don’t take care of your wound so it can be infected and it can go to the extent of cutting the penis.*
19. I: Mmm, okay. Now let’s talk about couple testing and counseling here at VMMC clinic.
20. *R: Okay.*
21. I: Tell me what happens when a man brings his partner to the VMMC clinic?
22. *R: I wouldn’t know because I have never brought my partner here.*
23. I: And you have never seen anyone bring his partner here?
24. *R: No, I have never seen.*
25. I: Okay, what prevented you from bringing your partner here?
26. *R: It’s because I don’t have [laughing]*
27. I: [laughing] So let’s imagine you have a partner, what would motivate you or discourage you to bring her to the VMMC clinic?
28. *R: If I had a partner I would bring her to the VMMC clinic because if you love someone then at some point you end up having sex with her, so you just need to know her status because you don’t know how she has been leading her life in the past.*
29. I: Mmm
30. *R: So you need to know whether she is HIV positive or if there are some things about her that would potentially harm your future, especially for us the youth.*
31. I: Mmm. What do you think discourages men to bring their partners? Or should we say all of them that came did not have partners?
32. *R: [chuckles] Well I think it may be shame, and sometimes your partner can feel like you don’t trust her if you tell her to go for HIV testing.*
33. I: Mmm
34. *R: Yea so sometimes it comes down to trust issues.*
35. I: Mmm, do you think men are aware that they can bring their loved ones to the VMMC clinic?
36. *R: I doubt that because most people view VMMC clinic as a clinic for men and circumcision and not a place for couple testing and counseling. So I doubt that people know.*
37. I: So what are your thoughts about bringing in your partners for HIV testing at Voluntary Medical Male circumcision clinic
38. *R: It depends on how open your partner is because some partners can feel shy thinking that there will only be men at the clinic. But it’s a good idea to be coming as a couple.*
39. I: Okay what is your opinion on integrating couple HIV counseling with Voluntary Medical Male circumcision services?
40. *R: I think it’s a good idea only that men need to be sensitized about it.*
41. I: Do you think the integration can be acceptable to men?
42. *R: Yes they can accept it. It’s only that right now people don’t know about it but if they can be sensitized then they can accept it the same way they accepted VMMC.*
43. I: What do you think can be the concerns or barriers for this integration?
44. *R: I think it’s only the trust issues and secrets that could prevent people from coming but otherwise there is nothing that could hold them back*
45. I: Okay. Now I would like us to discuss about sexual reproductive health services and Pills for HIV prevention: called pre-exposure prophylaxis. (PrEP) Sexual reproductive health includes services that promote good sexual health and reproduction.
46. *R: Mmm*
47. I: They include but not limited to family planning, cervical cancer screening sexual transmitted infection management cervical, condom distribution and many more
48. *R:*
49. I: Today we will only discuss family planning, Sexual transmitted infection management cervical screening and PrEP. We will look at each of these one by one. Let us start with sexually transmitted infection management. Tell me what you have ever learnt about STIs when you came to Voluntary Medical Male circumcision clinic?
50. *R: That VMMC reduces the risk of STIs like HIV and the like… they said when you have done VMMC your risk of infection for HIV reduces by 60 percent, and that you can be 60 percent positive that you cannot be infected.*
51. I: Mmm
52. *R: And… yea I think that’s that.*
53. I: How about other STIs other than HIV?
54. *R: Mmm, they didn’t tell us anything about that. They only focused on HIV and cervical cancer which starts because of the bacteria in the foreskin. As for the other STIs they didn’t tell us more.*
55. I: Okay. What did they say would happen if they suspected or diagnosed you with STIs?
56. *R: They said if they diagnosed you with STIs then you would not be eligible for VMMC.*
57. I: What kind of STIs would make you ineligible?
58. *R: At first they said “if we diagnosed you with HIV you will not be eligible”. But later when we went into the screening room they said “If we diagnose you with ulcerations and other infections then you will not be eligible for VMMC”.*
59. I: Why did they say you would not be eligible?
60. *R: No, they didn’t say.*
61. I: Okay so what is your opinion…firstly, were you screened for the STIs?
62. *R: Yes.*
63. I: Okay so what is your opinion on integrating STI services with Voluntary Medical Male Circumcision services? So that men can also be coming with their partners for screening.
64. *R:[silence]*
65. I: We have talked about couple counseling and testing but now we want to talk about screening for STIs.
66. *R: Well it’s a good initiative only that it would depend on how open you are with your partner.*
67. I: Why do you say so it’s a good initiative?
68. *R: Because some people can go to a clinic and be diagnosed with an STI but still come to their partner and tell them they are okay. But with the integration you will both know about the infection so one wouldn’t lie to their partner about it and you can just talk about how he got infected as a couple and things can be normal again.*
69. I: What do you think can be the barriers or concerns for thins integration?
70. *R: There can be concerns because it can happen that my partner has an STI and I don’t have. So one can have a lot of questions to say “Where did you get the infection? Who have you been sleeping with apart from me?” And that can put marriages on the line.*
71. I: Mmm
72. *R: Otherwise it’s a good initiative.*
73. I: Okay, how best do you think the screening for STIs be included in the VMMC clinic flow?
74. *R: At the very beginning…soon after HIV testing... I think in the HIV testing room because after that room the other rooms are congested so that can lead to delays for the partners. So it can be good for the screening for STIs to be done at the earliest stage so they can go because sometimes long waiting time can lead to fears and some can go back due to that.*
75. I: Mmm
76. *R: But if they can do it at the earliest stage so they can hear their test results that can be good.*
77. I: Okay. Are there any other advantages that you can think of about this integration?
78. *R: Aah, maybe it can also protect an unborn child. That’s all I can think about.*
79. I: Okay. You said there can be a concern that some marriages can be at risk due to this integration. How do you think we can address this concern?
80. *R: It’s hard to address such a concern because that has nothing to deal with you as health care providers; it’s a problem which is in their marriage. So it’s hard to predict what can happen but it depends on the type of people that you are dealing with.*
81. I: Okay. So let’s say none of the partners has ever come for screening and they are both worrying what will happen if they get screened and things turn bad. How can we address such a concern?
82. *R: You can tell them that sometimes STIs can be transmitted from mother to child so it can happen that one has the infection but got it from his mother. So that could also help.*
83. I: Mmm, alright. Let’s talk about family planning. Tell me anything that you know about family planning.
84. *R: Like…?*
85. I: Like what would say family planning is?
86. *R: It’s about taking care of a child that you have.*
87. I: Mmm.
88. *R: That’s the only response that I have right now.*
89. I: Mmm, do you know any family planning methods that are available?
90. *R: Mmm, well I don’t know if maybe Safe Plan is one of the methods.*
91. I: Mmm
92. *R: Yea that’s the only one I know of.*
93. I: Alright. There are different types of family planning, some of which are female related and others for men. It just depends on the couple whether they decide that the man should be the one family planning or it should be the woman. So family planning is all the methods that people use in order not to conceive at that time. This can include the Safe Plan that you mentioned or condoms or vasectomy for men, depo, pills and so on.
94. *R: Okay.*
95. I: So what is your opinion on integrating Family planning in Voluntary Medical Male circumcision services/clinic?
96. *R: As in they should be offering family planning at the VMMC clinic?*
97. I: Yes so that people can be accessing family planning at the VMMC clinic.
98. *R: That can be good. It can be the same as screening for STIs because that is all about sexual and reproductive health, right?*
99. I: Mmm
100. *R: Yea so it can be a good thing.*
101. I: How do you think men can benefit if family planning is integrated in VMMC clinic?
102. *R: It can be easy for them to access the service because some men find it difficult to go to a clinic for family planning services in the same way people fear coming to the VMMC clinic. So both services are at one place it can benefit men a lot.*
103. I: Mmm okay. What do you think could be the barriers or concerns on family planning and Voluntary Medical Male circumcision integration? We have talked about the benefits but now what could be the barriers or what is it that you would not like about it.
104. *R: I don’t see anything wrong or any concern that a person can have because this is for his own benefit.*
105. I: Mmm
106. *R: So I don’t think there is any draw back with this integration.*
107. I: In your opinion don’t you think men can have concerns with this integration?
108. *R: No I don’t think so.*
109. I: Okay at what time point do you think family planning service can be integrated into VMMC clinic?
110. *R: [chuckles] I feel at the very beginning of the process because towards the end of the process there are generally delays because the surgery takes time so people pile up waiting for the surgery.*
111. I: Mmm.
112. *R: So I feel that it would be better that once people come they should be told about family planning or maybe they can just combine everything starting from what is involved for one to be circumcised, STI screening…or they can either be offering the service at the beginning and at the HTC room because some people don’t feel comfortable being told issues like these in a group while others are comfortable like that, it just depends on the approach they use.*
113. I: Mmm
114. *R: So right at the beginning and at the HTC room can help people to feel free.*
115. I: Mmm, now let’s talk about cervical cancer screening.
116. *R: Mmm*
117. I: Tell me what you know about cervical cancer.
118. *R: I know that it’s a disease that is caused bacteria that develops under the foreskin of a man’s penis, some people call it “gaga”. So if you deposit it in the woman’s cervix it can cause cancer.*
119. I: Mmm, okay. Have you ever heard about cervical cancer screening?
120. *R: Yes I have ever heard.*
121. I: What did you hear about it?
122. *R: I just heard people say “There is this other cancer which can be screened and if diagnosed at an early stage they can help you. If they find it later then there is some medication which they can give you to prolong your life”.*
123. I: mm. So what is your opinion on integrating partner cervical cancer screening with Voluntary Medical Male circumcision services? So they can be screening cervical cancer for women at the VMMC clinic.
124. *R:Mmm, I feel like sometimes it’s hard for men and women to fell free with each other.*
125. I: Mmm
126. *R: So as it is at the VMMC clinic, I think it’s easier for men to come and talk about issues concerning VMMC especially for those that went through it already to encourage others because sometimes it’s easy to understand someone that went through an experience more than a doctor.*
127. I: Mmm
128. *R: So I feel that it is better for the VMMC clinic to be the way it is so that men can freely talk about issues. Sometimes we speak so loudly that it help someone who is not there speaking with us but if we combine then we cannot speak as loud as we do because there will be women around. That’s why I said earlier on that any type of screening should be done at the beginning so that the partners can be done, go home and leave men to have a chance to talk and encourage each other.*
129. I: Mmm. So you feel it can be difficult?
130. *R: Yes it can be difficult.*
131. I: Mmm okay. Now let’s talk about PrEP.
132. *R: PrEP is anti-HIV drug right?*
133. I: Yes. But not PEP right?
134. *R: [chuckles] Oh Yes, I know PEP not PrEP.*
135. I: Okay PrEP is an anti-HIV drug that keeps HIV-negative people from being infected. There is a single pill that is taken once daily, and if you take it regularly, it is highly effective at prevention people from being infected.
136. *R:Mmm.*
137. I: So a single pill needs to be taken every day not just when you feel like you are exposed or will; be exposed to HIV.
138. *R: Oh you need to be taking the pill each and every day?*
139. I: Yes. If you are taking it regularly then you can prevent HIV infection even if your partner is HIV positive.
140. *R: Okay.*
141. I: So do you think it is necessary for PrEP to be made available to HIV-negative men and women?
142. *R: I can say it’s a good thing but the fact that people will be taking drugs every day…I fell they may see it as a burden for them to be taking it. If maybe there could be a drug which they can be taking once a month, then that could help a lot.*
143. I: Mmm
144. *R: Yea instead of them taking the drug every day.*
145. I: Mmm
146. *R: Yea but it’s a good option because it’s better to be safe than at risk.*
147. I: okay so what is your opinion on integrating PrEP with Voluntary Medical Male circumcision services? How happy would you be to access them?
148. *R: It’s a good option because at the VMMC clinic you meet other people that are HIV positive and they tell you their experience which makes you think about HIV that much. So if there is another option apart from circumcision that could reduce the risk of HIV infection then it can be a better option.*
149. I: Okay, how happy would you be to access the drugs?
150. *R: I would access the drugs but…[laughing]*
151. I: Would you take them?
152. *R: It would be an issue for me to take them…the fact that you need to be taking the drugs each and every day would be something. But it’s a good option because you can be taking the drugs regularly but…[laughing] it’s something that would face negativity from people because taking the drugs each and every day is an issue.*
153. I: What would be your concern if people were taking PrEP? That’s apart from the issue that some would see it as a burden to be taking a pill every day.
154. *R: There is nothing wrong with it.*
155. I: You have no other concern?
156. *R: No I don’t see anything wrong with it.*
157. I: Okay, so how do you think we can address the concern that you, mentioned that people would think it’s a burden to take a pill every day?
158. *R: I think if they can transform the drug into an injection so that we can get one shot in a month just like they transformed some ARVs…I just heard there are some ARVs which can be taken once a year or once a month.*
159. I: Mmm
160. *R: Yea that can help people a lot.*
161. I: How do you think PrEP would be offered in this clinic?
162. *R: They can be offering it together with the painkillers which they give after the surgery. They can firstly describe what PrEP is in the HTC room and then offer it at the end of the procedure.*
163. I: Mmm. Okay. Let’s go back to the cervical cancer screening.
164. *R: Mmm*
165. I: You mentioned that providing screening at the VMMC clinic would make some men uncomfortable. So what can be done so that the screening for cervical cancer is done in a way that makes men comfortable?
166. *R: Mmm well I don’t know what takes place on the other side of the clinic but I just noted that there are a lot of women there. So if maybe they can be doing testing and counseling at the very beginning so that after testing the women can be heading to the other side of the corridor where there are a lot of women and men can continue with the flow, because there is nothing wrong with the integration.*
167. I: Oh okay, so that doesn’t mean there should be no integration?
168. *R: No they should still screen for cervical cancer because it’s very important that they do so.*
169. I: Mmm, what is it that you would like about this integration?
170. *R: I heard that cancer is fatal so if they can be screening and treating it at an early stage then they can save people from death or discomfort.*
171. I: Okay. What do you think can be the barriers on the part of men to bring their partners for screening or for women to come for screening?
172. *R: As for women I think it can be fear. As for men it depends on how they love and talk to their wives about it. But I feel it’s possible, it all depends on the nature of relationships that people are in because there are some relationships where partners are not free to talk to each other but are free with an outsider. So such cases can be difficult for a couple to have such a discussion and come together so that while the man goes for circumcision the woman goes for cervical cancer screening.*
173. I: Mmm, you mentioned that women cannot come because of fear. Why would they be afraid?
174. *R: They can be afraid that if they are found to have cancer then that’s the end of their lives and that people would look differently at them.*
175. I: Okay. So there are other services that are available in other clinics which we have not discussed here. So imagine you have the power to choose and integrate such services with VMMC, which ones would you choose?
176. *R: Which services?*
177. I: Services which can be integrated with VMMC. So far we have discussed couple testing and counseling, STI services, screening for cervical cancer, family planning and PrEP.
178. *R: Mmm*
179. I: So if you are given the power to choose and integrate, which services would you choose?
180. *R: I feel like sexual health related services are already available at the VMMC. For example they screen for STIs and perform VMMC. So I feel like they are already available at the VMMC clinic and I don’t think you can add any other reproductive health service.*
181. I: I was saying this in the context that at the VMMC clinic they don’t offer family planning, PrEP or screening for cervical cancer. So which services would you choose to integrate with VMMC service?
182. *R: The ones you have just mentioned?*
183. I: Yes the ones I have mentioned and any other that you feel can be integrated with VMMC service.
184. *R: I feel like PrEP is a good option and very helpful except for the fact that you need to be taking it every day. But otherwise I feel like it’s a good option to end HIV. But as for other services I feel that they could e screening for cancer in men…that’s because I heard men can also have cancer.*
185. I: So they should be screening for cancer in men?
186. *R: Yes.*
187. I: Why have you chosen that?
188. *R: heard that there is testicular cancer which attacks men. So if they are screening for cervical cancer in women then it’s only fair to also be screening cancer in men as well.[chuckling]*
189. I: So how best can that screening for cancer in men be done?
190. *R: In the screening room where they screen for STIs and the like, so they can use that opportunity to be screening for cancer.*
191. I: Alright. Thank you for taking your time to discuss with me today.
192. *R: Thank you*
193. I: Your answers will be very helpful in improving the health service delivery at circumcision clinics
194. *R:* Alright.
195. I: Before we close do you have anything to add?
196. *R: No, there is nothing.*
197. I: Alright thank you.

**D 43 STUDY**

**Date of Interview: 03 July 2018**

**Type of Participant: Male Index Participant**

**Interview Number: D-43-0012**

**Interviewer: C. L.**

**Total Interview Time: 37 minutes 24 seconds**

**Interview Summary:** **(from summary sheet)**

| **SERVICE TO BE INTERGRATED** | **THOUGHTS ON INTERGRATION** |
| --- | --- |
| Couple HIV Testing and Counseling | A good idea only that men need to be motivated to come with their partners. No circumcision for those that don’t come with their partners. |
| STI Services | Good idea because as a couple if one is infected then the other is also at risk so there is need for them to know. There needs to be proper flow at the VMMC clinic |
| Family Planning | The integration would make it easier for men to have access to family planning since most of them do not know a lot about family planning. |
| Cervical Cancer Screening | Feels cervical cancer and VMMC are directly connected so couples need to be screened before the man goes for VMMC so they can be treated and protected. |
| PrEP | A good option because most men that come to the VMMC clinic are HIV negative so they need the pills more. Only concerned that people would not protect themselves. |
| Other Services | Thinks couple testing and counseling, STI services and family planning are the best to integrate with VMMC. |

**Remarks:**

**Participant was relaxed and had a good sense of humor. He had lots of suggestions for the integrations and it was easy for him to understand the questions.**

**Interview Text:**

1. I: Welcome to our discussion today and thank you for taking the time to talk with me today. I would like to ask you some questions today about the way you feel and what you think about some issues related to the service you receive here and how we can include other services in Voluntary Medical Male Circumcision (VMMC) clinics.
2. *R: Okay*
3. I: There is no right or wrong answers to these questions. We would like to hear your opinion and your experiences in your own words.
4. *R: Okay.*
5. I: Do you have any questions before we begin?
6. *R: No I have no questions.*
7. I: You have no questions?
8. *R: No.*
9. I: Okay. Do you ever talk to health care providers about the services that you receive at the VMMC clinic?
10. *R: Yes, I do.*
11. I: Can you give an example of when you talked to them about the services?
12. *R: Like before I came to the VMMC clinic, I met some health care providers who gave me information about VMMC and what is involved for one to be circumcised.*
13. I: Mh…
14. *R: So I felt that it’s a good thing for them to give me the information before I went to the clinic because it was like they were giving me the power of knowing what happens when you go to the VMMC clinic so I could choose whether I should undergo the procedure or not.*
15. I: Okay. So what did you talk to them about when you came to the VMMC clinic?
16. *R: We had a good discussion as well…*
17. I: Mh…
18. *R: There were two providers: one female and the other male. We talked a lot about VMMC.*
19. I: What did they say about it or what kinds of questions did you ask them?
20. *R: I asked them more especially about the pain that I would go through. SO I asked them what they do to deal with the pain and they said they use anesthesia to reduce the pain and after the pain is reduced that’s when they do they surgery.*
21. I: What concerns did you have?
22. *R: I was afraid that I might feel pain during the surgery but it turned out that I didn’t feel anything. Things went on well.*
23. I: Okay. Now I want us to talk about HIV testing at the VMMC clinic.
24. *R: Okay.*
25. I: In your opinion, what happens when a man brings his partner to the VMMC clinic for couple testing and counseling?
26. *R: It’s a good idea for a man to bring his partner for couple testing and counseling because it’s an advantage for both of you to know your status before the man gets circumcised because some people think that once you are circumcised you cannot get HIV but you can get HIV if you are not protecting yourself.*
27. I: Alright. Have you ever brought your partner at the VMMC clinic?
28. *R: No, I have never brought my partner to the VMMC clinic but we have ever tested for HIV together.*
29. I: Where did you go for testing?
30. *R: We tested here at the hospital.*
31. I: At the VMMC clinic or the other part of the hospital…?
32. *R: The other part of the hospital not at the VMMC clinic.*
33. I: Mm, why did you not bring your partner to the VMMC clinic?
34. *R: I can say it was because we were far from each other at the time I came for VMMC because I just thought about coming here for VMMC when I was already in town.*
35. I: Mh…
36. *R: Yes, so that’s why it was kind of hard for us to communicate to come together here at the clinic.*
37. I: Did you know that you could come together with your partner for couple testing and counseling here at the VMMC clinic?
38. *R: I didn’t know at that time…*
39. I: But when you came they explained that you could come with your partner?
40. *R: Yes, they said it’s very important for us to be coming with our partners.*
41. I: Have you ever seen any man bring his partner at the VMMC clinic?
42. *R: No, at the time I came for surgery I didn’t see any man bringing his partner at the clinic.*
43. I: How about at another time when you came?
44. *R: Yes, at the time I came for review I saw a man bringing his partner.*
45. I: What do you think makes men not to bring their partners to the VMMC clinic?
46. *R: For some men it’s because of shame. Others it’s because of lack of communication between them and their partners that they fail to talk to the partner that they are coming for the surgery, that’s why they come alone.*
47. I: Mmm
48. *R: But if they can talk about it while at home, it can be easier for them to be coming as couples.*
49. I: Okay. So what do you think can be done for men to be coming with their partners for couple counseling and testing at the VMMC clinic?
50. *R: I think motivating the men that come for VMMC to come with their partners can help to spread the message. Another way is by turning back the men that come for VMMC to come back the next day for the surgery with their partners.*
51. I: Okay, so telling them to come back…?
52. *R: Yes, before they undergo the surgery telling them to bring their partners either the same day or the next so they can get couple testing and counseling.*
53. I: Okay. So let’s imagine we have that rule in place that all men should be bringing their partners for couple testing and counseling before they get VMMC? What concerns can there be for this integration?
54. *R: There cannot be any concerns because before one gets circumcised he goes for HIV so there cannot be an issue with one getting tested with his partner because if the man really wants to get VMMC then he needs to get his blood tested. So I don’t see any concerns with that.*
55. I: Okay. You already know the clinic flow at the VMMC clinic right?
56. *R: Yes*
57. I: So imagine they tell you “You need to bring your partner first to get couple counseling and testing before you can get circumcised” How could you accept that or how do you think other men can accept that?
58. *R: I think some men can welcome it while others cannot because people are different. However, I feel like most men can welcome the idea.*
59. I: What can make other men not to welcome the idea?
60. *R: It can be because of fear and also because they are not aware of what is involved for one to get circumcised.*
61. I: Mmm
62. *R: So it can be fear because they didn’t know that HIV testing is also done at the VMMC clinic.*
63. I: Mmm. So how can we deal with such a concern or barrier?
64. *R: I think there should be special counselors who can be reaching out to such men frequently, so they can no longer be afraid.*
65. I: Okay. What kind of message should that be?
66. *R: [Clears his throat] Motivating them and telling them that HIV testing is not the end of everything, it’s just a way of knowing one’s status.*
67. I: Mm... Now let’s talk about sexual reproductive health services and Pills for HIV prevention: called pre-exposure prophylaxis. (PrEP) Sexual reproductive health includes services that promote good sexual health and reproduction. They include but not limited to family planning, cervical cancer screening sexual transmitted infection management cervical, condom distribution and many more.
68. *R: Mh…*
69. I: Today we will only discuss family planning, Sexual transmitted infection management cervical screening and PrEP. We will look at each of these one by one. Let us start with STI services.
70. *R: Mh…*
71. I: Tell me what you have ever learnt about STIs when you came to Voluntary Medical Male circumcision clinic?
72. *R: I learnt that one can get STIs even after being circumcised because VMMC reduces one’s risk of infection by say 60 percent. That means the remaining 40 percent shows that one can still get infected.*
73. I: *Mh…*
74. *R: So I can say the greatest thing that I have learnt is using protection like condoms.*
75. I: Alright. What did health care workers say would happen to you if they suspected or diagnosed you? I am not just talking about HIV but I mean all the STIs.
76. *R: They didn’t say anything in connection with that but I think if they diagnose you with an STI like syphilis or gonorrhea then they cannot be able to do the surgery unless you get treated for the STI first.*
77. I: Why do you think they cannot be able to do the surgery?
78. *R: I think it can be difficult because… [clears throat] it can be like…I can say the infection can cause complications to the VMMC surgery.*
79. I: Okay.
80. *R: Yes.*
81. I: Did they screen you for STIs?
82. *R: Yes, they did.*
83. I: Okay, what is your opinion on integrating STI services with VMMC services so that you can be bringing your partner at the VMMC clinic to be accessing such services?
84. *R: It’s a good idea.*
85. I: It’s a good idea?
86. *R: Yes.*
87. I: Why do you say so?
88. *R: It’s because as a couple you are one body and if one is infected then it means both of you are infected. Of course it can happen that one is infected while the other is not but still you need to know if one is infected or not.*
89. I: So how do you think men can welcome this idea if they hear that women will also be coming to the VMMC clinic to access STI services?
90. *R: I think they can welcome the idea. Like for me, I feel that it’s a good development.*
91. I: Mmm. As someone who has undergone the VMMC clinic flow, when is the best time to be offering this STI service at the VMMC clinic with this integration?
92. *R: Like…?*
93. I: Like at what time point should they be offering the STI service? After what service or room?
94. *R: Okay, I feel the right time is when they are doing HIV testing.*
95. I: So they should be screening for STIs as a couple when they do HIV testing or it should be done one on one?
96. *R: Mh…*
97. I: I am asking that for couples.
98. *R: [chuckles] there can be some couples that wouldn’t like it but generally it’s important that both should be tested together.*
99. I: Why do you think it’s important for couples to be tested together?
100. *R: It’s because they are a couple and they are doing one thing.*
101. I: So what *is it that you would like about the integration of diagnosis and management of STI services in* Voluntary Medical Male *services?*
102. *R: There are some people that are shy and they have no information about STI screening and management, so for such people to go to the hospital by themselves just to get screened for STIs it can be difficult. But because at the VMMC clinic it’s like people will be going for other services as well then it will be easier.*
103. I: What is it that you don’t like about this integration?
104. *R: What I don’t like about this integration is a situation where a female nurse screens a man and a male nurse screens a woman.*
105. I: Why would that bother you?
106. *R:* *[Interruption]…*
107. I: What would bother you if a female nurse screens a man?
108. *R: It may depend on how people think but it’s not a serious issue. I am just stating my opinion that I wouldn’t like it.*
109. I: Okay, who screened you for STIs when you came? Was it a man or a woman?
110. *R: It was a man.*
111. I: So that made you happy?
112. *R: Yes, that made me happy.*
113. I: Okay. What do you think can be concerns or barriers to this integration? Let’s start with women: what do you think can make them, fail to come for the STI services?
114. *R: For this integration to work there needs to be two offices, otherwise if one office is used for couples and singles then that would pose a problem. If there can be two offices so that one is used for coupes and the other is used for singles, then it can be okay.*
115. I: Okay so what do you think can be a concern or barrier for women to come?
116. *R: If they have not been told properly what they would be doing at the clinic then they can fail to come. Otherwise there is nothing that would make them fail to come.*
117. I: How about for men? What would make them not to bring their wives?
118. *R: What can make them fail to bring their wives…?*
119. I: Yes, at the VMMC clinic.
120. *R: It may be due to shyness or misinformation from their friends. Say a man tells his friends that he is about to take his wife to the VMMC clinic, how his friends can react is what can make him fail to come to the clinic.*
121. I: Okay. How do you think this shyness can be dealt with? You have mentioned that there needs to be two rooms: one for couples and the other for singles. But how else can we address this issue of shyness?
122. *R: I think…like I said that there needs to be proper flow at the clinic so that when clients come they should be properly welcomed and not be spoken to harshly especially by the doctors. So that will make clients happy and willing to come and access these services because they will know that the reception here is very good.*
123. I: Okay. Now let’s talk about family planning. Tell me anything you know about family planning.
124. *R: I know that family planning is good. I also know family planning methods like the one they use injection and the other they call it Loop.*
125. I: Mmm
126. *R: Yea.*
127. I: So there are other different types of family planning methods like pills,

implanon and also another one for men. Have you ever heard about family?

planning that involves men?

1. *R: No I have never heard about that?*
2. I: It’s called Vasectomy.
3. *R: Oh, vasectomy...*
4. I: Yes there is **tubal ligation** and there is Vasectomy for men. All this are family

planning methods.

1. *R: Oh okay.*
2. I: So as an individual what is your opinion on integrating Family planning in

Voluntary Medical Male circumcision services/clinic?

1. *R: It’s a good thing because if it will be like that at the VMMC clinic then couples will be coming together at the clinic after discussing what services you will be needing at the clinic and you will know what you will be getting when you come at the clinic. So it’s a good thing that family planning should be made available at the VMMC clinic.*
2. I: What else do you like about this integration?
3. *R: There is something else that I like about this integration…most men do not*

*have enough information about family planning but if they are given that information when they come to the VMMC clinic I feel like they can readily accept it.*

1. I: Mh…
2. *R: Yes.*
3. I: What is it that you don’t like about this integration?
4. *R: I don’t see any problem with this integration.*
5. I: At what time point do you think family planning can be offered in the VMMC

clinic flow?

1. *R: It should be offered before one goes for surgery.*
2. I: Before surgery?
3. *R: Yes.*
4. I: Which rooms should that be?
5. *R: It should be like I said that if it’s a couple then there should be a separate*

*room so that these services can be offered on concurrently*

1. I: How about for those that are not married? Say they are partners but not

married?

1. *R: Even those can go to the couple’s room because they will also come as a*

*couple.*

1. I: Okay. So what do you think can be the barriers or concerns for some men when they hear that family planning has been integrated into VMMC clinic? You know this is a VMMC clinic and we are trying to integrate it with another service that’s normally not there right?
2. *R: Yes.*
3. I: So what do you think can be the concerns or barriers for this integration?
4. *R: I think the concerns can come from those men that are not married because then there will be more couples coming to the clinic. So they may feel shy or something.*
5. I: So how can we deal with that?
6. *R: Reaching out to those people with sensitizations and how they can still use the VMMC services.*
7. I: Mmm, are there other concerns or barriers for this integration?
8. *R: Ah no, there is nothing else.*
9. I: Alright. Now let’s talk about cervical cancer screening. Tell me anything you know about cervical cancer.
10. *R: [Brief silence]*
11. I: Have you ever heard about cervical cancer?
12. *R: Yes, I have ever heard about cervical cancer.*
13. I: What is it that you heard?
14. *R: I heard cervical cancer is a type of cancer that attacks women who have ever had sex but the high risk is for those whose partners have never done VMMC because of the bacteria that develops under the foreskin.*
15. I: Okay. So have you ever heard about screening for cervical cancer?
16. *R: Aah, no. I have never heard about that.*
17. I: You have never heard about that?
18. *R: No.*
19. I: Alright. So some clinics screen for cervical cancer in women.
20. *R: Okay.*
21. I: As an individual what is your opinion on integrating partner cervical cancer screening with Voluntary Medical Male circumcision services?
22. *R: Ah, it can be good if such a service is integrated into the VMMC service because these services have a connection. Cervical cancer develops before circumcision so it’s only good that screening should be done before the surgery so that it can be treated and people can continue protecting themselves.*
23. I: Okay. What do you think can be the concerns or barriers for this integration?
24. *R: There is none.*
25. I: Would you be willing to bring your partner to access this service?
26. *R: Yes, very much.*
27. I: How best can this service be integrated into the VMMC clinic flow? At what time points and where can it be offered?
28. *R: It should be offered before the surgery.*
29. I: Okay…
30. *R: Sure…*
31. I: Should the screening room be inside the VMMC clinic or it should be separate?
32. *R: It should be separate.*
33. I: Why do you say so?
34. *R: It’s important that screening for cervical cancer should be done in separate rooms: one for married people and the other for singles.*
35. I: The question was specifically asking about whether the screening room should be within the VMMC clinic or outside.
36. *R: It should be outside the VMMC clinic.*
37. I: What do you think can be the barriers or concerns for this integration?
38. *R: I don’t think there can be any barriers or concerns.*
39. I: No barriers.
40. *R: No.*
41. I: Now let’s talk about PrEP…
42. *R: PEP?*
43. I: No PrEP, there is an “R”.
44. *R: Okay.*
45. I: Have you ever heard about PrEP?
46. *R: No, I have never heard of it.*
47. I: So since you have not heard about PrEP, I will explain how the medicine works. PrEP is anti-HIV medicine that keeps HIV-negative people from being infected.
48. *R: Mh…*
49. I: There is a single pill that is taken once daily,
50. *R: Okay.*
51. I: And if you take it regularly, it is highly effective at prevention people from being infected.
52. *R: Can I ask a question?*
53. I: Yes, you can ask.
54. *R: Are these pills… only give to those that are HIV-negative?*
55. I: Yes.
56. *R: Okay.*
57. I: Because the aim is to keep those that are HIV-negative to be negative. So once you have started taking the pills you shouldn’t stop. In that way even if you have sex with an infected person you may not get infected.
58. *R: For how many days or years do you have to take the pills? [both laughing] I am asking because one will be taking the pills when they are not sick.*
59. I: If you want to be protected for 10 years then you take the pills for 10 years [both laughing]
60. *R: Okay.*
61. I: Sure. So how do you feel about PrEP?
62. *R: This very good because it will help to reduce HIV infections.*
63. I: Okay. Do you think it is necessary to make this PrEP available to HIV-negative men and women?
64. *R: Yes, it is supposed to be made available because if PrEP is protecting HIV-negative people then it’s automatic that it should be given to those that are HIV negative not those that are positive. So it should be made available only to those that are HIV negative.*
65. I: Alright. Is it necessary for PrEP to be made available for people to be receiving it?
66. *R: Yes, it’s necessary.*
67. I: Why do you think so?
68. *R: It’s because…I feel if someone who is HIV-negative takes this PrEP she will give birth to HIV-negative children.*
69. I: Alright. What is your opinion about integrating PrEP in VMMC clinic?
70. *R: Can I ask first?*
71. I: Mmm.
72. *R: Will this PrEP be given for free?*
73. I: Yes, just like VMMC.
74. *R: So it’s necessary that it should be available at the VMMC clinic.*
75. I: Why do you say so?
76. *R: It’s because most of the men that go to the VMMC clinic and get HIV test are found HIV negative.*
77. I: Okay. How happy would you be to access PrEP?
78. *R: I would be happy but not [background noise] with thinking that since I have taken PrEP then I can be promiscuous, no. I can still be protecting myself and remaining faithful to my partner.*
79. I: How do you think PrEP would be offered at the VMMC clinic?
80. *R: The can be offering PrEP in the HTC room.*
81. I: Okay, what concerns would you have about people taking PrEP?
82. *R: My concern would be that people would no longer protecting themselves because they would be relying on the drugs which they will be taking thinking that even if they can be having unprotected sex they would be protected. So that would be my worry.*
83. I: So how can this concern be addressed?
84. *R: I think if maybe people are not told that PrEP prevents HIV infection. If maybe there was another way of making them take the drugs without telling them the truth.*
85. I: Can you give an example of what you would say to someone to take the pills without telling them the real purpose?
86. *R: I can tell the person that the drugs reduce the risk of HIV infection when having sex.*
87. I: Alright. So if you were given the power to choose and integrate services into VMMC clinic, which ones would you choose?
88. *R: Mh…*
89. I: Remember we have talked about couple testing, STI services, family planning, screening for cervical cancer and PrEP, remember?
90. *R: Yes.*
91. I: But there might be other services which we haven’t talked about here. So imagine you have the power to choose and integrate, which ones would you choose to integrate with VMMC services?
92. *R: Other services?*
93. I: Yes, other services. Which ones would you choose?
94. *R: I feel like we have already mentioned the ones that I would choose from.*
95. I: Which ones are those?
96. *R: Couple testing and counseling, STI services and family planning.*
97. I: Those three?
98. *R: Yes.*
99. I: Why have you chosen couple testing and counseling?
100. *R: I have chosen couple counseling because it will help partners to know each other’s status.*
101. I: How about family planning?
102. *R: I have chosen family planning because as a couple you can decide which one*

*of you stays on a method.*

1. I: Mm… how about STI services?
2. *R: So that as a couple you know if you have infections or not.*
3. I: Mm, okay
4. *R: Sure.*
5. I: Thank you for taking your time to discuss with me today. Your answers will be very helpful in improving the health service delivery at circumcision clinics.
6. *R: Alright.*
7. I: Before we close, do you have anything you want to add?
8. *R: No I don’t have anything else.*
9. I: Alright, thank you for your time.
10. *R: Thanks.*

*END*

**D 43 STUDY**

**Date of Interview: 03 July 2018**

**Type of Participant: Male Index Participant**

**Interview Number: D-43-0013**

**Interviewer: C. L.**

**Total Interview Time: 38 minutes 50 seconds**

**Interview Summary:** **(from summary sheet)**

| **SERVICE TO BE INTERGRATED** | **THOUGHTS ON INTERGRATION** |
| --- | --- |
| Couple HIV Testing and Counseling | A good opportunity for couples to test together. Partner can motivate you to get VMMC. |
| STI Services | Thinks STI services would help more men to get VMMC. Fears the VMMC clinic would be crowded |
| Family Planning | The integration would make it easier for men to have access to family planning since most of them do not know a lot about family planning. |
| Cervical Cancer Screening | Feels cervical cancer is a dangerous disease so integration is very important. |
| PrEP | A good idea as this integration would help to fight HIV better. Only concerned that people would not protect themselves. |
| Other Services | Thinks family Planning services and PrEP are the best for the integration. |

**Remarks:**

**Participant was relaxed and had a good sense of humor. He easily understood the questions and was very open-minded.**

**Interview Text:**

1. I: Thank you for taking the time to talk with me today. I would like to ask you some questions today about the way you feel and what you think about some issues related to the service you receive here and how we can include other services in Voluntary Medical Male Circumcision (VMMC) clinics.
2. *R:Okay*
3. I: There is no right or wrong answers to these questions. We would like to hear your opinion and your experiences in your own words.
4. *R: Alright.*
5. I: Do you have any questions before we begin?
6. *R: No I don’t have any questions.*
7. I: Do you ever talk to your care providers about how the services are provided here?
8. *R: Yes. I am saying that because the last time I came here I had a lot of questions that I asked them and they were able to respond…and they were open minded to me.*
9. I: What kind of questions did you ask them?
10. *R: I asked them how the procedure is like for me to get VMMC because when we are out there in the communities or at home we hear a lot of things about VMMC, some of which is just to scare us but it’s not true, but some is the truth. I can give you an example: some people tell us that sometimes people are turned back when they come here for VMMC and that made us wonder why there is such discrimination. But when we came here and asked they said “it’s not discrimination. We fear that if we perform the procedure while they have an infection, the wound may fail to heal”.*
11. I: Mm..; okay. Now let’s talk about couple testing and counseling.
12. *R: Okay.*
13. I: Tell me what happens when a man brings his partner to the VMMC clinic?
14. *R: Well I came alone…but I have ever seen some men bring their wives to the clinic only that I didn’t get to know what happened with them.*
15. I: Mm, okay. Why did you not bring your partner?
16. *R: Well, it’s been a long time since I got an HIV test, so I felt that it was not wise for me to bring my partner for couple testing when I was not aware of my status. Another reason was that I was not fully aware what the procedure was like here.*
17. I: You mean you didn’t know that you could come with your partner for couple counseling and testing?
18. *R: Yes, it’s like I didn’t have a clear picture of what happens here.*
19. I: Mmm. Let’s imagine you knew that you could come with your partner here, would you have come with her considering the fact that you have said that it’s been a long time since you had an HIV test?
20. *R: Aah it would have been a bit hard but if I had talked to her and she accepted then I could have brought her here.*
21. I: Have you ever seen a man bring his partner here at the VMMC clinic?
22. *R: Yes, on the day that I came for VMMC I saw a man bring his partner here but since he was someone I just saw here and never had a chance to talk to him, I didn’t know why he brought his partner here or what they have been told.*
23. I: In your opinion do you think it’s a lot of men that come with their partners here or it’s just a few?
24. *R: It’s a few men.*
25. I: It’s a few men?
26. *R: Yes.*
27. I: What do you think makes men not to bring their partners here?
28. *R: I think its fear because it’s not a lot of people that go into a relationship with a mind of getting married to that person. Most people go into a relationship just to have fun and some do it with someone whom they clearly know is married so because of such issues one can be afraid to take such a person to the VMMC clinic for couple counseling and testing.*
29. I: Mh…
30. *R: And also another thing which also happened with me was that I didn’t know whether it was necessary to bring her or not. What I am trying to say is that if men don’t know they can bring their partners here then they feel like the partners have no part in this.*
31. I: For those that bring their partners here what do you think motivates them to do so?
32. *R: I think it’s because maybe they saw someone bring his partner here and that motivated him to also bring his.*
33. I: Mh…
34. *R: I believe so.*
35. I: How do think the partner is involved in the services that men received here at the clinic?
36. *R: I think she comes here for the HIV testing and counseling…or do women get circumcised?[laughing]*
37. I: [Laughing]… So what do you think can be done to make men bring their partners for couple counseling and testing?
38. *R: I have ever seen land Cruisers which are written VMMC and they have speakers on them with a message about VMMC. I feel that it can be good if such vehicles could be circling not just in town but also in typical villages. They can also be advertising these issues on the radio because most of us listen to the radio.*
39. I: Mh…
40. *R: I am saying all of this because what I saw here is quite different from what I was hoping to find here. The fear that I had was gone when I came here so yea, I feel that can be good.*
41. I: What are your thoughts about bringing in your partners for HIV testing at Voluntary Medical Male circumcision clinic
42. *R: When I think about it, I feel that it’s a good idea because we all need to know our status. There are some people who are lazy about coming to the hospital and get tested so this can be an opportunity for the couple to get tested together. Another thing is that it can work to the man’s advantage because the way I was before I came here and after the procedure was different because after the procedure I was failing to walk properly. So if you are with someone who loves and cares for you can be good so that she strengthens you. It’s not like you feel pain right there and then because they give you anesthesia, but still more you need someone to hold you or carry your bag for you.*
43. I: Mh…
44. *R: So I feel like it can work to the man’s advantage in that way.*
45. I: Alright. What is your opinion on integrating couple HIV counseling with Voluntary Medical Male circumcision services?
46. *R: I think it’s a good idea. Like I said couples need to know their status so it can be a good thing integrating the two because it will help couples to know their partner’s status. Another thing is that if one knows that he is HIV negative, he may have some fears and not want to get infected. But when you come alone for testing you may still have the fear while your partner plays around since she doesn’t know her status.*
47. I: Mm, what do you mean by “playing around”?
48. *R: Like having sex with other men and infect you in the long run. While if both of you got tested together and you are both negative, you can sit each other down and agree to protect yourselves.*
49. I: Mh…What do you think are the barriers and concerns on this integration?
50. *R: Even though I said couples need to know each other’s status, it’s not everyone who would be willing to bring his partner.*
51. I: Mh…
52. *R: Yea so if someone is not willing to bring his partner here then it’s a concern for him because he might think “should my partner know my status now?”*
53. I: How can we address such a concern?
54. *R: It’s like I said that people need to be sensitized either by using the radio or other means. If people have enough information while at home they will have no concerns by the time they get here.*
55. I: Mh…
56. *R: Sure.*
57. I: I would like us to discuss about sexual reproductive health services and Pills for HIV prevention: called pre-exposure prophylaxis. (PrEP) Sexual reproductive health include services that promote good sexual health and reproduction. They include but not limited to family planning, cervical cancer screening sexual transmitted infection management cervical, condom distribution and many more.
58. *R: Okay.*
59. I: Today we will only discuss family planning, Sexual transmitted infection management cervical screening and PrEP. We will look at each of these one by one. Let us start with STI services.
60. *R: Mh...*
61. I: Tell me what you have learnt about STIs when you came to the VMMC clinic?
62. *R: What I have learnt is that… firstly when I came here they screened me for STIs and after they were done they told me that if one is found with an STI they don’t perform the surgery because the wound heals slowly with such infections. I also saw other people being sent back without doing the surgery and after they had told me about this that’s when I realized why they were turned back. So in short I learnt that we need to protect ourselves from these STIs because once we have them they may prevent us from getting other opportunities like getting VMMC because some people failed to have the surgery when they were diagnosed with STIs.*
63. I: So which STIs did they say would prevent one form doing VMMC?
64. *R: Like HIV, Syphilis, gonorrhea and so on.*
65. I: Mh…So right now I want us to concentrate on the other STIs part from HIV.
66. *R: Mh...*
67. I: What did the health care workers say would happen to you if they suspected or diagnosed you with an STI?
68. *R: They said they would not perform the surgery until I get treatment for the STI. After which I could come back and they check if I could be able to have the surgery.*
69. I: So you mentioned that they tested you for infections, what tests did they perform on you?
70. *R: Gonorrhea, syphilis, HIV and also…even before performing these tests they firstly checked my Blood Pressure, then my blood sugar level.*
71. I: Mh…
72. *R: Yes.*
73. I: As an individual, what is your opinion on integrating STI services with Voluntary Medical Male Circumcision services?
74. *R: I think that’s a welcome idea because let’s say there comes a man here who has never thought about VMMC and he gets screened for STIs and gets treatment. Now the providers here are very kind enough and they tell you “since you have been diagnosed with this STI then you cannot get VMMC” So the man can wonder and ask some two or three questions which can make him curious to come for VMMC.*
75. I: Mh…
76. *R: It can also be the same with women because if women come here and get treatment, it can help to prevent infection for their partners.*
77. I: Mh…
78. *R: Sure.*
79. I: So how do you think is the best way to offer STI services at the Voluntary Medical Male Circumcision clinics? At what time point and where should the service be offered?
80. *R: I feel like it would be better for the service to be offered right here at the VMMC clinic because let’s imagine there is a clinic outside there written “we treat syphilis and gonorrhea” and people should be going to that clinic for such infection. I believe people would rather go to a traditional healer or die due to the shame that they may feel if people see them going to that clinic. While if such a clinic is in here, one can come here and people will think he is coming for VMMC when he himself knows he is coming for STI services.*
81. I: Oh okay.
82. *R: So I feel like we need to sensitize people so that more people come for such services.*
83. I: Mh…So at what time point should this service be offered?
84. *R: I feel like the best time is before the surgery. When a man comes to the clinic and tells the health care providers what he is there for, they should give him counsel then screen him for STIs and give him treatment. That is the best time.*
85. I: So the best time is after counseling, like after HIV counseling and testing?
86. *R: Like when they suspect that you have an STI and they screen you, that’s the best time to give the treatment.*
87. I: Okay, what I meant here is when should the STI services be offered? Not necessarily the treatment but the whole process including the screening.
88. *R: I feel like the best time is when they are screening for other diseases like checking for blood sugar level.*
89. I: Okay, so they should be screening at that time even if there will be couples?
90. *R: Yes, I don’t see any problem with that because these health care providers are very confidential people. They see a lot of diseases and yet they don’t break their confidentiality. So when it’s your turn to be seen by them, I don’t see any problem for them to check for the STIs.*
91. I: Okay, thank you. What is it that you would not like about this integration?
92. *R: What I may not like is the fact that the VMMC clinic may be crowded with people. You know public hospitals are always crowded but here because this is a VMMC clinic it’s not that crowded. So this integration will make people to stand in queues here at the VMMC clinic and that I don’t like.*
93. I: Okay. What do you think are the concerns and barriers for this integration?
94. *R: The concerns can be misconceptions. Say you are coming here for VMMC only and there is this integration. People will think you are just saying you are coming for VMMC when you are coming for STI services, which is a shame to most people.*
95. I: Mmm
96. *R: But still that cannot be a big issue because we don’t tell people where we are going most of the times, only that there may be some cases where people see you coming here and will think otherwise.*
97. I: Mm… so how do you think we can address this concern?
98. *R: Aah…I think we can address this by… if a person is coming here he needs to mind his own business and not tell other people that he is coming here because this is a personal issue.*
99. I: Mh…
100. *R: Another thing, say this integration has been implemented and I want to come here for VMMC, I would not just come straight here. I would pretend like I am visiting a friend here at the hospital and check if there is anyone I know who could spread the message somewhere.*
101. I: Mh…
102. *R: Sure[chuckles]*
103. I: Okay. Now I want us to talk about family planning. Tell me anything you know about family planning.
104. *R: I don’t know much about family planning because I am not married but my girlfriend and I we use condoms which I also know is part of family planning.*
105. I: Mh…
106. *R: But I have ever heard that people get injections for family planning while others take pills. So I just know that family planning is used so that a woman doesn’t fall pregnant.*
107. I: Okay. How you ever heard about family planning for men?
108. *R: Yes I ever heard but it was way back when I was in school. Back then I was young and I didn’t really understand what they were trying to mean, plus I was shy to ask the teachers.*
109. I: Mh…
110. *R: Yes, so I don’t know what is involved in the procedure.*
111. I: Mh…
112. *R: Sure.*
113. I: So there are different family planning methods like the pills that you have mentioned, injection, implanon which is inserted in the arm, there is Intra-Uterine Device, condoms and also vasectomy for men.
114. *R: Oh! Can I ask a question?*
115. I: Sure
116. *R: What is involved for one to get vasectomy?*
117. I: Ah for that we will ask the health care providers later after the interview.
118. *R: Okay.*
119. I: So all these family planning methods are there to help a woman not to get pregnant.
120. *R: Okay.*
121. I: As an individual what is your opinion on integrating Family planning in Voluntary Medical Male circumcision services/clinic?
122. *R: I think that can be a good idea.*
123. I: Mh…
124. *R: It’s the same like I explained about STI services. I feel it’s the same here because it’s only a few people that…like here you have told me about the different kinds of family planning and within minutes I have learnt a lot. So if these two could be integrated it could mean knowledge for some people. Some people don’t even have the little knowledge that I had about family planning so the integration can help to bring knowledge to the people.*
125. I: What *is it that you would like about integrating Family Planning services in Voluntary* Medical Male *Circumcision?*
126. *R: We know that if one gets an injection as a family planning method then she cannot use condoms…like in my case we use condoms and sex doesn’t feel the same as without a condom. So if say I come here and with my partner and she gets say an implanon, then we cannot be using condoms. So we can plan to come to the VMMC clinic to kill two birds with one stone.*
127. I: Okay.
128. *R: Sure.*
129. I: What is it that you would not like about integrating Family Planning services in VMMC?
130. *R: What I cannot like is what I have already mentioned that there would be too many people coming. And the other thing is that we all say time is money…so when we come here we want to be done as quickly as possible so we can go home and do other things. So if so many services are integrated here we would be spending a lot of time here, say if the whole VMMC process takes 1 hour then we can be spending 2 hours here just to hear about these other services. So it can be time consuming.*
131. I: Okay. What do you think is the best way to provide the family planning services at the VMMC clinic?
132. *R: Ah, we earlier on talked about bringing partners here. So I think if a man brings his partner and they get screened for STIs, after that it’s when we can be briefed about family planning methods.*
133. I: Mh…
134. *R: I feel that can be the right time.*
135. I: So you mentioned that the integration can be taking up much of your time. What do you think can be done so that not much of your time is taken?
136. *R: I think if maybe they can be giving us the only information that we need but not everything, then that could save us some time.*
137. I: Mh…
138. *R: Because when we come here all our minds are on the surgery. So even when they are busy screening us or giving us counsel, all we think about is the end of it all-the surgery. Like for me when I came my heart was racing when I thought about the surgery. So if it takes too much time it can be putting stress on us and all we can be thinking is “when will they do the surgery? What will happen after that?” So I feel like they do not need to take too much time explaining things.*
139. I: Mh…
140. *R: Sure.*
141. I: When did you say is the best time to offer family planning?
142. *R: I said soon after screening for STIs.*
143. I: Okay. What do you think could be the barriers or concerns on family planning and Voluntary Medical Male circumcision integration?
144. *R: I don’t think there can be any barriers because family planning is not a strange issue which can make people afraid or ashamed of. So there I don’t see any concerns or barriers.*
145. I: There are no concerns or barriers?
146. *R: No.*
147. I: Okay. Now let’s talk about cervical cancer screening. Have you ever heard about cervical cancer?
148. *R: I just heard the name but I don’t really know what it is about.*
149. I: Okay. This is a type of cancer which attacks a woman’s cervix and some clinics do screen women to check if they have it or not so they can get treatment.
150. *R: Okay.*
151. I: As an individual, what is your opinion on integrating partner cervical cancer screening with Voluntary Medical Male circumcision services
152. *R: I feel like it’s very important because when I just hear the word “cancer” I know that it’s a deadly disease. Some people even say cancer is more dangerous than HIV. So integrating such a service with VMMC is a very good idea because people are now coming for VMMC services and if they can be told about such a service I feel it can help a lot.*
153. I: Mh…What is it that would like about integrating cancer screening with Voluntary Medical Male Circumcision services?
154. *R: I have a girlfriend and this disease can also affect her. So it can be very painful to me if one day she gets sick and we go to the hospital only to learn that she has cervical cancer which could have been screened earlier at the VMMC clinic had I gone with her there and could have saved her life…*
155. I: Mh…
156. *R: That could be painful to me knowing that she could have been assisted earlier and things could not have been so bad.*
157. I: Mh…
158. *R: Sure.*
159. I: What is it that you would not like about this integration?
160. *R: Ah it’s not like there is anything I would not like about it but I would just be anxious as to what would happen if my wife tests positive.*
161. I: Okay. So when is the best time to offer this service?
162. *R: The right time is when they check blood sugar and BP. Or when they do HIV testing and counseling because this is also a disease so they have to treat it as one.*
163. I: Okay. So they should offer the service at the time they check for BP?
164. *R: Yes.*
165. I: And its partners that will be screened…?
166. *R: Yes it will be partners*
167. I: So when they are checking your BP there are men in the rooms right?
168. *R: Yes.*
169. I: So how will that work?
170. *R: Ah, since you are a couple and you get tested together for HIV so I don’t see anything strange with your partner being screened while you are there.*
171. I: Alright. So who do you think should be screened for cervical cancer at the VMMC clinic?
172. *R: It should be everyone that comes for the service because this disease can attack any woman.*
173. I: Mh… do you think could be the barriers or concerns with partner cervical cancer screening and Voluntary Medical Male circumcision integration?
174. *R: Some people are lazy and that can be one of the barriers to this integration. Another barrier would be lack of knowledge to the people.*
175. I: What can be done to address these concerns?
176. *R: It’s by continuing to sensitize people. Another thing, I see posters like those of Kwashiorkor posted on the walls in many hospitals, even here. So posting such posters on the walls or doors at the clinic can arouse interest in people about the services here.*
177. I: So these posters should be posted where?
178. *R: On the walls or doors at a clinic like this one. Like on that door there you can put a brief poster about the services and people can be curious to know more when they see such things.*
179. I: Okay. Now let us talk about PrEP. Have you ever heard about PrEP?
180. *R: PrEP or PEP?*
181. I: No, PrEP, there is an “R”.
182. *R: No I have never heard about PrEP. All I know is PEP which is a drug that one takes to prevent infection when he has had unsafe sex.*
183. I: Oh okay. So PrEP is different in a way that you don’t just take it on the days when you have unsafe sex. It’s a pill that you take once every day so you can remain HIV negative. SO PrEP is an anti-HIV drug which helps to keep HIV-negative people negative. There is a pill that one needs to take every day to prevent HIV infection.
184. *R: Oh okay.*
185. I: So how do you feel about PrEP?
186. *R: I feel it’s a good drug. You say you take the pill to prevent infection right?*
187. I: Yes.
188. *R: So it’s a really good drug [laughing] They need to be made available and if you have some you can share me some [laughing]*
189. I: [laughing] Now do you think it’s necessary for PrEP to be made available to HIV-negative men and women?
190. *R: Yes, I feel it’s necessary.*
191. I: Why do you say so?
192. *R: If there is a disease that most people are afraid of is HIV and AIDS. So if there is such a drug that can help to prevent infection of such a disease, I feel it’s a good development.*
193. I: Okay. What would be your concern if people are taking PrEP?
194. *R: My main concern would be that people would be having sex anyhow. I can give you an example with condoms: previously before condoms were made available, our parents were afraid to be sleeping around with people for fear that they would impregnate them or get infected. Right now people have sex anyhow because they know they will use condoms. This is the same with PrEP because others will be thinking “I won’t get infected so what holds me back?”*
195. I: Mh… If PrEP become available, what is your opinion on integrating PrEP with Voluntary Medical Male circumcision services?
196. *R: I think I can be very happy if they do such an integration.*
197. I: Why is that?
198. *R: It’s because PrEP is anti-HIV drugs. When we come here for VMMC, all our thoughts are on HIV prevention because they say the risk for HIV infection is lower for those that get VMMC. So the bottom line in all this is HIV prevention. If VMMC is for HIV prevention and PrEP is also for the same cause, then combining this two will be for the better good.*
199. I: Mh… How happy would you be to access them?
200. *R: I can be very happy to access them*
201. I: Mh…
202. *R: Not because I want to be having sex anyhow but because we are all human. It can happen that maybe my partner sleeps around with infected people but if I take PrEP then I still can be safe.*
203. I: Mh… How do you think PrEP would be offered in this clinic?
204. *R: I think the first thing that needs to be done is asking those that are interested to be taking the pills because other people are lazy when it comes to taking pills.*
205. I: Mh…
206. *R: Yes, so after the surgery they can ask if you have any interest in taking PrEP. That is the best time.*
207. I: So they should firstly ask after the surgery if one is interested.
208. *R: Yes, they should do that.*
209. I: To whom should PrEP be offered?
210. *R: I think it should be offered to those that have done VMMC surgery and not to everyone.*
211. I: Okay. You mentioned that your concern if people are taking PrEP would be that they would be having sex anyhow.
212. *R: Mm…*
213. I: How do you think we can address such concern?
214. *R: I think people need to be warned that the pills prevent HIV infection but if the other person has other STIs then you can get infected. So the person will be made aware that PrEP only prevents HIV infection and not STIs and that can help them to be more careful.*
215. I: Mh…
216. *R: Sure*
217. I: If you were given powers to choose and integrate services in Voluntary Medical Male Clinics, what are the services that you would think of Integrating?
218. *R: I can opt for family planning services and PrEP [chuckles]*
219. I: Why would you choose family planning?
220. *R: It’s because I am still learning about it so I can be happy if it is integrated. I*

*only knew a few methods but today I have realized that there are so many different types of them and I don’t need to be stuck up with condoms. I can have sex without condoms and yet not make a woman pregnant.*

1. I: Mh…
2. *R: So that’s why I have like family planning.*
3. I: Okay. How about PrEP?
4. *R: I have chosen PrEP because it would help to prevent HIV infection.*
5. I: Mh…
6. *R: Sure*
7. I: Thank you very much for your time. Your answers will be very helpful in improving the health service delivery at circumcision clinics.
8. *R: Mm…*
9. I: Before we close, is there anything more you would like to say?
10. *R: I have a question about PrEP?*
11. I: Mh…
12. *R: Is PrEP available in other clinics such that people are able to access it?*
13. I: No, that’s why the question was “If PrEP became available” which means it’s

not yet available.

1. *R: Okay.*
2. I: Thank you very much for talking to me today.
3. *R: Thank you.*

END

**D 43 STUDY**

**Date of Interview: 17 August 2018**

**Type of Participant: Male Index Participant**

**Interview Number: D-43-0036**

**Interviewer: I.N.**

**Total Interview Time: 29 minutes 41 seconds**

**Interview Summary:** **(from summary sheet)**

| **SERVICE TO BE INTERGRATED** | **THOUGHTS ON INTERGRATION** |
| --- | --- |
| Couple HIV Testing and Counseling | Good idea. Couples can start treatment once they know they are HIV positive. |
| STI Services | Thinks people will be encouraged if they know that they will get treatment for STIs. |
| Family Planning | A good idea. Will make more people learn about family planning. |
| Cervical Cancer Screening | Good initiative. Women will know whether they have cancer or not. |
| PrEP | A good initiative. Partners will be able to protect each other if there are trust issues between them. |
| Other Services | Thinks family planning and PrEP are the best for integration. |

**Remarks:**

**Participant understood most of the questions and was open-minded about issues.**

**Interview Text:**

1. I: Thank you for taking the time to talk with me today. I would like to ask you some questions today about the way you feel and what you think about some issues related to the service you receive here and how we can include other services in Voluntary Medical Male Circumcision (VMMC) clinics. There are no right or wrong answers to these questions. We would like to hear your opinion and your experiences in your own words. Do you have any questions before we begin?
2. *R: What service are those?*
3. I: I will be mentioning them then you should be giving your opinions on them. Okay. Do you ever talk to your care providers about how the services are provided here?
4. *R: Yes…*
5. I: Can you give me an example of a time when you managed to talk to your health care provider about the services you received here? Did you ever ask questions?
6. *R: Yes, we could ask questions for instance; “what happens when one is doing VMMC?” and also; “what benefits are there for someone who has done VMMC?” So they explained that VMMC helps one to be clean and prevent infections. For married people, it protects against cervical cancer for female partners.*
7. I: Okay, now let us talk about partner HIV testing here at the circumcision clinic? Have you ever seen a man coming together with his female partner here at the time you came for circumcision?
8. *R: No I have never seen.*
9. I: How about when you came for review; did you see any man with a partner?
10. *R: Yes I saw some.*
11. I: So what happened to such men who brought their partners to the clinic?
12. *R: I think they wanted to tested for HIV.*
13. I: Did they get the HIV test?
14. *R: Yes they did.*
15. I: Did you bring your partner the time you came for circumcision?
16. *R: No, I did not.*
17. I: Why didn’t you bring your partner?
18. *R: I came with a relative.*
19. I: Why didn’t you bring your partner?
20. *R: It’s because I didn’t know that I could bring her here.*
21. I: What do you think motivated the men who brought their partners here to do so?
22. *R: I think they wanted to know their status.*
23. I: How about those who didn’t bring their partners; what made them not to bring them?
24. *R: For some, it’s because they don’t want their partners to know their status…*
25. I: What do you think about bringing your female partner here at circumcision clinic?
26. *R: First of all, most men do not go with their partners like during pregnancy checkups because they think that is the duty or role of the women. But for coming together as a couple for circumcision, I think it is a good thing, firstly, for the wife to witness that my husband has really been circumcised and it is risky if the men come alone. Secondly, it is because once the women have seen that my husband has been circumcised means he is clean, I should also follow his footsteps and be clean.*
27. I: Okay, you as Individual, what is your opinion on integrating couple HIV counseling with Voluntary Medical Male circumcision services?
28. *R: I think it’s a good idea for couples to know their status so that if they are found HIV positive, they can start treatment or know how to protect themselves if they are found HIV negative.*
29. I: What do you think should be done so that men bring their partners to couple counseling in Voluntary Medical Male circumcision clinic?
30. *R: Encouraging them and telling them the benefits of brining their partners to the VMMC clinic.*
31. I: Who should be encouraging them?
32. *R: It should be their peers, those who already did VMMC.*
33. I: What do you think are the barriers and concerns on this integration?
34. *R: Some people would be afraid that if they bring their partner to the clinic the partner will know their status and if they are negative they may fear divorce and that people will know their status.*
35. I: What can be done to deal with such barriers?
36. *R: Telling them that life is like that…*
37. I: Telling the men?
38. *R: Yes. And also encouraging them to bring their partners since they are married anyways.*
39. I: I would like us to talk about sexual reproductive health services and Pills for HIV prevention: called pre-exposure prophylaxis. (PrEP) Sexual reproductive health includes services that promote good sexual health and reproduction. They include but not limited to family planning, cervical cancer screening sexual transmitted infection management cervical, condom distribution and many more. So today we will only discuss family planning, Sexual transmitted infection management cervical screening and PrEP. We will look at each of these one by one. Let us start with STI services. Explain to me, what you have ever learnt about STIs when you came to Voluntary Medical Male circumcision clinic?
40. *R: That STIs start if you have unprotected sex with a person who is HIV positive.*
41. I: So…. what did the staff say would happen if you were found or suspected with STIs... before circumcision
42. *R: They would give counsel then give you treatment.*
43. I: Did they say you would do VMMC if you were diagnosed with STIs?
44. *R: Yes, it happens [sounding doubtful]*
45. I: You have explained that they give treatment for the infection first, do they also do VMMC right away?
46. *R: Yes.*
47. I: Okay…as an individual, what is your opinion on integrating STI services with Voluntary Medical Male Circumcision services?
48. *R: [No response]*
49. I: Should I repeat the question?
50. *R: Yes.*
51. I: As an individual, what is your opinion on integrating STI services with Voluntary Medical Male Circumcision services?
52. *R: I think people will be encouraged that if they have STIs they will get treatment.*
53. I: Were you screened for STIs at the time you came for VMMC?
54. *R: Ah, no.*
55. I: You were not screened?
56. *R: No.*
57. I: What is it that you would like about this integration?
58. *R: It’s that many people will know their status and many can be accessing such services.*
59. I: Why do you think so?
60. *R: It’s because they will want to know their status.*
61. I: Okay so here am not only talking about HIV but other STIs. Do you know them?
62. *R: Yes. Like gonorrhea, syphilis.*
63. I: Does one already know that they have say gonorrhea?
64. *R: No they don’t know but they are caused by unsafe sex.*
65. I: What do you think can motivate people to be coming to access the STI service?
66. *R: They will live a healthy life and can reduce the spread of these infections.*
67. I: What would you not like about this integration?
68. *R: That people will be dying.*
69. I: Why will they die?
70. *R: Due to lack of such services.*
71. I: Mmm let’s say the services are available and can be accessed at the VMMC clinic. What is it that you would not like about this integration?
72. *R: Some people will not be coming to access them.*
73. I: Why is that?
74. *R: Some people don’t want others to know whether they have an infection or not.*
75. I: What do you think can be done to deal with such barriers?
76. *R: By encouraging them on the benefits of seeking such services.*
77. I: Let us talk about Family planning. Explain to me about anything that you know about family planning.
78. *R: I know that it helps couples to have a specific number of children in the family and the space between children.*
79. I: Okay so there are different family planning methods that couples can take that is others are for males while others for females. So as an individual, what is your opinion on integrating Family planning in Voluntary Medical Male circumcision services/clinic?
80. *R: That will help…ah… that can help couples to have few children*
81. I: Okay what is it that you like about this integration?
82. *R: It will help those that don’t know about family planning to start it.*
83. I: Mmm. What is it that you don’t like about this integration?
84. *R: Still some people may not be taking the service.*
85. I: Why is that?
86. *R: [chuckles] it’s because some people don’t have the specific number of children that they want to have.*
87. I: Okay. What do you think is the best way to provide the family planning services at the VMMC clinic?
88. *R: At the time when a man comes in the clinic with his partner, that’s the best time to offer them family planning because by then they already know about such services.*
89. I: Mmm. Okay you know the clinic flow at the VMMC clinic right? Can you give a brief description of the clinic flow?
90. *R: when a man comes he goes to the reception.*
91. I: After that, where does he go?
92. *R: [chuckles]*
93. I: I am asking this because I want you to tell me when in the clinic flow you think is the best time to offer family planning.
94. *R: I think in that room where they explain more about VMMC.*
95. I: Okay, who should be offered this service?
96. *R: I think anyone who wants family planning.*
97. I: Okay. What do you think could be the barriers or concerns on family planning and Voluntary Medical Male circumcision integration?
98. *R: [a bit of silence] I think for some people that don’t know about family planning may think they are being forced and may not get any method.*
99. I: How can we deal with such a barrier?
100. *R: By telling them that they have a choice whether to get a method or not.*
101. I: Okay. Now let’s talk about cervical cancer screening. Have you ever heard about cervical cancer?
102. *R: Yes*
103. I: Okay, can you tell me what you know about cervical cancer screening?
104. *R: They screen a woman’s cervix to check if they have an infection.*
105. I: As an individual, what is your opinion on integrating partner cervical cancer screening with Voluntary Medical Male circumcision services
106. *R: I feel like it will help women to know if they have the cancer or not.*
107. I: Mmm. What is it that would like about integrating cancer screening with Voluntary Medical Male Circumcision services?
108. *R:Many women will be coming for screening*
109. I: Mmm. would you bring your partner for screening?
110. *R: Yes I would.*
111. I: What is it that you would not like about this integration?
112. *R: [silence]*
113. I: There is nothing?
114. *R: [chuckles]*
115. I: What is it that you would not like about this integration? Or what do you think could be the problem with this integration?
116. *R: I don’t see any problem there.*
117. I: Okay. So when is the best time to offer this service?
118. *R: The right time is when they come to be checked.*
119. I: Each time a woman comes to be checked?
120. *R: No at the VMMC clinic there are some procedures that are followed.*
121. I: What procedure are those?
122. *R: There are questions which they ask regarding that.*
123. I: Mm, so when a person comes to be asked such questions that when she should be screened?
124. *R: Yes.*
125. I: Which room should they be screening in?
126. *R: It should be a private room.*
127. I: Why?
128. *R: Because some people may be shy and need some privacy.*
129. I: Mmm so let’s say a man needs VMMC and comes with his partner who also wants cervical cancer screening. When should the screening be done?
130. *R: Ah, while the man goes for VMMC then the woman can be going for cervical screening.*
131. I: Okay. Now let us talk about PrEP. Have you ever heard about PrEP?
132. *R: PrEP or PEP?*
133. I: No, PrEP, there is an “R”.
134. *R: No I have never heard about PrEP. All I know is PEP which is a drug that one takes to prevent infection when he has had unsafe sex.*
135. I: Oh okay. So PrEP is different in a way that you don’t just take it on the days when you have unsafe sex. It s a pill that you take once every day so you can remain HIV negative. SO PrEP is an anti-HIV drug which helps to keep HIV-negative people negative. There is a pill that one needs to take every day to prevent HIV infection.
136. *R: Oh okay.*
137. I: So how do you feel about PrEP?
138. *R: I feel it’s a good drug. You say you take the pill to prevent infection right?*
139. I: Yes.
140. *R: So it’s a really good drug [laughing] They need to be made available and if you have some you can share me some [laughing]*
141. I: [laughing] Now do you think it’s necessary for PrEP to be made available to HIV-negative men and women?
142. *R: Yes I feel it’s necessary.*
143. I: Why do you say so?
144. *R: If there is a disease that most people are afraid of is HIV and AIDS. So if there is such a drug that can help to prevent infection of such a disease, I feel it’s a good development.*
145. I: Okay. What would be your concern if people are taking PrEP?
146. *R: My main concern would be that people would be having sex anyhow. I can give you an example with condoms: previously before condoms were made available, our parents were afraid to be sleeping around with people for fear that they would impregnate them or get infected. Right now people have sex anyhow because they know they will use condoms. This is the same with PrEP because others will be thinking “I won’t get infected so what holds me back?”*
147. I: Mh... If PrEP become available, what is your opinion on integrating PrEP with Voluntary Medical Male circumcision services?
148. *R: I think I can be very happy if they do such an integration.*
149. I: Why is that?
150. *R: It’s because PrEP is anti-HIV drugs. When we come here for VMMC, all our thoughts are on HIV prevention because they say the risk for HIV infection is lower for those that get VMMC. So the bottom line in all this is HIV prevention. If VMMC is for HIV prevention and PrEP is also for the same cause then combining this two will be for the better good.*
151. I: Mh... How happy would you be to access them?
152. *R: I can be very happy to access them*
153. I: Mh…
154. *R: Not because I want to be having sex anyhow but because we are all human. It can happen that maybe my partner sleeps around with infected people but if I take PrEP then I still can be safe.*
155. I: Mh… How do you think PrEP would be offered in this clinic?
156. *R: I think the first thing that needs to be done is asking those that are interested to be taking the pills because other people are lazy when it comes to taking pills.*
157. I: Mh…
158. *R: Yes, so after the surgery they can ask if you have any interest in taking PrEP. That is the best time.*
159. I: So they should firstly ask after the surgery if one is interested.
160. *R: Yes they should do that.*
161. I: To whom should PrEP be offered?
162. *R: I think it should be offered to those that have done VMMC surgery and not to everyone.*
163. I: Okay. You mentioned that your concern if people are taking PrEP would be that they would be having sex anyhow.
164. *R: Mm.*
165. I: How do you think we can address such concern?
166. *R: I think people need to be warned that the pills prevent HIV infection but if the other person has other STIs then you can get infected. So the person will be made aware that PrEP only prevents HIV infection and not STIs and that can help them to be more careful.*
167. I: Mh…
168. *R: Sure*
169. I: If you were given powers to choose and integrate services in Voluntary Medical Male Clinics, what are the services that you would think of Integrating?
170. *R: I can opt for family planning services and PrEP [chuckles]*
171. I: Why would you choose family planning?
172. *R: It’s because I am still learning about it so I can be happy if it is integrated. I only knew a few methods but today I have realized that there are so many different types of them and I don’t need to be stuck up with condoms. I can have sex without condoms and yet not make a woman pregnant.*
173. I: Mh…
174. *R: So that’s why I have liked family planning.*
175. I: Okay. How about PrEP?
176. *R: I have chosen PrEP because it would help to prevent HIV infection.*
177. I: Mmm
178. *R: Sure*
179. I: Thank you very much for your time. Your answers will be very helpful in improving the health service delivery at circumcision clinics.
180. *R: Mm…*
181. I: Before we close, is there anything more you would like to say?
182. *R: I have a question about PrEP?*
183. I: Mmm
184. *R: Is PrEP available in other clinics such that people are able to access it?*
185. I: No, that’s why the question was “If PrEP became available” which means it’s not yet available.
186. *R: Okay.*
187. I: Thank you very much for talking to me today.
188. *R: Thank you.*

END

**D 43 STUDY**

**Date of Interview: 20 August 2018**

**Type of Participant: Male index participant**

**Interview Number: D-43-0037**

**Interviewer: I.N.**

**Total Interview Time: 50 minutes 01 seconds**

**Interview Summary :( from summary sheet)**

| **SERVICE TO BE INTERGRATED** | **THOUGHTS ON INTERGRATION** |
| --- | --- |
| Couple HIV Testing and Counseling | Thinks it’s a good idea since people especially who are unfaithful to their wives will start thinking twice in afraid of being found positive in the presence of their wives. |
| STI Services | Thinks it’s a good combination because they are both concerned with sexual intercourse, and both services will be accessed at the same point |
| Family Planning | He welcomes the idea but thinks people won’t feel comfortable to receive these services in presence of opposite gender |
| Cervical Cancer Screening | Supports the idea since women will be protected from cervical cancer |
| PrEP | Likes the idea since it will protect the positive partner from HIV and giving them chance to take care of children, in case the positive one dies |
| Other Services | Thinks all services should be integrated into VMMC as they fuse well |

**Remarks:** The participant was open and confident. He could explain clearly and more.

**Interview Texts:**

1. I: Thank you for taking the time to talk with me today. I would like to ask you some questions today about the way you feel and what you think about some issues related to the service you receive here and how we can include other services in Voluntary Medical Male Circumcision (VMMC) clinics. There are no right or wrong answers to these questions. We would like to hear your opinions and your experiences in your own words. Do you have any questions before we begin?
2. *R: Ah no, there no questions.*
3. I: Do your ever talk to your care providers about how the services are provided here at the VMMC clinic?
4. *R: No*
5. I: Why don’t you discuss with the providers?
6. *R: Sometimes, what I have noted is that the ones providing the services are women; as for me, I have grown up a village life and village life, I know it well. It looks easy because we are in town and we are learning new life; like knowledgeable people, maybe town or English life but during circumcision process, when you are on the queue, it is a man who comes and get you but upon reaching inside, you find that there are women but your mindset thinks it is the man who is going to operate you but you just get surprised that all men leave and only women remain. To me I don’t think there is a problem but to a person from the village, telling him that it is women who operate…ah, they cannot come. Many cannot go.*
7. I: Ehm... when you came for check-up, did you talk to the service provider about anything?
8. *R:* *Yes, I did, after the service and I went home I started bleeding so when I came for review, I told the service provider. First I didn’t come because I couldn’t walk due to the pain and swollen but then after some days I came to talk to the*
9. I: Now let us talk about partner HIV testing here at the circumcision clinic. Tell me what happens when a man brings in a spouse at the Voluntary Medical Male circumcision clinic…
10. *R: What I saw, as for me, the way I socialize and how the wife sees it, I don’t think there is any problem to bring my wife, but for most men, they are afraid to bring their partners, since they don’t want them to know their HIV statuses*.
11. I: How is the partner involved in the services that men received here at the clinic?
12. *R: But I have never seen anyone bring their partner*
13. I: If you brought your spouse, what motivated you to bring her in?
14. *R:* *I believe, it’s a matter understanding of each other, they have nothing to be afraid of because they know they are royal to each other*
15. I: In your opinion, what are your thoughts about bringing in your partners for HIV testing at Voluntary Medical Male circumcision clinic?
16. *R: It is a very good idea. Sometimes it happens in a family especially us males, especially for those who travels a lot, like going in Mzuzu or Zambia they like shaving sex with other women, so it’s good to go together for testing to know each other’s status. Of course it can be hard at first when one partner is found positive, but with time you understand and find the way forward like how will the other partner can take care of the children in case of death of the affected partner.*
17. I: What do you think can be done to make men bring their partners here for HIV testing and counseling?
18. *R: There is a big work to be done. People should be sensitized, maybe through radios, books, so that people should understand that it should be like that*.
19. I: You as Individual, what is your opinion on integrating couple HIV counseling with Voluntary Medical Male circumcision services.
20. *R: It’s a good thing, because if you do that frequently, if one partner was unfaithful, it will make them to think twice, in afraid of being found positive in front of their partner. For example, if I come here with my wife and I have done both circumcision and HIV testing, and after three months my wife says we should go for HIV testing again, I will start being careful not to be sleeping with other women in afraid of being found positive in my wife’s presence.*
21. I: What do you think are the barriers and concerns on this integration?
22. *R: Barriers can be there, especially from village people, it’s hard to convince, them to bring their partners to VMMC.*
23. I: What do you think should be done to overcome these concerns and barriers to couple counseling in Voluntary Medical Male circumcision clinic?
24. *R: They need a good strategy for example using dramas, radios as I already said*
25. I: Now I would like us to discuss about sexual reproductive health services and Pills for HIV prevention: called pre-exposure prophylaxis (PrEP). Sexual reproductive health includes services that promote good sexual health and reproduction. They include but not limited to family planning, cervical cancer screening sexual transmitted infection management cervical, condom distribution and many more. Today we will only discuss family planning, Sexual transmitted infection management cervical screening and PrEP. We will look at each of these one by one. Let us start with: STI services. Explain to me, what you have ever learnt about STIs when you came to Voluntary Medical Male circumcision clinic?
26. *R: What* *I have leant is, circumcision does not prevent STIs 100%, it just reduces the risk of STIs because when your penis is has no foreskin then skin at the tip becomes a bit hard making it hard for viruses to penetrate.*
27. I: What did the staff say would happen if you were found or suspected with STIs?
28. *R: They didn’t say anything*
29. I: Or maybe let me ask in this way, if you go for circumcision and you are diagnosed with STI what happens next?
30. *R: I just heard from someone who said, if you are diagnosed with STI you with first be treated of the infection. Before circumcision*
31. I: As an individual, what is your opinion on integrating STI services with Voluntary Medical Male Circumcision services?
32. *R: I think it’s a good combination because they are both concerned with sexual intercourse, so if organizations or the government can be integrated it can help since both services will be accessed at the same point. For example, if I am diagnosed with STI and am told to go to section B, I will just go home and not in the section B but if the service is in here I will force myself to receive the service.*
33. I: How do you think is the best way to offer STI services at the Voluntary Medical Male Circumcision clinics like terms of place, should it be in the hospital outside or in a private room?
34. *R: As Malawians, we shouldn’t just be copying other people’s cultures in the name of democracy, as in our culture it is not good for these services to be provided at a public place rather in a private room or place. Circumcision includes children with like 13 years,18 years, as for me am 38 years am almost a man now, so if the service is done at a public place it can be shameful for me so it should be done in a private room*
35. I: But at what stage in the process of circumcision should this service be provided, before or after circumcision.
36. *R: When you are diagnosed with infections, for example ARV you are supposed to be given to you to take home but for infections like candidiasis it will disturb your mindset so it should be given before circumcision the services should be provided before circumcision.*
37. I: What is it that you do not like integrating STI services in Voluntary Medical Male circumcision services?
38. *R: Respect, it’s shameful, the combination of women and men receiving these services at one place may not sound well for most people. It’s unnatural for most people. We come from same areas with these women and we know each other and they know our wives, so it’s hard to receive like for say STI drugs in their presence, women talk too much, they don’t keep a secret. So people won’t feel comfortable*
39. I: What do you think are the barriers and concerns on this integration of STI services with Voluntary Medical Male circumcision services?
40. *R:* *Ah… I don’t think there are any barriers.*
41. I: Now let us talk about Family planning. Explain to me about you know about family planning (vasectomy for men and family planning for female partners?
42. *R: I am married with two kids; I was supposed to have four children but two are dead. The reason is my first born was born in September 2001, because of ignorance about family planning, I gave my wife another pregnancy and she gave birth in 2003. After people laughed at us, they taught us about family planning. My wife went to the hospital and she was told to be given an injection not pills. She ignored the doctor’s advice and started using pills. She started to have stomach aches until we went to the hospital where we were told that she should be using injection, up to now she is okay and we have other children together, our youngest was born in 2015*
43. I: Do you know that men also do family planning like vasectomy?
44. *R: Yes, I know but I have never done it before.*
45. I: As an individual, what is your opinion on integrating Family planning in Voluntary Medical Male circumcision services/clinic?
46. *R: Ah, as for me, since family planning is optional, they should be asked first if they want the procedure, I think it’s a good idea because you won’t force them, you will counsel them first, maybe you ask them how many kids they, have maybe 5 so you ask them if they are willing to do vasectomy and go ahead with the procedure, they should be comfortable and in total agree.*
47. I: What would make you not want integrating Family Planning services in Voluntary Medical Male Circumcision Services?
48. *R: Nothing.*
49. I: How do you think is the best way to offer family planning services within Voluntary Medical Male circumcision services clinics?
50. *R: I was thinking the right time is the time a person has just arrived at the clinic. You may even ask for family planning service before circumcision but it should be done in the same room.*
51. I: What do you think could be the barriers or concerns on family planning and Voluntary Medical Male circumcision integration?
52. *R: Both barriers and concern are there, for example, when I do both family planning procedure and circumcision and I tell people, people won’t understand especially at the village if I tell them that “I already have 3 kids and they are enough for me.” People won’t understand that it is optional, they will think maybe doing circumcision will make you barren, you may marry a girl and fail to have kids*
53. I: How do you think these concerns and barriers can be addressed?
54. *R: It can be addressed as I already said; people should be sensitized especially from the village. At least because of high education levels, people from urban areas easily understand these things, people at the village are hard because of traditions. Have a good strategy, each through dramas so the people should feel comfortable. People should know that circumcision is optional*
55. I: Now let us talk of cervical cancer screening. Explain to me what you know about cervical cancer screening
56. *R: I am from Zomba where they are mixed cultures, (Names of cultures). My father is (Name of Tribe) while my mother is (Name of Tribe), so from where I come from many of my friends did traditional circumcision during holidays. From what I saw when they get circumcised they loved women so hated it, so I was not circumcised, but when I come to town, one of my friend’s wife was diagnosed with cervical cancer so the doctor advised my friend to do circumcision since he said circumcision reduces the risk of women to get cervical cancer so he did. I have two kids, one is in standard 8, so I was thinking what if my wife is also diagnosed with cervical cancer and I can’t afford the drug and she dies and leave me with the kids. So I decided to get circumcised to prevent problems like this one.*
57. I: As an individual, what is your opinion on integrating partner cervical cancer screening with Voluntary Medical Male circumcision services
58. *R: it’s a good idea because you will be going together*
59. I: What is it that would make you not want integrating cervical cancer screening with Voluntary Medical Male Circumcision?
60. *R: Nothing.*
61. I: How do you think is the best way to offer Cervical Cancer screening services within Voluntary Medical Male circumcision clinics?
62. *R: What I can see is... Doctors and nurses should be friendly to clients, for say when client alive that should make sure that they should be problem for people to feel comfortable*
63. I: How about place, where should it be, inside, outside or where
64. *R: I think if it can be the in a private room not outside, and not in another room because people thinks too much. For say you are done with circumcision and you are told to go in another room for cancer screening in another room, people can be thinking that you have been diagnosed of HIV and you are going for ARV. Segregation for HIV people is still there and I don’t think it will end. It should be in the same room that when you are done you should be going straight home*
65. I: In the procedure of circumcision, what time do you think it should be offered?
66. *R: I was thinking after circumcision, before the pain starts, that should be the time, just after circumcision, then you go with your wife into the screening room and after that you go straight home*
67. I: What do you think could be the barriers or concerns with partner cervical cancer screening and Voluntary Medical Male circumcision integration?
68. *R: I don’t think there can be any barrier because when leaving home, you know exactly what you are going there for. So I don’t think there can be any barriers*
69. I: Now let us discus about PrEP. Have you heard about PrEP
70. *R: Yes*
71. I: Tell me what you know about PrEP.
72. *R: I heard from my friend, PrEP is a drug you take after sleeping with an HIV person. I have a friend who slept with a certain girl who was born with HIV. When he was told by his friends, he went to the hospital and he was given the drug*.
73. I: That drug is called PEP, PrEP is another drug. PrEP is anti-HIV medicine that keeps HIV negative people from being infected. There is a single pill that is taken once daily, and if you take it regularly, it is highly effective at prevention people from being infected. Now, how do you feel about Prep?
74. *R: you said it is a drug that you take before intercourse with an HIV positive person to protect yourself.*
75. I: Is it necessary to make PrEP available to HIV- men and women?

*R: Yes, very much.*

1. I: Why?
2. *R: If PrEP is not available it’s given you will get HIV and this may lead to death leaving your children without any one to support them. But if prep is available you can even give birth, so if you are using this drug and you are on ARV, you still can’t live longer so if you die, your partner can take care of the child*
3. I: If PrEP become available, what is your opinion on integrating PrEP with Voluntary Medical Male circumcision services?
4. *R: It’s a good thing because, when you come to the hospital with your wife and one of you is positive, the doctors or nurses should say that the drug is available that can be used to prevent cross infection, you can be having intercourse even having kids without any problem*
5. I: How can u feel if you can have the access to PrEP?
6. *R: I can feel good because even if one of the partner ids positive the other one is protected*
7. I: How do you think PrEP would be offered in this clinic?
8. *R: After circumcision and it should be offered like any other drug at the hospital and it should be offered if one partner is found positive.*
9. I: What concerns would you have about people taking PrEP?
10. *R: there are no concerns, because even if one partner is positive the other one is protected*
11. I: Let us now talk about other services. If you were given powers to choose and integrate services in Voluntary Medical Male Clinics, what are the services that you would think of Integrating?
12. *R: What I can choose is respect for human rights, because we were born different, we were raised differently and cultured differently. Some people don’t find a problem to be naked or to be dancing in the presence of women. Coming to a clinic like here, undressing women anyhow is not good; yes, like in a labor room it is necessary even a male doctor can undress a woman. But as for me I can’t get circumcised by a woman, if I find a woman in the circumcision room I can turn back home, so maybe people should be given option on whether they are comfortable or not to be treated with which gender. Maybe there should be two room one with a woman for female clients and the other with a man for male clients.*
13. I: How about the services I mentioned which ones can you choose to integrate with VMMC?
14. *R: I can choose family planning, cervical cancer screening and all other services*
15. I: Explain to me what the reasons are for your choice.
16. *R: This* can help to save time since all the services will be provided at one place
17. I: How do you think these services should be offered in the clinic
18. *R: It s*hould be provided in a private room.
19. I: Thank you for taking your time to discuss with me today. Your answers will be very helpful in improving the health service delivery at circumcision clinics. Before we close, do you have anything to say?
20. *R: I have a question, where is the interview coming from? Is it from the government?*
21. I: *It is from (Name) hospital but it is a government initiative*
22. *R: Okay.*
23. I: Again, thank you so much for taking your time to speak with me.
24. THE END

**D43 STUDY**

**Date of Interview: 04 September 2018**

**Type of Participant: Male Index Participant**

**Interview Number: D-43-0046**

**Interviewer: I. N.**

**Total Interview Time: 24 minutes 02 seconds**

**Interview Summary:** **(from summary sheet)**

| **SERVICE TO BE INTERGRATED** | **THOUGHTS ON INTERGRATION** |
| --- | --- |
| Couple HIV Testing and Counselling | Thinks its good development if you involve your partner they will know about your health and when counselled they would know how to take care of the wound. |
| STI Services | Thinks it’s a good idea because those things are related because VMC is like reducing the chances of contracting STI. |
| Family Planning | Thinks it is not a good idea; we already have clinics that offer family planning services hence VMC to focus on the services that they are providing. |
| Cervical Cancer Screening | Thinks it’s not a good idea because it’s not necessary since you provide the counselling. If anything, the clients can be referred to another hospital to do the screening. |
| PrEP | Likes the idea of integrating PrEP with VMMC services because if you take prep you are safe, though people will be having sex anyhow. |
| Other Services | Thinks integrating VMC services with STI management then its fine |

**Remarks:**

The participant was confident but not open enough. He did not see any link between VMMC with PrEP.

**Interview Texts:**

1. I: Thank you for taking your time to talk to me today, I would like to ask you some questions on how you feel and the services you received here and how we can include other services in the voluntary male circumcision clinics. There are no right or wrong answers to these questions. We would like to hear your experiences in your own words. Do you have any questions before we begin?
2. *R: No. I don’t have.*
3. I: do you ever talk to your service providers about how the services are provided here?
4. *R: No. I have never talked to any health service provider.*
5. I: The time you came for checks ups here, did you ever give feedback to your service providers ….eeehhh….how things were going and anything you experienced after the circumcision?
6. *R: Ah...when I went back for the follow ups...for the checkups…hmm we didn’t have time to discuss about how the services were offered but... hmm yes I would say we didn’t have time for that because I went straight into the checkup room and after the checkup I went back home.*
7. I: Okay. But have you experienced some changes or differences before you were circumcised and after the circumcision?
8. *R: Differences in terms of…?*
9. I: The feelings or any experiences after the circumcision, maybe a complement about it…
10. *R: I would say it’s good because mm… the benefits of circumcision are that they help to protect women from cervical cancer and the other thing is that you reduce the chances of contracting HIV/AIDS but otherwise I wouldn’t say I have experienced something unusual about the circumcision.*
11. I: Ah…okay, let us now talk about couple HIV testing and counselling; tell me what happens when a man brings a wife here at the voluntary male medical circumcision clinics?
12. *R: I never had any experience or saw any man bringing a woman to the circumcision clinic because I only came for the circumcision here so yeah I did not have such an experience.*
13. I: The time you went for circumcision; you didn’t see any man with his partner?
14. *R: No. I did not see.*
15. I: How is a partner involved in the services that are provided here at the clinic?
16. *R: Ah, I wouldn’t say anything about that, because the day I went for the circumcision I just said that I didn’t any man bringing a woman to the clinic so I wouldn’t say I know anything about women involvement in the circumcision clinics.*
17. I: Okay. Let’s assume you take your partner to the clinics, there are some men who brings their partners to the clinics, what do you think motivates such men to bring their spouses to the circumcision clinics?
18. *R: Ah... the motivators would be maybe they love their partner that much and they would want to involve them in everything they are doing in their life so they may involve their partners but then any special motivators to that like myself I wasn’t motivated in any way to come for circumcision so I wouldn’t say there are motivators to bring your wife to the clinics.*
19. I: Okay. If you did not, what made you not to bring your partner at the circumcision clinic?
20. *R: When I was coming for the circumcision I was single, I had no girlfriend, no wife so I couldn’t bring any one with me to the circumcision.*
21. I: So in your opinion, what are your thoughts about bringing your spouses to the VMMC clinics for HIV testing?
22. *R: In my opinion, I think it’s a good idea it’s a good thing because mm… if you involve your partner, if you have a girlfriend or maybe you are married and then you bring them to the circumcision clinic, they will know everything that happens there and if you will be counselled, the given instructions on wound care, how long it takes for the wound to heal, and they give you a period whereby you are not supposed to have sex so it would be easy for the couples to understand each on the instructions given by the service providers so it’s a good idea, it’s a good thing.*
23. I: Okay. So what do you think can be done to make men bring their partners for HIV testing and counselling at the circumcision clinics?
24. *R: I think it’s a matter of sensitization, uuuhmm the service providers should sensitize the communities on aaahh how good it is to involve your partner when you are going for the circumcision service because mostly people are not aware of the importance of circumcision and the essence of partner involvement since mostly circumcision is for men so women do not really see themselves playing a role in this service.*
25. I: So you as an individual, what is your opinion on integrating couple HIV testing and counselling with VMMC services?
26. *R: Ah it is good, it is a good idea because if you integrate HIV testing and voluntary male medical circumcision aaahhh there is….it’s more like a saying which is there for circumcision which everybody talks about that when you are circumcised you are protected 60% from contracting HIV so some misquote that so involving both partners will make them understand that the 40% chances are still there for them to contract the virus so condom use should not be ignored so I think the integration of these services will be a good idea.*
27. I: But what do you think are the barriers and concerns of this integration?
28. *R: Ah…. the barriers will maybe be misinformation, if people are not well informed about the essence of integrating the two, there will be a barrier of implementation, service providers will not find it easy to implement because people may not be coming for HIV testing and circumcision at the same time so the only barrier that I see is about that information deficit to the clients so if you can the information I think that’s the only barrier yes, information deficit.*
29. I: So what do you think should be done to overcome the barrier you just mentioned?
30. *R: Well as I have said, the barrier is about information deficit so what needs to be done is just to sensitize the communities, inform the people what needs to be done and with that I think that barrier is not really a big deal.*
31. I: Now I would like us to discuss about sexual reproductive health services, pills for HIV prevention called pre-Exposure Prophylaxis. sexual reproductive health includes services that promote good sexual health and reproduction they include but not limited to family planning, cervical cancer screening, Sexually Transmitted Infections management and condom distribution and many more. Today we will only discuss about family planning, cervical cancer screening, Sexually Transmitted Infections management and PrEP. We will look into each one of these one by one, so let’s start with STI services. Explain to me what you ever learnt about STIs when you came to VMMC clinic.
32. *R: I did not learn anything about STIs when I came for circumcision service.*
33. I: Okay. What did the stuff say if you are suspected or diagnosed with STIs?
34. *R: if you are found or suspected of STIs you will not be circumcised because aaahh they wound doesn’t heal fast because there are opportunistic infections that come in because usually when you have an STI your immune system is weakened and infections come in on the wound making it not to heal fast.*
35. I: So as an individual what is your opinion in integrating STI services with the VMMC services?
36. *R: It is a good idea because those things are related in the sense that the benefit of circumcision is to reduce chances of contracting STIs such as HIV so yeah it’s a good idea.*
37. I: What would you not like about the integration?
38. *R: Mm… I wouldn’t say I wouldn’t like anything, everything is okay for me.*
39. I: what do you think is the best way to offer STI services at the voluntary male medical circumcision clinics?
40. *R: I would say, when you go for circumcision at the VMMC clinics, the first thing is counselling, and then you go for the HIV test so the HIV testing and the services you can receive from the STIs are very similar so I think that’s the best time before the circumcision as you are receiving the counseling for HIV testing you can as well integrate STI service.*
41. I: So what do you think are the barriers and concerns of integrating the STI services with the VMMC services?
42. *R: Mm… barriers mh, I don’t think there are any barriers, because the benefits outweighs the barriers, barriers I can’t point out now so integrating STI teachings during the HIV counselling I don’t think there can be any barriers.*
43. I: Now let us discuss about family planning, how do you understand by the term family planning? Or what comes to your mind when you hear the term family planning?
44. *R: Family planning are various methods* *that are used to control the rate at which families are making babies and I think there are a lot of methods that are put in place to control the rate at which babies are born in families.*
45. I*:* Can you just give me some of the methods of family planning for men and women?
46. *R: Condom use, vasectomy, Norplant for women, tube legation and injection.*
47. I: As an individual, what is your opinion about integrating family planning in the VMMC clinics?
48. *R: Ah, I don’t see the essence of integrating family planning in the VMMC clinics, we already have clinics that offer family clinics services so I think the best way is for VMMC is to focus on the services they are offering rather bringing services that are also wider on their own which will end up ruining the quality of services they are already offering.*
49. I: let us look at cervical cancer screening then, tell me anything you know about cervical cancer screening.
50. *R*: *Ah, I just know that women are encouraged to go for cervical cancer screening, because screening is the process whereby a client goes to the hospital to screen her if she has cervical cancer or not*.
51. I*:* So do you think it’s necessary for women to go for cervical cancer screening?
52. *R*: *Yes, it is necessary because screening will help in early detection of the cancer which makes it easier to be controlled.*
53. I: So as an individual, what is your opinion on integrating partner cervical cancer screening with VMMC services?
54. *R: Mm… I don’t really see the importance of doing that, otherwise the question you asked previously was if it’s important for spouses to escort each other to the VMMC clinics which I said when you go there you learn the benefits of circumcision which is to protect women from cervical cancer so it’s already tackled in some aspects of VMMC already but to bring cervical cancer as another service in the VMMC will just ruin the service quality currently offered here, focus on your services.*
55. I: So you don’t like the integration?
56. *R*: *The integration of cancer screening and VMMC services, yes I don’t like it. You can always refer cancer clients from VMMC clinics to cancer clinics.*
57. I: Ah, now let us discuss about PrEP, have you ever heard about PrEP?
58. *R: Yes, I have.*
59. I: tell me what you know about PrEP.
60. *R: It is a drug that is taken by an HIV negative person so that it should protect the person from contracting HIV even if this person has sexual intercourse with an HIV positive person*
61. I: So are these pills available?
62. *R: No. I have never seen PrEP in pharmacies or hospitals.*
63. I: How did you know about PrEP?
64. *R: I think I read from the internet.*
65. I: So it’s found on the internet?
66. *R: Yes, the information is there on internet that there is PrEP.*
67. I: Is it necessary to make PrEP available to HIV negative men and women?
68. *R: Yes, it is necessary.*
69. I: What are the reasons for your point?
70. *R: I would say it’s necessary because hmmm HIV is everywhere you can never know sometimes even if you use a condom it may burst you contract the disease so if you take PrEP effectively, you remain protected so I think it’s good though somehow it may lead to immoral sexual behaviors because they will no longer be afraid of the virus.*
71. I: So if PrEP become available, what is your opinion on integrating PrEP with VMMC services?
72. *R: Ah, actually I see no link between PrEP and VMMC, so that integration is not necessary unless it’s done at the VCT or ART clinics because that’s the thing that when you go to the VCT and you test negative and your spouse tests positive, they can be encouraged to take PrEP but for VMMC and PrEP I don’t see any linkage for these services.*
73. I: So you wouldn’t like the integration of VMMC and PrEP?
74. *R: Not that I wouldn’t like, but I don’t see the need of doing that unless people see that need.*
75. I: Let us look at the other part which is other services, if you are given the power to integrate other services to the VMMC services, what are the services you would think of integrating?
76. *R: I would say integrating STI management with VMMC services is okay. Aaah other services hmmm what other services uuhhmm but yeah I think STI management and VMMC services is okay.*
77. I: How about other services besides which we talked about here?
78. *R: Mm… other services, aaah no I can’t figure out of extra services now maybe if you can come back later with the interviews I would have figure something out. Otherwise I would encourage the VMMC clinics to focus on the services they offer otherwise they end up offering poor services if they bring in more services.*
79. I: So you have chosen STI management, but can you explain to me the reasons for your choice?
80. *R: I said it before, STI screening and management and HIV testing and counseling, those things are similar and when you go for circumcision you first under the process of HIV counselling so it’s easy to integrate the two…. they are more less like the same thing.*
81. I: But how do you think these things should be offered in the VMMC clinics?
82. *R: They should be offered just like the same way they do with the HIV counselling, that’s the best time when the STI management can be integrated within the VMMC clinics*
83. I: Ah, thank you for taking your time to discuss with me today, your answers will be helpful in improving the health service delivery at circumcision clinics. Before we close, do you have anything to say?
84. I: Once again thank you for taking your time to speak with me today.
85. *R: Thank you.*
86. THE END.

**D43 STUDY**

**Date of Interview: 05 September 2018**

**Type of Participant: Male Index Participant**

**Interview Number: D-43-0049**

**Interviewer: I.N.**

**Total Interview Time: 25 minutes 32 seconds**

**Interview Summary:** **(from summary sheet)**

| **SERVICE TO BE INTERGRATED** | **THOUGHTS ON INTERGRATION** |
| --- | --- |
| Couple HIV Testing and Counseling | Thinks its good development because I think it’s important, and it’s good that as a couple should be going for testing at the same time |
| STI Services | Thinks it’s a good idea because the good thing is that they should be testing before circumcision to know what to be done next. |
| Family Planning | Thinks it is not a good idea because a person should make her own special day to get the service and it can be a lot of work and tiresome for one to go for VMMC and also go for family planning. |
| Cervical Cancer Screening | Thinks it’s a good idea because it helps one to know how to prevent cancer after knowing that she doesn’t have cancer. |
| PrEP | Like the idea of integrating PrEP with VMMC services because there wouldn’t space at the hospital for one to get HIV infected. |
| Other Services | Thinks after a person is circumcised, he needs to be given money because most of their work is on hold. |

**Remarks:** Participant was relaxed but provided short answers. In addition, it was difficult for the participant to grasp the real gist of the question.

**Interview Text:**

1. I: Thank you for taking the time to talk with me today. I would like to ask you some questions today about the way you feel and what you think about some issues related to the service you receive here and how we can include other services in Voluntary Medical Male Circumcision (VMMC) clinics. There are no right or wrong answers to these questions. We would like to hear your opinion and your experiences in your own words. Do you have any questions before we begin?
2. *R: No.*
3. I: Do your ever talk to your care providers about how the services are provided here?
4. *R: We have never talked about it.*
5. I: You did not go for checkup so that they ask you how you are doing.
6. *R: They came, and checked how we were doing and they told us to continue taking care of the wound.*
7. I: Did they explain to you how VMMC is offered, on how it works on the whole process
8. *R: Yes, they did.*
9. I: Can you give me an example of a time when you managed to talk to your health care provider about the services you received here?
10. *R: They came to do a campaign, took us in their cars and went to (Name of Hospital) when we were there they cancelled us saying that after circumcision you have to take of the wound with salt,*
11. I: Now let us talk about partner HIV testing here at the circumcision clinic. Tell me what happens when a man brings in a spouse at the Voluntary Medical Male circumcision clinic
12. *R: Something related to HIV you say?*
13. I: Yes.
14. *R: They check your HIV status, whether you have it or not*
15. I: Have you seen any man taking his wife
16. *R: No, I have never seen them, I only see men going alone*
17. I: What makes them not to take their wife when going for VMC
18. *R: I think they want the wife to be doing other work at home*
19. I: For those who do not take their wife when coming for VMMC, what do you think makes them to do so?
20. *R: I think because of worry that if one of them is found negative and the other positive then the relationship will be destroyed*
21. I: What about those who bring their wives, what makes them to do so?
22. *R: The men want when they go together they should all hear it for themselves how they are*
23. I: In your opinion, what are your thoughts about bringing in your partners for HIV testing at Voluntary Medical Male circumcision clinic
24. *R: I think it’s important, and it’s good that as a couple they should be going for testing at the same time*
25. I: What do you think can be done to make men bring their partners here for HIV testing and counselling?
26. *R: What can happen is that, these people should be civic educated, so that they know the correct information*
27. I: You as Individual, what is your opinion on integrating couple HIV counseling with Voluntary Medical Male circumcision services.
28. *R: Like adding on to the services?*
29. I: Yes, so what do you think about the integration?
30. *R: For people to be going as a couple for testing, then it’s important, they should be taking these people on the clinic transport and not on their own so that they shouldn’t have trouble in transportation.*
31. I: What do you think are the barriers and concerns on this integration?
32. *R: I think information should be reaching people faster before the time they go for HIV testing and VMC.*
33. I: Now I would like us to discuss about sexual reproductive health services and Pills for HIV prevention: called pre-exposure prophylaxis (PrEP). Sexual reproductive health includes services that promote good sexual health and reproduction. They include but not limited to family planning, cervical cancer screening sexual transmitted infection management cervical, condom distribution and many more. Today we will only discuss family planning, Sexual transmitted infection management cervical screening and PrEP. We will look at each of these one by one. Let us start with STI services. Explain to me, what you have ever learnt about STIs when you came to Voluntary Medical Male circumcision clinic?
34. *R: We have learned that HIV and AIDS and other diseases like Gonorrhea*
35. I: What did the staff say would happen if you were found or suspected with STIs?
36. *R: You start with HIV testing for verification*
37. I: What happens when you are positive?
38. *R: They give you counselling as well as ma ARVs*
39. I: They don’t proceed with the circumcision, like they have found you with gonorrhea what happens?
40. *R: They did not explain to us what happens after that, but according to me, after testing, they found that am negative, so you proceed with circumcision but I did not see anyone going back home without being circumcised after testing.*
41. I: As an individual, what is your opinion on integrating STI services with Voluntary Medical Male Circumcision services?
42. *R: The good thing is that they should be testing before circumcision to know what to be done next*
43. I: How do you think is the best way to offer STI services at the Voluntary Medical Male Circumcision clinics? Should it be inside or outside?
44. *R: Either way, but it should be a private room.*
45. I: Why private room?
46. *R: So that a person keeps her privacy and it’s not for the public*
47. I: What is it that you do not like integrating STI services in Voluntary Medical Male circumcision services?
48. *R: There is nothing that I don’t like.*
49. I: What do you think are the barriers and concerns on this integration of STI services with Voluntary Medical Male circumcision services?
50. *R: Maybe because of fear, people would be worried to go for testing*
51. I: What do you think should be done to address these concerns and barriers?
52. *R: By telling the people that it is private thing, no one will be told about your status, and you will be the only one who will know.*
53. Let us talk family planning. Explain to me about anything that you know about family planning (vasectomy for men and family planning for female partners)?
54. *R: I heard about Norplant and pills.*
55. I: What happens when a woman wants a nail plant and a male want vasectomy?
56. *R: You go to where you will get vasectomy and nail plant*
57. I: Bwaila clinic or where they offer VMMC or just (Name of Hospital) hospital?
58. *R: (Name of Hospital)*
59. I: As an individual, what is your opinion on integrating Family planning in Voluntary Medical Male circumcision services/clinic?
60. *R: I think, they should not combine, a person should make her own special ay to get the services.*
61. I: Why should they not combine?
62. *R: It will be a lot of work and tiresome for one to go for VMMC and also go for family planning*
63. I: How do you think is the best way to offer family planning services within Voluntary Medical Male circumcision services clinics?
64. *R: I would love it if after circumcision you should go for family planning*
65. I: Now let us talk about cervical cancer screening. Explain to me what you know about cervical cancer screening?
66. *R: What I know is cancer is screened at the hospital to know if the woman has cervical cancer*
67. I: As an individual, what is your opinion on integrating partner cervical cancer screening with Voluntary Medical Male circumcision services
68. *R: I think, they should be screening the woman for cancer and the man should be circumcised*
69. I: What is it that would like about integrating cancer screening with Voluntary Medical Male Circumcision services?
70. *R: It helps one to know how to prevent cancer after knowing that she doesn’t have cancer*
71. I: How do you think is the best way to offer Cervical Cancer screening services within Voluntary Medical Male circumcision clinics? Should be inside or outside?
72. *R: It should be outside.*
73. I: Why outside?
74. *R: When is inside it will disturb circumcision work but it should be outside*
75. I: What do you think could be the barriers or concerns with partner cervical cancer screening and Voluntary Medical Male circumcision integration?
76. *R: There is a concern, since a couple comes from one house to go to the hospital, they would not have someone at home to support them*
77. I: How do you think these concerns and barriers be addressed?
78. *R: By the time people are taken to go get the services, they should not take a lot of time at the hospital so that they should go back and look after their home*
79. I: Now let us discus about PrEP. Have you heard about PrEP?
80. *R: Yes.*
81. I: If you have heard about PrEP, please tell me what you know about PrEP?
82. *R: This is when someone who is negative takes the medicine before having sex with someone who is positive so that he shouldn’t be infected*
83. I: How did you learn about this?
84. *R: They explained to us how it works and gave us.*
85. I: Are the drugs available or they are finished?
86. *R: These medicines are still available there is a process that will make them available*
87. I: Is it necessary to make PrEP available to HIV negative men and women?
88. *R: No I don’t think so.*
89. I: If No; what are your concerns?
90. *R: Those who do not have HIV need to be abstaining from HIV*
91. I: Who is supposed to drink these medicine?
92. *R: The one who is negative when his or her partner is negative*
93. I: So I have asked a question, is it important that PrEP should be available to men and women without HIV
94. *R: No it shouldn’t*
95. I: But it should be available to those whom are positive?
96. *R: No, it should be available to those families that has one partner with HIV*
97. I: If PrEP become available, what is your opinion on integrating PrEP with Voluntary Medical Male circumcision services? How happy would you be to access them?
98. *R: Before circumcision, the person should drink prep first, maybe they circumcised someone who is positive the other person should not get the virus*
99. I: What if PrEP is integrated with VMC
100. *R: I would love it*
101. I: What will make you love it?
102. *R: There wouldn’t space at the hospital for one to get HIV infected*
103. I: How do you think PrEP would be offered in this clinic?
104. *R: After testing*
105. I: If found positive
106. *R: If found positive, then you need to be circumcised, but the one behind him should take prep*
107. I: What about those who are coming to the hospital just to get prep, how should it be handled?
108. *R: They should not be given prep but only to those who have come for circumcision*
109. I: What concerns would you have about people taking PrEP?
110. *R: There is no concern.*
111. I: Let us talk about other services. If you were given powers to choose and integrate services in Voluntary Medical Male Clinics, what are the services that you would think of Integrating?
112. *R: After a person is circumcised he needs to be given money because most of their work is on hold, hence they need the money to buy painkillers, of course they do give us painkillers but we they get done*
113. I: What other help would you need?
114. *R: No I don’t have anything.*
115. I: How do you think these services should be offered in the clinic
116. *R: After being circumcised.*
117. I: Thank you for taking your time to discuss with me today. Your answers will be very helpful in improving the health service delivery at circumcision clinics. Before we close, do you have anything to say?
118. *R: There is nothing really, maybe the government should increase VMMC centers because we have a lot of people in the country*
119. I: Again, thank you so much for taking your time to speak with me.
120. THE END

**D43 STUDY**

**Date of Interview: 05 September 2018**

**Type of Participant: Male Index Participant**

**Interview Number: D-43-0050**

**Interviewer: I. N.**

**Total Interview Time: 21 minutes 07 seconds**

**Interview Summary:** **(from summary sheet)**

| **SERVICE TO BE INTERGRATED** | **THOUGHTS ON INTERGRATION** |
| --- | --- |
| Couple HIV Testing and Counseling | Thinks its good development because if it happens that all of you are positive you can start the treatment together. |
| STI Services | Thinks it’s a good idea because if a person is found with an STI they can still get help at the same hospital. |
| Family Planning | Thinks it is a good idea because sometimes it happens that you are getting circumcised and the woman gets family planning |
| Cervical Cancer Screening | Thinks it’s a good idea because by the time one is getting circumcised it helps the woman not to get cancer. |
| PrEP | Likes the idea of integrating PrEP with VMMC services because it can happen that a friend would happens to sleep with someone who is positive so when a couple are drinking prep it can help. |
| Other Services | Thinks supposed to have a hospital where they have condoms |

**Remarks:** Participant was relaxed, calm but less confident.

**Interview Text:**

1. I: Thank you for taking the time to talk with me today. I would like to ask you some questions today about the way you feel and what you think about some issues related to the service you receive here and how we can include other services in Voluntary Medical Male Circumcision (VMMC) clinics. There are no right or wrong answers to these questions. We would like to hear your opinion and your experiences in your own words. Do you have any questions before we begin?
2. *R: No.*
3. I: Do your ever talk to your care providers about how the services are provided here?
4. *R: Yes, we are able to.*
5. I: Can you give me an example of a time when you managed to talk to your health care provider about the services you received here?
6. *R: When they finished explaining to us about VMC I asked them questions about the procedures and how it helps us*
7. I: After circumcision during checkup what did you discuss with the service providers?
8. *R: they were told me how I can take care of the wound they were saying I should be washing it 3 times a day with salt water, it helped because I had no problems at all*
9. I: Now let us talk about partner HIV testing here at the circumcision clinic. Tell me what happens when a man brings in a spouse at the Voluntary Medical Male circumcision clinic
10. *R: What happens is; it’s a good thing when you go together when the man wants to be circumcised because they tell us a lot of things so when the man forgets the woman can remind him.*
11. I: How is the partner involved in the services that men received here at the clinic?
12. *R: No I haven’t*
13. I: For those men who bring their partners when going for VMMC, what do you think makes them do so?
14. *R: They want to know about their life.*
15. I: What about those who do not bring their partners?
16. *R: They are afraid if they will be found positive what will happen next…*
17. I: In your opinion, what are your thoughts about bringing in your partners for HIV testing at Voluntary Medical Male circumcision clinic
18. *R: I think it’s good, because if it happens that all of you are positive, you can start the treatment together*
19. I: What do you think can be done to make men bring their partners here for HIV testing and counselling?
20. *R: We should provide information and counselling that they can go to the hospital and get tested together with their partners.*
21. I: What do you think are the barriers and concerns on this integration?
22. *R: There is no barrier because each one of them will know their results*
23. I: Now I would like us to discuss about sexual reproductive health services and Pills for HIV prevention: called pre-exposure prophylaxis (PrEP). Sexual reproductive health includes services that promote good sexual health and reproduction. They include but not limited to family planning, cervical cancer screening sexual transmitted infection management cervical, condom distribution and many more. Today we will only discuss family planning, Sexual transmitted infection management cervical screening and PrEP. We will look at each of these one by one. Let us start with STI services. Explain to me, what you have ever learnt about STIs when you came to Voluntary Medical Male circumcision clinic?
24. *R: Sexual transmitted diseases, it’s good that you go to the hospital and get circumcised because it’s not easy to get STIs*
25. I: What did the staff say would happen if you were found or suspected with STIs
26. *R: They said that they are able to give him medicine to help him so that the viral lead should not be high and they are able to circumcise him*
27. I: As an individual, what is your opinion on integrating STI services with Voluntary Medical Male Circumcision services?
28. *R: I think it’s good because if a person is found with an STI they can still get help at the same hospital.*
29. I: As an individual what do you think is the best way to offer these services together?
30. *R: There is need to have different people so that they can help out in providing the services.*
31. I: Should the room be inside or outside in a private place?
32. *R: It should be inside, because if you are found positive you should go get the medicine inside.*
33. I: When should the person be treated?
34. *R: After circumcision*
35. I: What do you think are the barriers and concerns on this integration of STI services with Voluntary Medical Male circumcision services?
36. *R: There are no any barriers.*
37. I: Let us talk about family planning. Explain to me about anything that you know about family planning (vasectomy for men and family planning for female partners.
38. *R: It’s good to be here at VMMC for women so that they can also get help about family planning, when the man gets circumcised the woman can get family planning*
39. I: As an individual, what is your opinion on integrating Family planning in Voluntary Medical Male circumcision services/clinic?
40. *R: I would like it to be an integration*
41. I: What is it that you would like about integrating Family Planning services in Voluntary Medical Male Circumcision?
42. *R: It’s good because sometimes it happens that you are getting circumcised and the woman gets family planning, it’s good that you will go together so that when the man is getting circumcised the woman should be getting family planning services*
43. I: What would make you not want integrating Family Planning services in Voluntary Medical Male Circumcision services?
44. *R: There is nothing.*
45. I: How do you think is the best way to offer family planning services within Voluntary Medical Male circumcision services clinics?
46. *R: By the time the man is getting circumcised the woman should also be getting family planning services.*
47. I: What about if the man wants the family planning services?
48. *R: By the time after getting circumcised the man can go and get family planning services.*
49. I: How many ways of family planning do you know?
50. *R: Nor plant and injection.*
51. I What about male family planning?
52. *R: There is using of condoms and vasectomy*
53. I: Should the services be offered outside or inside?
54. *R: It should be in the same room that the man is getting circumcised*
55. I: What do you think could be the barriers or concerns on family planning and Voluntary Medical Male circumcision integration?
56. *R: There is no barrier*
57. I: Let us talk about cervical cancer screening**.** Explain to me what you know about cervical cancer screening?
58. *R: It’s good to do cancer screening so that before the cancer has manifested you can be able to receive medicine to deal with cancer*
59. I: As an individual, what is your opinion on integrating partner cervical cancer screening with Voluntary Medical Male circumcision services
60. *R: It’s very good because by the time you were getting circumcised it helps the woman not to get cancer, so it can help to know if your wife has cancer or not*
61. I: What is it that would like about integrating cancer screening with Voluntary Medical Male Circumcision services?
62. *R: It can help in the way that, everyone would know if your wife has cancer and the woman would also know that she doesn’t have cancer*
63. I: What is it that would make you not want integrating cervical cancer screening with Voluntary Medical Male Circumcision?
64. *R: There is nothing.*
65. I: How do you think is the best way to offer Cervical Cancer screening services within Voluntary Medical Male circumcision clinics?
66. *R: It should be offered at the same time when the man is getting circumcised or before circumcision sot that they can go for screening to know if the wife is cancer free or not*
67. I: It should be in the same building.
68. *R: Yes.*
69. I: What do you think could be the barriers or concerns with partner cervical cancer screening and Voluntary Medical Male circumcision integration?
70. *R: There is no barrier at all.*
71. I**:** Now let us discus about PrEP. Have you heard about PrEP?
72. *R: No I haven’t.*
73. I: If you have not heard about PrEP, I will explain how the medicine works. PrEP is anti-HIV medicine that keeps HIV negative people from being infected. There is a single pill that is taken once daily, and if you take it regularly, it is highly effective at prevention people from being infected. Now, how do you feel about PrEP?
74. *R: I have understood it; it can be a good thing when it comes to HIV to be drinking prep*
75. I: If someone asks you about prep what can you tell him?
76. *R: I can tell them that prep is a medicine that you drink before sleeping with a woman who you know that she is HIV positive, so that you would not get HIV infection*
77. I: Is it necessary to make PrEP available to HIV negative men and women?
78. *R: Yes, because it can happen that your friend would not go in the right path and may have sex with someone who is positive so when you and your wife are taking prep, it can help that you wouldn’t infect the other*
79. I: If PrEP become available, what is your opinion on integrating PrEP with Voluntary Medical Male circumcision services? How happy would you be to access them?
80. *R: It’s good because people at the time, it can happen that when the man gets circumcised, the woman would be promiscuous, so the man can be given prep so that he should not get infected.*
81. I: Would you want to receive PrEP?
82. *R: Yes.*
83. I: How do you think PrEP would be offered in this clinic?
84. *R: After someone has been circumcised he should be given PrEP.*
85. I: Before going home?
86. *R: Yes*
87. I: What concerns would you have about people taking PrEP?
88. *R: There is no concern.*
89. I: Or I should say what your concerns are when people are receiving PrEP?
90. *R: There is no concern because it helps to prevent HIV infection*
91. I: Let us talk about other services. If you were given powers to choose and integrate services in Voluntary Medical Male Clinics, what are the services that you would think of Integrating?
92. *R: What we have discussed?*
93. I: Yes, including others you know….
94. *R: There is supposed to have a hospital where they have condoms*
95. I: That’s family planning?
96. *R: Yes, plus those medicine that prevents one from getting infected with HIV*
97. I: there is nothing else?
98. *R: No.*
99. I: Explain to me what the reasons are for your choice.
100. *R: I have chosen prep because firstly you would not get HIV infection and women will be helped not to get unnecessary pregnancy*
101. I: How do you think these services should be offered in the clinic?
102. *R: The time when the man is getting circumcised the woman can go get family planning services as well as prep or after circumcision, they can go together and get PrEP*
103. I: Thank you for taking your time to discuss with me today. Your answers will be very helpful in improving the health service delivery at circumcision clinics. Before we close, do you have anything to say?
104. *R: Yes, male circumcision for those from the village, most of them don’t know about it, so there is need for them to get access to be information, maybe through newspapers so that they should know the importance of VMC.*
105. I: Again, thank you so much for taking your time to speak with me.
106. THE END.

**D 54 STUDY**

**Date of Interview: 10 September 2018**

**Type of Participant: Male index participant**

**Interview Number: D-43-0054**

**Interviewer: I.N.**

**Total Interview Time: 33 minutes 08 seconds**

**Interview Summary:** **(from summary sheet)**

| **SERVICE TO BE INTERGRATED** | **THOUGHTS ON INTERGRATION** |
| --- | --- |
| Couple HIV Testing and Counseling | Thinks it’s a good development because it will help those service providers to know if one is positive so it can help them to be careful when dealing with contaminate blood, it can also help a client to know if they are positive so I should avoid getting re-infected |
| STI Services | Thinks it’s a good idea because for a lot of people fail to just go to the hospital to get tested when you go for VMC it helps when you were avoiding to still know your status |
| Family Planning | Thinks it is a good idea because it is good to combine because it’s like killing two birds with one stone, one you get circumcised and two you get family planning services |
| Cervical Cancer Screening | Thinks it’s a good idea because it will help to know if the woman has cancer |
| PrEP | Like the idea of integrating PrEP with VMMC services because it should be available because it will reduce the spread of the disease |
| Other Services | Thinks they should add an injection so that you can stay a week without the pain after circumcision |

Remarks:

Participant was open and confident. He was able to explain everything and clearly.

1. I: Thank you for taking the time to talk with me today. I would like to ask you some questions today about the way you feel and what you think about some issues related to the service you receive here and how we can include other services in Voluntary Medical Male Circumcision (VMMC) clinics. There are no right or wrong answers to these questions. We would like to hear your opinion and your experiences in your own words. Do you have any questions before we begin?
2. R*: No*
3. I: Do your ever talk to your care providers about how the services are provided here?
4. *R: Yes*
5. I: Can you give me an example of a time when you managed to talk to your health care provider about the services you received here?
6. *R: Firstly, they tell us how circumcision is done, it’s benefits and we were given time to ask questions before circumcision to know exactly what will happen and other things so that you can make your own decision.*
7. I: Now let us talk about partner HIV testing here at the circumcision clinic. Tell me what happens when a man brings in a spouse at the Voluntary Medical Male circumcision clinic?
8. *R: A man is already circumcised or not?*
9. I: It can either be both but we are talking of a man bringing his partner for testing
10. *R: It can help in a way, if the couple was not trusting each other; whether one of them has it or not, so if they can resolve that conflict, both of them would agree to be faithful to each other but if one of them is[HIV] positive it helps to prevent the other one from getting it.*
11. I: How is the partner involved in the services that men received here at the clinic? Have you seen anyone come with their partner for testing?
12. *R: Let’s say escorting each other when they are bringing their child*
13. I: What do you think are the motivators that make the men bring their spouses here for testing?
14. *R: Maybe they want to tell their wives that they are faithful “even if you don’t believe me”, so when you take your partner for testing it’s like telling her that “you should not doubt me.”*
15. I: What do you think demotivates men to bring their partners here for HIV counselling and testing?
16. *R: It depends on how you live your life when you are married; it can happen that you are having multiple sex partners; so to take the wife for testing is not easy so you want to hide if you have the virus from your wife*
17. I: What do you think can be done to make men bring their partners here for couple testing and counselling?
18. *R: The main thing that is needed is civic education, telling people the advantages of going for testing together as a couple because marriage is between two people but take the kids as well for testing so that the children they can grow well and teach the children the importance of HIV testing as a family.*
19. I: You as Individual, what is your opinion on integrating couple HIV counseling with Voluntary Medical Male circumcision services?
20. *R: I think it’s good to get tested before circumcision because it will help those service providers to know if one is [HIV] positive. So it can help them to be careful when dealing with contaminated blood, it can also help you to know that you are [HIV] positive so I should avoid getting re-infection.*
21. I: So you agree with the integration?
22. *R: Yes.*
23. I: What do you think are the barriers and concerns on this integration
24. *R: Integration can be there meaning that men will have fear to come to the clinic for VMMC if they know they will get tested. So, maybe it can be a reason for men not to come for VMMC because of testing. So there is need to sensitize these men that being [HIV] positive is not the end of the world. When these men are sensitized they should be coming with confidence since they have undergone counselling on the importance of HIV testing and VMC.*
25. I: Now I would like us to discuss about sexual reproductive health services and Pills for HIV prevention: called pre-exposure prophylaxis (PrEP). Sexual reproductive health includes services that promote good sexual health and reproduction. They include but not limited to family planning, cervical cancer screening sexual transmitted infection management cervical, condom distribution and many more. Today we will only discuss family planning, Sexual transmitted infection management cervical screening and PrEP. We will look at each of these one by one. Let us start with STI services. Explain to me, what you have ever learnt about STIs when you came to Voluntary Medical Male circumcision clinic?
26. *R: Like AIDS… syphilis… so, like the service providers explained that if you are found with STIs that means as an individual, you are at risk; not only you but there is a risk in your family and those that surround you. Like at work, you may not be strong and healthy enough to work, so that’s what can happen to you if you have the STI.*
27. I: What did the staff say would happen if you were found or suspected with STIs?
28. *R: If you have STIs they said they can still circumcise you but it depends on the condition. For example, if you are found with syphilis and gonorrhea, that person can’t be circumcised because he might still be at risk so he is sent home to get better and come back to be circumcised*
29. I: As an individual, what is your opinion on integrating STI services with Voluntary Medical Male Circumcision services?
30. *R: I personally think it is a good idea to integrate because for a lot of people to just go to the hospital to get tested because we are sick, we fail. So when you go for VMMC, it is helpful if you were avoiding it; you still know your status anyways so that helps those who avoids testing.*
31. I: How do you think is the best way to offer STI services at the Voluntary Medical Male Circumcision clinics?
32. *R: It should be given when one is not circumcised yet. he has known what is happening in his body, he has to know his status before any procedures so that you get counselled*
33. I: What is it that you do not like integrating STI services in Voluntary Medical Male circumcision services?
34. *R: There is nothing, we need to combine*
35. I: What do you think are the barriers and concerns on this integration of STI services with Voluntary Medical Male circumcision services?
36. *R: I don’t think there is any barrier, because combining all these there won’t be any barriers, maybe at work but still it happens that you told them you are taking the day off?*
37. I: Now let us talk about Family planning. Explain to me about anything that you know about family planning.
38. *R: Family planning like vasectomy; it depends on the kids they have and the care they give to the children, so when we talk about family planning, it is good because it has a lot of benefits.*
39. I: Tell us some of the benefits…
40. *R: If someone is on family planning and you have 3 kids, you are able to send the kids to school. Give them what they want and people would see that these people are happy than those that are just giving birth and your home looks good.*
41. I how many family planning methods do you know
42. *R: In- plant… I don’t know others well, let’s say pills*
43. I: What about men?
44. *R: Pills so that they shouldn’t erect, condoms, vasectomy*
45. I: As an individual, what is your opinion on integrating Family planning in Voluntary Medical Male circumcision services/clinic?
46. *R: It is good to combine because it is like killing two birds with one stone; firstly, you get circumcised and secondly, you get family planning services, if you have children, you can get the service and be free to do whatever you want because you are satisfied with the number of the children you have.*
47. I**:** What would make you not want integrating Family Planning services in Voluntary Medical Male Circumcision Services?
48. *R: Maybe because of being shy, like the men would be shy to go ask about family planning and tell them about the number of kids they have*
49. I: How can we deal with it?
50. *R: The big thing is to it down with the person despite having a lot of children and tell him the benefits of family planning so that he shouldn’t add more*
51. I: How do you think is the best way to offer family planning services within Voluntary Medical Male circumcision services clinics?
52. *R: It should be given before circumcision, during counselling we should be told about family planning*
53. I: What do you think could be the barriers or concerns on family planning and Voluntary Medical Male circumcision integration?
54. *R: There are no concerns because it’s good, maybe the only concern can be that that already have a lot of children everywhere and they feel like they made a mistake but it’s not a big of a concern*
55. I: How do you think these concerns and barriers be addressed?
56. *R: Just teaching them, going in villages with a speaker and talk about family planning so that they should know the services they will be getting at the hospital*
57. I: Now let us talk about cervical cancer screening. Explain to me what you know about cervical cancer screening
58. *R: Cervical cancer is not a good disease that makes women not to give birth, I don’t know if there are medicine for it.*
59. I: As an individual, what is your opinion on integrating partner cervical cancer screening with Voluntary Medical Male circumcision services
60. *R: If they can combine the two, since it involves screening of women, it will help to know if the woman has cancer, when you know that the woman has it or not, you can receive counsel to avoid cancer, maybe the man is a carrier it can help the woman not to be infected.*
61. I: You like it
62. *R: Yes*
63. I: What is it that would make you not want integrating cervical cancer screening with Voluntary Medical Male Circumcision?
64. *R: Maybe when testing the woman, if the person doing screening is a man, I wouldn’t like it, but if it’s a woman then it’s okay but if it’s a man it’s better to go back home*
65. I: How do you think is the best way to offer Cervical Cancer screening services within Voluntary Medical Male circumcision clinics?
66. *R: Maybe leaving woman nurses to deal with women so that they can be able to get comfortable and not get serviced by men*
67. I: What do you think could be the barriers or concerns with partner cervical cancer screening and Voluntary Medical Male circumcision integration?
68. *R: Maybe if the woman doesn’t want to come with at the hospital I think that the biggest problem that means he won’t know her status*
69. I: How do you think these concerns and barriers be addressed?
70. *R: By the time the man is coming for circumcision, I he has a wife, it’s better to tell the woman the goodness to cancer screening so that they can come together for counselling so that when the woman hears this he will have the desire to come for screening*
71. I: Now let us discus about PrEP. Have you heard about PrEP?
72. *R: No I don’t think so, is it the one where one takes when he has slept with some with HIV positive for 30 days?*
73. I: No, that is PEP. Let me tell you about prep. PrEP is anti-HIV medicine that keeps HIV-negative people from being infected. There is a single pill that is taken once daily, and if you take it regularly, it is highly effective at prevention people from being infected. Now, how do you feel about PrEP?
74. *R: It is alright. These are pills that you take not to get infected; that means this prep is needed for people who can’t abstain from sex.*
75. I: Is it necessary to make PrEP available to HIV negative men and women?
76. R: *Yes, it should be available maybe to prostitutes, truck drivers because drinking it dairy is tiresome*
77. I: It should be available to men and women who doesn’t have HIV?
78. *R: It should be available because it will reduce the spread of the disease, let me say I take it and sleep with someone with HIV I am safe and I won’t get infected*
79. I: Would you be happy?
80. *R: Yes, I would be.*
81. I: If PrEP become available, what is your opinion on integrating PrEP with Voluntary Medical Male circumcision services? How happy would you be to access them?
82. *R: I would like it.*
83. I: How do you think PrEP would be offered in this clinic?
84. *R: In a VMC clinic it should be given when one has completed his dosage that relives the pain of circumcision.*
85. I: What concerns would you have about people taking PrEP?
86. R: *People will be having sex with no protection*
87. I: What should be done to address these concerns?
88. *R: People would be counseled on how* PrEP *works and* PrEP *should be given to those families where one is positive and the other one don’t have*
89. I: Let us talk about other services. If you were given powers to choose and integrate services in Voluntary Medical Male Clinics, what are the services that you would think of Integrating?
90. *R: Maybe the medicine after circumcision they don’t go together, Panadol and the pain that you feel it doesn’t help, maybe if they can add an injection so that you can stay a week without the pain, and Panadol should not be given, but other pills to replace Panadol for the pain*
91. I: Explain to me what the reasons are for your choice.
92. *R: The reason is to increase strength to the injection they give us, it’s a pain that is excruciating so it doesn’t match the drug with the medicine, o if they can increase its power people will not be afraid of the people because people believe that there is excruciating pain after circumcised so when that is done, people will be coming*
93. I: How do you think these services should be offered in the clinic
94. *R: It should be given after being circumcised*
95. I: Thank you for taking your time to discuss with me today. Your answers will be very helpful in improving the health service delivery at circumcision clinics. Before we close, do you have anything to say?
96. *R: The big thing is that, the medicine we get after being circumcised doesn’t match with the pain, because it brings fear to the one who wanted to come to get circumcised when they hear about the pain that their friend Is experiencing*
97. I: Again, thank you so much for taking your time to speak with me.

THE END.

**D43 STUDY**

**Date of Interview: 10 September 2018**

**Type of Participant: Male Index Participant**

**Interview Number: D-43-0055**

**Interviewer: I. N.**

**Total Interview Time: 21 minutes 35 seconds**

**Interview Summary:** **(from summary sheet)**

| **SERVICE TO BE INTERGRATED** | **THOUGHTS ON INTERGRATION** |
| --- | --- |
| Couple HIV Testing and Counselling | Thinks that it will make a couple to have faith in the medical services and health life but also it will enhance trust on each other. |
| STI Services | It is very good because it could assist in saving time since all will be found in one place on a clinic |
| Family Planning | It is very good since it will help reduce the number of children per household and infections which would help government to provide efficient development initiatives to the communities. In addition, it will help in improving availability of land and lessens the pressure on land. |
| Cervical Cancer Screening | Warmly welcome the idea because cancer is very dangerous so it very important that we be aware on how you can get it and how you can transmit to others |
| PrEP | It is necessary because it will help to reduce cases of HIV among people and also people will be protected from contracting the virus since it reduces the chances of reducing the chances. |
| Other Services | Blood pressure and TB screening services |

**Remarks:**

The participant was confident and open enough.

**Interview Texts:**

1. I: Thank you for taking your time to talk to me today, I would like to ask you some questions on how you feel and opinion on the services you receive here and how we can include other services in the voluntary male circumcision clinics. There are no right or wrong answers to these questions. We would like to hear your experiences in your own words. Do you have any questions before we begin?
2. *R: No. I don’t have any question.*
3. I: Are you able to discuss with your health service providers on how the services are offered here?
4. *R: Yes.*
5. I: Can you explain to me an example where you ever talked to your service providers about the service you received here?
6. *R: I talked to them through a question that “if I found that my blood pressure is high during circumcision, can they proceed with the circumcision?” so they said “no” and they explained to me that “if the blood pressure is high… but if am okay they can do the circumcision.”*
7. I: Mh… Now let us talk about HIV couple testing. Tell me what happens when a man brings a wife here at the voluntary male medical circumcision clinics? Or I should ask that have you ever seen a man coming along with their spouse at the circumcision clinic?
8. *R: Yes, I have ever seen.*
9. I: Okay. What happens?
10. *R: It happens that both partners would like to know their status and this helps the couple not to have doubts on each other if it happens that one gets infected so it promotes unity.*
11. I: okay. What role does the spouse play on the service offered during circumcision of their partner?
12. *R: they are advised to take of the husband and be taking care of each other so that the love between them should continue.*
13. I: Okay. If you brought your spouse what prompted you to do so? Did you bring your spouse to the circumcision?
14. *R: No I came alone.*
15. I: Okay. But like for other men that bring their spouse to the circumcision clinic, what do you think prompts them to do so?
16. *R: I think they just do that out of love so that their marriage should be a good one.*
17. I: Like you did not bring your loved one, what made you to do so?
18. *R: For me my partner stays very far from here and I also stay away but still she encouraged me to come and do circumcision because it helps.*
19. I: So what makes men not to bring their spouse to the clinic during circumcision?
20. *R: Maybe it’s just out of shyness but to my side I think what is needed is to just encourage each other that they should be coming together.*
21. I: Okay. In your opinion, what do you think about bringing your partner at the circumcision clinic to do an HIV test as a couple?
22. *R: My opinion is that if we make a decision to do that we should also encourage each other not be involved in immoral sexual behaviors so that we remain safe from the infection.*
23. I: Okay. But can you like the idea?
24. *R: Yes, I will like the idea.*
25. I: Okay. Aaah what is your opinion about the integration of couple HIV testing within the male medical circumcision clinics?
26. *R: As for me I think it will make me and my wife to have faith in the medical services and also have health life but also it will make us to have trust on each other.*
27. I: So, how do you receive the idea that people should be able to test for HIV as a couple at the circumcision clinics?
28. *R: I receive that news with keen interest because circumcision will also help in the blood status. If one is diagnosed of HIV s/he will be provided with the right counselling.*
29. I: What concerns or barriers do you think can be there between this integration?
30. *R: Maybe misunderstandings because when others test positive they become depressed but they receive counselling that you and your wife should be encouraging each other and that testing positive is not the end of everything but it’s the beginning of another new life so this integration is very good.*
31. I: Now I would like us to discuss about sexual reproductive health services, pills for HIV prevention called pre-Exposure Prophylaxis. sexual reproductive health include services that promote good sexual health and reproduction they include but not limited to family planning, cervical cancer screening, Sexually Transmitted Infections management and condom distribution and many more. Today we will only discuss about family planning, cervical cancer screening, Sexually Transmitted Infections management and PrEP. We will look into each one of these one by one, so let’s start with STI services. Explain to me what you have learnt about sexually transmitted infections since you came to the male medical circumcision clinic. What did health service say if you would be suspected or diagnosed of STIs at circumcision?
32. *R: They said that maybe they can assist us immediately but if we can tell them that we were diagnosed of STIs otherwise the services are always there.*
33. I: In your own opinion, what do you think about integrating STI services within the VMMC clinics?
34. *R: I think all of these are very good because they assist in saving time for us since they will all be found in one place on a clinic so yeah it can be of great help.*
35. I: This service of screening and management of STIs, how can it be provided in the circumcision clinics?
36. *R: After they asked you if you have STIs or have been diagnosed and then they give you drugs and tell you to come again later after recovering from the infection.*
37. I: okay. What do you think are the concerns and barriers to integration?
38. *R: It is possible that some people are shy that should I let the doctor see my condition, maybe they will insult me because of this and all that. But all in all medical practitioners they provide the assistance.*
39. I: Okay. So what can we do to resolve that concern?
40. *R: Maybe the health service providers should just be providing information to their clients how these things work maybe through youth clubs and all that.*
41. I: Now let us talk about family planning, can you explain to me anything you know about family planning. Be it vasectomy or female methods of it.
42. *R: Let us talk about women, family planning is very good because it helps to reduce the number of children in a household which becomes a problem in terms of school fees if you have a lot of children so family planning helps to reduce the number of children per family and also allows healthy living and enough food availability per household.*
43. I: What family planning methods do you know for women or men?
44. *R: Nor-plant for women and vasectomy for men and condoms.*
45. I: For women some they say loop or depo. So what is your opinion about integrating family planning services within the circumcision clinics?
46. *R: Family planning is very good since it helps to reduce the number of children per household and also helps to reduce infections and this helps government to provide efficient development initiatives to the communities and also it helps in enough availability of land and lessens the pressure on land.*
47. I: What can make you not to want the integration of family planning within the circumcision clinics?
48. *R: Some say it brings some side effects so maybe sometimes I may have stress that it would bring some problems.*
49. I: Problems like what?
50. *R: some that can make the person become infertile forever and am not sure how true that is.*
51. I: Mm… it depends on the method you have chosen. Some may choose permanent closure of the reproductive passages and this will make them never to bear children anymore. So for this concern, how do you think we can deal with it?
52. *R: Maybe just explaining to people how it goes because if they become aware, i am sure if would be of help to them.*
53. I: How can family planning be provided at the circumcision clinics?
54. *R: Maybe after the circumcision so that they can be taught of all the family planning methods.*
55. I: What place should it be provided?
56. *R: Maybe after circumcision they should be going to another room within the same building.*
57. I: Ah… let us look at cervical cancer screening. What do you about cervical cancer screening?
58. *R: Okay. Cervical cancer is very difficult so a man’s penis is a carrier of cancer so if you do circumcision you prevent the ability of transmitting the infection to your wife*
59. I: What is your opinion about integrating cervical cancer screening within the VMMC clinics?
60. *R: I think they should just have another room for cervical cancer screening so that you should be moving from the circumcision clinic to the cervical cancer screening room within the same building so that is saves time for clients.*
61. I: But how are you receiving the news about this new idea of integrating these two services?
62. *R: I will warmly welcome it because cancer is very dangerous so it very important that we be aware on how you can get it and how you can transmit to others.*
63. I: Ah… what barriers or concerns are there to this integration?
64. *R: Maybe to others can be stressed but all these are very important since cancer is very dangerous but if you detect it farmers you are able to get the help and be cured.*
65. I: So what concern can there be?
66. *R: Stress is that maybe they will do an operation on me or they will cut out something on me and all that.*
67. I: So what do you think can be done to do away with this concern?
68. *R: Maybe health service providers should be explaining properly we can learn something on how we can go about it.*
69. I: Now let us talk about PrEP. Have you ever heard about PrEP?
70. *R: No. this is my first time.*
71. I: Alright. If you have never heard about PrEP I will explain it to you how it works. PrEP is a drug that helps in HIV to prevent HIV negative from contracting the virus. There is one pill that is taken once every day and if taken properly it is effective in preventing the virus. Now how do you understand about PrEP?
72. *R: Now I feel good because I am aware about how it works.*
73. I: Is it important to make PrEP available to negative men and women?
74. *R: Yes, it is necessary.*
75. I: Why do you think it is necessary?
76. *R: Because it will help to reduce the cases of HIV among people and also people will be protected from contracting the virus since it reduces the chances of reducing the chances.*
77. I: So if PrEP is to be made available, what is your opinion on integrating it within the VMMC clinics?
78. *R: Because it is all about medical stuff, so maybe they should just have branches like in the healthy centers where it can be provided to people so that HIV cases are reduced because AIDS is very dangerous and HIV which causes is can be reduced.*
79. I: So you are happy with this integration?
80. *R: Yes, am happy with that?*
81. I: So how can you like that this PrEP should be provided in the VMMC clinics?
82. *R: To me, PrEP can help when I need and also I can encourage my friends to get assisted with it like in the circumcision clinics they can be able to explain about this PrEP and people will be assisted.*
83. I: Ah… let’s see………. At the circumcision, when should PrEP be provided?
84. *R: Ah… after circumcision, let’s say I have done circumcision today on 3 and then I be told to come again next week for check-up and then on 15 another check-up maybe at this date PrEP can then be provided to assist me wherever I can go.*
85. I: What concerns do you have about making PrEP available?
86. *R: You know some people may have beliefs and people become stressed that if they take these drugs they may never be able to bear children again.*
87. I: So that concern how can we deal with it?
88. *R: Maybe health service providers should explain to us how it works am sure it can help.*
89. I: Let us discuss the last part which is about other services. If you are given the power to integrate other services to the VMMC services, what are the services you would think of integrating even apart from what we have discussed?
90. *R: Maybe if they would also include blood pressure and TB screening services it would as well be good.*
91. I: Explain to mw the reasons for your choices
92. *R: Because when a person comes to the clinic, they ask you if you have high blood pressure and I said “no yet,” I had no idea about it. So if they offer such services, people will be able to know if they have high blood pressure or not.*
93. I: Ah… how do you think such services can be provided at the circumcision clinics?
94. *R: Maybe before circumcision within the clinic, they can have a separate room where they can be doing blood pressure checks so that one becomes aware before circumcision.*
95. I: Thank you so much for taking your time to discuss these things today, your answers will be helpful in improving the health service delivery at circumcision clinics. Before we close, do you have anything to say?
96. *R: Yes, I have something so say on the issue of circumcision, I did circumcision but the very first week of circumcision the pain was just too much so maybe if they can be providing other strong painkillers apart from just Panadol it will be a better thing.*
97. I: Okay fine. Aaahh thank you for taking your time to talk to me.
98. *R: Thank you.*
99. THE END.

**D 43 STUDY**

**Date of Interview: 12 September 2018**

**Type of Participant: Male Index Participant**

**Interview Number: D-43-0056**

**Interviewer: I.N.**

**Total Interview Time: 36 minutes 31 seconds**

**Interview Summary:** **(from summary sheet)**

| **SERVICE TO BE INTERGRATED** | **THOUGHTS ON INTERGRATION** |
| --- | --- |
| Couple HIV Testing and Counseling | Could help couples know their HIV status together. |
| STI Services | Would help couples to get treatment together. |
| Family Planning | Youths will have a chance to get family planning in a private space |
| Cervical Cancer Screening | Important to integrate so that women can know if they have the cancer or not |
| PrEP | Important to integrated. Only concerned about taking the pills daily |
| Other Services | Thinks cervical cancer screening , family planning and PrEP are the best to be integrated into VMMC |

**Remarks:**

**Participant was relaxed, quick to respond to questions and straight to the point when responding.**

**Interview Text:**

1. I: Thank you for taking the time to talk with me today. I would like to ask you some questions today about the way you feel and what you think about some issues related to the service you receive here and how we can include other services in Voluntary Medical Male Circumcision clinics. There are no right or wrong answers to these questions. We would like to hear your opinion and your experiences in your own words. Do you have any questions before we begin?
2. *R: No I don’t have any questions.*
3. I: Sure. So the first question is: Do you ever talk to your care providers about how the services are provided here?
4. *R: Yes, we do talk to them about the challenges we can meet when we have done VMMC and how we can minimize them.*
5. I: Can you give me an example of what you talked when you came?
6. *R: I asked them about wound healing because we people are different. So we asked them what we can do if the wound doesn’t heal. So they gave us their phone numbers which we can call if the wound doesn’t heal quickly.*
7. I: Ok.
8. *R: Sure.*
9. I: Alright, so I want us to talk about partner HIV testing here at the circumcision clinic. What happens when a man brings a partner at the Voluntary Medical Male circumcision clinic?
10. *R: When a man brings his partner to the clinic he gets the chance to get couple testing with his partner. We do that because sometimes it’s hard for us to come back another day for testing because the partner may not accept it even if we are acting in their best interest. So when we tell them to come with us they feel proud and happy.*
11. I: Ok.
12. *R: Yes.*
13. I: Or did you see anyone who brought a partner at the circumcision clinic?
14. *R: Yes, I have ever seen them but mostly it’s those that are married that bring their partners. For unmarried men like me it’s hard, of course we may want to bring them but sometimes it becomes hard to meet with them.*
15. I: Did you bring your partner to the VMMC clinic?
16. *R: No.*
17. I: Ok. So why do you think is the reason that makes men to bring their partners at the Voluntary Medical Male circumcision clinic?
18. *R: I think what makes them to bring their partners is the issue of VMMC because they discuss about the benefits of VMMC and agree to come together. So when the man is circumcised they need to help each other as a couple.*
19. I: Ok. What do you think makes men not to bring their partners?
20. *R: It’s because the men come to the clinic without talking to their partners about it so they can’t be comfortable to bring them to the VMMC clinic. They prefer to come with their friends instead of their partners. However, it’s important that they bring their partners because a partner is someone you get used to not your friend and you can both have firsthand information about what’s happening with the man.*
21. I: Ok.
22. *R: Yes.*
23. I: Ok, you as Individual, what is your opinion on integrating couple HIV counseling with Voluntary Medical Male circumcision services?
24. *R: I think it’s a good idea because the couple can have a chance of getting tested together and test for cancer as well. So it’s good for them to be tested together and get VMMC as well.*
25. I: Ok. So what do you think are barriers and concerns on this integration?
26. *R: There cannot be any barriers on this integration. The only thing I see is goodness on the part of the clients because they will get tested together.*
27. I: Now I would like us to discuss about sexual reproductive health services and Pills for HIV prevention: called pre-exposure prophylaxis. (PrEP) Sexual reproductive health include services that promote good sexual health and reproduction. They include but not limited to family planning, cervical cancer screening sexual transmitted infection management cervical, condom distribution and many more. Today we will only discuss family planning, Sexual transmitted infection management cervical screening and PrEP. We will look at each of these one by one. Let us start with: screening and sexual transmitted infections. Explain to me, what you have ever learnt about STIs when you came to Voluntary Medical Male circumcision clinic? Explain to me, what you have ever learnt about STIs when you came to Voluntary Medical Male circumcision clinic?
28. *R: I have learnt about STIs when I came here because they said before you are circumcised you can easily contract Gonorrhea, Syphilis and other STIs because when you have sex your foreskin can easily bruise because it’s too soft. When you have done VMMC your penis becomes hard which makes it hard for it to get bruised.*
29. I: What did they tell you would happen in case they suspect or actually test you positive for STIs?
30. *R: They told me that I would still get VMMC because VMMC does not only reduce the risk of HIV but also other STIs so they would still do the surgery so I could be protected from the other STIs.*
31. I: So what is your opinion on integrating STI services with Voluntary Medical Male Circumcision services, so that both of you should be coming here so that the process can take place while you are together with your partner?
32. *R:I feel that it can be good because the men can be tested for STIs before VMMC and that way the benefits of VMMC can be maximized and the client can kill two birds with one stone because he will get VMMC and screening for STIs*
33. I: How do you think is the best way to offer STI services at the Voluntary Medical Male Circumcision clinics?
34. *R: I feel that at the time you come for VMMC and they suspect that you have an STI, that’s the right time for you to get the service. It should be in the same room so that people won’t feel ashamed to switch rooms just to get the service*
35. I: What is it that you do not like integrating STI services in Voluntary Medical Male circumcision services?
36. *R: I wouldn’t say there is anything that I wouldn’t like about the integration. All that is needed is for one to believe in himself so he can be tested for the STIs and not feel good about yourself when coming to the VMMC clinic.*
37. I: What do you think are the barriers and concerns on this integration of STI services with Voluntary Medical Male circumcision services?
38. *R: The barriers can be there depending on how a person thinks about the service. Someone might say “ If I go there they will diagnose me with an STI” SO some people will be afraid to come to the VMMC clinic for fear that they will be tested for STIs*
39. I: What can we do to eliminate such barriers?
40. *R: They can be eliminated by giving the people knowledge so that they don’t feel afraid anymore. They should be motivated just like they are motivated bout VMMC which makes them to want to come to the clinic.*
41. I: Alright. So, let us now talk about family planning; Explain to me about anything that you know about family.
42. *R: Well, I don’t know much about family planning because I have never met a woman who does family planning. But I just hear that family planning is good when a woman comes to the clinic with her partner.*
43. I: What does family planning help?
44. *R: It helps to reduce the number of children that people can have because some people have too many children whom they cannot be able to take care of.*
45. I: what family planning methods do you know of?
46. *I: I cannot be able to list them because I have never done that.*
47. I: So there are condoms, vasectomy for men and there are also several methods for women such as DEPO, Loop, Norplant, condoms, pills. So individually.
48. *R: Oh okay.*
49. I: Alright. So, what is it that you would like about integrating Family Planning services in Voluntary Medical Male Circumcision?
50. *R: It can be a great idea because when one has done VMMC, it does not mean that he is free from contracting STIs, no. You still can get STIs because VMMC reduces one’s risk of HIV by 60% and if one is not using condoms he can still get STIs and HIV. So some people decline family planning because they think that once they have done VMMC then they are protected from everything.*
51. I: What would make you not want integrating Family Planning services in Voluntary Medical Male Circumcision services?
52. *R: As I said, most people think that once they have done VMMC then they are protected from everything. But VMMC reduces the risk by 60% and the remaining 40% means you can get STIs. So it will be better for one to get family planning.*
53. I: Ok. How do you think is the best way to offer family planning services within Voluntary Medical Male circumcision services clinics?
54. *R: I think there should be a special room for family planning.*
55. I: Should the room be inside the clinic or outside?
56. *R: It should be inside the clinic.*
57. I: Why is that?
58. *R: If the room is outside the clinic then it will be a special family planning clinic which can make people to feel shy to access such services since they are on the open. So making the room to be inside will help people to go for family planning without shame because others won’t know that they have gotten such services.*
59. I: Alright. What do you think could be the barriers or concerns on family planning and Voluntary Medical Male circumcision integration?
60. *R: I don’t think there can be any barriers.*
61. I: Ok. Let’s now talk about cervical cancer. Have you ever heard about cervical cancer screening?
62. *R: I don’t know anything about cervical cancer screening, so there is nothing I can say about that.*
63. I: Do you know anything about cervical cancer or what causes it?
64. *R: Yes, I know that it’s caused by the dirt that lies under the foreskin of men’s penises especially due to lack of cleanliness.*
65. I: So women are screened for this type of cancer in other clinics. Do they screen at the VMMC clinic?
66. *R: No they don’t. They just provide VMMC service*
67. I: As an individual, what is your opinion on integrating partner cervical cancer screening with Voluntary Medical Male circumcision services? Say, you come with your partner and she is screened for cancer?
68. *R: It can really help because at the time I come for VMMC I can know if I infected my wife with this disease before I did VMMC. So I can be happy to know whether my wife has the disease or not.*
69. I: What is it that would make you not want integrating cervical cancer screening with Voluntary Medical Male Circumcision?
70. *R: What I cannot like is what I have said that for some of us it’s not easy to bring our partners to places like this one. So some women cannot view this as acting in their best interest. They may feel like they can’t have this disease.*
71. I: Ok, so how do you think is the best way to offer Cervical Cancer screening services within Voluntary Medical Male circumcision clinics?
72. *R: There should be a room where the screening can be done so that when a man presents to the clinic with his wife for screening they can be shown where they can access the service.*
73. I: So let’s imagine you have come for VMMC with your partner, when in the VMMC clinic flow can this service be offered?
74. *R: When a man comes wanting VMMC, he should not be told about the cervical screening on the same day. He can be told on the day that he comes for review so his wife can be screened for cervical cancer.*
75. I: Ok. What do you think could be the barriers or concerns with partner cervical cancer screening and Voluntary Medical Male circumcision integration?
76. *R: There can be concerns because when it comes to VMMC people become reserved. They may choose not to come to the clinic depending on the experience which their friends have had. For cervical cancer screening it’s the same thing. Some women may not want to come for fear that they may be found to have the cancer. So in short what lacks in people is self-confidence. They fear that they may be found with a disease not knowing that they can receive treatment.*
77. I: How can we deal with such barriers?
78. *R: It’s easy to deal with such concerns. The issue here is that there should be rooms where cervical cancer screening can conducted another for family planning and so on. That is easy because people usually wait for something to begin so they can adopt it in the end. So you as medical people need to just start and the people will take it from there.*
79. I: Mm… okay.
80. *R: Sure.*
81. I: NOW Let us discus about PrEP. Have you heard about PrEP?
82. *R: No I have never heard about PrEP.*
83. I: I will explain it to you. PrEP are medicines that help to prevent HIV, they help in sense that a person who is HIV negative should not contract HIV. So they are medicines that when you take, you do not contract HIV. The dose is, a person swallows one pill per day. One pill per day, daily. And if you are following the instructions, this pill works very effectively in helping people prevent from getting HIV.
84. *R: Yea I have understood but I have a question.*
85. I: Yes.
86. *R: Is it HIV positive people that are supposed to take PrEP?*
87. I: No it’s HIV negative people.
88. *R: Okay, now I understand. I feel that the problem can be the prescription because some people can find it hard to be taking drugs every day.*
89. I: Yes, that’s a condition that they need to be taking the drugs every day. So is that a concern to you?
90. *R: Yes, it’s a concern because like in my case, I can find it hard to be taking the drugs every day. I can even forget in the first months because I might not take it seriously consider.*
91. I: Do you feel it’s necessary for PrEP to be made available to people who are HIV negative?
92. *R: It’s necessary because it will help protect people from HIV.*
93. I: If PrEP becomes available, what is your opinion on integrating PrEP with Voluntary Medical Male circumcision services?
94. *R: If PrEP is available, the proper procedure can be that it should be made available in the cervical cancer screening room because in there will be couples. So that way it will be both of them getting the drugs*
95. I: If PrEP becomes available, what is your opinion on integrating PrEP with Voluntary Medical Male circumcision services?
96. *R: I can be pleased with such integration.*
97. I: What concerns can you have about this integration?
98. *R: My concern is that we don’t take drugs when we are not sick. So taking such drugs when you are okay can cause other illnesses. So people can be taking PrEP and be protected but that can also cause other problems in tier bodes since they are taking drugs when they are not sick.*
99. I: How can such concerns be dealt with?
100. *R: People should be taking PrEP but there should be a limited number of days when they can be taking the pills not like every day because that can be very difficult.*
101. I: It’s because it’s protecting them.
102. *R: Yes but still.*
103. I: With this PrEP you can be having unprotected sex with your partner who is HIV positive and still remain negative…
104. *R: Oh okay.*
105. I: Alright. If you were given powers to choose and integrate services in Voluntary Medical Male Clinics, what are the services that you would think of Integrating?
106. *R: Cervical cancer screening and family planning are the ones which I feel like they can be integrated with VMMC because in time everyone will be coming to the clinic with his partner so I feel that they should be accessing such services as a couple rather than be sent to other clinics for such services when they need them. It can be hard for couples to create a separate day to come for testing so taking advantage of the days which they already are at the clinic can help*
107. I: What other services apart from the ones we have discussed here can be integrated with VMMC?
108. *R: I can’t think of anything because most of the services I can think of are already there, like HIV testing which is already done when one comes for VMMC.*
109. I: Okay. How about other services which we have not talked about here?
110. *R: The ones I was thinking are family planning, cervical cancer screening and PrEP because these are the kinds of services which most people here in Malawi find it difficult to go to hospitals for because they feel like they are not really necessary. So integrating cervical cancer screening can be good for those that have difficulties with transport and family planning can be good for youths and women. So these are important services which should be integrated.*
111. I: Thank you for taking your time to discuss with me today. Your answers will be very helpful in improving the health service delivery at circumcision clinics. Before we close, do you have anything to say?
112. *R: Mine is just an advice to my fellow youths because the future of this country is in our hands. So we need to follow medical instructions so we can have meaningful life. We need to come for VMMC, receive PrEP so we can protect ourselves from HIV.*
113. I: I really appreciate.
114. *R: Thank you.*
115. I: Yeah.

END

**D 43 STUDY**

**Date of Interview: 12 September 2018**

**Type of Participant: Male Index Participant**

**Interview Number: D-43-0057**

**Interviewer: I. N.**

**Total Interview Time: 32 minutes 59 seconds**

**Interview Summary:** **(from summary sheet)**

| **SERVICE TO BE INTERGRATED** | **THOUGHTS ON INTERGRATION** |
| --- | --- |
| Couple HIV Testing and Counseling | Could help couples know their HIV status together. |
| STI Services | Would help couples to get treatment together. |
| Family Planning | Youths will have a chance to get family planning in a private space |
| Cervical Cancer Screening | Important to integrate so that women can know if they have the cancer or not. |
| PrEP | Important to be integrated and be given to the youths |
| Other Services | Thinks cervical cancer screening , family planning and PrEP are the best to be integrated into VMMC |

**Remarks:**

**Participant was relaxed, quick to respond to questions and straight to the point when responding.**

**Interview Text:**

1. I: Thank you for taking the time to talk with me today. I would like to ask you some questions today about the way you feel and what you think about some issues related to the service you receive here and how we can include other services in Voluntary Medical Male Circumcision clinics. There are no right or wrong answers to these questions. We would like to hear your opinion and your experiences in your own words. Do you have any questions before we begin?
2. *R: Questions concerning VMMC?*
3. I: Yes. Like do you have anything you want to say before we begin?
4. *R: Aah no.*
5. I: So the first question is: Do you ever talk to your care providers about how the services are provided here?
6. *R: Yes, because they usually talk to us about the benefits of VMMC before we go for surgery, then we go to HTC room where they talk about HIV and STIs. Then we go to the screening room where they test us for STIs. So yes we talk to them.*
7. I: Can you give me an example of what you talked when you came?
8. *R: Like after I came out of the surgery we didn’t talk about anything with them*
9. I: Ok. How about at check up?
10. *R: During checkup they just said that the wound was clean and that I was supposed to be cleaning it with salt. That’s all.*
11. I: Alright, so I want us to talk about partner HIV testing here at the circumcision clinic. What happens when a man brings a partner at the Voluntary Medical Male circumcision clinic?
12. *R: When a man brings his partner to the clinic the woman enters with him in the screening room where they talk about wound care after surgery. So the woman being there helps her to understand that her husband needs some time without sex and wound care. It also helps her to understand the benefits of VMMC that it reduces the risk of HIV infection by 60% and also reduces the risk of penile cancer.*
13. I: Ok.
14. *R: Yes.*
15. I: Or did you see anyone who brought a partner at the circumcision clinic?
16. *R: Yes, I have ever seen them but mostly it’s those that are married that bring their partners. For unmarried men like me it’s hard, of course we may want to bring them but sometimes it becomes hard to meet with them.*
17. I: Did you bring your partner to the VMMC clinic?
18. *R: No*
19. I: Ok. So what do you think is the reason that makes men to bring their partners at the Voluntary Medical Male circumcision clinic?
20. *R: I think what motivates them is the desire to do things together as a couple. It also helps like in the case of VMMC they learn about wound care and couple testing even though it’s not by force that they get HIV testing. So let’s say you have both been found HIV positive as a couple, they give you that message together and it becomes lighter rather than each of you to learn on your own. You get strong together and plan your life together.*
21. I: Ok. What do you think makes men not to bring their partners?
22. *R: It’s because some men think that their partners are less important in their lives so they don’t tell them about such issues. Some don’t open up about issues in their marriages so anything they do becomes something private. Some men even don’t tell their wives that they have been found HIV positive just because they don’t feel like they are important in their lives.*
23. I: Ok. So what do you think can be done so that more men bring their partners to the VMMC clinic?
24. *R: What’s needed is civic education because most men don’t know that they can bring their partners. Others feel that if they bring their partners then they will spread the message that the men have done VMMC. So civic education is very important so they can know the benefit of bringing their partners to the clinic. This message can be broadcasted on radios, on TV and any other media.*
25. I: Ok, you as Individual, what is your opinion on integrating couple HIV counseling with Voluntary Medical Male circumcision services?
26. *R: I think it’s a good idea because the couple can have a chance of getting tested together, being counseled together and they will know each other’s status as compared to one person coming for HTC and reporting the results So it’s good for them to be tested together and get VMMC as well*
27. I: Ok. So what do you think are barriers and concerns on this integration?
28. *R: The concerns that can be there are that some men may not feel comfortable to bring their partners to the VMMC clinic for fear that the partners will spread the message that the men have undergone VMMC. So some men may fail to come to the VMMC clinic for the surgery. The solution to this is civic education because when people are made aware of the benefits of coming to the clinic with their partners, they may be less fearful. Even when it comes to couple testing at normal clinics, some men fail to come with their partners for fear that if they are found HIV Positive then their marriage will be over*
29. I: Now I would like us to discuss about sexual reproductive health services and Pills for HIV prevention: called pre-exposure prophylaxis. (PrEP) Sexual reproductive health includes services that promote good sexual health and reproduction. They include but not limited to family planning, cervical cancer screening sexual transmitted infection management cervical, condom distribution and many more. Today we will only discuss family planning, Sexual transmitted infection management cervical screening and PrEP. We will look at each of these one by one. Let us start with: screening and sexual transmitted infections. Explain to me, what you have ever learnt about STIs when you came to Voluntary Medical Male circumcision clinic? Explain to me, what you have ever learnt about STIs when you came to Voluntary Medical Male circumcision clinic?
30. *R: I have learnt that you can contract Gonorrhea, Syphilis and other STIs when you have unprotected sex with a person who has the STIs. Others say if you are bruised and the person who is infected is bruised as well and you get in contact with them then you can also contract STIs.*
31. I: What did they tell you would happen in case they suspect or actually test you positive for STIs?
32. *R: For infections like HIV, I heard that they don’t stop you from getting VMMC only that they put you on ART and give you ways to protect yourself and your partner*
33. I: So what is your opinion on integrating STI services with Voluntary Medical Male Circumcision services, so that both of you should be coming here so that the process can take place while you are together with your partner?
34. *R: I feel that VMMC is important because it reduces the risk of HIV infection by 60% as compared to someone who has not done VMMC. So if one can use protection like condoms then he can be fully protected. This shows that VMMC plays a great role in protecting people against HIV infections. Another issue is that it promotes cleanliness of the penis.*
35. I: What is it that you do not like about integrating STI services in Voluntary Medical Male circumcision services?
36. *R: There is nothing that I would not like about this integration because these services are related. What’s needed is civic education to the people so they can understand why this is happening because some people can just decline getting the service just because they don’t understand it. But it’s important that this integration should be there.*
37. I: How do you think is the best way to offer STI services at the Voluntary Medical Male Circumcision clinics?
38. *R: They can be talking about the STI service at the HTC room and the screening room. They can also do the testing there if it’s required that they perform the tests and give treatment when needed. I remember they told us in the screening room that if you are found with an STI they don’t do the surgery right away until you take treatment and your condition becomes better. So giving people such information before the surgery can really help them to understand than telling them they have an infection after the surgery.*
39. I: What do you think are the barriers and concerns on this integration of STI services with Voluntary Medical Male circumcision services?
40. *R: The barriers can be there like lack of openness of clients to the doctors because say they have diagnosed you with syphilis or gonorrhea, it’s required that you come to the clinic a certain number of times for you to complete your treatment. Now some people may choose not to come back after just one dose. But still if the people are given enough information they can be coming for the subsequent doses.*
41. I: Alright. So, let us now talk about family planning; Explain to me about anything that you know about family.
42. *R: Well, what I know about family planning is that it prevents a woman from getting pregnant. There are several methods like condoms, pills, vasectomy for men and Bilateral Tubal Ligation for women. These are the types of family planning methods that help.*
43. I: What is your opinion about integrating family planning with VMMC?
44. *R: That’s a good idea because most men don’t like accompanying their wives for medical care even if it’s a serious issue. Even if they know the wife is going to (name of clinic), they still deny escorting them. So integrating family planning in the VMMC clinic will make the men listen to information about family planning. If they are given enough information they can change their perceptions about family planning because there are some people who believe that it’s sinful to use family planning because God said, we should multiply like the grains of sand. So integrating family planning with VMMC can provide them with enough and useful information.*
45. I: What would make you not want integrating Family Planning services in Voluntary Medical Male Circumcision services?
46. *R: Nothing.*
47. I: Ok. How do you think is the best way to offer family planning services within Voluntary Medical Male circumcision services clinics?
48. *R: After we were done with HTC they were teaching us about correct condom use. So that can be the right time to talk about family planning by telling the people “This condom protects one from infections and pregnancies but there are also other family planning methods that are available like…” So the men can know that they can combine condom use with family planning.*
49. I: Alright. What do you think could be the barriers or concerns on family planning and Voluntary Medical Male circumcision integration?
50. *R: I don’t think there can be any concerns apart from that some men may not feel comfortable with the integration. But I don’t think there can be real issues.*
51. I: Okay, so what can be done so men can be comfortable with this?
52. *R: Civic education can help give the men information. Even when we are at the church and the pastor just says “You should stop doing this” I don’t think people can remain in that church. So going into the villages and performing some plays can attract the people. Even role plays that are broadcasted in radios can also help to spread the message. If you do the plays, the people can listen and spread the information among themselves and the men will be able to know what family planning is and the procedures here rather than for them to just find out that such services are being offered here while they are already here.*
53. I: Ok. Let’s now talk about cervical cancer. Have you ever heard about cervical cancer screening?
54. *R: I don’t know anything about cervical cancer screening, so there is nothing I can say about that.*
55. I: Do you know anything about cervical cancer?
56. *R: Yes, I have ever heard about it in radios and other avenues.*
57. I: What did you hear about cervical cancer?
58. *R: That when a man gets VMMC he also protects his wife from cervical cancer but I don’t know what really causes cervical cancer.*
59. I: As an individual, what is your opinion on integrating partner cervical cancer screening with Voluntary Medical Male circumcision services? Say, you come with your partner and she is screened for cancer?
60. *R: It is important that they integrate so that the partners can also benefit when they escort the men to the VMMC clinic. If the women just stay home, they can’t know whether they have the disease or not but when they come here they will be screened and will be able to know whether they have the disease or not. If they stay home it can be difficult for the women to make plans to go to a clinic for screening but when they come here, they can easily do that while escorting their partners*
61. I: What is it that would make you not want integrating cervical cancer screening with Voluntary Medical Male Circumcision?
62. *R: There is nothing that would make me not to like the integration.*
63. I: Ok, so how do you think is the best way to offer Cervical Cancer screening services within Voluntary Medical Male circumcision clinics?
64. *R: Since this is a men’s issue…there is a certain section where they do screening. So if maybe there could be a separate room where they can be doing the family planning and cervical cancer screening,*
65. I: Ok. What do you think could be the barriers or concerns with partner cervical cancer screening and Voluntary Medical Male circumcision integration?
66. *R: I don’t see any barriers or concerns.*
67. *R: Sure.*
68. I: Now Let us discus about PrEP. Have you heard about PrEP?
69. *R: Yea they say PrEP or PEP?*
70. I: It’s PrEP.
71. *R: Yea they say if maybe you were involved in an accident or you had unprotected sex with someone, you need to go to the hospital and get PrEP so that the HIV virus in your body can die. That’s what I heard.*
72. I: Okay so that’s PrEP, it’s called post exposure prophylaxis.
73. *R: Oh okay. How about PrEP?*
74. I: That’s Pre Exposure Prophylaxis which you take before you get exposed or before you have unprotected sex while PEP you take after unprotected sex.
75. *R: Oh, I have never heard about that.*
76. I: I will explain it to you. PrEP are medicines that help to prevent HIV, they help in sense that a person who is HIV negative should not contract HIV. So they are medicines that when you take, you do not contract HIV. The dose is, a person swallows one pill per day. One pill per day, daily. And if you are following the instructions, this pill works very effectively in helping people prevent from getting HIV. So how do you feel about PrEP?
77. *R: I have understood and I feel that PrEP is much better than PEP and if it’s to be integrated into VMMC clinic then it can be a good thing because when we come to the VMMC clinic then we will take advantage of that visit to get PrEP and condoms. So it’s really useful.*
78. I: Do you feel it’s necessary for PrEP to be made available to people who are HIV negative?
79. *R: Yes, it’s necessary*
80. I: If PrEP becomes available how can it be offered in the VMMC clinic?
81. *R: It can be offered after HTC and after you have learnt your status for those that want to get the drugs.*
82. I: What concerns can you have about this integration?
83. *R: My concern if people are taking PrEP is that people will think they are safe and cannot protect themselves from pregnancies. SO there can be lots of unwanted pregnancies and school dropouts. However, since we talked about family planning I think it can be good if such services are provided at youth-friendly spaces because you know youths are difficult, they may not have a girlfriend but still be having sex around. So there should be a special program for the youths so they can be accessing such services.*
84. I: So that way these concerns can be dealt with?
85. *R: Yes, because say they have done family planning at such spaces, we will be assured that they are protected.*
86. I: Alright, let’s talk about the last part of the discussion which is other services. If you were given powers to choose and integrate services in Voluntary Medical Male Clinics, what are the services that you would think of Integrating?
87. *R: Other services like?*
88. I: Any which you can think of.
89. *R: I don’t know if STI drugs are available at the VMMC clinic? Or is it also part of what we have talked about here?*
90. I: Yes, it’s part of STI management and diagnosis.
91. *R: Okay so that one is also important.*
92. I: What other services apart from the ones we have discussed here can be integrated with VMMC?
93. *R: Maybe like counseling…but I think that’s not necessary because we already get counseled after HTC. But I feel PrEP and family planning really need to be integrated in the VMMC clinic.*
94. I: Okay. Why do you say they are important?
95. *R: It’s because here in Malawi many young people get pregnant. So this can help us to have a special place for ourselves and protect ourselves from unwanted pregnancies. Because if a standard 8 girl gets pregnant, that means her future is ruined and the future of the country is ruined as well. So this special place could be of real help to us because youths make mistakes most of the times and we might think we are doing the right things when in actual sense we are not.*
96. I: Thank you for taking your time to discuss with me today. Your answers will be very helpful in improving the health service delivery at circumcision clinics. Before we close, do you have anything to say?
97. *R: I feel what you are doing is a good program but I don’t know if you are only here at Bwaila?*
98. I: Yes, it’s only done here at Bwaila for now.
99. *R: Okay so I feel that this program is very good and it will help the youth a lot because we will have our own space. Imagine even when it comes to VMMC and our parents are there, we don’t tell them that we also have done VMMC or we want to because of shyness but now things will be better.*
100. I: Alright thank you so much for your time.
101. *R: Thank you.*
102. I: Yeah.

END

**D 43 STUDY**

**Date of Interview:**

**Type of Participant: Male Index Participant**

**Interview Number: D-43-0059**

**Interviewer: I.N.**

**Total Interview Time: 32 minutes 28 seconds**

**Interview Summary:(from summary sheet)**

| **SERVICE TO BE INTERGRATED** | **THOUGHTS ON INTERGRATION** |
| --- | --- |
| Couple HIV Testing and Counseling | Good idea. Couples can know their status |
| STI Services | Thinks it is good to integrate because people will know their status. |
| Family Planning | Family planning should not be integrated with VMMC. |
| Cervical Cancer Screening | Good initiative. Women will benefit from cervical cancer screening when they accompany their partners to VMMC. |
| PrEP | It is a good idea because people will protect themselves from HIV. |
| Other Services | None. |

**Remarks:**

**Participant understood most of the questions and was open-minded about issues.**

**Interview Text:**

1. I: Thank you for taking the time to talk with me today. I would like to ask you some questions today about the way you feel and what you think about some issues related to the service you receive here and how we can include other services in Voluntary Medical Male Circumcision (VMMC) clinics. There are no right or wrong answers to these questions. We would like to hear your opinion and your experiences in your own words. Do you have any questions before we begin?
2. *R: Right now I don’t have any question.*
3. I: Do your ever talk to your care providers about how the services are provided here at VMMC clinic?
4. *R: We don’t talk much…*
5. I: But you still talk?
6. *R: Yes we do talk.*
7. I: Like asking each other questions and the like?
8. *R: Yes we do talk.*
9. I: Like when you went for a checkup you discussed?
10. *R: Yes we did.*
11. I: Can you give me an example of a time when you managed to talk to your health care provider about the services you received here?
12. *R: On the first day we discussed that when a person is circumcised, you reduce the risk of getting HIV.*
13. I: What did you discuss when you came for checkup about the services you received here?
14. *R: We did not talk much. They just told me how I should take care of my wound.*
15. I: Did you not ask any questions?
16. *R: I was not able to ask.*
17. I: Did they not ask you any question about the services here?
18. *R: They asked me how I am fairing…*
19. I: What did s/he say?
20. *R: He said that it is normal to feel a slight pain.*
21. I: Okay. Now let us talk about partner HIV testing here at the circumcision clinic? Have you ever seen a man coming together with his female partner here at the time you came for circumcision?
22. *R: Okay, what happens is that they come as a coupe and they go to a different room and they test both of them for HIV. After some two minutes, they give them the results.*
23. I: Have you ever seen a couple coming here at VMMC?
24. *R: Yes, I did.*
25. I: What role does the partner take?
26. *R: The partner benefits because they both get tested for HIV.*
27. I: Did you bring your partner when you were coming for VMMC?
28. *R: No, I did not.*
29. I: Why did not bring her?
30. *R: I am not married.*
31. I: What about a girl friend?
32. *R: I also don’t have.*
33. I:What do you think motivated the men that brought their partners here to do so?
34. *R: Because they also test for HIV. So they are able to take their partners to come for HIV testing so that they can know their status.*
35. I: How about those who don’t bring their partners, what made them not to bring them?
36. *R: I cannot be able to answer that one.*
37. I:What do you think make them not to bring their partners?
38. *R: That is not a good thing because when they come here as a couple, they are able to know their HIV status.*
39. I:How do you think we can help men to bring their partners here at VMMC for couple counseling and testing?
40. *R: There is need to encourage the men to come here at VMMC for couple HIV testing and counseling.*
41. I: How do you think we can encourage the men?
42. *R: [Takes time to respond]… I have failed to answer that one…*
43. I:What is your opinion on integrating couple HIV testing and counseling with Voluntary Medical Male circumcision services?
44. *R: That is a good idea because when we come for circumcision, we are also tested for HIV and if HIV testing and counseling can be done while your partner is there, it can be a good idea.*
45. I:What do you think are the barriers and concerns on this integration?
46. *R: There should be no barriers or concerns.*
47. I:Now, I would like us to talk about sexual reproductive health services and Pills for HIV prevention: called pre-exposure prophylaxis. (PrEP) Sexual reproductive health includes services that promote good sexual health and reproduction. They include but not limited to family planning, cervical cancer screening sexual transmitted infection management cervical, condom distribution and many more. So today we will only discuss family planning, Sexual transmitted infection management cervical screening and PrEP. We will look at each of these one by one.Let us start with STI services.Explain to me, what you have ever learnt about STIs when you came to Voluntary Medical Male circumcision clinic?
48. *R: We have learned that STIs are like HIV, gonorrhea, syphilis and bubbles.*
49. I: What did the staff say would happen if you were found or suspected with STIs... before circumcision?
50. *R: They said that if you are diagnosed with STIs, you cannot be circumcised, they would first treat the STI and circumcise you after.*
51. I: What is your opinion on integrating STI services with Voluntary Medical Male Circumcision services?
52. *R: That is a good idea because it will help the people to know their status.*
53. I:What would make you to like the integration?
54. *R: [No response]…*
55. I:What would make you to like the integration?
56. *R: Mm… it is difficult to answer that one.*
57. I: How do you think screening for STIs should be done?
58. *R: It should be done when they are doing the counseling. Because when you have just been circumcised, you cannot be listening to the messages but before they circumcise you, they should counsel you and give you all that information.*
59. I: Where should STI screening be done?
60. *R: It should be in a private place within the clinic.*
61. I: What makes you think that way?
62. *R: Because sometimes there are young boys which would be shameful to older men to be seen by young men.*
63. I: What do you think can be the barriers or concerns of integrating STIs in VMMC?
64. *R: I don’t see any.*
65. I: Fine. Now let us talk about family planning. Explain to me about anything that you know about family planning; whether vasectomy or family planning for women…?
66. *R: The issue of family planning should be done by women. It can be difficult for a man to do family planning.*
67. I: How do women do family planning?
68. *R: When a woman is injected family planning drugs, a man is not able to impregnate her.*
69. I: What methods of family planning do you know of?
70. *R: There are pills, using condoms… you cannot impregnate a woman…*
71. I: There is another one called in-plant, loop, depo… that is in terms of women right?
72. *R: Okay…*
73. I: And there is condoms as you have mentioned, pills, injection and there is vasectomy for men… So, what is your opinion on integrating Family planning in Voluntary Medical Male circumcision services/clinic?
74. *R: Can you come again?*
75. I:What is your opinion on integrating Family planning in Voluntary Medical Male circumcision services/clinic?
76. *R: Family planning should not be integrated with VMMC.*
77. I: What makes you feel that way?
78. *R: Because family planning is for women and they need to have their own place for that.*
79. I: What about men who want to do family planning?
80. *R: Men who want to do family planning can do it at VMMC but they should have their own room.*
81. I: So what is your opinion on integrating Family planning in Voluntary Medical Male circumcision services/clinic?
82. *R: It can be integrated.*
83. I: But you don’t want men to do family planning?
84. *R: I am in the group of men who don’t want to do family planning.*
85. I:What makes you not want to do family planning?
86. *R: As you know I am a young man; I cannot do family planning now when I have no child. Maybe in the future after I have two or three children I can do family planning.*
87. I: So, how do you think family planning services be provided?
88. *R: It should be provided where the men get circumcised so that while they are being circumcised, they should also be given family planning method.*
89. I:What can be the concerns or barriers of integrating family planning with VMMC?
90. *R: Integrating family planning with VMMC?*
91. I: Yes…
92. *R: I don’t think there can be any barriers or concerns because family planning is one way of reducing overpopulation.*
93. I:In your opinion, what do you think can be the barriers of integrating family planning with VMMC since you have said that you cannot do family planning?
94. *R: I feel it cannot be good to integrate family planning with VMMC…*
95. I: In what way?
96. *R: Like for me as a young man, it can be difficult to do family planning because I have never had a child.*
97. I: So, what do you think can be done to address those concerns?
98. *R: [Takes time to respond]… I have failed that one.*
99. I: Now, we should talk about cervical cancer screening; what do you know about cervical cancer screening?
100. *R: [Takes time to respond]… I have no idea about that.*
101. I: Have you ever heard about cervical cancer?
102. *R: Yes, I have heard doctors talk about it.*
103. I: What did they say?
104. *R: I heard that when we are circumcised, it reduces the risk of cervical cancer in women.*
105. I:Have you heard about breast cancer, Kaposi’s sarcoma… those cancers…
106. *R: I have ever heard about them.*
107. I: So, cervical cancer affects the cervix…
108. *R: Okay…*
109. I: So, they do screening like the way you are screened for diseases like STIs, they also screen women for cervical cancer.
110. *R: Okay…*
111. I: So, what is your opinion of integrating cervical cancer screening with VMMC?
112. *R: It is good to integrate the service because the woman will be accompanying the man when he goes for VMMC.*
113. I:What makes you feel that way?
114. *R: Because a woman also takes part in VMMC services and she can benefit from cervical cancer screening.*
115. I: What would make you not like this service?
116. *R: This is a good service. It should be integrated.*
117. I: How do you think this service should provide at VMMC clinic; when is the best time to offer this service??
118. *R: I feel the best time is when the couple is being tested for HIV. That is the best time to screen the woman for cervical cancer because you will be counselled together.*
119. I:What do you think can be the barriers in integrating cervical cancer screening with VMMC?
120. *R: I don’t know how to answer that one.*
121. I: What could be the concerns?
122. *R: There are no concerns.*
123. I: Okay. Now let us talk about PrEP. Have you ever heard about PrEP?
124. *R: No, I have never heard about it.*
125. I: Okay, I will explain to you; PrEP is different in a way that you don’t just take it on the days when you have unsafe sex. It s a pill that you take once every day so you can remain HIV negative. SO PrEP is an anti-HIV drug which helps to keep HIV-negative people negative. There is a pill that one needs to take every day to prevent HIV infection.
126. *R: Fine…*
127. I: Have you understood?
128. *R: Yes.*
129. I:So how do you feel about PrEP?
130. *R: That is another good way of protecting yourself from HIV.*
131. I: Do you think it’s necessary for PrEP to be made available to HIV-negative men and women?
132. *R: It is necessary.*
133. I: Why?
134. *R: Because it can help men and women to be protected from HIV.*
135. I:If PrEP is available, what is your opinion of integrating PrEP in VMMC?
136. *R: It is good to integrate PrEP in VMMC because people can be protected from HIV.*
137. I: So, how do you think PrEP can be provided at VMMC in terms of place, time?
138. *R: It should be provided during the time when they give us the pills; while they are giving us those pills after circumcision, they should also provide PrEP.*
139. I: What concerns do you have if people are receiving PrEP?
140. *R: There are no concerns.*
141. I: Now let us talk about other services… If you were given powers to choose and integrate services in Voluntary Medical Male Clinics, what are the services that you would think of Integrating that we have not talked about?
142. *R: [Takes time to respond] Those that we have not talked about?*
143. I: Yes. If you were given powers to choose and integrate services in Voluntary Medical Male Clinics, what are the services that you would think of Integrating?
144. *R:* [No response]….
145. I:Mh..?
146. *R: [No response]…*
147. I: Yes…
148. *R: It is difficult to answer that one.*
149. I:Thank you very much for your time. Your answers will be very helpful in improving the health service delivery at circumcision clinics.Before we close, is there anything more you would like to say?
150. *R: My comment is that male circumcision is good because it helps to reduce the risk of getting HIV.*
151. I: Do you have other comments?
152. *R: No.*
153. I: Thanks you very much for your time. This marks the end of our discussion.
154. *R: Thank you.*

END
