# Supplementary material for: Opinions on integrating couple counselling and female sexual reproductive health services into Voluntary Medical Male Circumcision services in Lilongwe, Malawi
Source: PLoS One. 2022 Sep 9;17(9):e0273627. doi: 10.1371/journal.pone.0273627 (PMC9462804; doi:10.1371/journal.pone.0273627)
Supplement: S4 File — (DOCX) [file pone.0273627.s004.docx]

**Interview guide for Male Index**

Zikomo potenga nthawi kulankhula ndi ine lero. Ndikufuna ndikufunseni mafunso ena okhudza ndi mmene mukumvera/mukuganizira pa zinthu zina zokhudzana thandizo limene mukulandira pano ndi mmene mukuganizira pa za kuonjera thandizo lina pamwamba pa thandizo la mdulidwe wa abambo wa kuchipatala(VMMC). Palibe yankho lokhoza kapena lolakwika ku mafunsowo. Tikufuna timve maganizo anu ndi zokumana nazo ndi pakamwa panu. Muli ndi mafunso ena alionse tisanayambe?

1. Kodi mumatha kukambirana ndi wopereka thanzo za mmene thandizo limaperekedwera pa kiliniki ino?
2. Tandifotokozerani chitsanzo cha nthawi imene munatha kukambanawo za mmene munalandiria thandizo pa kiliniki ino?

**Tsopano tiyeni tikambirane zokhudza kuyezetsa HIV kwa wokondedwa wanu konkuno ku kiliniki ya mdulidwe wa abambo wa kuchipatala?**

1. Tandifotokozerani, kodi chimachitika ndi chiyani abambo akabweretsa wokondedwa wao kuno ku kiliniki ya mdulidwe wa abambo wakuchipatala?
2. Nanga wokondedwa amatengapo mbali yanji pa thandizo limene abambo amalandira kuno ku kiliniki ya mduludwe wa abambo wa kuchipatala?
3. Ngati inu munabweretsa wokondedwa wanu, chinakulimbikitsani ndi chiyani kuti muwabweretse?
4. Ngati simunabweretse, chinakupangitsani ndichiyani kuti musawabweretse kuno wokondedwa anu?
5. Mmaganizo anu, mukuganizapo zotani zobweretsa wokondedwa wanu kudzayezetsa magazi ku kachirombo ka HIV kuno ku kiliniki ya mdulidwe wa abambo wa kuchipatala?
6. Kodi mukuganiza kuti tichite chiyani kuti amuna athe kubwera ndi wokondedwa awo kuno kuti adzayezetse HIV limodzi?
7. Monga inu panokha, mukuganizapo bwanji pa zophatikizapo kuyezesa HIV ngati banja pa ndondomeko ya thandizo la mduluidwe wa abambo wa kuchipatala?
8. Mukuganiza kuti pangakhale zolepheretsa kapena nkhawa zanji pa ndondomeko ya kuphatikiza thandizoli?
9. Mukuganiza kuti pakuyenera kuchita chiyani kuti tithetse nkhawa ndi zolepheretsa za kuyezetsa ngati banja ku kiliniki ya mdulidwe wa abambo wa kuchipatala?

**Tsopano ndikufuna kuti tikambirane zokhudzana ndi thandizo a uchembere ndi mapilitsi woteteza ku HIV wotchedwa kuti PrEP**

**Uchembere wabwino ukuthandauza thandizo limene likuphatikizapo kugonana komanso kubereka/kuchembeza kwabwino. Zikuphatikizapo koma wosalekezera pomwepa kulera, kuunika khansa ya khomo la chiberekero, kuunika ndi kuthandiza matenda wopatsirana pogonana, kugawa makondomu ndi zina zambiri.**

**Lero tingokambirana za kulera, kuunika ndi thandizo la matenda wopatsirana pogonana, kuunika khansa ya khomo la chiberekero ndi PrEP. Tikambirana chilichonse pachokha pachokha: Tiyeni tiyambe ndi:**

**1, Kuunika ndi thandizo la matenda wopatsirana pogonana**

1. Tandilongosolereni za zomwe mwaphunzirapo za matenda opatsirana pogonana,mmene mwabwera kuno ku kiliniki ya mdulidwe wa abambo wa kuchipatala?

**Probe:**

*Kodi ogwira ntchito yachipatala anati chitachitike ndi chiyani mukaganiziridwa kapena kupezeka ndi matenda opatsirana pogonana*

1. Monga inu panokha, mukuganizapo bwanji pa zophatikiza kupereka thandizo la matenda opatsirana pogonana ndi mduluidwe wa abambo wa kuchipatala?
2. Kodi mukuona kuti ndi njira yiti yabwino yoperekera thandizo la matenda wopatsirana pogonana kuno ku kiliniki ya mdulidwe wa abambo wa kuchipatala?

**Probes**:

1. ***Kodi zimene zingakutsangalatseni kuti thandizo la kuunika ndi kuthandiza matenda opatsirana pogonana kuti liphatikizane ndi thandizo la mdulidwe wa abambo wa kuchipatala ndi chiani?***
2. *Nanga ndi zinthu zanji zimene singakutsangalatseni pankhani ya kuphatikiza thandizo la matenda wopatsirana pogonana ndi mdulidwe wa a bamboo wa kuchipatala?*
3. Kodi mukuganiza kuti pali zolepheretsa komanso nkhawa zanji pa kuphatikizana kwa thandizo la matenda wopatsirana pogonana ndi mdulidwe wa abambo wa kuchipatala?
4. Mukuganiza kuti t**ingatani** kuti tithetse nkhawa ndi zolepheretsazi?

**2.0 Kulera**

1. Tandifotokozereni zokhudza china chirichonse chimene mukudziwapo cha nkhani ya kulera (kutseka kwa abambo ndiponso kulera kwa amai).
2. Monga inuyo panokha, muli ndi maganizo anji pa nkhani yakuti aphatikizepo kulera pa thandizo la mdulidwe wa abambo wa kuchipatala/kiliniki ya mdulidwe?

**Probes:**

1. *Kodi zimene zingakutsangalatseni kuti thandizo la kulera liphatikizane ndi thandizo la mdulidwe wa abambo wa kuchipatala ndi chiani? mungasangalatsidwe bwanji kubwera ndi kumadzalandira thandizo.*
2. *Ndichiyani chimene chingakupatseni kusafuna mphatikizo wa thandizoli mu ndondomeko ya mdulidwe wa abambo wa kuchipatala*
3. Mukuganiza kuti ndi njira ziti zabwino zoperekera thandizo la kulera muno mu kiliniki ya mdulidwe wa abambo wa kuchipatala?
4. Mukuganiza kuti pali zolepheretsa kapena nkhawa zanji pa kuphatikizana kwa kulera ndi Mdulidwe wa abambo wa kuchipatala?
5. Mukuganiza kuti nkhawa ndi zoletsazi zingathetsedwe bwanji?

**Kuunika khansa ya khomo lachiberekero**

1. Tandifotokozereni za zimene mukudziwa za kuunika khansa ya khomo lachiberekero
2. Monga inuyo panokha, muli ndi maganizo anji pa kuphatikiza thandizo la kuunika wokondedwa anu khansa ya khomo la chiberekero ndi thandizo la mdulidwe wa abambo wa kuchipatala?

**Probes:**

1. *Ndichiani chimene chingakusangalatseni pa mphatikizo wa thandizo la kuunika khansa ya khomo la chiberekero ndi thandizo la mdulidwe abambo wa kuchipatala?* Mungasangalatsidwe bwanji kubwera ku thandizo?
2. *Ndichiyani chimene chingakupatseni kusafuna mphatikizo wa thandizo la kuunika khansa ya khomo la chiberekero ndi thandizo la mdulidwewa abambo wa kuchipatala?*
3. Mukuganiza kuti ndi njira ziti zabwino zoperekera thandizo la kuunika khansa ya khomo la chiberekero kwa wokondedwa anu mu kiliniki ya mdulidwe wa abambo wa kuchipatala?
4. Mukuganiza kuti pangakhale zolepheretsa kapena nkhawa zanji pa kuphatikiza thandizo la kuunika khansa ya khomo la chiberekero kwa okondedwa anu ndi Mdulidwe wa abambo?
5. Mukuganiza kuti nkhawa ndi zolepheretsazi zingathetsedwe bwanji?

**TSOPANO tiyeni tikambilane za PrEP**

Munayamba mwamvako zokhudza PrEP?

Ngati munamvako zokhudza PrEP,Chonde ndiuzeniko zimene mukudziwa zokhudza PrEP?

1. Munadziwa bwanji za zimenezi?
2. Mumamva bwanji mtimamu za zimenezi?

Ngati simunamvepo zokhudza PrEP, ndikufotokozerani mmene mankhwala amagwirira ntchito. PrEP ndi mankhwala wothandiza ku HIV amene amapangitsa anthu amene alibe HIV kuti asatengere. Pali pilitsi limodzi limene limamwedwa kamodzi pa tsiku tsiku lililonse ndipo ukamamwa nthawi ndi nthawi mwandondomeko limagwira ntchito bwino kwambiri kuteteza anthu kutengera kachirombo.

Tsopano mukumva bwanji zokhudza PrEP?

**Kodi nkofunika kuti PrEP akhale akupezeka kwa amai ndi abambo amene alibe kachirombo ka HIV?**

**Ngati ai ;** Nkhawa zanu ndizotani?

Ngati inde,

1. Ngati PrEP akhale akupezeka, maganizo anu ndi otani pa nkhani yophatiza mundondomeko ya thandizo la mdulidwe wa abambo wa kuchipatala? Mungatsangalatsidwe motani kulandira nawo?
2. Mukuganiza kuti PrEP angamaperekedwe bwanji pa kiliniki ino?
3. Muli ndi nkhawa zanji ngati anthu akulandira PrEP?
4. Ngati pali nkhawa mukuganiza pakuyenera kuchitika chiyani kuti athetse nkhawazi?

**Zithandizo zina**

Mutati mwapatsidwa mphamvu zosankha zithandizo zimene zitha kuphatizana pa kiliniki ya thandizo la mdulidwe wa abambo wa kuchipatala kodi ndi zithandizo ziti inu mungasankhe kuziphatikiza ?

1. Ndifotokozereni zifukwa zake pa zimene mwasankha.
2. Mukuganiza kuti thandizoli liziperekedwa bwanji mu kiliniki?

Zikomo kwambiri chifukwa chotenga nthawi yanu kukambirana zinthuzi lero. Mayankho anu athandiza popititsa patsogolo thandizo la zaumoyo mu makiliniki a mdulidwe wa abambo wa kuchipatala.

Tisanatseke, muli ndi chinachirichonse mukufuna kulankhulapo?

Zikomo kwambiri chifukwa cha nthawi yanu pokambirana nane.

**Interview guide for Men Index**

Thank you for taking the time to talk with me today. I would like to ask you some questions today about the way you feel and what you think about some issues related to the service you receive here and how we can include other services in Voluntary Medical Male Circumcision (VMMC) clinics. There are no right or wrong answers to these questions. We would like to hear your opinion and your experiences in your own words. Do you have any questions before we begin?

1. Do your ever talk to your care providers about how the services are provided here?
2. Can you give me an example of a time when you managed to talked to your health care provider about the services you received here?

**1.0 Couple HIV testing and counselling**

**Now let us talk about partner HIV testing here at the circumcision clinic?**

1. Tell me what happens when a man brings in a spouse at the Voluntary Medical Male circumcision clinic
2. How is the partner involved in the services that men received here at the clinic?
3. If you brought your spouse, what motivated you to bring her in
4. If you did not. What made you not to bring your partner here.
5. In your opinion, what are your thoughts about bringing in your partners for HIV testing at Voluntary Medical Male circumcision clinic
6. What do you think can be done to make men bring their partners here for HIV testing and counselling?
7. You as Individual, what is your opinion on integrating couple HIV counseling with Voluntary Medical Male circumcision services.
8. What do you think are the barriers and concerns on this integration?
9. What do you think should be done to overcome these concerns and barriers to couple counseling in Voluntary Medical Male circumcision clinic?

**Now I would like us to discuss about sexual reproductive health services and Pills for HIV prevention: called pre-exposure prophylaxis. (PrEP)**

**Sexual reproductive health includes services that promote good sexual health and reproduction. They include but not limited to family planning, cervical cancer screening sexual transmitted infection management cervical, condom distribution and many more.**

**Today we will only discuss family planning, Sexual transmitted infection management cervical screening and PrEP. We will look at each of these one by one. Let us start with:**

**2.0, STI services**

1. Explain to me, what you have ever learnt about STIs when you came to Voluntary Medical Male circumcision clinic?

**Probe:** *What did the staff say would happen if you were found or suspected with STIs*

1. As an individual, what is your opinion on integrating STI services with Voluntary Medical Male Circumcision services?
2. How do you think is the best way to offer STI services at the Voluntary Medical Male Circumcision clinics?

**Probes**:

1. *What is it that you would like about the integration of diagnosis and management of STI services in* Voluntary Medical Male *services?*
2. *What is it that you do not like integrating STI services in* Voluntary Medical Male circumcision  *services?*
3. What do you think are the barriers and concerns on this integration of STI services with Voluntary Medical Male circumcision services?
4. What do you think should be done to address these concerns and barriers?

**3.0 Family planning**

1. Explain to me about anything that you know about family planning (vasectomy for men and family planning for female partners.
2. As an individual, what is your opinion on integrating Family planning in Voluntary Medical Male circumcision services/clinic?

**Probes:**

- 1. *What is it that you would like about integrating Family Planning services in Voluntary* Medical Male *Circumcision?*

*Services? How happy would you be to come and access the services?*

- 1. *What would make you* ***not*** *want integrating Family Planning services in* Voluntary Medical Male *Circumcision services*

1. How do you think is the best way to offer family planning services within Voluntary Medical Male circumcision services clinics?
2. What do you think could be the barriers or concerns on family planning and Voluntary Medical Male circumcision integration?
3. How do you think these concerns and barriers be addressed?

**4.0 Cervical cancer screening**

1. Explain to me what you know about cervical cancer screening
2. As an individual, what is your opinion on integrating partner cervical cancer screening with Voluntary Medical Male circumcision services

**Probes:**

1. *What is it that would like about integrating cancer screening with* Voluntary Medical Male *Circumcision services?*
2. *What is it that would make you* ***not*** *want integrating cervical cancer screening with* *Voluntary* Medical Male *Circumcision*
3. How do you think is the best way to offer Cervical Cancer screening services within Voluntary Medical Male circumcision clinics?
4. What do you think could be the barriers or concerns with partner cervical cancer screening and Voluntary Medical Male circumcision integration?
5. How do you think these concerns and barriers be addressed?

**5.0 NOW Let us discus about PrEP**

Have you ever heard about PrEP?

If you have heard about PrEP, please tell me what you know about PrEP?

1. How did you learn about this?
2. How do you feel about this?

If you have not heard about PrEP, I will explain how the medicine works. PrEP is anti-HIV medicine that keeps HIV-negative people from being infected. There is a single pill that is taken once daily, and if you take it regularly, it is highly effective at prevention people from being infected.

Now, how do you feel about Prep?

Is it necessary to make PrEP available to HIV negative men and women?

**If No;** what are your concerns?

If yes,

1. If PrEP become available, what is your opinion on integrating PrEP with Voluntary Medical Male circumcision services? How happy would you be to access them?
2. How do you think PrEP would be offered in this clinic?
3. What concerns would you have about people taking PrEP?
4. What should be done to address these concerns?

**6.0 Other services**

If you were given powers to choose and integrate services in Voluntary Medical Male Clinics, what are the services that you would think of Integrating?

1. Explain to me what the reasons are for your choice.
2. How do you think these services should be offered in the clinic?

Thank you for taking your time to discuss with me today. Your answers will be very helpful in improving the health service delivery at circumcision clinics.

Before we close, do you have anything to say?

Again, thank you so much for taking your time to speak with me.

**Peers and Clinic Aides Interview guide**

Zikomo potenga nthawi kulankhula ndi ine lero. Ndikufuna ndikufunseni mafunso ena okhudza ndi mmene mukumvera/mukuganizira pa zinthu zina zokhudzana thandizo limene mumapereka ndi mmene mukuganizira pa za kuonjezera thandizo lina pamwamba pa thandizo la mdulidwe wa abambo wa kuchipatala (VMMC). Palibe yankho lokhoza kapena lolakwika pa mafunsowo. Tikufuna timve maganizo anu ndi pakamwa panu. Muli ndi mafunso tisanayambe?

**Mungandiuzeko kuti inuyo mumatengapo mbali yanji yathandizo limene limaperekedwa pa kiliniki ino?**

**Probes**

1. *Kodi anzinzanu amatha kulankhula nanu za thandizo limene limaperekedwa pa kiliniki ino?*
2. *Mungandiuzeko chitsanzo cha nthawi imene achinzanu analankhula nanu za thandizo analandira kuno.*

**1.0 Kuyezetsa HIV ngati Banja**

**Tsopano tiyeni tikambirane za kuyezetsa HIV ngati banja pa kiliniki ino ya mdulidwe wa abambo wa kuchipatala**

1. Tandifotokozerani, kodi chimachitika ndi chiyani abambo akabweretsa wokondedwa wao kuno ku kiliniki ya mdulidwe wa abambo wa kuchipatala?
2. Mukuganiza kuti, chimawapangitsa ndi chiyani kuti abambo abweretse wokondedwa awo kudzayezetsa HIV?
3. Mukuganiza kuti chimapangitsani abambo ndichiyani kuti asawabweretse wokondedwa awo kuno kudzayesetsa HIV
4. Kodi mukuganiza kuti tichite chiani kuti abambo athe kubweretsa wokondedwa awo kuno kudzayezesa HIV ngati banja
5. Kodi inuyo monga wothandizira anzanu, mukuganizapo bwanji pa zophatikizapo kuyezesta HIV ngati banja pa ndondomeko ya thandizo la mdulidwe wa abambo wa kuchipatala?
6. Mukuganiza kuti pangakhale zolepheretsa kapena nkhawa zanji pa ndondomeko ya muphatikizo wa thandizoli.
7. Mukuganiza kuti pakuyenera kuchita chiyani kuti tithetse nkhawa ndi zolepheretsa za kuyezetsa ngati banja ku kiliniki ya mdulidwe wa abambo wa kuchipatala?

**Tsopano ndikufuna kuti tikambirane zokhudzana ndi thandizo la uchembere ndi mapilitsi woteteza ku HIV wotchedwa kuti PrEP**

**Uchembere wabwino ukuthandauza thandizo limene likuphatikizapo kugonana komanso kubereka/kuchembeza kwabwino. Zikuphatikizapo koma wosalekezera pomwepa kulera, kuunika khansa ya khomo la chiberekero, kuunika ndi kuthandiza matenda wopatsirana pogonana, kugawa makondomu ndi zina zambiri.**

**Lero tingokambirana za Kulera, kuunika ndi thandizo la matenda wopatsirana pogonana, kuunika khansa ya khomo la chiberekero ndi PrEP. Tikambirana chilichonse pachokha pachokha: Tiyeni tiyambe ndi:**

- 1. **Kuunika ndi thandizo la matenda wopatsirana pogonana**

1. Tandilongosolereni kodi chimachitika ndi chiyani pano munthu akaganiziridwa kapena wapezeka kuti ali ndi matenda opatsirana pogonana?
2. Inuyo monga wothandizira anzanu, mukuganizapo bwanji pa nkhani ya zophatikizapo kuunika ndi thandizo la matenda opatsirana pogonana pa ndondomeko ya mdululidwe wa abambo wa kuchipatala?

**Probes**:

1. *Nanga ndi zinthu zanji zimene mukugwirizana nazo pankhani yakuphatikizana kuunika ndi thandizo la matenda wopatsirana pogonana ndi mdulidwe wa abambo wa kuchipatala?*
2. *Ndi zinthu zanji zimene simukugwirizana nazo pankhani ya kuphatikiza kuunika ndi thandizo la matenda wopatsirana pogonana ndi mdulidwe wa abambo wa kuchipatala?*
3. Kodi mukuona kuti ndi njira ziti zabwino zounika ndi zoperekera thandizo la matenda wopatsirana pogonana kuno ku kiliniki ya mdulidwe wa abambo wa kuchipatala?
4. Kodi mukuganiza kuti pali zolepheretsa komanso nkhawa zanji pa kuphatikizana kwa thandizoli?
5. Mukuganiza kuti nkhawa ndi zolepheretsazi zingathetsedwe bwanji?

**3.0 Kulera**

1. Tandifotokozereni kuti chimachitika ndi chiyani munthu akafuna njira yolera (monga kutseka kwa abambo ndiponso kulera kwa amai).
2. Inuyo monga wothandizira anzanu, mukuganizapo bwanji pa nkhani yakuti aphatikizepo kulera pa thandizo la mdulidwe wa abambo wa kuchipatala?

**Probes:**

1. *Kodi zimene zingakutsangalatseni kuti thandizo la kulera liphatikizane ndi thandizo la mdulidwe wa abambo wa kuchipatala ndi chiani?*
2. *Ndichiyani chimene chingakupatseni kusafuna mphatikizo wa thandizo la kulera mu ndondomeko ya mdulidwe wa abambo wa kuchipatala?*
3. Mukuganiza kuti ndi njira ziti zabwino zoperekera thandizo la kulera muno mu kiliniki ya mdulidwe wa abambo wa kuchipatala?
4. Mukuganiza kuti pali zolepheretsa kapena nkhawa zanji pa kuphatikizana kwa kulera ndi Mdulidwe wa abambo wa kuchipatala?

1. Mukuganiza kuti pachitike chiani kuti nkhawa ndi zolepheretsazi zithetsedwe?

**4.0 Kuunika Khansa ya khomo la chiberekero kwa wokondedwa awo amai**

1. Tandifotokozereni kuti chimachitika ndi chiyani amai akafuna kuyezetsa khansa ya khomo lachibelekero?
2. Inuyo monga wothandiza anzanu, mukuganizapo bwanji pa nkhani ya kuphatikiza thandizo la kuunika khansa ya khomo la chiberekero kwa amai ndi thandizo la mdulidwe wa abambo wa kuchipatala?

**Probes:**

- 1. *Ndichiyani chimene chingakusangalatseni pa mphatikizo wa thandizo la kuunika khansa ya khomo la chiberekero kwa wokondedwa wao ndi mdulidwe wa abambo wa kuchipatala?*
  2. *Ndichiyani chimene chingakupatseni kusafuna mphatikizo wa thandizo la kuunika khansa ya khomo la chiberekero kwa okondedwa awo ndi mdulidwe wa abambo wa ?*

1. Mukuganiza kuti ndi njira ziti zabwino zoperekera thandizo la kuunika khansa ya khomo la chiberekero kwa wokondedwa awo pa kiliniki ya mdulidwe wa bamboo wakuchipatala?
2. Mukuganiza kuti pangakhale zolepheretsa kapena nkhawa zanji pa kuphatikiza thandizo la kuunika khansa ya khomo la chiberekero kwa okondedwa awo ndi Mdulidwe wa abambo wa kuchipata?
3. Mukuganiza kuti nkhawa ndi zoletsazi zingathetsedwe bwanji?

**5.0 Tsopano tiyeni tikambirane za PrEP**

Munayamba mwamvako zokhudza PrEP

Ngati munamvako zokhudza PrEP,Chonde ndiuzeniko zimene mukudziwa zokhudza PrEP?

- - 1. Munadziwa bwanji za zimenezi?
    2. Mumamva bwanji mtimamu za zimenezi?

**Ngati simunamvepo za PrEP**, ndikufotokozerani mmene mankhwala amagwirira ntchito. PrEP ndi mankhwala wothandiza ku HIV amene amapangitsa anthu amene alibe HIV kuti asatengere. Pali pilitsi limodzi limene limamwedwa tiku lililonse ndipo umamwa nthawi ndi nthawi. Ngati mukumwa mwandondomeko amagwira ntchito bwino kwambiri kuteteza anthu kutengera kachilombo ka HIV.

**Tsopano mukumva bwanji za PrEP?**

Kodi nkofunika kuti PrEP akhale akupezeka kwa amai ndi abambo amene alibe kachirombo ka HIV?

Ngati ai ; Nkhawa zanu ndizotani?

Ngati inde,

1. Muli ndi zifukwa zanji zimene mungalimbikitsire anthu kumwa PrEP
2. Ngati PrEP akhale akupezeka, maganizo anu ndi otani pa nkhani yomuphatiza mundondomeko ya thandizo la mdulidwe wa abambo wakuchipatala? Mungatsangalatsidwe kuwauza anthu kulandira nawo?
3. Mukuganiza kuti PrEP angamaperekedwe bwanji pa kiliniki ino yamudulidwe wa ababmbo wakuchipatala?
4. Mukuganiza kuti pali zolepheretsa ndi nkhawa zanji pa kuphatikiza thandizo la PrEP ndi ndondomeko ya mdulidwe wa abambo wa kuchipatala?

5. Mukuganiza pakuyenera kuchitika chiyani kuti tithetse zolepheretsa ndi nkhawazi?

**6.0 Zithandizo zina**

Mutati mwapatsidwa mphamvu zosankha zithandizo zimene zitha kuphatizana ndi thandizo la mdulidwe wa abambo wa kuchipatala,inu mungasankhe kuphatiza ndi zithandizo ziti?

1. Ndifotokozereni zifukwa zake pa zimene mwasankha.
2. Mukuganiza kuti thandizoli liziperekedwa bwanji pa kiliniki ino?

Zikomo kwambiri chifukwa chotenga nthawi yanu kukambirana zinthuzi lero. Mayankho anu athandiza popititsa patsogolo thandizo la zaumoyo muma kiliniki a mdulidwe wa abambo wa kuchipatala.

Tisanatseke, muli ndi chinachirichonse mukufuna kulankhulapo?

Zikomo kwambiri chifukwa cha nthawi yanu pokambirana nane.

**Peers and Clinic aids interview guide**

Thank you for taking the time to talk with me today. I would like to ask you some questions today about the way you feel and what you think about some issues related to the service you provide and how we can include other services in Voluntary Medical Male Circumcision (VMMC) clinics. There are no right or wrong answers to these questions. We would like to hear your opinion and your experiences in your own words. Do you have any questions before we begin?

**Can you tell me how you are involved in the client care at this clinic?**

**Probes**

Do your **peers** talk to you about how the services are provided here?

Can you give me an example of a time that your **Peer** talked to you about the services he received here?

1.0 **Couple HIV Testing and Counselling**

**Now let us talk about partner HIV testing here at the Voluntary Medical Male** **circumcision clinic?**

1. Tell me what happens if a man brings a spouse here at the Voluntary Medical Male circumcision clinic
2. What do you think are the motivators that make the men bring their spouses here for testing?
3. What do you think demotivates men to bring their partners here for HIV counselling and testing?
4. What do you think can be done to make men bring their partners here for couple testing and counselling?
5. You as a peer, what is your opinion on integrating couple counseling with Voluntary Medical Male circumcision services.
6. What do you think are the barriers and concerns on this integration?
7. What do you think can be done to overcome these concerns and barriers to couple counseling in Voluntary Medical Male circumcision services clinic?

**Now I would like to discuss with you about sexual reproductive health services and Pills HIV prevention: called pre-exposure prophylaxis. (PrEP). Sexual reproductive health includes services that promote good sexual health and reproduction. They include but not limited to family planning, sexual transmitted infection management, cervical cancer screening, Condom distribution and many more.**

**Today we will only discuss about family planning, diagnosis and management of STIs, Cervical cancer screening, and PrEP. We will look at each of these one by one. Let us start with:**

**2.0, STI SERVICES**

1. Explain to me what happens if a client is suspected or diagnosed with an STI here?
2. You as a peer what is your opinion on integrating STI services with circumcision services.

**Probes**:

- 1. *What is it that you would like about the integration of diagnosis and management STIs with Voluntary Medical Male Circumcision services*
  2. *What is it that you would not like the integration of diagnosis and management STIs with* Voluntary Medical Male *Circumcision services*

1. How do you think STI services should be offered at the Voluntary Medical Male circumcision clinic?
2. What do you think are the barriers and concerns on this integration?
3. What do you think should be done to address these concerns and barriers?

**3.0 Family planning**

1. Explain to me what happens if a client needs a family planning methods (vasectomy for men and family planning for female partners?
2. As a peer, what is your opinion on integrating Family planning in circumcision services?

**Probes**:

1. *What is it that you would like about the integration of Family planning with Voluntary Medical Male Circumcision services?*
2. *What is it that you would not like about the integration of family planning with* Voluntary Medical Male *Circumcision services?*

3. How do you think family planning services would be offered within Voluntary Medical Male circumcision clinics?

4. What do you think are the barriers and concerns with the integration family planning and

Voluntary Medical Male circumcision services?

5. What do you think should be done to address these concerns and barriers?

**4.0 Cervical cancer screening for female partners**

1. Explain to me what happens if a woman needs cervical cancer screening?

2. As a peer, what is your opinion on integrating cervical cancer screening for female partners with Voluntary Medical Male Circumcision services?

**Probes:**

1. *What is it that you would like about partner’s cervical cancer screening integration with* Voluntary Medical Male *Circumcision services.*
2. *What is it that you would not like about the integration of partner cervical cancer screening with* Voluntary Medical Male *Circumcision services?*

3. How do you think is the best way to offer cancer screening within Voluntary Medical Male circumcision clinics?

4. What do you think are the barriers and concerns about integrating partner cervical cancer

screening and Voluntary Medical Male Circumcision services?

5. What do you think should be done to address these concerns and barriers?

**5.0 Now let us discuss about PrEP**

**Have you heard about this before?**

If you have heard about PrEP, please tell me what you know about PrEP?

- - 1. How did you learn about this?
    2. How do you feel about this?

If you have not heard about PrEP, I will explain how the medicine works. PrEP is anti-HIV medicine that keeps HIV-negative people from being infected. There is a single pill that is taken once daily, and if you take it regularly, it is highly effective at prevention people from being infected.

Now, how do you feel about PrEP?

**If PrEP was made available to HIV- men and women.**

**Do you think you could advise your HIV negative peers to accept to take PrEP?**

If No: what are your concerns?

If yes,

1. What are the reasons you would encourage your clients to take PrEP?
2. If PrEP becomes available, what is your opinion on integrating PrEP with Voluntary Medical Male circumcision services? Would you encourage clients to take PrEP?
3. How do you think PrEP would be offered here at Voluntary Medical Male Circumcision clinic?
4. What do you think could be the concern and barriers to integrating PrEP Voluntary Medical Male Circumcision services?
5. What do you think should be done to address these concerns and barrier?

**6.0 Other services**:

If you were given powers to choose and integrate services in Voluntary Medical Male Clinics, what are the services that you would think of to Integrate?

1. Explain to me what the reasons are for your choices.
2. How do you think these services would be offered in the clinic?

Thank you for taking your time to discuss with me today. Your answers will be very helpful in improving the health service delivery at Voluntary Medical Male circumcision clinics.

Before we close, do you have anything to say?

Again thank you so much.

**Service providers interview guide**

Zikomo potenga nthawi kulankhula ndi ine lero. Ndikufuna ndikufunseni mafunso ena okhudza ndi mmene mukumvera/mukuganizira pa zinthu zina zokhudzana thandizo limene mumapereka ndi mmene mukuganizala pa za kuonjezera thandizo lina pamwamba pa thandizo la mdulidwe wa abambo wa kuchipatala. Palibe yankho lokhoza kapena lolakwika pa mafunsowo. Tikufuna timve maganizo anu ndi pakamwa panu. Muli ndi mafunso ena alionse tisanayambe?

**Mungandiuzeko kuti inuyo mumatengapo mbali yanji kwa olandira thandizo limene limaperekedwa pa kiliniki ino?**

**Probes**

1. *Kodi anthu olandira thandizo pano amatha kulankhula nanu za thandizo limene limaperekedwa pa kiliniki ino?*

*Mungandiuzeko chitsanzo chimodzi pamene wolandira thandizo analankhula nanu za thandizo analandira kuno.*

**1.0 Kuyezetsa HIV ngati Banja**

**Tsopano tiyeni tikambirane za kuyezetsa HIV ngati banja pa kiliniki ino ya mdulidwe wa abambo wa kuchipatala?**

1. Tandifotokozerani, kodi chimachitika ndi chiyani abambo akabweretsa wokondedwa wao kuno ku kiliniki ya mdulidwe wa kuchipatala?
2. Mukuganiza kuti, chimawapangitsa ndichiani kuti abambo abweretse wokondedwa awo kudzayezetsa HIV?
3. Kwa abambo amene sabweretsa wokondedwa awo, mukuganiza kuti chimapangitsa ndichiyani kuti asawabweretse wokondedwa awo kuno kudzayesetsa HIV?
4. Mukuganiza kuti tipange chiyani kuti abambo athe kubweretsa wokondedwa awo kudzayezetsa ngati banja?
5. Monga inu wopereka thandizo lachipatala, mukuganizapo bwanji pa zophatikizapo kuyezetsa HIV ngati banja pa ndondomeko ya thandizo la mduluidwe wa abambo wa kuchipatala?
6. Mukuganiza kuti pangakhale zolepheretsa kapena nkhawa zanji pa ndondomeko ya kuphatikiza thandizoli.
7. Mukuganiza kuti pakuyenera kuchitika chiyani kuti tithetse nkhawa ndi zolepheretsa za kuyezetsa ngati banja ku kiliniki ya mdulidwe wa abambo wa kuchipatala?

**Tsopano ndikufuna kuti tikambirane zokhudzana ndi thandizo la uchembere ndi mapilitsi woteteza ku HIV wotchedwa kuti PrEP**

**Uchembere wabwino ukuthandauza chithandizo chimene chikuphatikizapo kugonana komanso kubereka/kuchembeza kwabwino. Zikuphatikizapo koma wosalekezera pomwepa kulera, kuunika khansa ya khomo la chiberekero, kuunika ndi kuthandiza matenda wopatsirana pogonana, kugawa makondomu ndi zina zambiri.**

**Lero tingokambirana za Kulera, kuunika ndi thandizo la matenda wopatsirana pogonana, kuunika khansa ya khomo la chiberekero ndi PrEP. Tikambirana chilichonse pachokha pachokha: Tiyeni tiyambe ndi:**

1. **Kuunika ndi thandizo la matenda wopatsirana pogonana**
2. Tandilongosolereni kodi chimachitika ndi chiyani mukamuganizira kapena mwapeza kuti munthu ali ndi matenda opatsirana pogonana kuno?
3. Monga inu wopereka thandizo la chipatala, mukuganizapo bwanji pa zophatikiza kuunika ndi kupereka thandizo la matenda opatsirana pogonana ndi thandizo la mduluidwe wa abambo wa kuchipatala?

**Probes**:

- 1. *Nanga ndi zinthu zanji zimene mukugwirizana nazo pankhani yakuphatikizana thandizo la matenda wopatsirana pogonana ndi mdulidwe wa abambo wa kuchipatala?*
  2. *Ndi zinthu zanji zimene simukugwirizana nazo pankhani yakuphatikiza thandizo la matenda wopatsirana pogonana ndi mdulidwe wa abambo wa kuchipatala?*

1. Kodi mukuona kuti ndi njira ziti zabwino zoperekera thandizo la matenda wopatsirana pogonana kuno ku kiliniki ya mdulidwe wa abambo wa kuchipatala
2. Kodi mukuganiza kuti pali zolepheretsa komanso nkhawa zanji pa kuphatikizana kwa thandizo la matenda wopatsirana pogonana ndi mdulidwewa abambo wa kuchipatala?
3. Mukuganiza kuti nkhawa ndi zolepheretsazi zingathetsedwe bwanji?
4. **Kulera**
5. Tandifotokozereni kuti chimachitika ndi chiani munthu akafuna njira yolera (kutseka kwa abambo ndiponso kulera kwa amai).
6. Monga inu wopereka thandizo la chipatala, mukuganizapo bwanji pa nkhani yakuti aphatikizepo kulera pa thandizo la mdulidwe wa abambo wa kuchipatala?

Probes;

- 1. *Kodi zimene zingakutsangalatseni kuti thandizo la kulera liphatikizane ndi thandizo la mdulidwe wa abambo wa kuchipatala ndi chiyani?*
  2. *Ndichiyani chimene chingakupatseni kusafuna mphatikizo wa thandizoli mu ndondomeko ya mdulidwe wa abambo wa kuchipatala?*

1. Mukuganiza kuti ndi njira ziti zabwino zoperekera thandizo la kulera muno mu kiliniki ya mdulidwe wa abambo wa kuchipatala?
2. Mukuganiza kuti pali zolepheretsa kapena nkhawa zanji pa kuphatikizana kwa kulera ndi Mdulidwe wa abambo wa kuchipatala?

1. Mukuganiza kuti nkhawa ndi zolepheretsazi zingathetsedwe bwanji?

**4.0 Kuunika Khansa ya khomo la chiberekero kwa amai**

1. Tandifotokozereni kuti chimachitika ndi chiyani amai akafuna kuyezetsa za khomo lachiberekero
2. Monga inu wopereka thandizo la chipatala, muli ndi maganizo anji pa kuphatikiza thandizo la kuunika khansa ya khomo la chiberekero ndi thandizo la mdulidwe wa a bamboo wa kuchipatala?

**Probes:**

- 1. *Ndichiyani chimene chingakusangalatseni pa mphatikizo wa thandizo la kuunika khansa ya khomo la chiberekero ndi thandizo la mdulidwe wa abambo wa kuchipatala?*
  2. *Ndichiani chimene chingakupangeni kusafuna mphatikizo wa thandizo la kuunika khansa ya khomo la chiberekero ndi thandizo la mdulidwe wa abambo wa kuchipatala?*

1. Mukuganiza kuti ndi njira ziti zabwino zoperekera thandizo la kuunika khansa ya khomo la chiberekero kwa wokondedwa awo mu kiliniki ya mdulidwe wa abambo wa kuchipatala?
2. Mukuganiza kuti pangakhale zoletsa kapena nkhawa zanji pa kuphatikiza thandizo la kuunika khansa ya khomo la chiberekero kwa okondedwa awo ndi mdulidwe wa abambo wa kuchipatala?
3. Mukuganiza kuti nkhawa ndi zolepheretsatsazi zingathetsedwe bwanji?

**5.0 Tsopano tiyeni tikambirane za PrEP**

Munayamba mwamvako zokhudza PrEP

Ngati munamvako zokhudza PrEP,Chonde ndiuzeniko zimene mukudziwa zokhudza PrEP?

1. Munadziwa bwanji za zimenezi?
2. Mumamva bwanji mtimamu za zimenezi?

**Ngati simunamvepo zokhudza PrEP**, ndikufotokozerani mmene mankhwala amagwirira ntchito. PrEP ndi mankhwala wothandiza ku HIV amene amapangitsa anthu amene alibe HIV kuti asatengere. Pali pilitsi limodzi limene limamwedwa kamodzi tsiku lililonse ndipo ukamamwa nthawi ndi nthawi mwa mwandondomeko limagwira ntchito bwino kwambiri poteteza anthu kutengera kachirombo.

**Tsopano mukumva bwanji zokhudza PrEP?**

Kodi Kukanakhala kuti PrEP akupezeka kwa amai ndi abambo amene alibe kachirombo ka HIV, mukanawalimbikitsa anthu kuti alandire nawo?

Ngati ai; Nkhawa zanu ndizotani?

Ngati inde,

1. Muli ndi zifukwa zanji zimene mungalimbikitsire anthu kumwa nawo PrEP?
2. Ngati PrEP akhale akupezeka, maganizo anu ndi otani pa nkhani yomuphatikiza mundondomeko ya thandizo la mdulidwe wa abambo wa kuchipatala? Mungatsangalatsidwe kuwauza anthu kulandira nawo?
3. Mukuganiza kuti PrEP angamaperekedwe bwanji pa kiliniki ino ya mdulidwe?
4. Mukuganiza kuti pali zolepheretsa ndi nkhawa zanji pa kuphatikiza thandizo la PrEP ndi ndondomeko ya mdulidwe wa abambo wa kuchipatala
5. Mukuganiza pakuyenera kuchitika chiani kuti tithetse zolepheretsa ndi nkhawazi?

**6.0 Zithandizo zina**

Mutati mwapatsidwa mphamvu zosankha zithandizo zimene zitha kuphatizana ndi thandizo la mdulidwe wa abambo wa kuchipatala inu mungasankhe kuphatikiza ndi zithandizo ziti?

1. Ndifotokozereni zifukwa zake pa zimene mwasankha.

2. Mukuganiza kuti thandizoli liziperekedwa bwanji mu kiliniki?

Zikomo kwambiri chifukwa chotenga nthawi yanu kukambirana zinthuzi lero. Mayankho anu athandiza popititsa patsogolo thandizo la zaumoyo mu makiliniki a mdulidwe wa abambo wa kuchipatala.

Tisanatseke, muli ndi chinachirichonse mukufuna kulankhulapo?

Zikomo kwambiri chifukwa cha nthawi yanu pokambirana nane.

**Service providers Interview guide**

Thank you for taking the time to talk with me today. I would like to ask you some questions today about the way you feel and what you think about some issues related to the service you provide and how we can include other services in Voluntary Medical Male Circumcision clinics. There are no right or wrong answers to these questions. We would like to hear your opinion and your experiences in your own words. Do you have any questions before we begin?

**Can you tell me how you are involved in the client care at this clinic?** **Probes**

Do your clients talk to you about how the services are provided here?

Can you give me an example of a time that your client talked to you about the services he received here?

**1.0 Couple HIV Testing and Counselling**

**Now let us talk about partner HIV testing here at the** Voluntary Medical Male **circumcision clinic?**

1. Tell me what happens if a man brings a spouse here at the Voluntary Medical Male circumcision clinic
2. What do you think are the motivators that make the men bring their spouses here for testing?
3. For those who do not bring their partners what do you think demotivates men to bring their partners here for HIV counselling and testing
4. What do you think can be done to make men bring their partners here for couple testing and counselling?
5. You as a health care provider, what is your opinion on integrating couple counseling with Voluntary Medical Male circumcision services.
6. What do you think are the barriers and concerns on this integration?
7. What do you think can be done to overcome these concerns and barriers to couple counseling in Voluntary Medical Male circumcision services clinic?

**Now I would like to discuss with you about sexual reproductive health services and Pills for HIV prevention: called pre-exposure prophylaxis. (PrEP)**

**Sexual reproductive health includes services that promote good sexual health and reproduction. They include but not limited to family planning, cervical cancer screening sexual transmitted infection (STI) management, and many more.**

**Today we will only discuss about family planning, diagnosis and management of STIs, Cervical cancer screening, and PrEP. We will look at each of these one by one. Let us start with:**

**2.0, STI SERVICES**

1. Explain to me what happens if a client is suspected or diagnosed with an STI here?
2. You as a health care provider what is your opinion on integrating STI services with Voluntary Medical Male circumcision services.

**Probes:**

1. *What is it that you do not like the integration of STI with Voluntary* Medical Male *Circumcision services?*
2. *What is it that you like of the integration STI services with* Voluntary Medical Male *Circumcision services****?***
3. How do you think STI services should be offered at the Voluntary Medical Male circumcision clinic?
4. What do you think are the barriers and concerns on this integration?
5. What do you think should be done to address these concerns and barriers?

**Family planning**

1. Explain to me what happens if a client needs a family planning methods (vasectomy for men and family planning for female partners?
2. As a health care provider, what is your opinion on integrating Family planning in circumcision services

**Probes:**

1. *What is it that you would not like the integration of family planning in* Voluntary Medical Male *Circumcision Clinic?*
2. *What is it that you like of the integration of Family planning with* Voluntary Medical Male *Circumcision services***?**
3. How do you think family planning services can be offered within Voluntary Medical Male circumcision clinics?
4. What do you think are the barriers and concerns with this integration?
5. What do you think should be done to address these concerns and barriers?

**Cervical cancer screening**

1. Explain to me what happens if a woman needs cervical cancer screening?

2. As a health care provider, what is your opinion on integrating cervical cancer screening in Voluntary Medical Male Circumcision services

**Probes:**

- 1. *What is it that you would not like about cervical cancer screening integration with* Voluntary Medical Male *Circumcision services?*
  2. *What is it that you like of the integration cervical cancer screening with* Voluntary Medical Male *Circumcision services?*

3. How do you think is the best way to offer cancer screening within Voluntary Medical Male circumcision clinics?

4. What do you think are the barriers and concerns on this integration?

5. What do you think should be done to address these concerns and barriers?

**Now let us discuss about PrEP**

**Have you heard about this before?**

If you have heard about PrEP, please tell me what you know about PrEP?

1. How did you learn about this?
2. How do you feel about this?

If you have not heard about PrEP, I will explain how the medicine works. PrEP is anti-HIV medicine that keeps HIV-negative people from being infected. There is a single pill that is taken once daily, and if you take it regularly, it is highly effective at prevention people from being infected.

Now how do you feel about PrEP?

**If PrEP was made available to HIV- men and women.**

**Do you think you could advise your HIV negative clients to accept to take PrEP?**

If No: what are your concerns?

If yes,

1. What are the reasons you would encourage your clients to take PrEP?
2. If PrEP becomes available, what is your opinion on integrating PrEP with Voluntary Medical Male circumcision services? Would you encourage clients to take it?
3. How do you think PrEP be offered in Voluntary Medical Male clinics?
4. What do you think are the concern and barriers to integrating PrEP in Voluntary Medical Male services?
5. What do you think should be done to address these concerns and barrier?

**Other Services**:

If you were given powers to choose and integrate services in Voluntary Medical Male Clinics, what are the services that you would think of Integrate?

1. Explain to me what the reasons are for your choices.
2. How do you think these services should be offered in the clinic?

Thank you for taking your time to discuss with me today. Your answers will be very helpful in improving the health service delivery at Voluntary Medical Male circumcision clinics.

Before we close, do you have anything to say?

Again, thank you so much for taking your time to speak with me.
